# Predicted Metabolic Pathway Distributions in Stool Bacteria in Very-Low-Birth-Weight Infants: Potential Relationships with NICU Faltered Growth

**DOI:** 10.3390/nu12051345

**Published:** 2020-05-08

**Authors:** Maureen Groer, Elizabeth M. Miller, Anujit Sarkar, Larry J. Dishaw, Samia V. Dutra, Ji Youn Yoo, Katherine Morgan, Ming Ji, Thao Ho

**Affiliations:** 1 College of Nursing, University of South Florida, Tampa, FL 33612, USA; anujit@usf.edu (A.S.); samiavaleria@usf.edu (S.V.D.); jiyounyoo@usf.edu (J.Y.Y.); mji@usf.edu (M.J.); 2 Department of Anthropology, University of South Florida, Tampa, FL 33620, USA; emm3@usf.edu; 3 College of Public Health, University of South Florida, Tampa, FL 33612, USA; 4 Morsani College of Medicine, Department of Pediatrics, University of South Florida, Tampa, FL 337013, USA; ldishaw@usf.edu (L.J.D.); tho1@usf.edu (T.H.); 5 College of Nursing, University of Tennessee, Knoxville, TN 37996, USA; kmorgan3@utk.edu

**Keywords:** dysbiosis, gut microbiota, gut microbiome metabolic pathway, very low birth weight, growth, NICU

## Abstract

Many very-low-birth-weight (VLBW) infants experience growth faltering in early life despite adequate nutrition. Early growth patterns can affect later neurodevelopmental and anthropometric potentials. The role of the dysbiotic gut microbiome in VLBW infant growth is unknown. Eighty-four VLBW infants were followed for six weeks after birth with weekly stool collection. DNA was extracted from samples and the V4 region of the 16S rRNA gene was sequenced with Illumina MiSeq. A similar microbiota database from full-term infants was used for comparing gut microbiome and predicted metabolic pathways. The class Gammaproteobacteria increased or remained consistent over time in VLBW infants. Out of 228 metabolic pathways that were significantly different between term and VLBW infants, 133 pathways were significantly lower in VLBW infants. Major metabolic differences in their gut microbiome included pathways involved in decreased glycan biosynthesis and metabolism, reduced biosynthetic capacity, interrupted amino acid metabolism, changes that could result in increased infection susceptibility, and many other system deficiencies. Our study reveals poor postnatal growth in a VLBW cohort who had dysbiotic gut microbiota and differences in predicted metabolic pathways compared to term infants. The gut microbiota in VLBW infants likely plays an important role in postnatal growth.

## 1. Introduction

A common outcome in very-low-birth-weight infants (VLBW infants (weight < 1500 g)) is postnatal growth failure. Significant efforts during Neonatal Intensive Care Unit (NICU) hospitalization are directed towards nutrition, with provision of nutrients and calories calculated to promote the growth that matches intrauterine growth for both length and weight. Equivalence to intrauterine growth is seldom achieved postnatally due to multiple reasons that are known and postulated [1,2]. The acute issues, such as cardiorespiratory conditions, are prioritized in medical management and they can be barriers to the optimization of nutrition. Fluid restriction to reduce other co-morbidities, such as chronic lung disease and patent ductus arteriosus, can limit ways to provide adequate calories [3]. The calculations of fetal energy requirement do not account for increased caloric utilization from stress and acute and chronic illnesses. These calculations also do not account for the potentially complex roles that maternally derived microbial metabolites serve in shaping neonatal development in utero [4,5], or the potential role of gut microbial dysbiosis in postnatal growth in VLBW infants. The infant microbiome plays important roles in shaping gut development both directly and indirectly [6,7]. We have previously reported that microbial diversity and volatility were positively associated with improved growth in the VLBW infant [8]. The gut microbiota as a potential source of this growth faltering has been observed in recent years but we need more evidence to clarify the underlying mechanisms of this association.

The ultimate goal of optimizing nutrition for growth is to reduce the long-term complications and achieve better outcomes for VLBW infants. The common quantitative measurements of growth in the Neonatal Intensive Care Units (NICUs) are serial weights, lengths, and head circumferences. The most commonly used calculations of weight gain are g/kg/day, g/day, and z-score relative to a growth chart [9]. Z-scores are standard deviation measurements of individuals compared to a reference population of a given sex and age, allowing for sex-independent comparisons to be made across different ages in population-based studies. Preterm infants often do not track along their z-score, with a smaller head circumference, weight and length for age at discharge. This faltered growth is associated with increased total body fat mass at term-equivalent age when compared to infants born at term [10].

A potential mechanism for this faltered growth may be microbial dysbiosis. *Enterobacteriaceae* are the most common family of Gammaproteobacteria in VLBW infants, and these bacteria are unable to digest human milk oligosaccharides HMOs, which represent 20% of the carbohydrate content of milk [11]. The *Enterobacteriaceae* are facultative anaerobes and are capable of saccharolytic fermentation but they will switch to proteolytic fermentation when carbohydrates are depleted. They use mixed acid fermentation anaerobically if there are available electron acceptors, such as nitrate or fumarate. If those electron acceptors are not available, they will ferment glucose to acetate, succinate, lactate, or formate. They produce less adenosine triphosphate (ATP) through metabolic pathways compared to *Bifidobacteria* [12]. The lack of butyrate production by *Enterobacteriaceae*, along with their production of proinflammatory LPS, may alter the enteric barrier and account for poorer colonic absorption of nutrients by the preterm infant [13].

We hypothesized that the Enterobacteriaceae dominant dysbiosis we have observed in VLBW infants plays a metabolic role in the faltered infant growth we have previously reported [8]. This dysbiosis does not allow for the adequate metabolism of human milk, so that energy harvest and utilization for infant growth is impaired. In addition, the nature of this infant gut microbial ecology and their deficiencies in metabolic by-products, such as short-chain fatty acids (SCFAs) and vitamins, may further impair digestion and absorption [14]. As a first step in analyzing the potential metabolic effects of the VLBW infant’s dysbiotic gut on growth, we compared the gut microbiota and the predicted metabolic pathways between a cohort of VLBW infants and a group of term infants with comparable demographics.

## 2. Materials and Methods

### 2.1. Participants

Participant recruitment of VLBW infants was done in a single NICU. Out of a pool of 220 admissions over the year and a half of recruitment, parents of 84 infants agreed to participate in the study (38%). Parents provided written informed consent and the study was approved by the hospital and university Institutional Review Boards (Pro00019955). The study was conducted in accordance with the Declaration of Helsinki. The exclusion criteria included HIV exposure, congenital anomalies, and moribund status. A comparable set of microbiome data of full-term infants from a previous study was used for comparison [15]. Feeding types were recorded and feeding compositions by volume were calculated daily. Fecal samples were obtained from infant diapers at least once a week, and stored at -80°C until processing for DNA extraction for 16S rRNA gene amplicon sequencing. Clinical data for the study (pregnancy and birth history, infant medications, complications, procedures, and outcomes) were extracted from the electronic medical record (EMR). Additional information about pregnancy, labor, and maternal characteristics were collected by questionnaire. Length, weight, and head circumference were measured weekly. These measures were transformed to z-scores using the Fenton growth charts [9].

### 2.2. Stool Sample Processing

DNA was extracted using the QIAamp PowerFecal DNA Kit (Qiagen, Carlsbad, CA, USA) with modifications based on the Earth Microbiome Project protocols (www.earthmicrobiome.org). Library preparation and sequencing methods for all the VLBW infants were as previously described [8]. Samples with less than 2000 reads were discarded from further analysis. In order to compare the gut microbiome of these VLBW infants with appropriate controls, FASTQ files from term infants from a previous study [15] were downloaded and analyzed together with the VLBW infants’ microbiome data. These control samples were targeted for the gut microbiome from the V4 region of the 16S rRNA gene and the same sequencing protocol was followed. The number of samples in each group is listed in Table 1. Individual FASTQ files were imported to R (https://cran.r-project.org) and further analyzed with DADA2 package v1.10.1 [16], to determine amplicon sequence variants (ASVs) from the current study.

Briefly, the forward and reverse reads were trimmed by 5 and 10 bases from the end, respectively, and the expected errors was set to 2 for forward and 4 for reverse. The phiX genome was removed and reads with ambiguous bases were discarded. ASVs were called separately from the forward and reverse filtered sequences, which were subsequently merged in the next step. Finally, identified chimeras were removed and the ASVs were classified using the Silva v132 database. The distribution of each ASV in all the samples was determined and their relative abundances were calculated using the DADA2 package in R. Shannon diversity for each sample was calculated using the vegan R package [17].

In order to predict the metagenome of all the samples, the ASV distribution table and the ASV sequences (FASTA format) were uploaded to the Piphillin server [18] and the predictions were made at 97% cutoff. Metabolic pathways were estimated using the latest Kyoto Encyclopedia of Genes and Genomes (KEGG) database version (October 2018). Differences in the distribution of bacterial genera and KEGG pathways across groups were determined by MaAsLin [19]. Principal component analysis (PCA) was performed in R using the prcomp function and was plotted using autoplot function implemented in R package ‘ggfortify’. To identify which pathways were contributed exclusively by the Gammaproteobacteria, we separated the ASV count tables into those that belonged to Gammaproteobacteria and those that did not. We ran the analysis in Piphillin again separately for the two groups.

## 3. Results

### 3.1. Demographics

A total of 84 VLBW infants (gestational age = 28.4 ± 2.4 weeks, birth weight = 1087 ± 218 g) were enrolled in a single NICU located in Tampa, Florida. C-sections were performed for 78.0% of the samples and 48.8% were male. Ethnicity was 20.5% Hispanic, 41.0% African American, and 32.0% Caucasian. All infants were prematurely born except for one small for gestational age infant born at 34 weeks. Feeding types were recorded and feeding compositions by volume were calculated daily. Fecal samples were obtained from infant diapers at least once a week, and stored at −80°C until processed for DNA extraction for 16S rRNA gene amplicon sequencing. Clinical data for the study (pregnancy and birth history, infant medications, complications, procedures, and outcomes) were extracted from the electronic medical record (EMR). Additional information about pregnancy, labor, and maternal characteristics were collected by questionnaire. Length, weight, and head circumference were measured weekly. These measures were transformed to z-scores using the Fenton growth charts [9]. Overall, clinical data were collected for the first six weeks of the NICU admission. For the comparison group of full-term infant, 43.0% of the infants were male and greater than 75% of the samples were from breastfed infants. The majority of infants were Hispanic and from two geographic areas (California and St. Petersburg, Florida), and 64.5% were delivered by vaginal birth. No other demographic data were publicly available for this sample.

### 3.2. Growth of VLBW Infants

**Figure 1:**
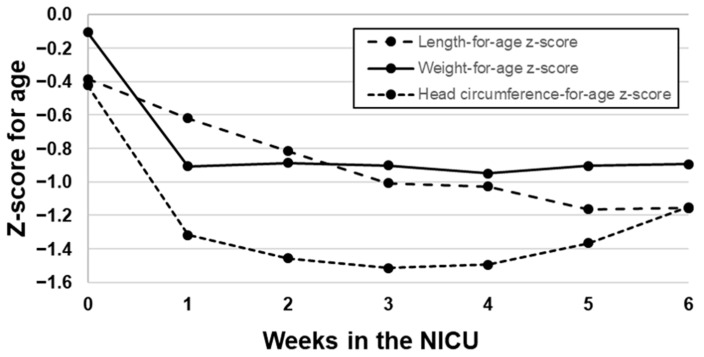
Length-for-age, weight-for-age, and head circumference-for-age Z-scores across a 6-week NICU stay.

On average, the VLBW population of infants were born with a z-score of 0, indicating that their birth weights were near the 50th percentile, on a Fenton growth chart. However, most infants lost weight within the first week but did not regain the birth weight as expected. Weight-for-age z-scores remained consistently almost one standard deviation lower after the initial weight loss. The infants’ length-for-age z-scores slowly and continuously decreased over time and became more stable by week 5 postpartum. Finally, infants lost almost a full standard deviation in head circumference during the first week postpartum, reached the lowest point at week 3, and made some recovery in weeks 5 and 6. Figure 1 depicts the changes in z-scores over time for length, weight, and head circumference.

### 3.3. Analyses Based on Distribution of ASVs

**Figure 2:**
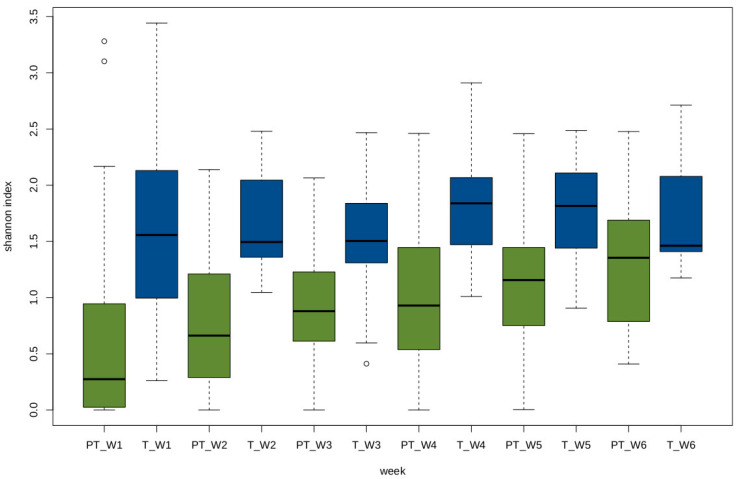
Shannon diversity index for the very-low-birth-weight (VLBW) infants and term infants for the first six weeks. The Shannon index was calculated based on the relative abundance of all the amplicon sequence variants (ASVs) for each sample. The *X*-axis indicates the group (PT: Preterm, T: Term and W: week).

**Figure 3:**
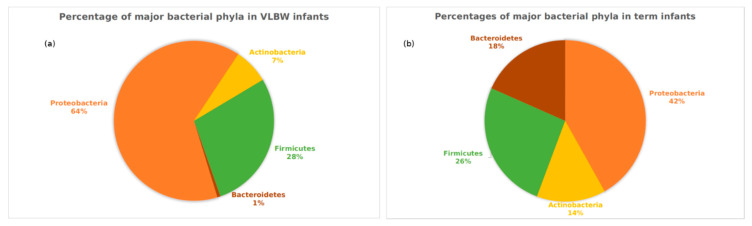
Percentages of four major bacterial phyla in the (**a**) VLBW infants and the (**b**) term infants. The pie charts are made for each group based on the phyla abundance at all time points.

**Figure 4:**
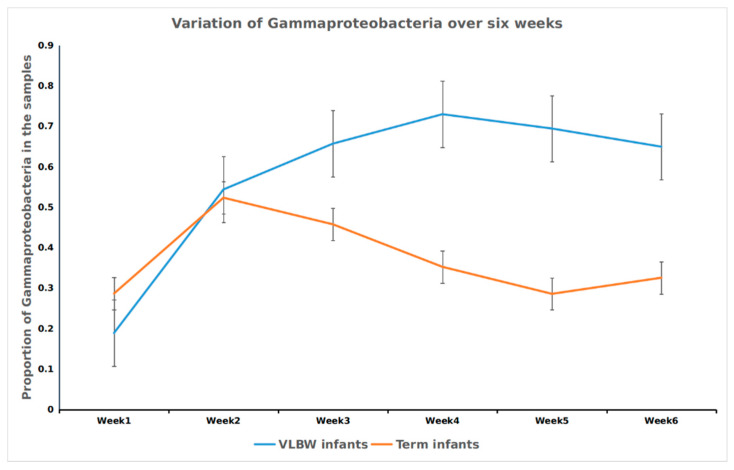
Variation in the proportion of Gammaproteobacteria among VLBW infants and term infants over the first six weeks. While the proportion of this class decreased in term infants over time, its abundance increased and remained consistent in the preterm infants. The *X*-axis indicates the week, while the *Y*-axis shows the relative abundance of Gammaproteobacteria.

The Shannon index, a representation of alpha diversity, increased from week 1 to week 6 in the VLBW infants. Alpha-diversity at week 2 was not significantly higher than week 1 (*p* = 0.08), but the increase was significant for week 3 (*p* = 0.009), week 4 (*p* = 0.005), week 5 (*p* = 0.0003) and week 6 (p=0.0001). No trend was observed for the Shannon index for the first six weeks of life in term infants (Figure 2). The differences among the full term and the VLBW infants were compared at all taxonomic levels based on the ASV distribution and their full classification. All hierarchical levels, which were not clearly identified, were discarded. The two groups had significant differences in the relative abundances of the four major phyla. The Proteobacteria were significantly higher in the VLBW infants (*p* = 7.2E × 10^−8)^ ), while the abundances of Bacteroidetes and Actinobacteria were lower (*p* = 4.5-49, and *p* = 10^−5^, respectively) (Figure 3). Firmicutes were not significantly different between the two groups (*p* = 0.76). Similarly, Gammaproteobacteria class was significantly higher in the VLBW infants (*p* = 1.22 × 10^−11^) while the abundances of Bacteroidia (1.14 × 10^-42^), Alphaproteobacteria (*p* = 5.88 × 10^−10^), and Betaproteobacteria (*p* = 1.14 × 10^−15^) were lower. Overall, 14 classes of bacteria were significantly associated with the VLBW infants differently than with the term infants as shown in Figure S1. It was observed that the class Gammaproteobacteria increased during the first few weeks then remained constant in VLBW infants, while the term infants showed an early increase in the first two weeks followed by a decrease (Figure 4, an effect previously observed by Dogra [20]). Differences between the terms and VLBW infants for the Order and Family taxonomic level were also analyzed and all the results are presented in Figures S2 and S3. The total number of bacteria differed between the VLW infants and term infants. In the VLBW infants. A total of 796 (ASVs) bacteria were observed in VLBWs, while term infants’ bacteria numbered 1827 (ASVs).

The pie chart is based on the relative abundances for all preterm and term infants across all the first six weeks.

### 3.4. Analyses Based on Distribution of Metabolic Pathways

The microbial metagenomes and corresponding KEGG pathways were predicted from all the samples. In this part of the work, the focus was once again on the differences in pathways between the VLBW and the term infants. A total of 279 different metabolic pathways were identified for all the infants. Out of 279, 228 pathways were significantly different between the term and the VLBW infants. Among them, 133 pathways were significantly lower in the VLBW infants, while the rest were higher. All the results are presented in Tables S1 and S2 and Figure S4.

As one of the main objectives of this study was to understand the association of the gut microbiome in VLBW infants with their growth, the top 20 significantly different metabolic pathways were analyzed for differences between VLBW infants and term infants. Table 2 and Table 3 depict these differences by groups of pathways.

Among the predicted pathways, we found that there were three which were specific to the Gammaproteobacteria. They are ko04961 (Endocrine and other factor-regulated calcium reabsorption), ko05130 (Pathogenic Escherichia coli infection) and ko05131 (Shigellosis).

**Figure 5:**
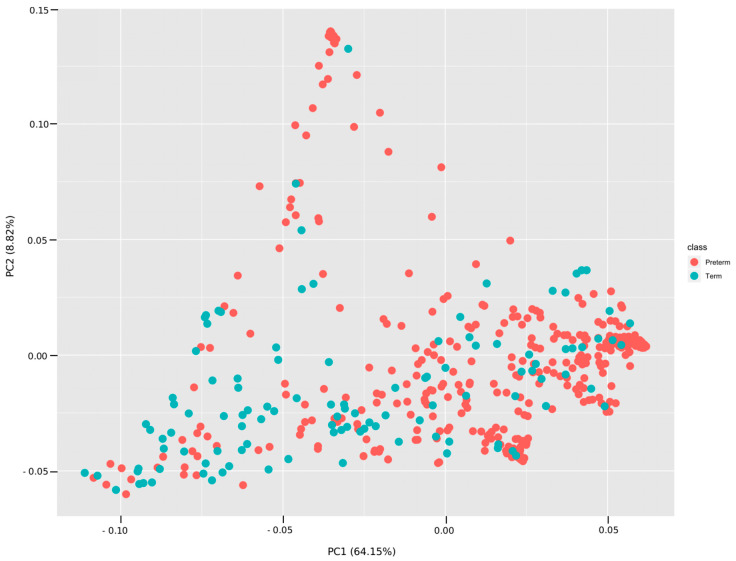
Principal component analysis (PCA) plot among terms and VLBW infants based on the sharing of metabolic pathways. The plot was made based on the pathway abundances for all samples using the prcomp function in R. The first and the second principal components explained 64.15% and 8.82% of the variance.

PCA plots (Figure 5) were constructed based on the common pathways between the VLBW infants and term infants for all the weeks combined (Figure 5) and for each week (see Table S2). The correlation coefficients of PC1 with the KEGG Orthologs (kos) indicate strong correlation (absolute value > 0.8). The correlations of kos with PC2 were small (absolute value < 0.2).

## 4. Discussion

The data indicate very significant differences in taxa, diversity, and predicted metagenomic pathways in VLBW infants compared to term infants. Gut microbiome alpha diversity was much lower in VLBW infants and the taxonomic composition was also very different, with a dominance of Enterobacteriaceae in the VLBW infants. The sources of Gammaproteobacteria, which ultimately dominate the VLBW infant gut, are unknown. Milk has a very substantial percentage of these organisms, so they may play an early role in the immune priming of the gut, but are normally transient in the term infant gut [21]. They may come from the environment, equipment, and personnel. Different NICU rooms contribute to the differences in microbial signatures in VLBW patients [22].

These facultative anaerobes remain dominant in the VLBW infant gut due to many factors including the immaturity of the GI tract and the inability to produce an anaerobic environment in the colon. Facultative anaerobic microbial abundance is potentially perpetuated by the depletion of butyrate, which is normally formed through interactions of *Bifidobacteria* and Firmicutes, both low in abundance in the VLBW infants. Butyrate is a product of bacterial fermentation. Upon the fermentation of HMOs and sugars, *Bifidobacteria* produce, in addition to lactic acid, acetate, which can cross-feed other Firmicutes, resulting in the production of butyrate [23]. Butyrate is the preferred energy source for colonocytes, and these cells sense butyrate by using the nuclear and peroxisome receptor, PPAR- γ. When PPAR-γ is activated, inducible nitric acid syntheses is suppressed and nitric oxide and nitrate are reduced, driving the cell to use oxygen as an electron receptor, and promoting beta oxidation as a source of energy for these highly metabolic colonocytes [24]. If butyrate is reduced as in Enterobacteriaceae dysbiosis, oxygen consumption is reduced in these cells and this perpetuates the further growth of the facultative anaerobes. A net effect of this dysbiosis is decreased mucus thickness, lower levels of particular mucus-associated T cells, increased enteric leakiness, and ultimately inflammation [25,26]. 

The VLBW infants in our study revealed a drop in both weight and height z- scores during the first 6–8 weeks postnatal age, and many were discharged home with faltered growth, even though they were provided the appropriate standard or higher daily caloric intake for VLBW infants. The dysbiotic, low propionate producing gut microbiome likely plays an intermediary role on poor postnatal growth in VLBW infants. The metabolic pathways predicted to be significantly decreased in these infants included pathways that likely contribute to energy harvest, fuel utilization and growth. They were also at increased risk for antibiotic resistance and enteric infections. A smaller study of preterm compared to term infants found similar metabolic pathways to be reduced in VLBW infants [27]. A recent metabolomic study of extremely-low-birth-weight infants with growth failure demonstrated elevated serum acylcarnitine, fatty acids, and other byproducts of lipolysis and fatty acid oxidation associated with disrupted gut microbiome maturation [28]. These authors remarked that infants with growth failure had a persistent physiologic state resembling fasting even though they received adequate nutrition for growth. The growth effects may be affected by phases of the gut microbiota succession in VLBW infants [29], a phenomena that needs further investigation.

Gastrointestinal (GI) immaturity impacts both the absorption of nutrients and the tolerance for enteral feeds required for normal growth. Peristalsis in the GI tract and the suck-swallow reflex are impaired by prematurity, which in turn impact feeding behaviors [30]. This immaturity can also impact the developing microbiome [31]. In general, the GI barrier is less developed and less effective and its maturation requires signaling from microbes [26]. In utero, fetal GI development involves significant swallowing of amniotic fluid (AF), particularly during the third trimester. AF provides important bioactive factors required for the maturation of the GI tract [32,33]. Preterm birth interrupts AF exposure, depriving the gut of required developmental factors [32]. Preterm infants are also born with a GI epithelium that produces mucus that is less viscous, reducing the barrier to microbial penetration [34]. In newborns, more so in VLBW infants, the passive transfer of immune factors from mother’s milk is essential for protecting the developing infant, because their GI production of secretory immunoglobulin A (sIgA) is reduced. sIgA helps to recognize and coat virulent microbes [35].

A well-established “gold standard” for VLBW infant nutrition is human milk, preferably from the mother [36], but often human milk feeds are fortified with Human Milk Fortifier (HMF) to meet the nutrient and caloric requirements for optimal growth in preterm infants [37,38,39]. Mother’s own milk (MOM) has the most suitable nutritional components for the VLBW infants because it provides the necessary fatty acid profiles that are difficult to reproduce in fortifiers or preterm infant formulas [40,41,42,43]. The recommended caloric and protein requirements for VLBW infants, and thus the need for fortification, are derived from calculations of fetal growth requirements at various stages of fetal development [37]. Therefore, fortification regimens are designed to help VLBW infants grow at an equivalent rate as a normally developing fetus [39]. To achieve a postnatal growth that is close to that of normal fetal growth, NICUs often implement a standard nutritional guideline on initiation, frequency and volume of advancement, the constituents, and caloric intake for both parenteral and enteral nutrition [44]. The current recommendations from the American Academy of Pediatrics include the use of MOM as first choice and pasteurized donor milk as an alternative when MOM is not available or contraindicated [45]. When neither form of human milk is available, preterm formula should be used. Because human milk alone does not meet the nutritional (mainly protein) or mineral (calcium, phosphorus, and iron) requirements for the normal growth of preterm infants, HMFs are necessary. Commercially available HMFs, bovine-based HMFs or human-milk-based HMFs contain protein, carbohydrate, fat, vitamins, minerals, and electrolytes.

The composition of human milk is well studied, with regards to its immunonutritional composition, the oligosaccharides (HMOs) content, and to some extent, its microbiome [46,47]. Since milk (or formula) is the only type of enteral nutrition these infants receive, the effects on the microbiota may be critical given that the term infant Bifidobacterial-dominated gut microbiota does not exist in most VLBW infants [48,49,50,51]. Often VLBW infants do not receive an exclusive MOM diet, and may have a mixture of MOM, donor milk and formula. Human milk favors the growth of *Bifidobacteria*, a largely commensal genus, but even exclusively breast milk fed VLBW infants do not develop *Bifidobacterial* dominance. Rather, these infants develop a delayed succession of bacterial blooms, and are dominated by Gammaproteobacteria for many weeks while in the NICU [50,51]. The *Bifidobacterial* dominance benefits infants because these microbes have a role in the development of immunity, protect against virulent microorganisms colonizing the gut, and support colonic health through production of SCFAs like butyrate [52]. Butyrate is an SCFA that is essential to colonic epithelial health. These microbes also produce B vitamins [53]. Infant growth faltering could also result from vitamin or mineral deficiency, impaired mucosal absorption and digestive systems, and impaired enzymatic function because of prematurity and pH or oxygenation status changes. When the preterm infant gut becomes dominated by Gammaproteobacteria, the net effect on metabolism may be to reduce the amount of ATP generated in microbial metabolism, to increase protein catabolism for energy production, to decrease beneficial SCFAs, to reduce essential vitamin synthesis, and increase the potential for inflammatory and immune effects in the gut and other systems. 

The current study has several limitations that impact interpretations and the significance of results. The full term infant group was collected separately from the VLBW group, so DNA extraction and sequencing may play a role in results. We do not have corresponding phenotypic data on the term infants. Our data for the VLBW infants only extends to the 6–8th postpartum weeks, so further longitudinal analysis over the entire admission to the NICU is warranted. The metabolic data is entirely based on microbial abundances so it is predictive only and we have not measured metabolites and transcription pathways directly in the stool sample.

## 5. Conclusions

In our VLBW infants, the predicted metabolic pathways from microbiota data showed major reductions in glycan biosynthesis and metabolism and biosynthetic capacity, interruptions in amino acid metabolism, increases in susceptibility to infections, and many other system deficiencies that play a role in normal gut microbiota succession and maturation as well as infant growth and development. Further functional studies are needed to clarify if microbes or particular metabolic pathways play a more direct effect on infant growth, and what interventions can be made, either nutritionally, or that could directly affect the dysbiosis, to improve health and nutrition as these infants grow. A follow-up validation study based on quantitative PCR (qPCR) would help to validate these findings. These preliminary data are the first step in generating hypotheses. In the future, we plan to validate the findings by metagenomics and metatranscriptomics.

## Figures and Tables

**Table 1:** Number of samples incorporated in this study.

Group	Unique IDs	Total Specimen
VLBW Infants	84	375
Term infants	15	112
Total	99	561

**Table 2:** Top 20 pathways significantly lower in VLBW infants compared to term infants. The positive coefficient indicates that the pathways were higher in term infants.

KO Number	Category	Sub-Category	Q-Value	Co-Efficient
ko00511	Glycan biosynthesis and metabolism	Other glycan degradation	3.51 × 10^−29^	0.017720
ko00600	Lipid metabolism	Sphingolipid metabolism	2.68 × 10^−28^	0.016071
ko04142	Transport and catabolism	Lysosome	4.87 × 10^−34^	0.015629
ko00603	Glycan biosynthesis and metabolism	Glycosphingolipid biosynthesis –globo and isoglobo series	1.40 × 10^−26^	0.013687
ko00523	Metabolism of terpenoids and polyketides	Polyketide sugar unit biosynthesis	1.56 × 10^−26^	0.012467
ko01230	Metabolism	Biosynthesis of amino acids	4.04 × 10^−13^	0.011963
ko00521	Biosynthesis of other secondary metabolites	Streptomycin biosynthesis	7.87 × 10^−33^	0.011445
ko00531	Glycan biosynthesis and metabolism	Glycosaminoglycan degradation	5.90 × 10^−30^	0.010446
ko01130	Lipid metabolism	Steroid hormone biosynthesis	4.78 × 10^−13^	0.009016
ko00513	Glycan biosynthesis and metabolism	Various types of N-glycan biosynthesis	5.96 × 10^-23^	0.008769
ko00604	Glycan biosynthesis and metabolism	Glycosphingolipid biosynthesis - ganglio series	6.84 × 10^−23^	0.008754
ko04974	Digestive system	Protein digestion and absorption	2.39 × 10^−26^	0.008621
ko04920	Endocrine system	Adipocytokine signaling pathway	1.79 × 10^−15^	0.007461
ko00460	Metabolism of other amino acids	Cyanoamino acid metabolism	7.62 × 10^−21^	0.007188
ko00525	Biosynthesis of other secondary metabolites	Acarbose and validamycin biosynthesis	1.23 × 10^−15^	0.006739
ko03010	Translation	Ribosome	1.13 × 10^−4^	0.006631
ko01210	Metabolism	2-Oxocarboxylic acid metabolism	7.45 × 10^−13^	0.006483
ko00250	Amino acid metabolism	Alanine, aspartate and glutamate metabolism	1.12 × 10^−18^	0.006323
ko00340	Amino acid metabolism	Histidine metabolism	1.20 × 10^−11^	0.006178
ko00311	Biosynthesis of other secondary metabolites	N-Glycan biosynthesis	4.12 × 10^−38^	0.006095

**Table 3:** Top 20 pathways significantly higher in VLBW infants compared to term infants. The negative coefficient indicates that the pathways were higher in preterm infants.

KO Number	Category	Sub-Category	Q-Value	Co-Efficient
ko02020	Signal transduction	Two-component system	9.9 × 10^−20^	−0.02705
ko02060	Membrane transport	Phosphotransferase system	1.1 × 10^−27^	−0.02545
ko02040	Cell motility	Flagellar assembly	2.3 × 10^−8^	−0.02141
ko02026	Cellular community—prokaryotes	Biofilm formation—Escherichia coli	5.6 × 10^−7^	−0.01179
ko00130	Metabolism of cofactors and vitamins	Ubiquinone and other terpenoid-quinone biosynthesis	5.8 × 10^−18^	−0.01034
ko01503	Drug resistance: antimicrobial	Cationic antimicrobial peptide (CAMP) resistance	4.2 × 10^−13^	−0.01026
ko00920	Energy metabolism	Sulfur metabolism	3.4 × 10^−9^	−0.00897
ko05111	Cellular community—prokaryotes	Biofilm formation—Vibrio cholerae	5.2 × 10^−9^	
ko01220	Metabolism	Degradation of aromatic compounds	1.1 × 10^−11^	−0.00851
ko00362	Xenobiotics biodegradation and metabolism	Benzoate degradation	9.2 × 10^−17^	−0.00797
ko02010	Membrane transport	ABC transporters	6.4 × 10^−14^	−0.00791
ko00053	Carbohydrate metabolism	Ascorbate and aldarate metabolism	4.6 × 10^−9^	−0.00783
ko05132	Infectious disease: bacterial	Salmonella infection	1.7 × 10^−13^	−0.00763
ko05133	Infectious disease: bacterial	Pertussis	2.6 × 10^−5^	−0.00762
ko01053	Metabolism of terpenoids and polyketides	Biosynthesis of siderophore group non-ribosomal peptides	9.8 × 10^−13^	−0.00729
ko00040	Carbohydrate metabolism	Pentose and glucuronate interconversions	6.7 × 10^−8^	−0.00728
ko02030	Cell motility	Bacterial chemotaxis	9.0 × 10^−3^	−0.00727
ko00633	Xenobiotics biodegradation and metabolism	Nitrotoluene degradation	4.6 × 10^−10^	−0.00718
ko01120	Metabolism	Microbial metabolism in diverse environments	6.5 × 10^−10^	−0.00713
ko00310	Amino acid metabolism	Lysine degradation	5.8 × 10^−11^	−0.00699

## Supplementary Materials

The following are available online at https://www.mdpi.com/2072-6643/12/5/1345/s1. Table S1. Bacterial KEGG pathway differences between VLBW infants and term infants. Table S2. PCA plots between VLBW infants and term infants by week. Figure S1. Differences in the relative abundances of bacterial class between VLBW infants and term infants. Figure S2. Differences in the relative abundances of bacterial order between VLBW infants and term infants. Figure S3. Differences in the relative abundances of bacterial family between VLBW infants and term infants. Figure S4. Comparison of bacterial KEGG pathways between VLBW infants and term infants.
