# Supplementary material for: Predicted Metabolic Pathway Distributions in Stool Bacteria in Very-Low-Birth-Weight Infants: Potential Relationships with NICU Faltered Growth
Source: Nutrients. 2020 May 8;12(5):1345. doi: 10.3390/nu12051345 (PMC7284701; doi:10.3390/nu12051345)
Supplement: Supplementary file 1 [file nutrients-12-01345-s001.zip › nutrients-769405-supplementary/Supplementary Meterials/S1. Supplementary_data.pdf]

Supplementary data

Metabolic Pathway Distributions in Very Low Birth Weight Infants: Potential Relationships with NICU Faltered Growth

S1. Association of the relative abundance of bacterial class between VLBW infants and term infants. Each page of this file shows a separate bacterial class which was significantly different between the two groups. The box plots indicate the association of the metadata (here, the two groups preterm and term infants) with the relative abundances. The strength of association is indicated by the p-value and an FDR-corrected q-value. The partial residual plot shows the relationship between the groups with abundances when all other variables are included in the model.

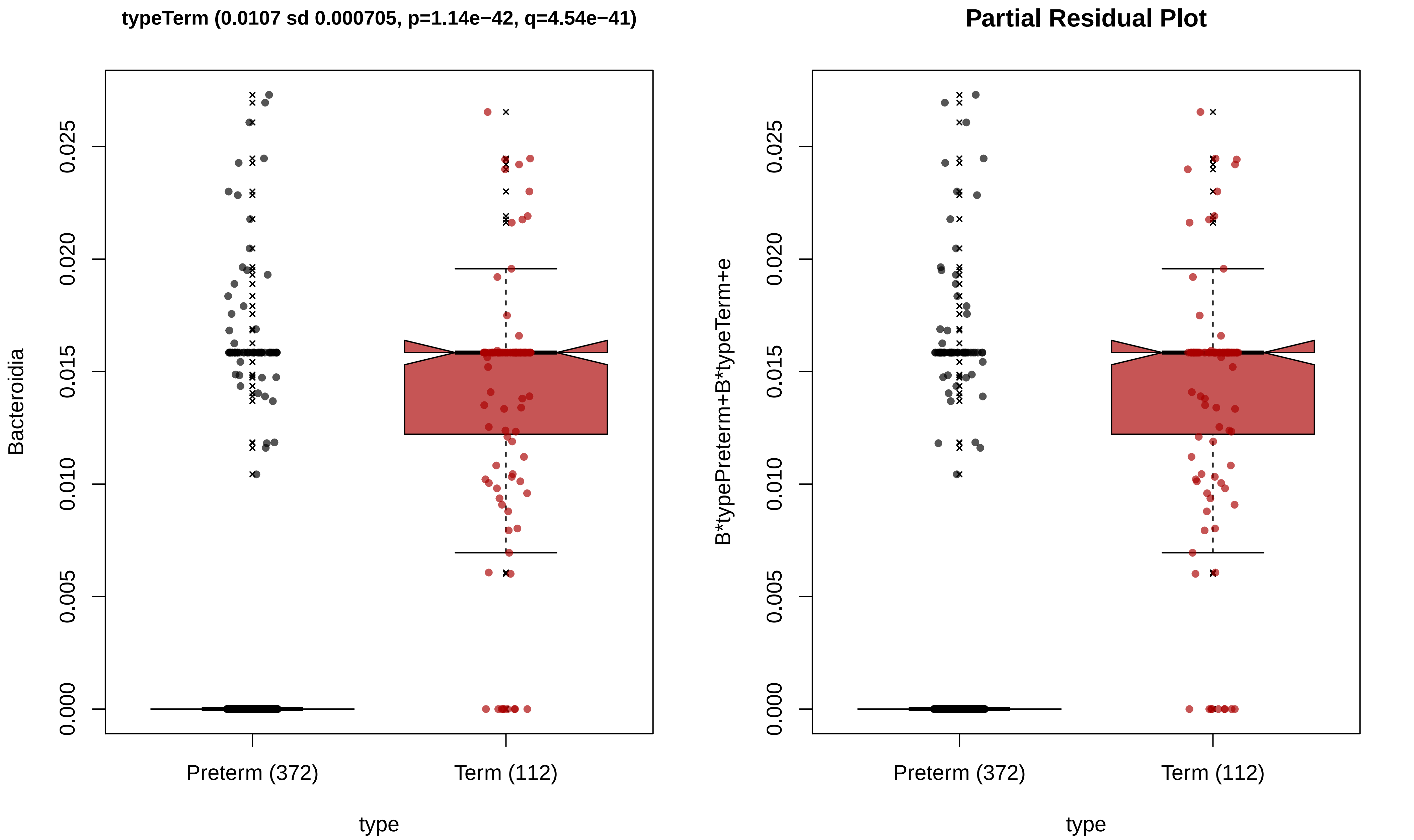

typeTerm (0.0107 sd 0.000705, p=1.14e−42, q=4.54e−41)
Bacteroidia
0.000
0.005
0.010
0.015
0.020
0.025
Preterm (372)
Term (112)
type
Partial Residual Plot
B*typePreterm+B*typeTerm+e
0.000
0.005
0.010
0.015
0.020
0.025
Preterm (372)
Term (112)
type

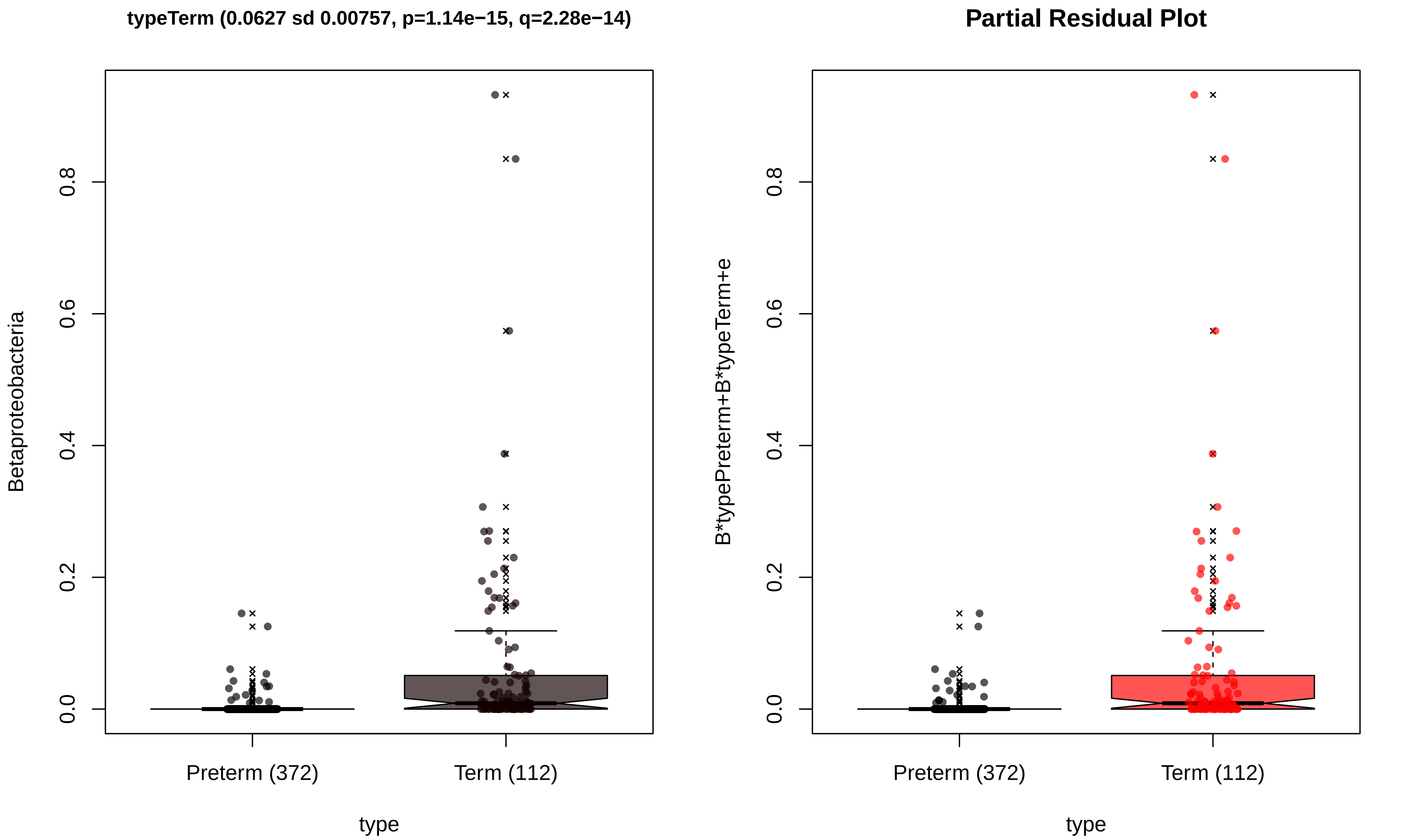
typeTerm (0.0627 sd 0.00757, p=1.14e−15, q=2.28e−14)
Betaproteobacteria
0.0
0.2
0.4
0.6
0.8
Preterm (372)
Term (112)
type
Partial Residual Plot
B*typePreterm+B*typeTerm+e
0.0
0.2
0.4
0.6
0.8
Preterm (372)
Term (112)
type

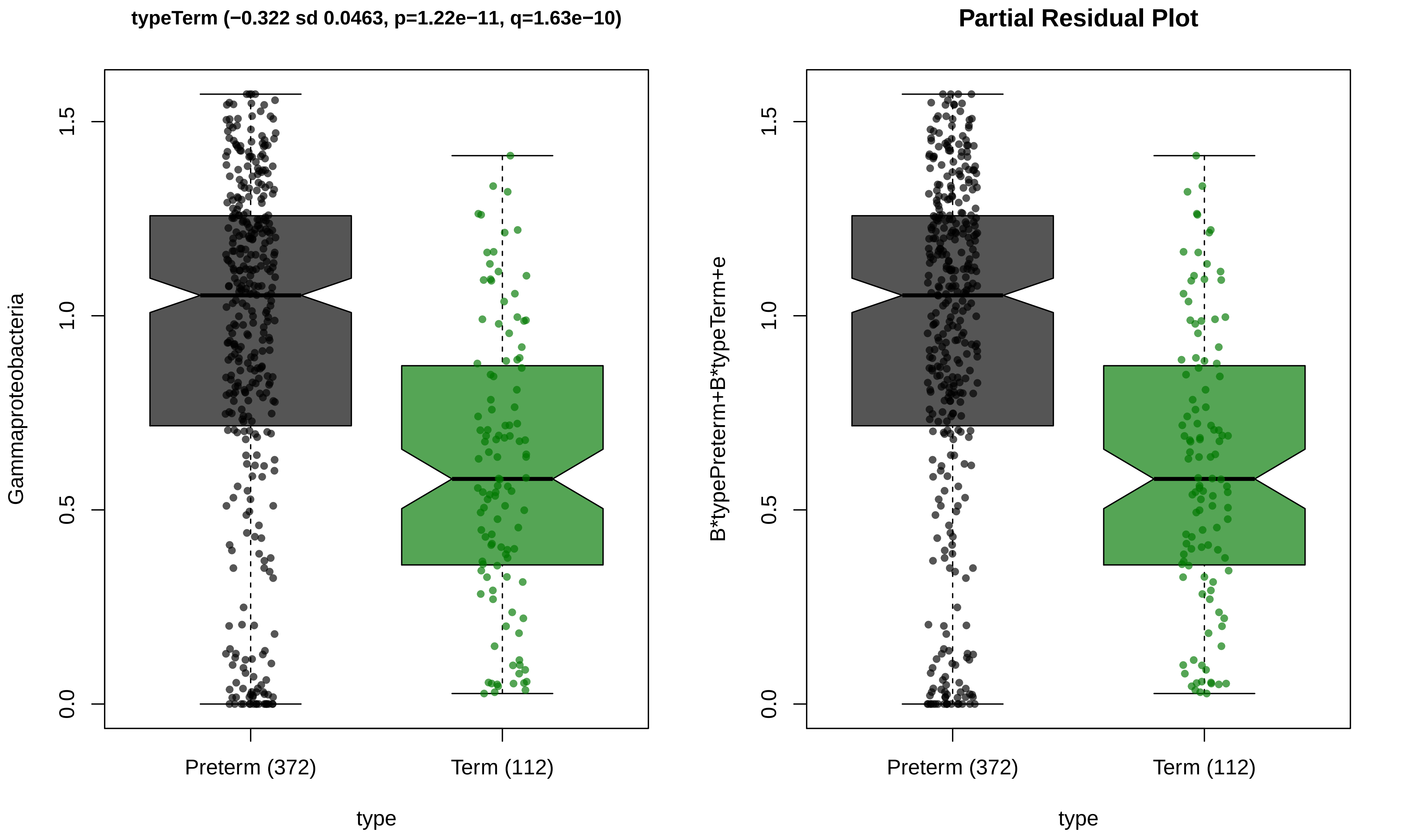
typeTerm (−0.322 sd 0.0463, p=1.22e−11, q=1.63e−10)
Partial Residual Plot
Gammaproteobacteria
B*typePreterm+B*typeTerm+e
0.0
0.5
1.0
1.5
Preterm (372)
Term (112)
type

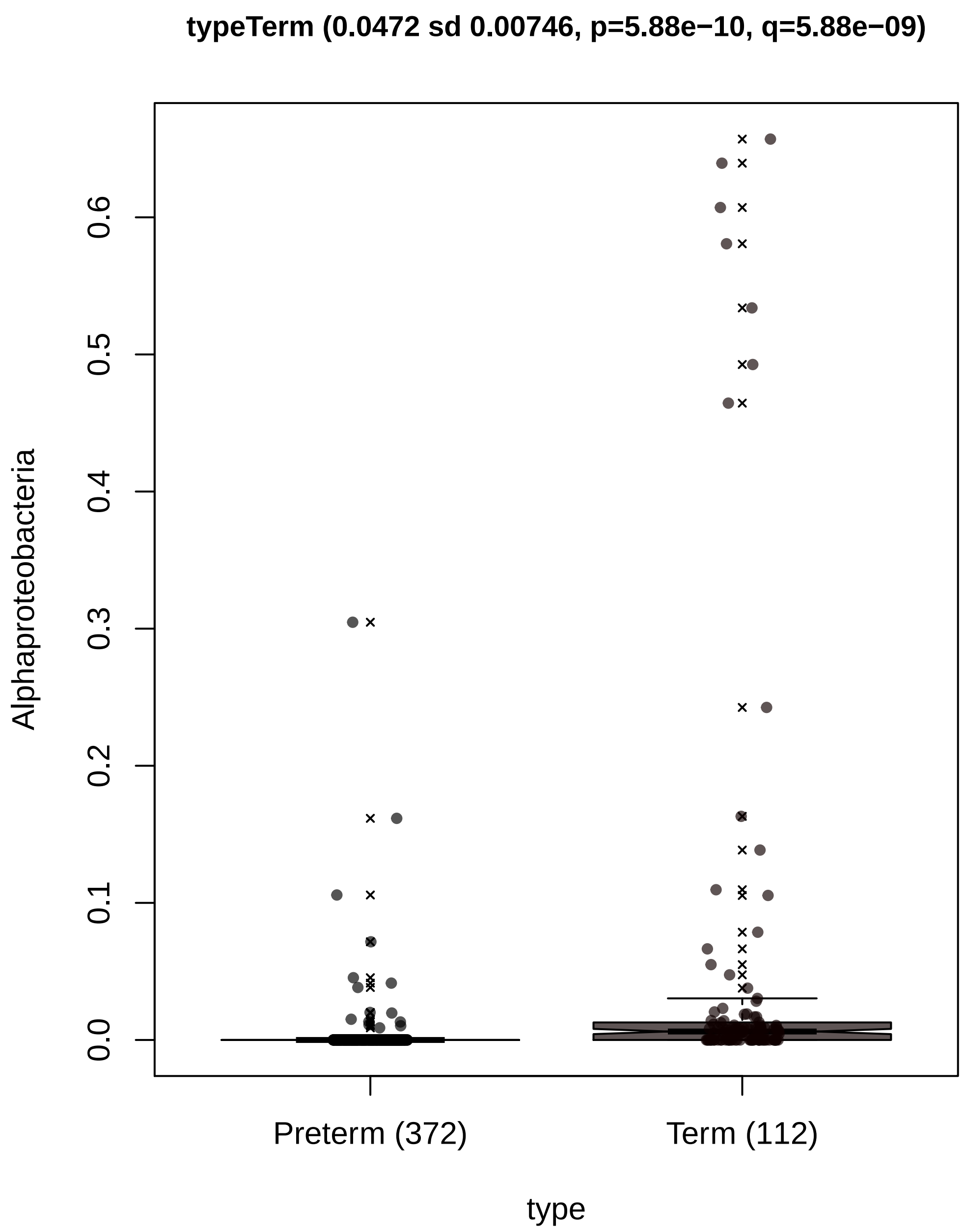
typeTerm (0.0472 sd 0.00746, p=5.88e−10, q=5.88e−09)
Alphaproteobacteria
0.0
0.1
0.2
0.3
0.4
0.5
0.6
Preterm (372)
Term (112)
type

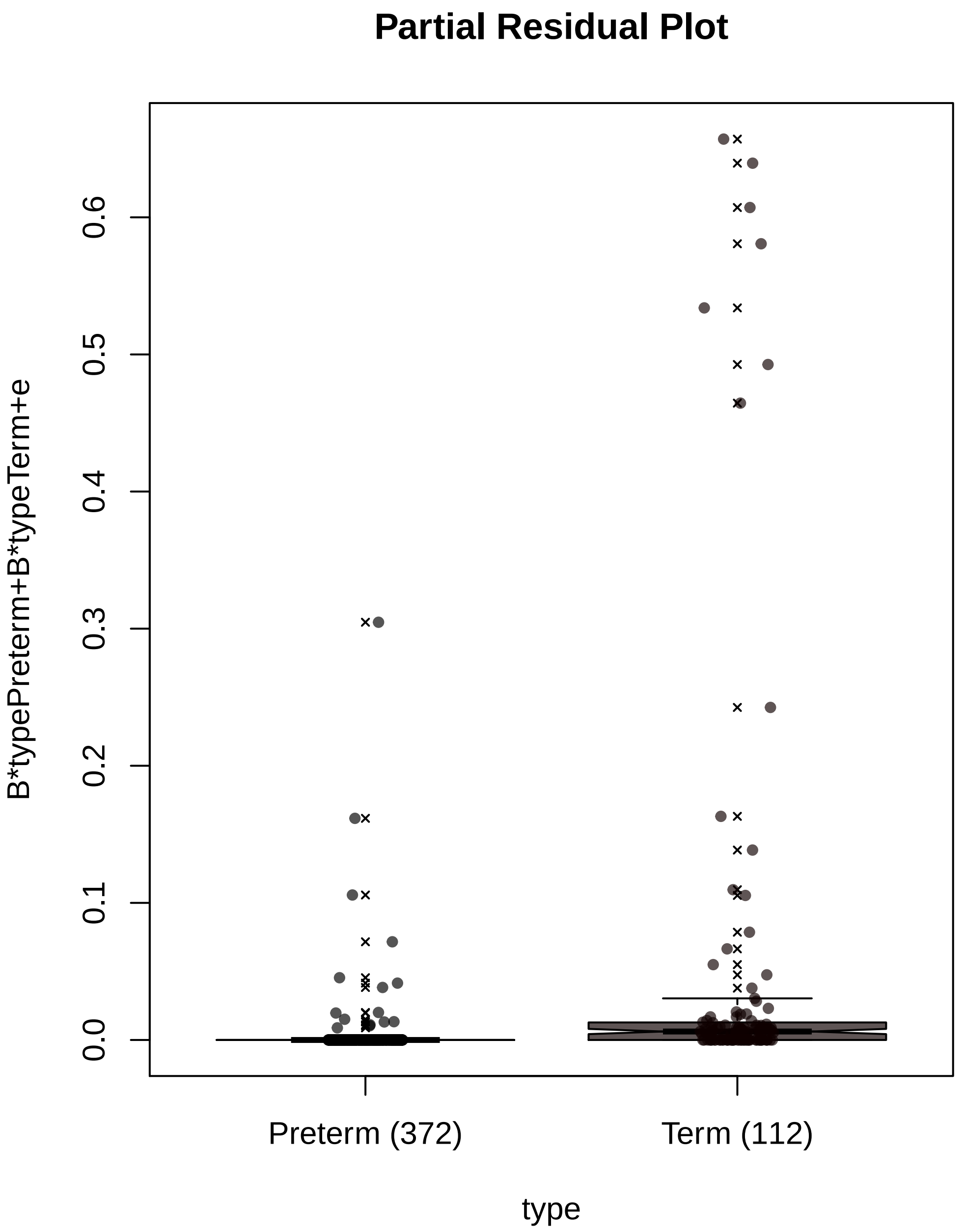
Partial Residual Plot
B*typePreterm+B*typeTerm+e
0.0
0.1
0.2
0.3
0.4
0.5
0.6
Preterm (372)
Term (112)
type

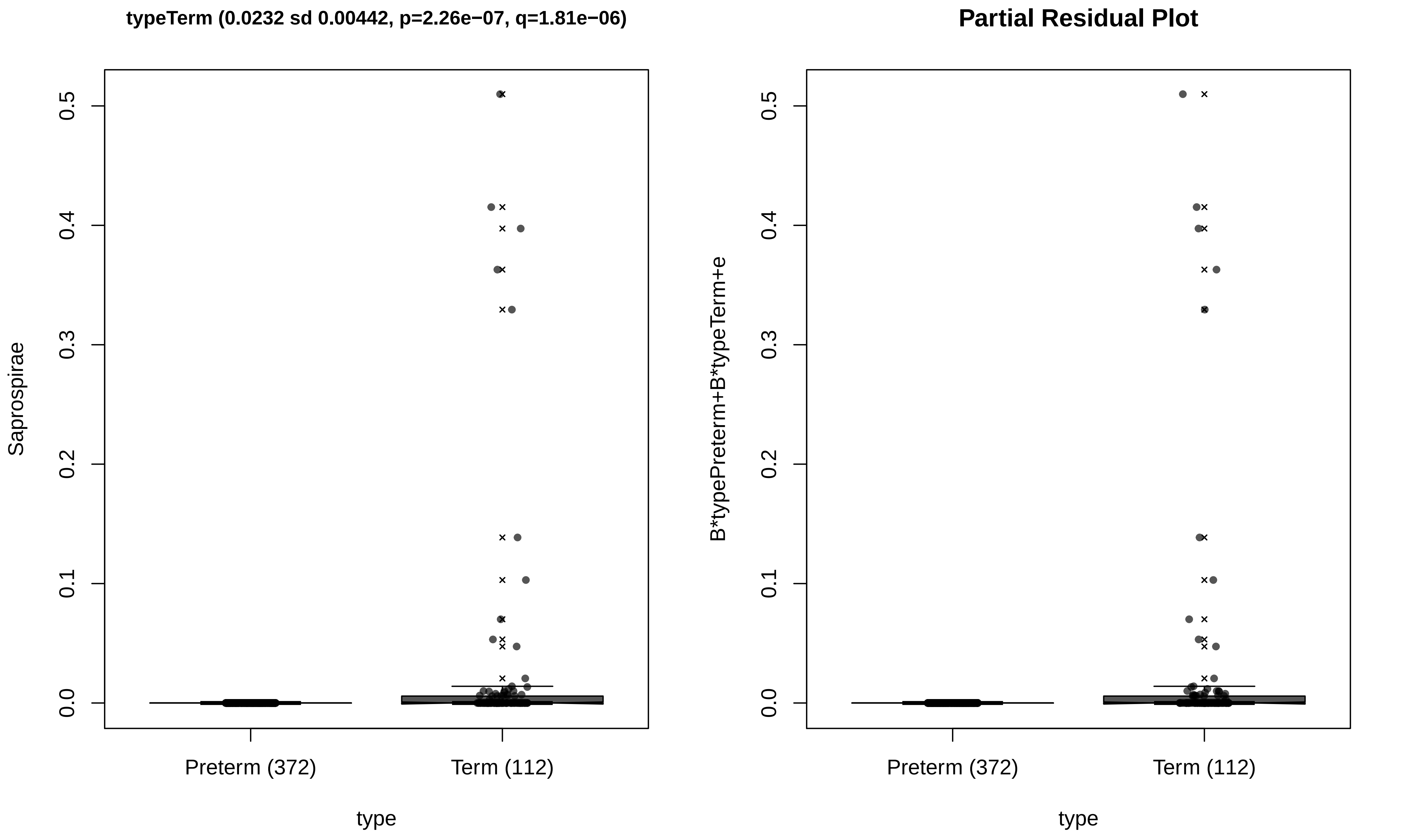

typeTerm (0.0232 sd 0.00442, p=2.26e−07, q=1.81e−06)
Partial Residual Plot
Saprospirae
B*typePreterm+B*typeTerm+e
0.0
0.1
0.2
0.3
0.4
0.5
Preterm (372)
Term (112)
type

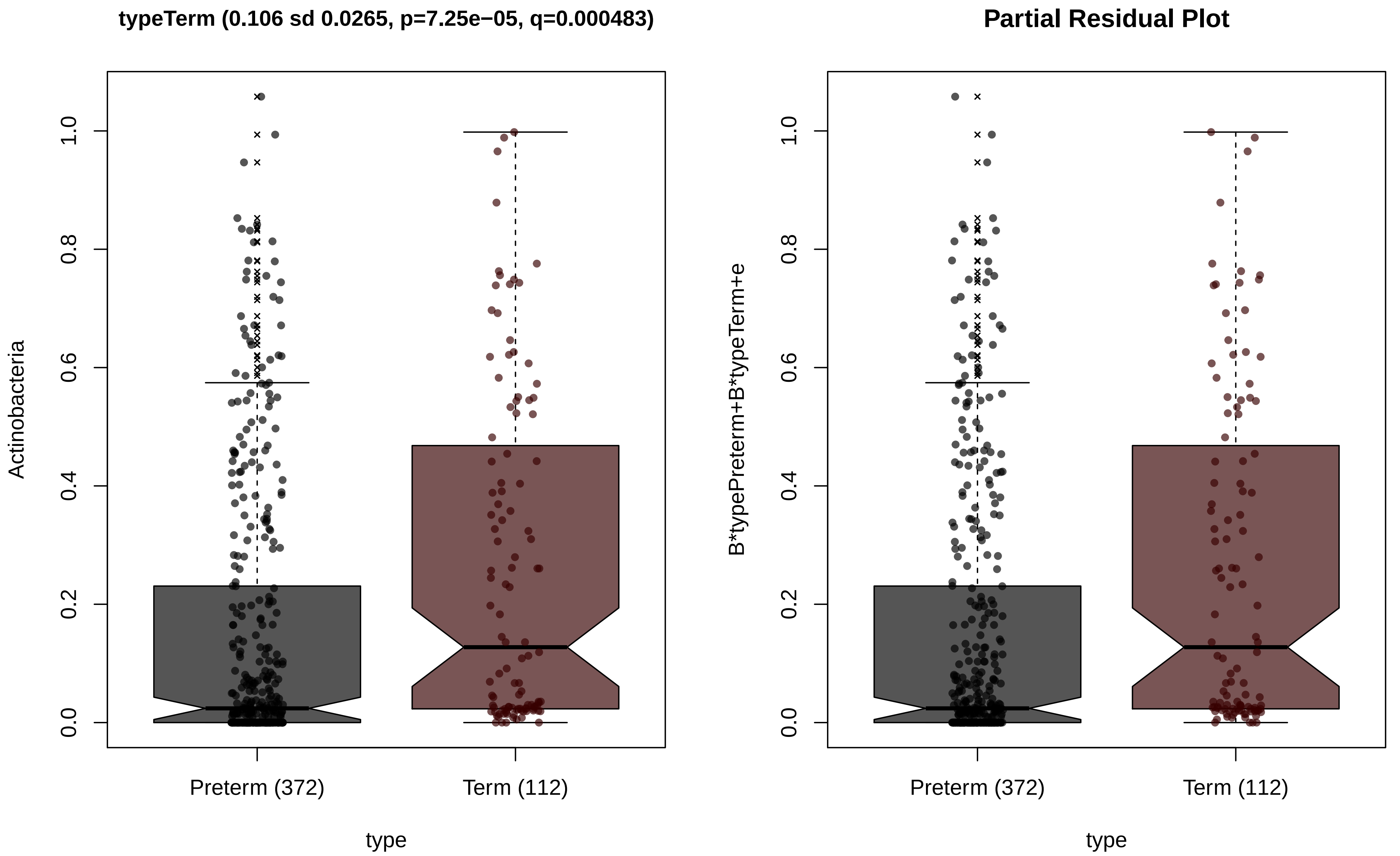
typeTerm (0.106 sd 0.0265, p=7.25e−05, q=0.000483)
Partial Residual Plot
Actinobacteria
B*typePreterm+B*typeTerm+e
0.0
0.2
0.4
0.6
0.8
1.0
Preterm (372)
Term (112)
type

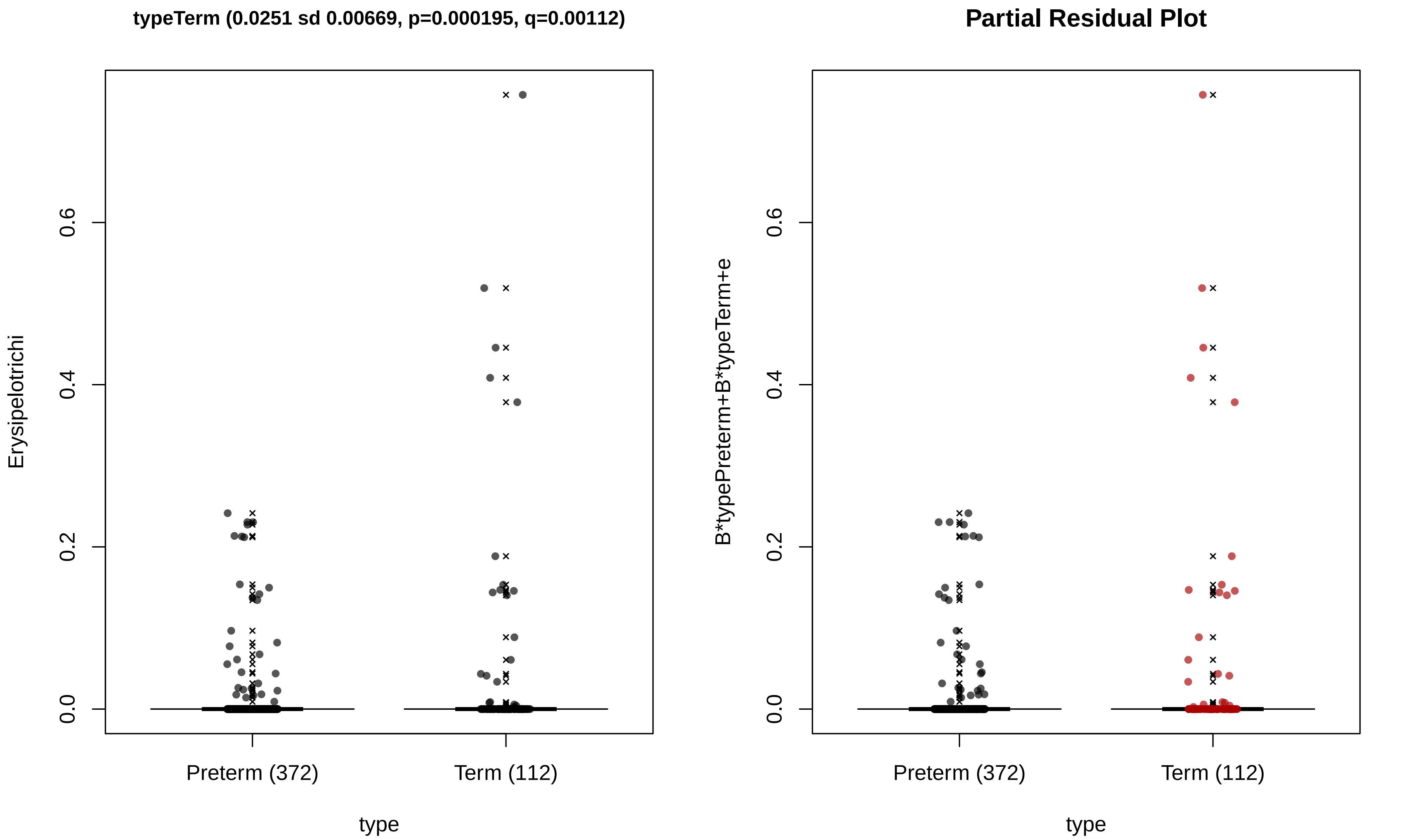

typeTerm (0.0251 sd 0.00669, p=0.000195, q=0.00112)
Partial Residual Plot
Erysipelotrichi
B*typePreterm+B*typeTerm+e
0.0
0.2
0.4
0.6
Preterm (372)
Term (112)
type

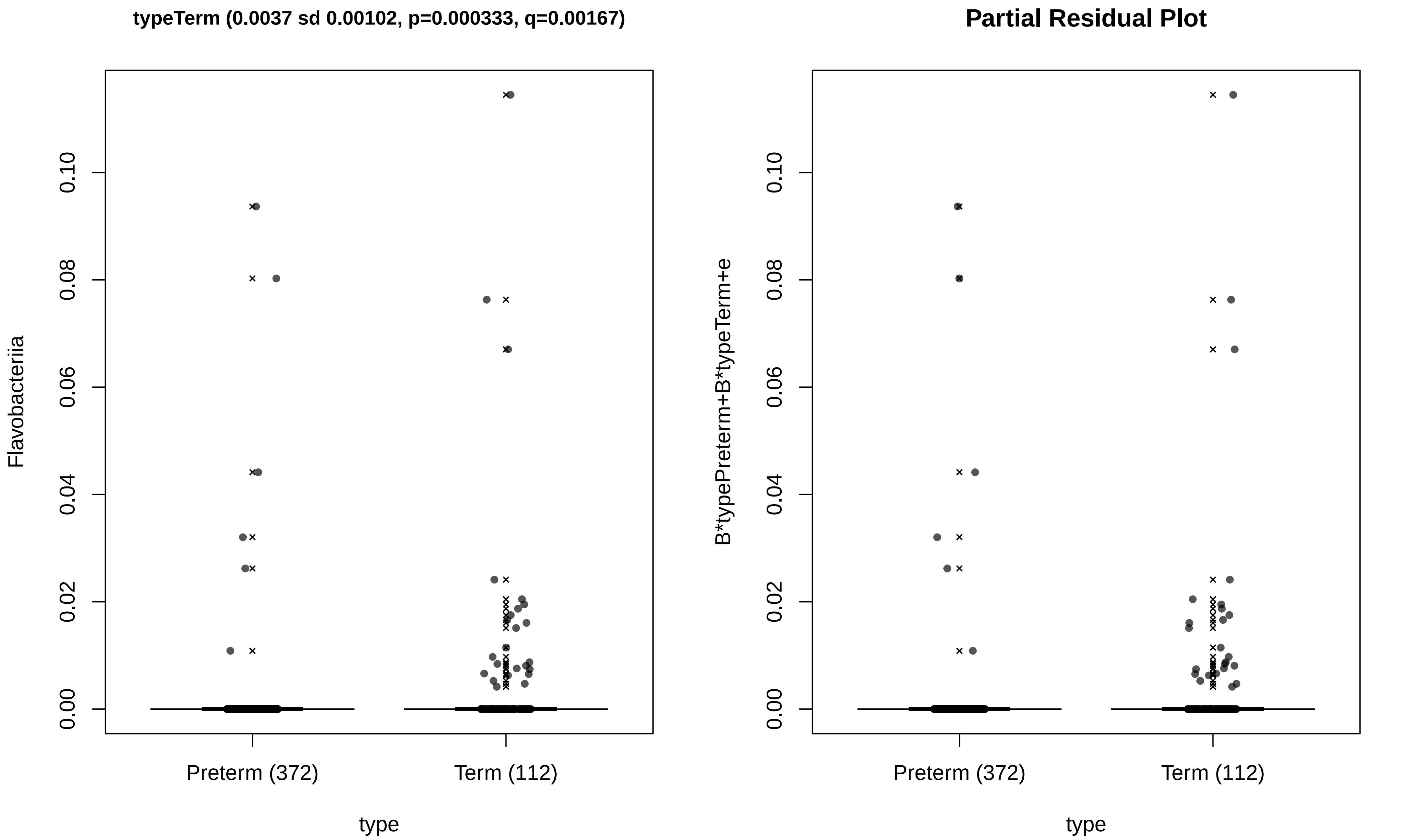
typeTerm (0.0037 sd 0.00102, p=0.000333, q=0.00167)
Flavobacteriia
0.00
0.02
0.04
0.06
0.08
0.10
Preterm (372)
Term (112)
type
Partial Residual Plot
B*typePreterm+B*typeTerm+e
0.00
0.02
0.04
0.06
0.08
0.10
Preterm (372)
Term (112)
type

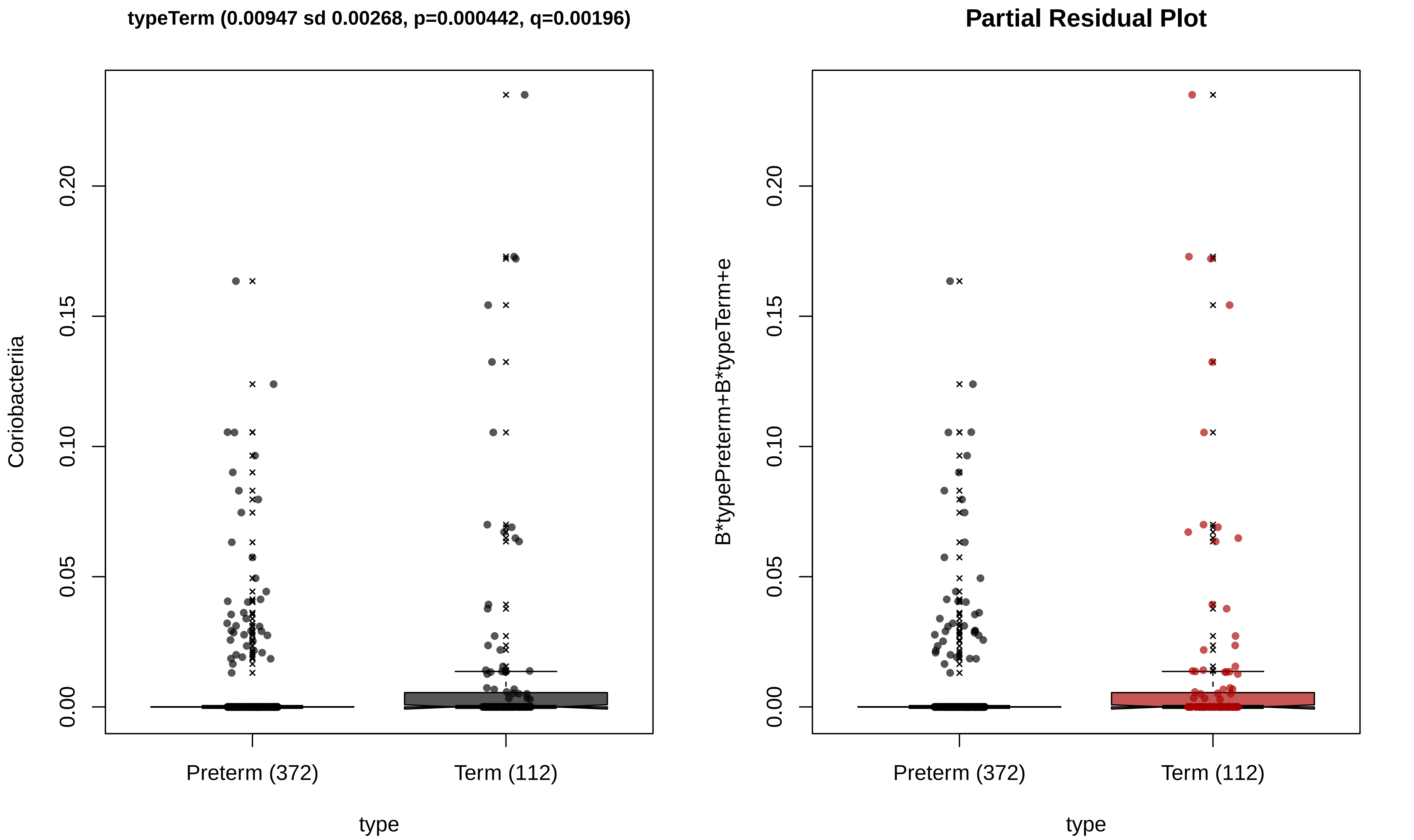

typeTerm (0.00947 sd 0.00268, p=0.000442, q=0.00196)
Coriobacteriia
0.00
0.05
0.10
0.15
0.20
Preterm (372)
Term (112)
type
Partial Residual Plot
B*typePreterm+B*typeTerm+e
0.00
0.05
0.10
0.15
0.20
Preterm (372)
Term (112)
type

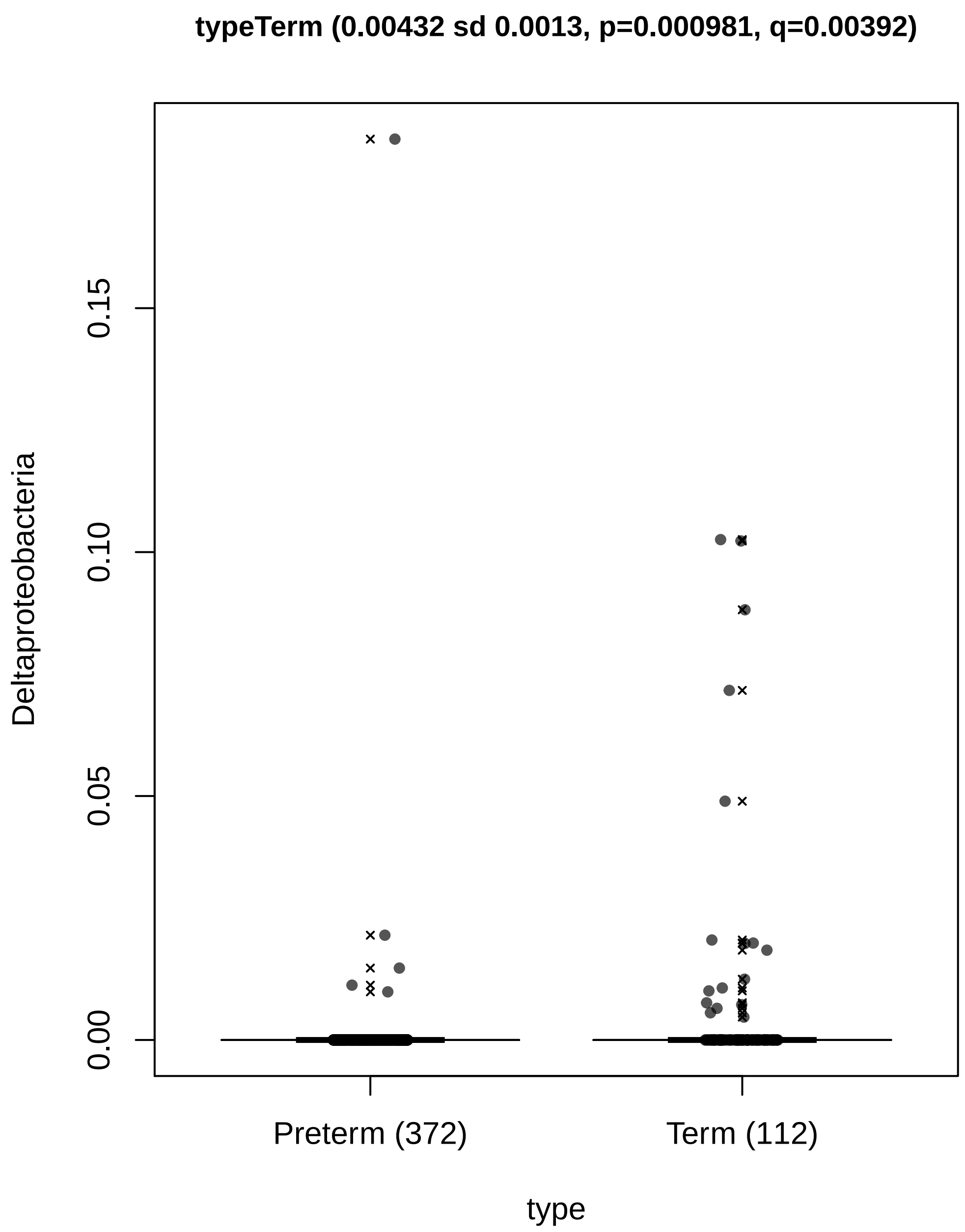
typeTerm (0.00432 sd 0.0013, p=0.000981, q=0.00392)
Deltaproteobacteria
0.00
0.05
0.10
0.15
Preterm (372)
Term (112)
type

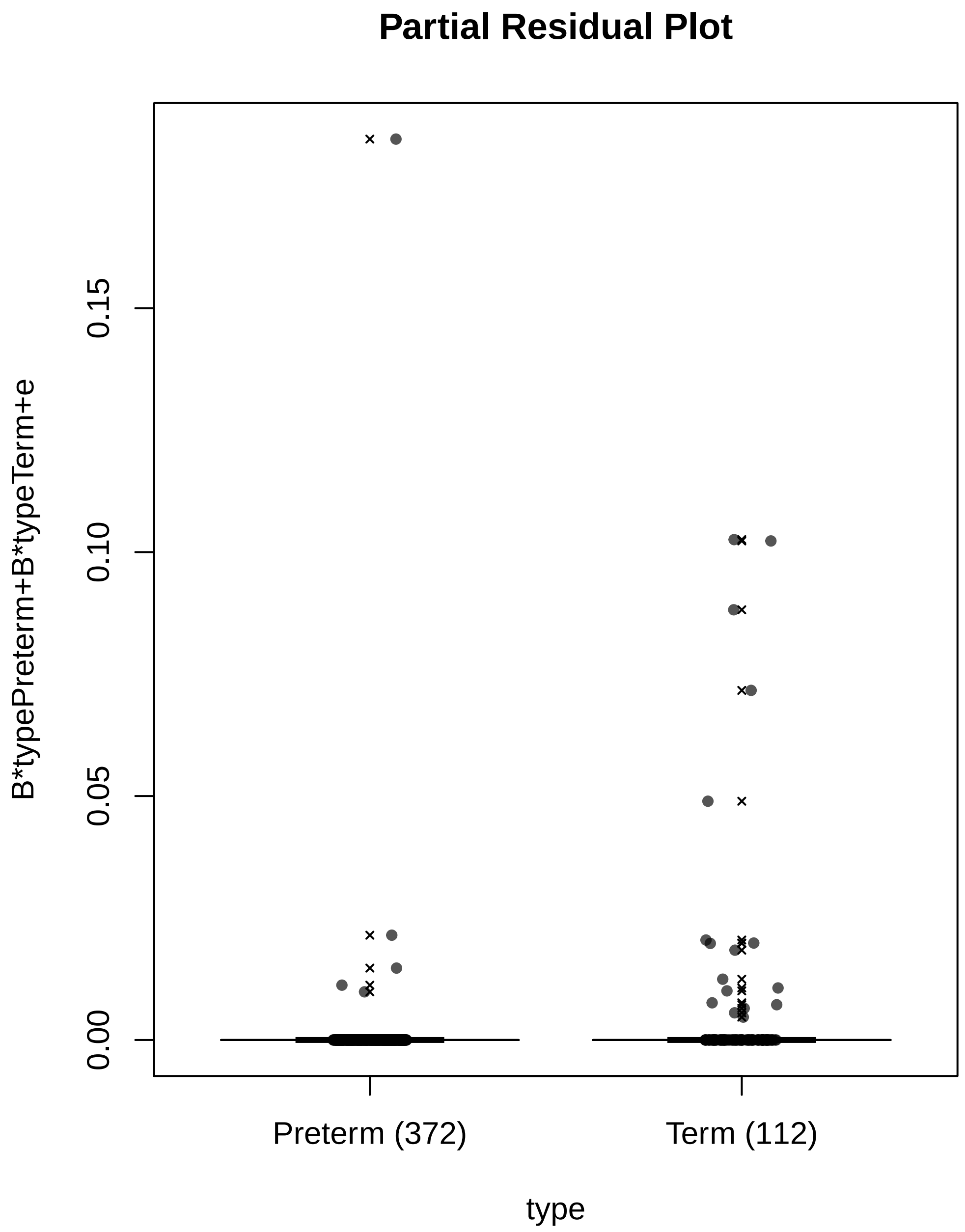
Partial Residual Plot
B*typePreterm+B*typeTerm+e
0.00
0.05
0.10
0.15
Preterm (372)
Term (112)
type

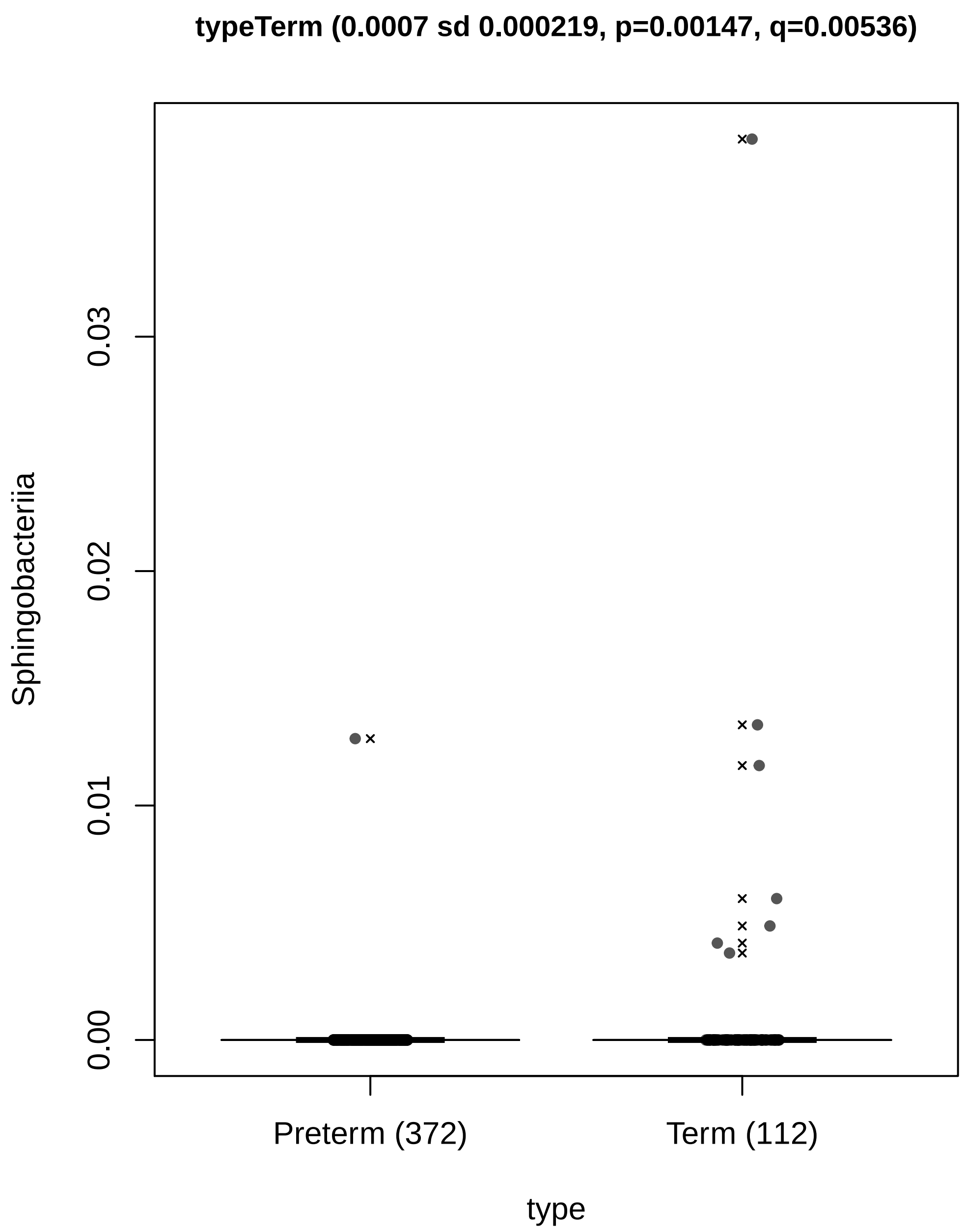

typeTerm (0.0007 sd 0.000219, p=0.00147, q=0.00536)
Sphingobacteriia
0.00
0.01
0.02
0.03
Preterm (372)
Term (112)
type

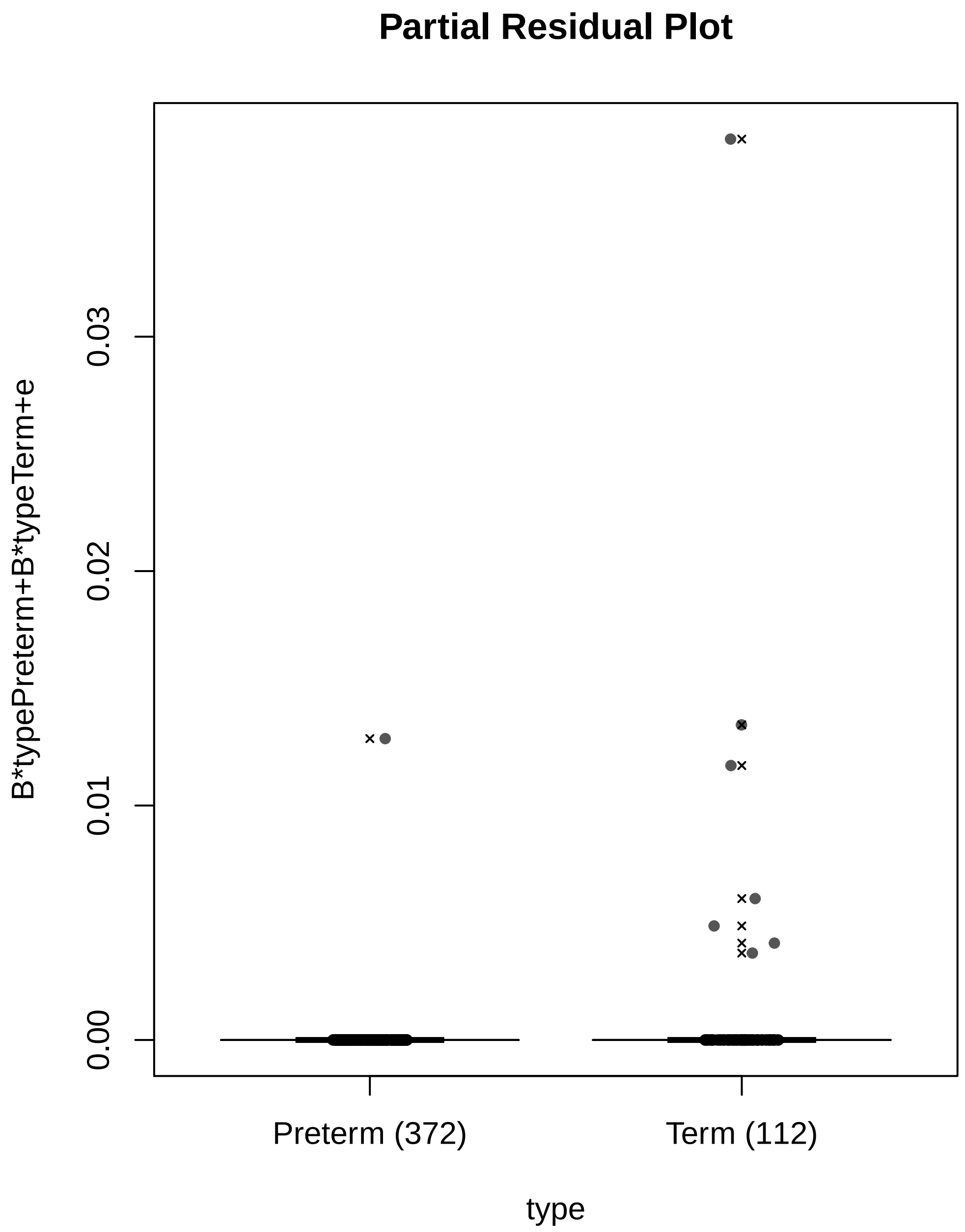

Partial Residual Plot
B*typePreterm+B*typeTerm+e
0.00
0.01
0.02
0.03
Preterm (372)
Term (112)
type

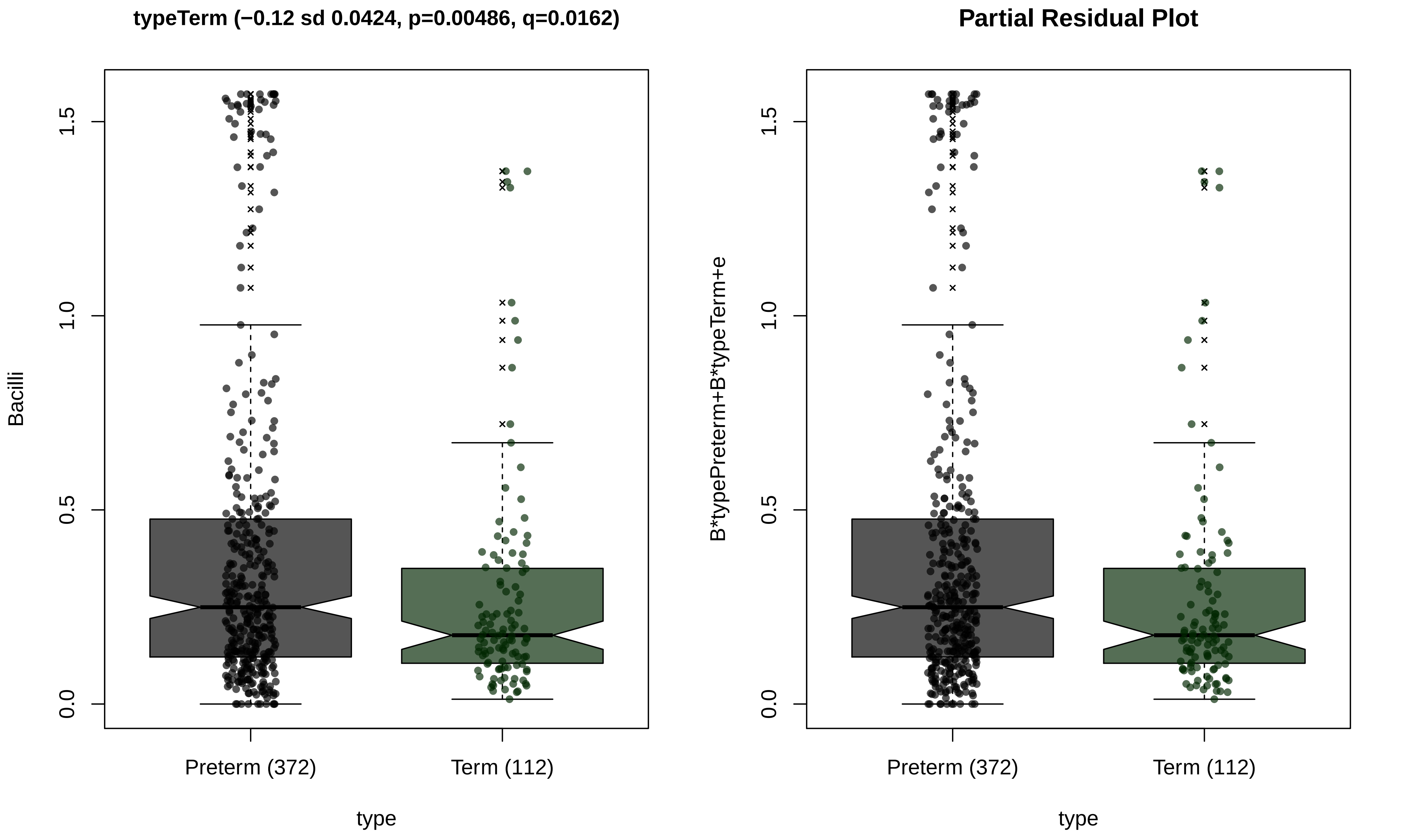

typeTerm (−0.12 sd 0.0424, p=0.00486, q=0.0162)
Bacilli
0.0
0.5
1.0
1.5
Preterm (372)
Term (112)
type
Partial Residual Plot
B*typePreterm+B*typeTerm+e
0.0
0.5
1.0
1.5
Preterm (372)
Term (112)
type

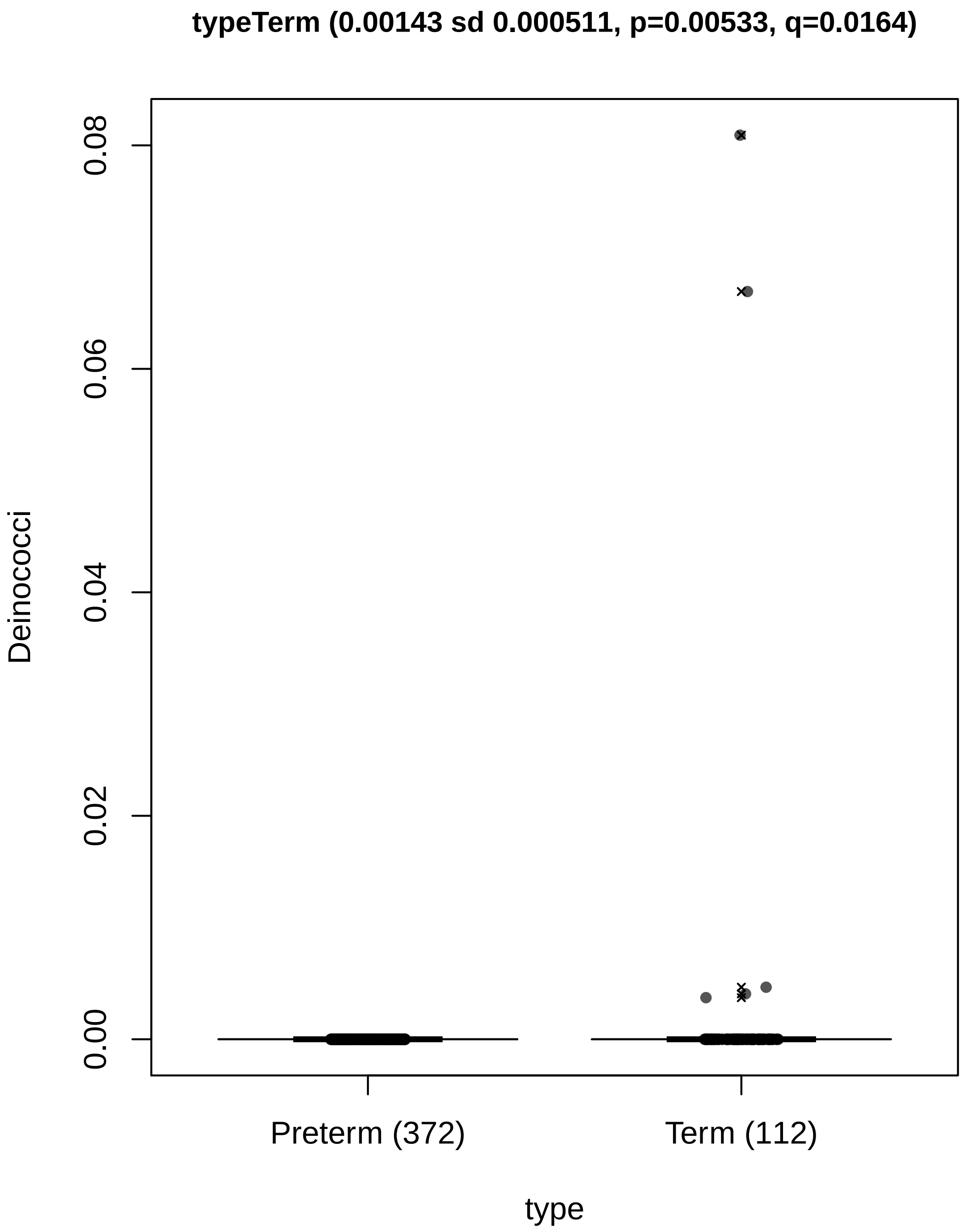
typeTerm (0.00143 sd 0.000511, p=0.00533, q=0.0164)
Deinococci
0.08
0.06
0.04
0.02
0.00
Preterm (372)
Term (112)
type

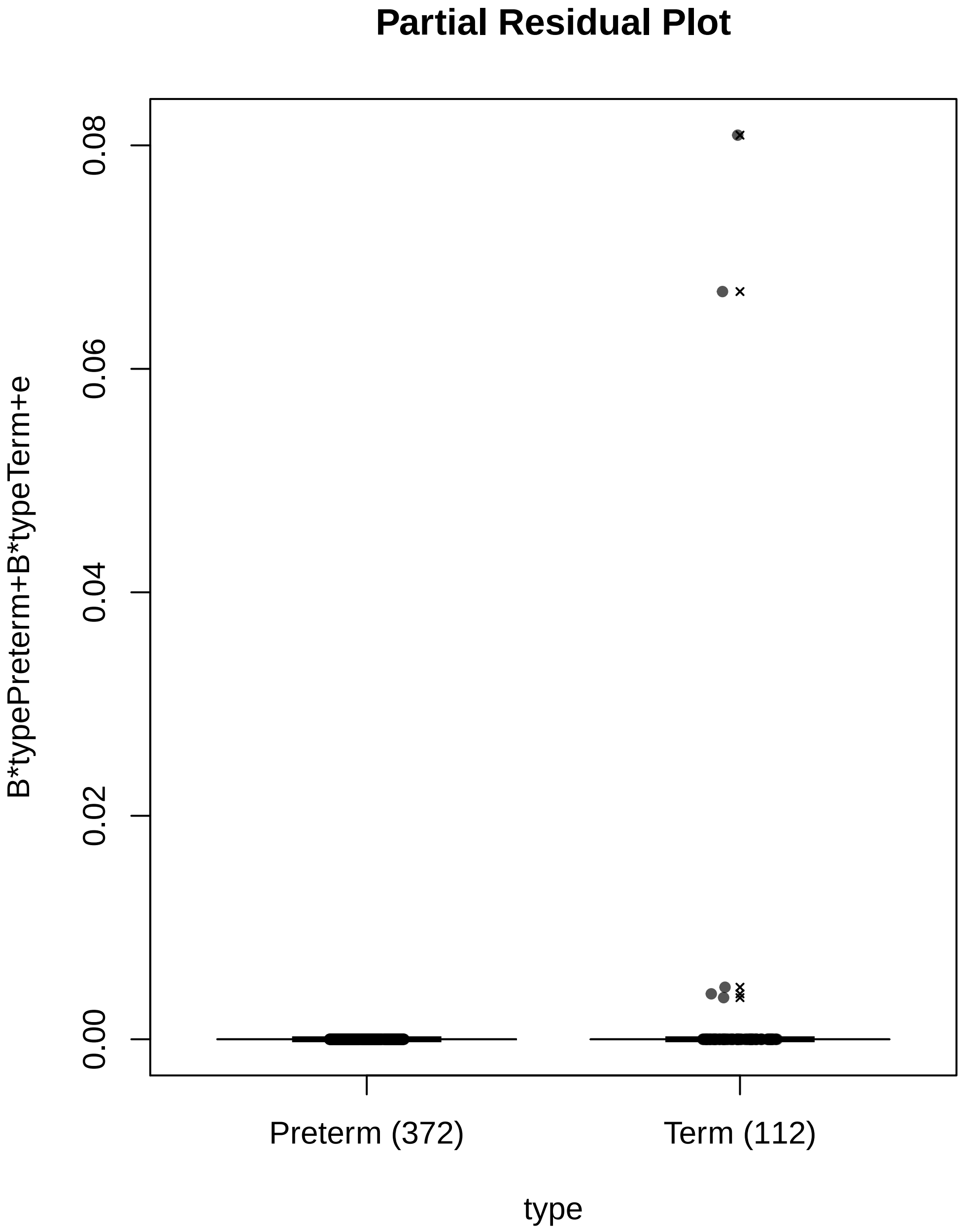
Partial Residual Plot
B*typePreterm+B*typeTerm+e
0.08
0.06
0.04
0.02
0.00
Preterm (372)
Term (112)
type

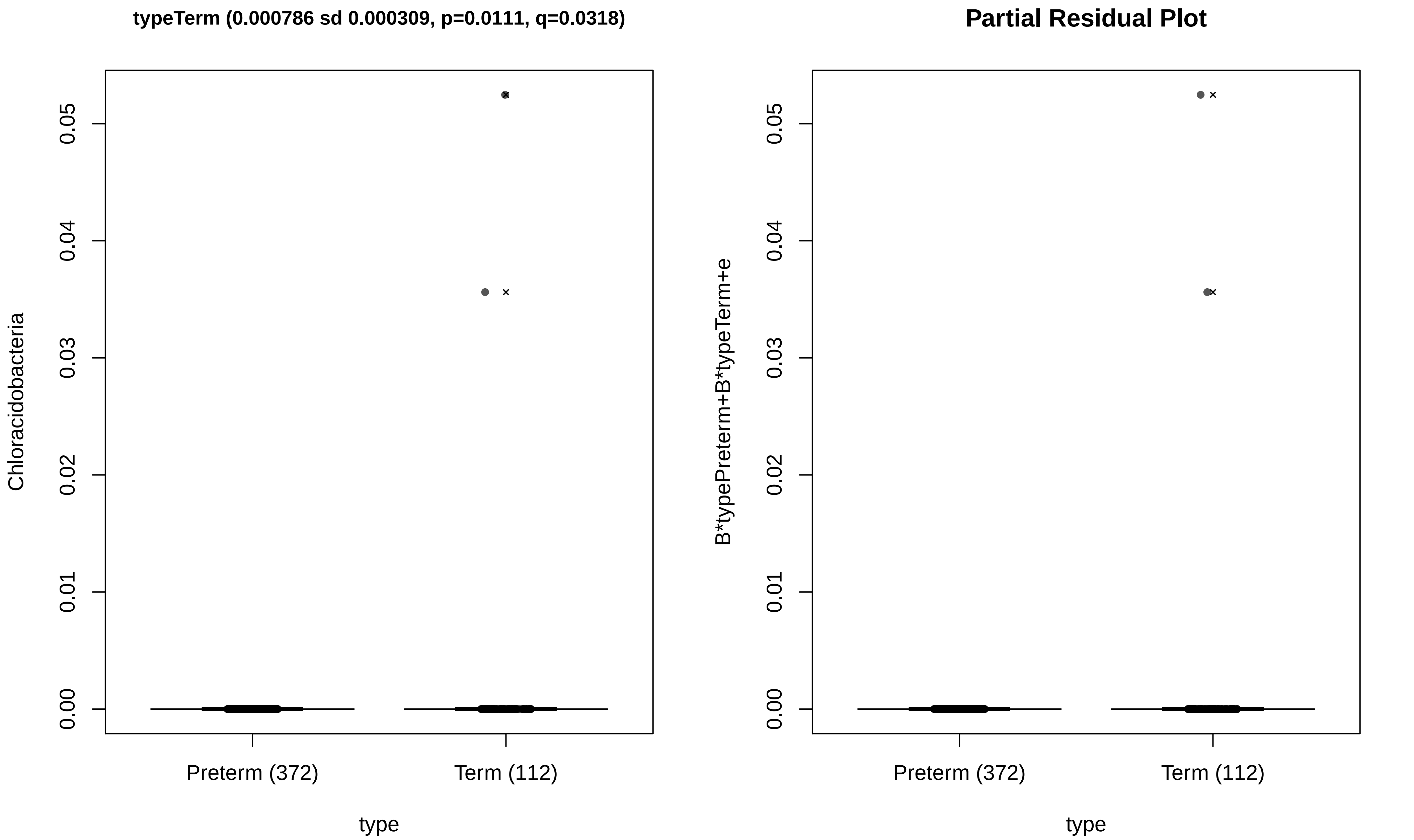

typeTerm (0.000786 sd 0.000309, p=0.0111, q=0.0318)
Chloracidobacteria
0.00
0.01
0.02
0.03
0.04
0.05
Preterm (372)
Term (112)
type
Partial Residual Plot
B*typePreterm+B*typeTerm+e
0.00
0.01
0.02
0.03
0.04
0.05
Preterm (372)
Term (112)
type
