# Supplementary material for: Predicted Metabolic Pathway Distributions in Stool Bacteria in Very-Low-Birth-Weight Infants: Potential Relationships with NICU Faltered Growth
Source: Nutrients. 2020 May 8;12(5):1345. doi: 10.3390/nu12051345 (PMC7284701; doi:10.3390/nu12051345)
Supplement: Supplementary file 1 [file nutrients-12-01345-s001.zip › nutrients-769405-supplementary/Supplementary Meterials/S2_Supplementary_data.pdf]

Supplementary data

Metabolic Pathway Distributions in Very Low Birth Weight Infants: Potential Relationships with NICU Faltered Growth

S2. **Association of the relative abundance of bacterial Order between VLBW infants and term infants**. Each page of this file shows a separate bacterial order which was significantly different between the two groups. The box plots indicate the association of the metadata (here, the two groups preterm and term infants) with the relative abundances. The strength of association is indicated by the p-value and an FDR-corrected q-value. The partial residual plot shows the relationship between the groups with abundances when all other variables are included in the model.

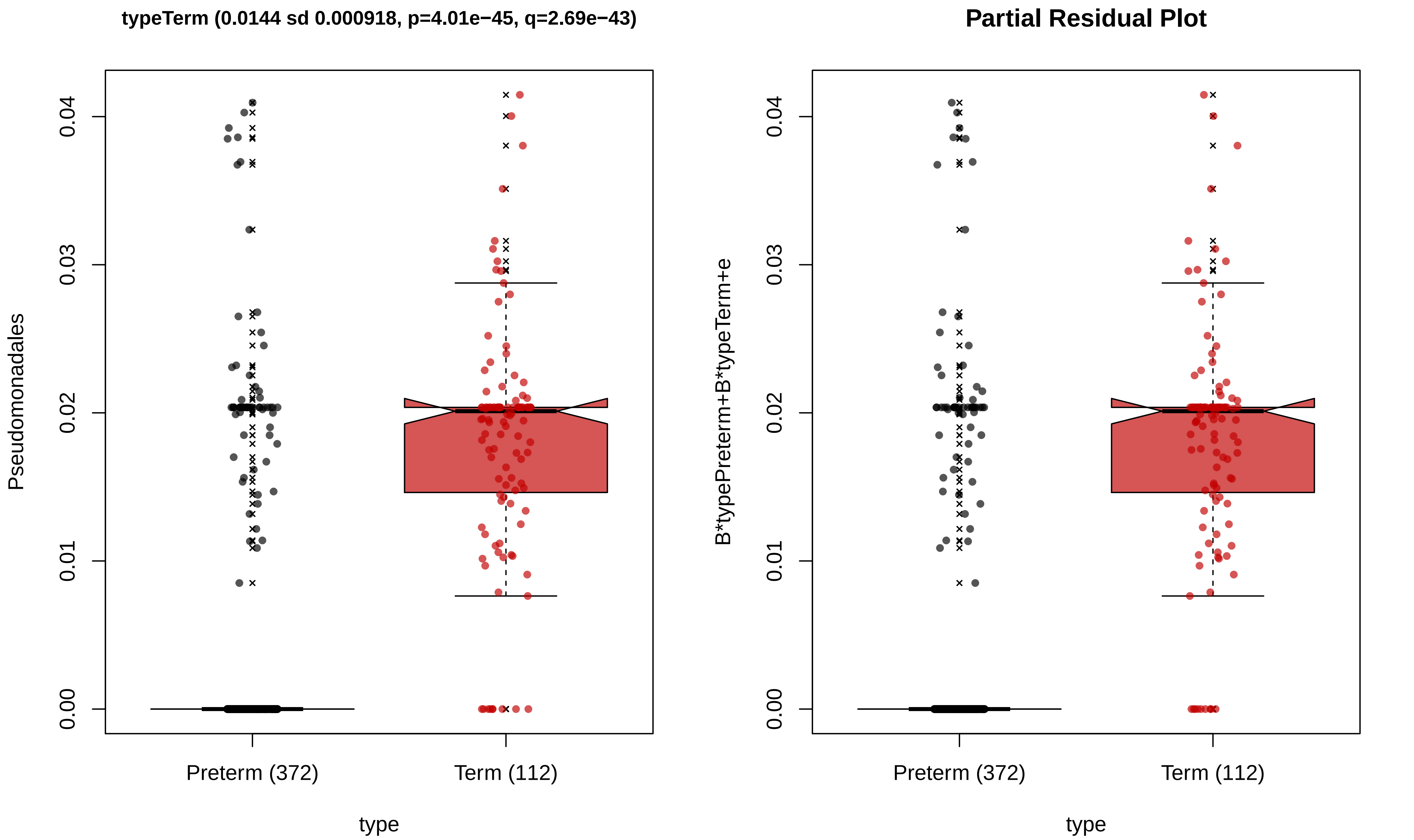
typeTerm (0.0144 sd 0.000918, p=4.01e−45, q=2.69e−43)
Partial Residual Plot
Pseudomonadales
B*typePreterm+B*typeTerm+e
0.00
0.01
0.02
0.03
0.04
Preterm (372)
Term (112)
type

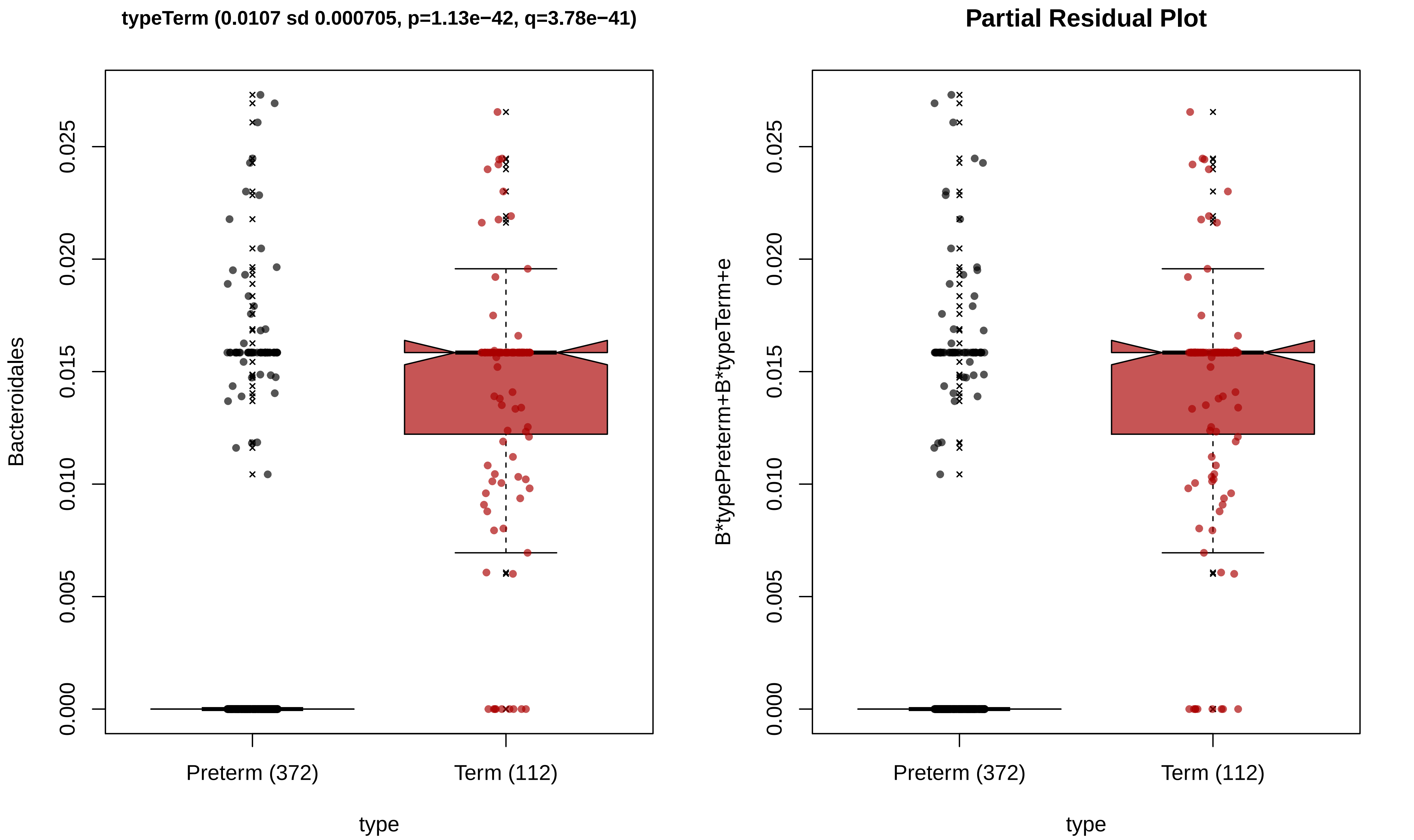
typeTerm (0.0107 sd 0.000705, p=1.13e−42, q=3.78e−41)
Partial Residual Plot
Bacteroidales
B*typePreterm+B*typeTerm+e
0.000
0.005
0.010
0.015
0.020
0.025
Preterm (372)
Term (112)
type

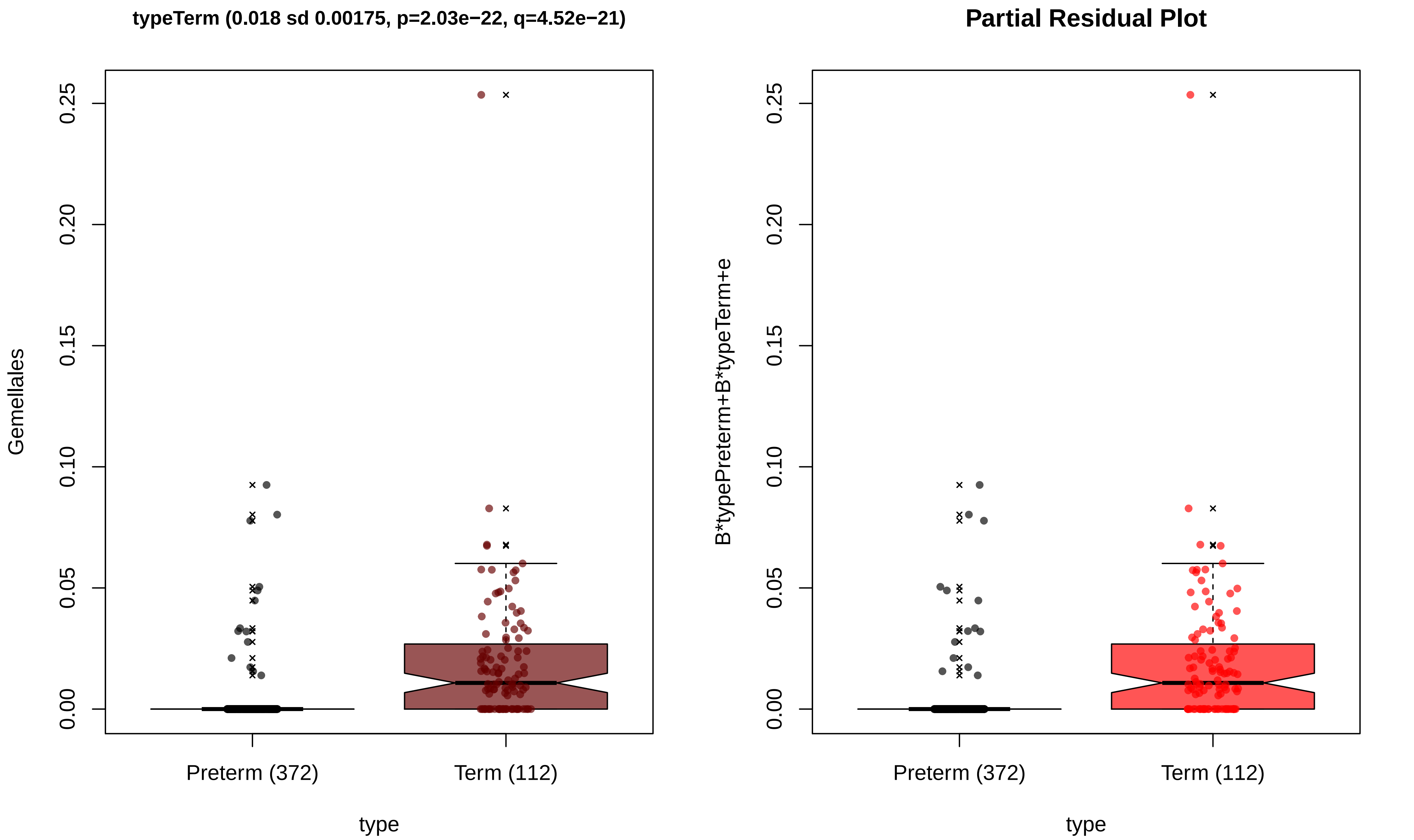
typeTerm (0.018 sd 0.00175, p=2.03e−22, q=4.52e−21)
Gemellales
0.00
0.05
0.10
0.15
0.20
0.25
Preterm (372)
Term (112)
type
Partial Residual Plot
B*typePreterm+B*typeTerm+e
0.00
0.05
0.10
0.15
0.20
0.25
Preterm (372)
Term (112)
type

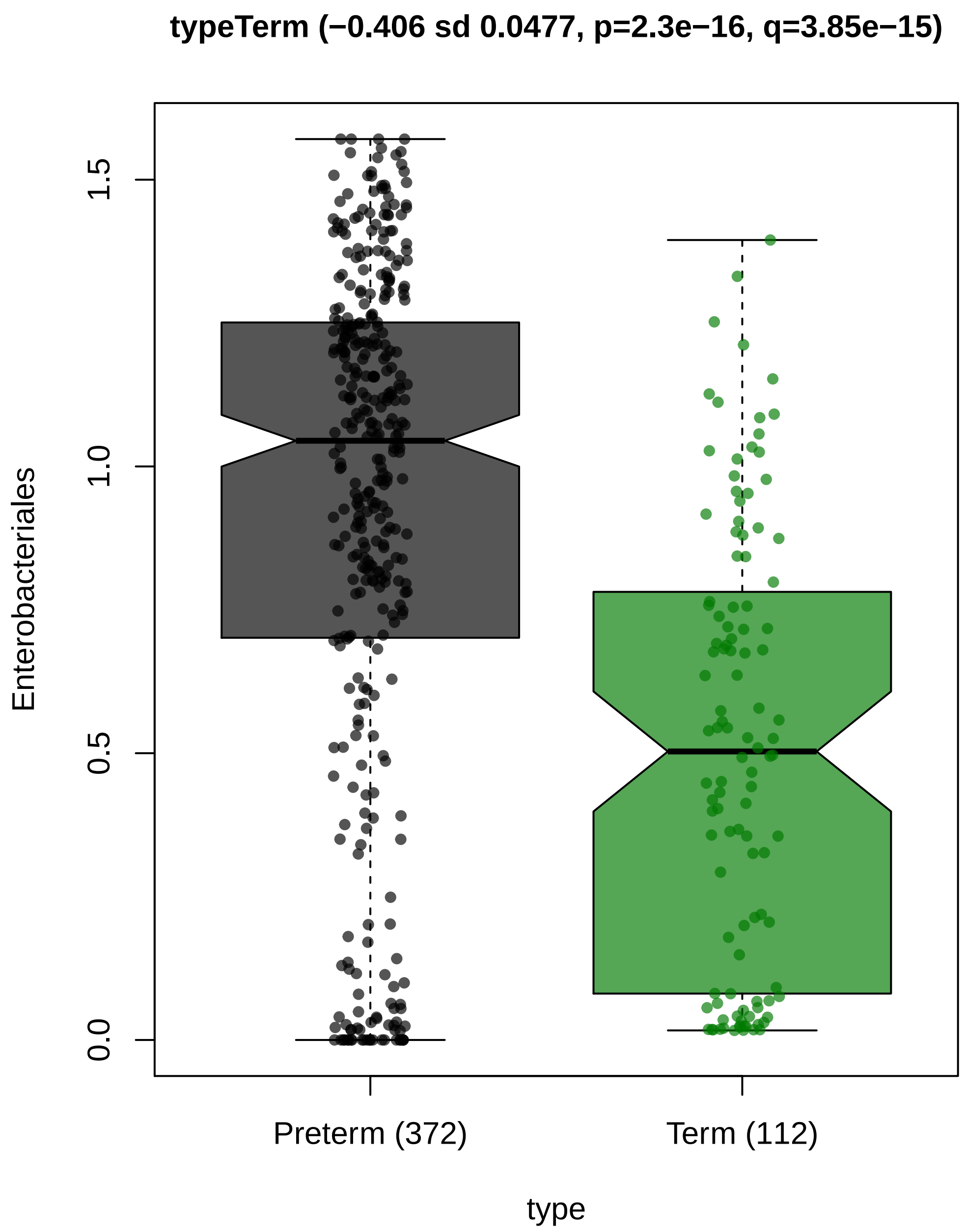
typeTerm (−0.406 sd 0.0477, p=2.3e−16, q=3.85e−15)
Enterobacteriales
0.0
0.5
1.0
1.5
Preterm (372)
Term (112)
type

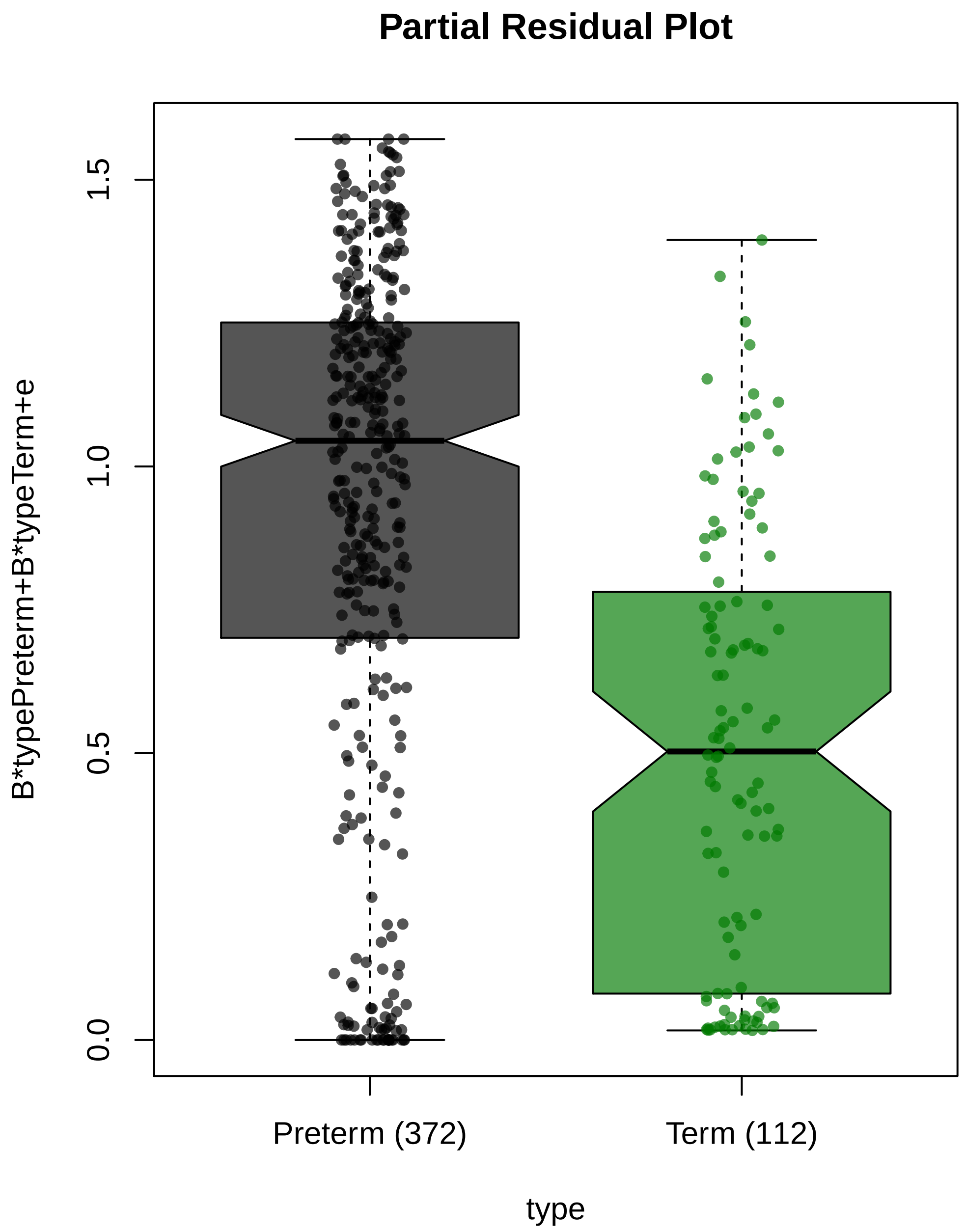
Partial Residual Plot
B*typePreterm+B*typeTerm+e
0.0
0.5
1.0
1.5
Preterm (372)
Term (112)
type

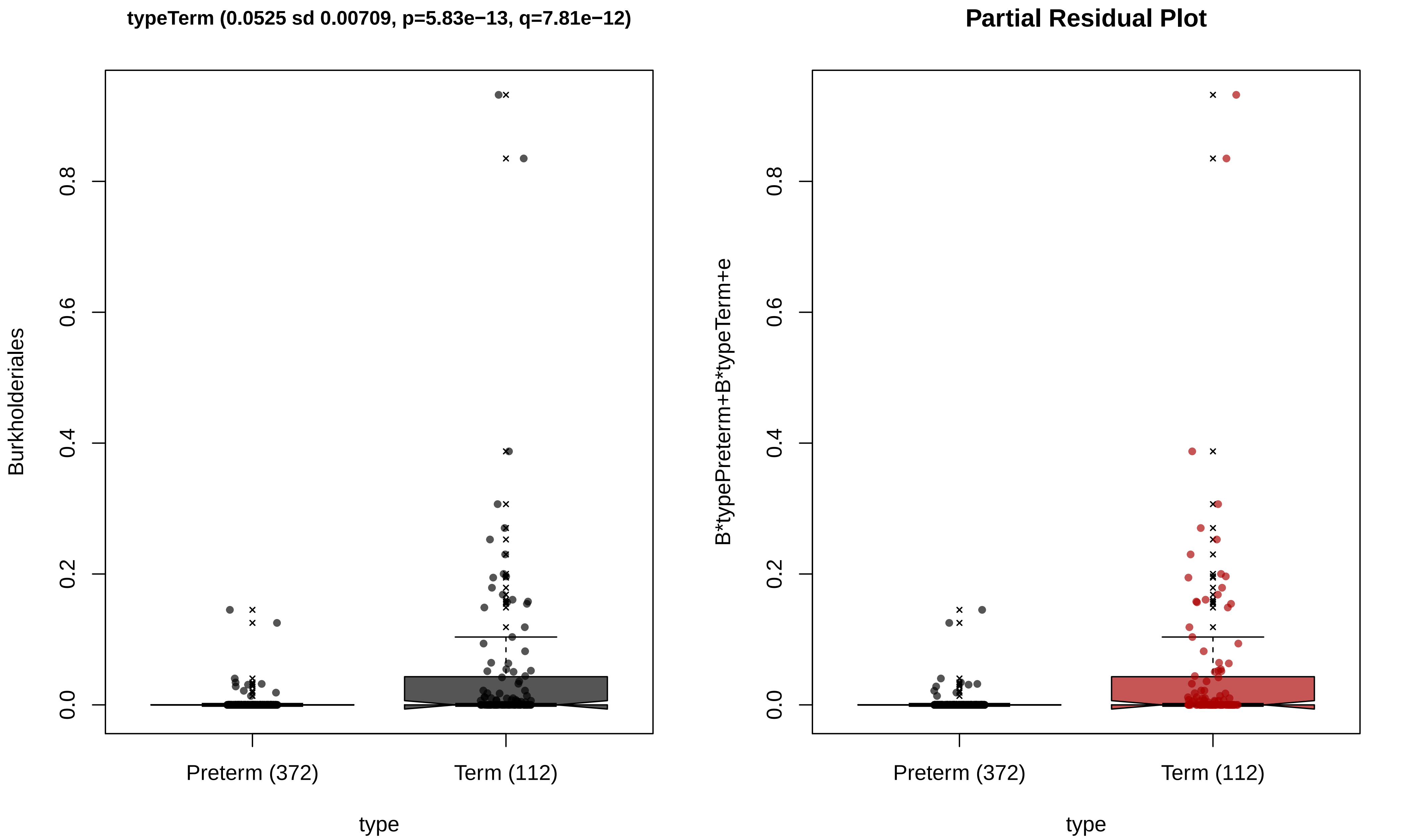
typeTerm (0.0525 sd 0.00709, p=5.83e−13, q=7.81e−12)
Burkholderiales
0.0
0.2
0.4
0.6
0.8
Preterm (372)
Term (112)
type
Partial Residual Plot
B*typePreterm+B*typeTerm+e
0.0
0.2
0.4
0.6
0.8
Preterm (372)
Term (112)
type

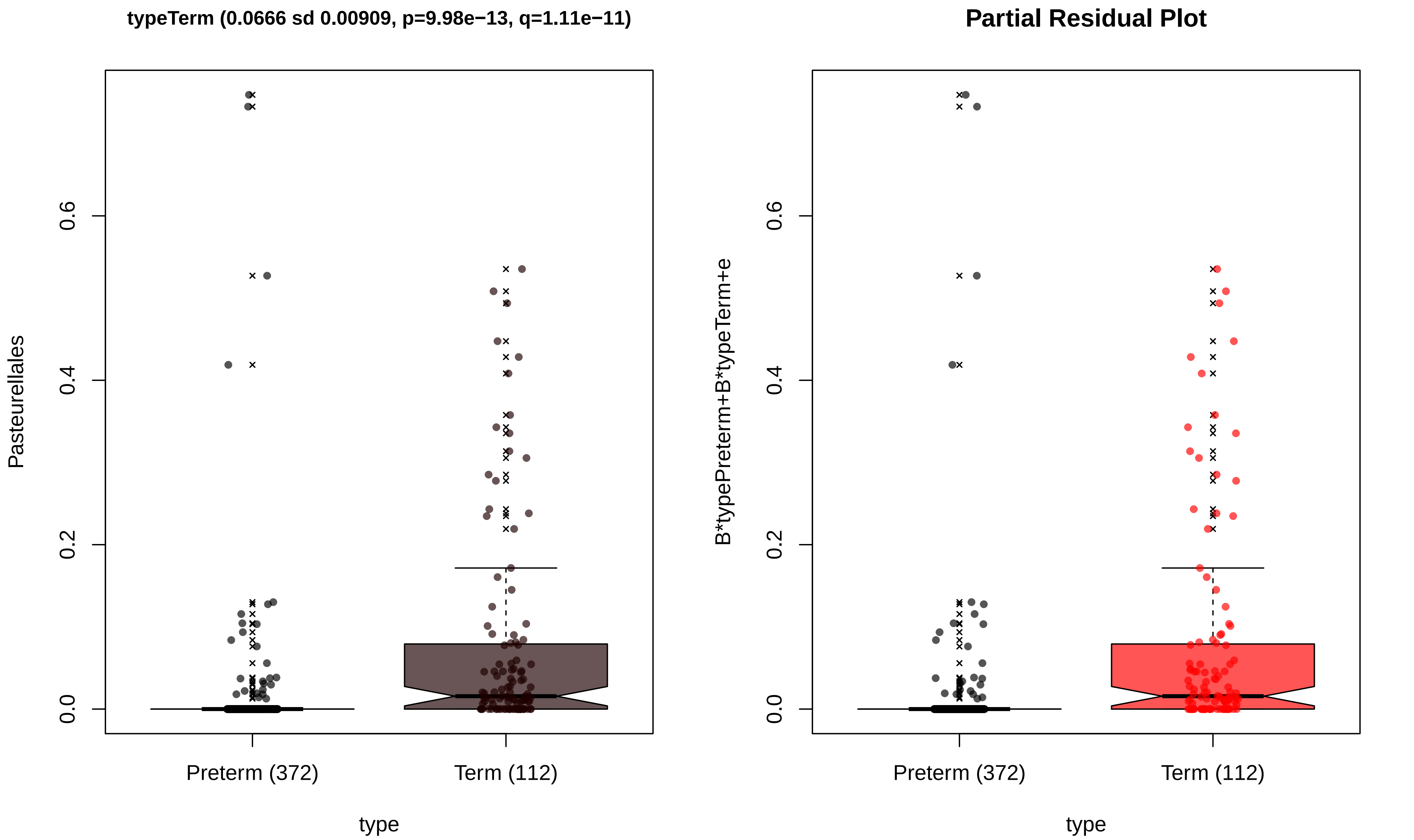
typeTerm (0.0666 sd 0.00909, p=9.98e−13, q=1.11e−11)
Pasteurellales
0.0
0.2
0.4
0.6
Preterm (372)
Term (112)
type
Partial Residual Plot
B*typePreterm+B*typeTerm+e
0.0
0.2
0.4
0.6
Preterm (372)
Term (112)
type

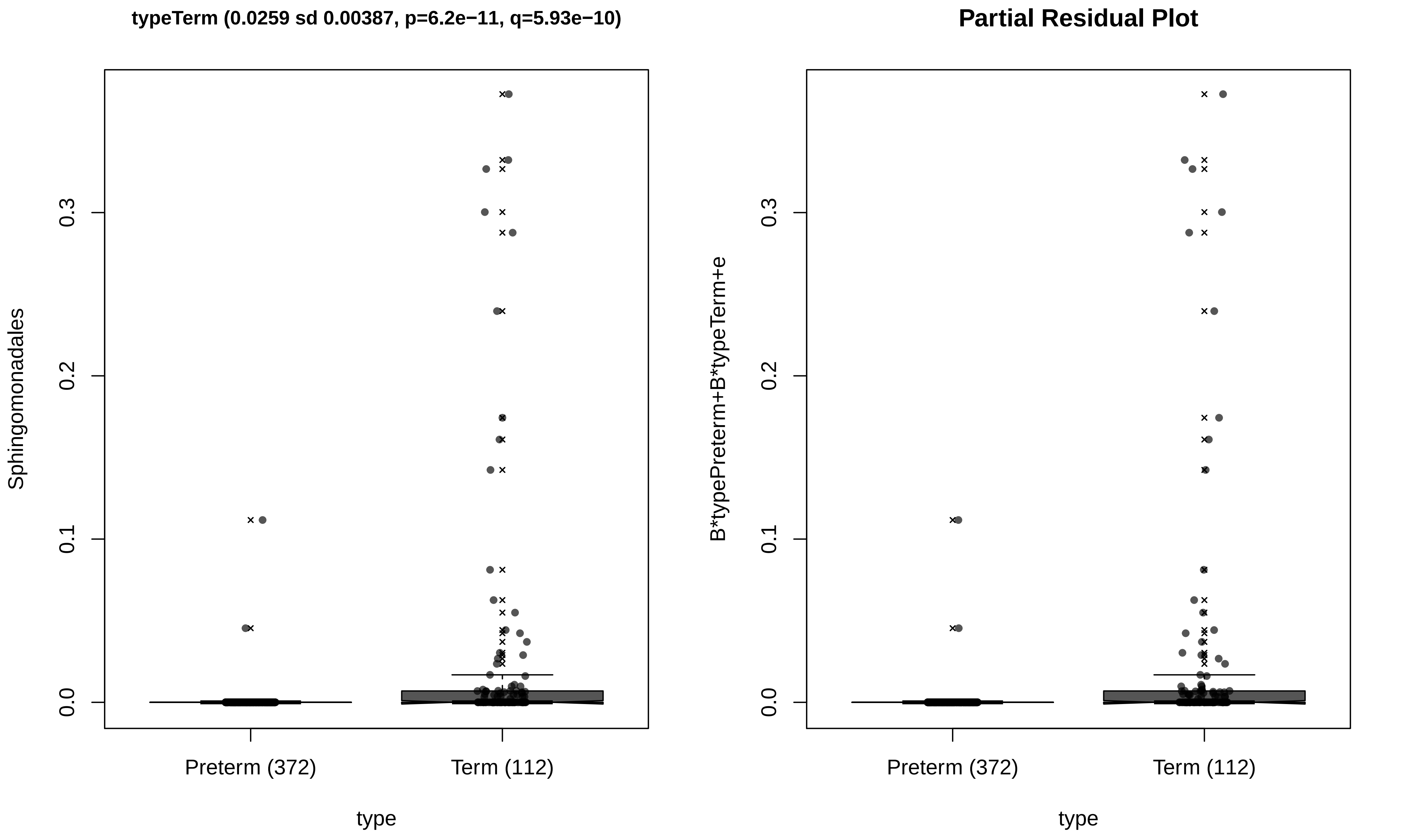

typeTerm (0.0259 sd 0.00387, p=6.2e−11, q=5.93e−10)
Sphingomonadales
0.0
0.1
0.2
0.3
Preterm (372)
Term (112)
type
Partial Residual Plot
B*typePreterm+B*typeTerm+e
0.0
0.1
0.2
0.3
Preterm (372)
Term (112)
type

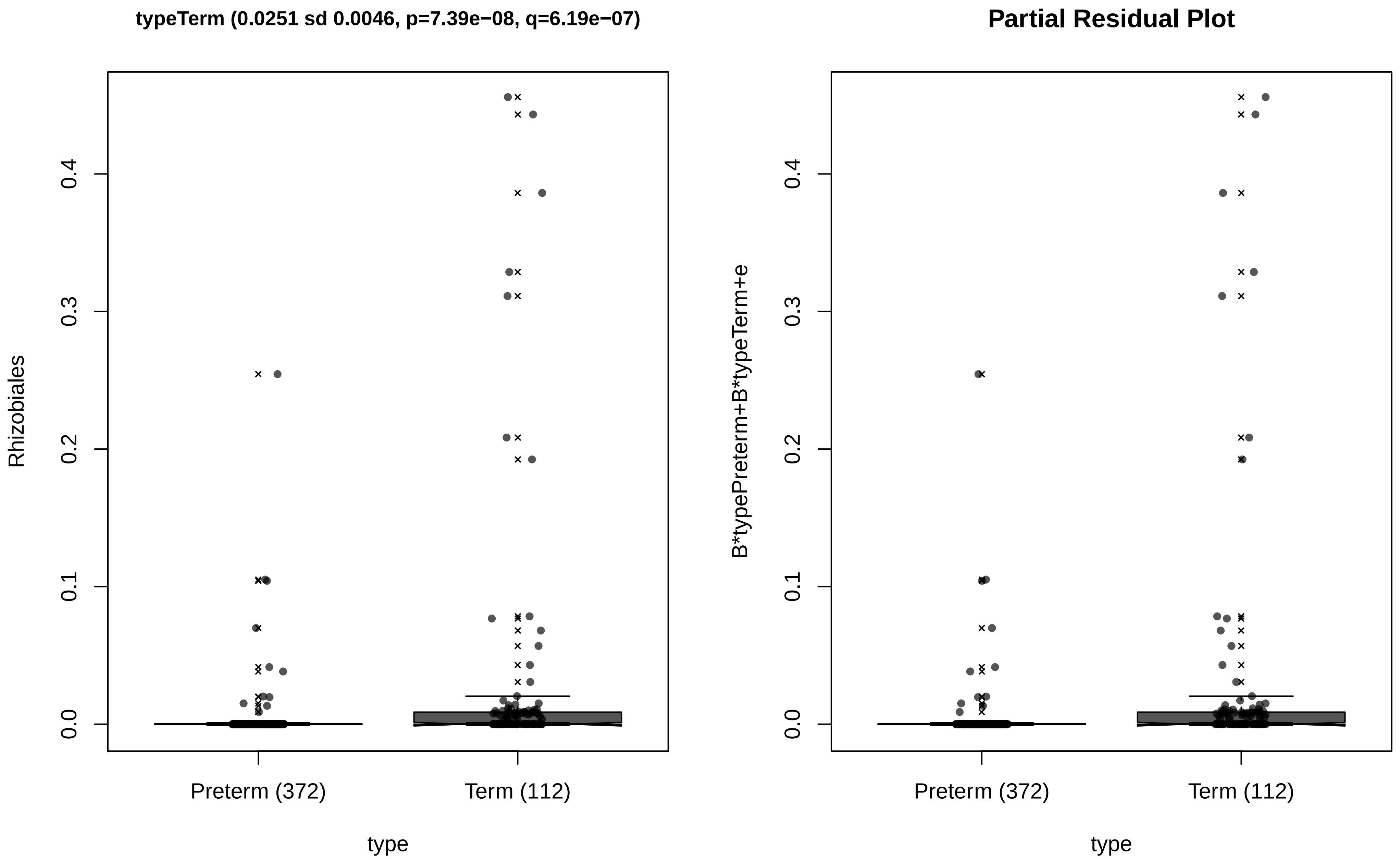
typeTerm (0.0251 sd 0.0046, p=7.39e−08, q=6.19e−07)
Rhizobiales
0.0
0.1
0.2
0.3
0.4
Preterm (372)
Term (112)
type
Partial Residual Plot
B*typePreterm+B*typeTerm+e
0.0
0.1
0.2
0.3
0.4
Preterm (372)
Term (112)
type

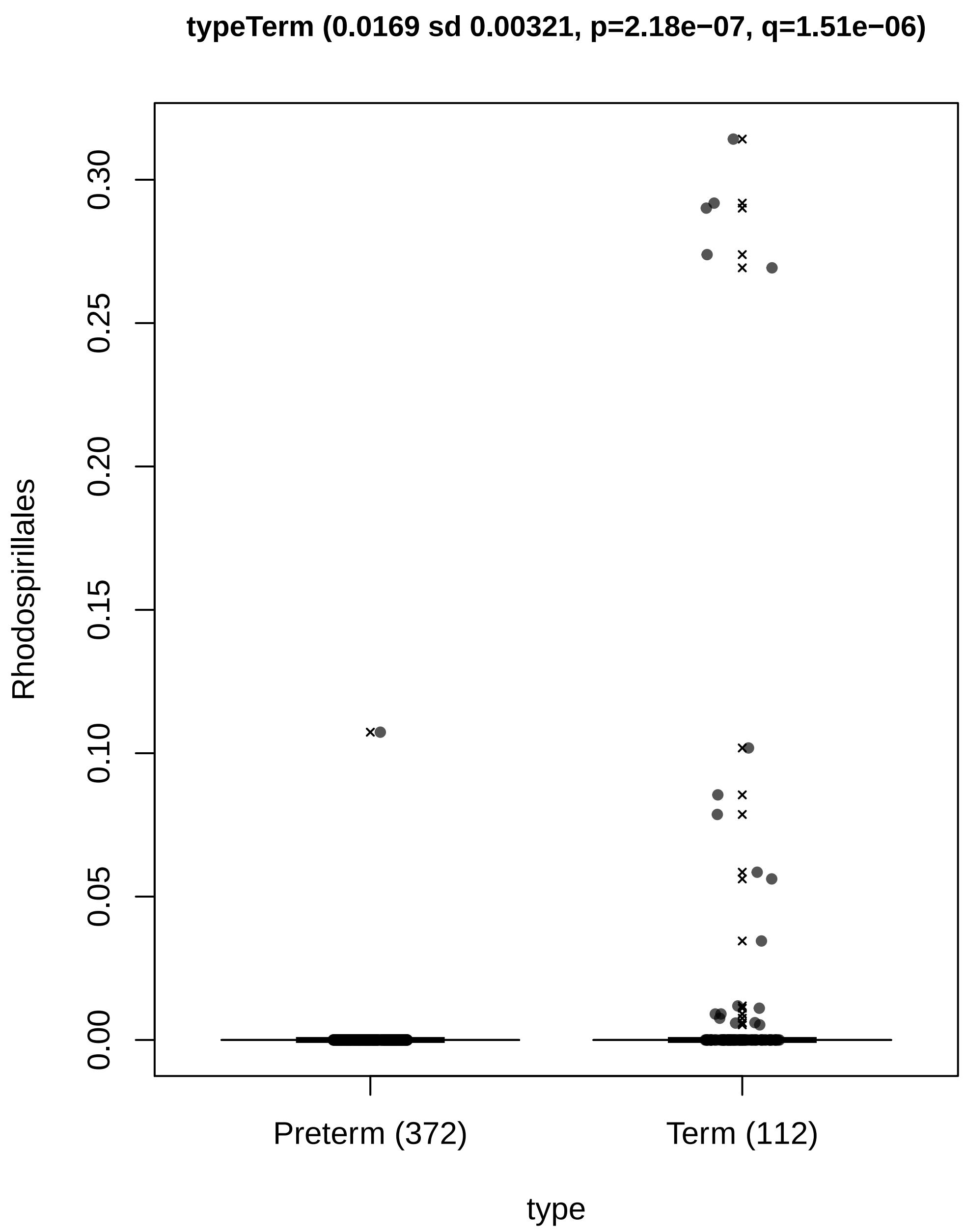

typeTerm (0.0169 sd 0.00321, p=2.18e−07, q=1.51e−06)
Rhodospirillales
0.00
0.05
0.10
0.15
0.20
0.25
0.30
Preterm (372)
Term (112)
type

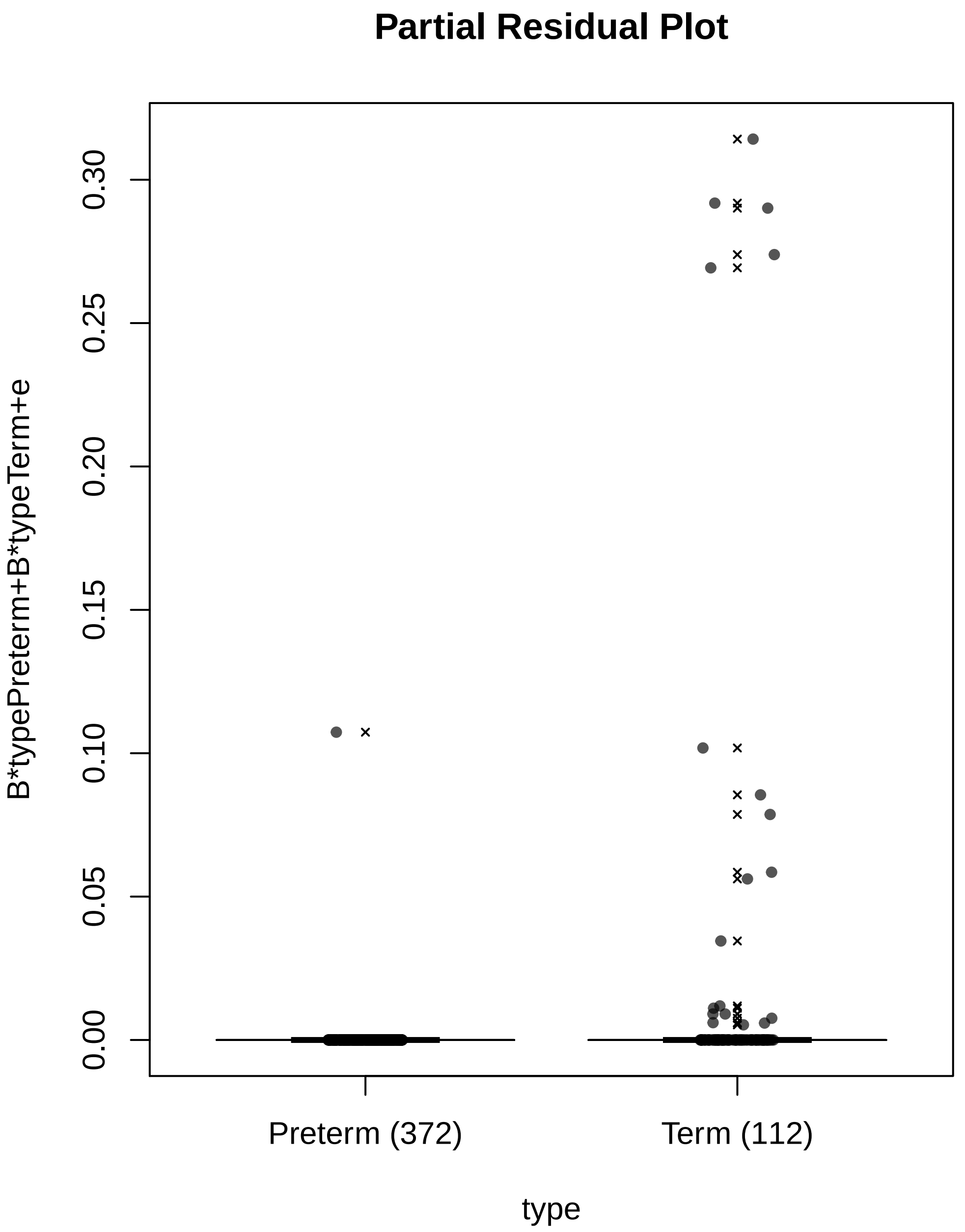

Partial Residual Plot
B*typePreterm+B*typeTerm+e
0.00
0.05
0.10
0.15
0.20
0.25
0.30
Preterm (372)
Term (112)
type

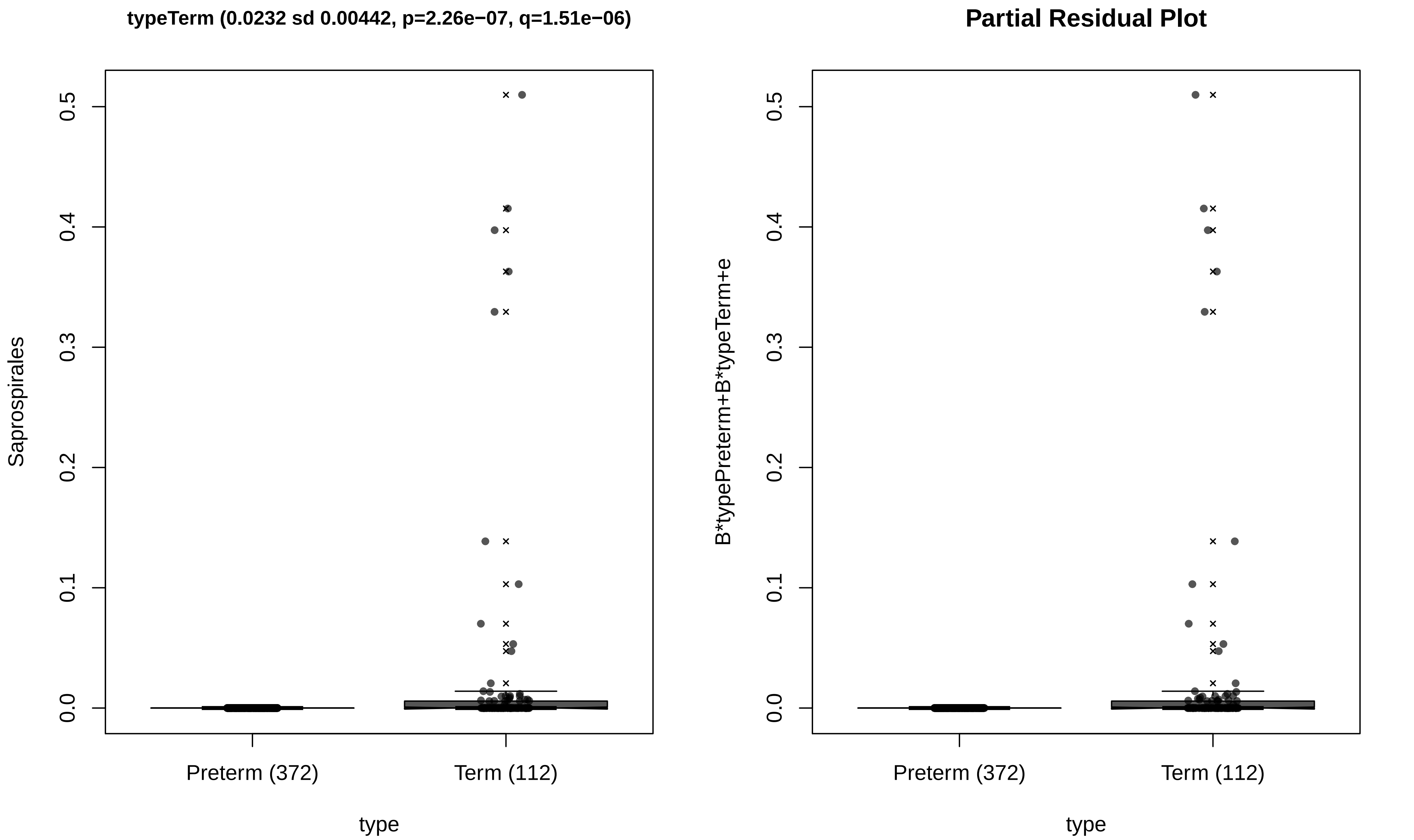
typeTerm (0.0232 sd 0.00442, p=2.26e−07, q=1.51e−06)
Saprospirales
0.0
0.1
0.2
0.3
0.4
0.5
Preterm (372)
Term (112)
type
Partial Residual Plot
B*typePreterm+B*typeTerm+e
0.0
0.1
0.2
0.3
0.4
0.5
Preterm (372)
Term (112)
type

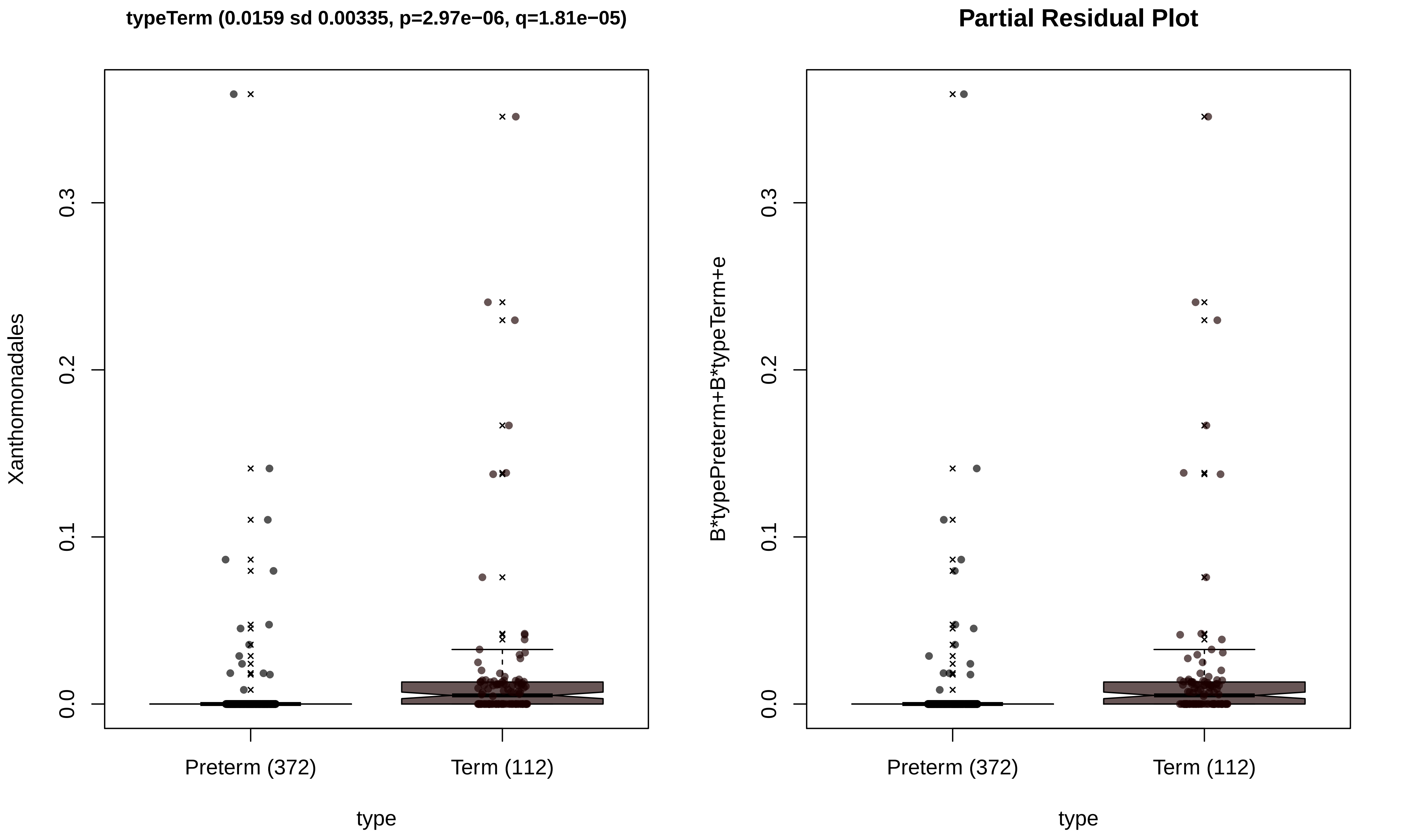

typeTerm (0.0159 sd 0.00335, p=2.97e−06, q=1.81e−05)
Xanthomonadales
0.0
0.1
0.2
0.3
Preterm (372)
Term (112)
type
Partial Residual Plot
B*typePreterm+B*typeTerm+e
0.0
0.1
0.2
0.3
Preterm (372)
Term (112)
type

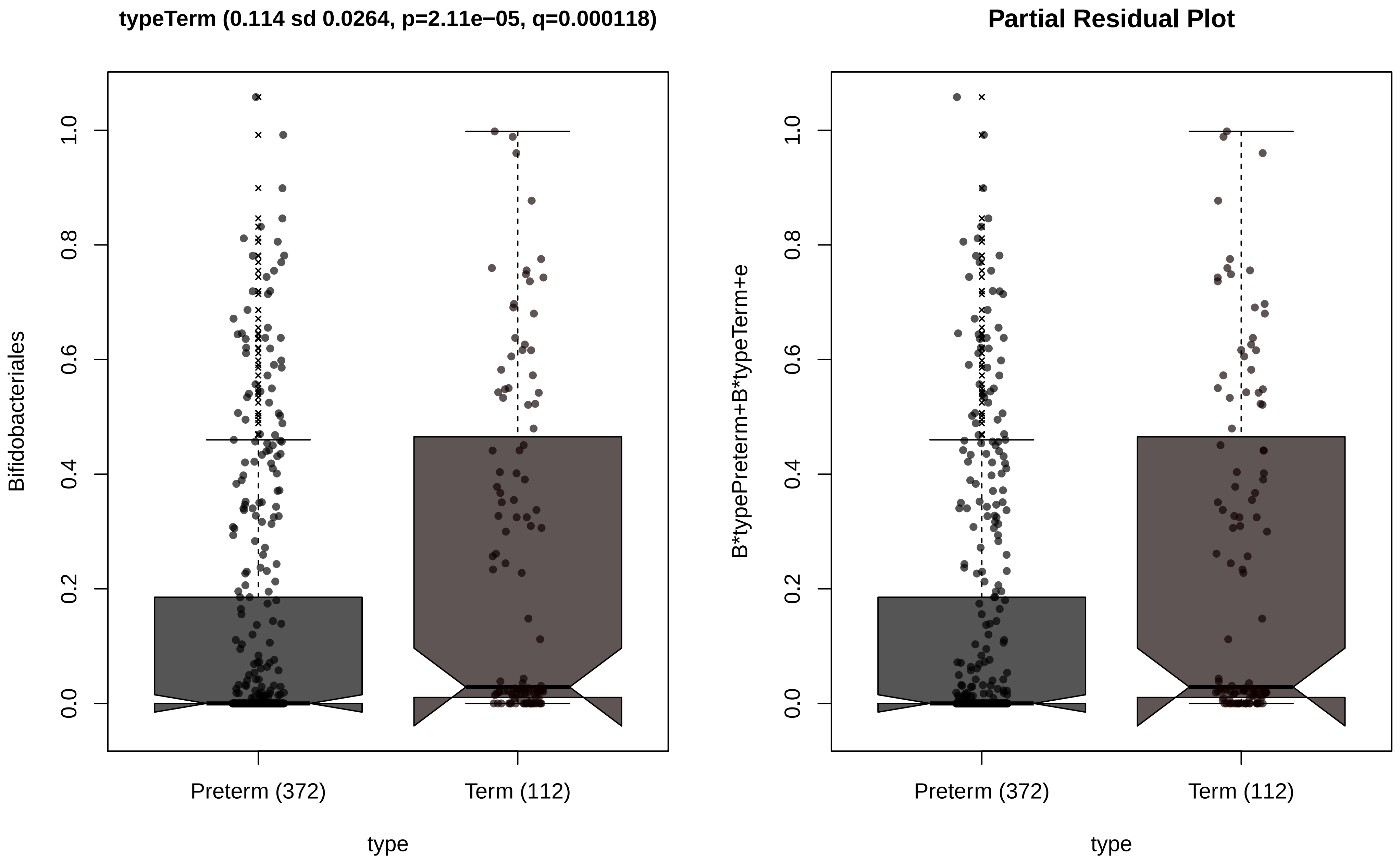

typeTerm (0.114 sd 0.0264, p=2.11e−05, q=0.000118)
Partial Residual Plot
Bifidobacteriales
B*typePreterm+B*typeTerm+e
0.0
0.2
0.4
0.6
0.8
1.0
Preterm (372)
Term (112)
type

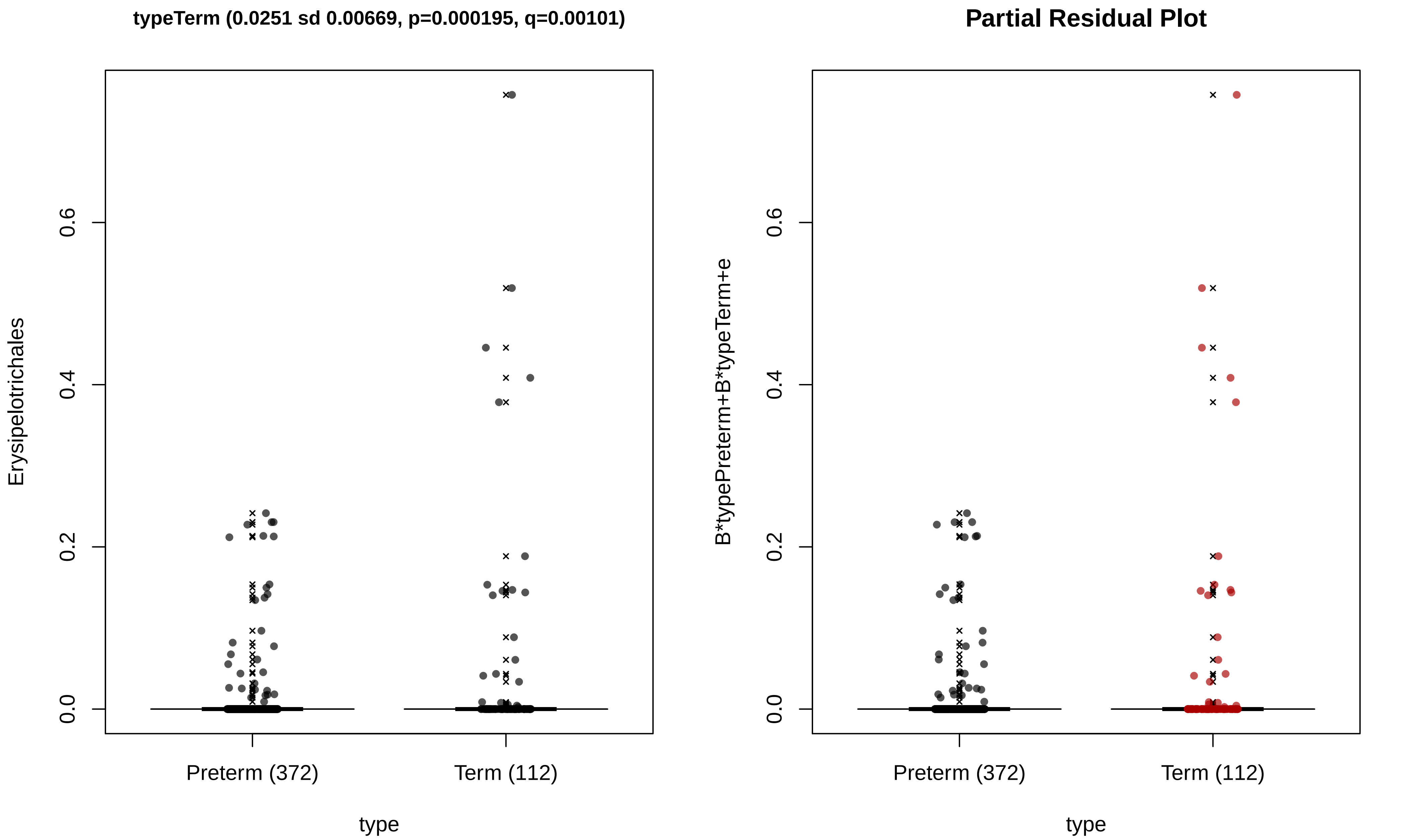
typeTerm (0.0251 sd 0.00669, p=0.000195, q=0.00101)
Partial Residual Plot
Erysipelotrichales
B*typePreterm+B*typeTerm+e
0.0
0.2
0.4
0.6
Preterm (372)
Term (112)
type

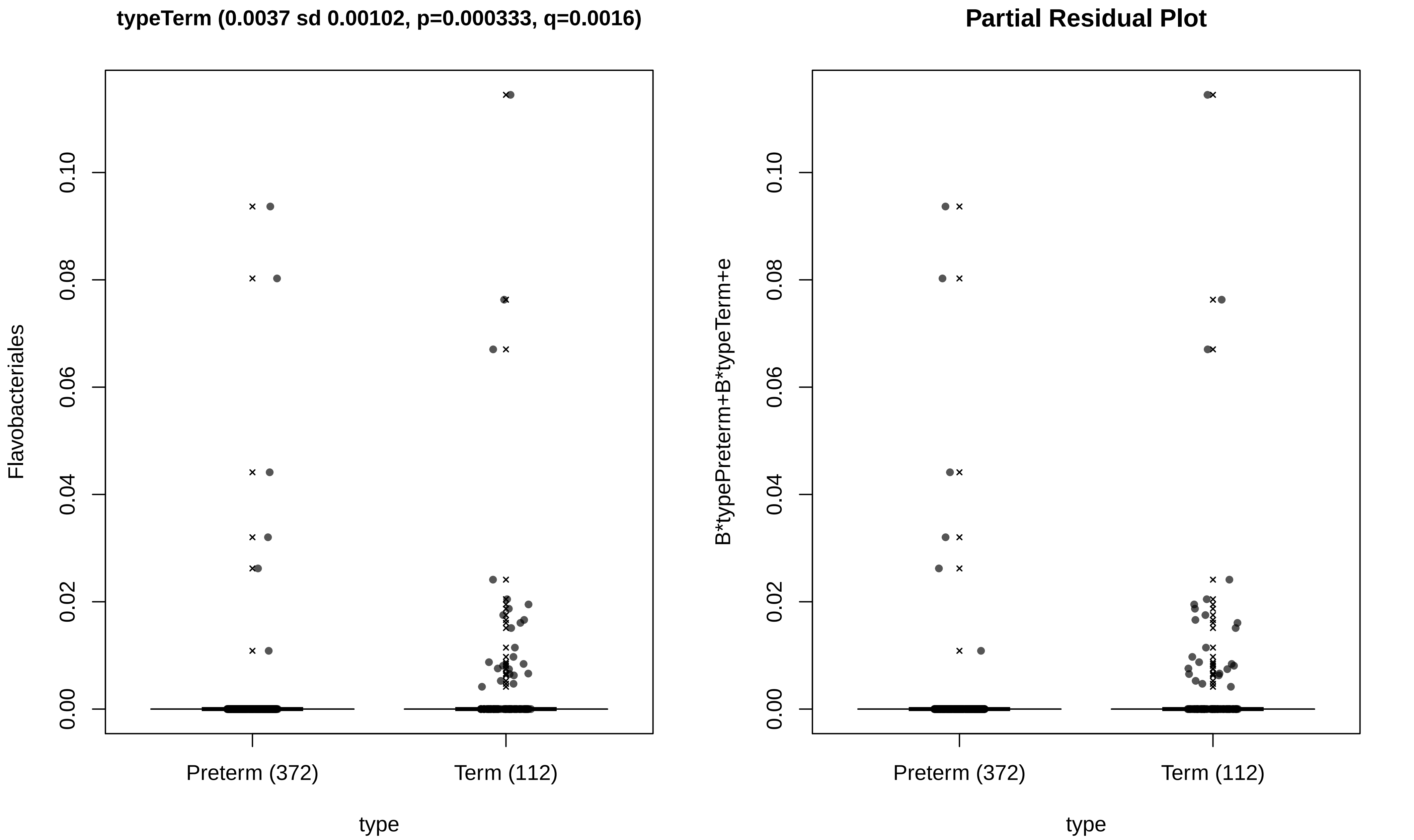
typeTerm (0.0037 sd 0.00102, p=0.000333, q=0.0016)
Partial Residual Plot
Flavobacteriales
B*typePreterm+B*typeTerm+e
0.00
0.02
0.04
0.06
0.08
0.10
Preterm (372)
Term (112)
type

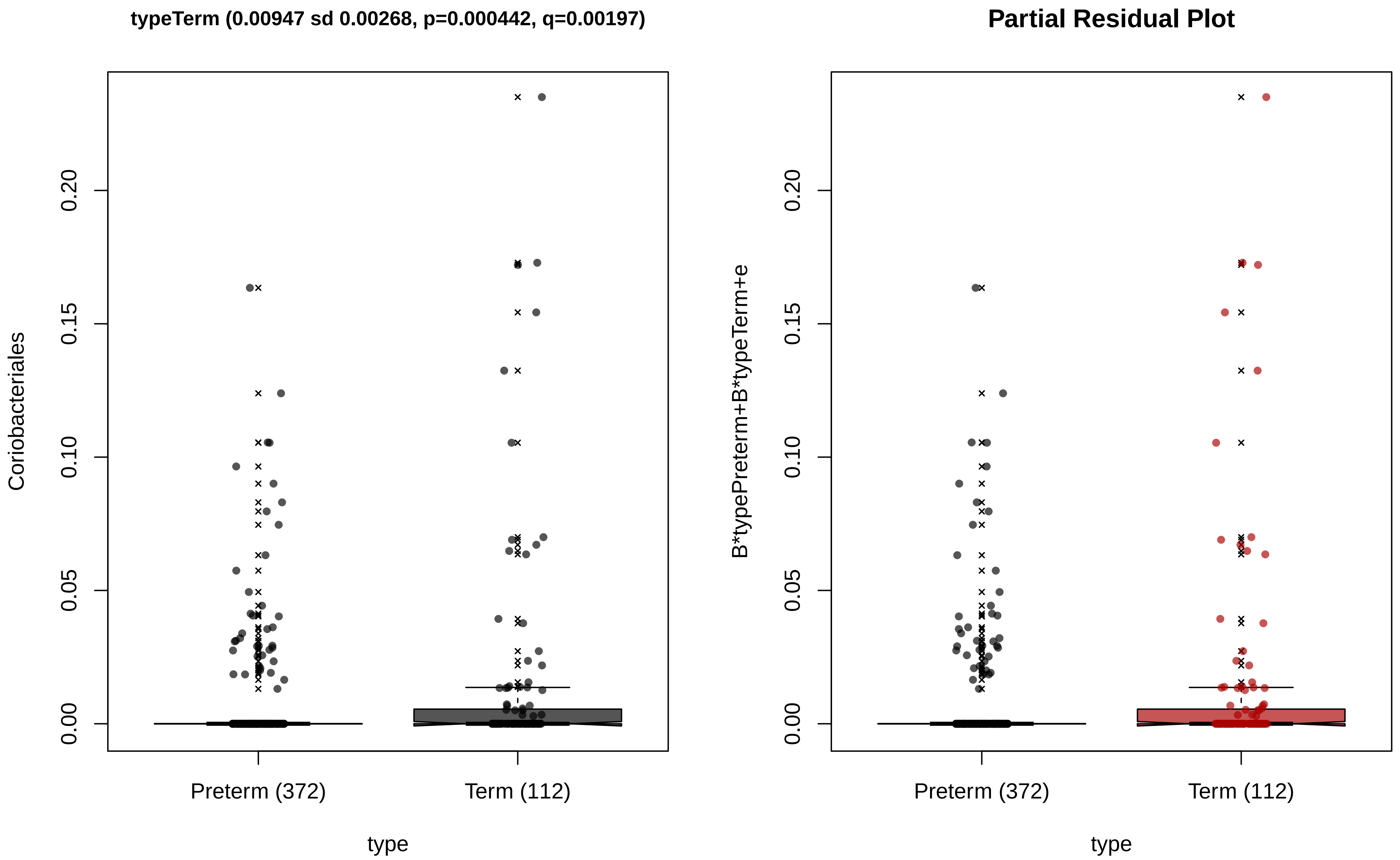

typeTerm (0.00947 sd 0.00268, p=0.000442, q=0.00197)
Coriobacteriales
0.00
0.05
0.10
0.15
0.20
Preterm (372)
Term (112)
type
Partial Residual Plot
B*typePreterm+B*typeTerm+e
0.00
0.05
0.10
0.15
0.20
Preterm (372)
Term (112)
type

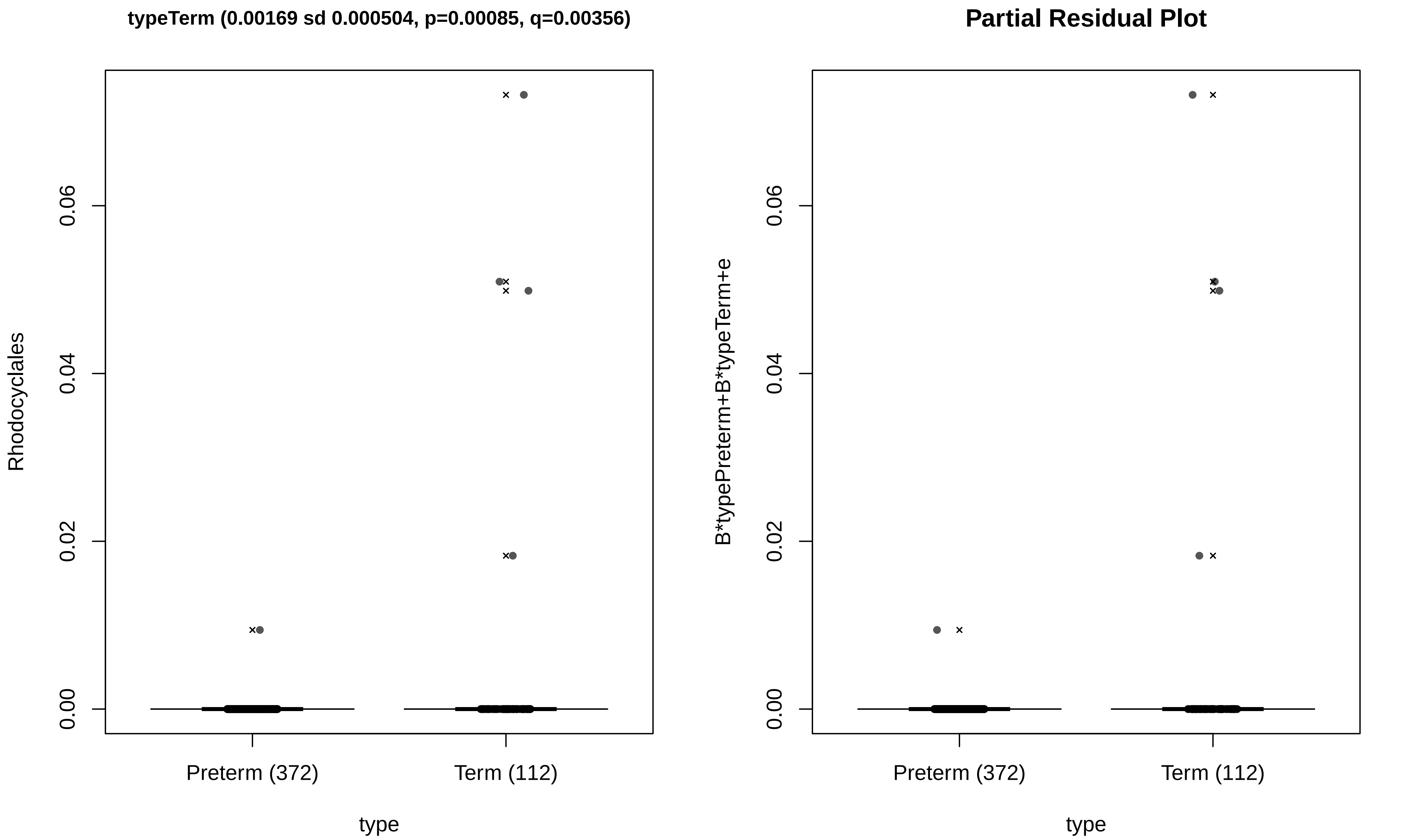
typeTerm (0.00169 sd 0.000504, p=0.00085, q=0.00356)
Rhodocyclales
0.00
0.02
0.04
0.06
Preterm (372)
Term (112)
type
Partial Residual Plot
B*typePreterm+B*typeTerm+e
0.00
0.02
0.04
0.06
Preterm (372)
Term (112)
type

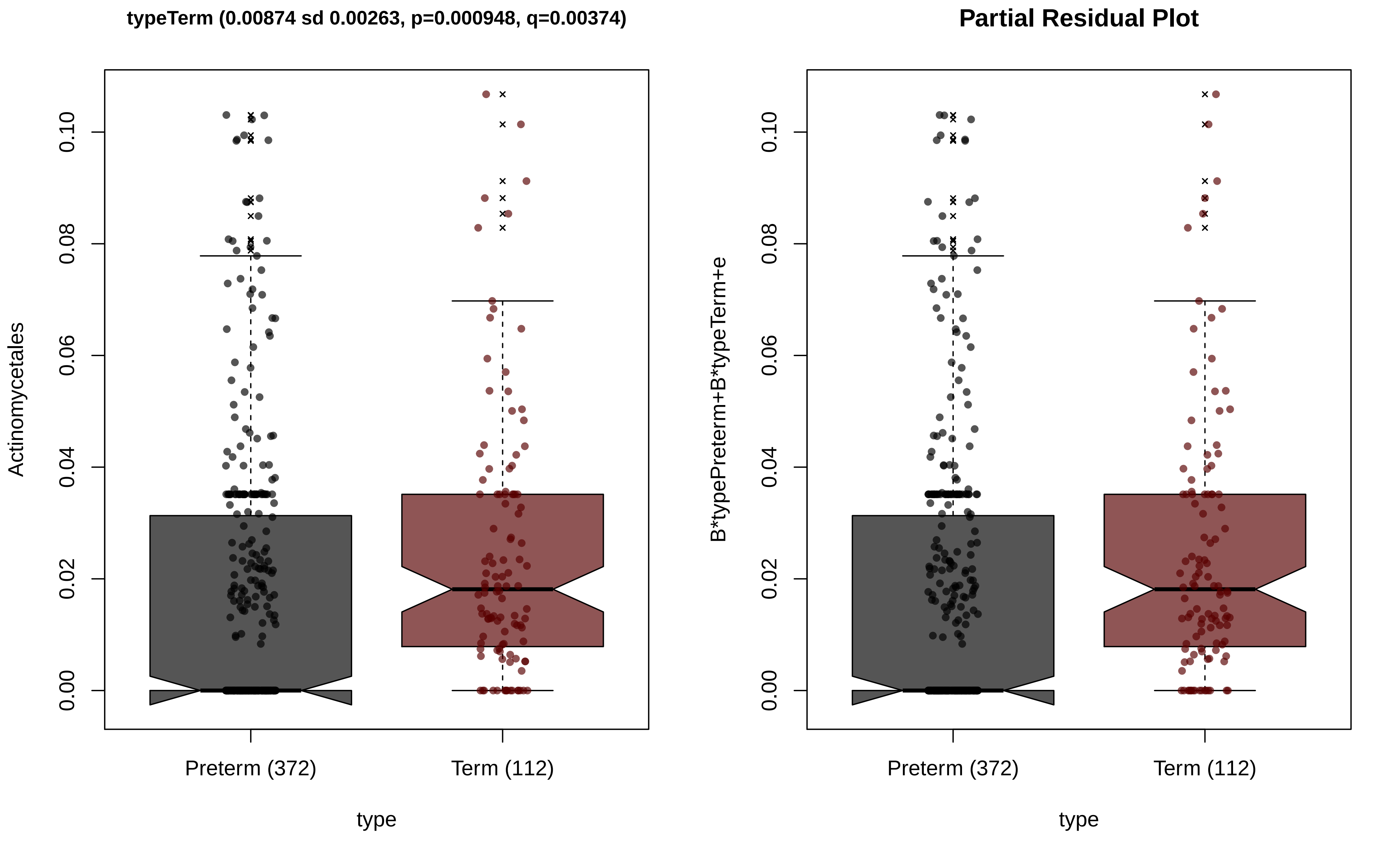
typeTerm (0.00874 sd 0.00263, p=0.000948, q=0.00374)
Actinomycetales
0.00
0.02
0.04
0.06
0.08
0.10
Preterm (372)
Term (112)
type
Partial Residual Plot
B*typePreterm+B*typeTerm+e
0.00
0.02
0.04
0.06
0.08
0.10
Preterm (372)
Term (112)
type

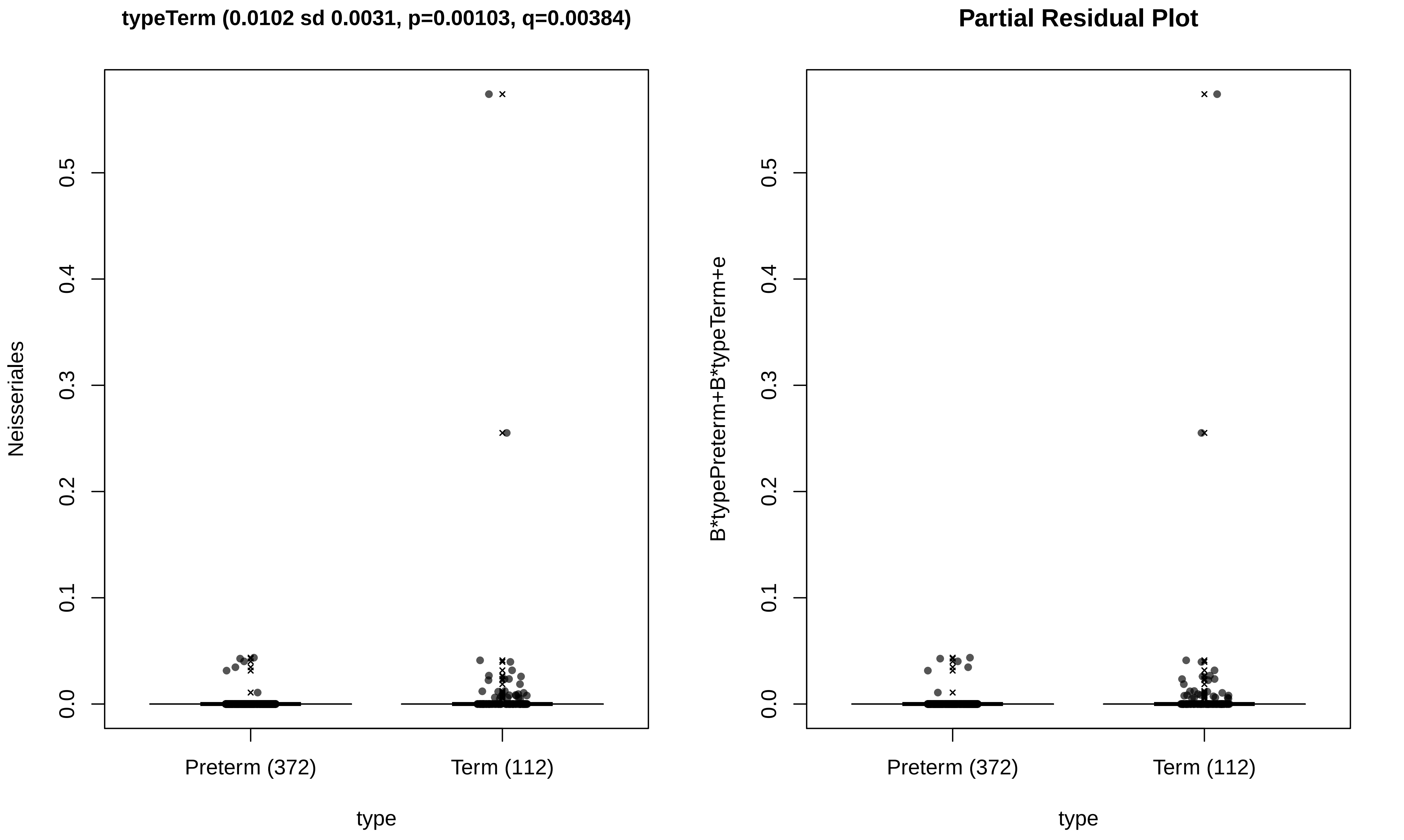
typeTerm (0.0102 sd 0.0031, p=0.00103, q=0.00384)
Neisseriales
0.0
0.1
0.2
0.3
0.4
0.5
Preterm (372)
Term (112)
type
Partial Residual Plot
B*typePreterm+B*typeTerm+e
0.0
0.1
0.2
0.3
0.4
0.5
Preterm (372)
Term (112)
type

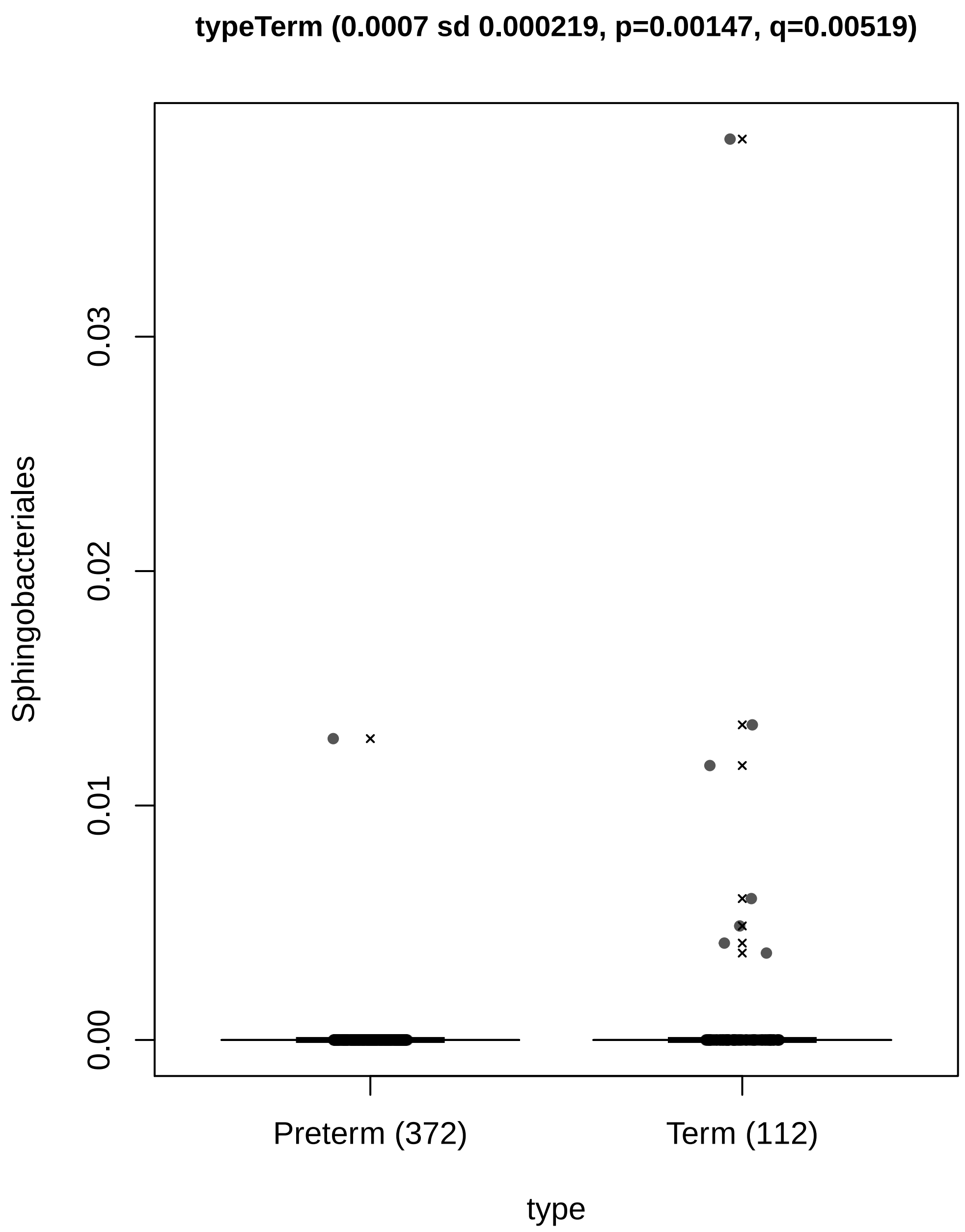

typeTerm (0.0007 sd 0.000219, p=0.00147, q=0.00519)
Sphingobacteriales
0.00
0.01
0.02
0.03
Preterm (372)
Term (112)
type

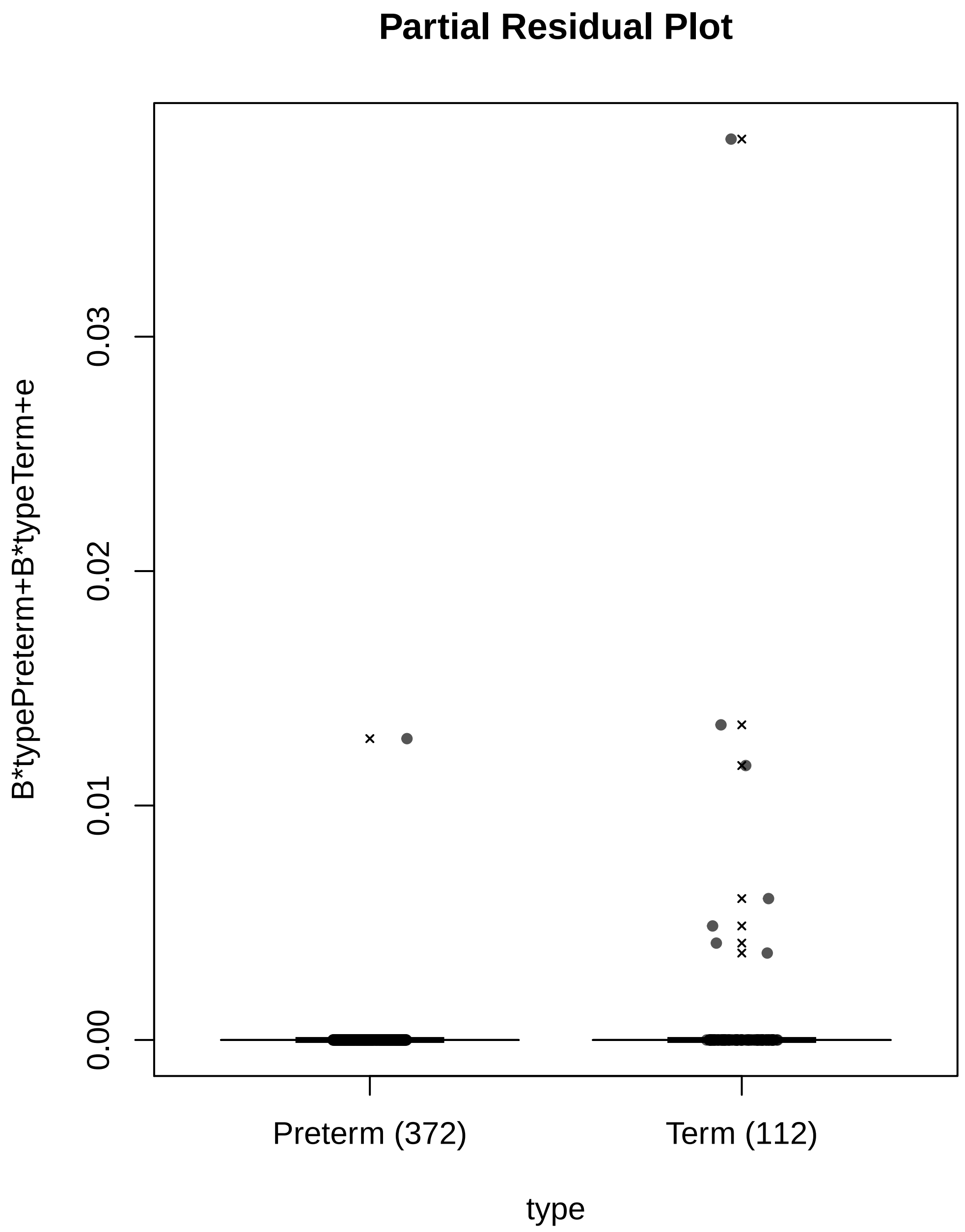

Partial Residual Plot
B*typePreterm+B*typeTerm+e
0.00
0.01
0.02
0.03
Preterm (372)
Term (112)
type

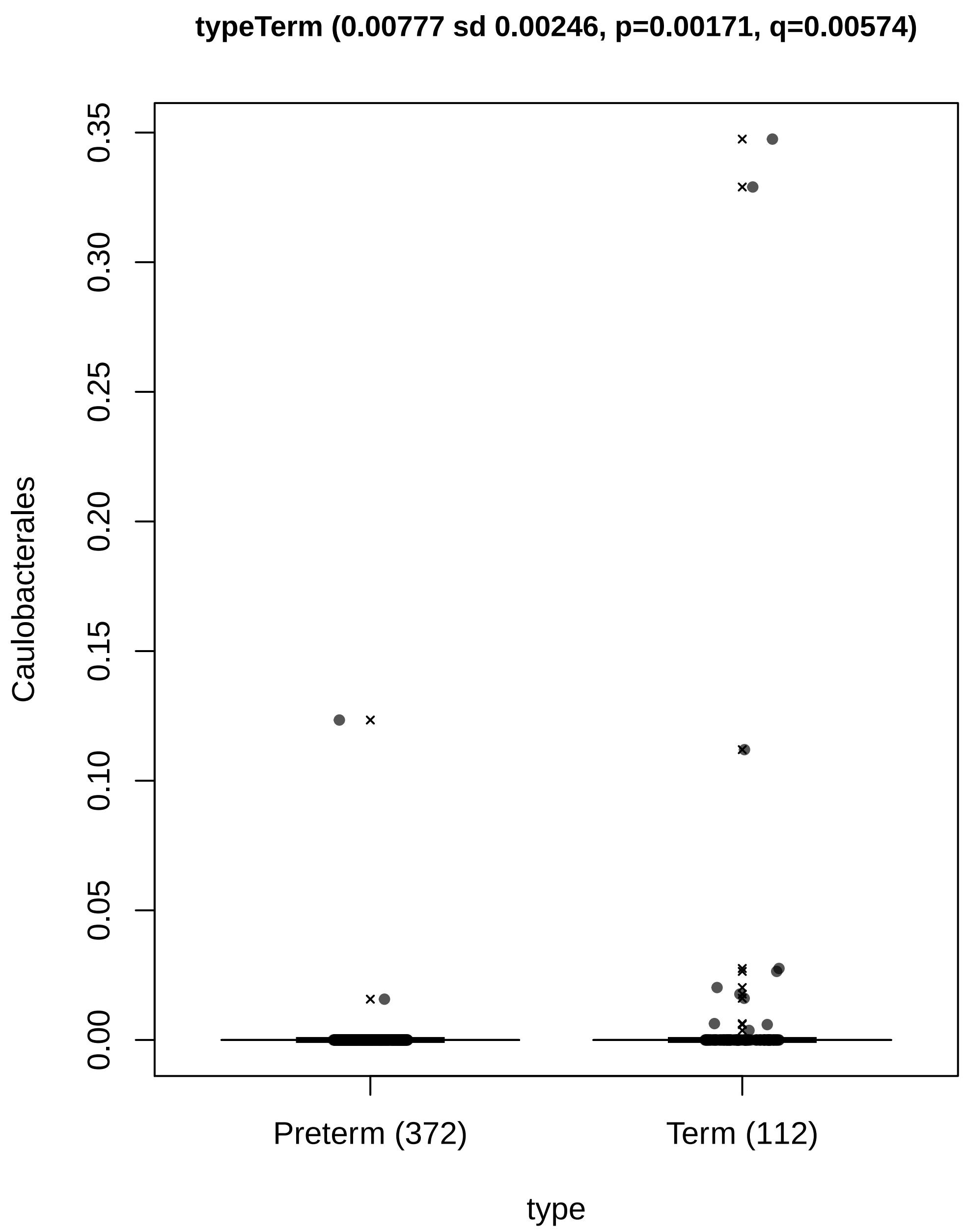
typeTerm (0.00777 sd 0.00246, p=0.00171, q=0.00574)
Caulobacterales
0.00
0.05
0.10
0.15
0.20
0.25
0.30
0.35
Preterm (372)
Term (112)
type

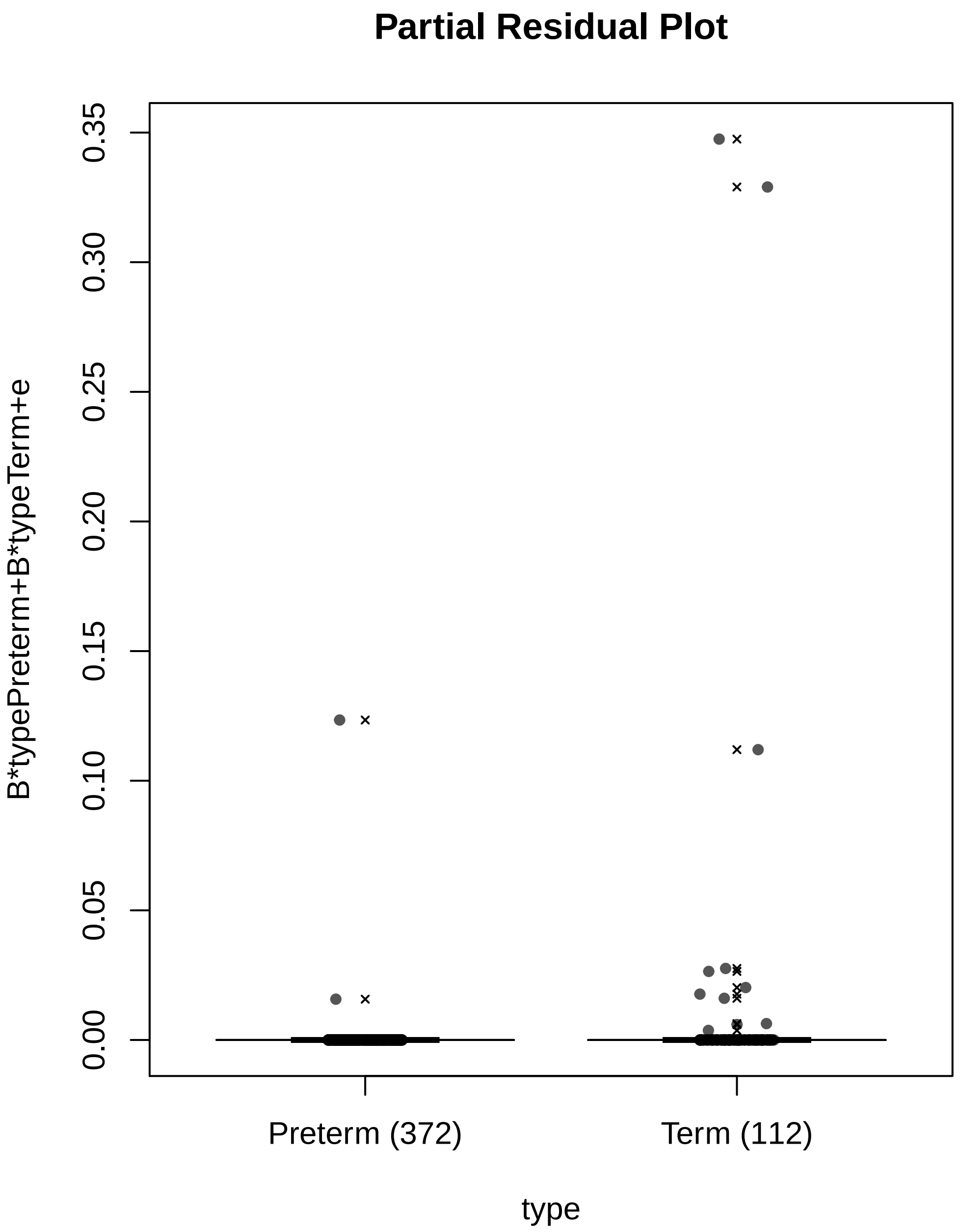
Partial Residual Plot
B*typePreterm+B*typeTerm+e
0.00
0.05
0.10
0.15
0.20
0.25
0.30
0.35
Preterm (372)
Term (112)
type

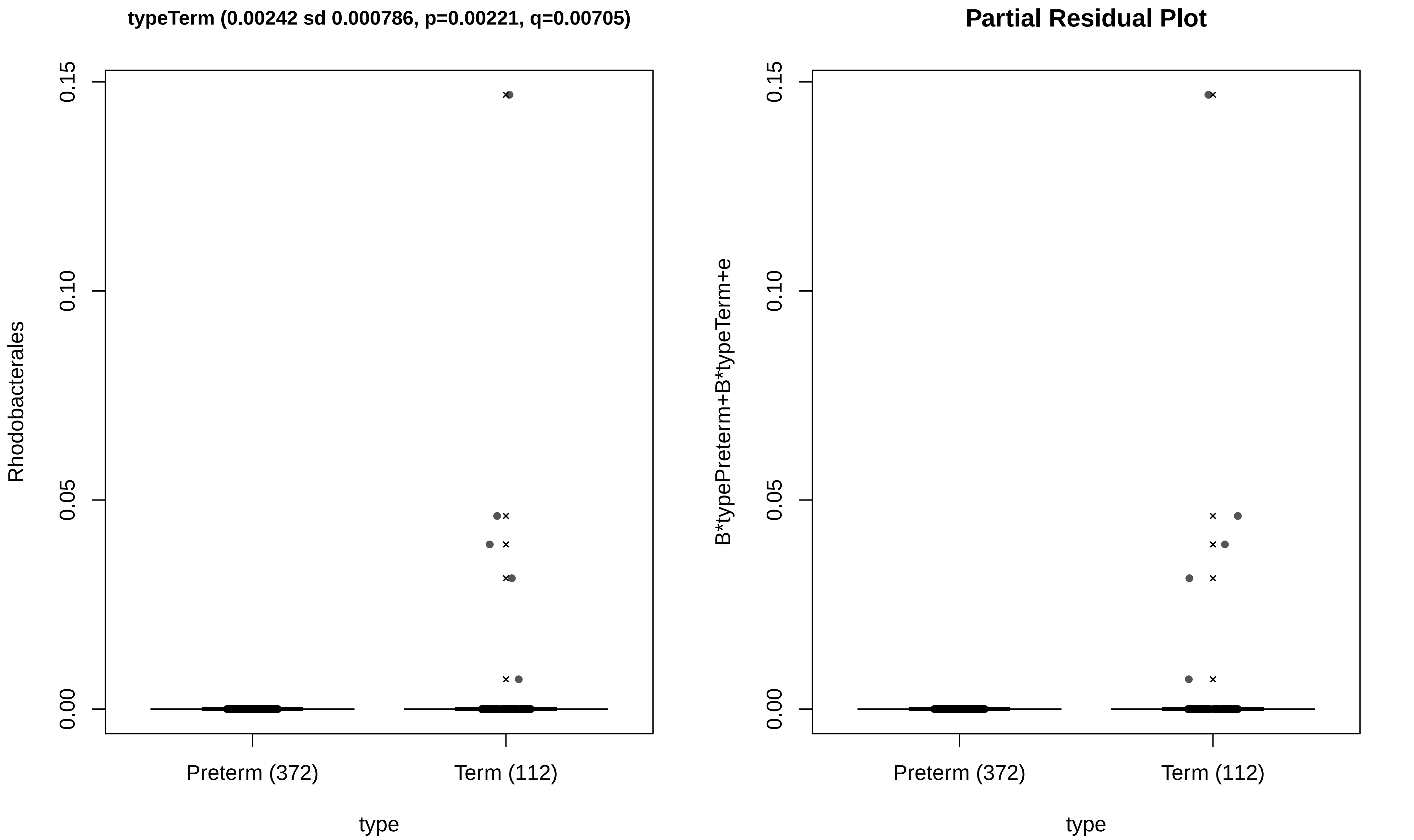

typeTerm (0.00242 sd 0.000786, p=0.00221, q=0.00705)
Rhodobacterales
0.15
0.10
0.05
0.00
Preterm (372)
Term (112)
type
Partial Residual Plot
B*typePreterm+B*typeTerm+e
0.15
0.10
0.05
0.00
Preterm (372)
Term (112)
type

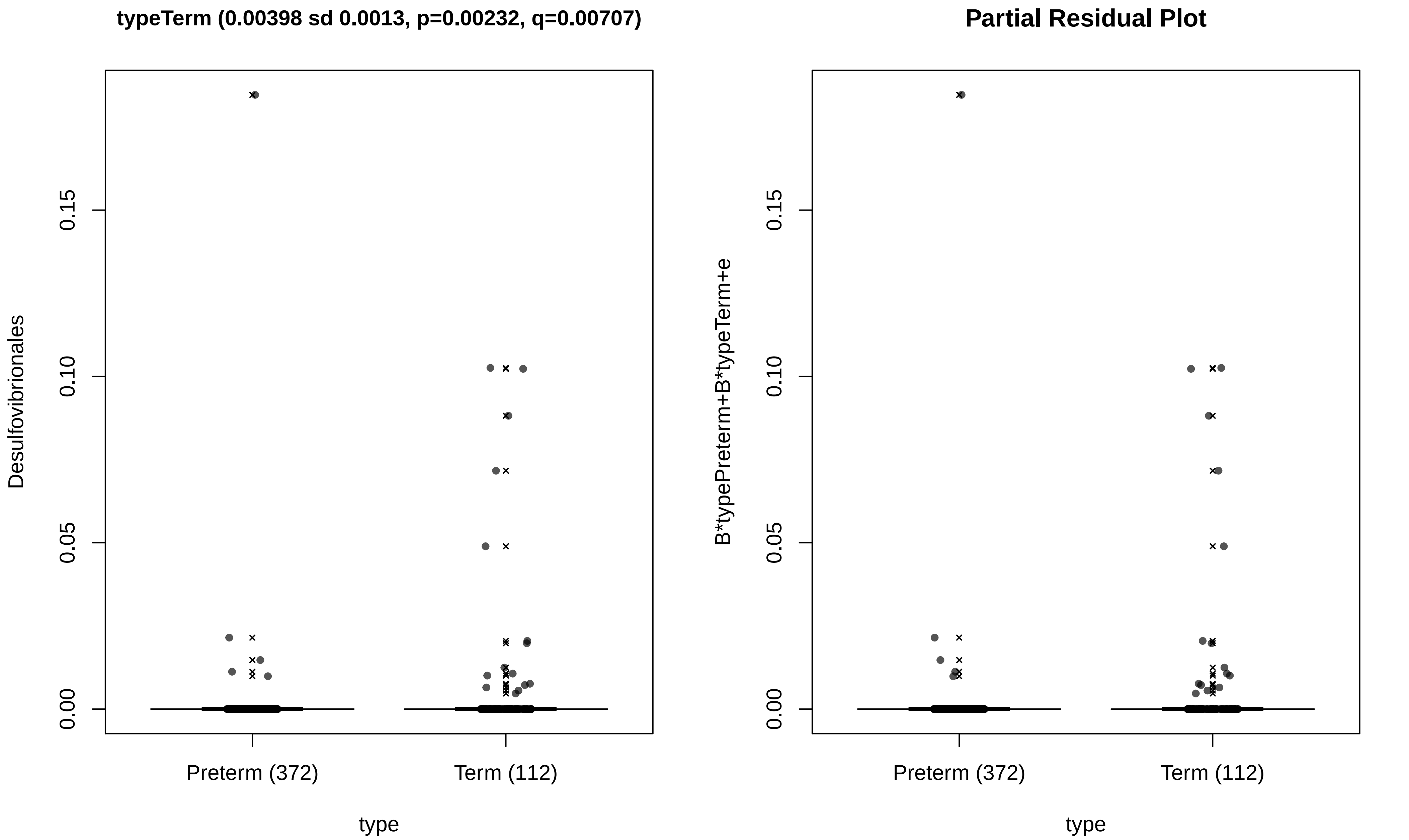
typeTerm (0.00398 sd 0.0013, p=0.00232, q=0.00707)
Desulfovibrionales
0.00
0.05
0.10
0.15
Preterm (372)
Term (112)
type
Partial Residual Plot
B*typePreterm+B*typeTerm+e
0.00
0.05
0.10
0.15
Preterm (372)
Term (112)
type

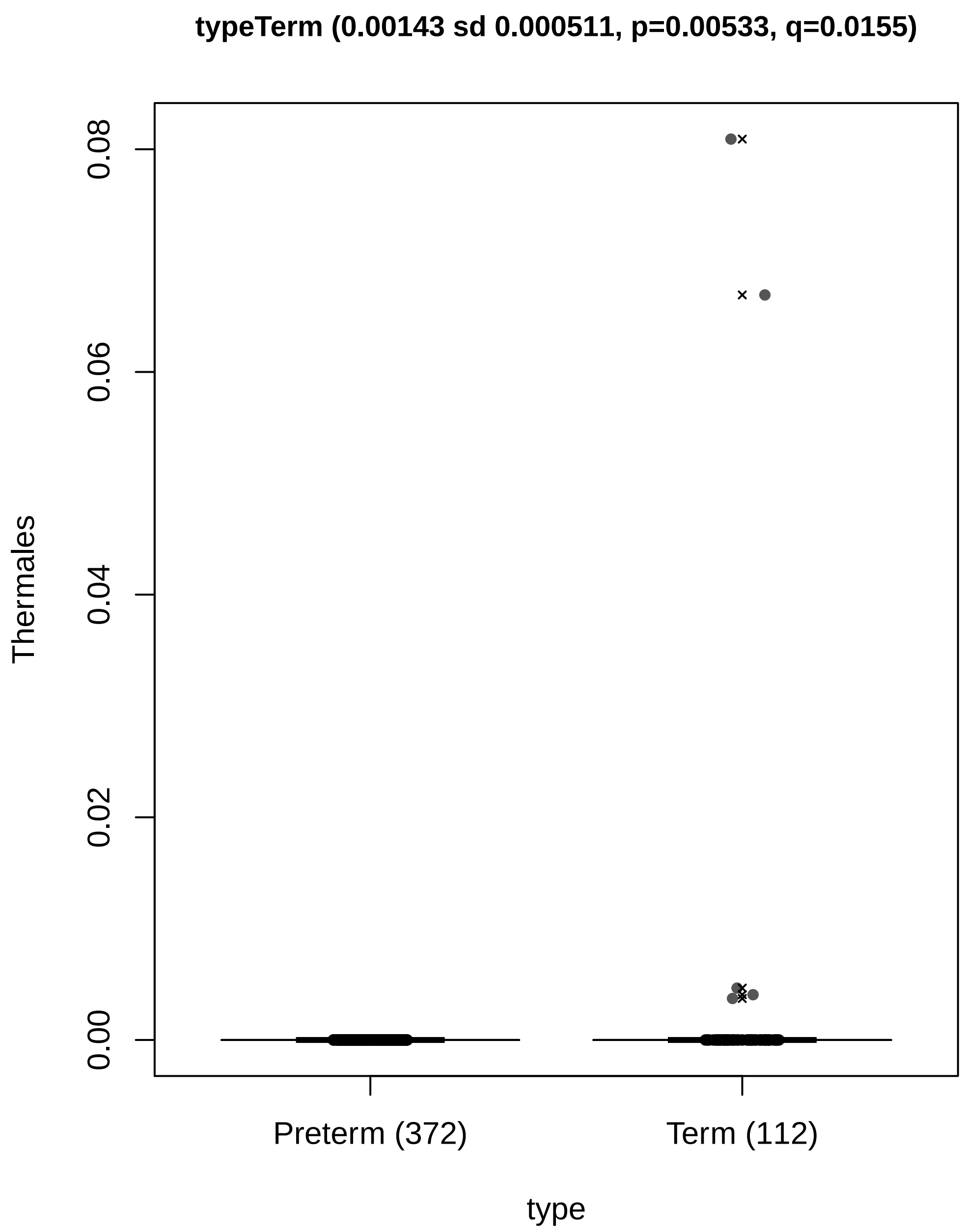
typeTerm (0.00143 sd 0.000511, p=0.00533, q=0.0155)
Thermales
0.00
0.02
0.04
0.06
0.08
Preterm (372)
Term (112)
type

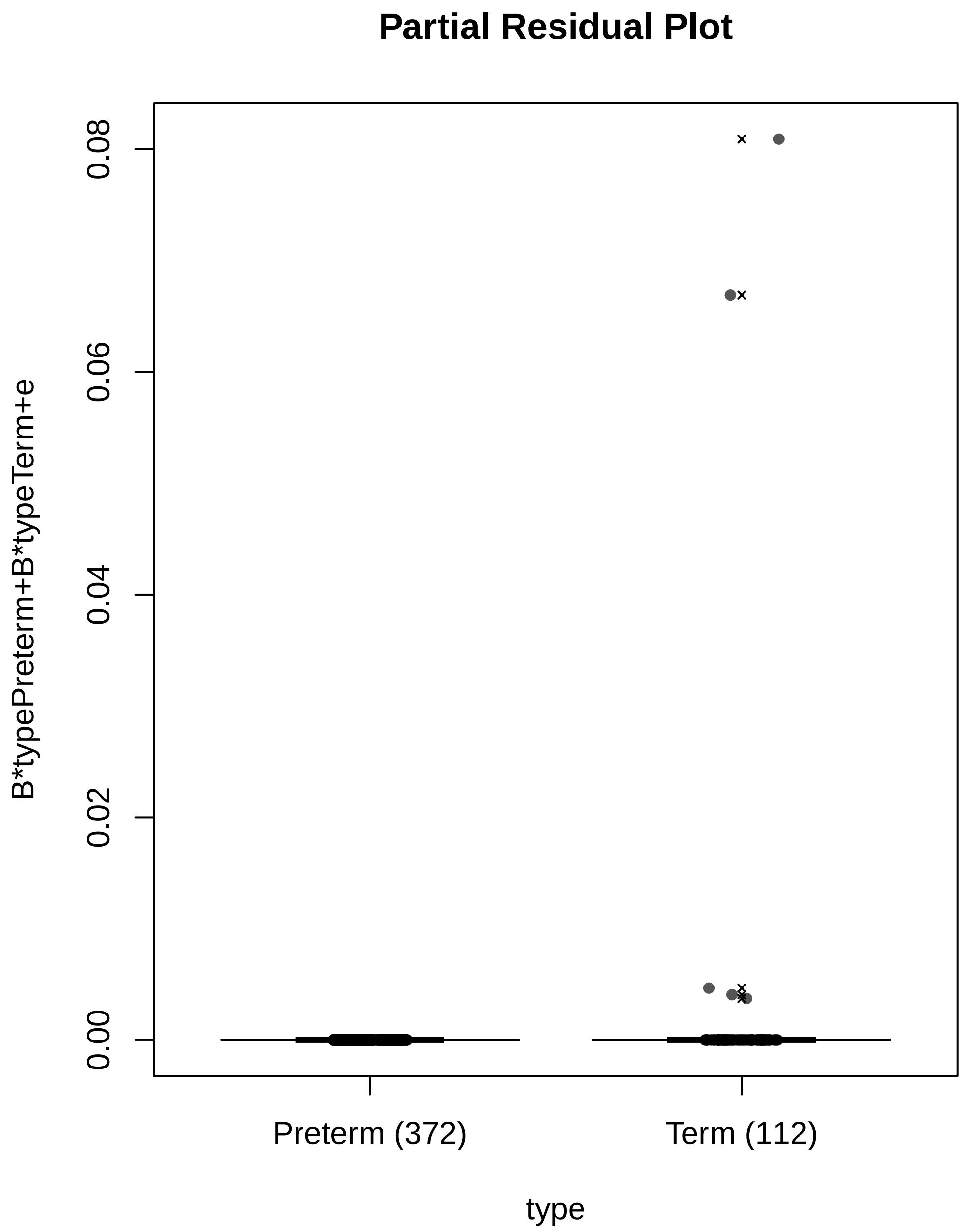
Partial Residual Plot
B*typePreterm+B*typeTerm+e
0.00
0.02
0.04
0.06
0.08
Preterm (372)
Term (112)
type

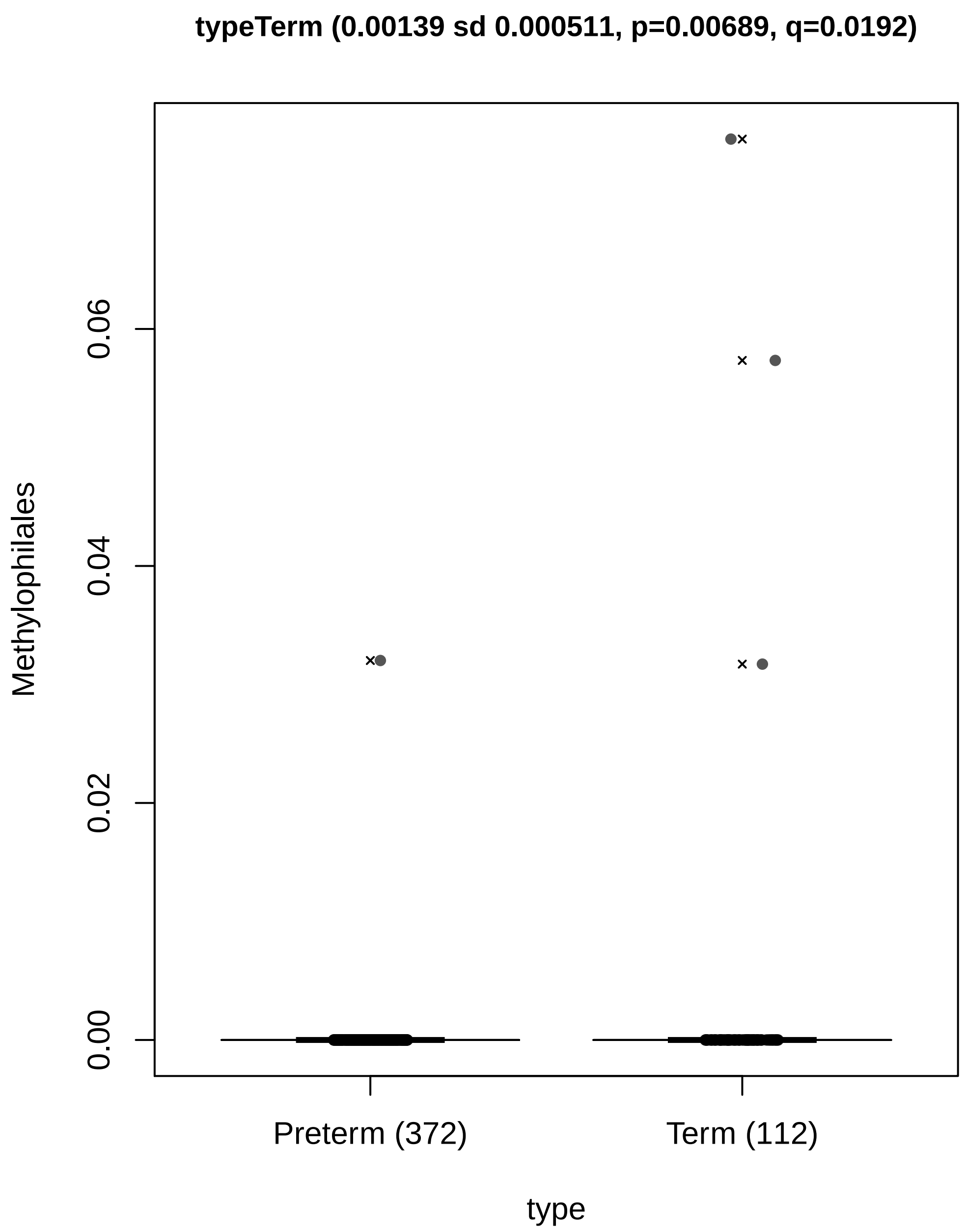
typeTerm (0.00139 sd 0.000511, p=0.00689, q=0.0192)
Methylophilales
0.00
0.02
0.04
0.06
Preterm (372)
Term (112)
type

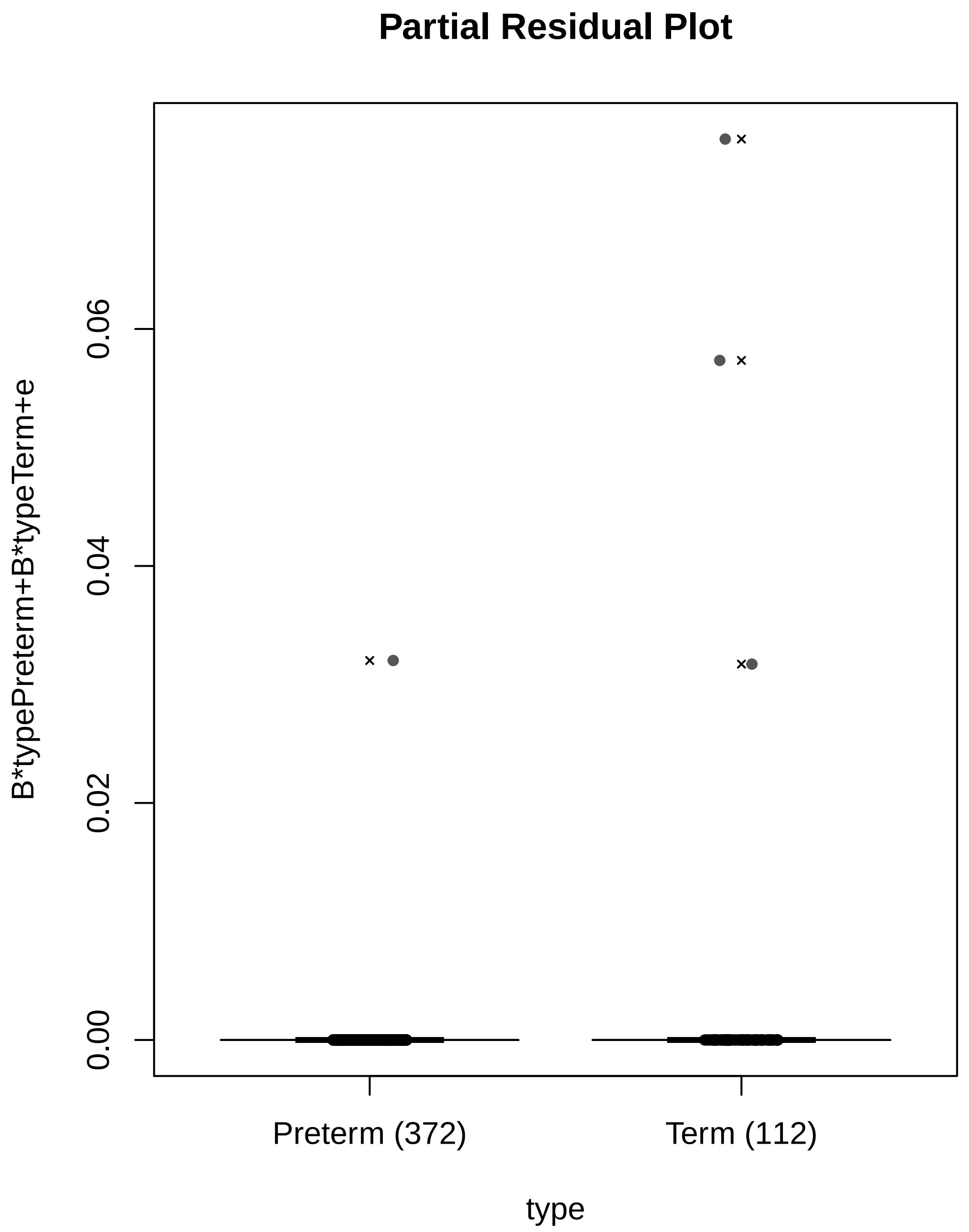
Partial Residual Plot
B*typePreterm+B*typeTerm+e
0.00
0.02
0.04
0.06
Preterm (372)
Term (112)
type

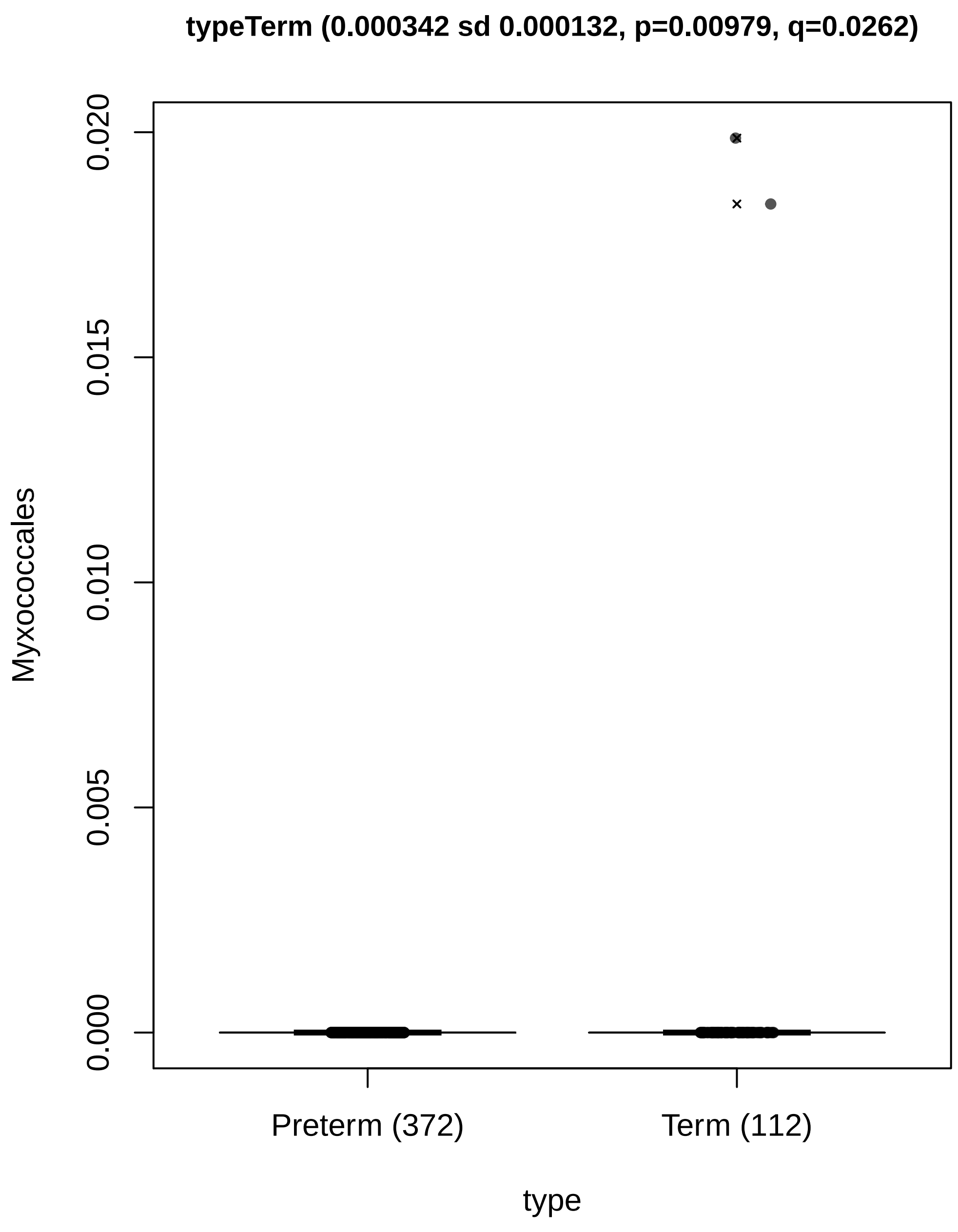
typeTerm (0.000342 sd 0.000132, p=0.00979, q=0.0262)
Myxococcales
0.000
0.005
0.010
0.015
0.020
Preterm (372)
Term (112)
type

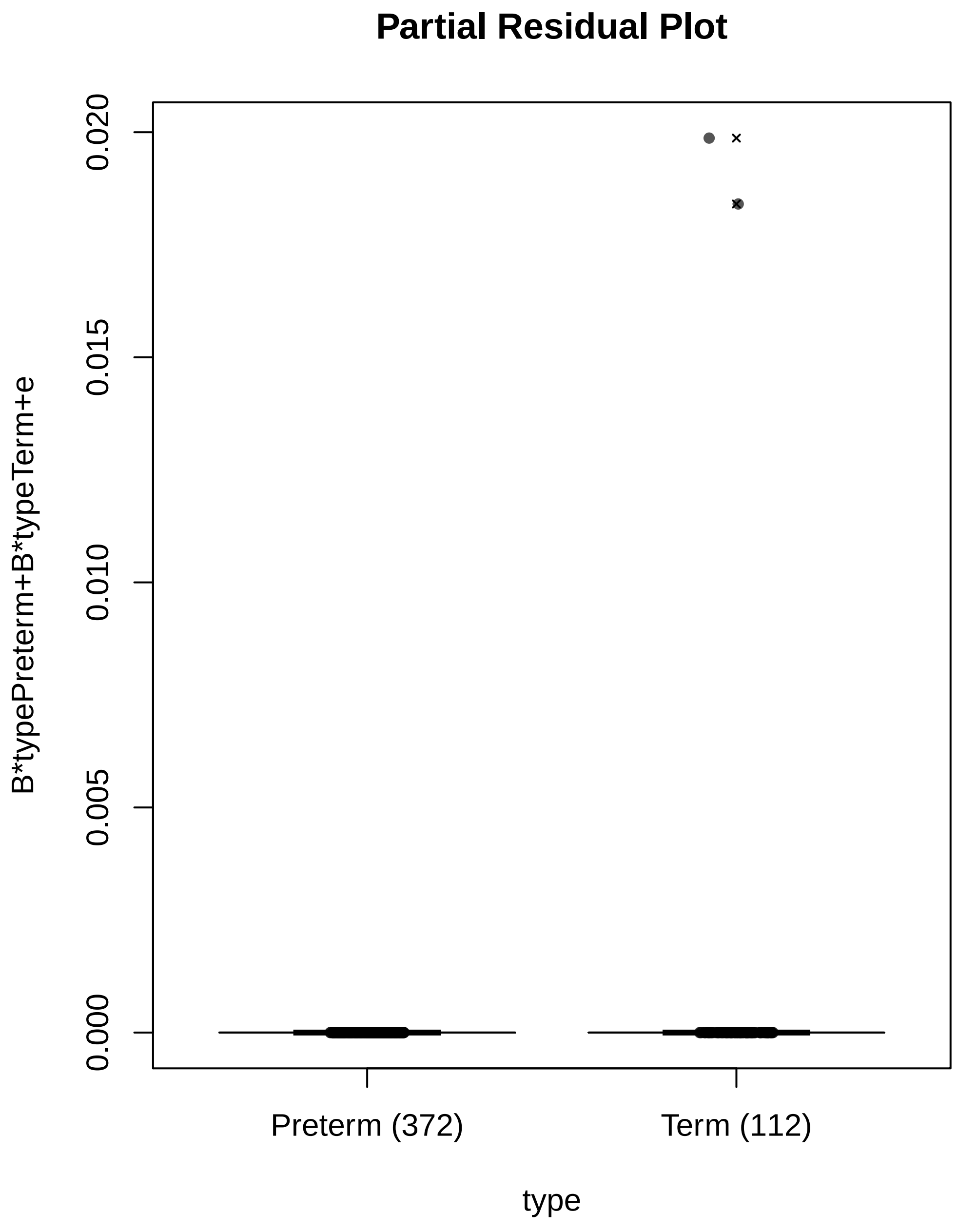
Partial Residual Plot
B*typePreterm+B*typeTerm+e
0.000
0.005
0.010
0.015
0.020
Preterm (372)
Term (112)
type

typeTerm (0.000786 sd 0.000309, p=0.0111, q=0.028)

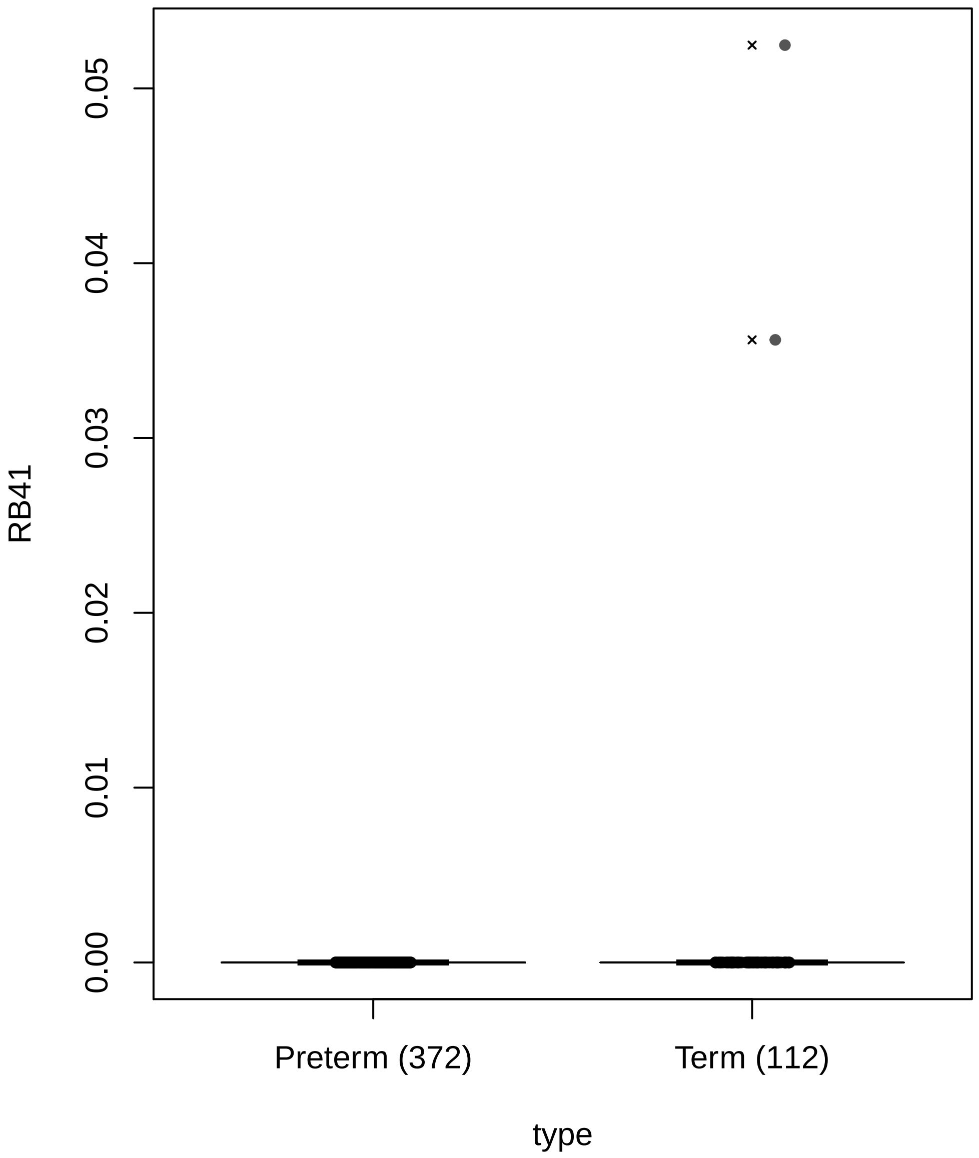

Partial Residual Plot

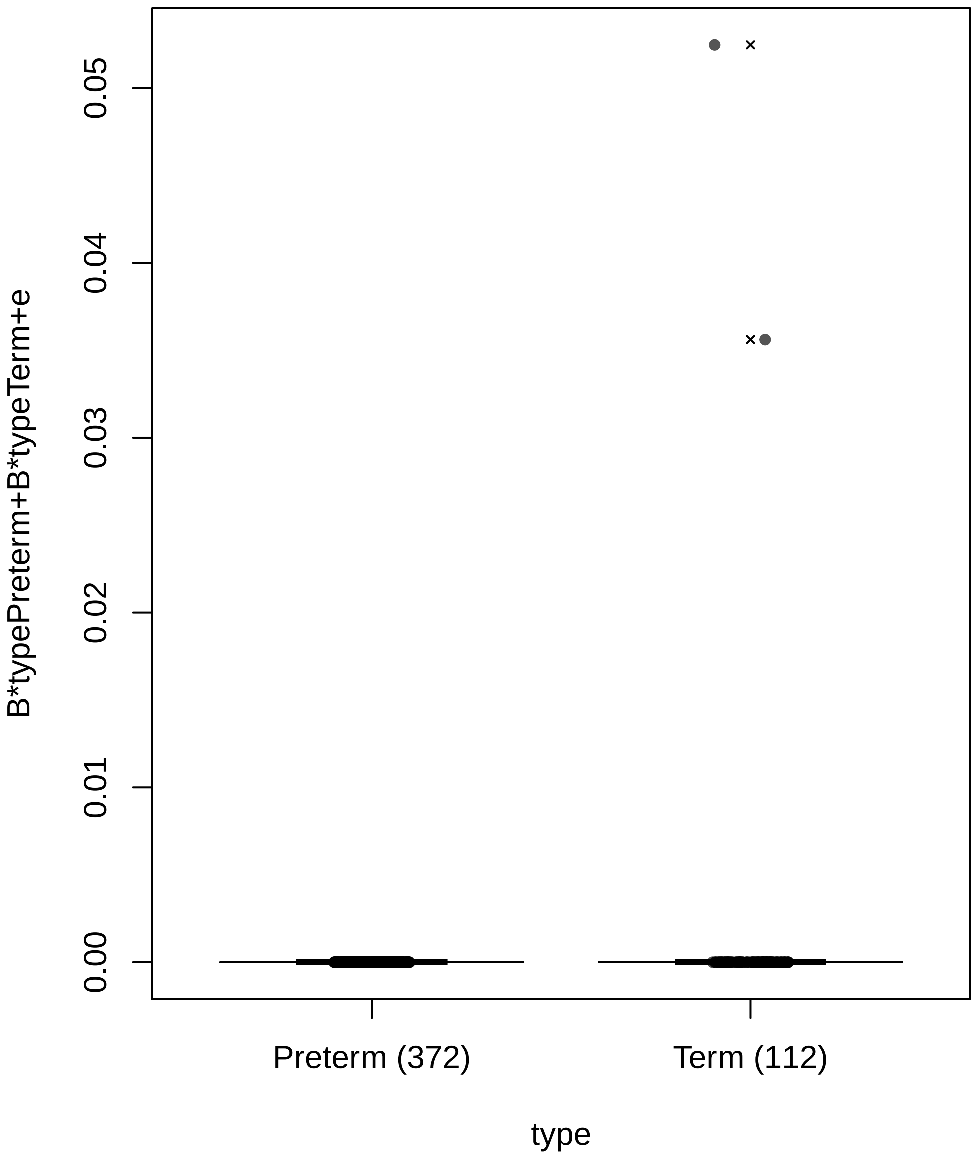

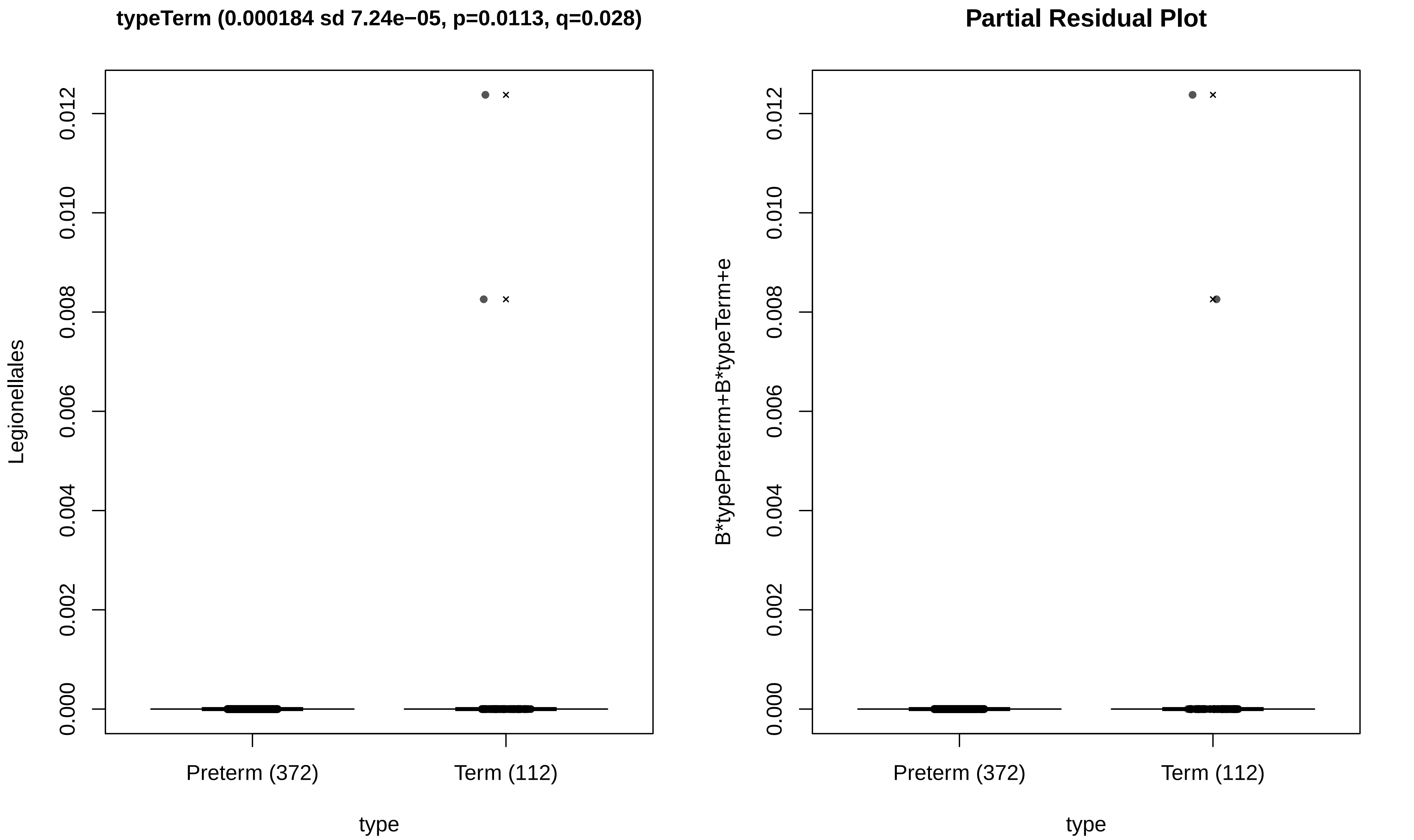
typeTerm (0.000184 sd 7.24e−05, p=0.0113, q=0.028)
Partial Residual Plot
Legionellales
B*typePreterm+B*typeTerm+e
0.000
0.002
0.004
0.006
0.008
0.010
0.012
Preterm (372)
Term (112)
type

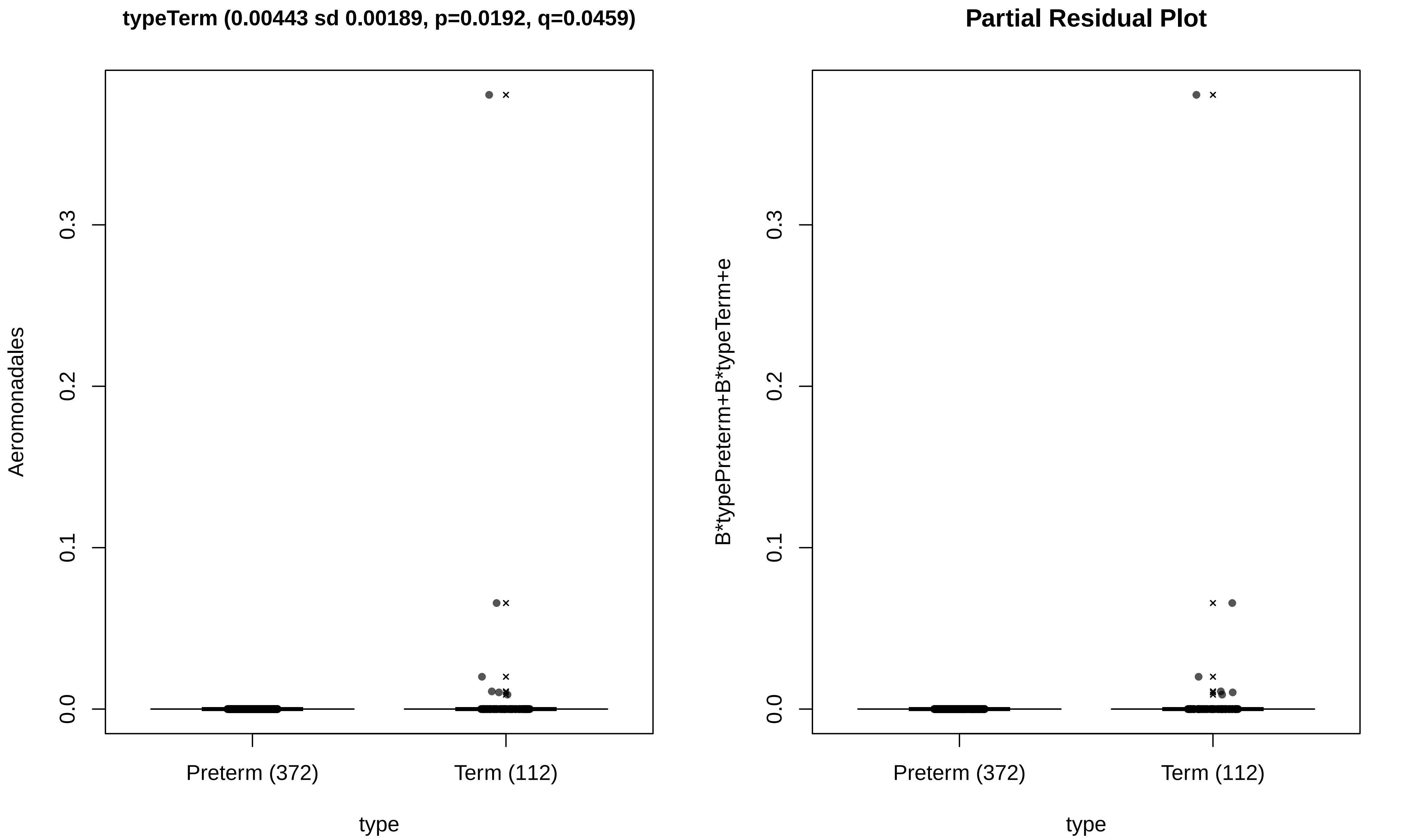

typeTerm (0.00443 sd 0.00189, p=0.0192, q=0.0459)
Aeromonadales
0.0
0.1
0.2
0.3
Preterm (372)
Term (112)
type
Partial Residual Plot
B*typePreterm+B*typeTerm+e
0.0
0.1
0.2
0.3
Preterm (372)
Term (112)
type
