# Supplementary material for: Predicted Metabolic Pathway Distributions in Stool Bacteria in Very-Low-Birth-Weight Infants: Potential Relationships with NICU Faltered Growth
Source: Nutrients. 2020 May 8;12(5):1345. doi: 10.3390/nu12051345 (PMC7284701; doi:10.3390/nu12051345)
Supplement: Supplementary file 1 [file nutrients-12-01345-s001.zip › nutrients-769405-supplementary/Supplementary Meterials/S3_Supplementary_data.pdf]

Supplementary data

Metabolic Pathway Distributions in Very Low Birth Weight Infants: Potential Relationships with NICU Faltered Growth

S3. **Association of the relative abundance of bacterial family between VLBW infants and term infants** . Each page of this file shows a separate bacterial family which was significantly different between the two groups. The box plots indicate the association of the metadata (here, the two groups preterm and term infants) with the relative abundances. The strength of association is indicated by the p-value and an FDR-corrected q-value. The partial residual plot shows the relationship between the groups with abundances when all other variables are included in the model.

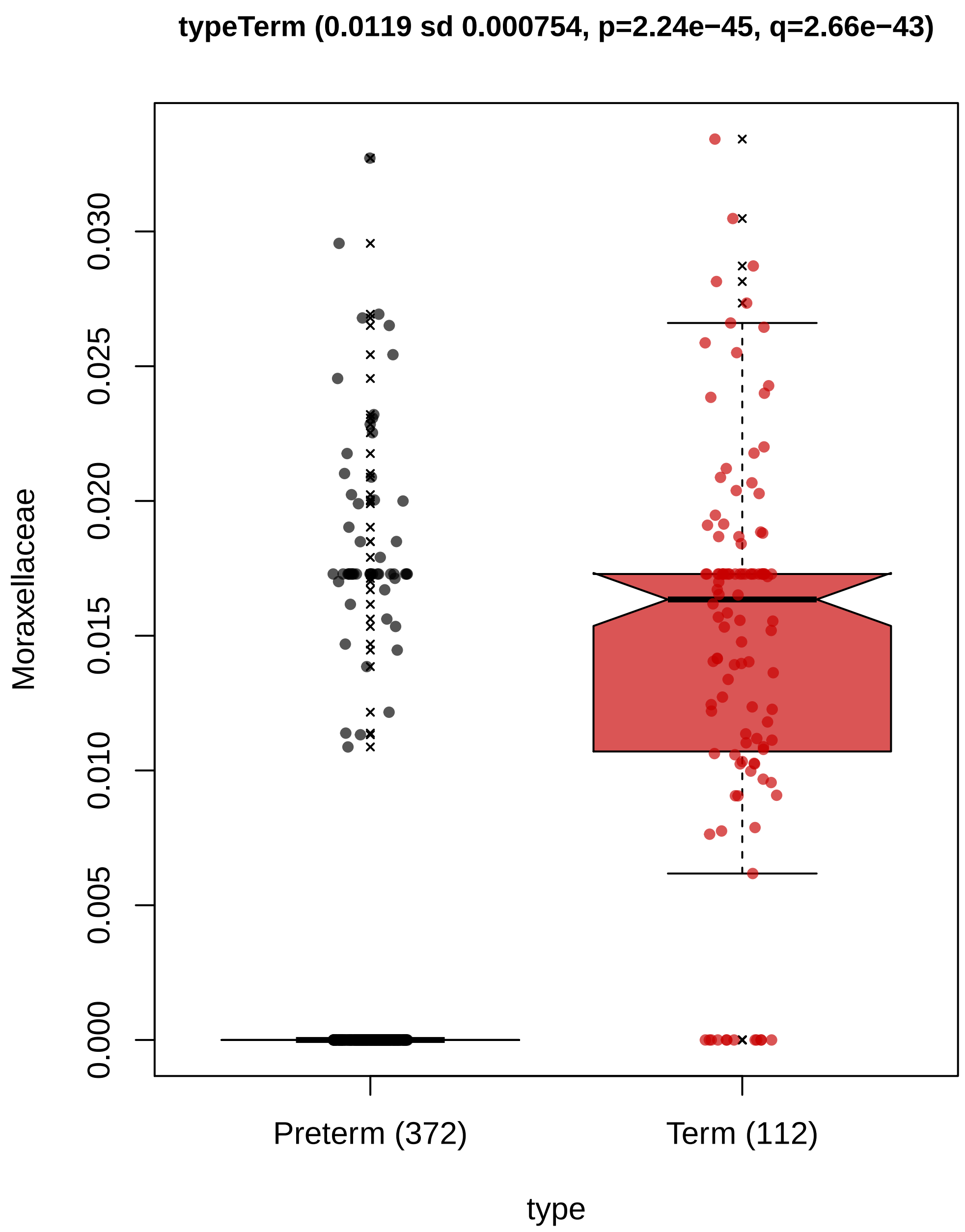
typeTerm (0.0119 sd 0.000754, p=2.24e−45, q=2.66e−43)
Moraxellaceae
0.000
0.005
0.010
0.015
0.020
0.025
0.030
Preterm (372)
Term (112)
type

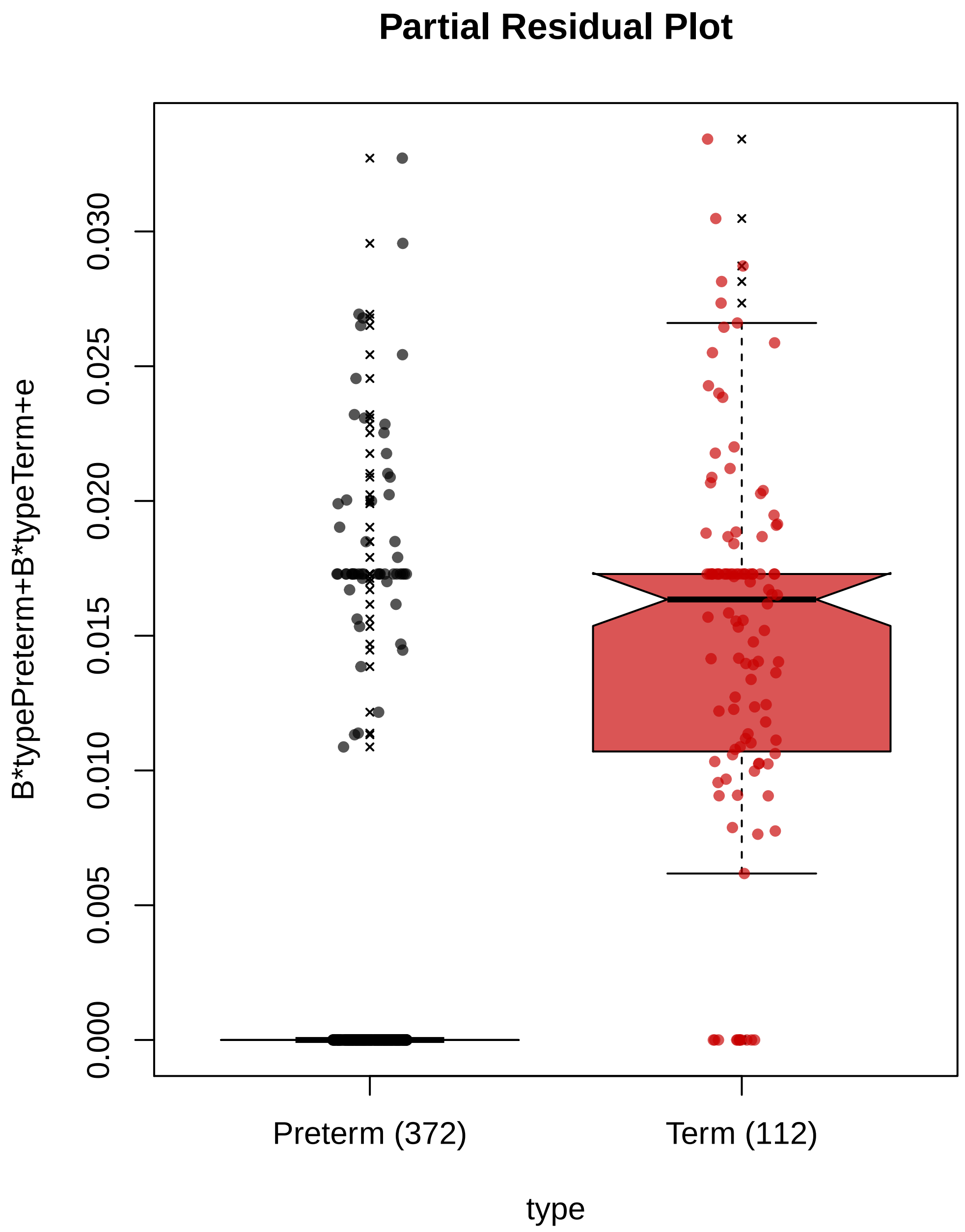
Partial Residual Plot
B*typePreterm+B*typeTerm+e
0.000
0.005
0.010
0.015
0.020
0.025
0.030
Preterm (372)
Term (112)
type

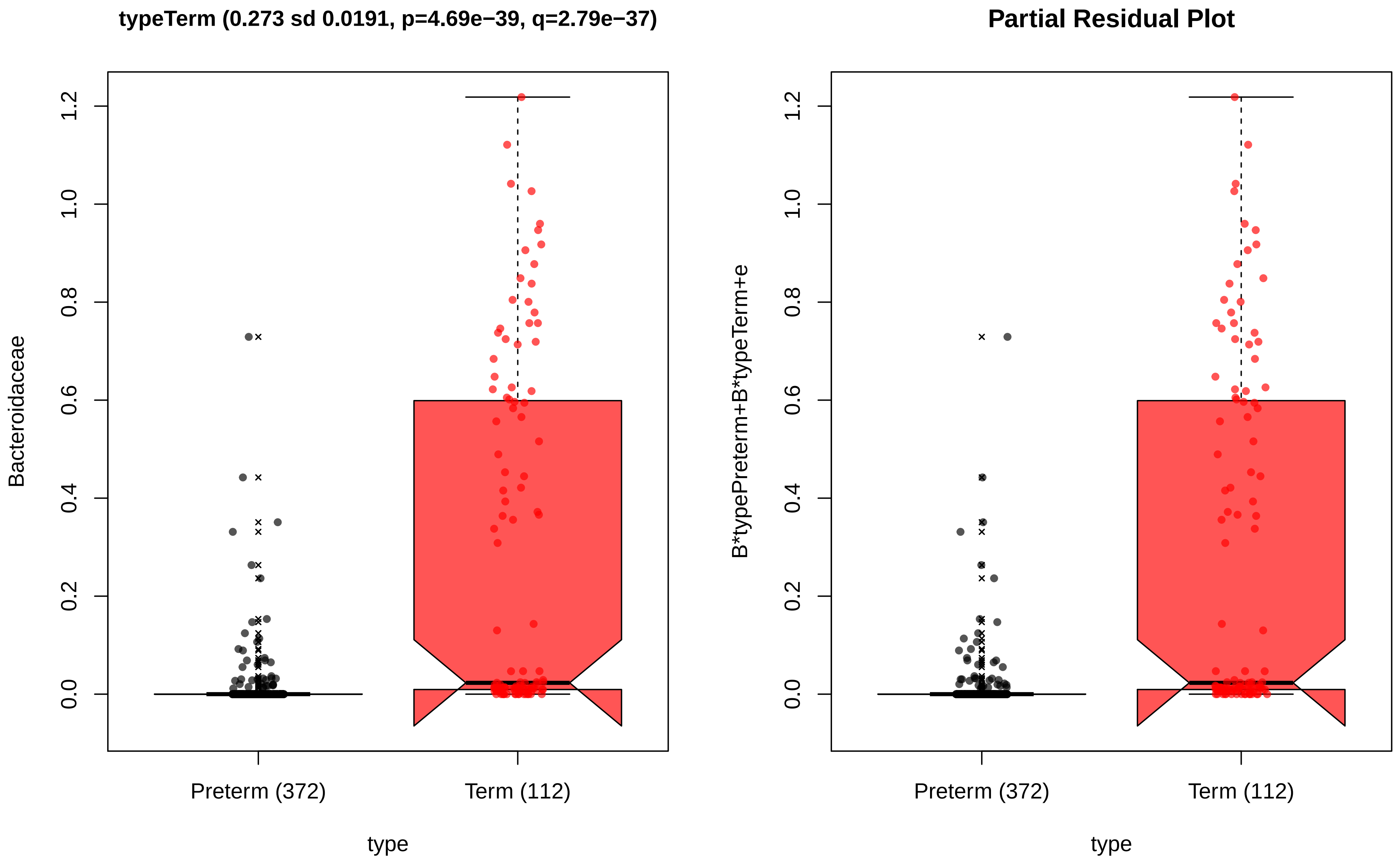

typeTerm (0.273 sd 0.0191, p=4.69e−39, q=2.79e−37)
Partial Residual Plot
Bacteroidaceae
B*typePreterm+B*typeTerm+e
0.0
0.2
0.4
0.6
0.8
1.0
1.2
Preterm (372)
Term (112)
type

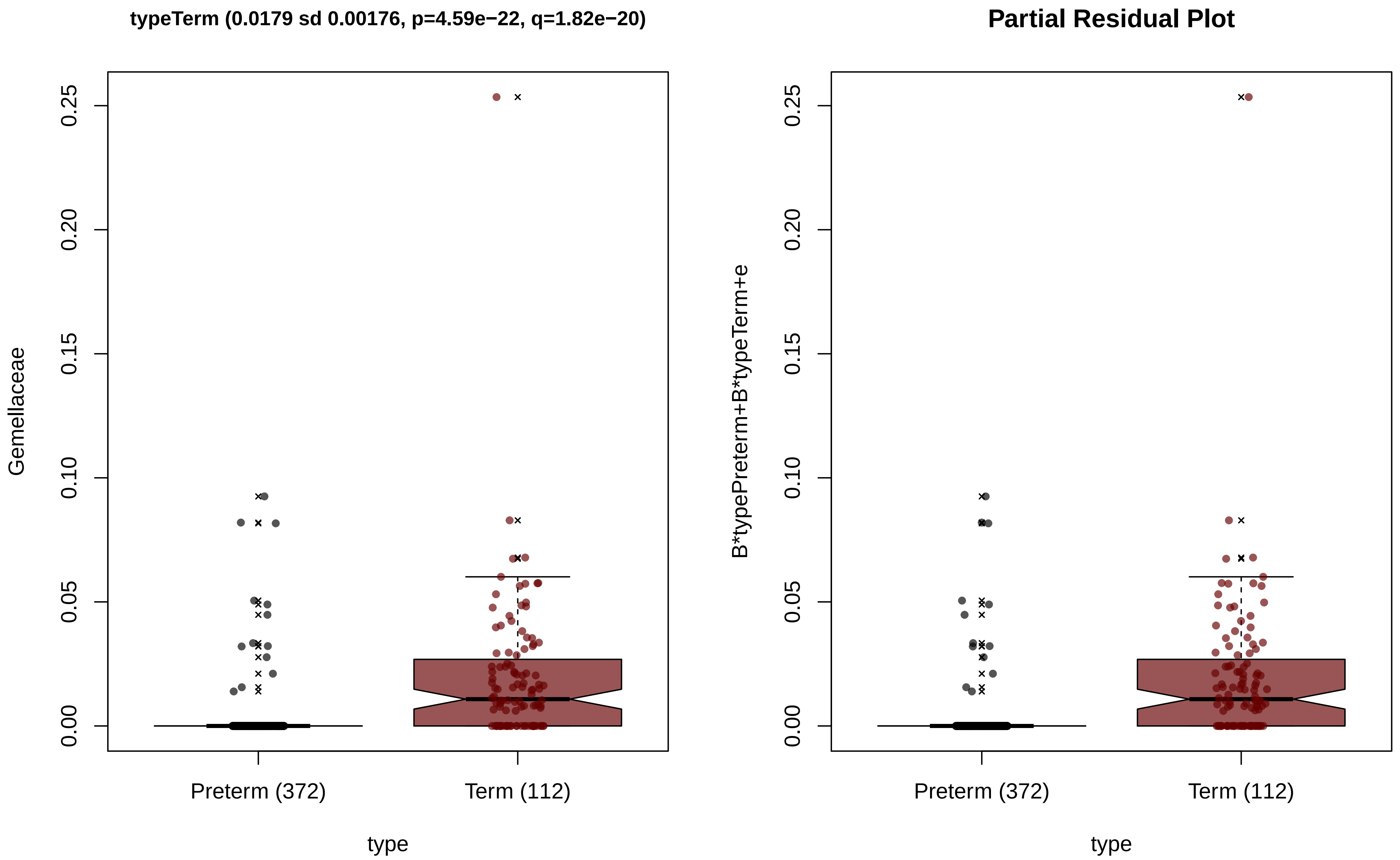

typeTerm (0.0179 sd 0.00176, p=4.59e−22, q=1.82e−20)
Gemellaceae
0.00
0.05
0.10
0.15
0.20
0.25
Preterm (372)
Term (112)
type
Partial Residual Plot
B*typePreterm+B*typeTerm+e
0.00
0.05
0.10
0.15
0.20
0.25
Preterm (372)
Term (112)
type

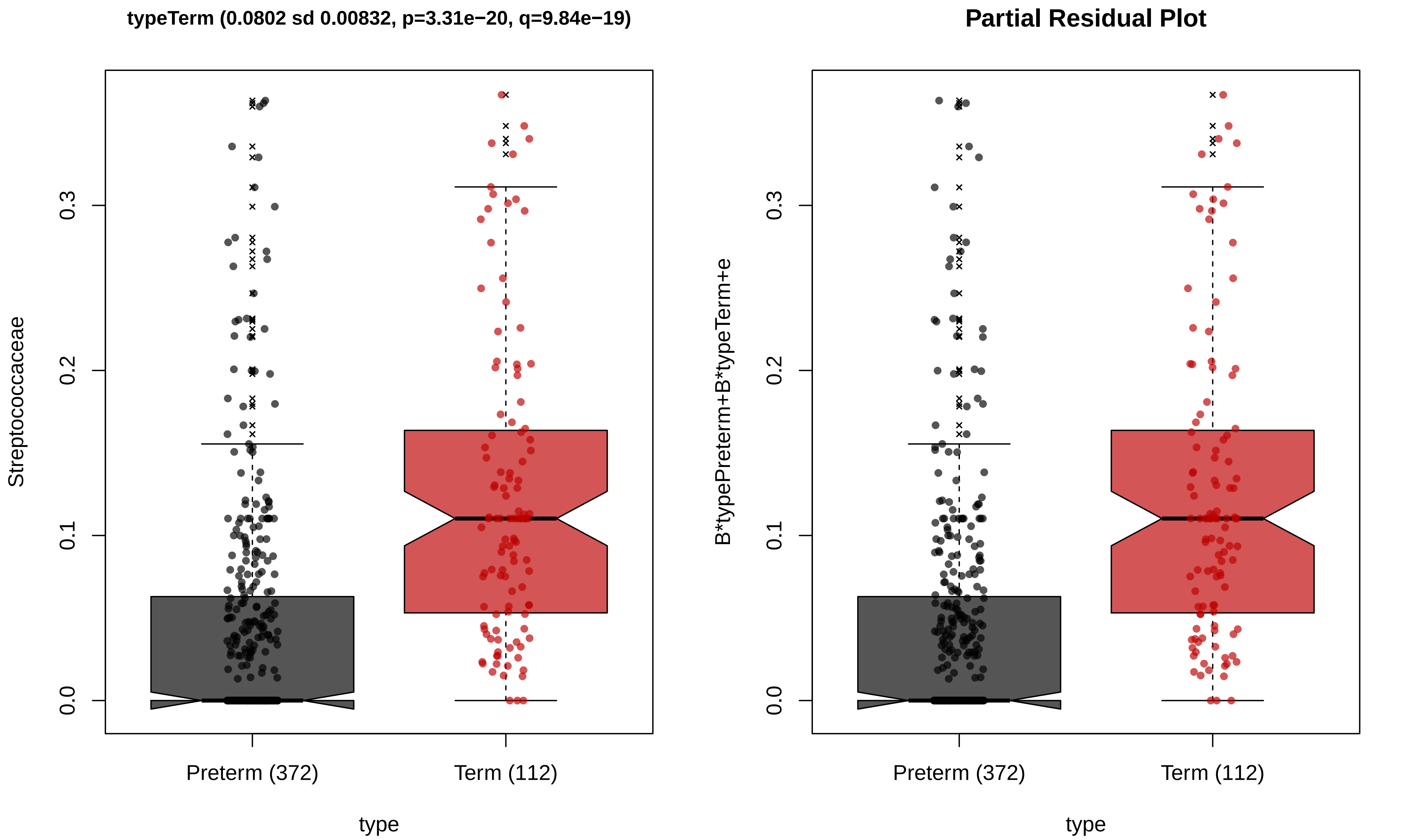
typeTerm (0.0802 sd 0.00832, p=3.31e−20, q=9.84e−19)
Partial Residual Plot
Streptococcaceae
B*typePreterm+B*typeTerm+e
0.0
0.1
0.2
0.3
Preterm (372)
Term (112)
type

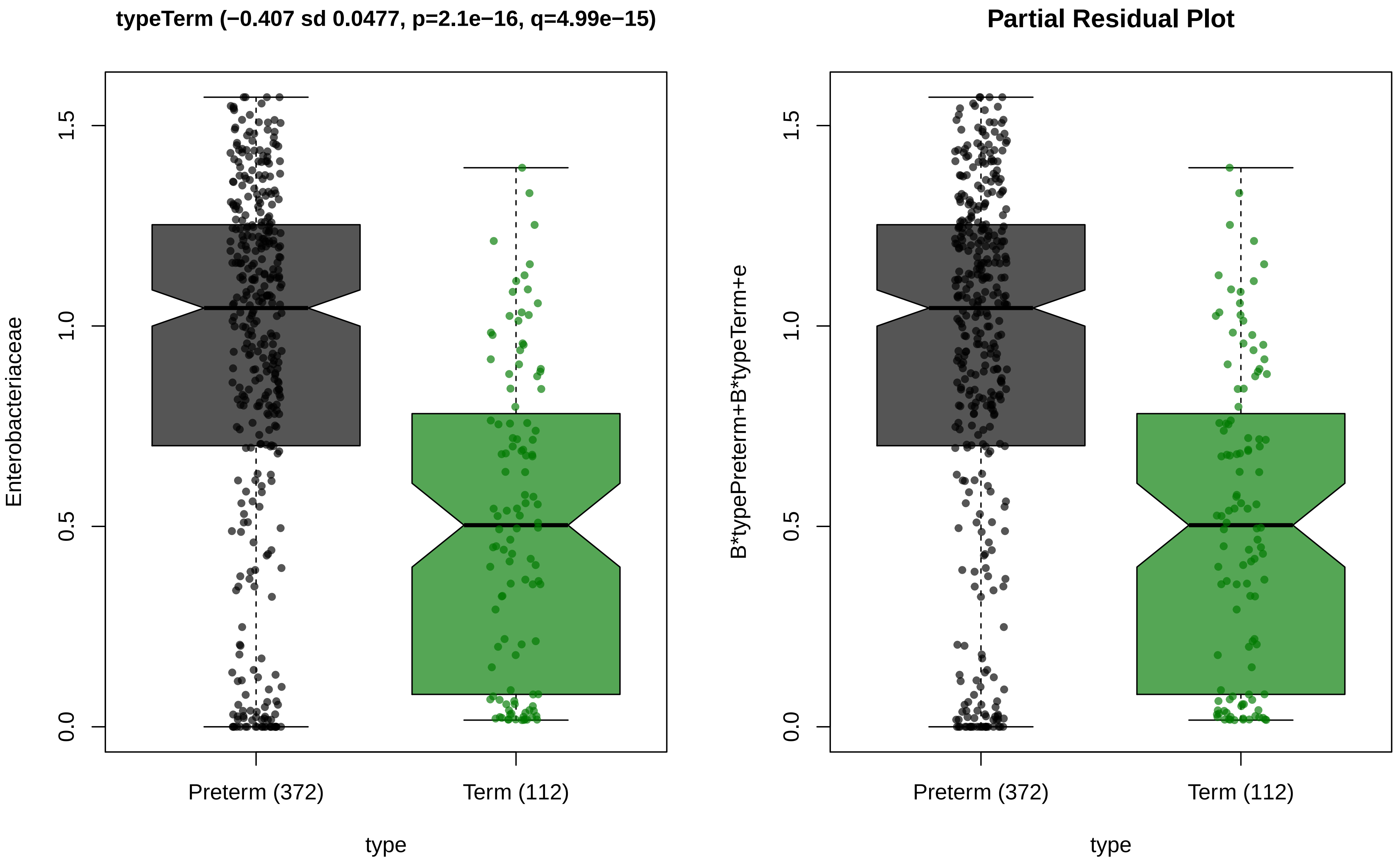

typeTerm (−0.407 sd 0.0477, p=2.1e−16, q=4.99e−15)
Enterobacteriaceae
0.0
0.5
1.0
1.5
Preterm (372)
Term (112)
type
Partial Residual Plot
B*typePreterm+B*typeTerm+e
0.0
0.5
1.0
1.5
Preterm (372)
Term (112)
type

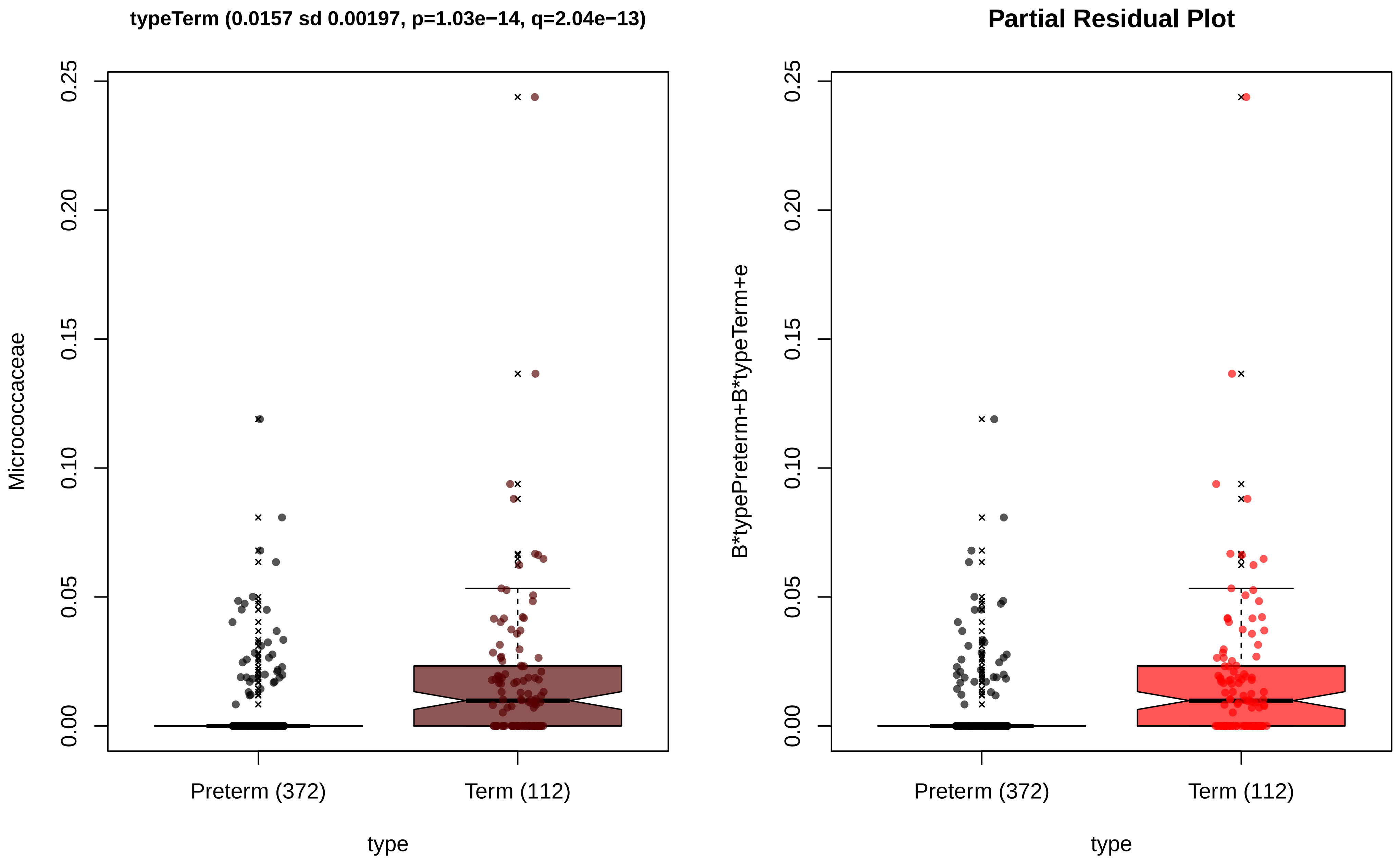
typeTerm (0.0157 sd 0.00197, p=1.03e−14, q=2.04e−13)
Micrococcaceae
0.00
0.05
0.10
0.15
0.20
0.25
Preterm (372)
Term (112)
type
Partial Residual Plot
B*typePreterm+B*typeTerm+e
0.00
0.05
0.10
0.15
0.20
0.25
Preterm (372)
Term (112)
type

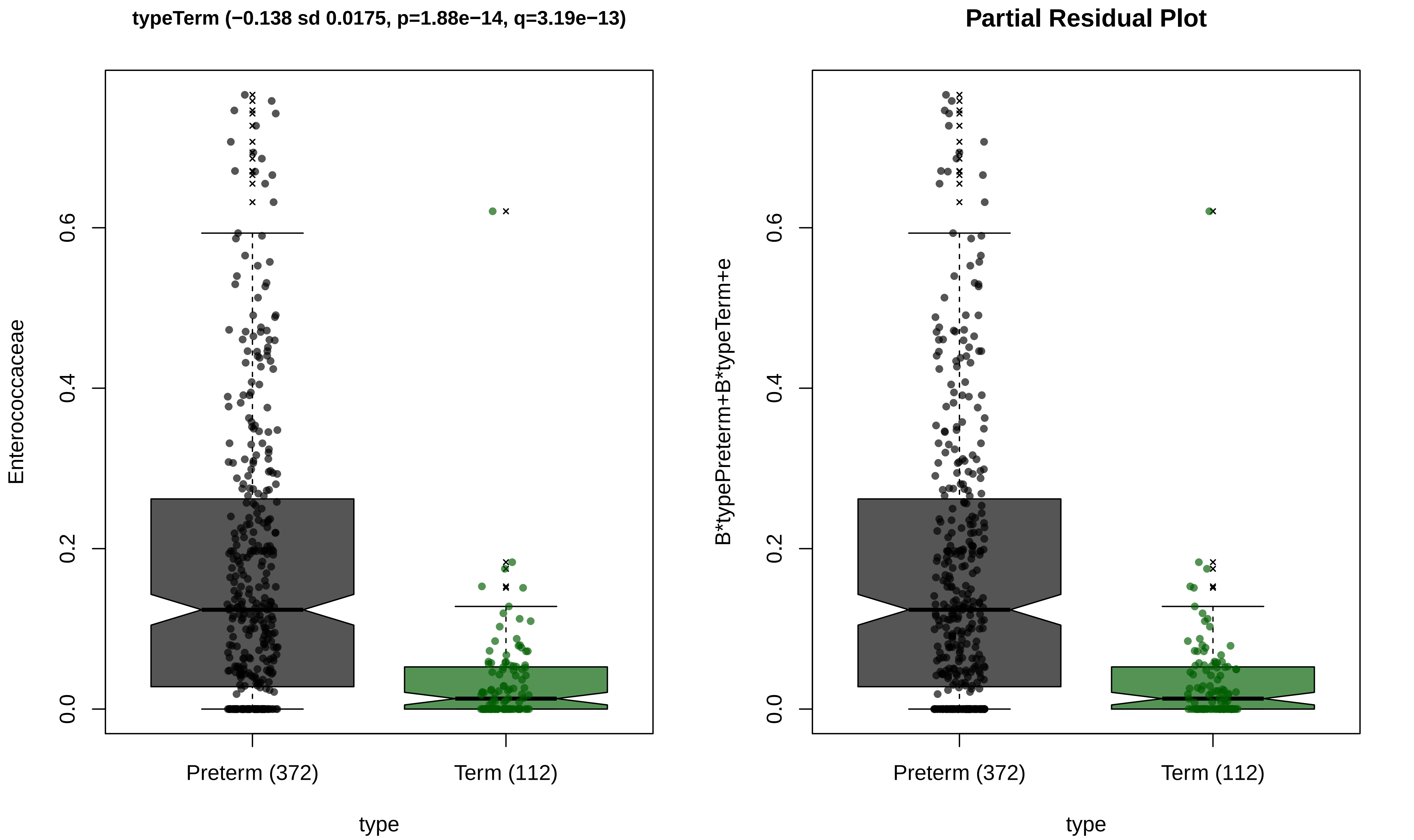
typeTerm (−0.138 sd 0.0175, p=1.88e−14, q=3.19e−13)
Enterococcaceae
0.0
0.2
0.4
0.6
Preterm (372)
Term (112)
type
Partial Residual Plot
B*typePreterm+B*typeTerm+e
0.0
0.2
0.4
0.6
Preterm (372)
Term (112)
type

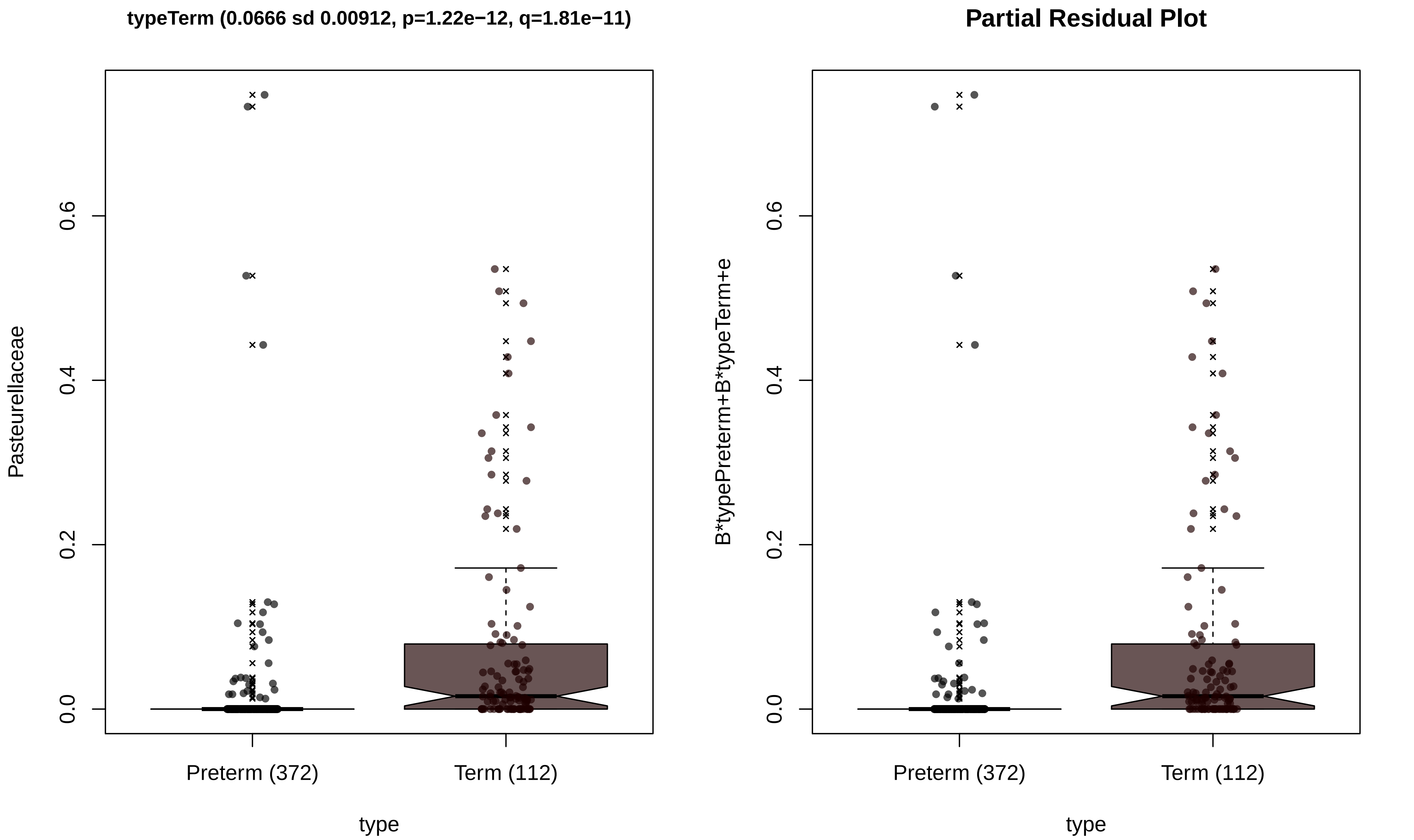
typeTerm (0.0666 sd 0.00912, p=1.22e−12, q=1.81e−11)
Partial Residual Plot
Pasteurellaceae
B*typePreterm+B*typeTerm+e
0.0
0.2
0.4
0.6
Preterm (372)
Term (112)
type

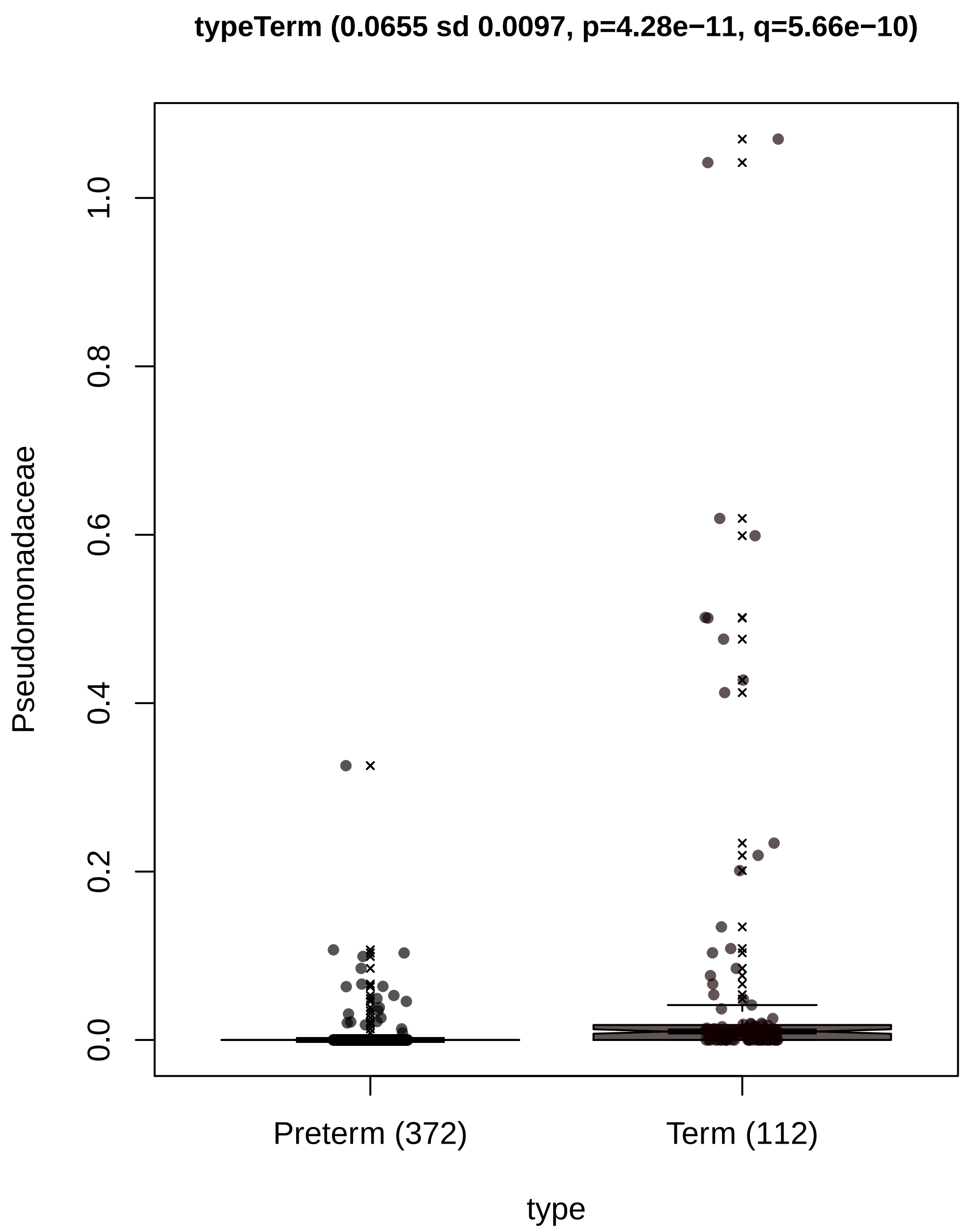

typeTerm (0.0655 sd 0.0097, p=4.28e−11, q=5.66e−10)
Pseudomonadaceae
0.0
0.2
0.4
0.6
0.8
1.0
Preterm (372)
Term (112)
type

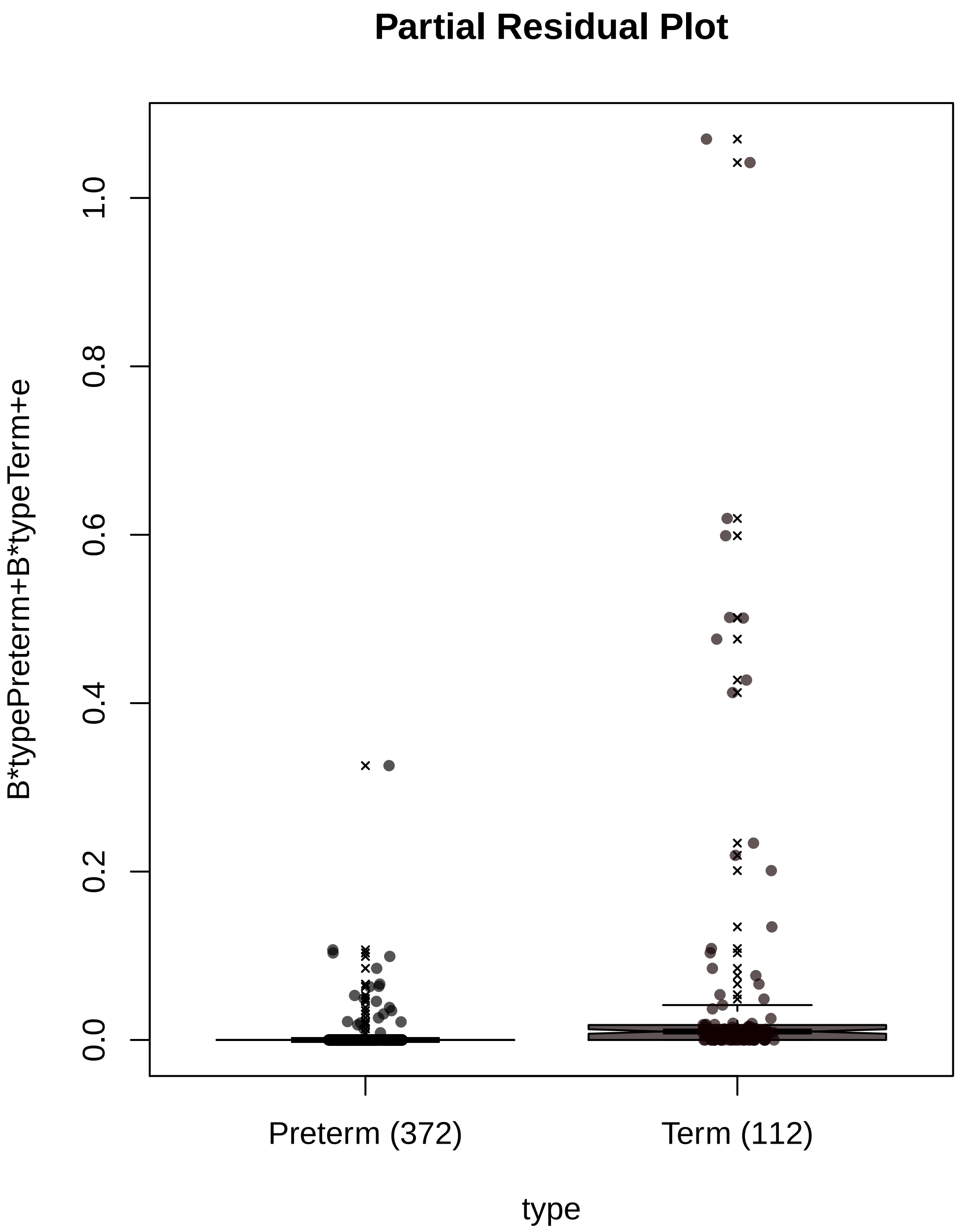

Partial Residual Plot
B*typePreterm+B*typeTerm+e
0.0
0.2
0.4
0.6
0.8
1.0
Preterm (372)
Term (112)
type

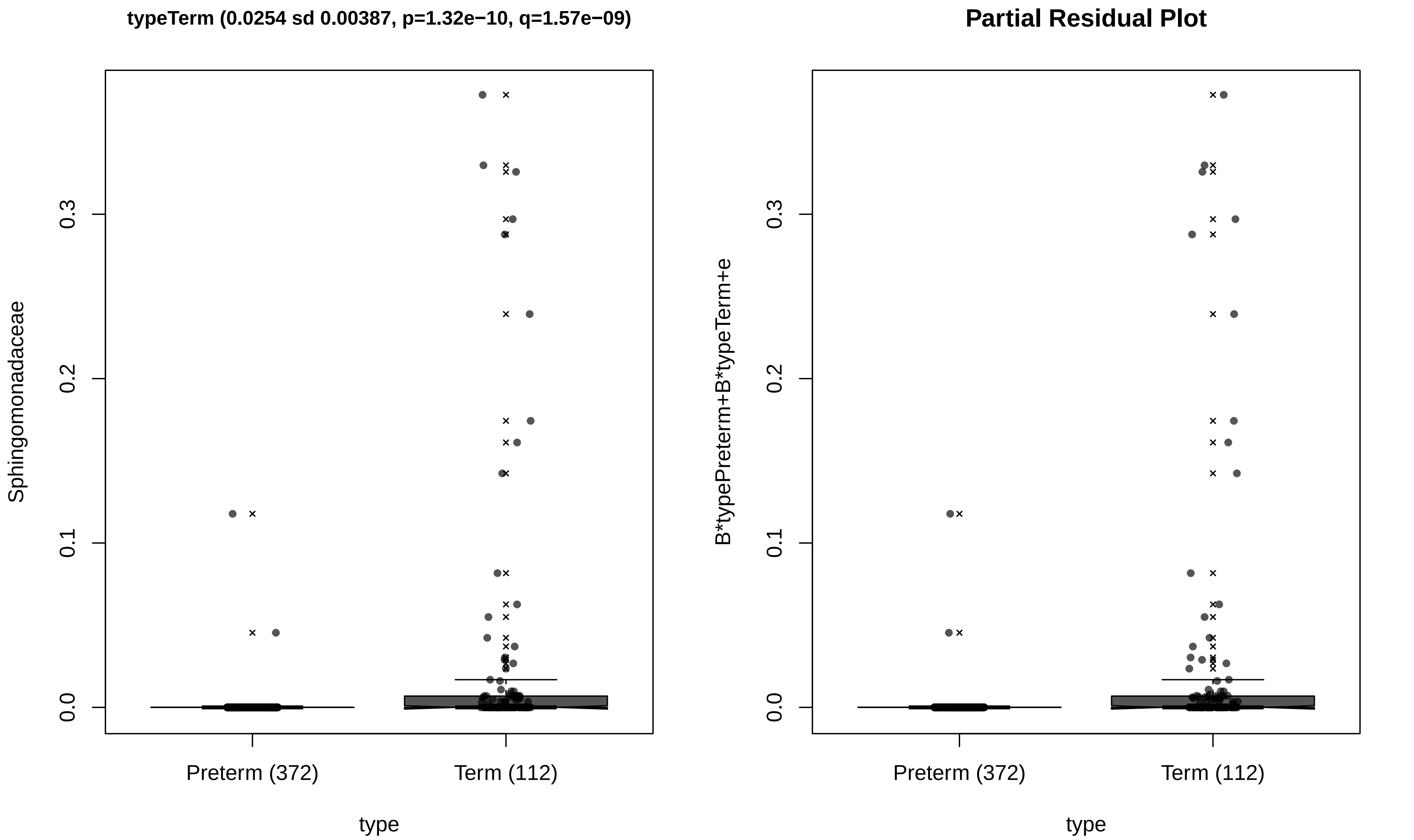

typeTerm (0.0254 sd 0.00387, p=1.32e−10, q=1.57e−09)
Partial Residual Plot
Sphingomonadaceae
B*typePreterm+B*typeTerm+e
0.0
0.1
0.2
0.3
Preterm (372)
Term (112)
type

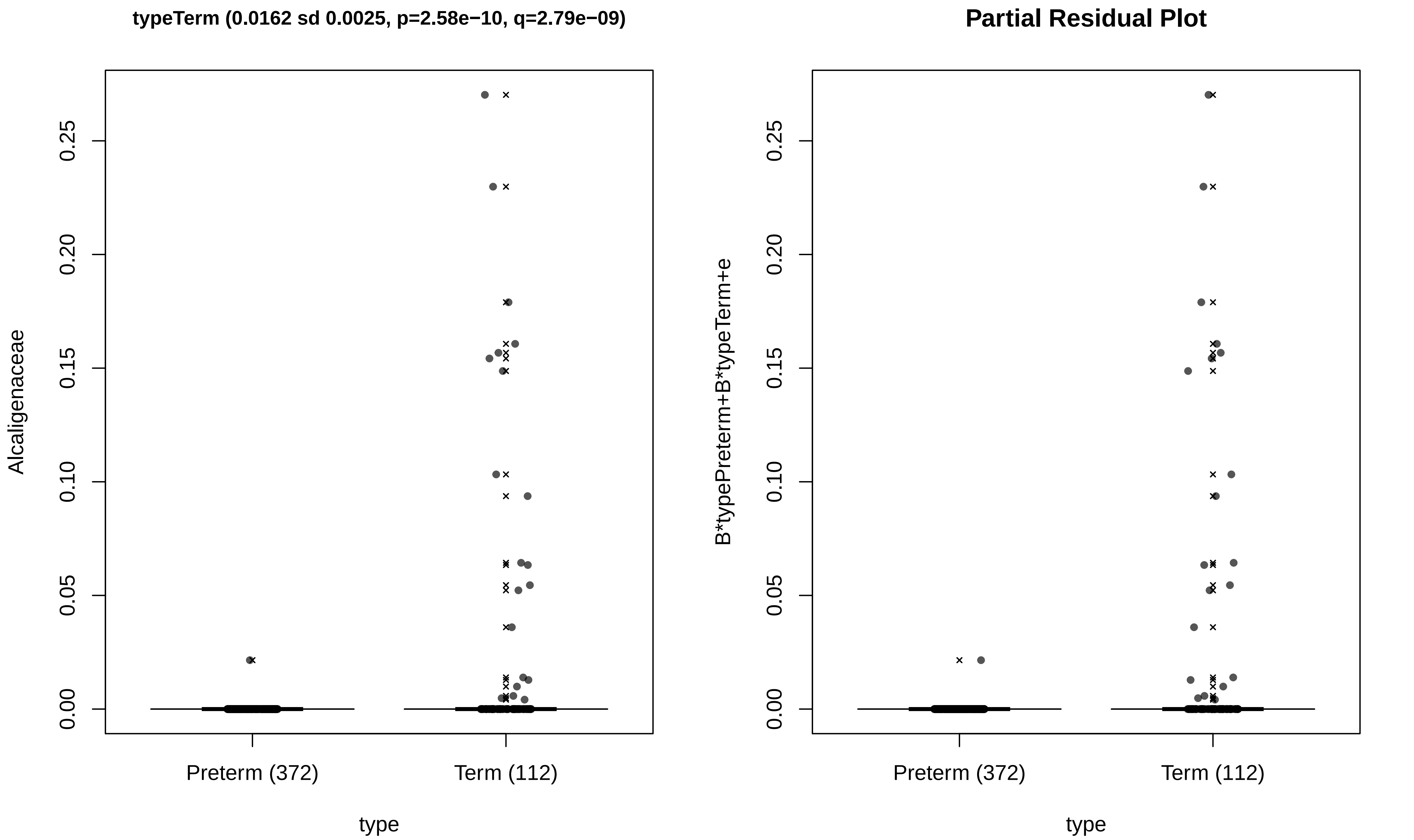
typeTerm (0.0162 sd 0.0025, p=2.58e−10, q=2.79e−09)
Alcaligenaceae
0.00
0.05
0.10
0.15
0.20
0.25
Preterm (372)
Term (112)
type
Partial Residual Plot
B*typePreterm+B*typeTerm+e
0.00
0.05
0.10
0.15
0.20
0.25
Preterm (372)
Term (112)
type

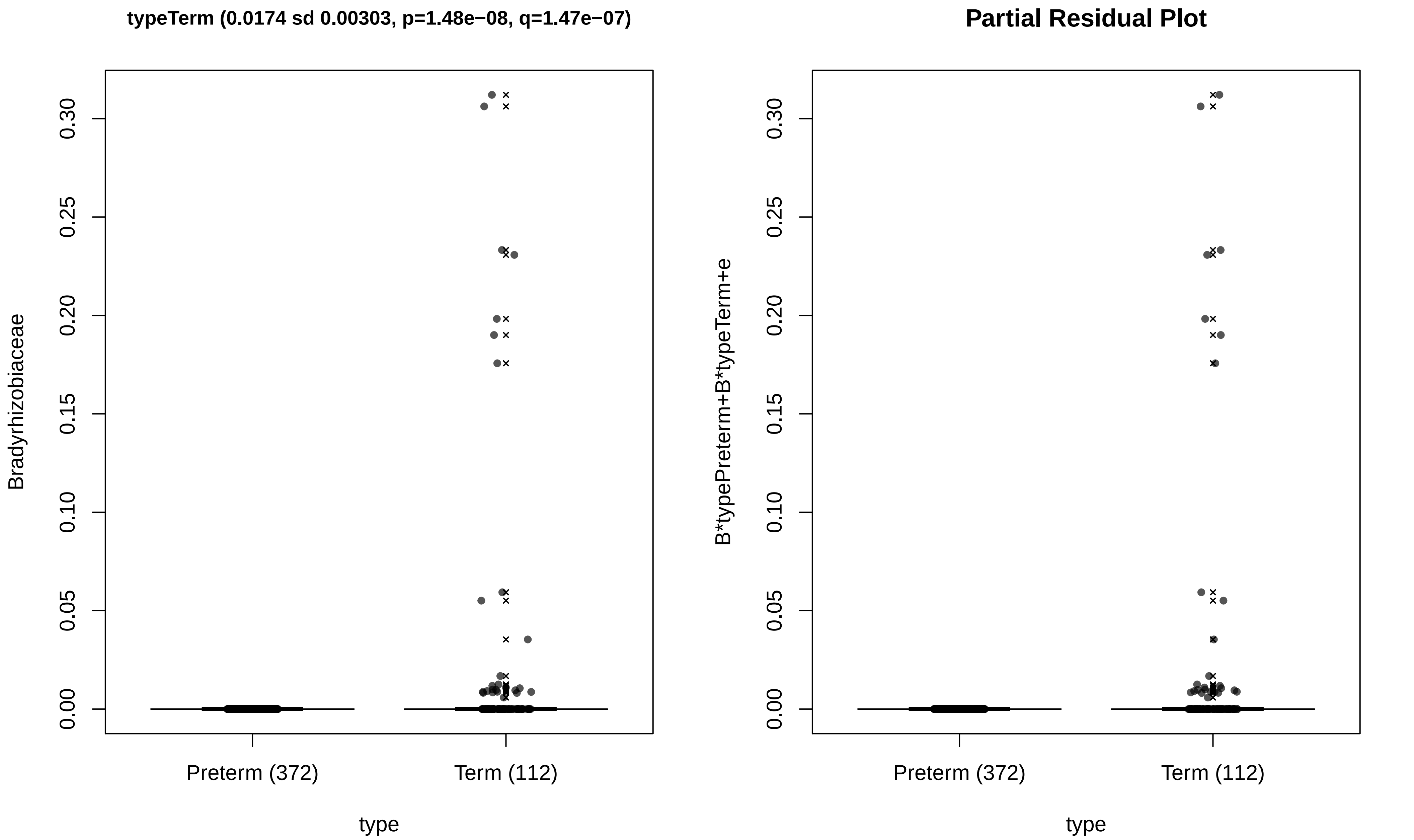
typeTerm (0.0174 sd 0.00303, p=1.48e−08, q=1.47e−07)
Partial Residual Plot
Bradyrhizobiaceae
B*typePreterm+B*typeTerm+e
0.00
0.05
0.10
0.15
0.20
0.25
0.30
Preterm (372)
Term (112)
type

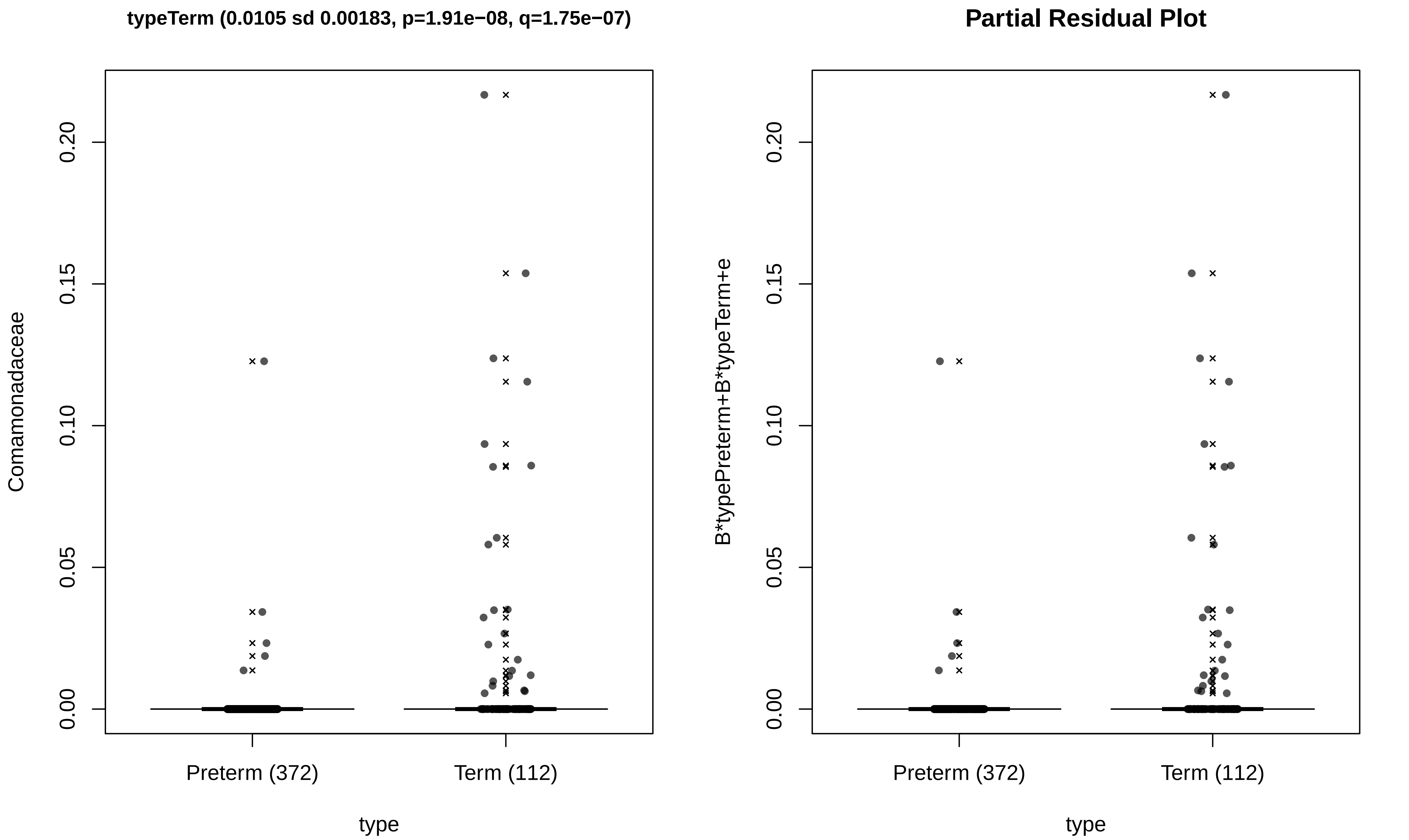

typeTerm (0.0105 sd 0.00183, p=1.91e−08, q=1.75e−07)
Comamonadaceae
0.00
0.05
0.10
0.15
0.20
Preterm (372)
Term (112)
type
Partial Residual Plot
B*typePreterm+B*typeTerm+e
0.00
0.05
0.10
0.15
0.20
Preterm (372)
Term (112)
type

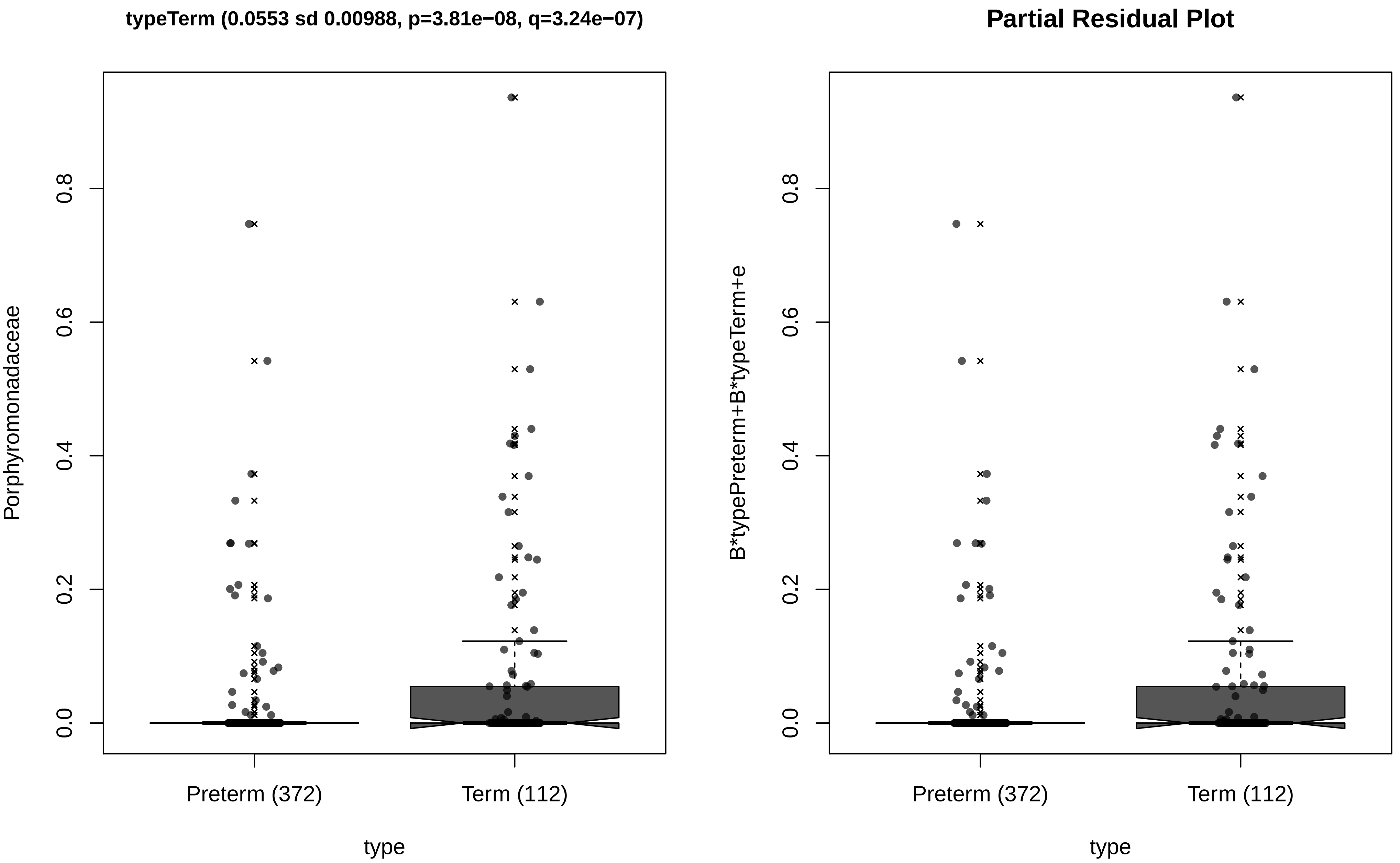

typeTerm (0.0553 sd 0.00988, p=3.81e−08, q=3.24e−07)
Porphyromonadaceae
0.0
0.2
0.4
0.6
0.8
Preterm (372)
Term (112)
type
Partial Residual Plot
B*typePreterm+B*typeTerm+e
0.0
0.2
0.4
0.6
0.8
Preterm (372)
Term (112)
type

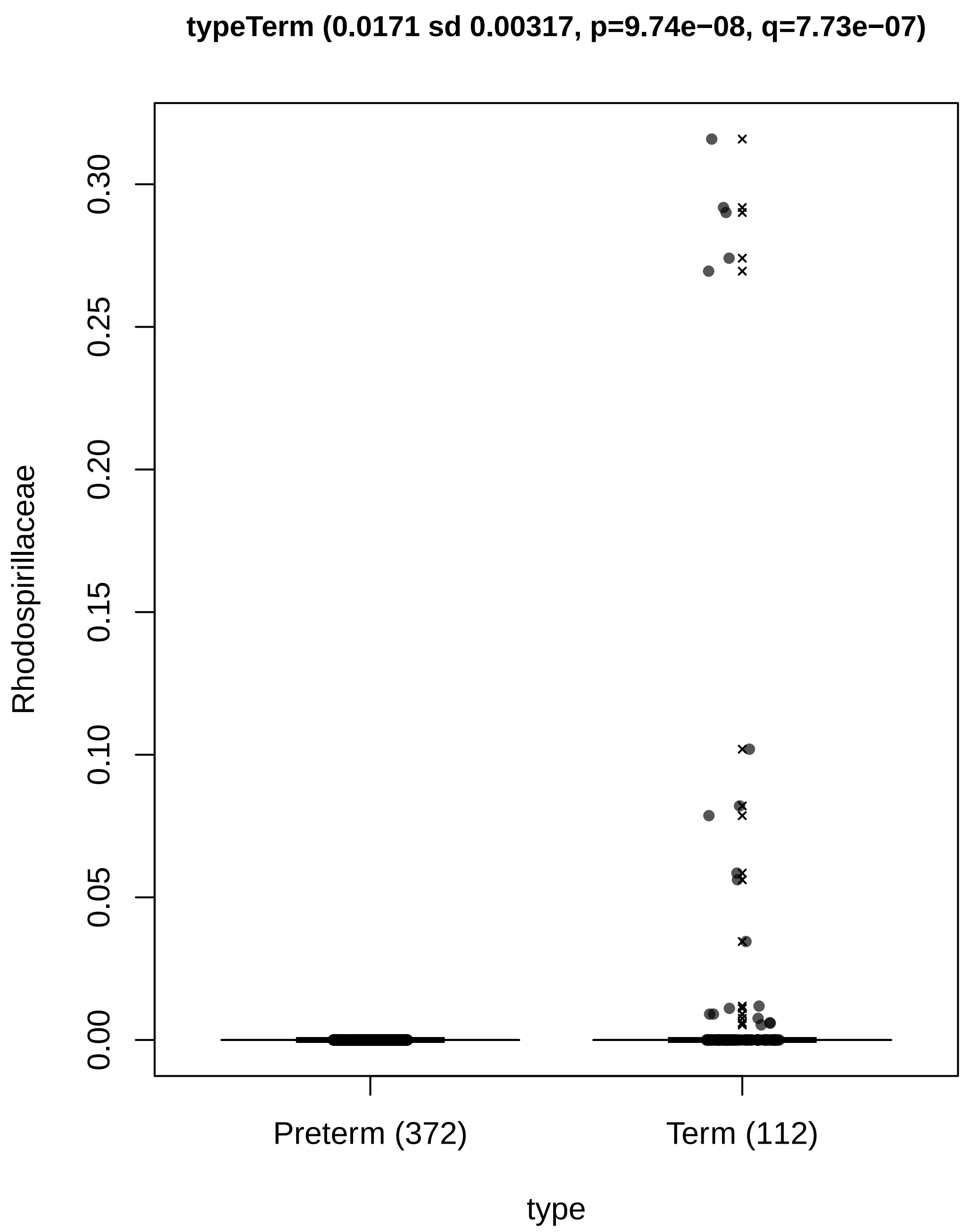

typeTerm (0.0171 sd 0.00317, p=9.74e−08, q=7.73e−07)
Rhodospirillaceae
0.00
0.05
0.10
0.15
0.20
0.25
0.30
Preterm (372)
Term (112)
type

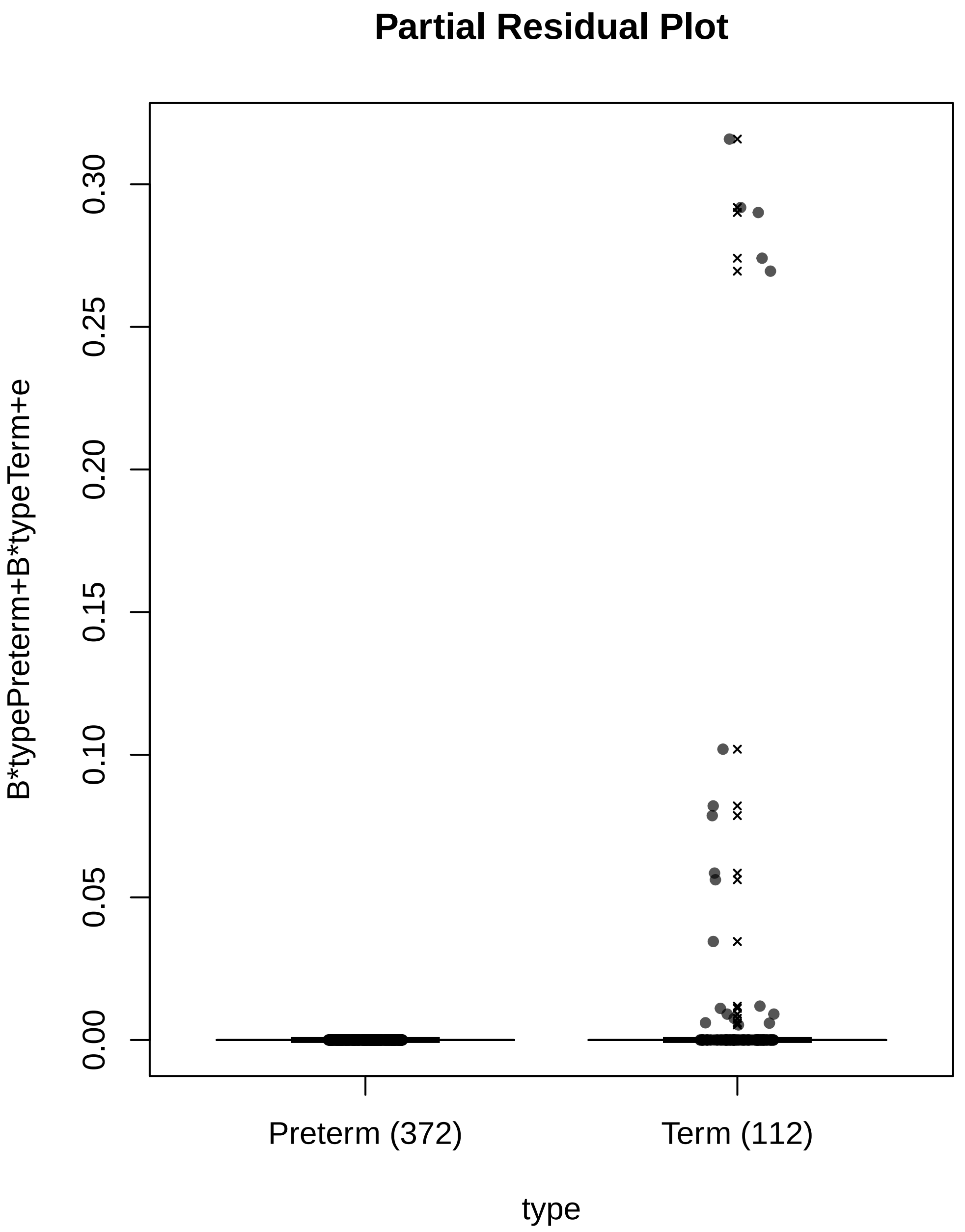

Partial Residual Plot
B*typePreterm+B*typeTerm+e
0.00
0.05
0.10
0.15
0.20
0.25
0.30
Preterm (372)
Term (112)
type

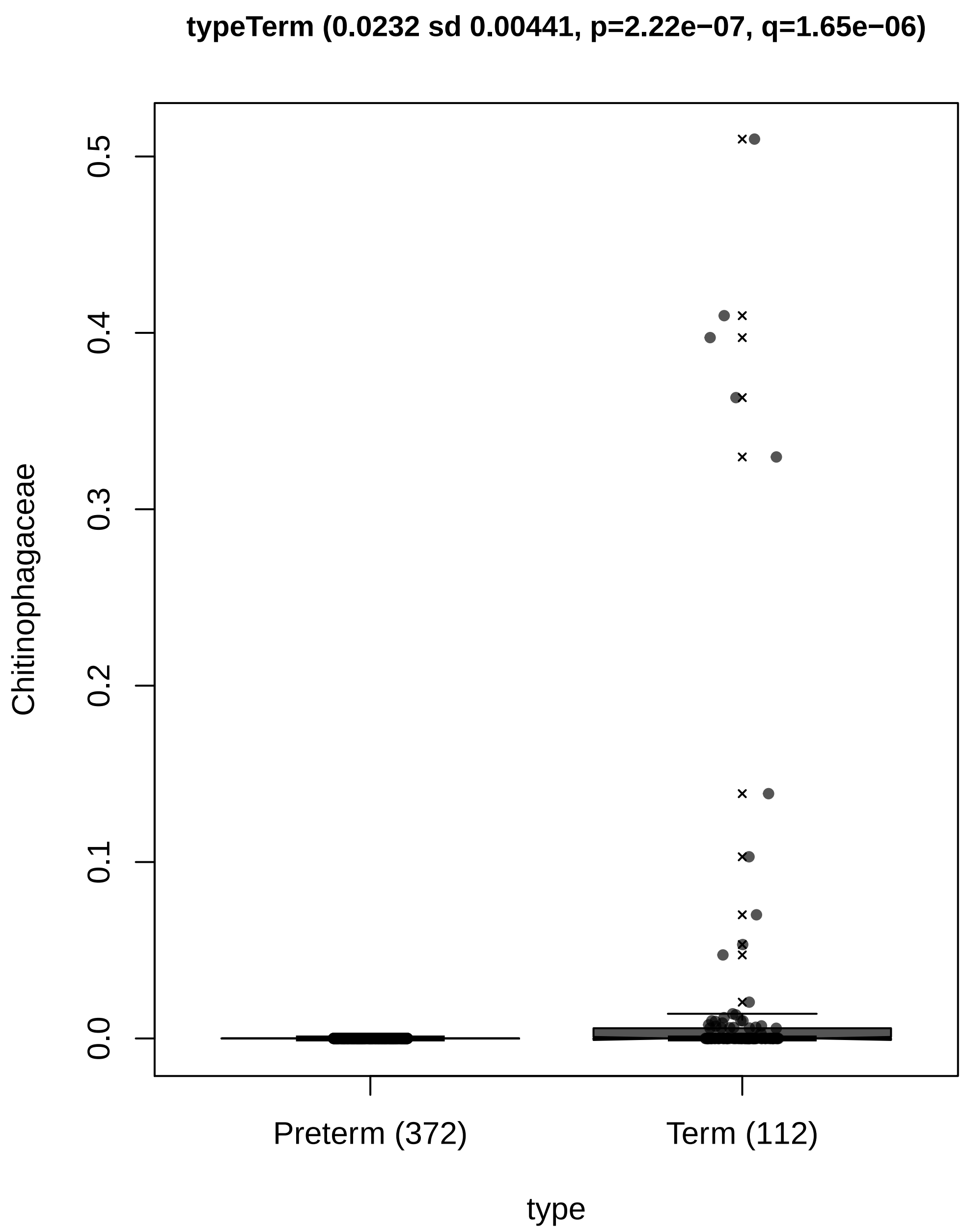

typeTerm (0.0232 sd 0.00441, p=2.22e−07, q=1.65e−06)
Chitinophagaceae
0.0
0.1
0.2
0.3
0.4
0.5
Preterm (372)
Term (112)
type

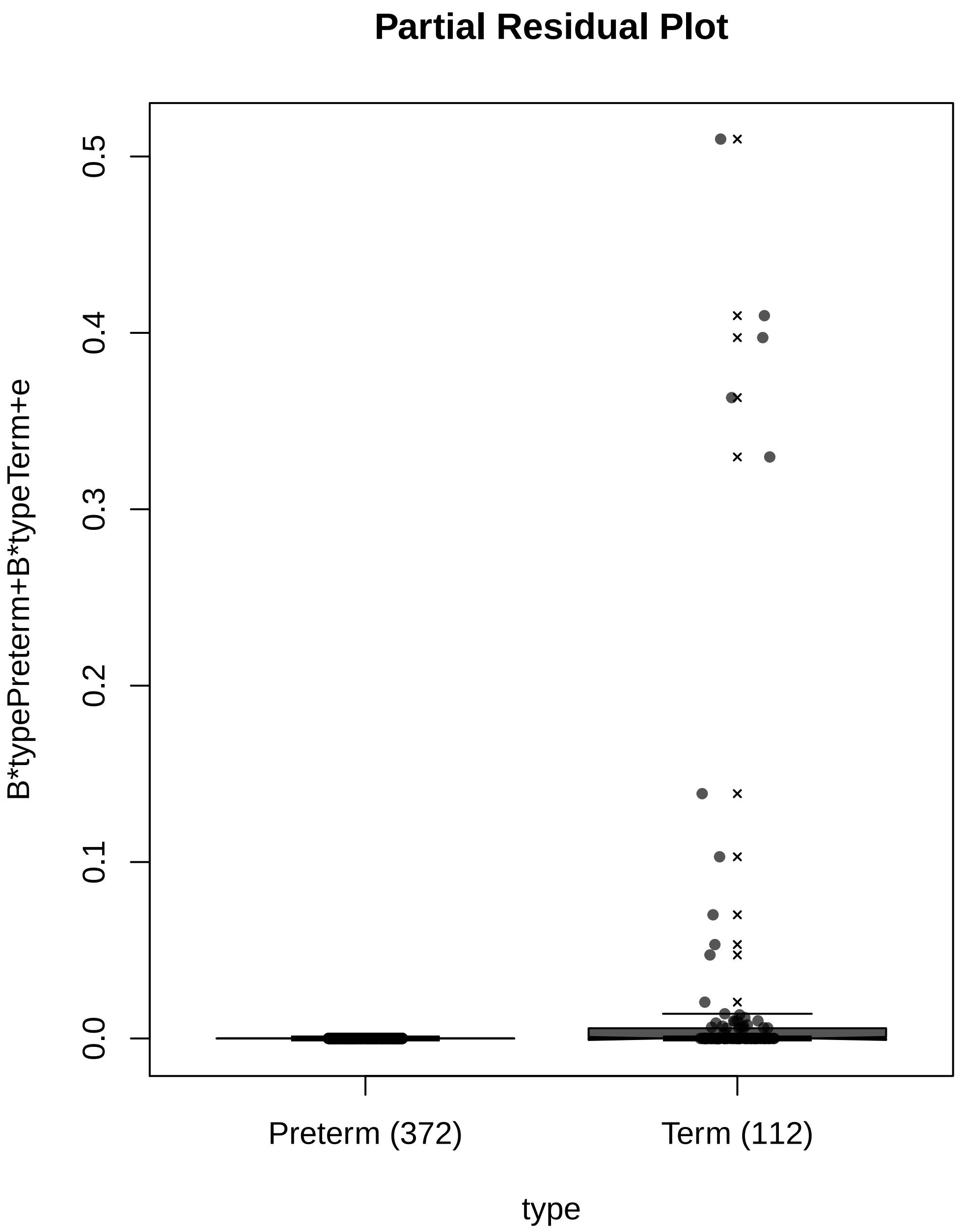

Partial Residual Plot
B*typePreterm+B*typeTerm+e
0.0
0.1
0.2
0.3
0.4
0.5
Preterm (372)
Term (112)
type

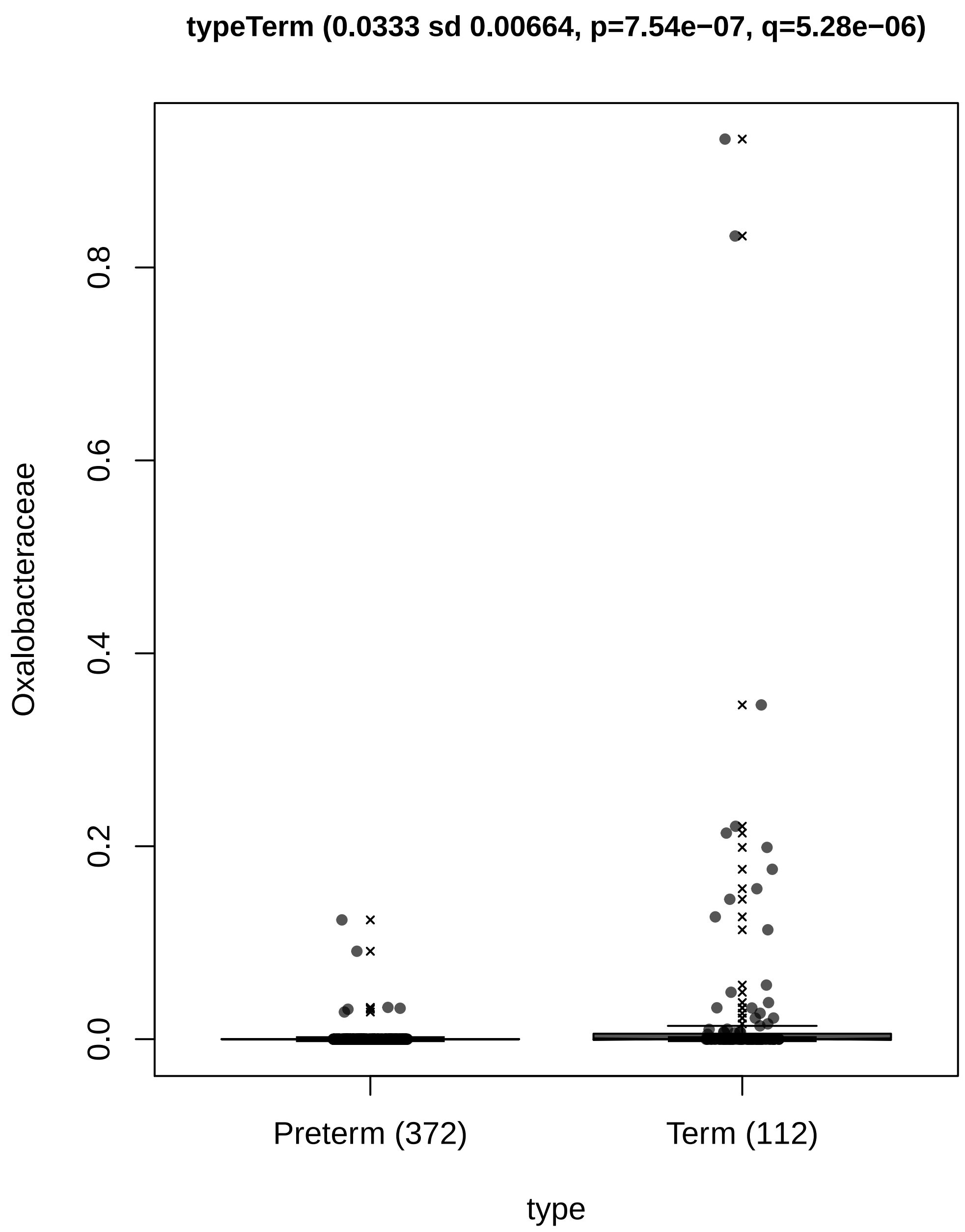
typeTerm (0.0333 sd 0.00664, p=7.54e−07, q=5.28e−06)
Oxalobacteraceae
0.0
0.2
0.4
0.6
0.8
Preterm (372)
Term (112)
type

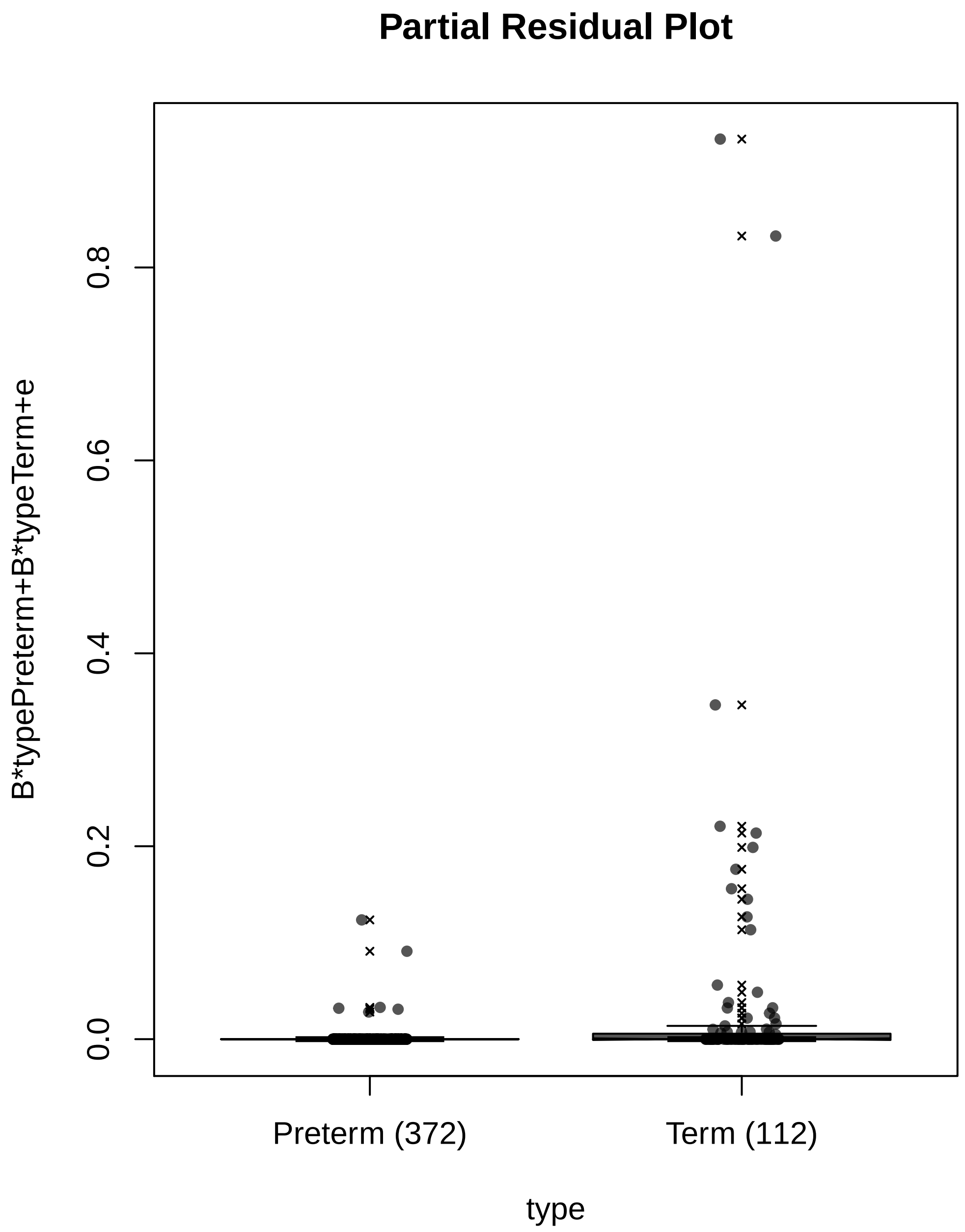
Partial Residual Plot
B*typePreterm+B*typeTerm+e
0.0
0.2
0.4
0.6
0.8
Preterm (372)
Term (112)
type

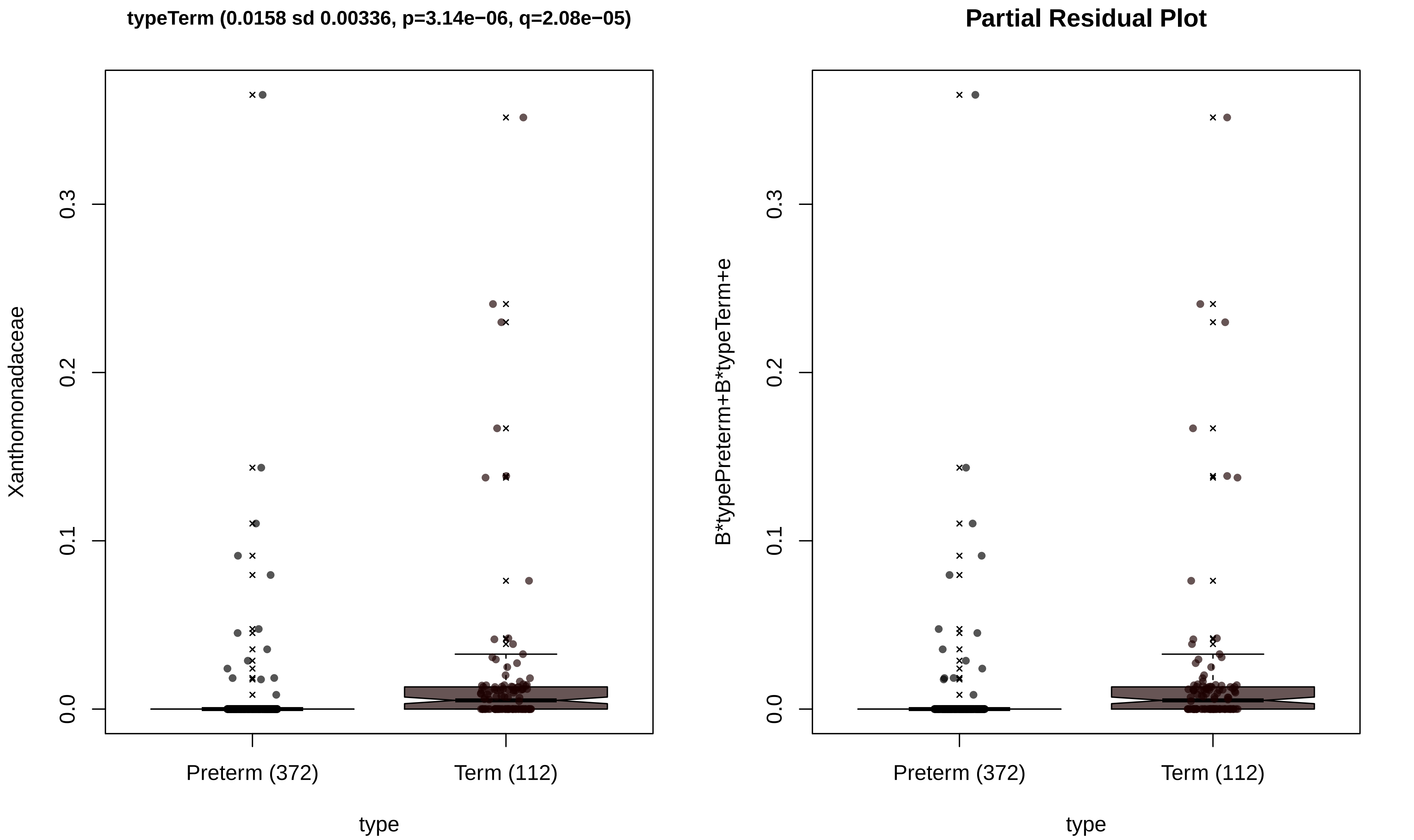

typeTerm (0.0158 sd 0.00336, p=3.14e−06, q=2.08e−05)
Xanthomonadaceae
0.0
0.1
0.2
0.3
Preterm (372)
Term (112)
type
Partial Residual Plot
B*typePreterm+B*typeTerm+e
0.0
0.1
0.2
0.3
Preterm (372)
Term (112)
type

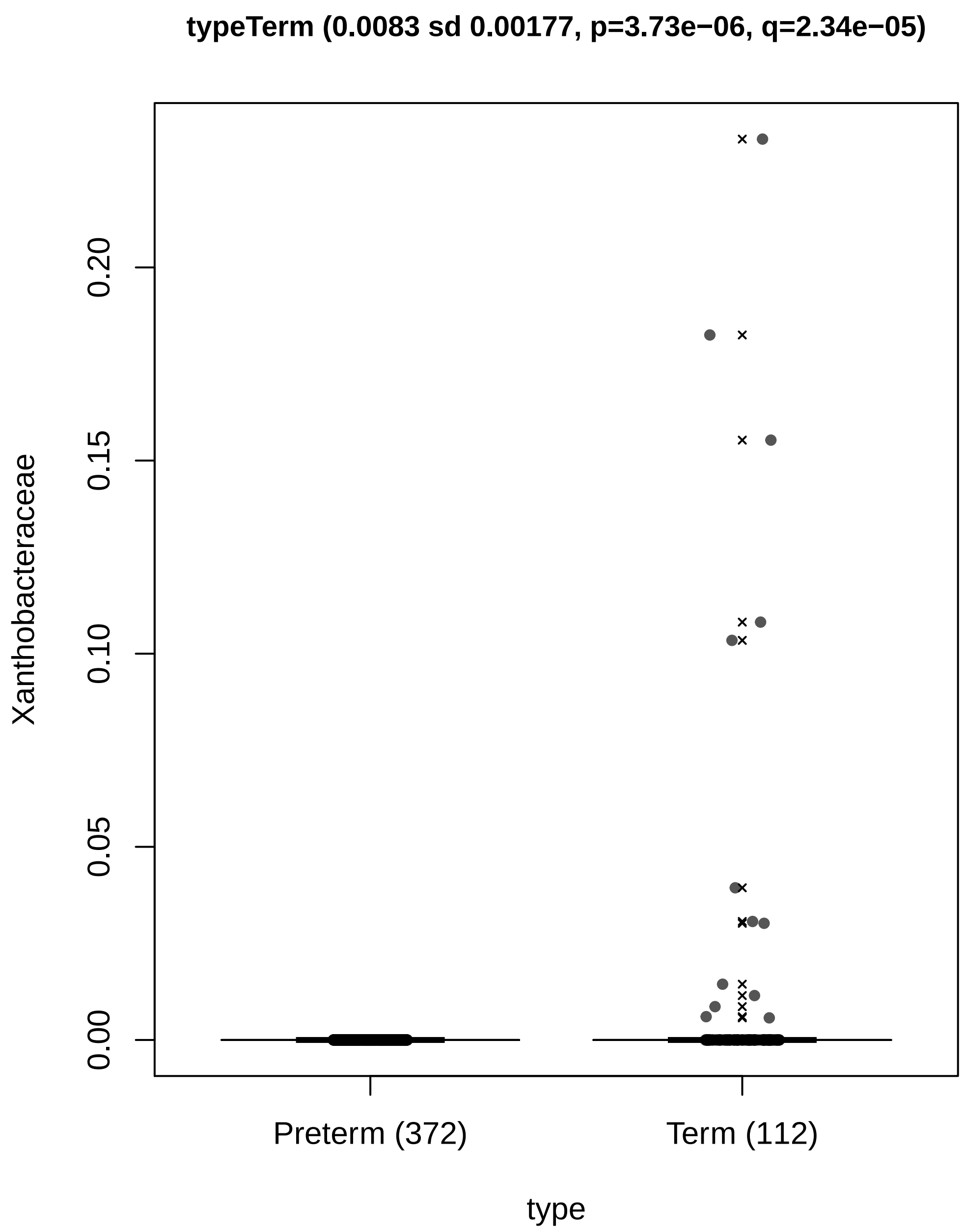

typeTerm (0.0083 sd 0.00177, p=3.73e−06, q=2.34e−05)
Xanthobacteraceae
0.00
0.05
0.10
0.15
0.20
Preterm (372)
Term (112)
type

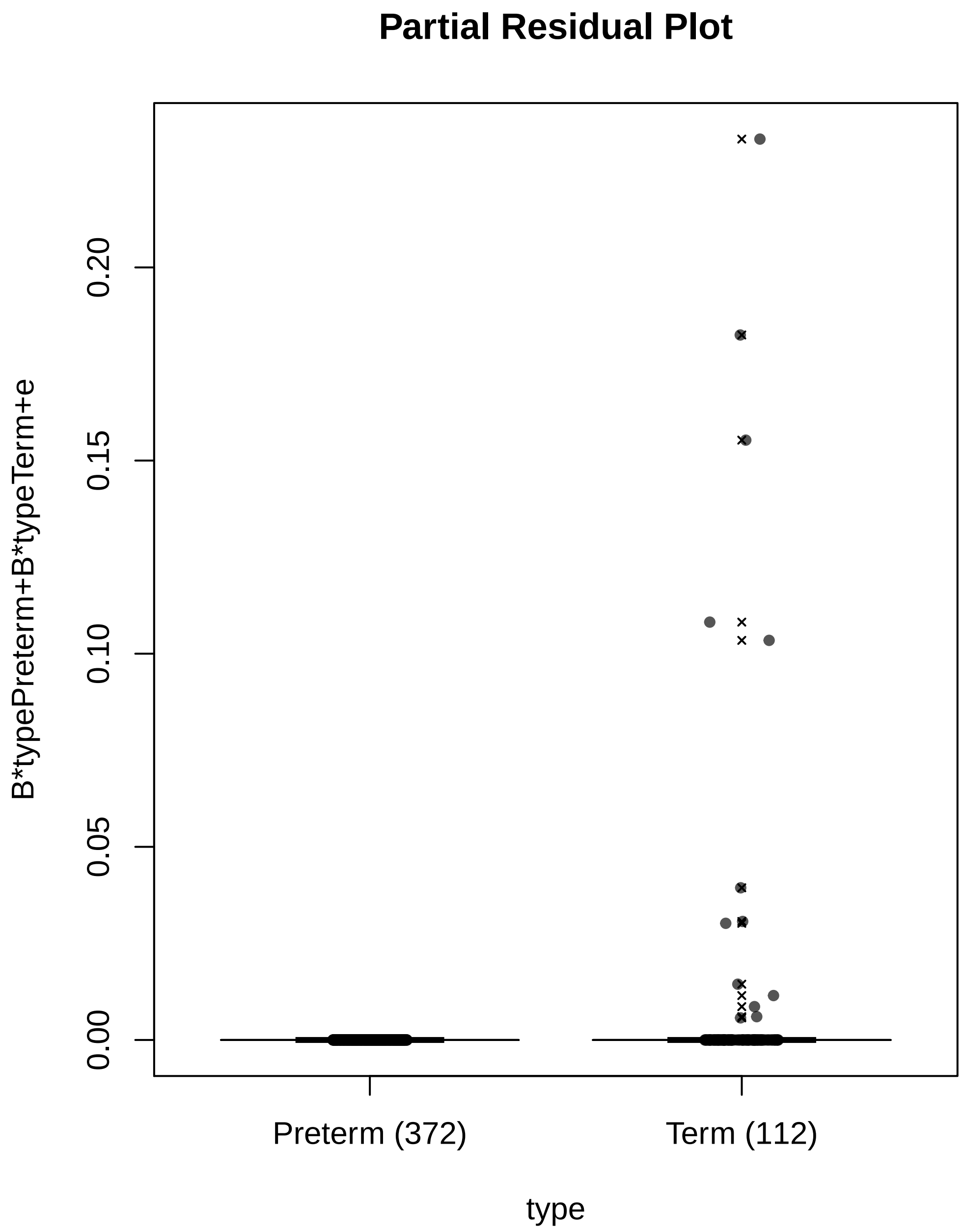

Partial Residual Plot
B*typePreterm+B*typeTerm+e
0.00
0.05
0.10
0.15
0.20
Preterm (372)
Term (112)
type

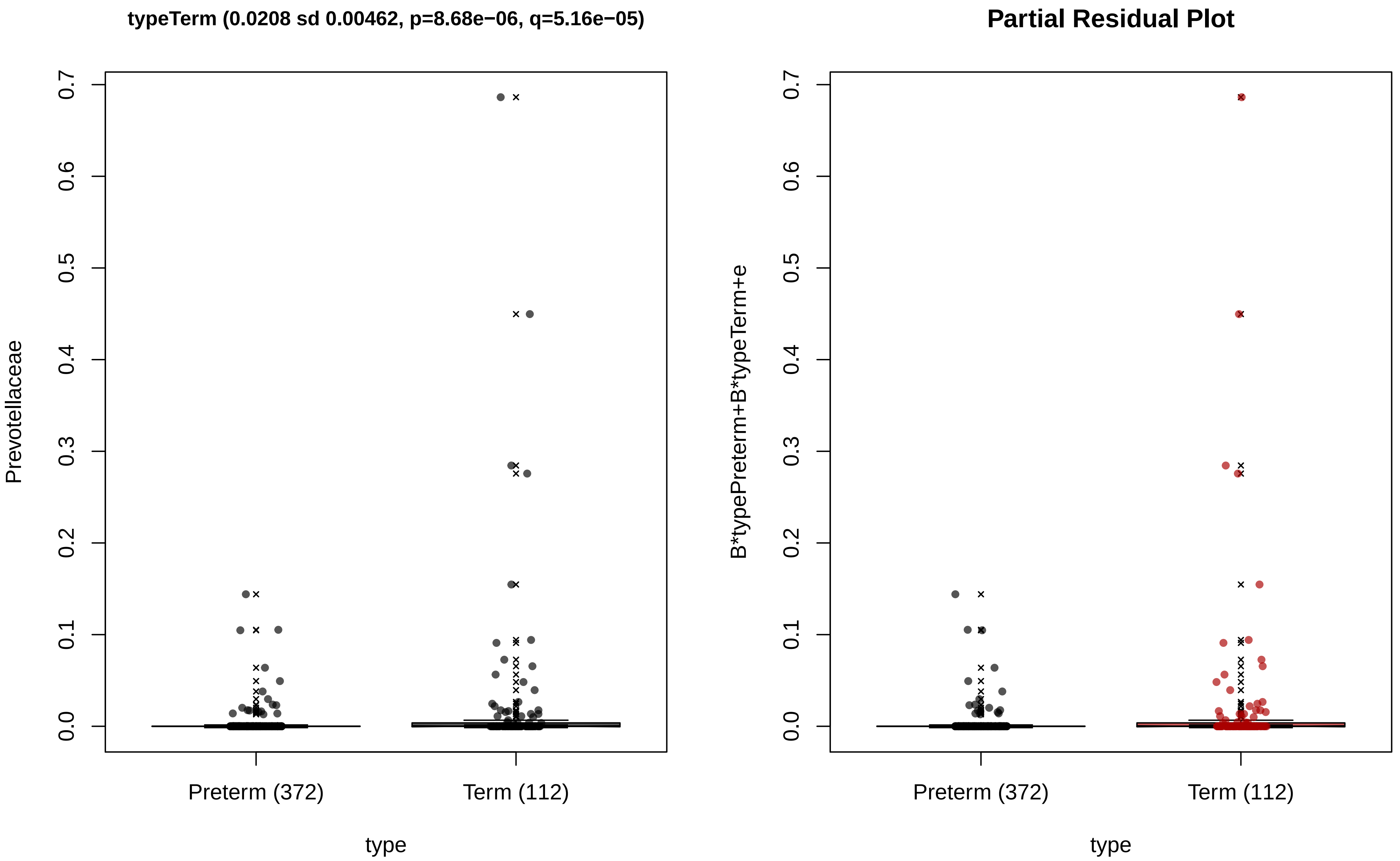

typeTerm (0.0208 sd 0.00462, p=8.68e−06, q=5.16e−05)
Prevotellaceae
0.0
0.1
0.2
0.3
0.4
0.5
0.6
0.7
Preterm (372)
Term (112)
type
Partial Residual Plot
B*typePreterm+B*typeTerm+e
0.0
0.1
0.2
0.3
0.4
0.5
0.6
0.7
Preterm (372)
Term (112)
type

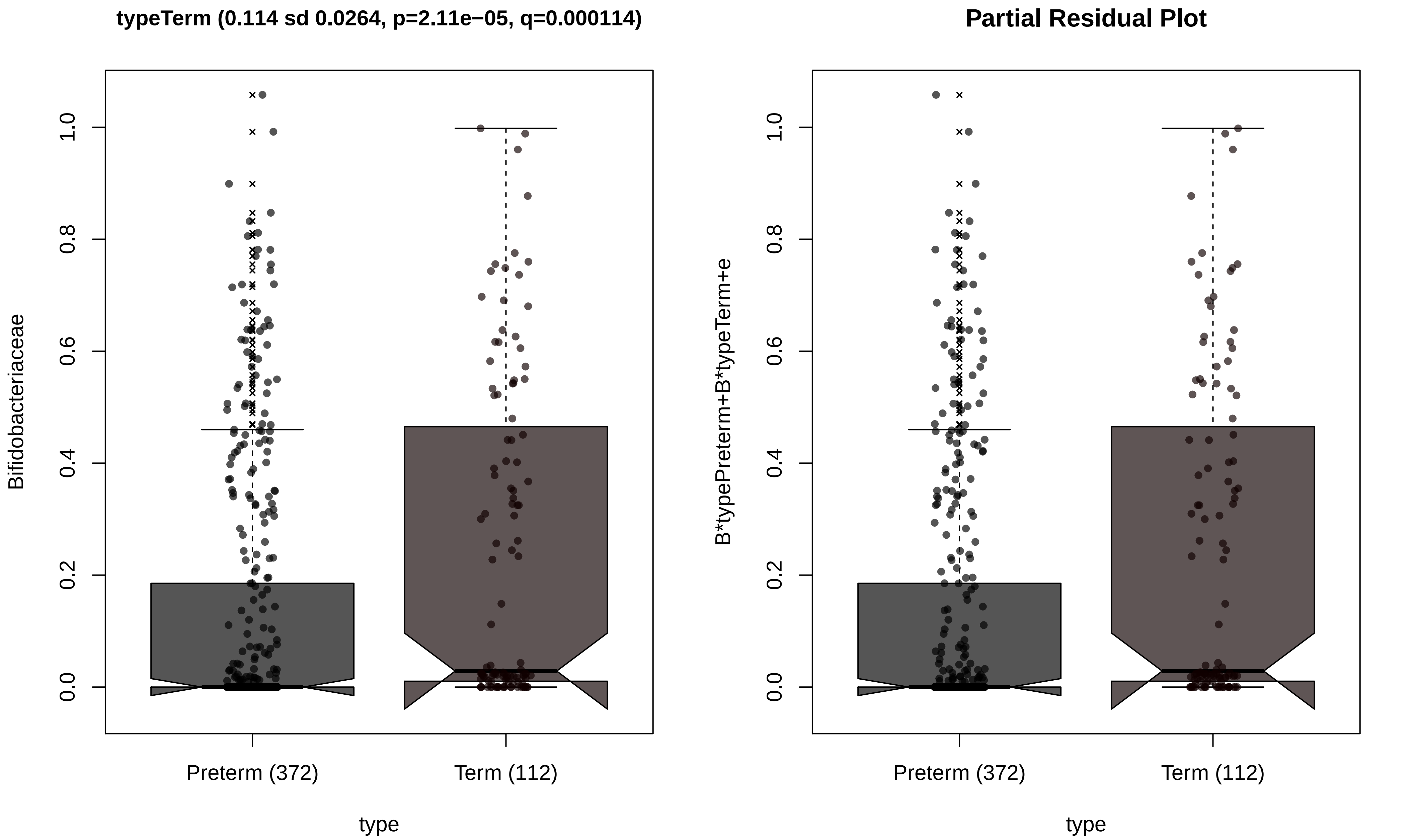
typeTerm (0.114 sd 0.0264, p=2.11e−05, q=0.000114)
Bifidobacteriaceae
0.0
0.2
0.4
0.6
0.8
1.0
Preterm (372)
Term (112)
type
Partial Residual Plot
B*typePreterm+B*typeTerm+e
0.0
0.2
0.4
0.6
0.8
1.0
Preterm (372)
Term (112)
type

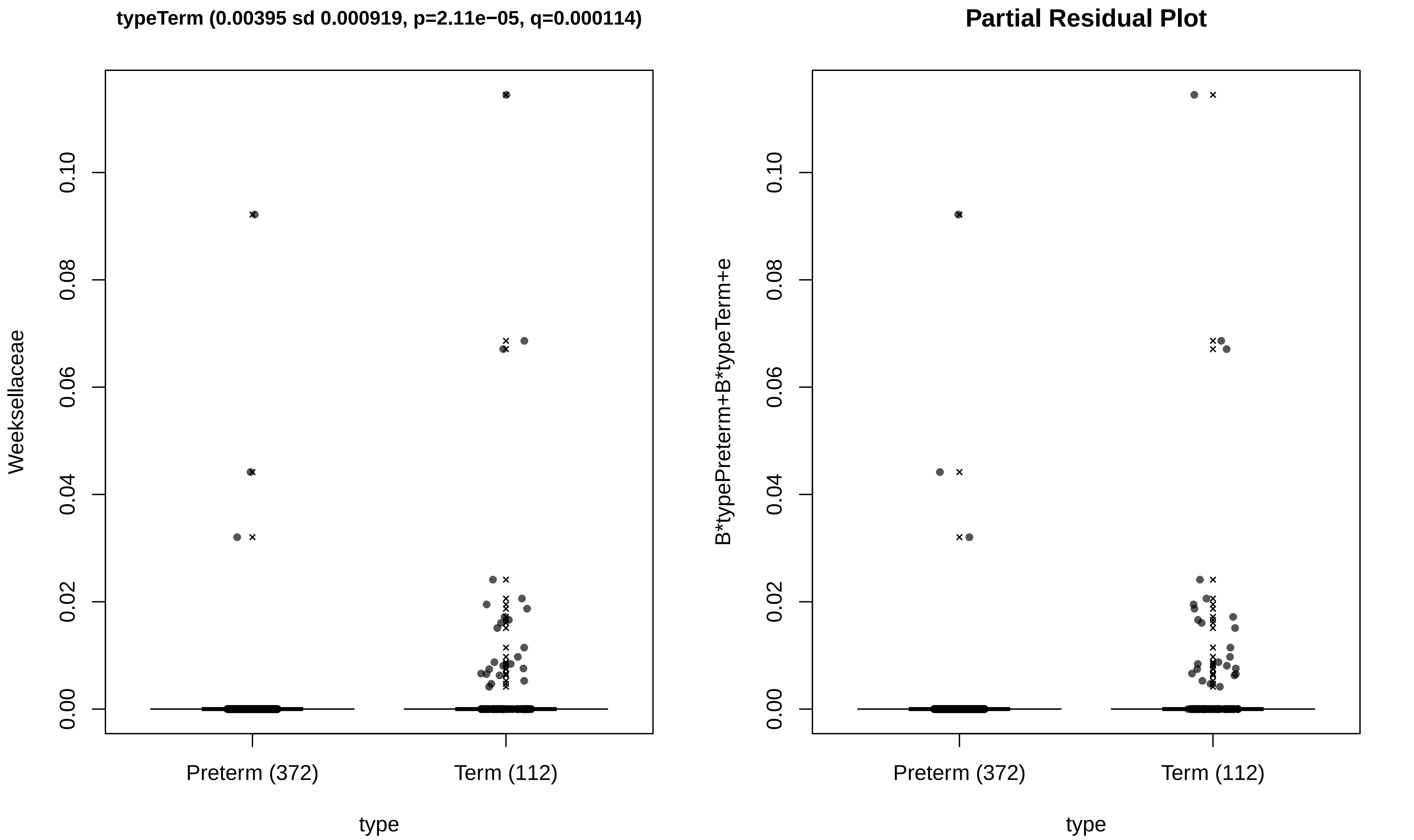
typeTerm (0.00395 sd 0.000919, p=2.11e−05, q=0.000114)
Weeksellaceae
0.00
0.02
0.04
0.06
0.08
0.10
Preterm (372)
Term (112)
type
Partial Residual Plot
B*typePreterm+B*typeTerm+e
0.00
0.02
0.04
0.06
0.08
0.10
Preterm (372)
Term (112)
type

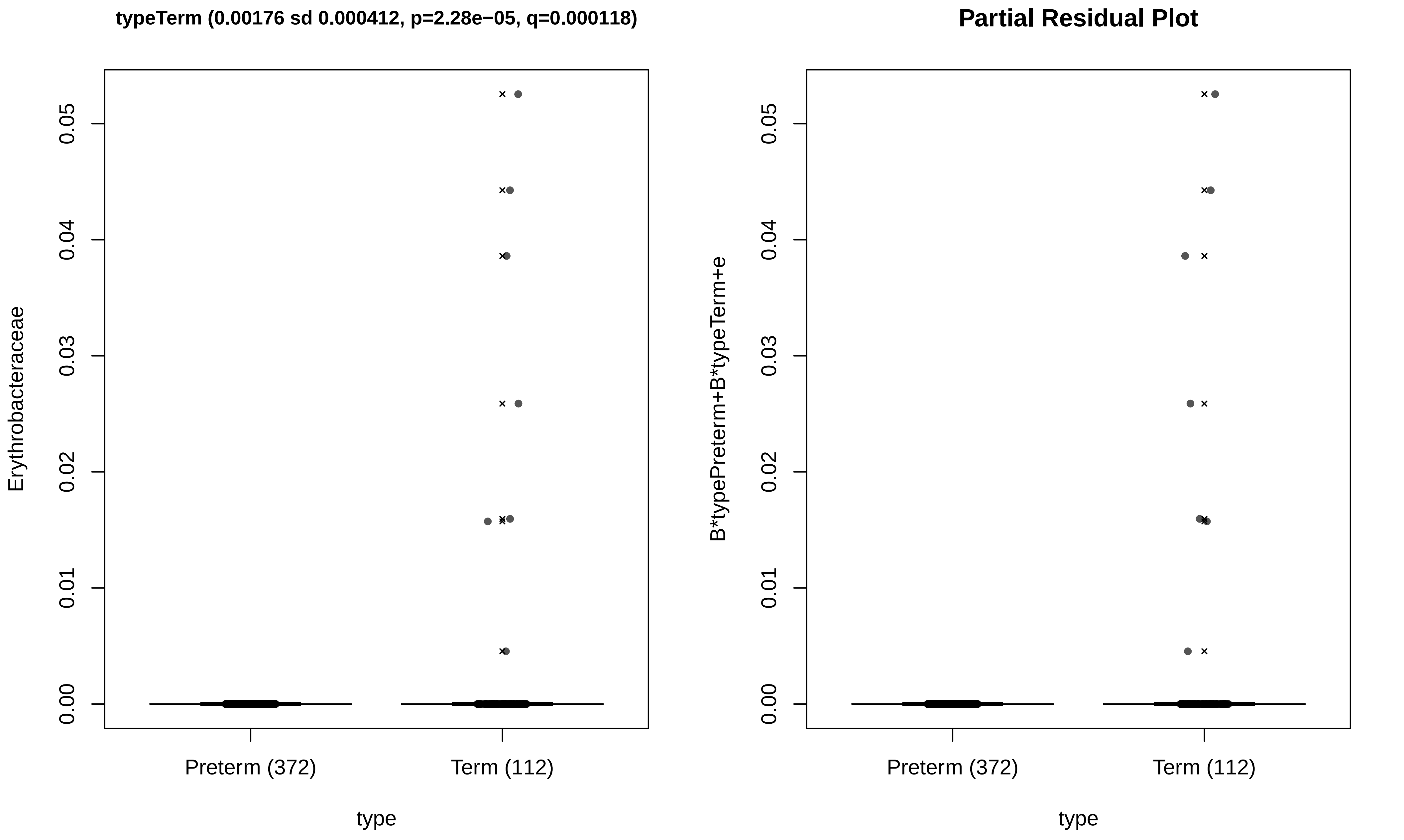
typeTerm (0.00176 sd 0.000412, p=2.28e−05, q=0.000118)
Partial Residual Plot
Erythrobacteraceae
B*typePreterm+B*typeTerm+e
0.00
0.01
0.02
0.03
0.04
0.05
Preterm (372)
Term (112)
type

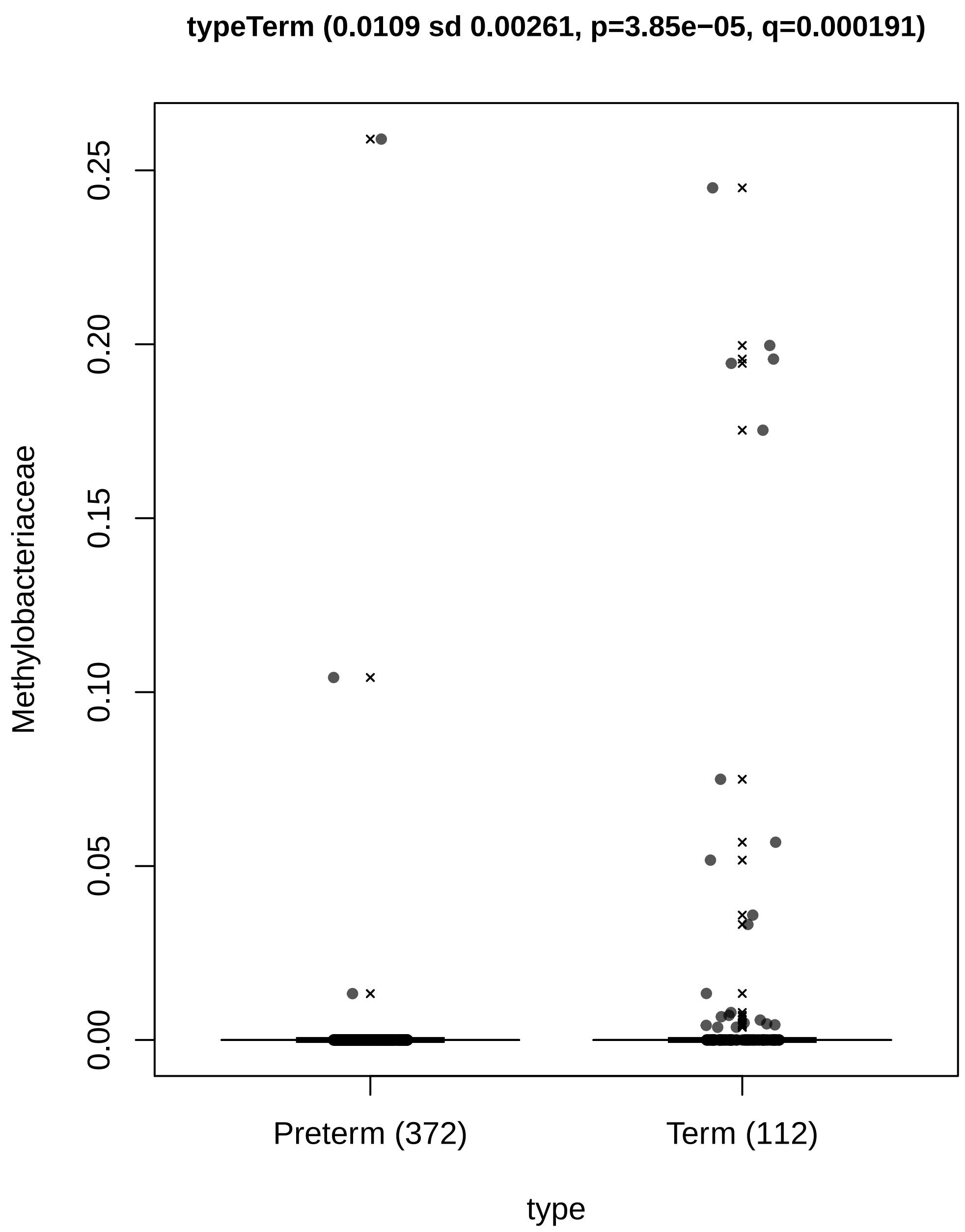
typeTerm (0.0109 sd 0.00261, p=3.85e−05, q=0.000191)
Methylobacteriaceae
0.00
0.05
0.10
0.15
0.20
0.25
Preterm (372)
Term (112)
type

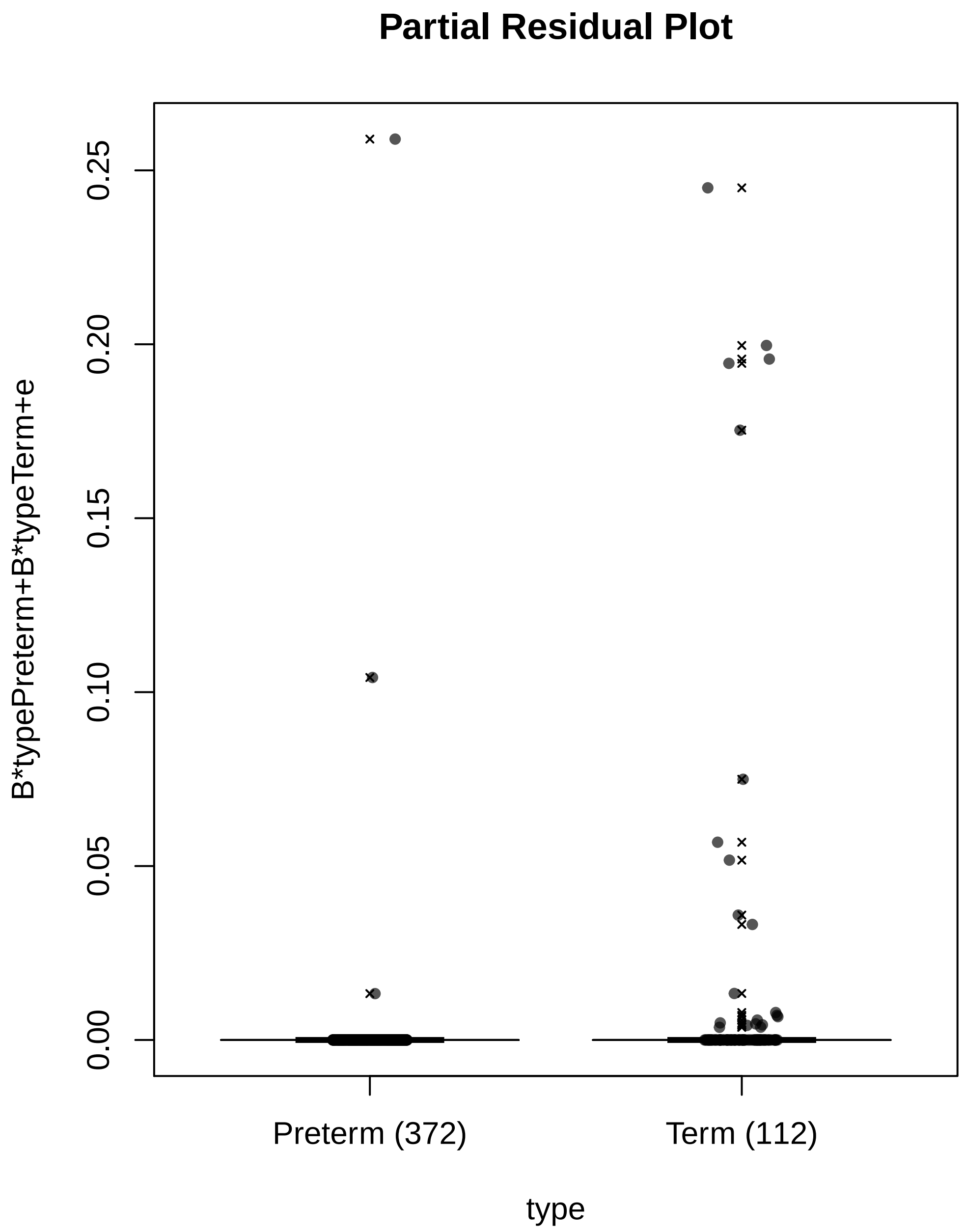
Partial Residual Plot
B*typePreterm+B*typeTerm+e
0.00
0.05
0.10
0.15
0.20
0.25
Preterm (372)
Term (112)
type

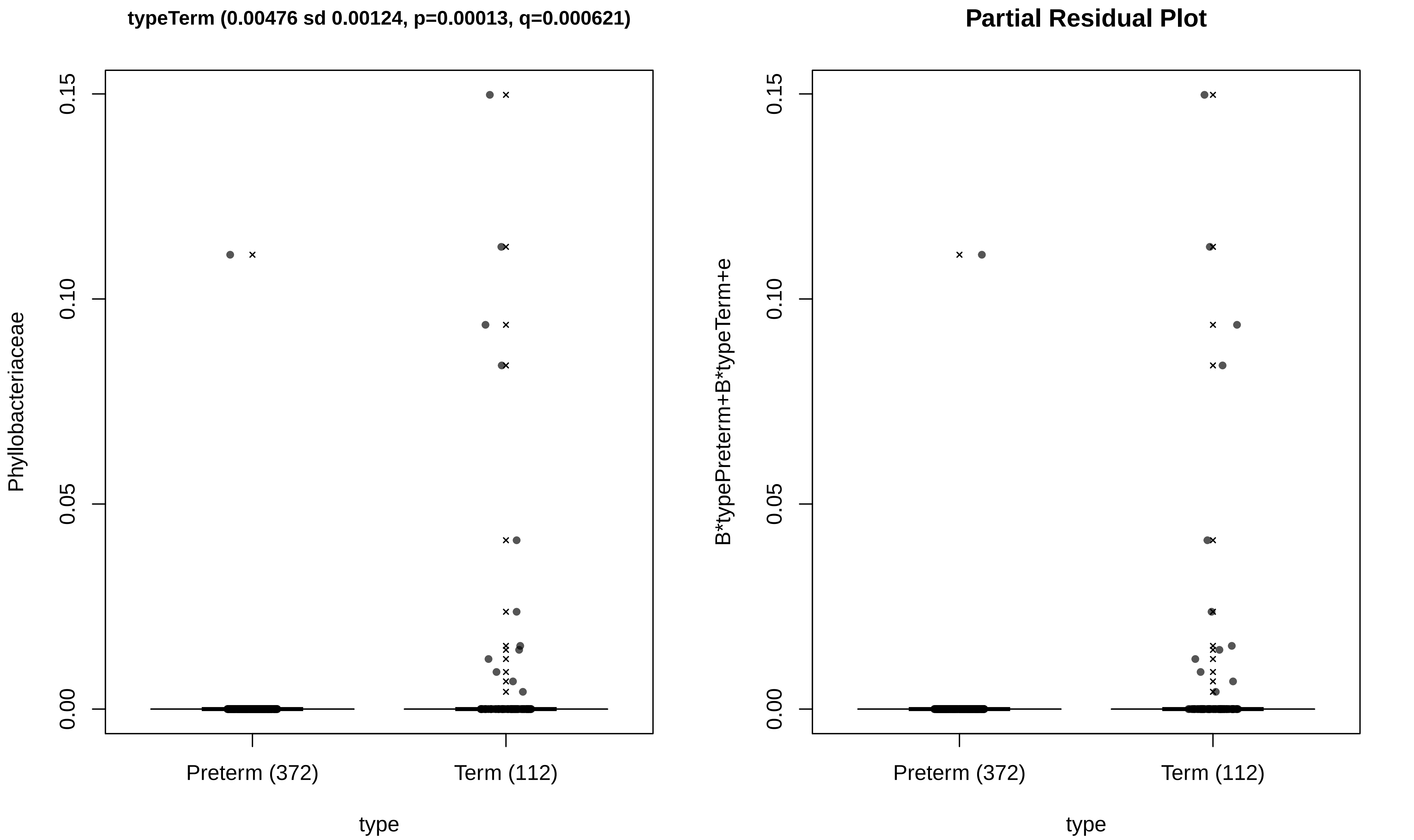
typeTerm (0.00476 sd 0.00124, p=0.00013, q=0.000621)
Partial Residual Plot
Phyllobacteriaceae
B*typePreterm+B*typeTerm+e
0.00
0.05
0.10
0.15
Preterm (372)
Term (112)
type

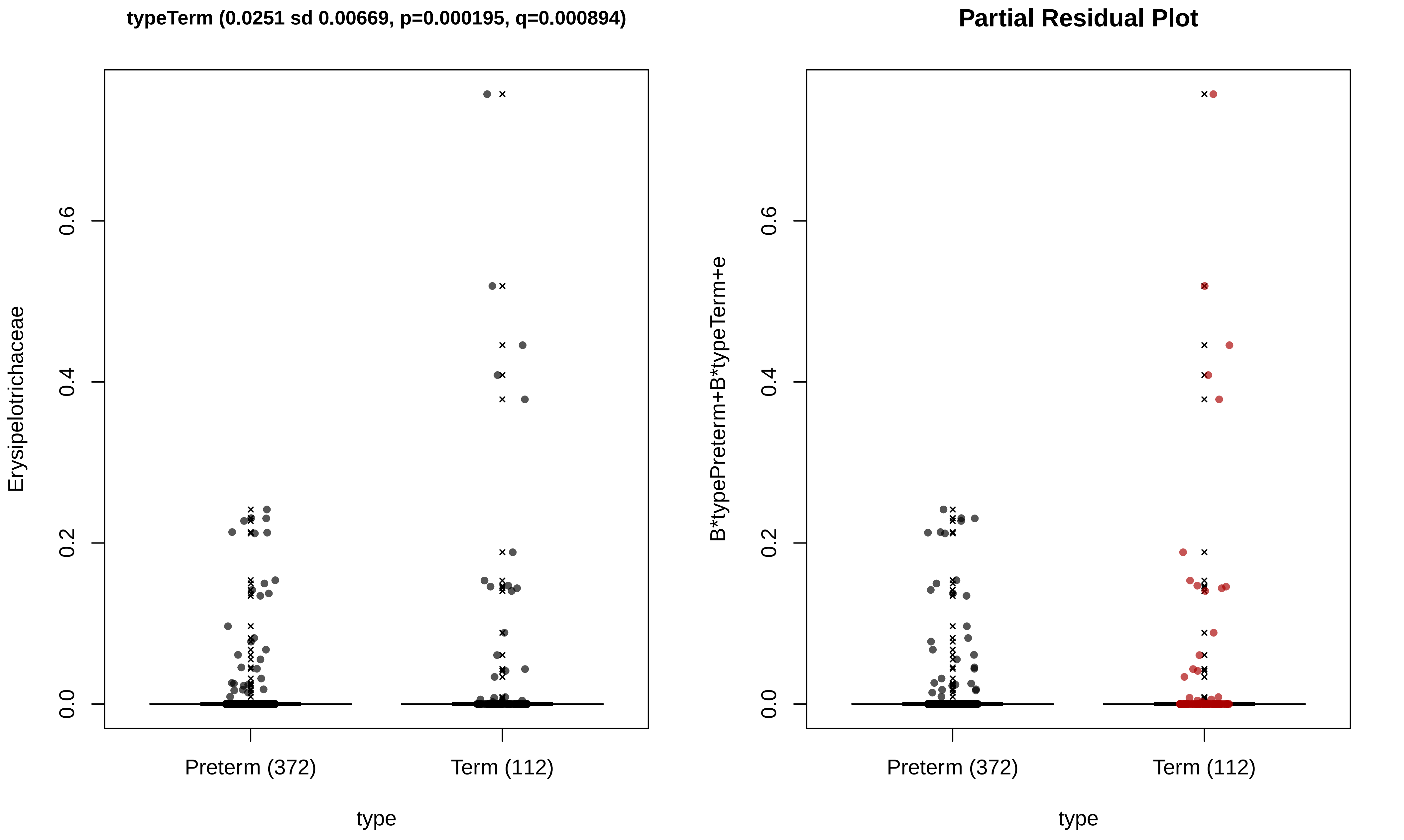
typeTerm (0.0251 sd 0.00669, p=0.000195, q=0.000894)
Partial Residual Plot
Erysipelotrichaceae
B*typePreterm+B*typeTerm+e
0.0
0.2
0.4
0.6
Preterm (372)
Term (112)
type

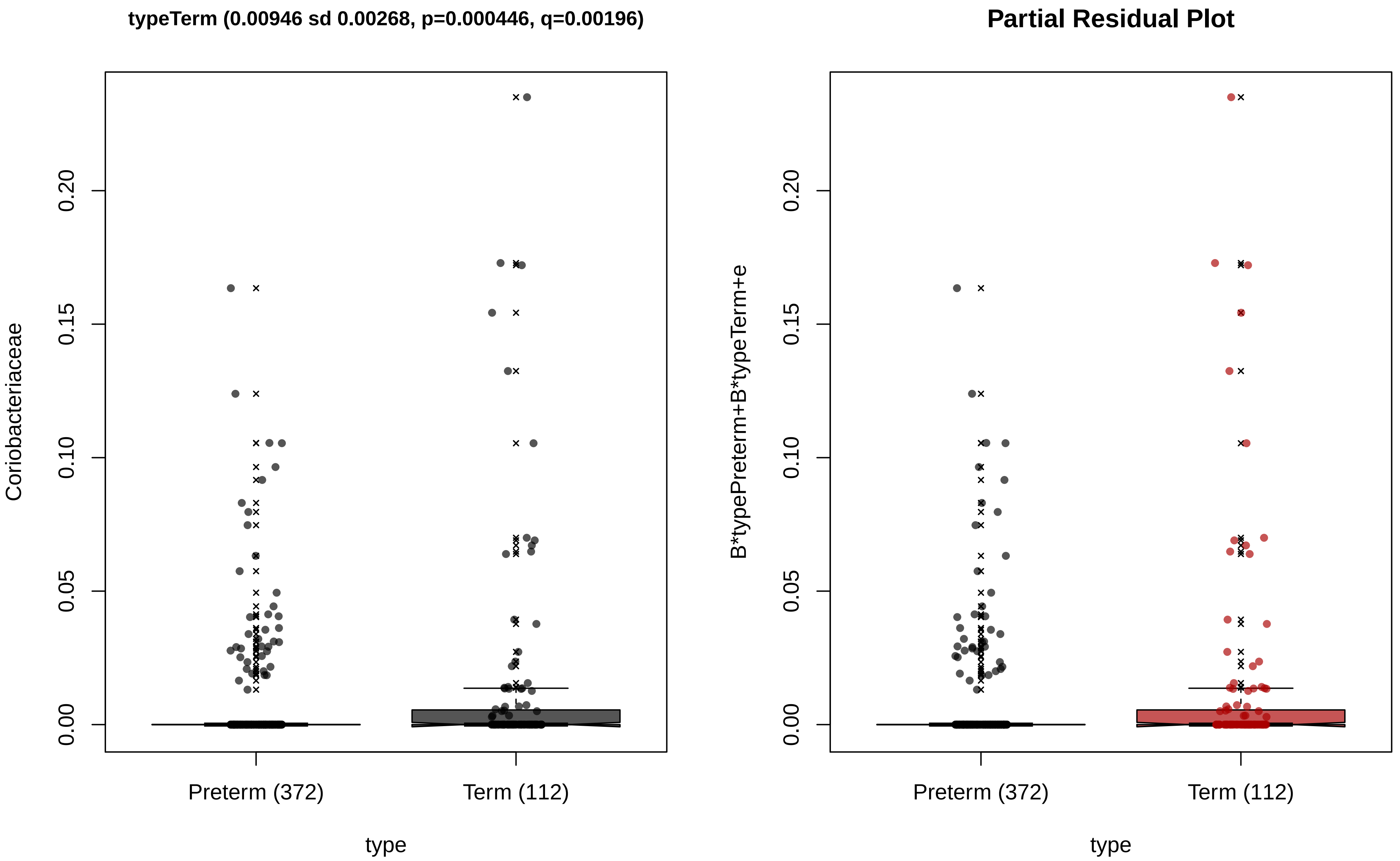
typeTerm (0.00946 sd 0.00268, p=0.000446, q=0.00196)
Partial Residual Plot
Coriobacteriaceae
B*typePreterm+B*typeTerm+e
0.00
0.05
0.10
0.15
0.20
Preterm (372)
Term (112)
type

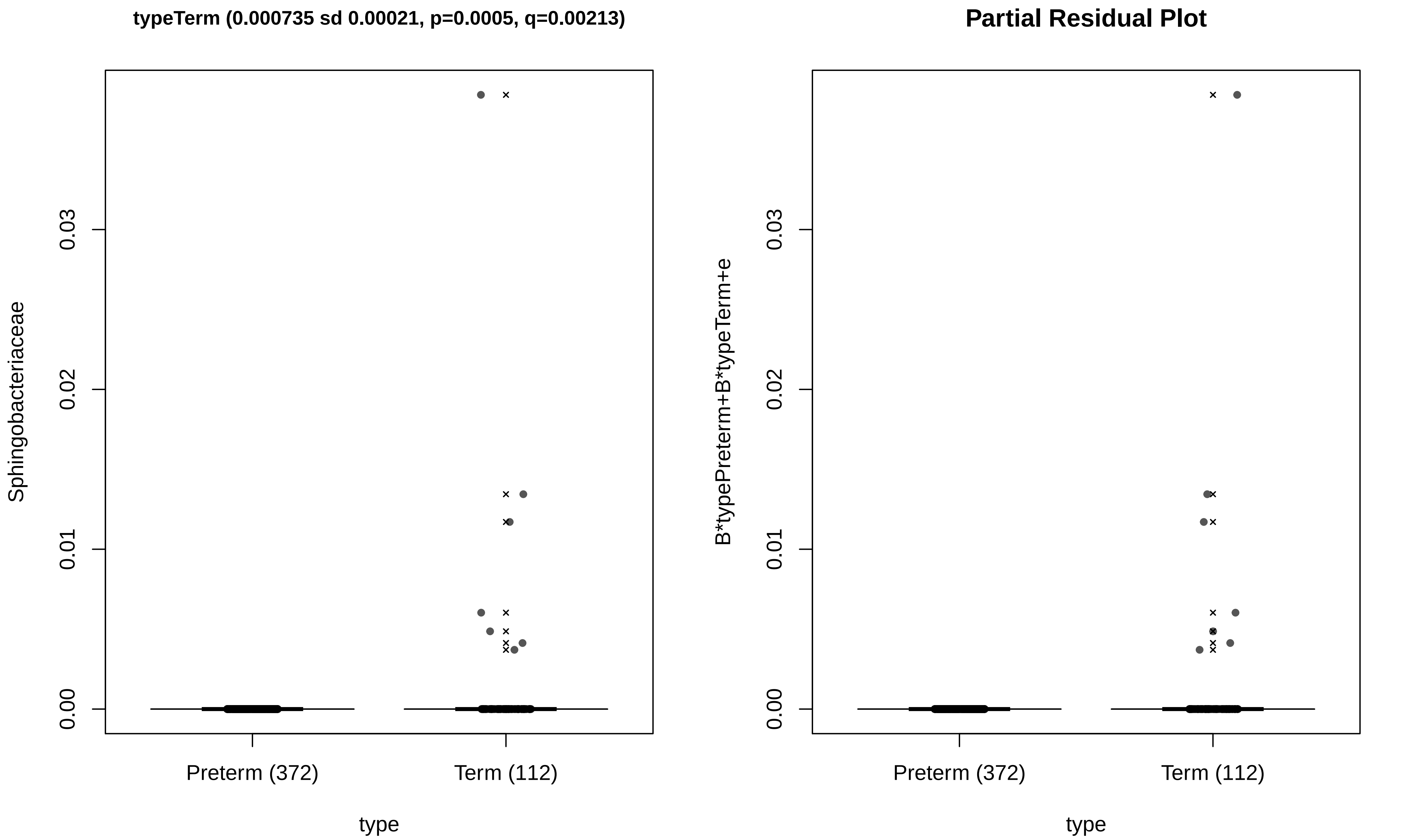
typeTerm (0.000735 sd 0.00021, p=0.0005, q=0.00213)
Sphingobacteriaceae
0.00
0.01
0.02
0.03
Preterm (372)
Term (112)
type
Partial Residual Plot
B*typePreterm+B*typeTerm+e
0.00
0.01
0.02
0.03
Preterm (372)
Term (112)
type

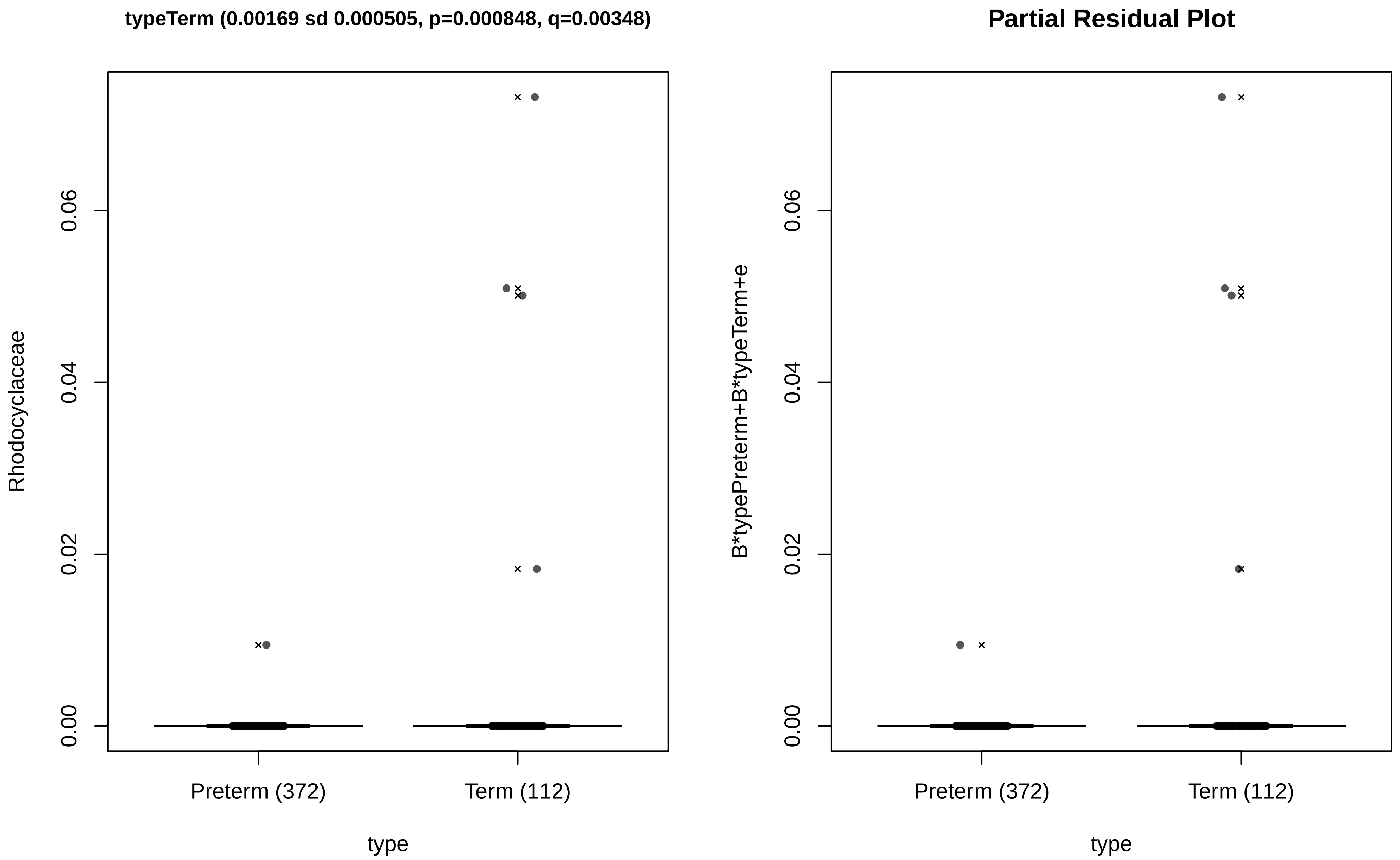
typeTerm (0.00169 sd 0.000505, p=0.000848, q=0.00348)
Partial Residual Plot
Rhodocyclaceae
B*typePreterm+B*typeTerm+e
0.00
0.02
0.04
0.06
Preterm (372)
Term (112)
type

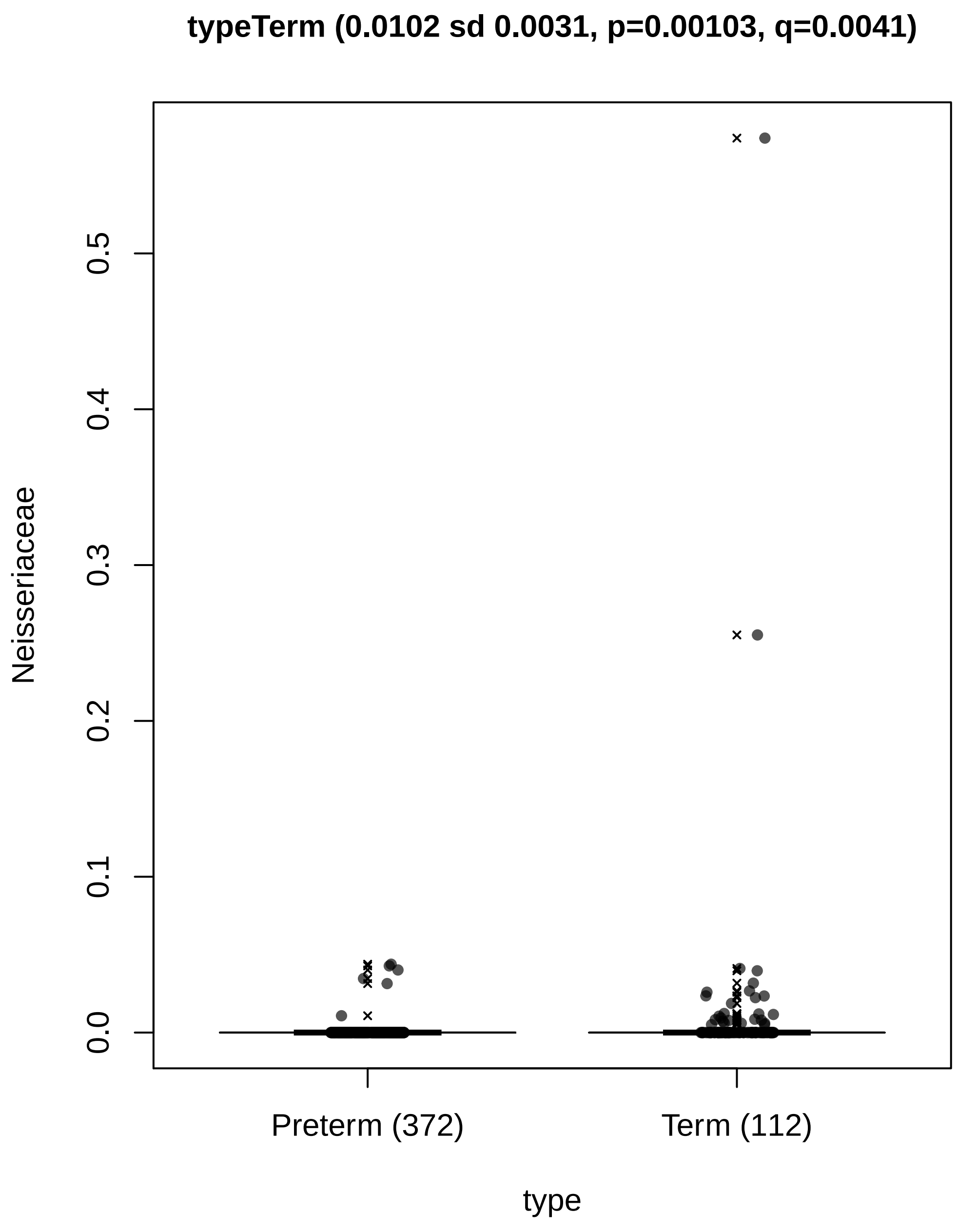
typeTerm (0.0102 sd 0.0031, p=0.00103, q=0.0041)
Neisseriaceae
0.0
0.1
0.2
0.3
0.4
0.5
Preterm (372)
Term (112)
type

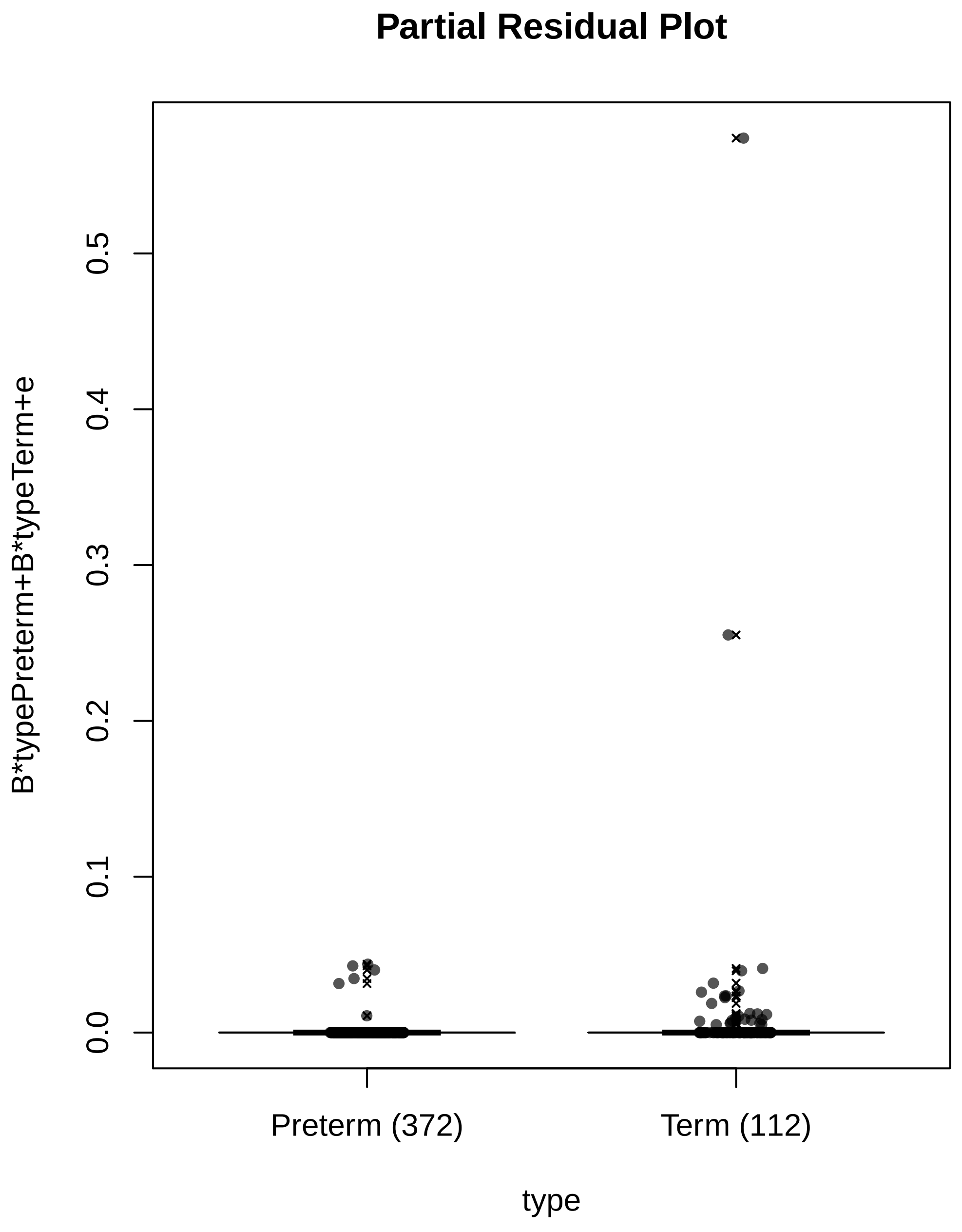
Partial Residual Plot
B*typePreterm+B*typeTerm+e
0.0
0.1
0.2
0.3
0.4
0.5
Preterm (372)
Term (112)
type

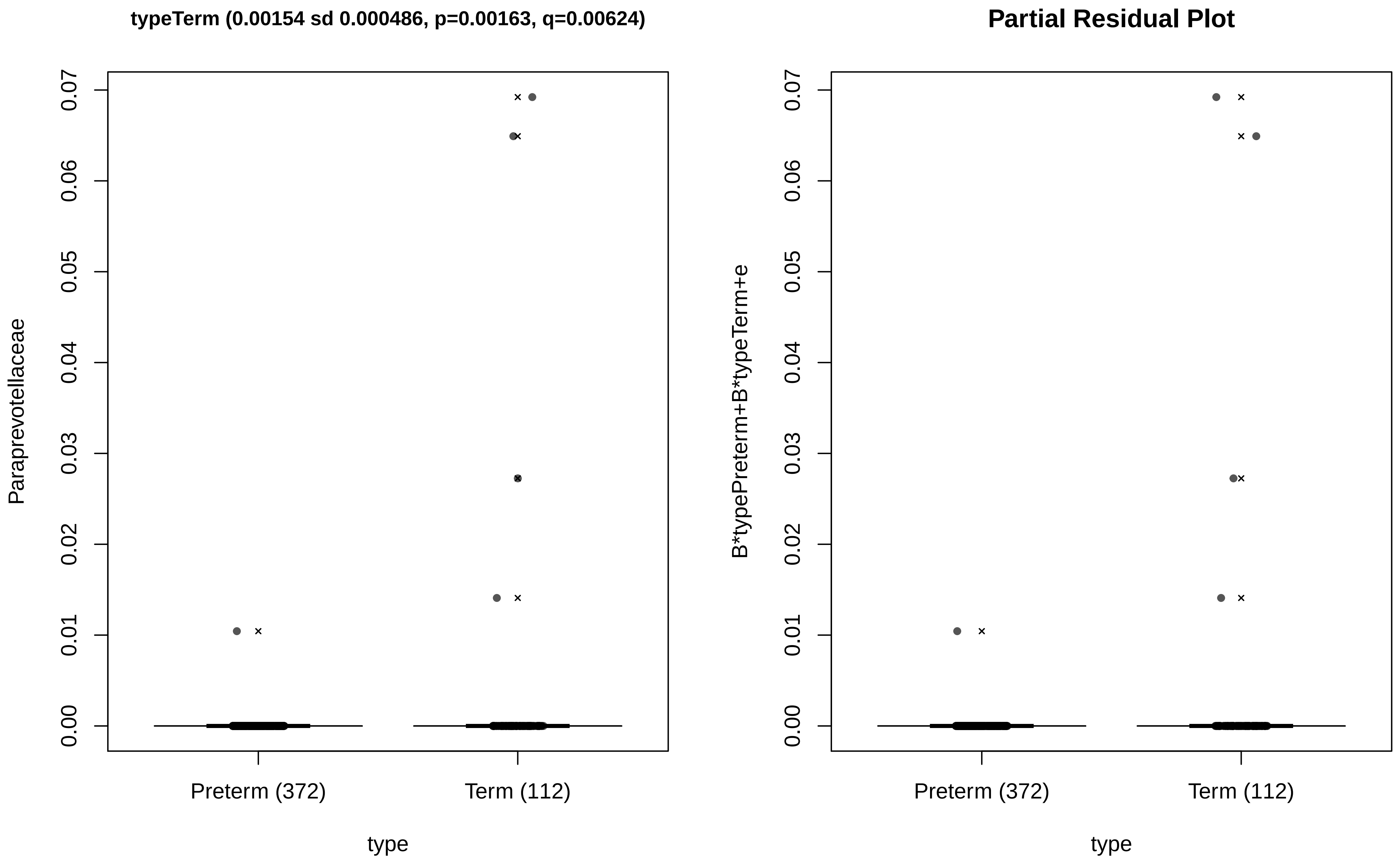
typeTerm (0.00154 sd 0.000486, p=0.00163, q=0.00624)
Paraprevotellaceae
0.00
0.01
0.02
0.03
0.04
0.05
0.06
0.07
Preterm (372)
Term (112)
type
Partial Residual Plot
B*typePreterm+B*typeTerm+e
0.00
0.01
0.02
0.03
0.04
0.05
0.06
0.07
Preterm (372)
Term (112)
type

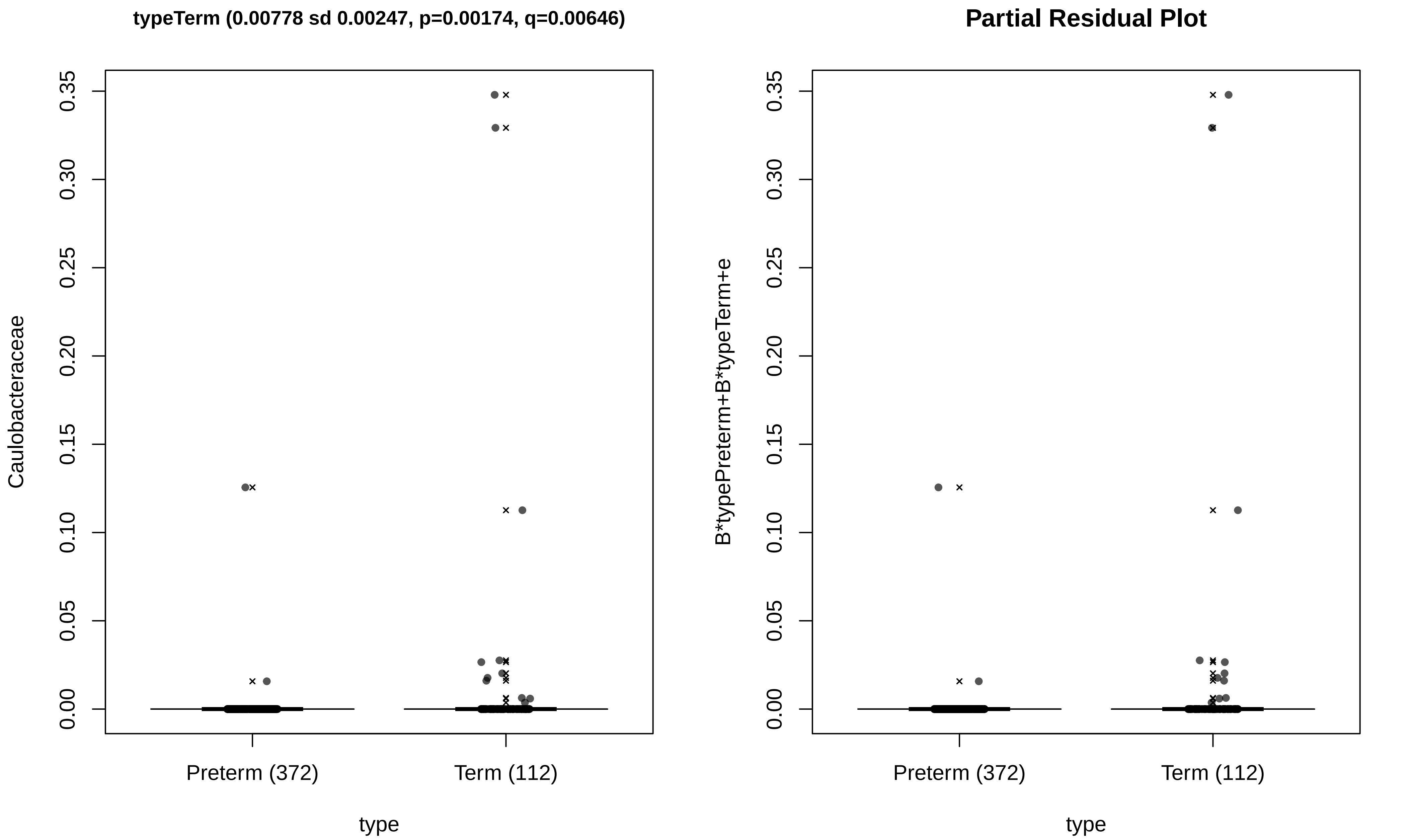
typeTerm (0.00778 sd 0.00247, p=0.00174, q=0.00646)
Partial Residual Plot
Caulobacteraceae
B*typePreterm+B*typeTerm+e
0.00
0.05
0.10
0.15
0.20
0.25
0.30
0.35
Preterm (372)
Term (112)
type

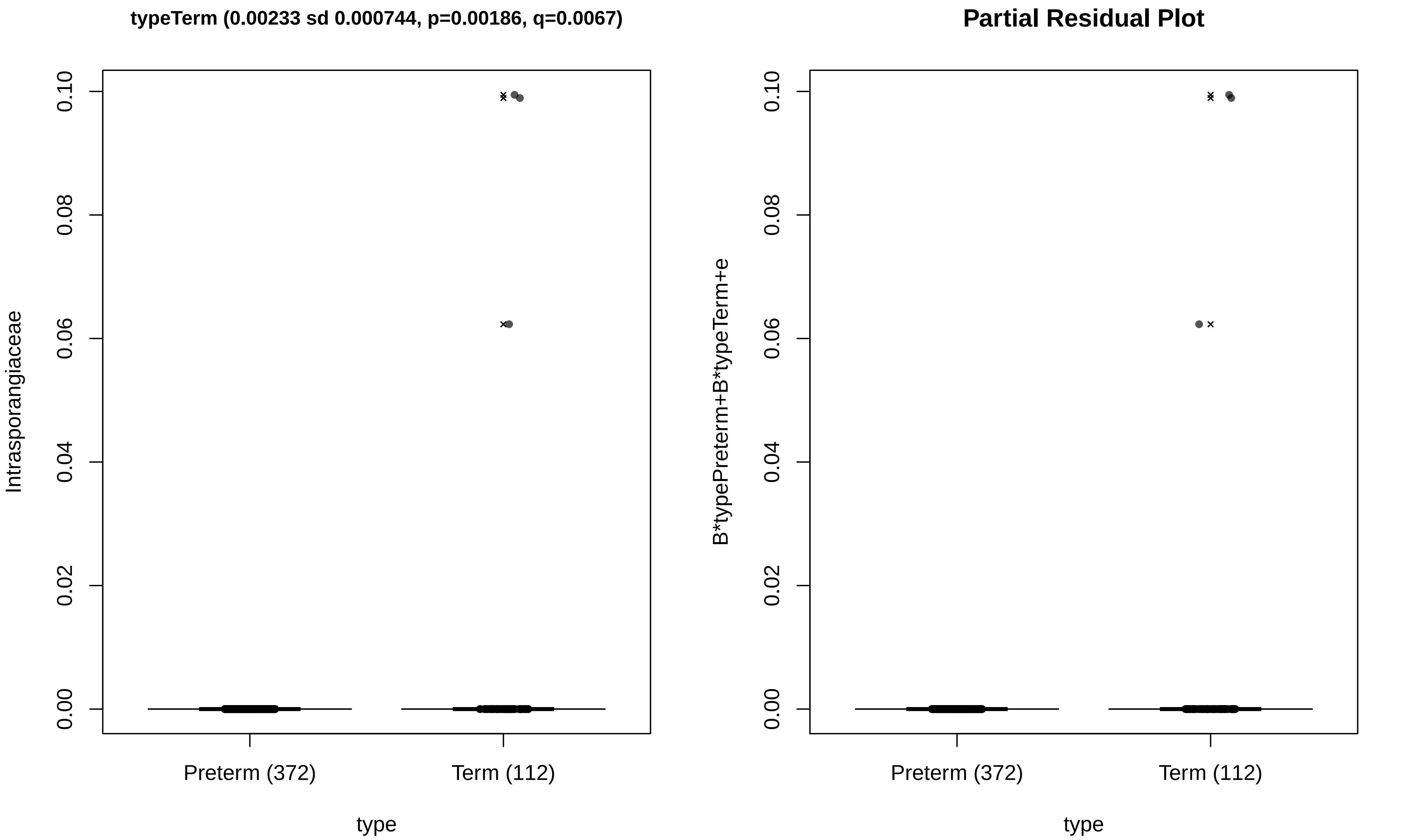
typeTerm (0.00233 sd 0.000744, p=0.00186, q=0.0067)
Intrasporangiaceae
0.00
0.02
0.04
0.06
0.08
0.10
Preterm (372)
Term (112)
type
Partial Residual Plot
B*typePreterm+B*typeTerm+e
0.00
0.02
0.04
0.06
0.08
0.10
Preterm (372)
Term (112)
type

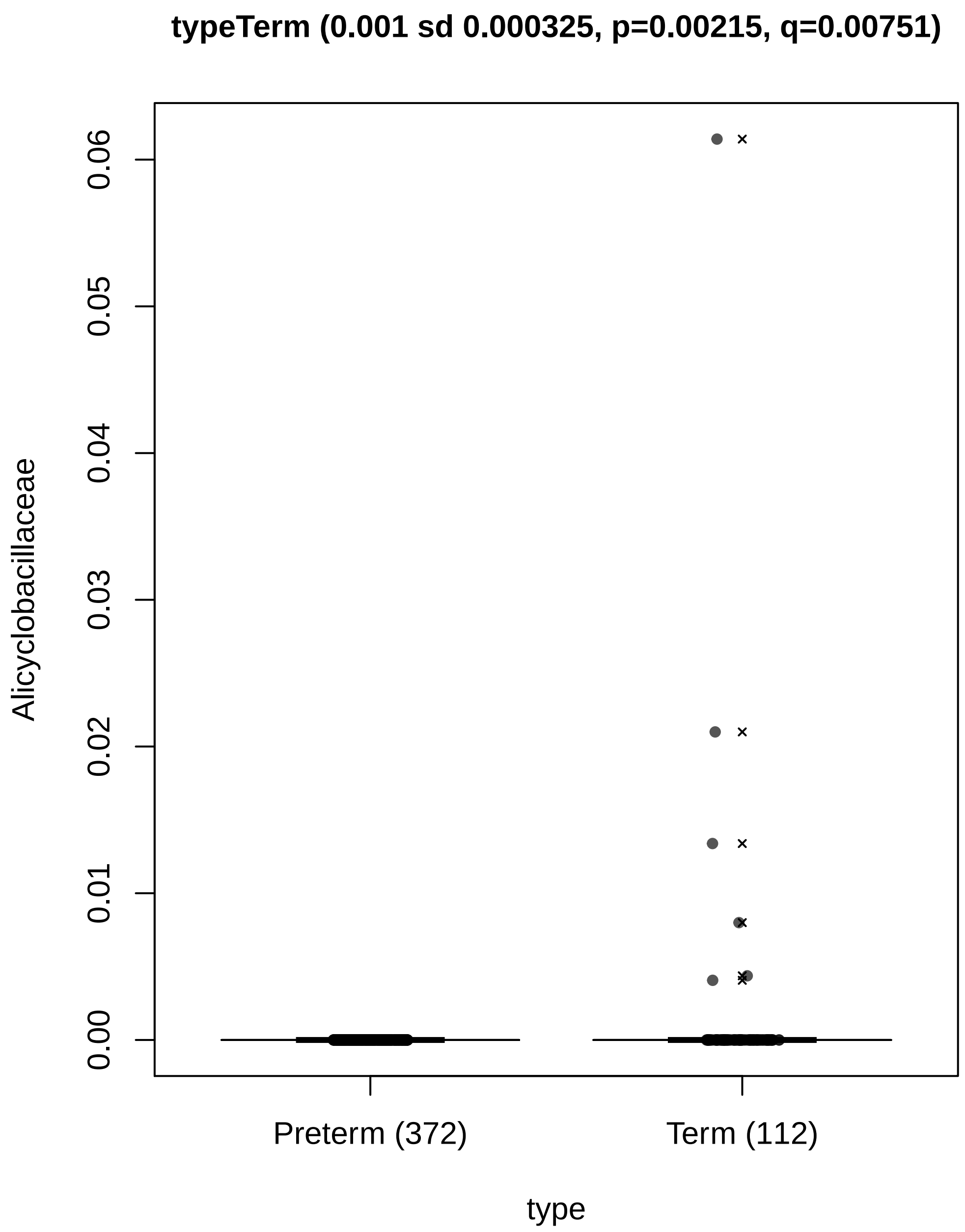
typeTerm (0.001 sd 0.000325, p=0.00215, q=0.00751)
Alicyclobacillaceae
0.00
0.01
0.02
0.03
0.04
0.05
0.06
Preterm (372)
Term (112)
type

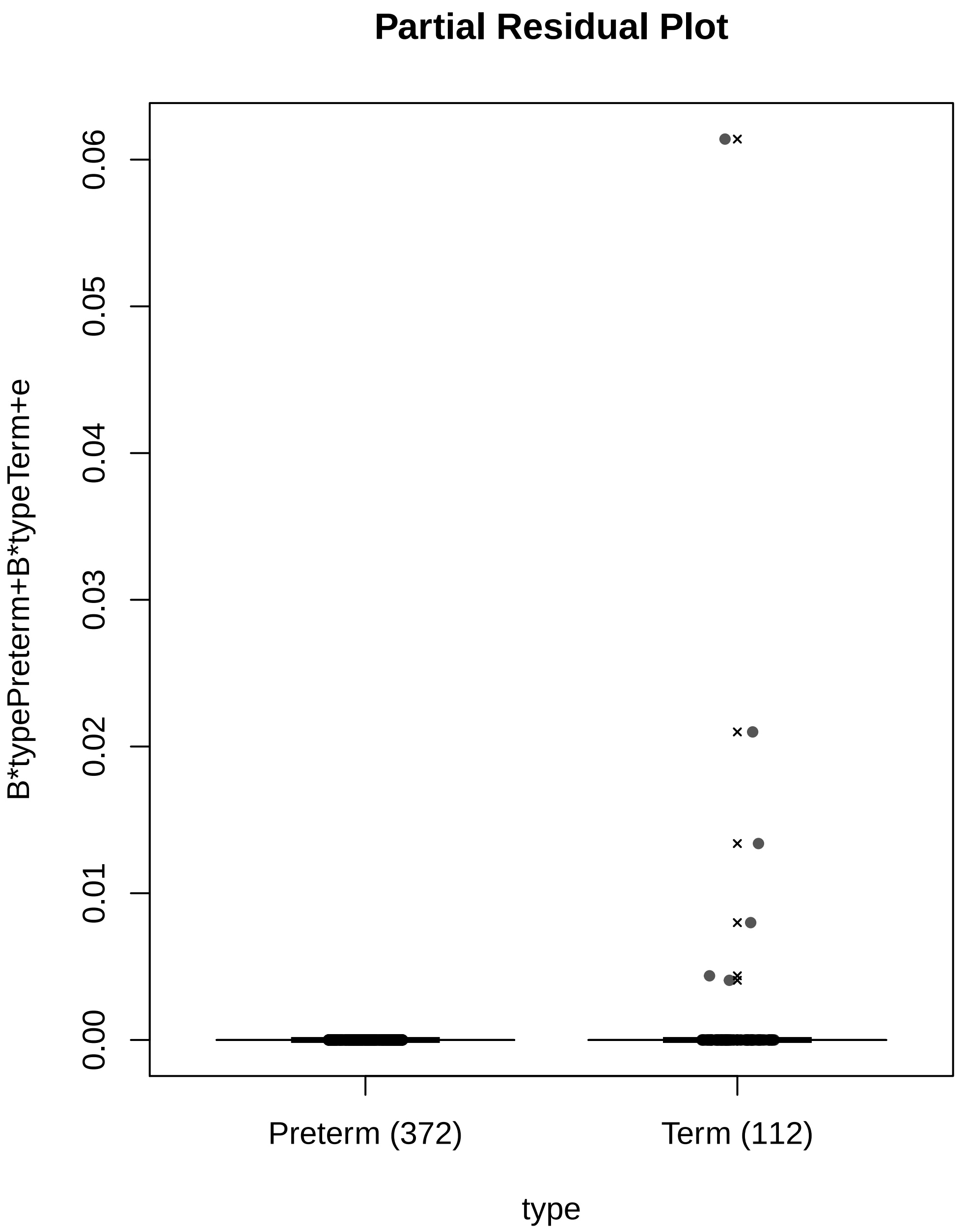
Partial Residual Plot
B*typePreterm+B*typeTerm+e
0.00
0.01
0.02
0.03
0.04
0.05
0.06
Preterm (372)
Term (112)
type

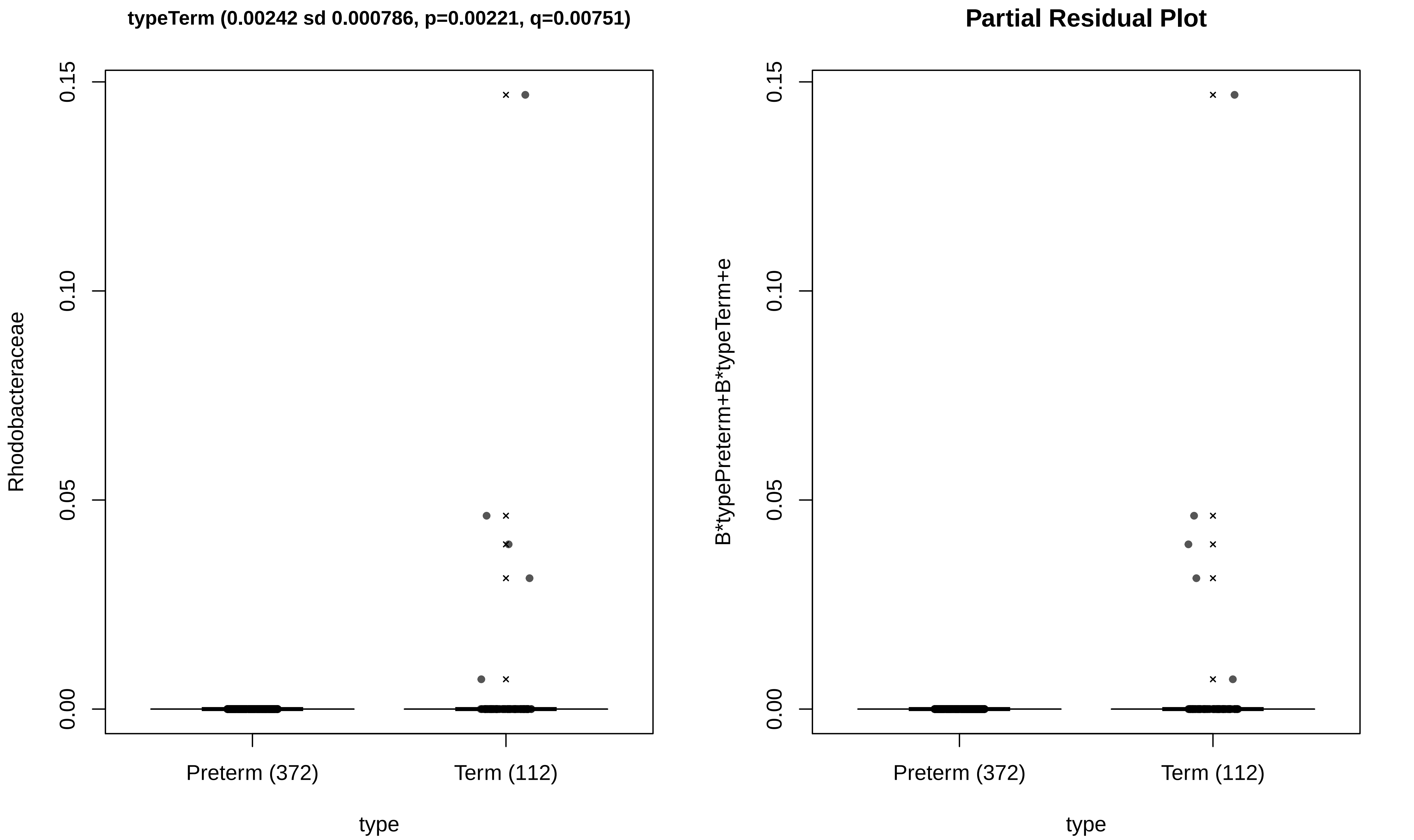
typeTerm (0.00242 sd 0.000786, p=0.00221, q=0.00751)
Rhodobacteraceae
0.00
0.05
0.10
0.15
Preterm (372)
Term (112)
type
Partial Residual Plot
B*typePreterm+B*typeTerm+e
0.00
0.05
0.10
0.15
Preterm (372)
Term (112)
type

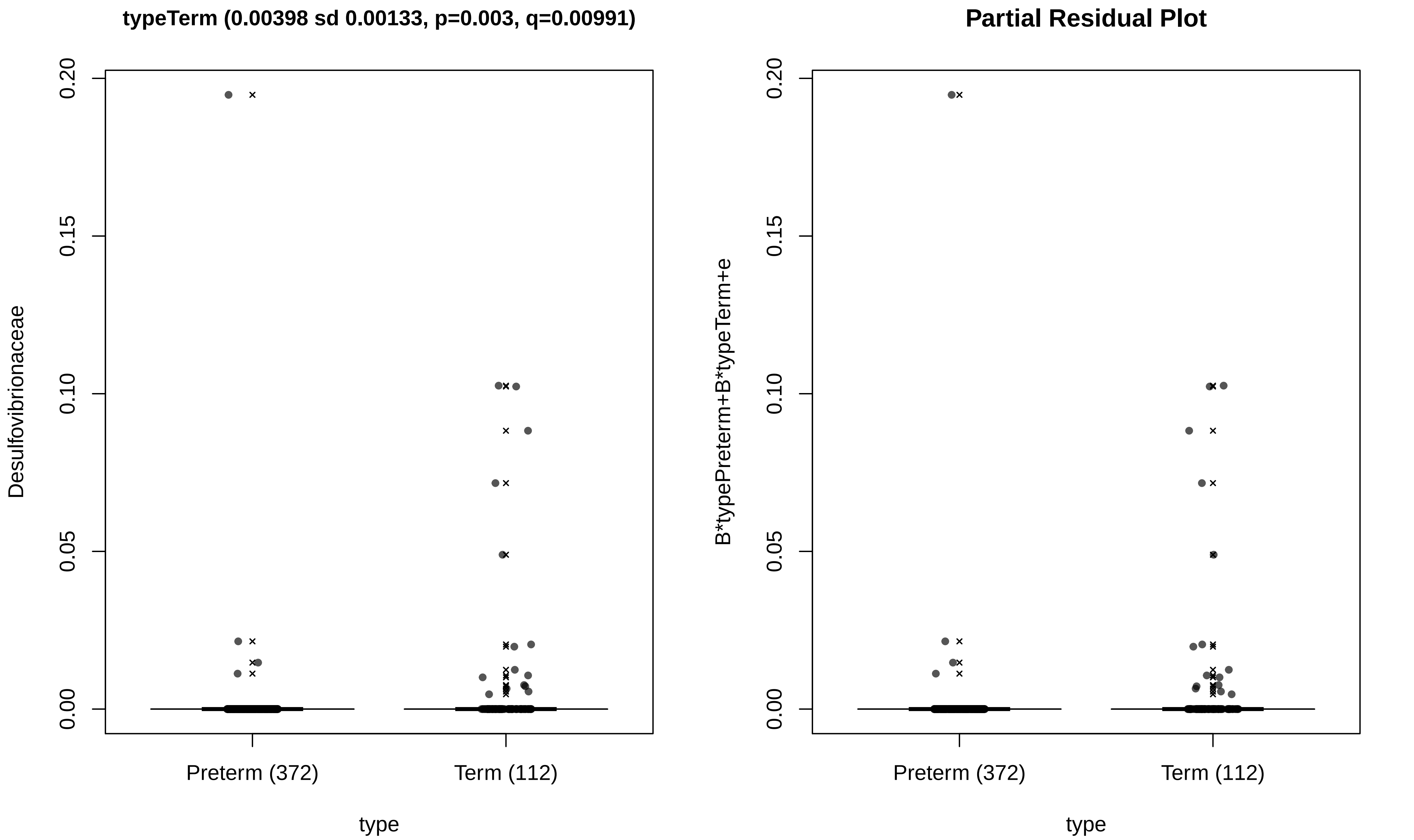

typeTerm (0.00398 sd 0.00133, p=0.003, q=0.00991)
Desulfovibrionaceae
0.00
0.05
0.10
0.15
0.20
Preterm (372)
Term (112)
type
Partial Residual Plot
B*typePreterm+B*typeTerm+e
0.00
0.05
0.10
0.15
0.20
Preterm (372)
Term (112)
type

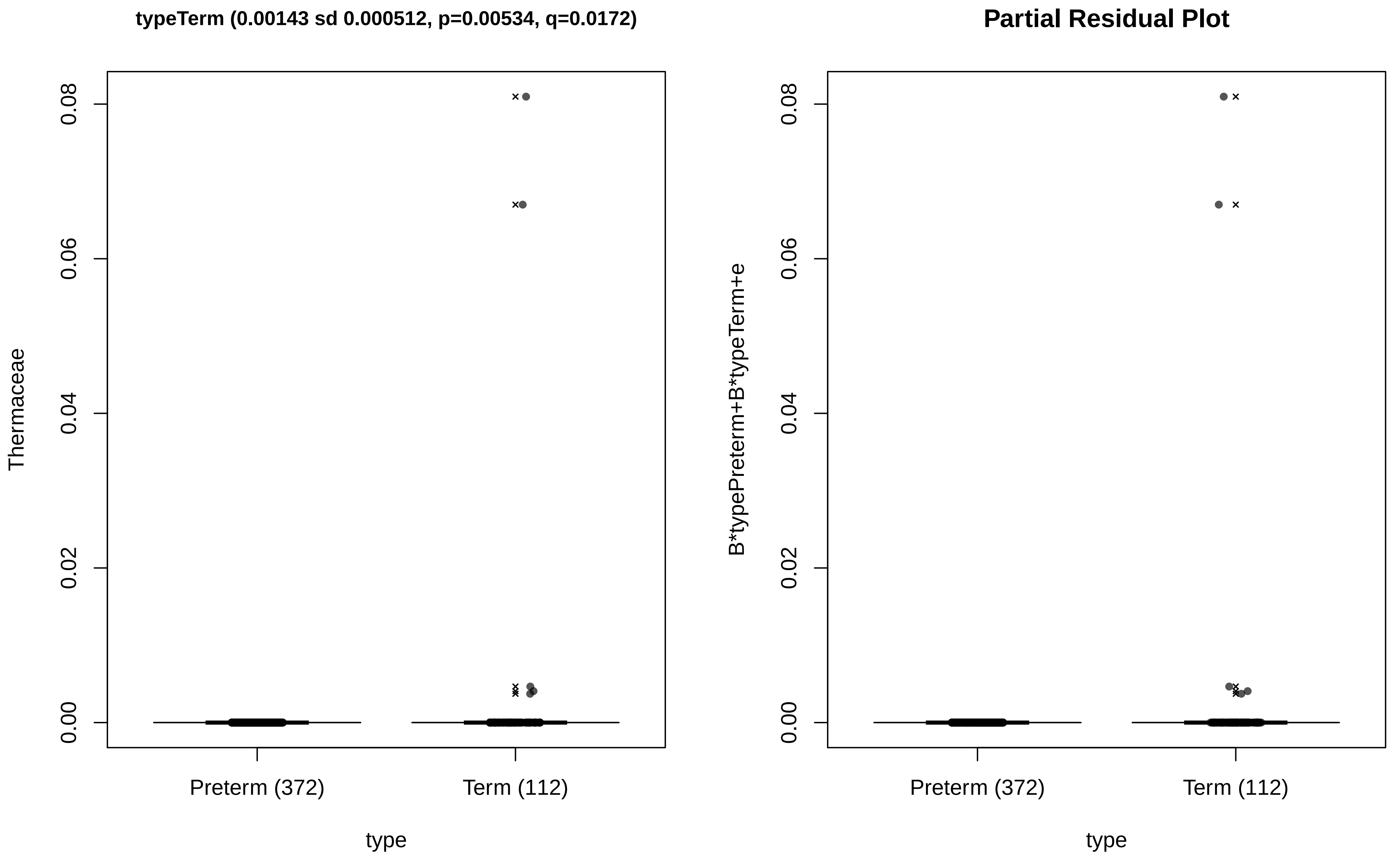

typeTerm (0.00143 sd 0.000512, p=0.00534, q=0.0172)
Thermaceae
0.00
0.02
0.04
0.06
0.08
Preterm (372)
Term (112)
type
Partial Residual Plot
B*typePreterm+B*typeTerm+e
0.00
0.02
0.04
0.06
0.08
Preterm (372)
Term (112)
type

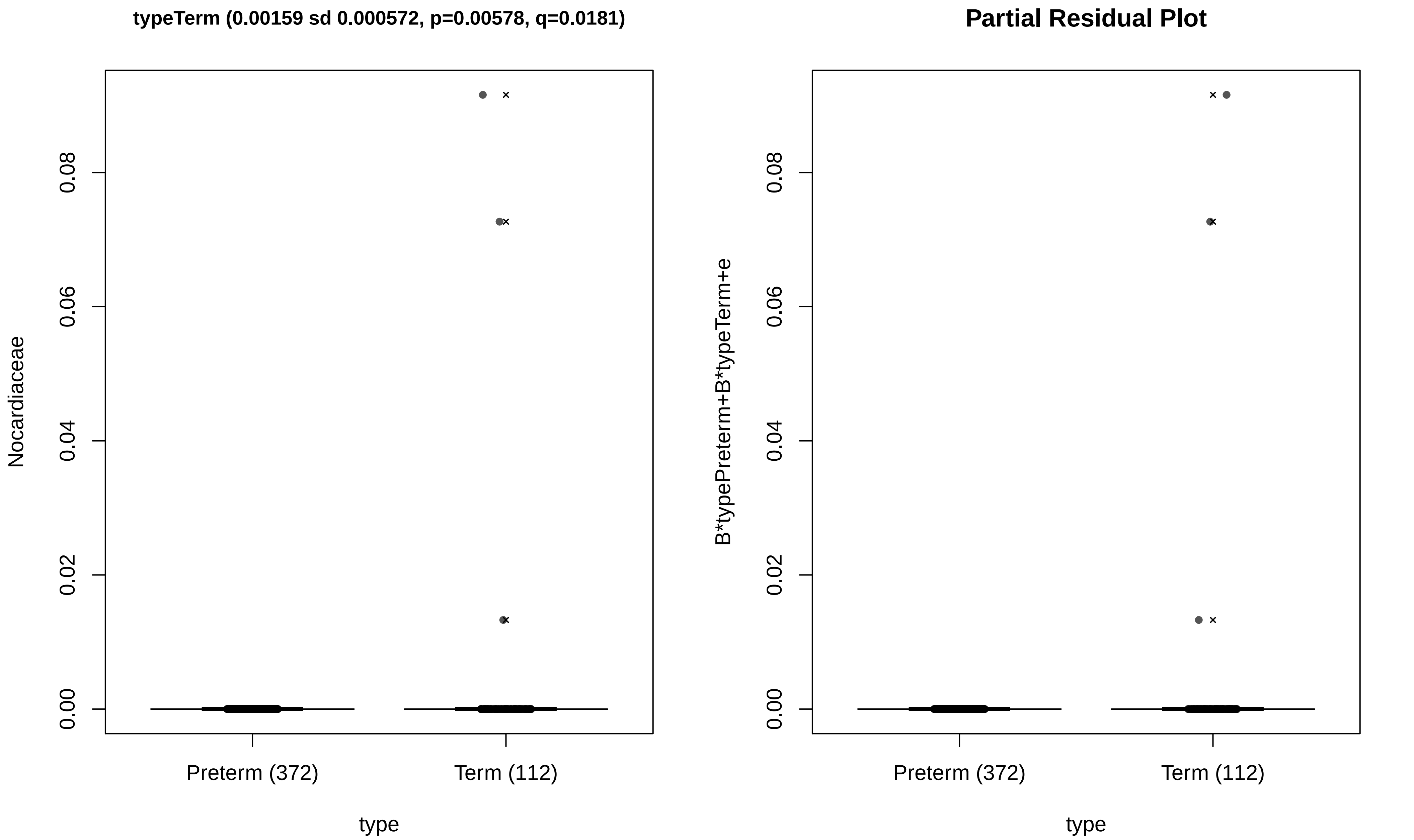
typeTerm (0.00159 sd 0.000572, p=0.00578, q=0.0181)
Nocardiaceae
0.00
0.02
0.04
0.06
0.08
Preterm (372)
Term (112)
type
Partial Residual Plot
B*typePreterm+B*typeTerm+e
0.00
0.02
0.04
0.06
0.08
Preterm (372)
Term (112)
type

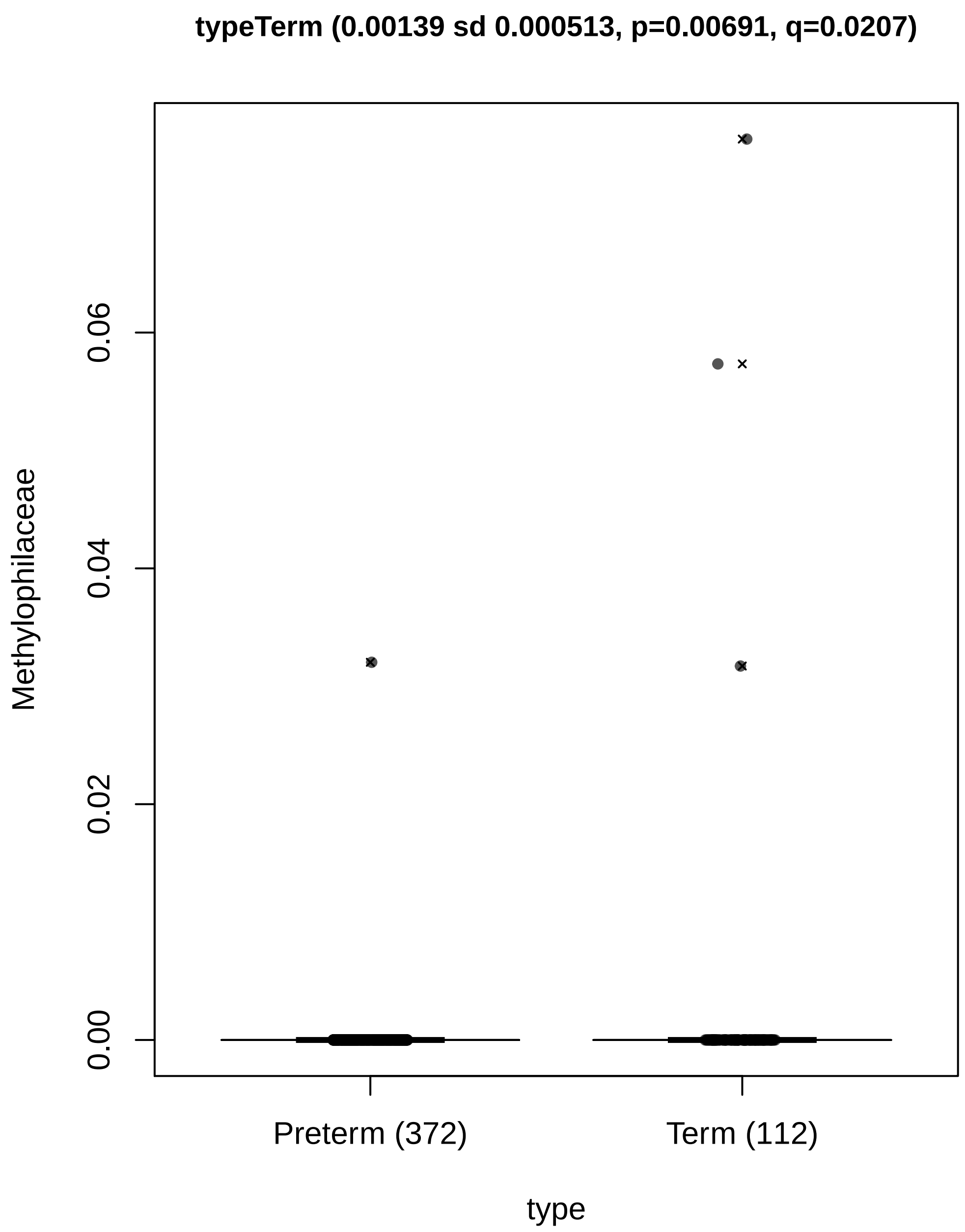

typeTerm (0.00139 sd 0.000513, p=0.00691, q=0.0207)
Methylophilaceae
0.00
0.02
0.04
0.06
Preterm (372)
Term (112)
type

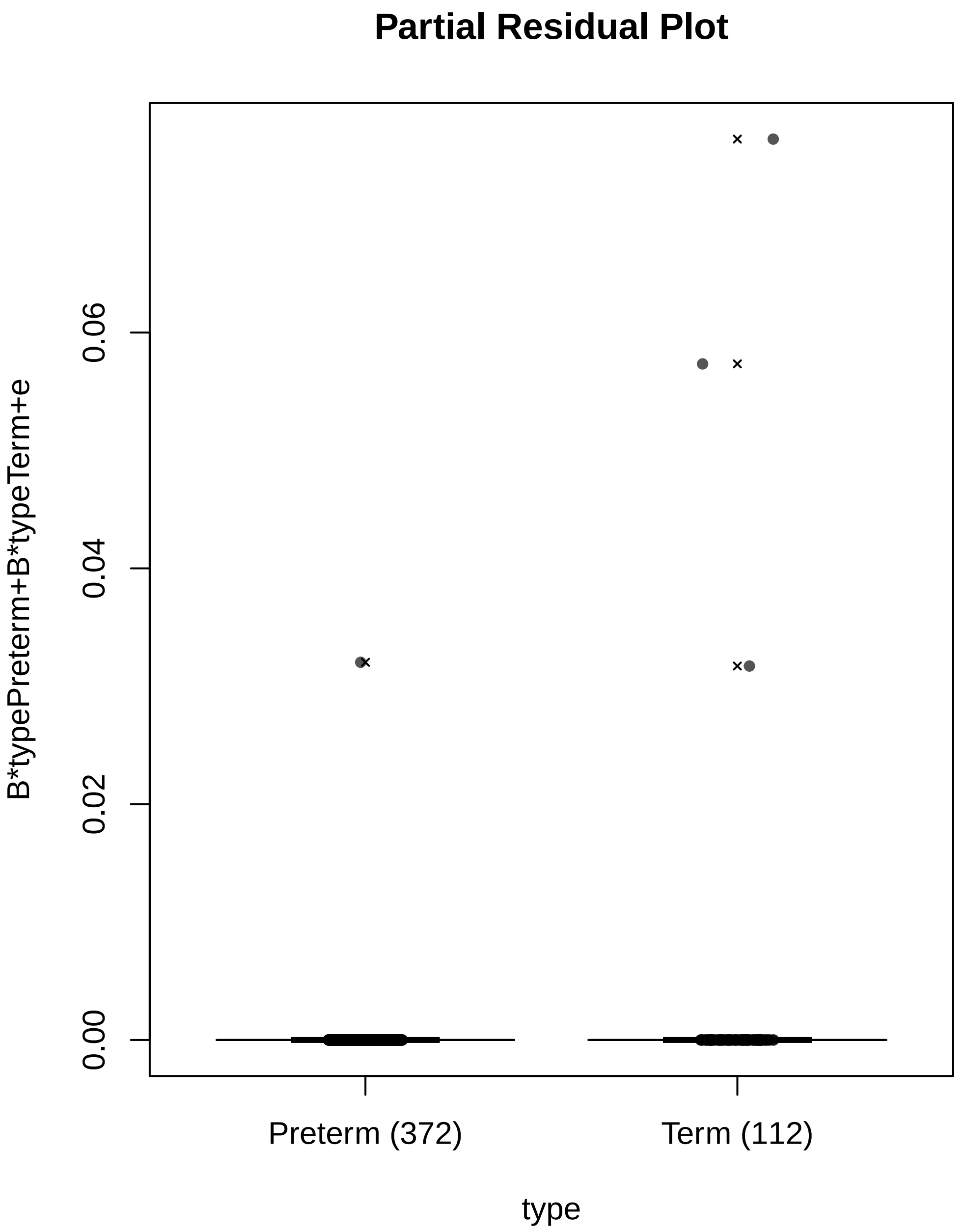

Partial Residual Plot
B*typePreterm+B*typeTerm+e
0.00
0.02
0.04
0.06
Preterm (372)
Term (112)
type

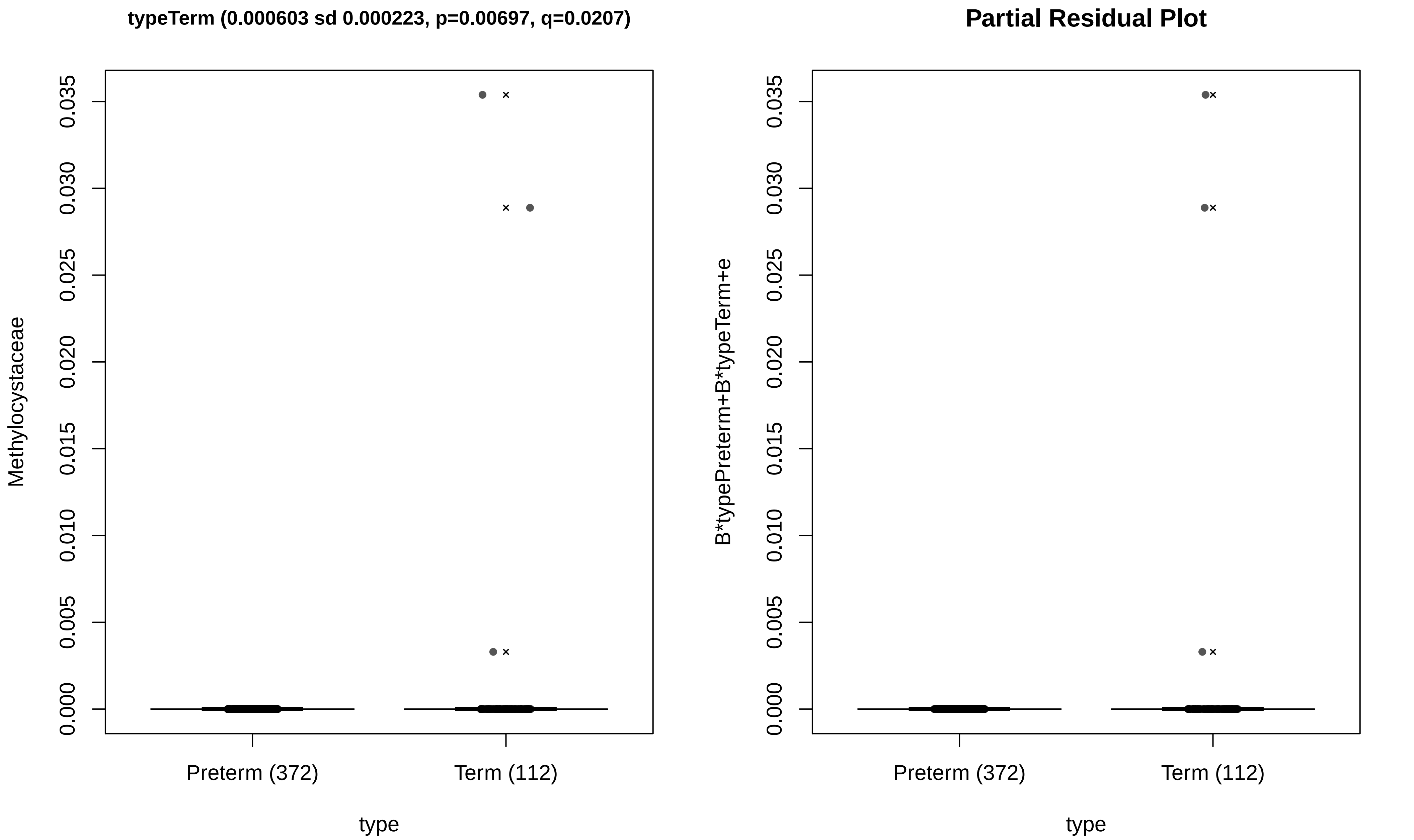

typeTerm (0.000603 sd 0.000223, p=0.00697, q=0.0207)
Methylocystaceae
0.000
0.005
0.010
0.015
0.020
0.025
0.030
0.035
Preterm (372)
Term (112)
type
Partial Residual Plot
B*typePreterm+B*typeTerm+e
0.000
0.005
0.010
0.015
0.020
0.025
0.030
0.035
Preterm (372)
Term (112)
type

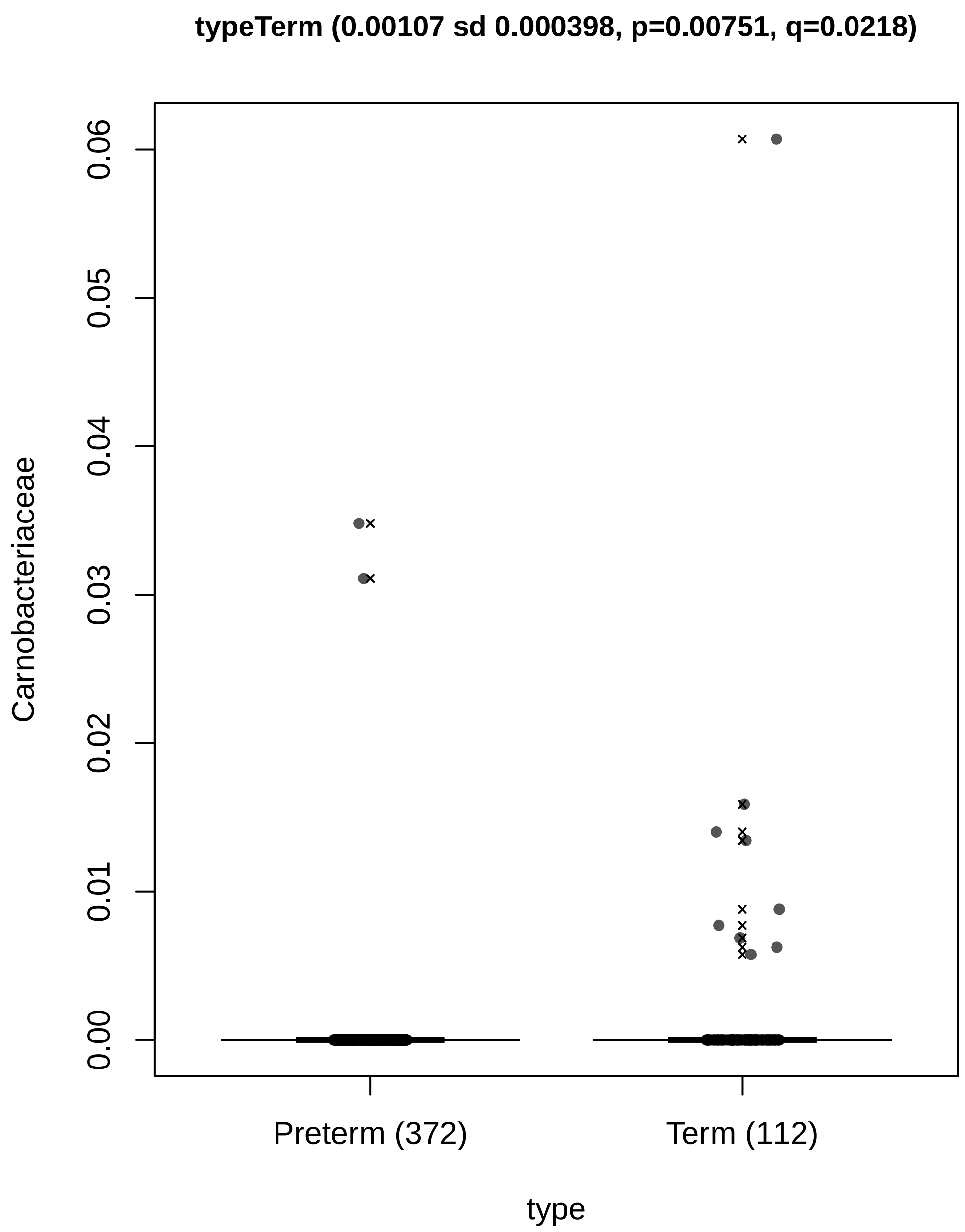

typeTerm (0.00107 sd 0.000398, p=0.00751, q=0.0218)
Carnobacteriaceae
0.00
0.01
0.02
0.03
0.04
0.05
0.06
Preterm (372)
Term (112)
type

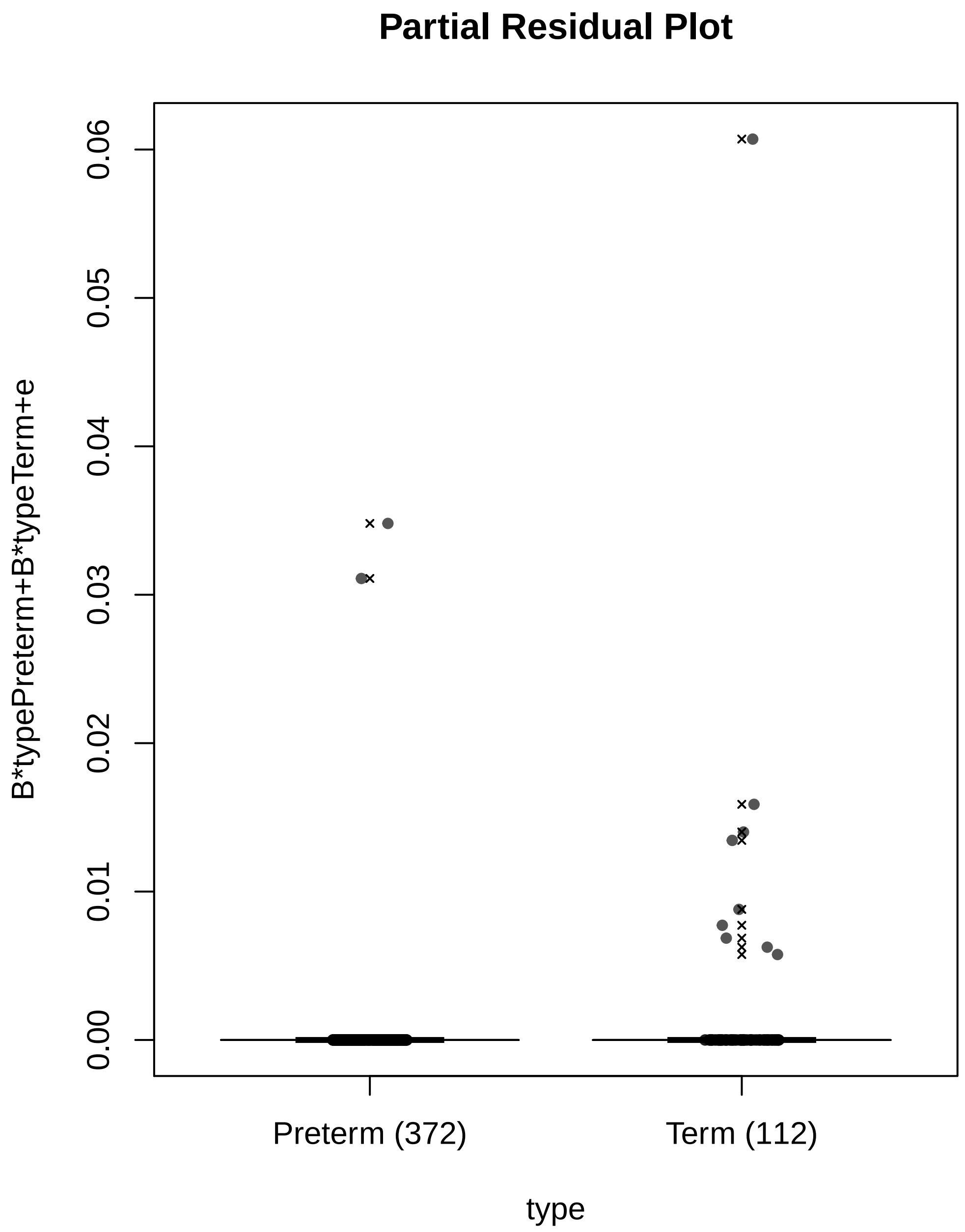

Partial Residual Plot
B*typePreterm+B*typeTerm+e
0.00
0.01
0.02
0.03
0.04
0.05
0.06
Preterm (372)
Term (112)
type

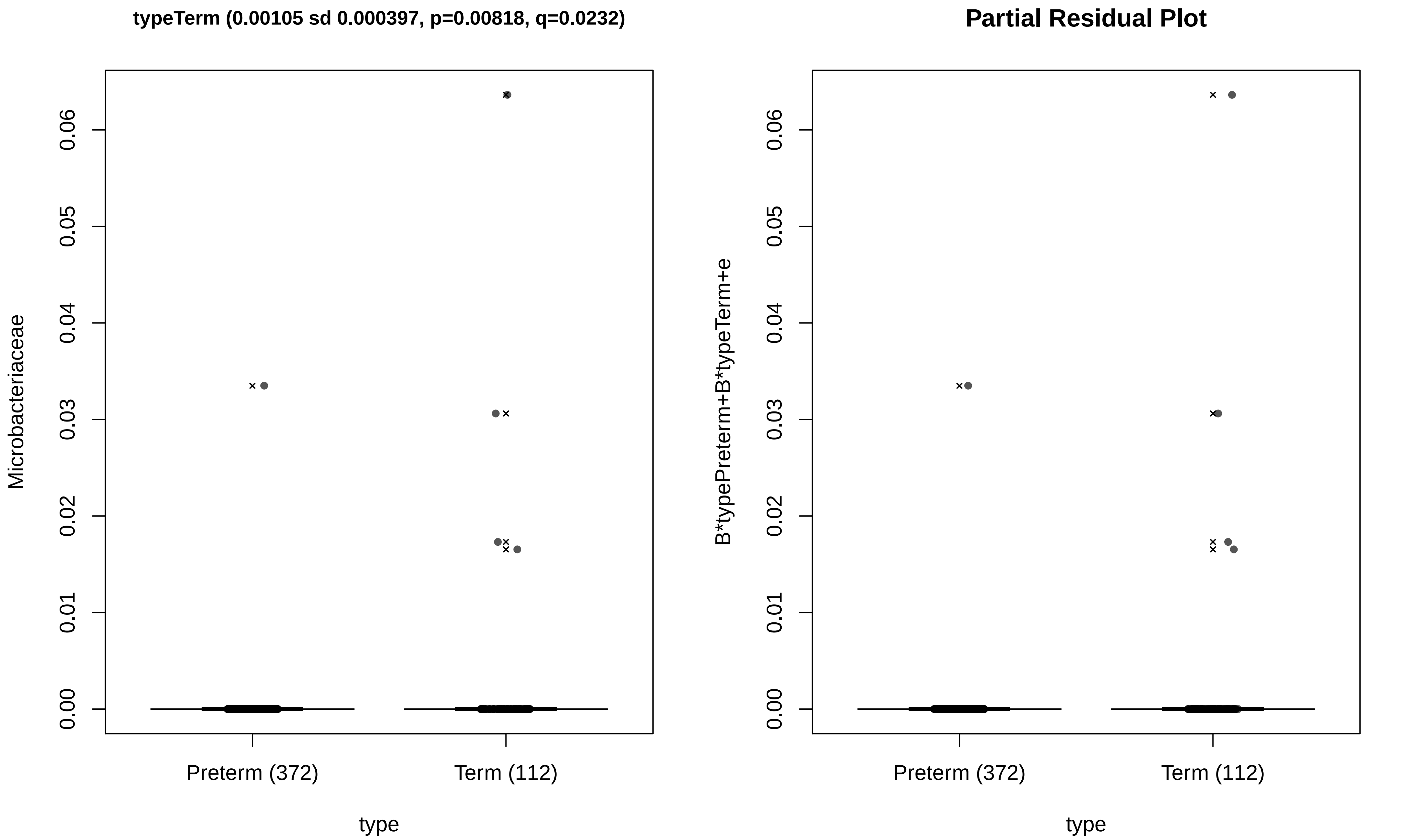

typeTerm (0.00105 sd 0.000397, p=0.00818, q=0.0232)
Partial Residual Plot
Microbacteriaceae
B*typePreterm+B*typeTerm+e
0.00
0.01
0.02
0.03
0.04
0.05
0.06
Preterm (372)
Term (112)
type

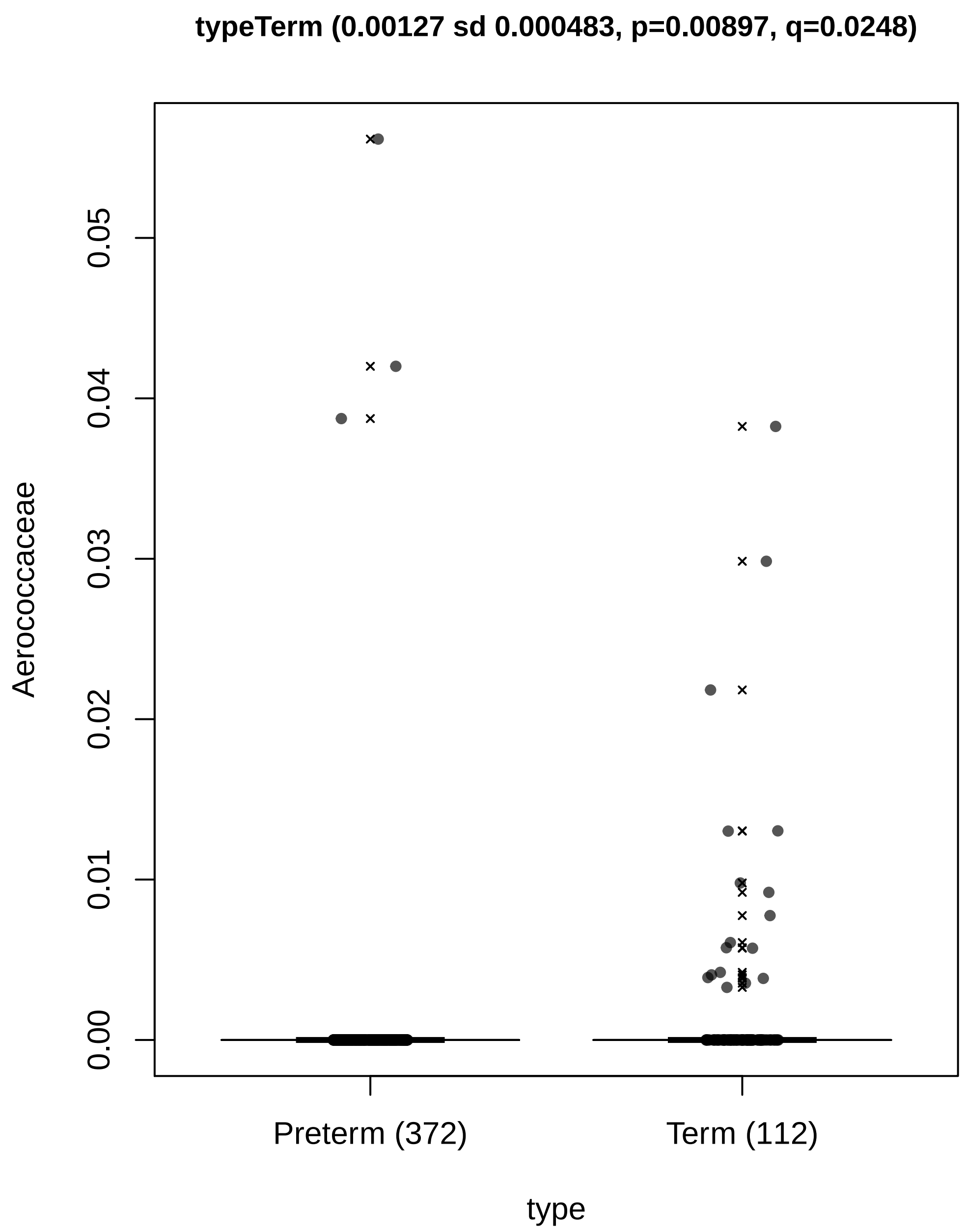
typeTerm (0.00127 sd 0.000483, p=0.00897, q=0.0248)
Aerococcaceae
0.00
0.01
0.02
0.03
0.04
0.05
Preterm (372)
Term (112)
type

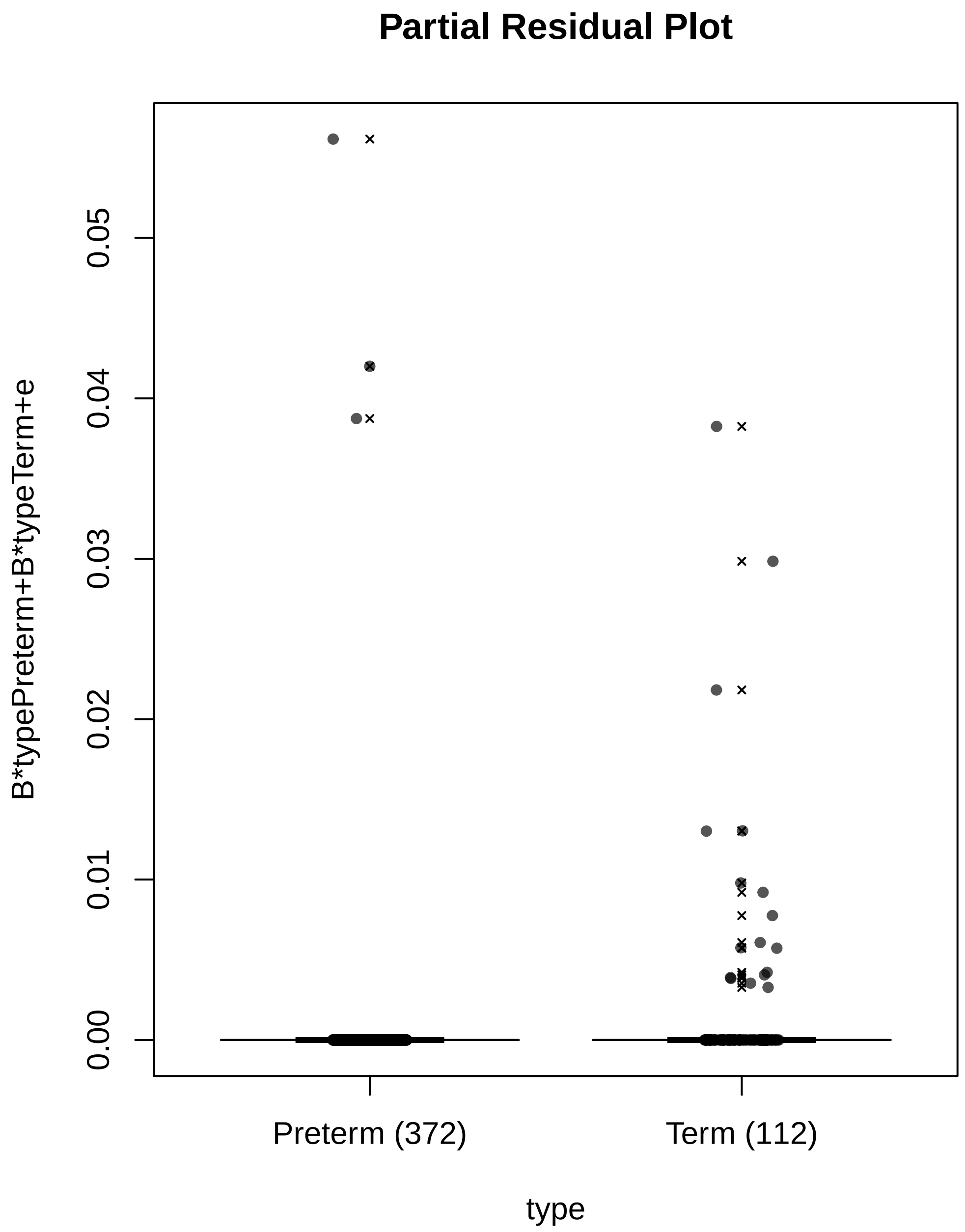
Partial Residual Plot
B*typePreterm+B*typeTerm+e
0.00
0.01
0.02
0.03
0.04
0.05
Preterm (372)
Term (112)
type

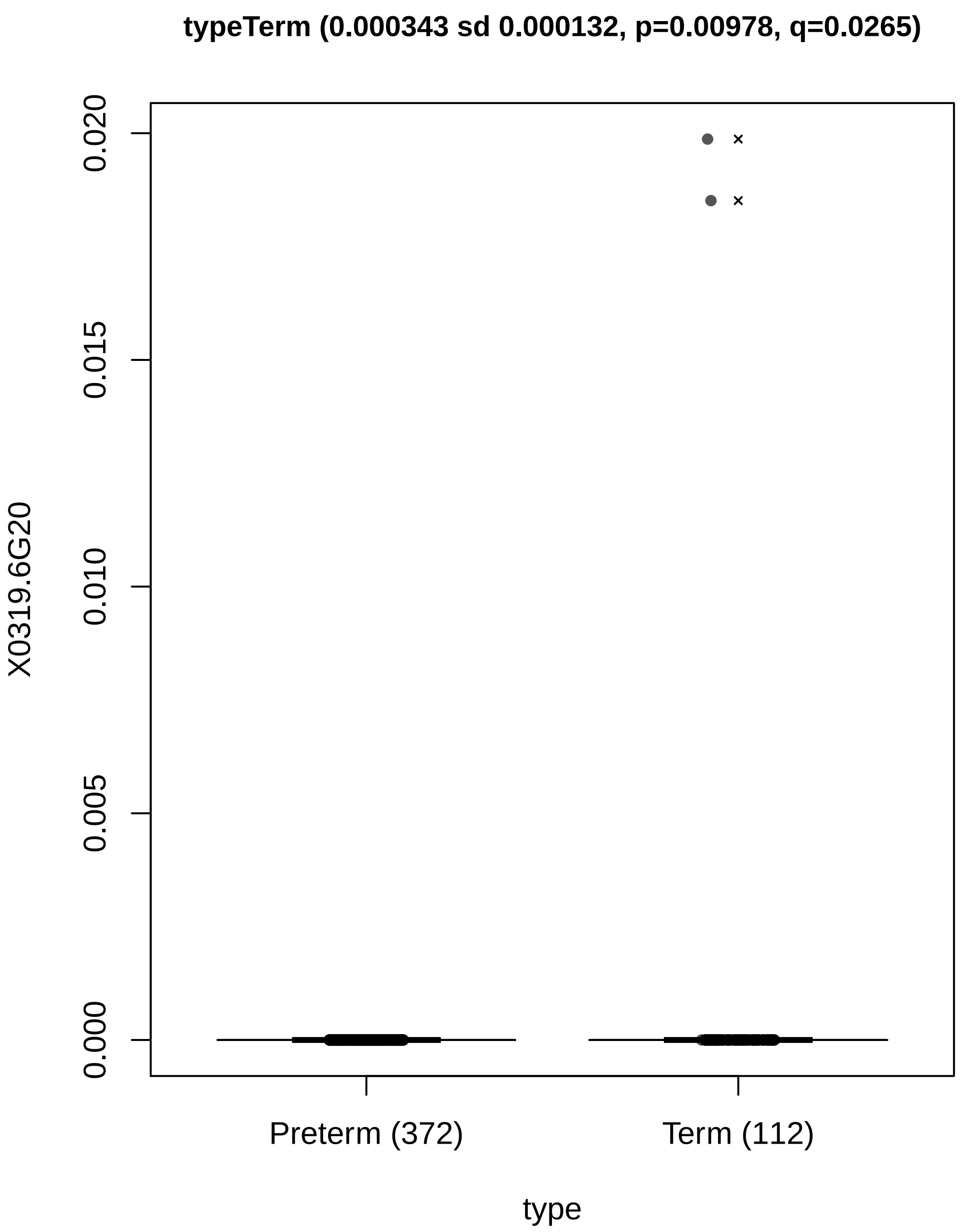
typeTerm (0.000343 sd 0.000132, p=0.00978, q=0.0265)
X0319.6G20
0.020
0.015
0.010
0.005
0.000
Preterm (372)
Term (112)
type

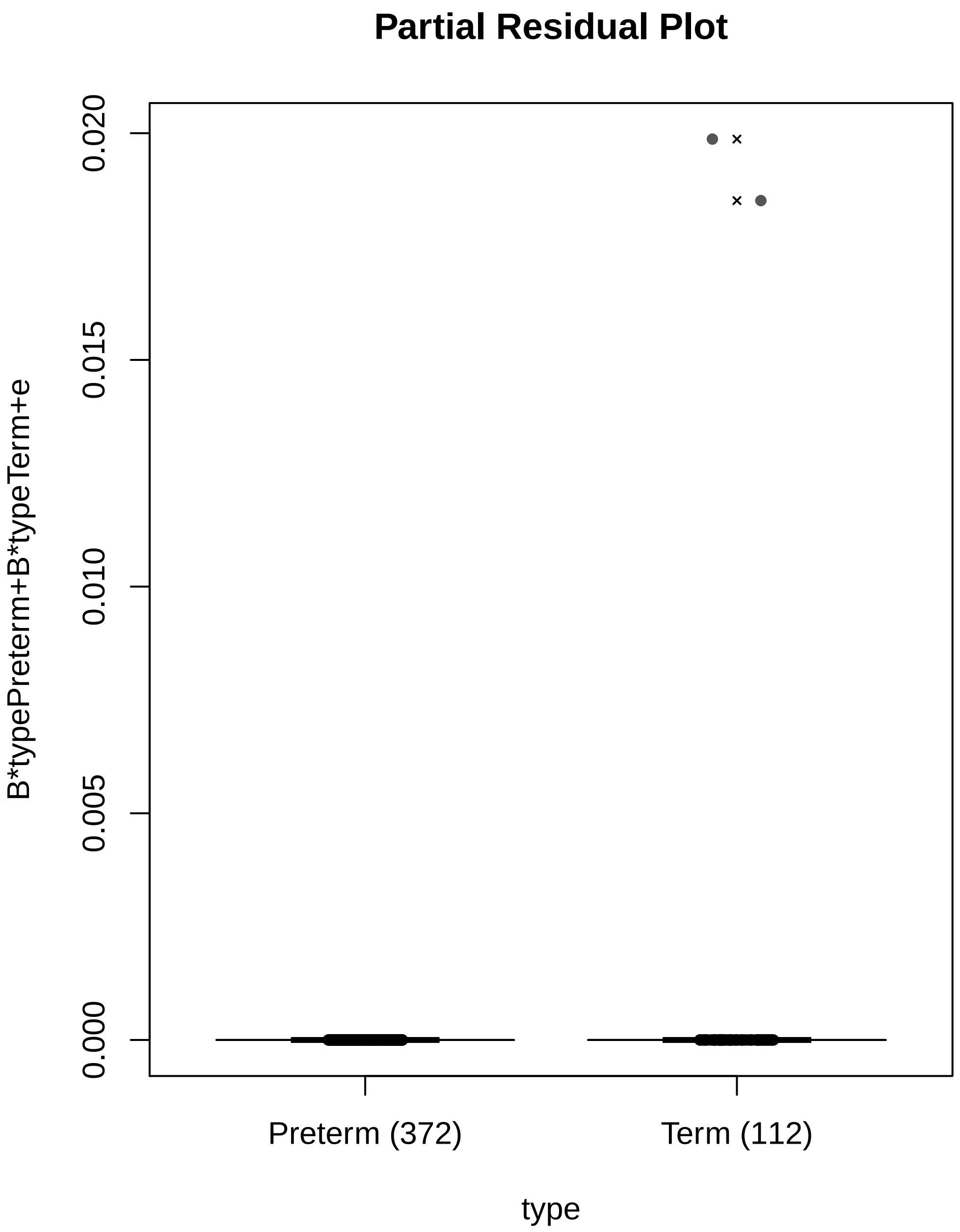
Partial Residual Plot
B*typePreterm+B*typeTerm+e
0.020
0.015
0.010
0.005
0.000
Preterm (372)
Term (112)
type

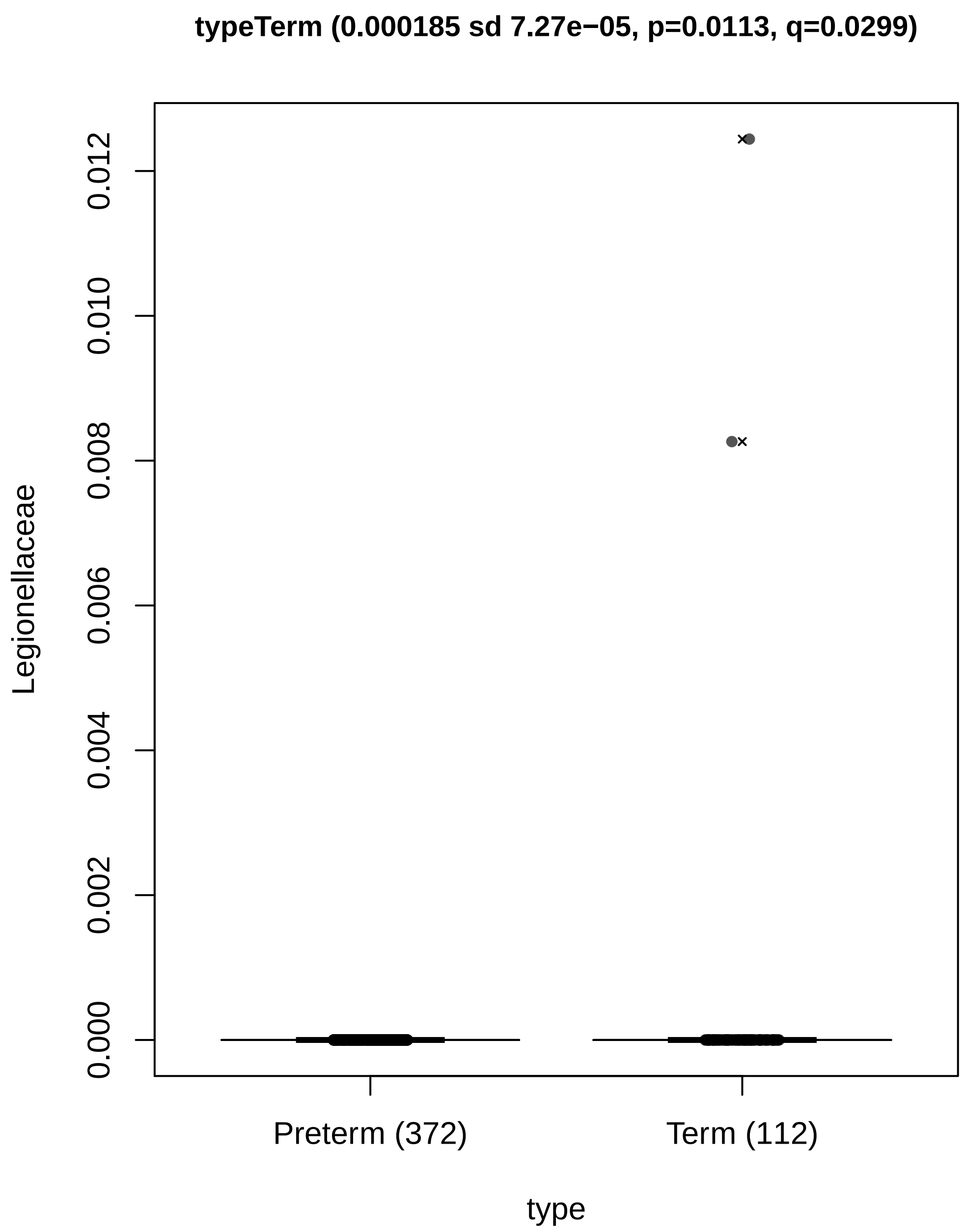

typeTerm (0.000185 sd 7.27e−05, p=0.0113, q=0.0299)
Legionellaceae
0.000
0.002
0.004
0.006
0.008
0.010
0.012
Preterm (372)
Term (112)
type

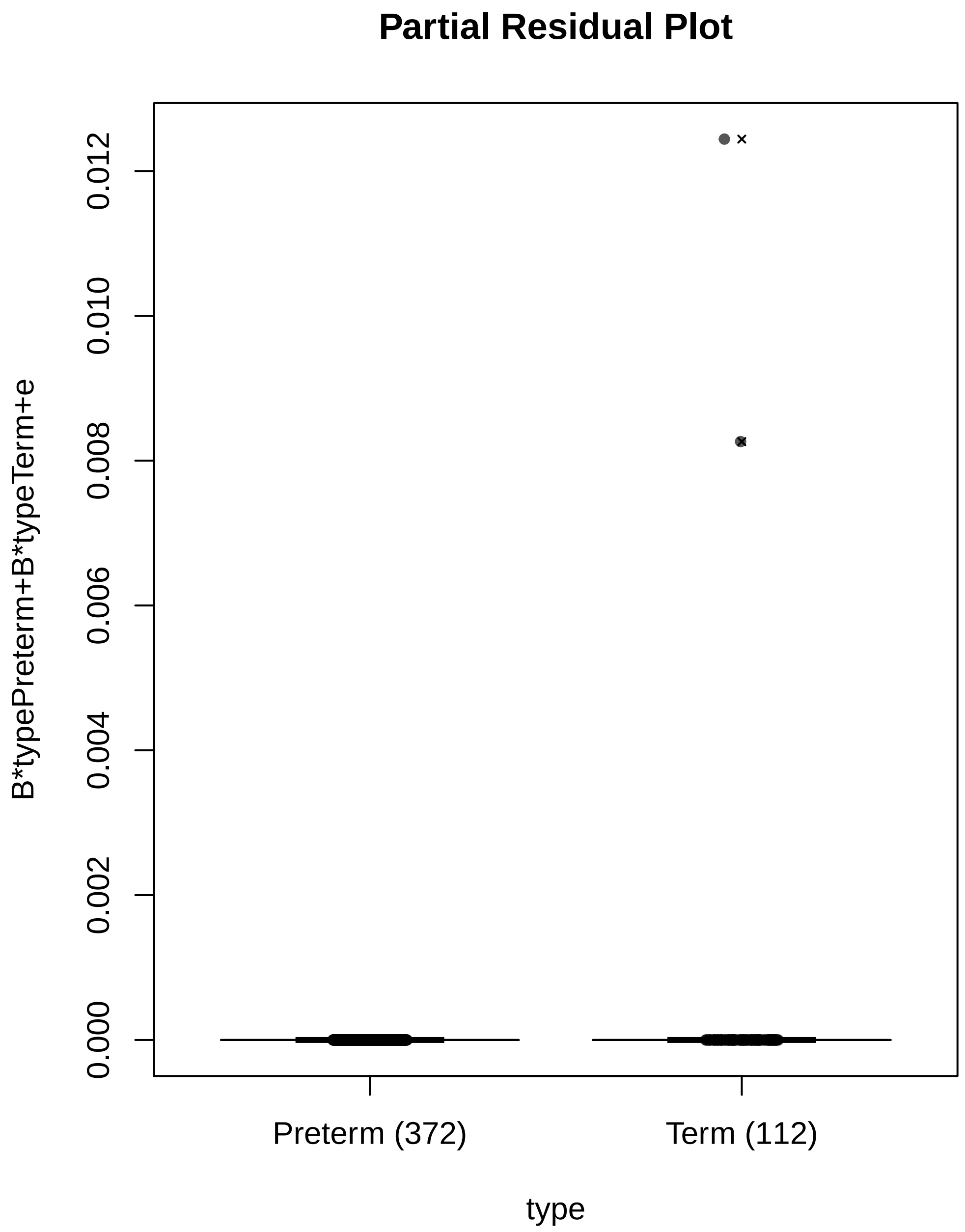

Partial Residual Plot
B*typePreterm+B*typeTerm+e
0.000
0.002
0.004
0.006
0.008
0.010
0.012
Preterm (372)
Term (112)
type

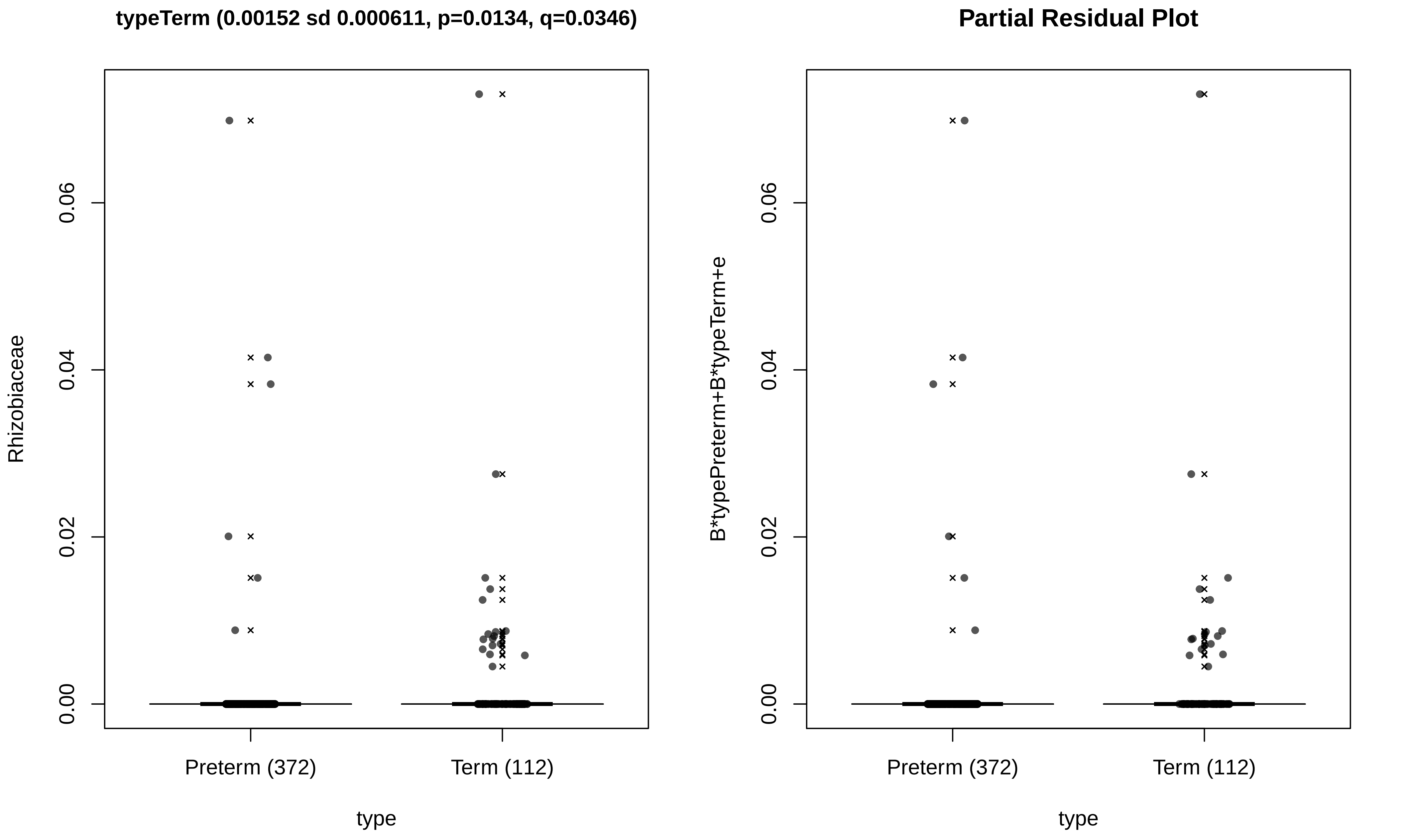
typeTerm (0.00152 sd 0.000611, p=0.0134, q=0.0346)
Rhizobiaceae
0.00
0.02
0.04
0.06
Preterm (372)
Term (112)
type
Partial Residual Plot
B*typePreterm+B*typeTerm+e
0.00
0.02
0.04
0.06
Preterm (372)
Term (112)
type

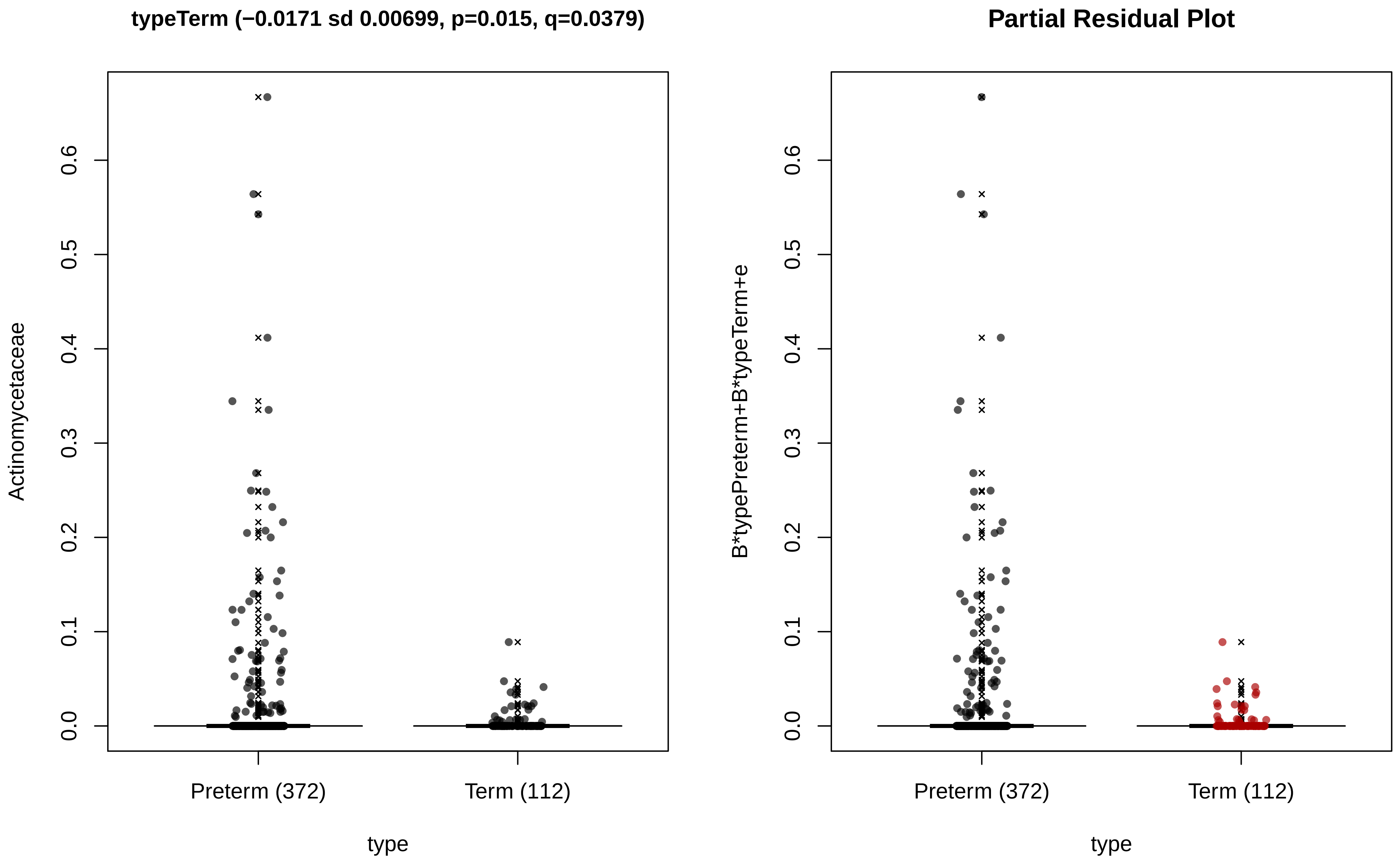

typeTerm (−0.0171 sd 0.00699, p=0.015, q=0.0379)
Actinomycetaceae
0.0
0.1
0.2
0.3
0.4
0.5
0.6
Preterm (372)
Term (112)
type
Partial Residual Plot
B*typePreterm+B*typeTerm+e
0.0
0.1
0.2
0.3
0.4
0.5
0.6
Preterm (372)
Term (112)
type

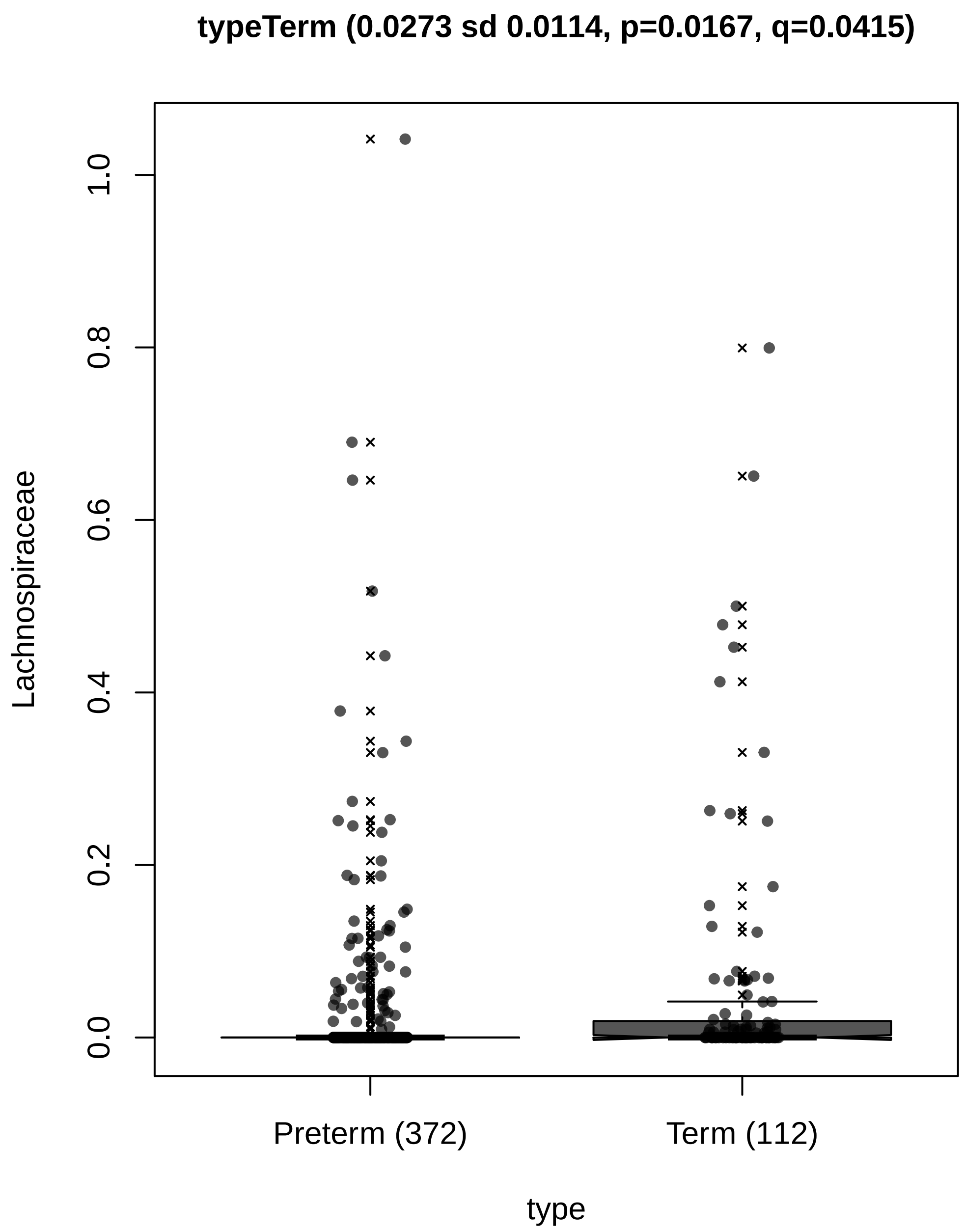
typeTerm (0.0273 sd 0.0114, p=0.0167, q=0.0415)
Lachnospiraceae
0.0
0.2
0.4
0.6
0.8
1.0
Preterm (372)
Term (112)
type

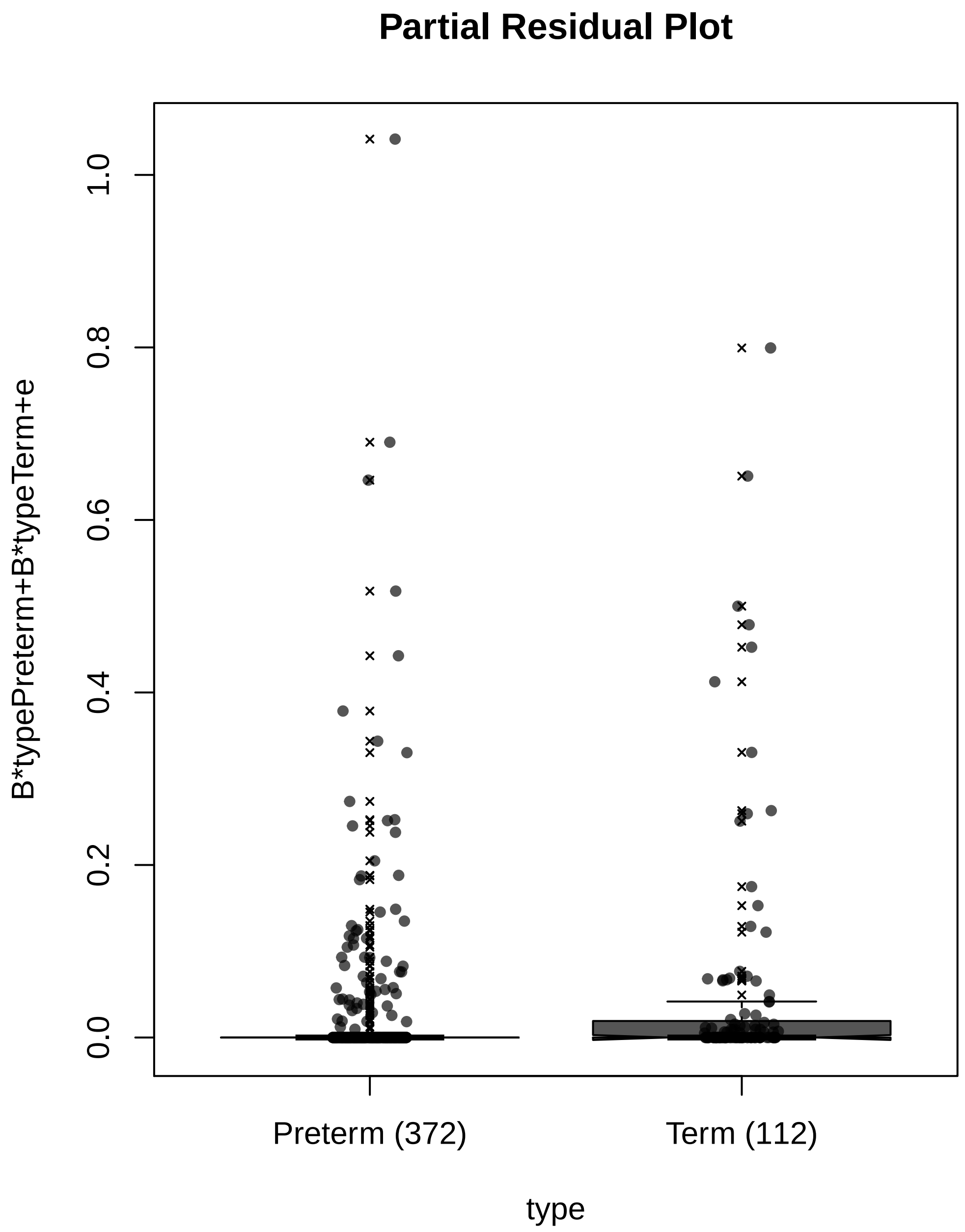
Partial Residual Plot
B*typePreterm+B*typeTerm+e
0.0
0.2
0.4
0.6
0.8
1.0
Preterm (372)
Term (112)
type

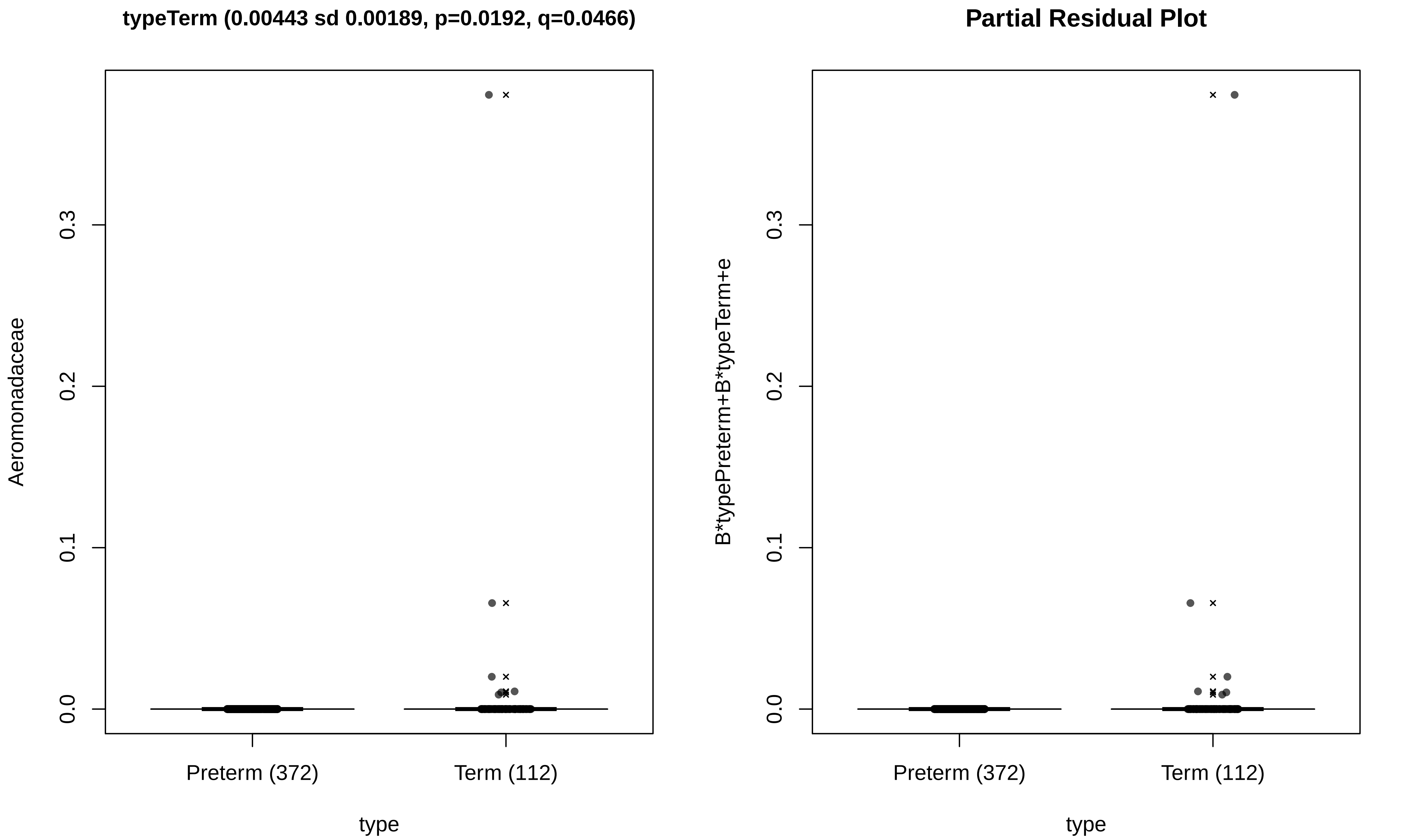
typeTerm (0.00443 sd 0.00189, p=0.0192, q=0.0466)
Aeromonadaceae
0.0
0.1
0.2
0.3
Preterm (372)
Term (112)
type
Partial Residual Plot
B*typePreterm+B*typeTerm+e
0.0
0.1
0.2
0.3
Preterm (372)
Term (112)
type
