# Supplementary material for: Predicted Metabolic Pathway Distributions in Stool Bacteria in Very-Low-Birth-Weight Infants: Potential Relationships with NICU Faltered Growth
Source: Nutrients. 2020 May 8;12(5):1345. doi: 10.3390/nu12051345 (PMC7284701; doi:10.3390/nu12051345)
Supplement: Supplementary file 1 [file nutrients-12-01345-s001.zip › nutrients-769405-supplementary/Supplementary Meterials/S4_Supplementary_data.pdf]

Supplementary data

Metabolic Pathway Distributions in Very Low Birth Weight Infants: Potential Relationships with NICU Faltered Growth

S4. Association of the relative abundance of bacterial KEGG pathways to the VLBW infants and term infants. Each page of this file shows a separate pathway which was significantly different between the two groups. The box plots indicate the association of the metadata (here, the two groups preterm and term infants) with the relative abundances. The strength of association is indicated by the p-value and an FDR-corrected q-value. The partial residual plot shows the relationship between the groups with abundances when all other variables are included in the model.

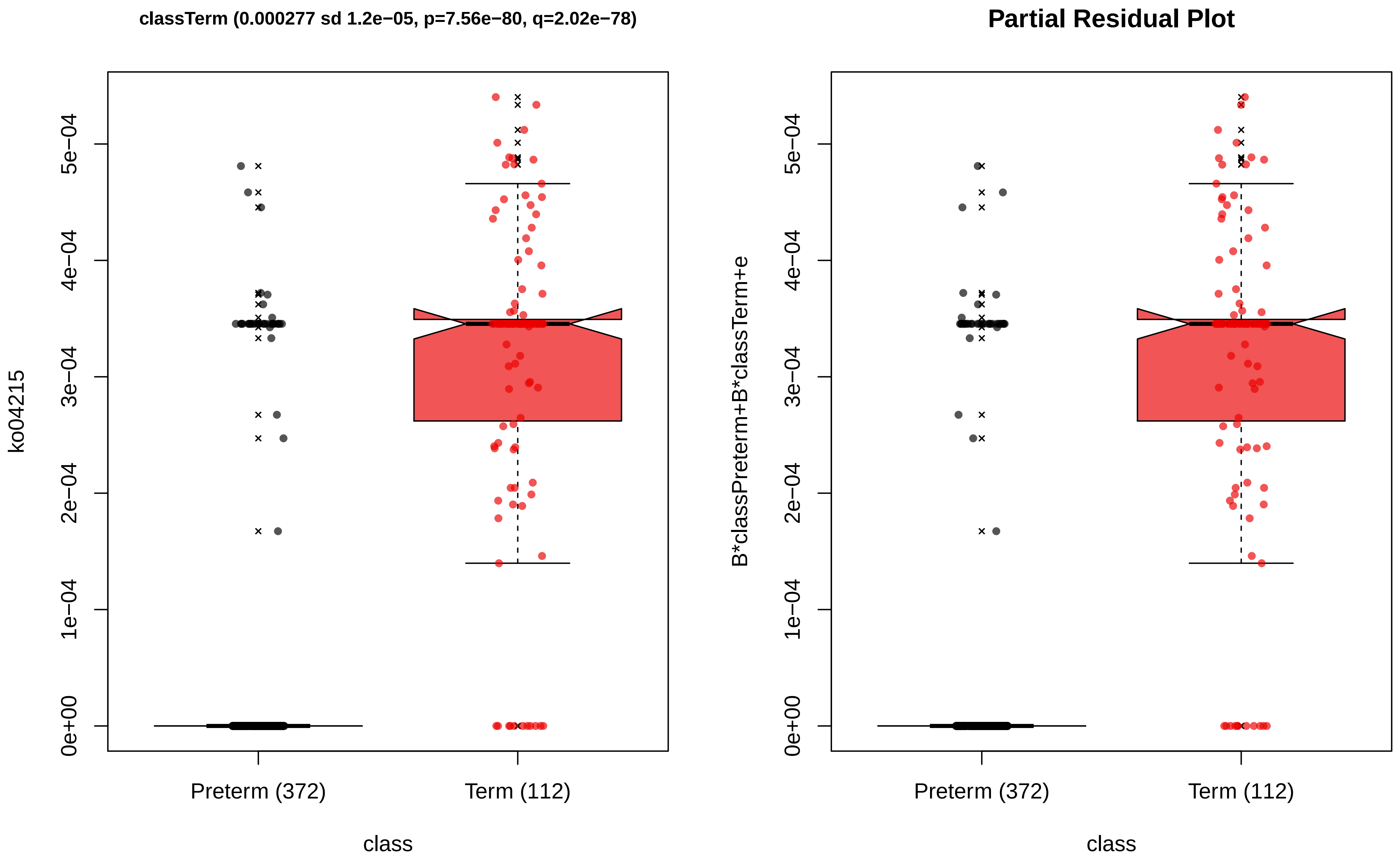

classTerm (0.000277 sd 1.2e−05, p=7.56e−80, q=2.02e−78)
ko04215
0e+00
1e−04
2e−04
3e−04
4e−04
5e−04
Preterm (372)
Term (112)
class
Partial Residual Plot
B*classPreterm+B*classTerm+e
0e+00
1e−04
2e−04
3e−04
4e−04
5e−04
Preterm (372)
Term (112)
class

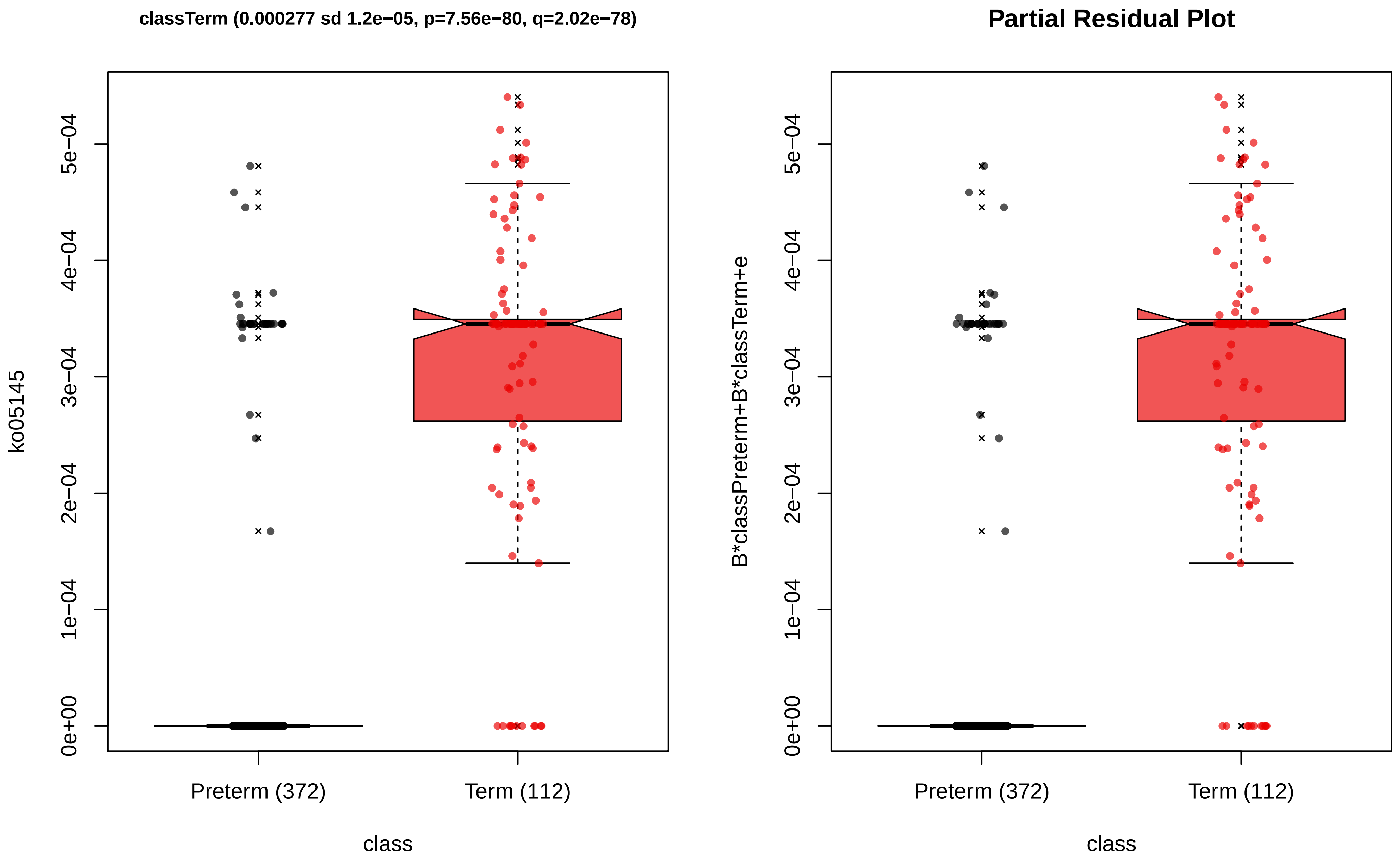

classTerm (0.000277 sd 1.2e−05, p=7.56e−80, q=2.02e−78)
ko05145
0e+00
1e−04
2e−04
3e−04
4e−04
5e−04
Preterm (372)
Term (112)
class
Partial Residual Plot
B*classPreterm+B*classTerm+e
0e+00
1e−04
2e−04
3e−04
4e−04
5e−04
Preterm (372)
Term (112)
class

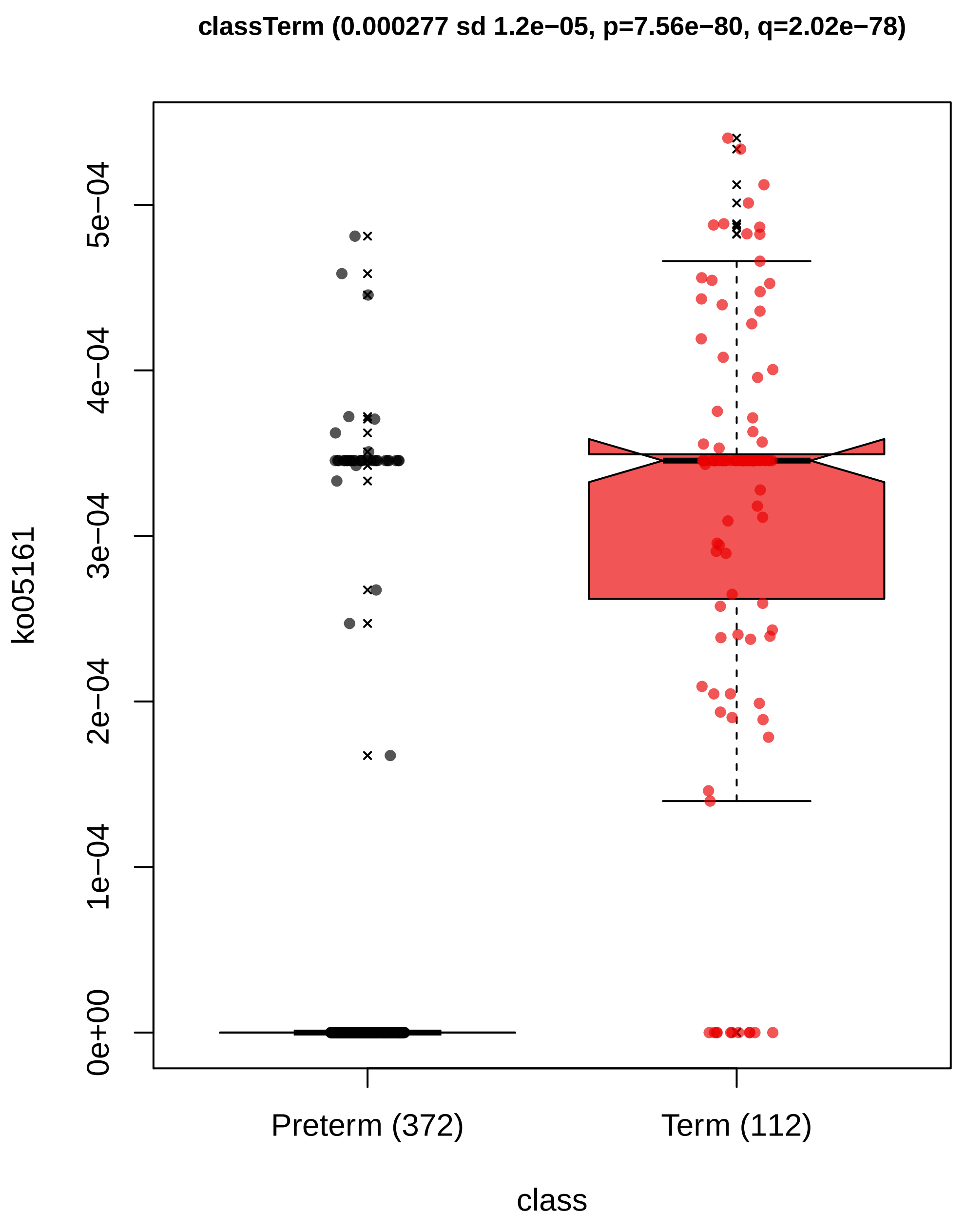

classTerm (0.000277 sd 1.2e−05, p=7.56e−80, q=2.02e−78)
ko05161
0e+00
1e−04
2e−04
3e−04
4e−04
5e−04
Preterm (372)
Term (112)
class

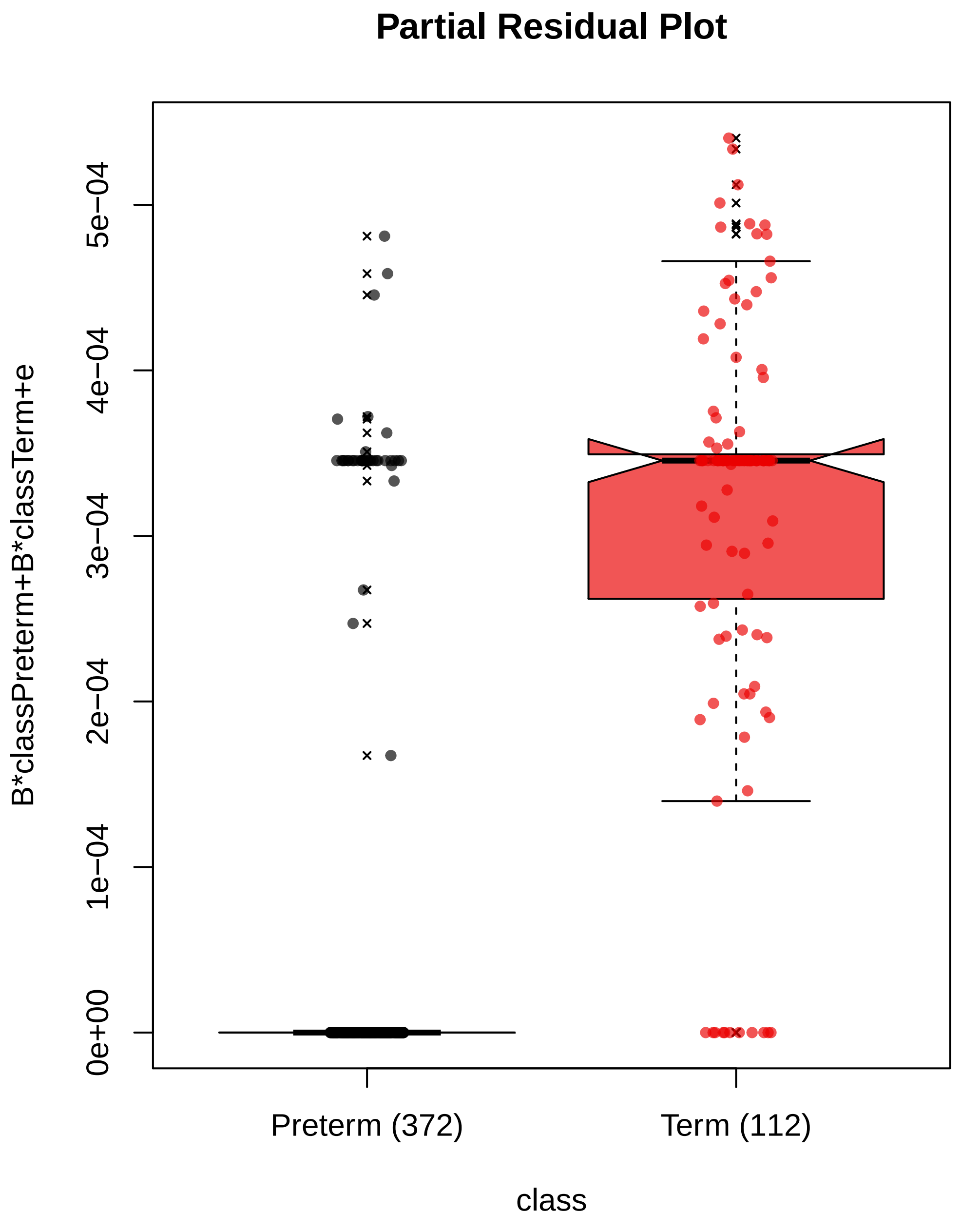

Partial Residual Plot
B*classPreterm+B*classTerm+e
0e+00
1e−04
2e−04
3e−04
4e−04
5e−04
Preterm (372)
Term (112)
class

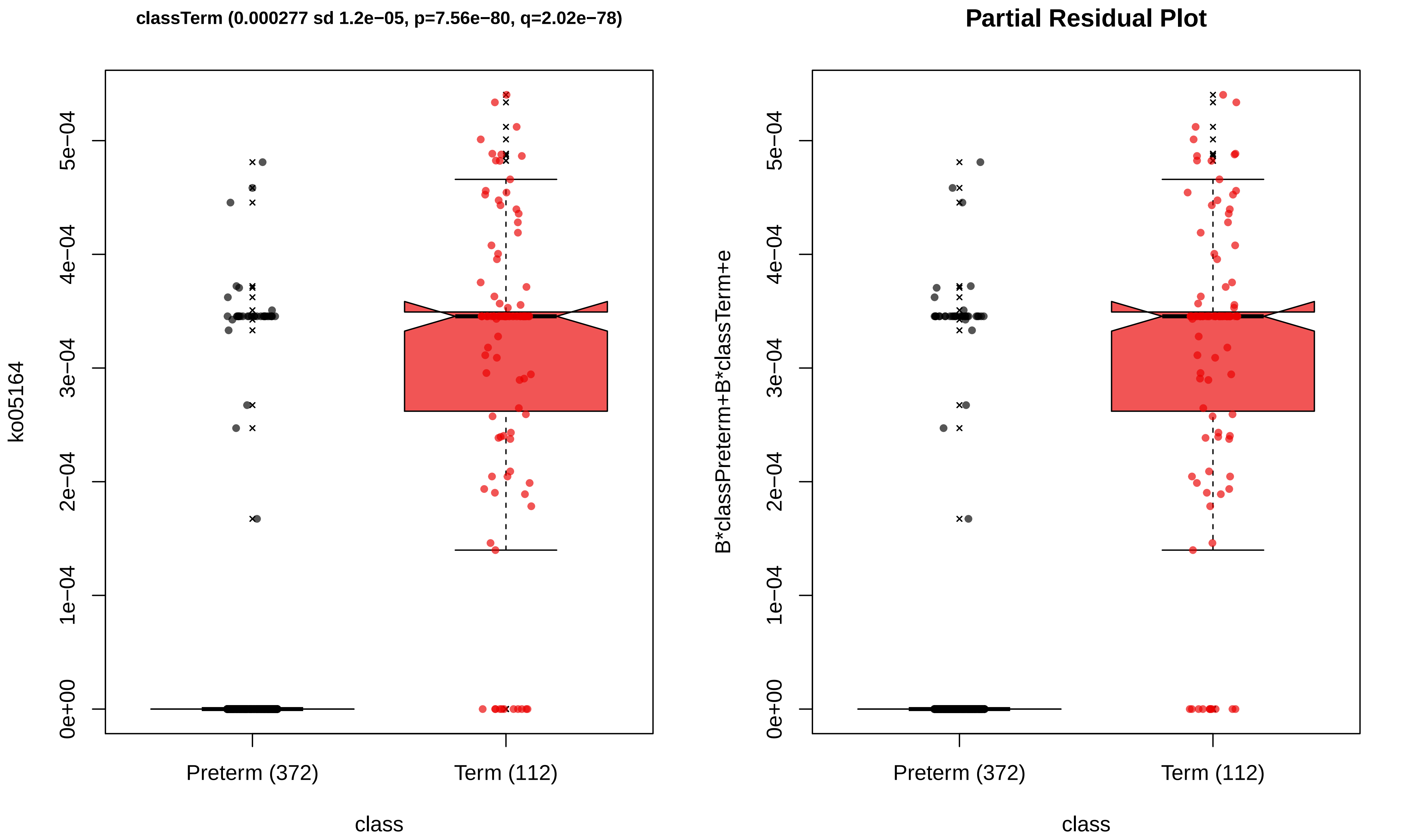
classTerm (0.000277 sd 1.2e−05, p=7.56e−80, q=2.02e−78)
ko05164
0e+00
1e−04
2e−04
3e−04
4e−04
5e−04
Preterm (372)
Term (112)
class
Partial Residual Plot
B*classPreterm+B*classTerm+e
0e+00
1e−04
2e−04
3e−04
4e−04
5e−04
Preterm (372)
Term (112)
class

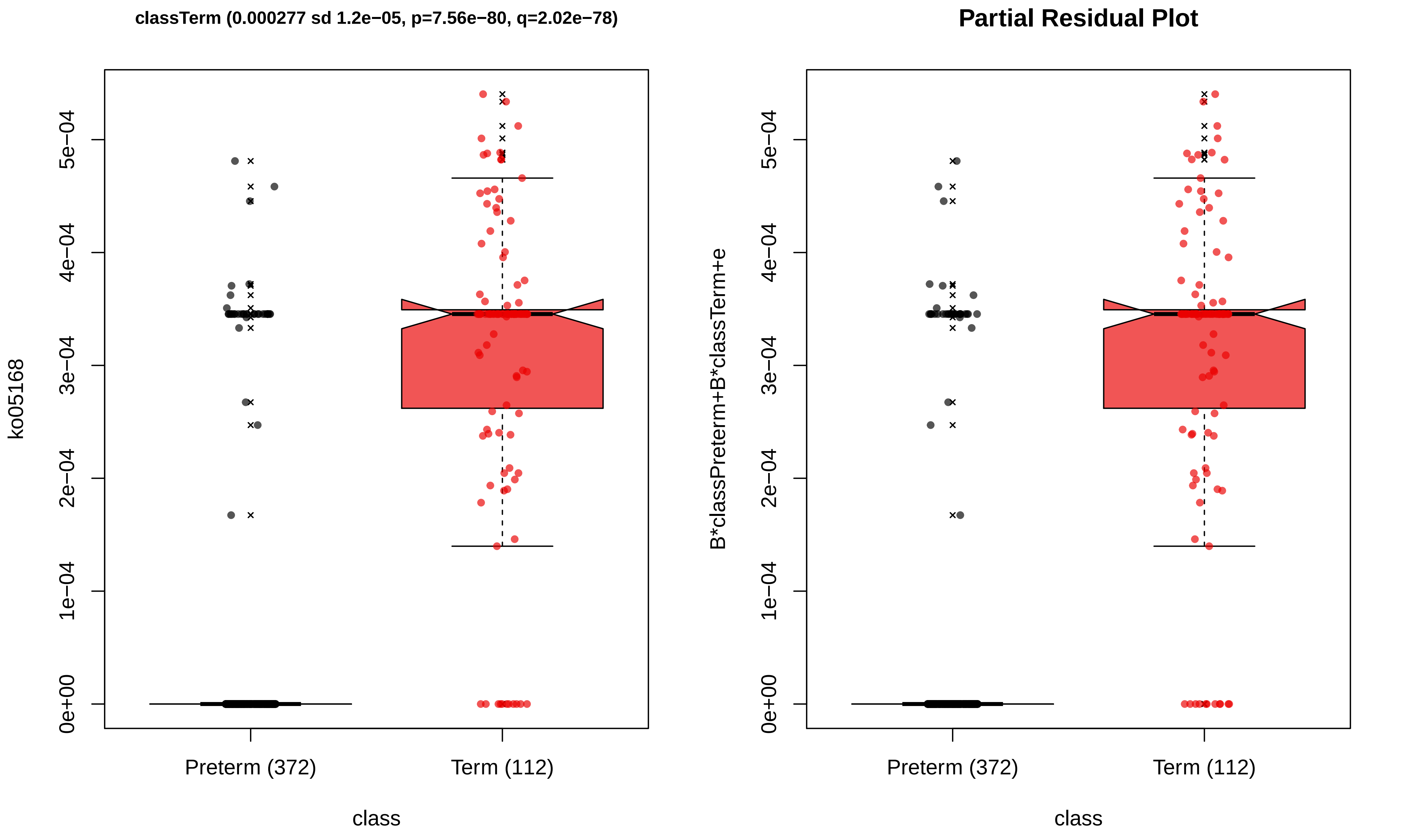
classTerm (0.000277 sd 1.2e−05, p=7.56e−80, q=2.02e−78)
Partial Residual Plot
ko05168
B*classPreterm+B*classTerm+e
0e+00
1e−04
2e−04
3e−04
4e−04
5e−04
Preterm (372)
Term (112)
class

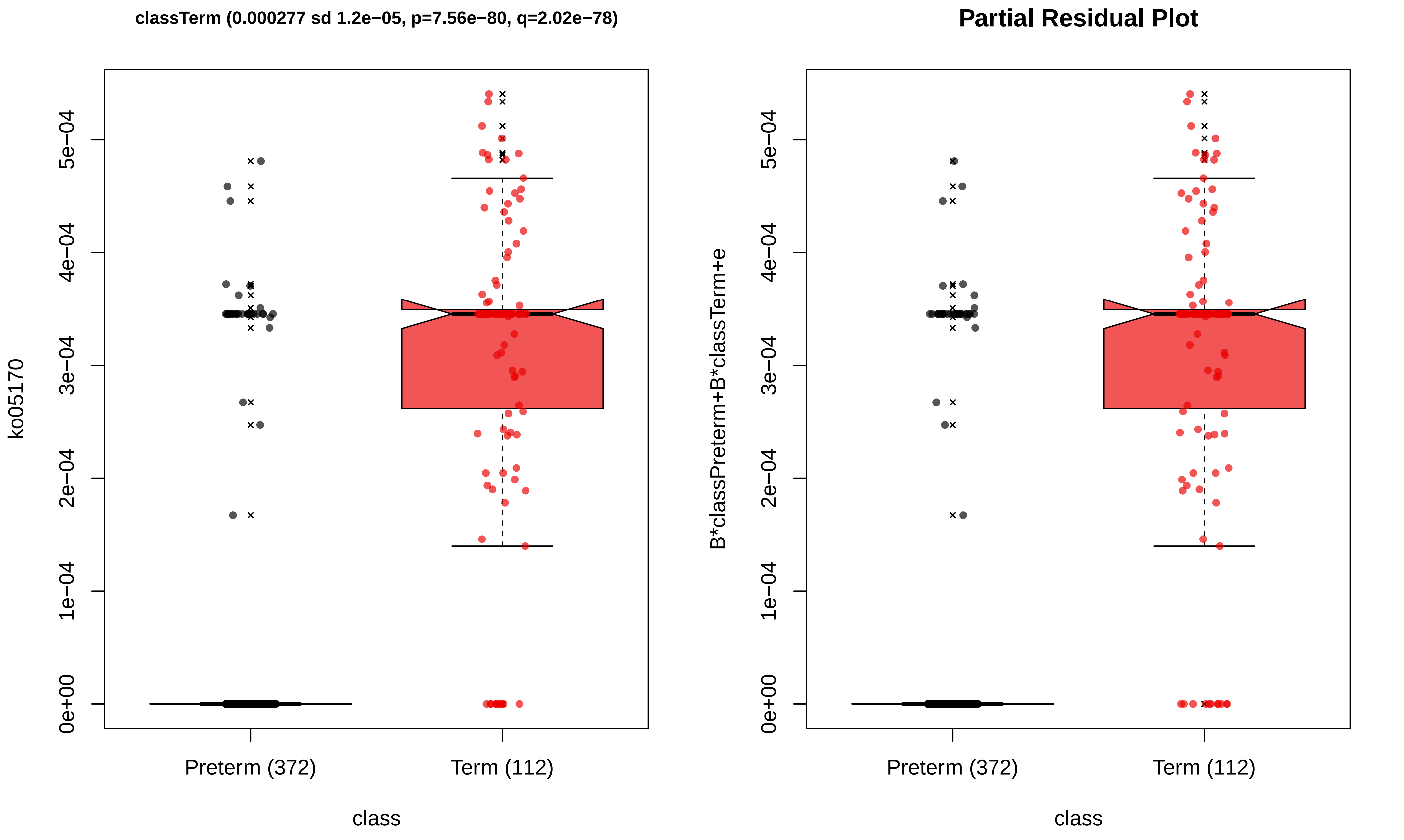

classTerm (0.000277 sd 1.2e−05, p=7.56e−80, q=2.02e−78)
ko05170
0e+00
1e−04
2e−04
3e−04
4e−04
5e−04
Preterm (372)
Term (112)
class
Partial Residual Plot
B*classPreterm+B*classTerm+e
0e+00
1e−04
2e−04
3e−04
4e−04
5e−04
Preterm (372)
Term (112)
class

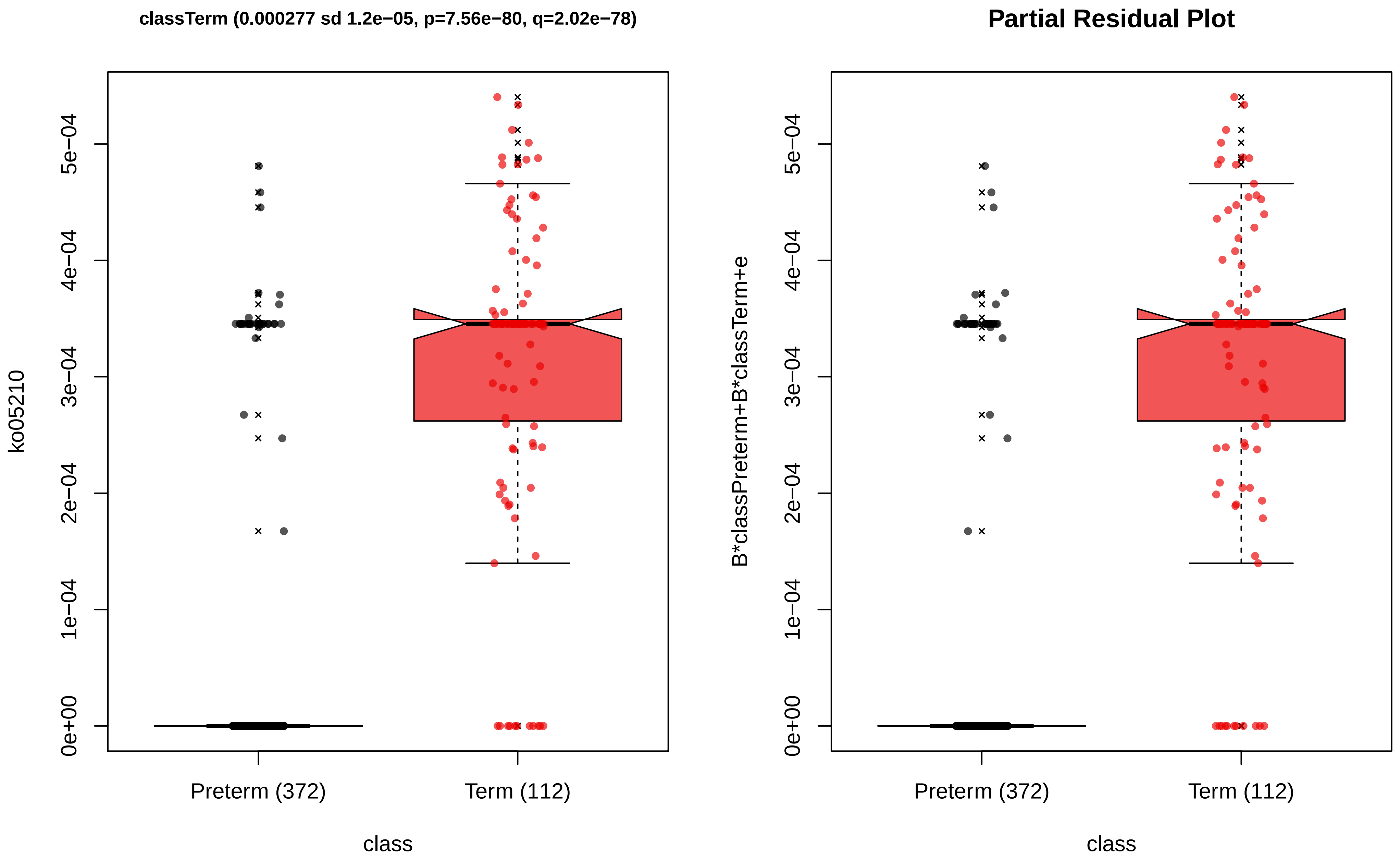
classTerm (0.000277 sd 1.2e−05, p=7.56e−80, q=2.02e−78)
Partial Residual Plot
ko05210
B*classPreterm+B*classTerm+e
0e+00
1e−04
2e−04
3e−04
4e−04
5e−04
Preterm (372)
Term (112)
class

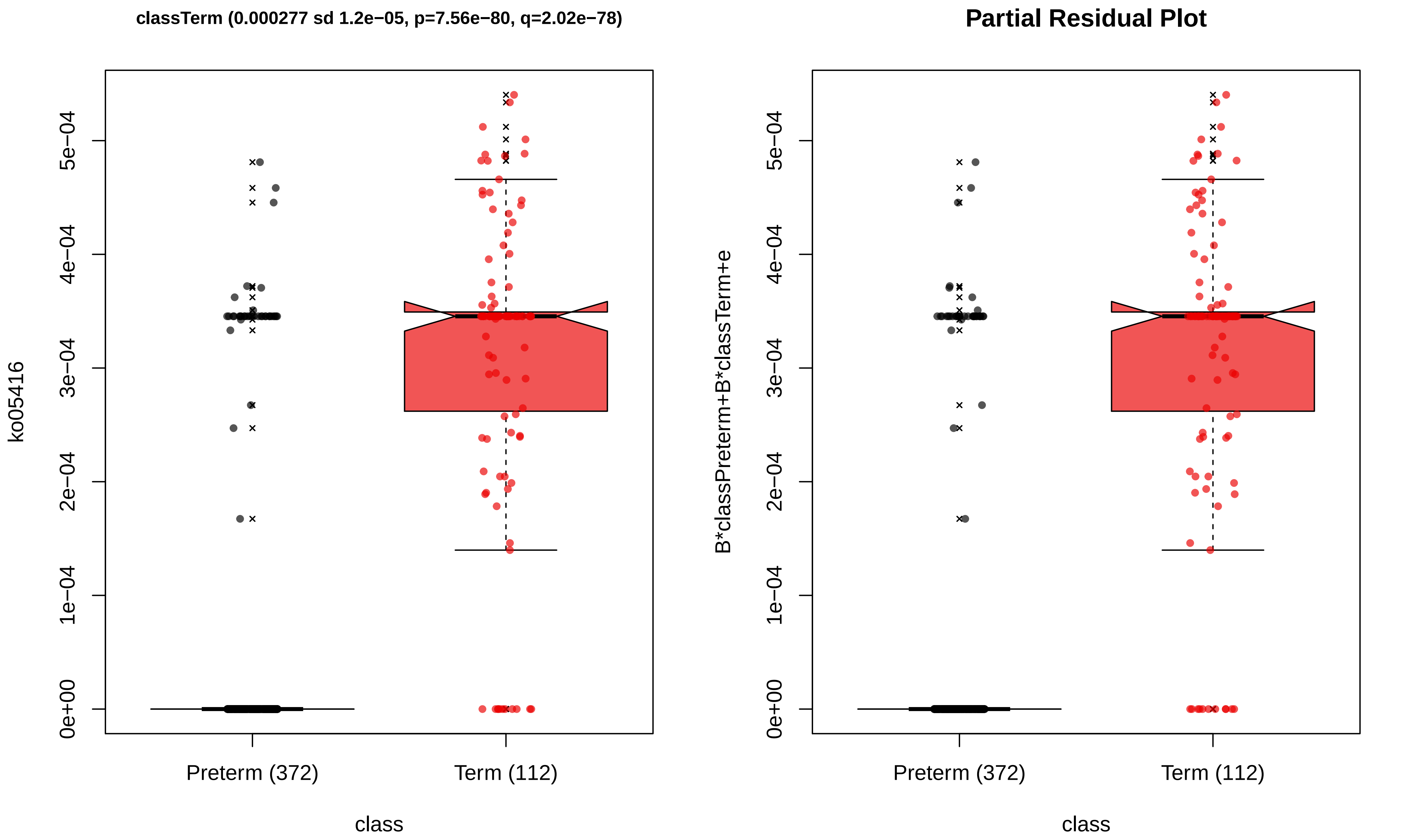

classTerm (0.000277 sd 1.2e−05, p=7.56e−80, q=2.02e−78)
Partial Residual Plot
ko05416
B*classPreterm+B*classTerm+e
0e+00
1e−04
2e−04
3e−04
4e−04
5e−04
Preterm (372)
Term (112)
class

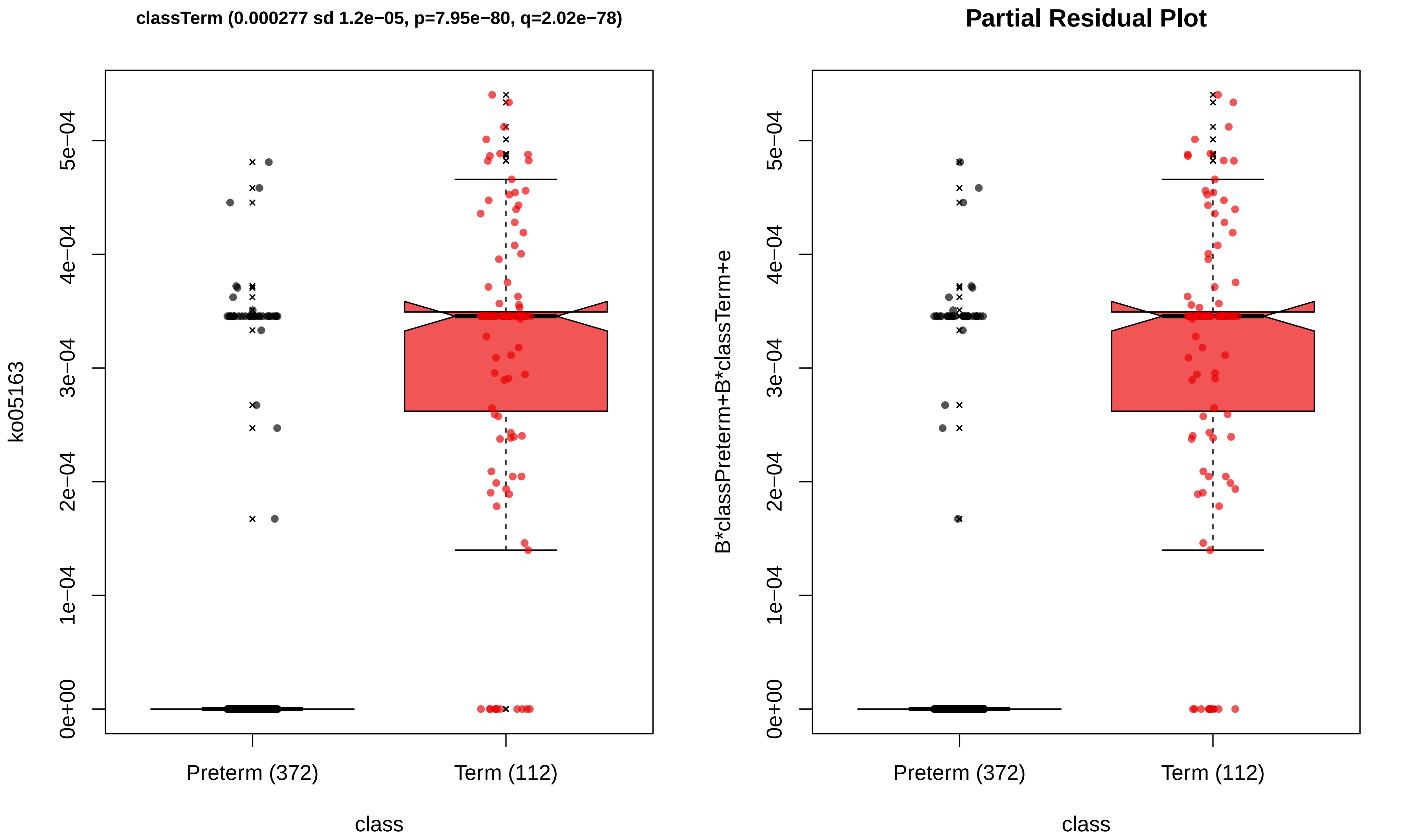
classTerm (0.000277 sd 1.2e−05, p=7.95e−80, q=2.02e−78)
ko05163
0e+00
1e−04
2e−04
3e−04
4e−04
5e−04
Preterm (372)
Term (112)
class
Partial Residual Plot
B*classPreterm+B*classTerm+e
0e+00
1e−04
2e−04
3e−04
4e−04
5e−04
Preterm (372)
Term (112)
class

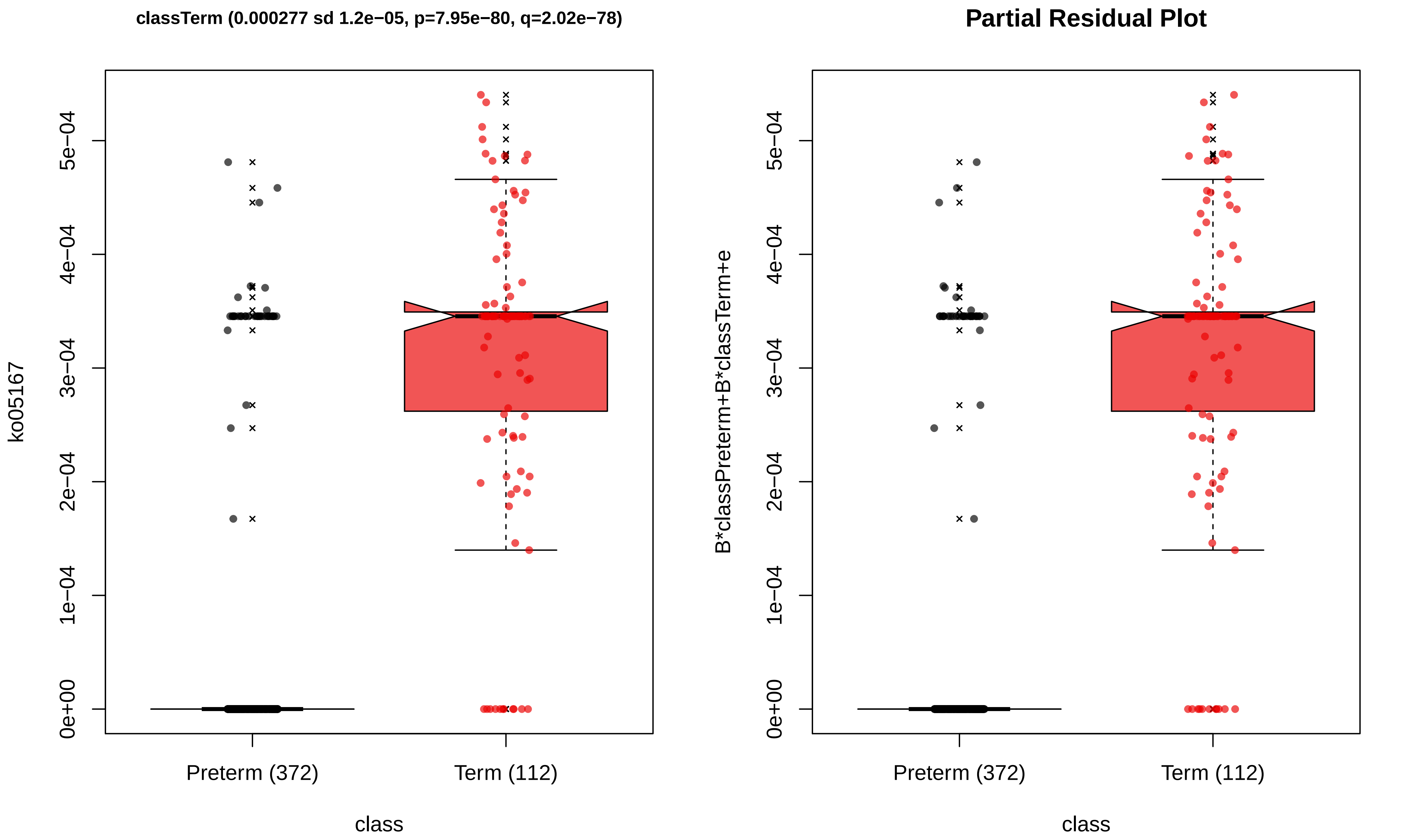

classTerm (0.000277 sd 1.2e−05, p=7.95e−80, q=2.02e−78)
ko05167
0e+00
1e−04
2e−04
3e−04
4e−04
5e−04
Preterm (372)
Term (112)
class
Partial Residual Plot
B*classPreterm+B*classTerm+e
0e+00
1e−04
2e−04
3e−04
4e−04
5e−04
Preterm (372)
Term (112)
class

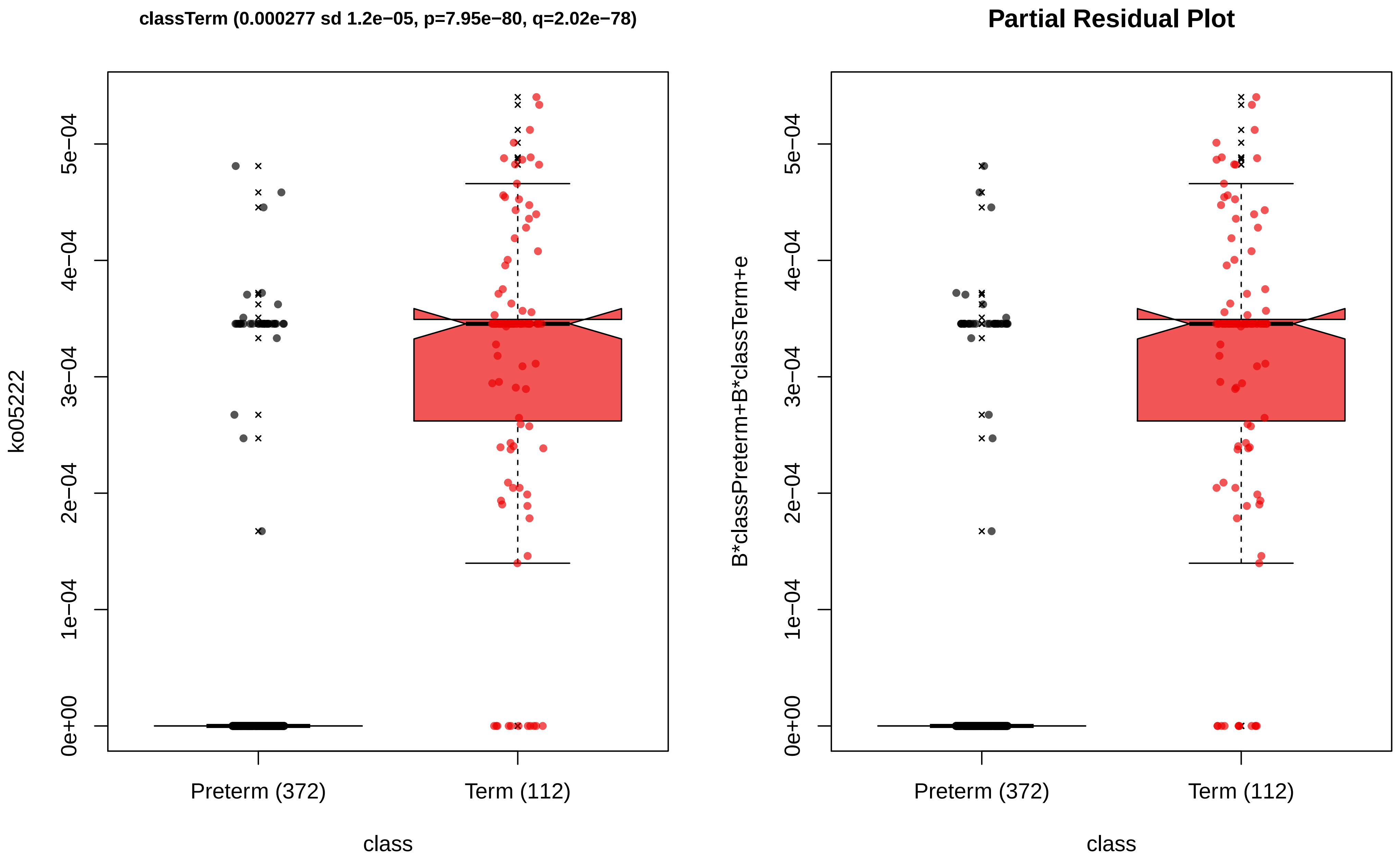

classTerm (0.000277 sd 1.2e−05, p=7.95e−80, q=2.02e−78)
ko05222
5e−04
4e−04
3e−04
2e−04
1e−04
0e+00
Preterm (372)
Term (112)
class
Partial Residual Plot
B*classPreterm+B*classTerm+e
5e−04
4e−04
3e−04
2e−04
1e−04
0e+00
Preterm (372)
Term (112)
class

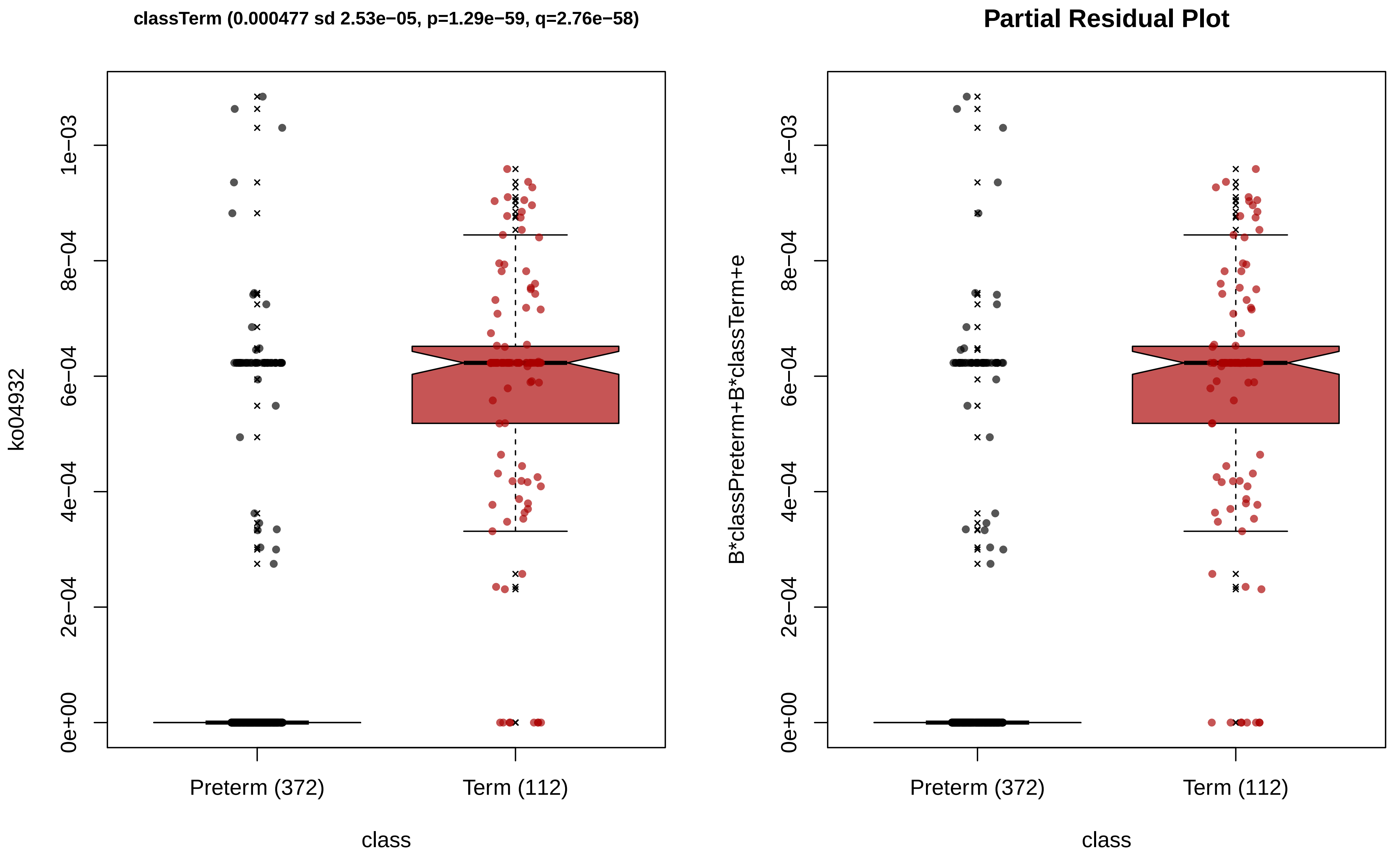

classTerm (0.000477 sd 2.53e−05, p=1.29e−59, q=2.76e−58)
ko04932
0e+00
2e−04
4e−04
6e−04
8e−04
1e−03
Preterm (372)
Term (112)
class
Partial Residual Plot
B*classPreterm+B*classTerm+e
0e+00
2e−04
4e−04
6e−04
8e−04
1e−03
Preterm (372)
Term (112)
class

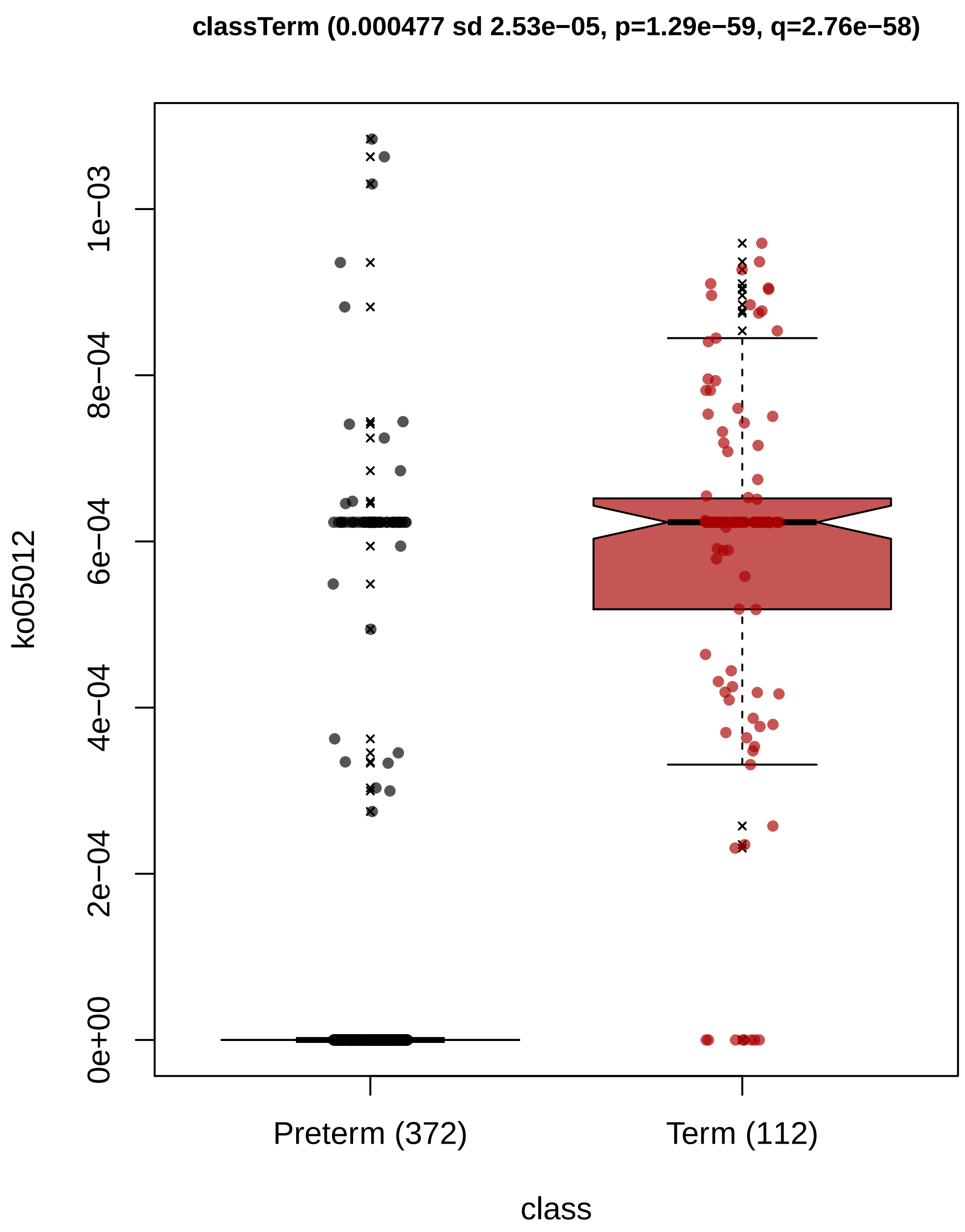
classTerm (0.000477 sd 2.53e−05, p=1.29e−59, q=2.76e−58)
ko05012
0e+00
2e−04
4e−04
6e−04
8e−04
1e−03
Preterm (372)
Term (112)
class

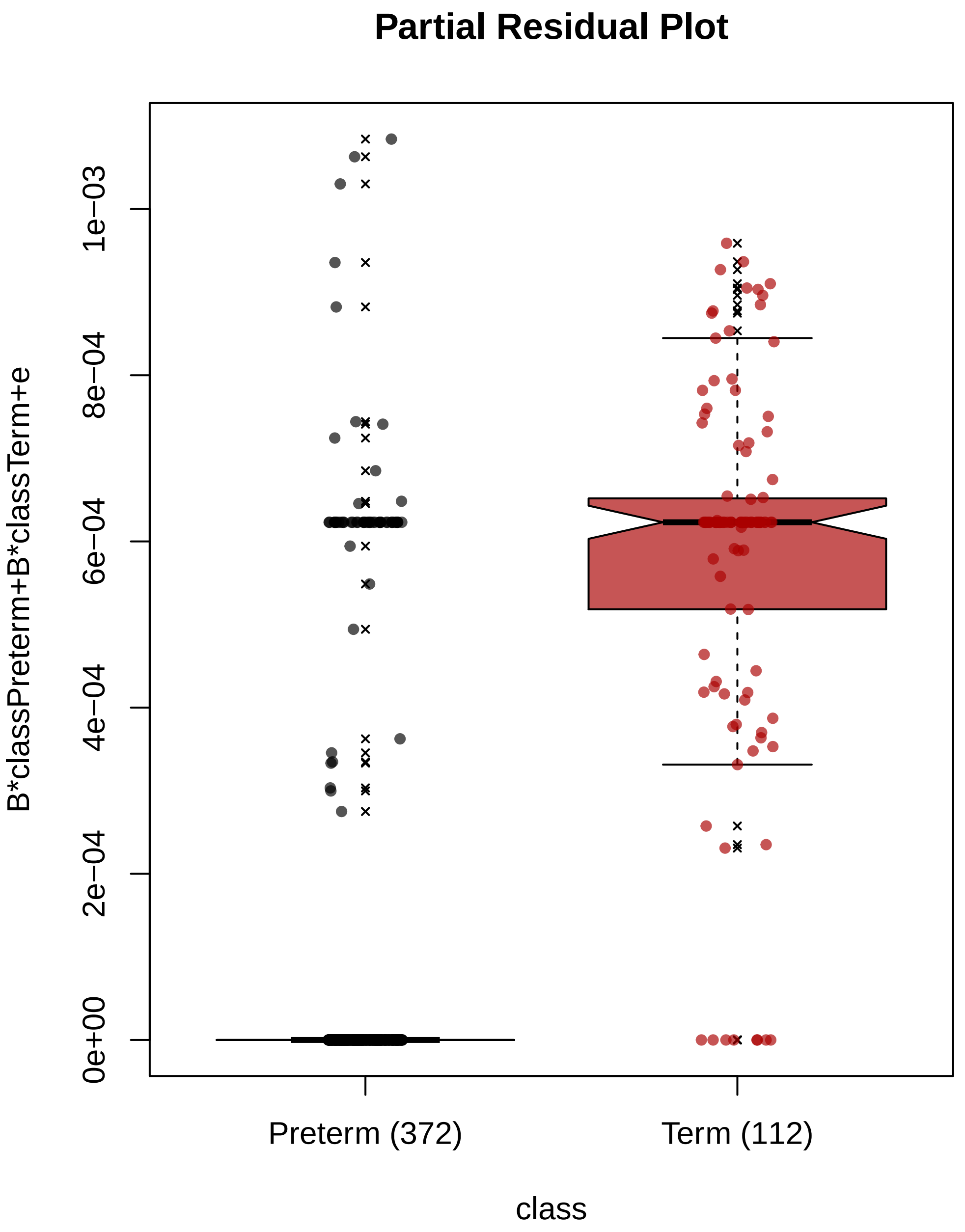
Partial Residual Plot
B*classPreterm+B*classTerm+e
0e+00
2e−04
4e−04
6e−04
8e−04
1e−03
Preterm (372)
Term (112)
class

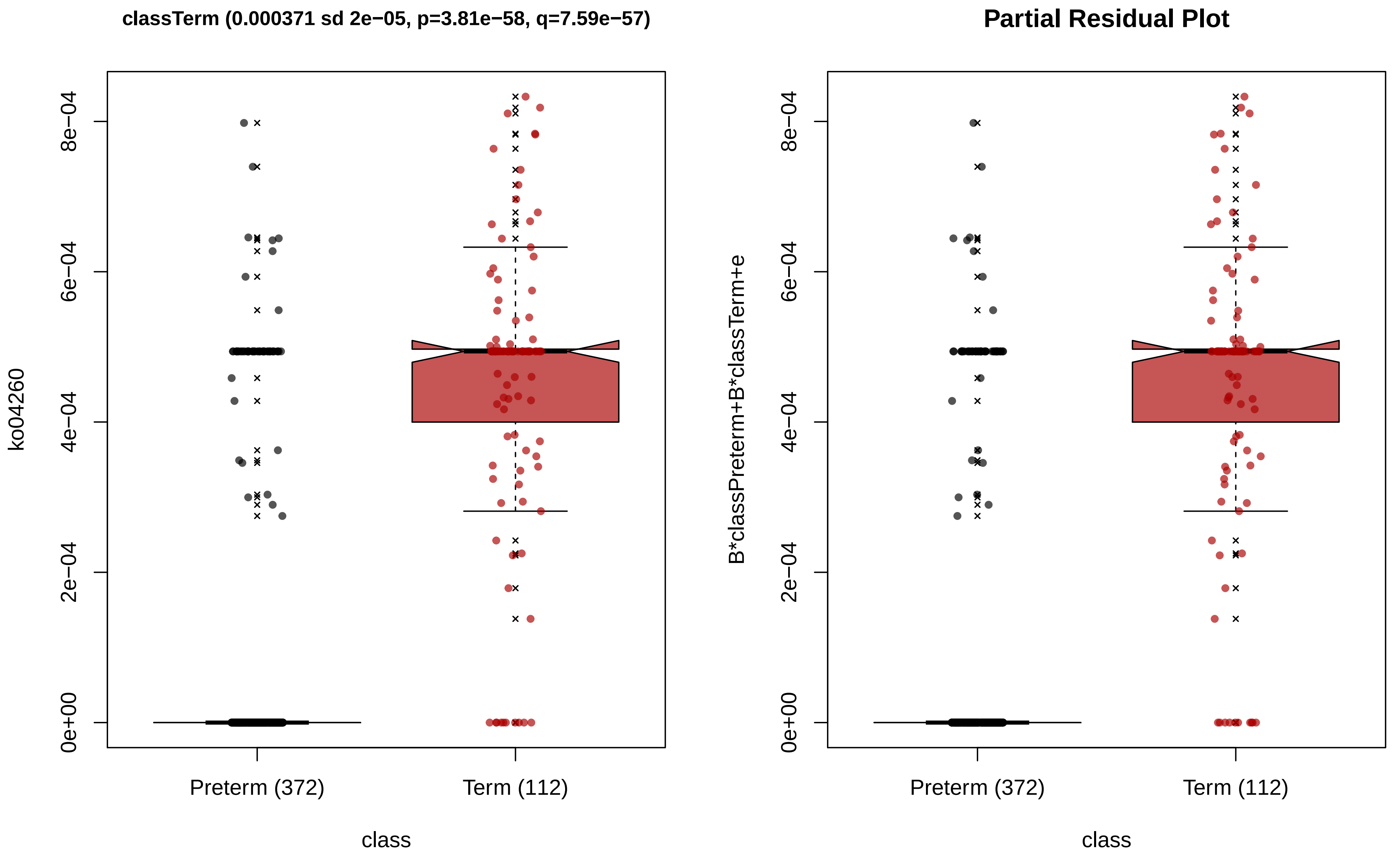

classTerm (0.000371 sd 2e−05, p=3.81e−58, q=7.59e−57)
Partial Residual Plot
ko04260
B*classPreterm+B*classTerm+e
0e+00
2e−04
4e−04
6e−04
8e−04
Preterm (372)
Term (112)
class

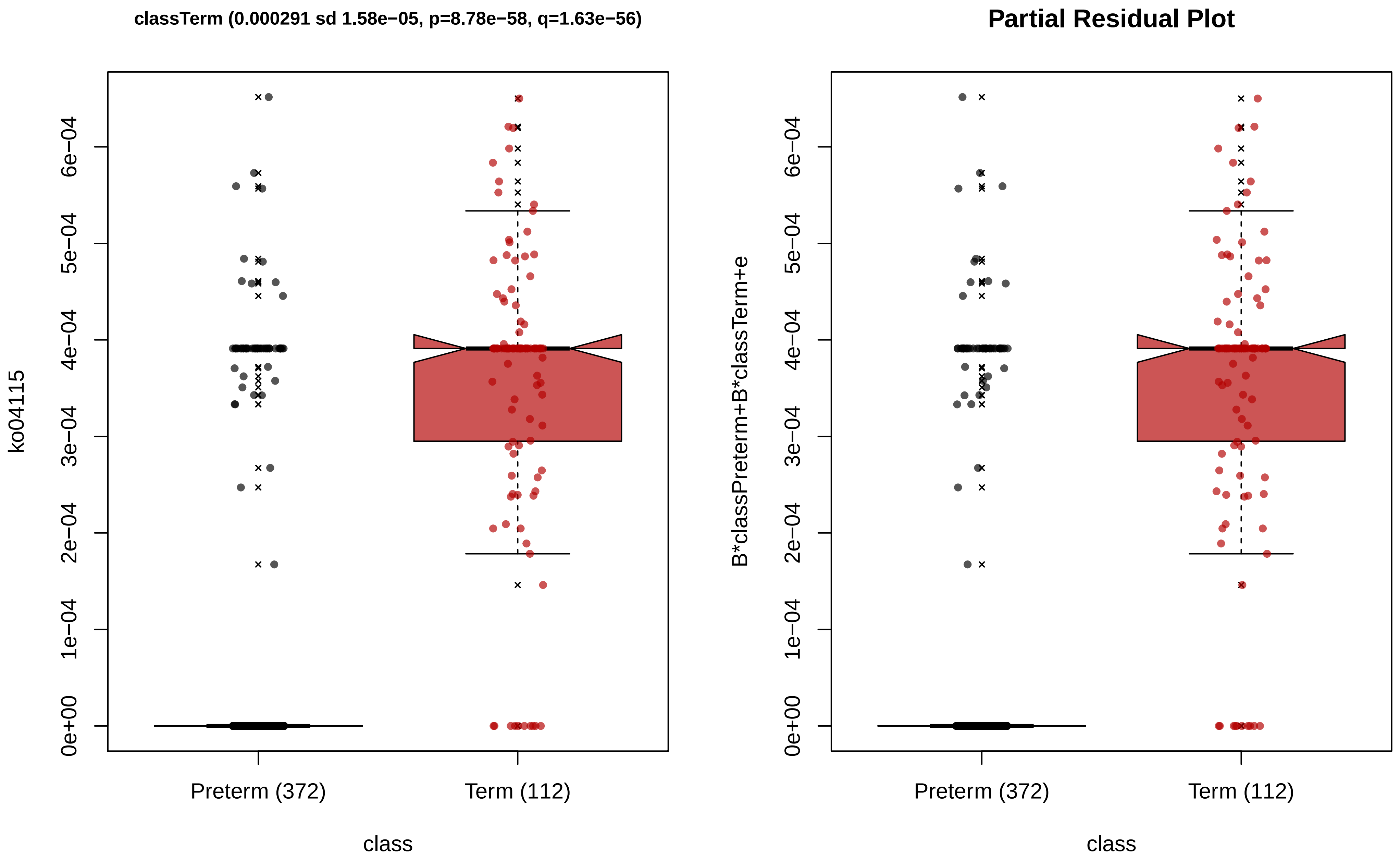

classTerm (0.000291 sd 1.58e−05, p=8.78e−58, q=1.63e−56)
ko04115
0e+00
1e−04
2e−04
3e−04
4e−04
5e−04
6e−04
Preterm (372)
Term (112)
class
Partial Residual Plot
B*classPreterm+B*classTerm+e
0e+00
1e−04
2e−04
3e−04
4e−04
5e−04
6e−04
Preterm (372)
Term (112)
class

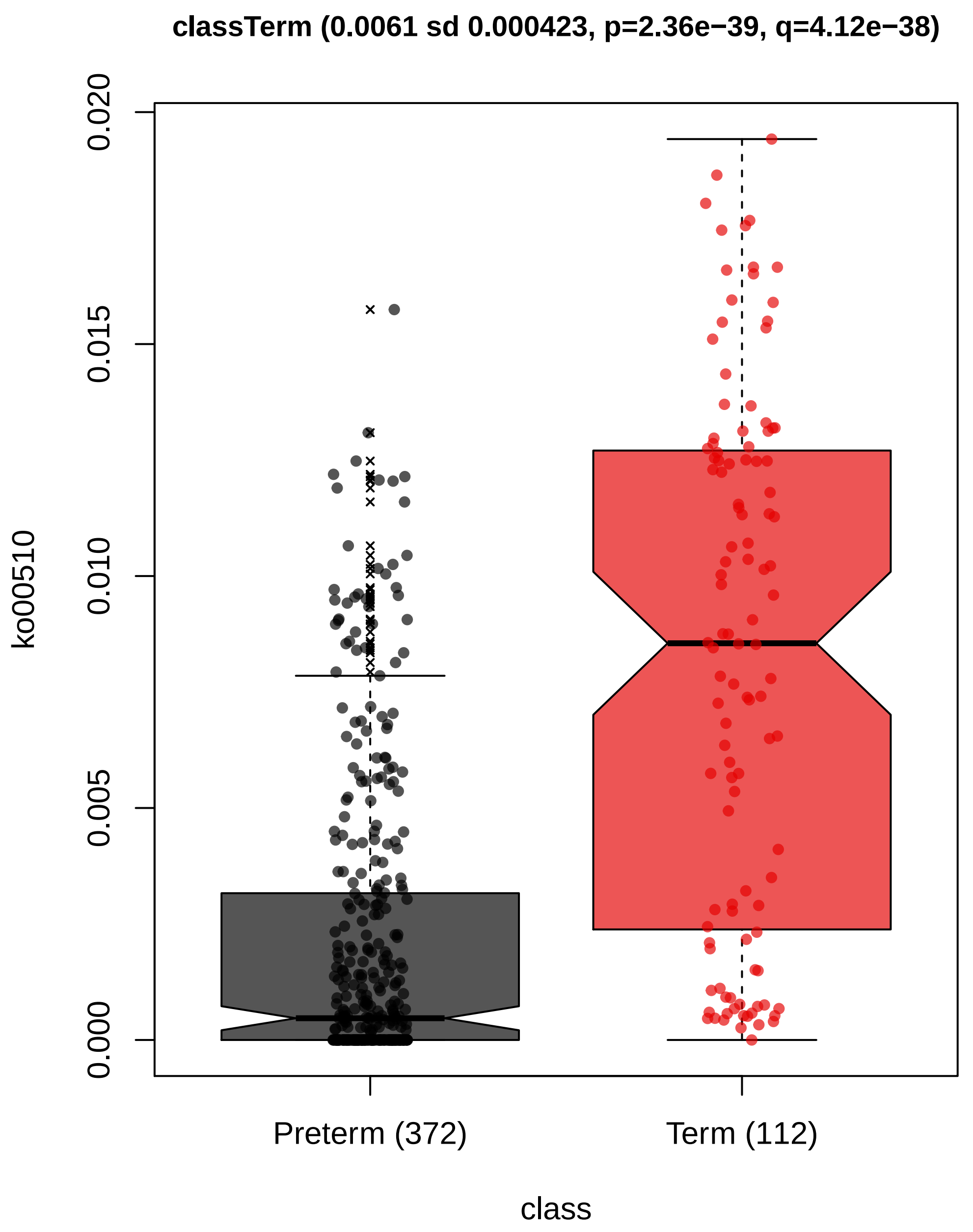

classTerm (0.0061 sd 0.000423, p=2.36e−39, q=4.12e−38)
ko00510
0.000
0.005
0.010
0.015
0.020
Preterm (372)
Term (112)
class

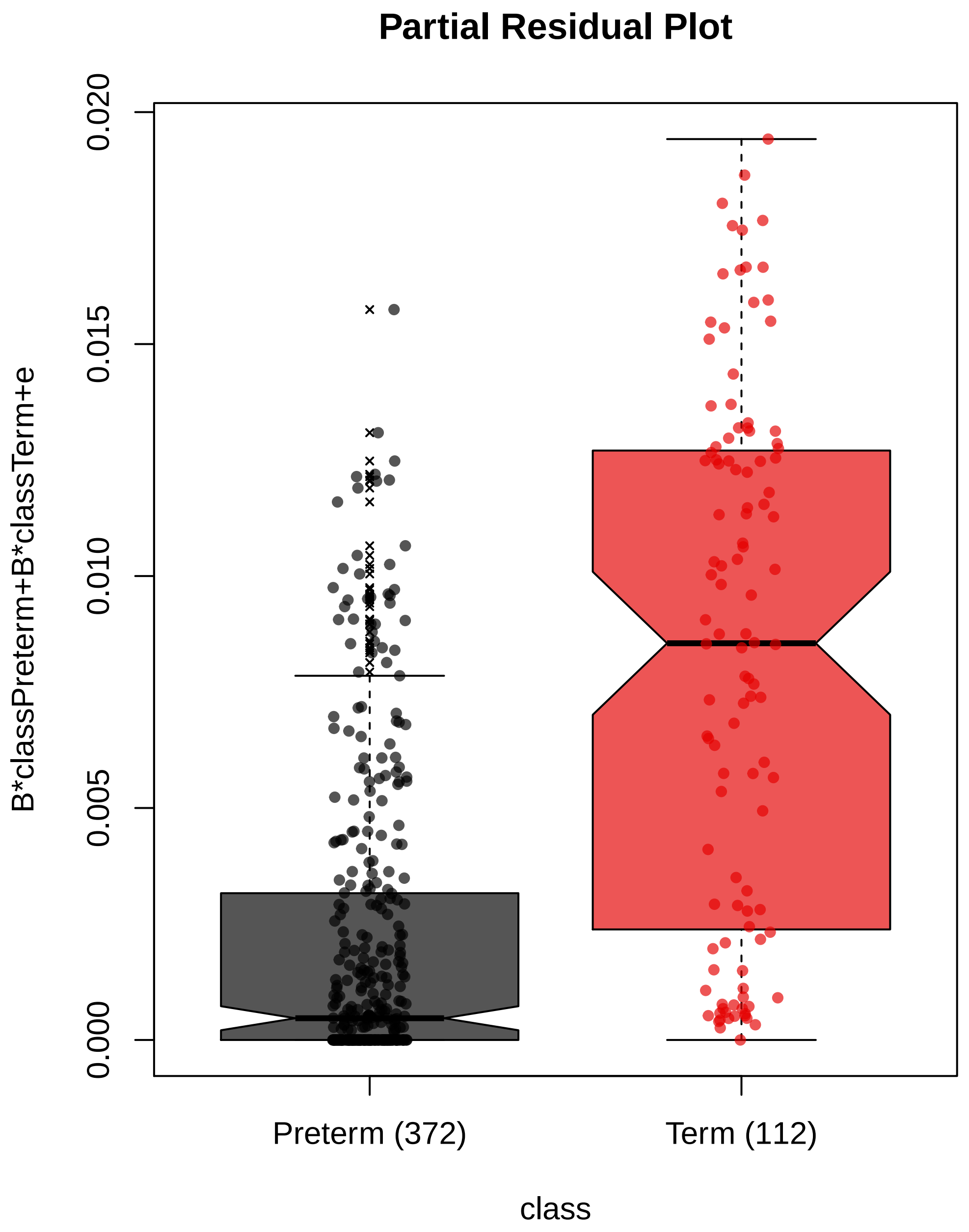

Partial Residual Plot
B*classPreterm+B*classTerm+e
0.000
0.005
0.010
0.015
0.020
Preterm (372)
Term (112)
class

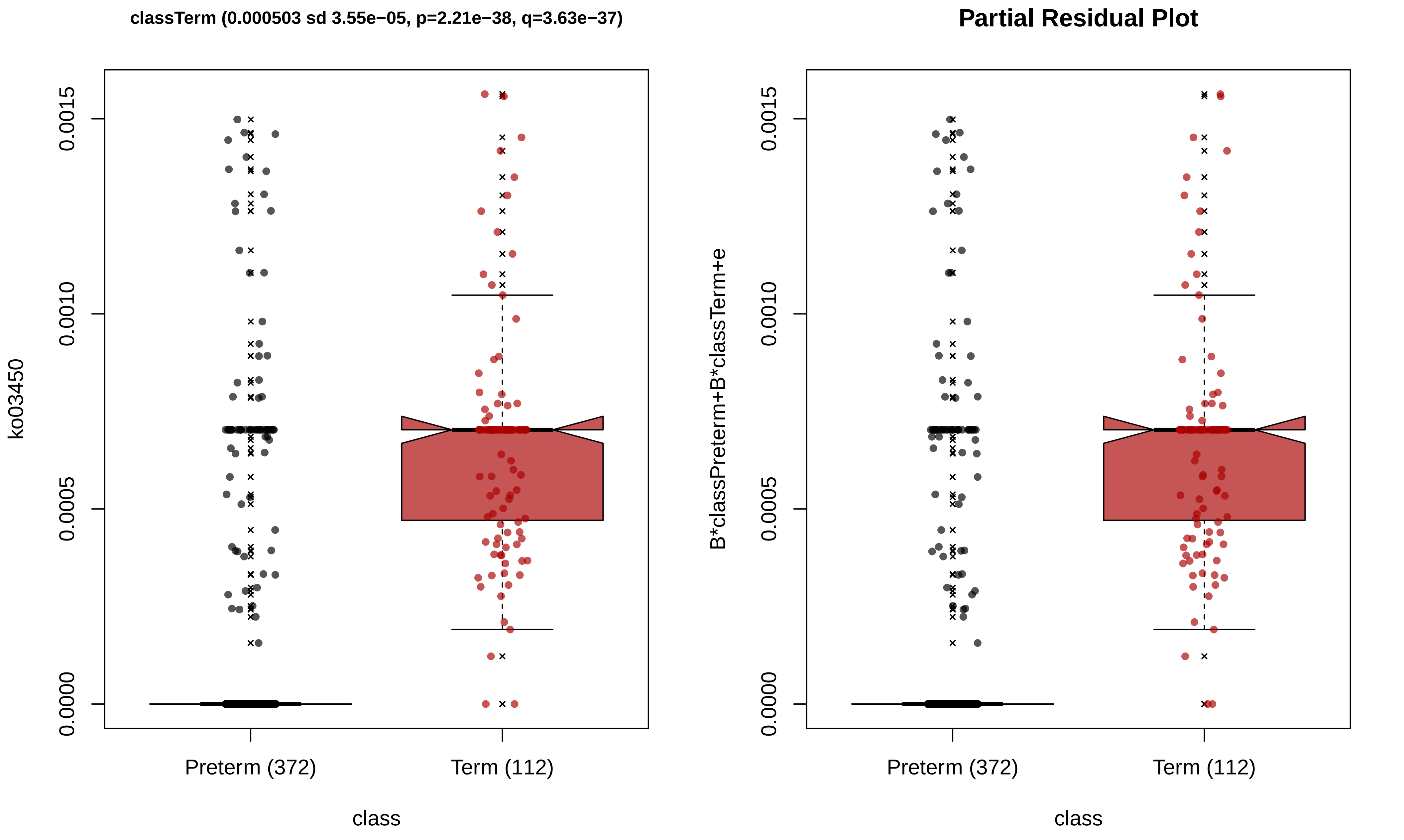

classTerm (0.000503 sd 3.55e−05, p=2.21e−38, q=3.63e−37)
ko03450
0.0000
0.0005
0.0010
0.0015
Preterm (372)
Term (112)
class
Partial Residual Plot
B*classPreterm+B*classTerm+e
0.0000
0.0005
0.0010
0.0015
Preterm (372)
Term (112)
class

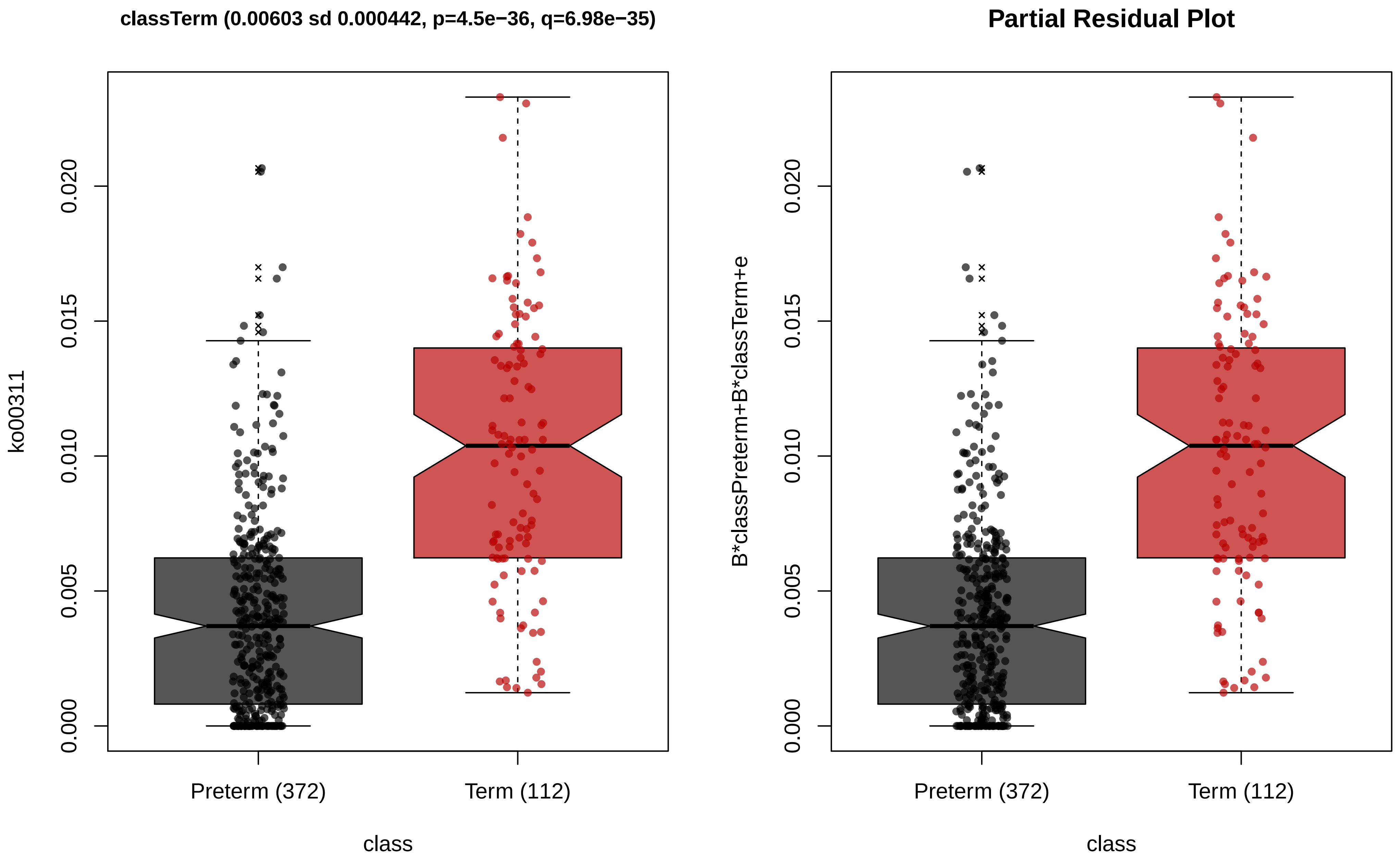

classTerm (0.00603 sd 0.000442, p=4.5e−36, q=6.98e−35)
ko00311
0.000
0.005
0.010
0.015
0.020
Preterm (372)
Term (112)
class
Partial Residual Plot
B*classPreterm+B*classTerm+e
0.000
0.005
0.010
0.015
0.020
Preterm (372)
Term (112)
class

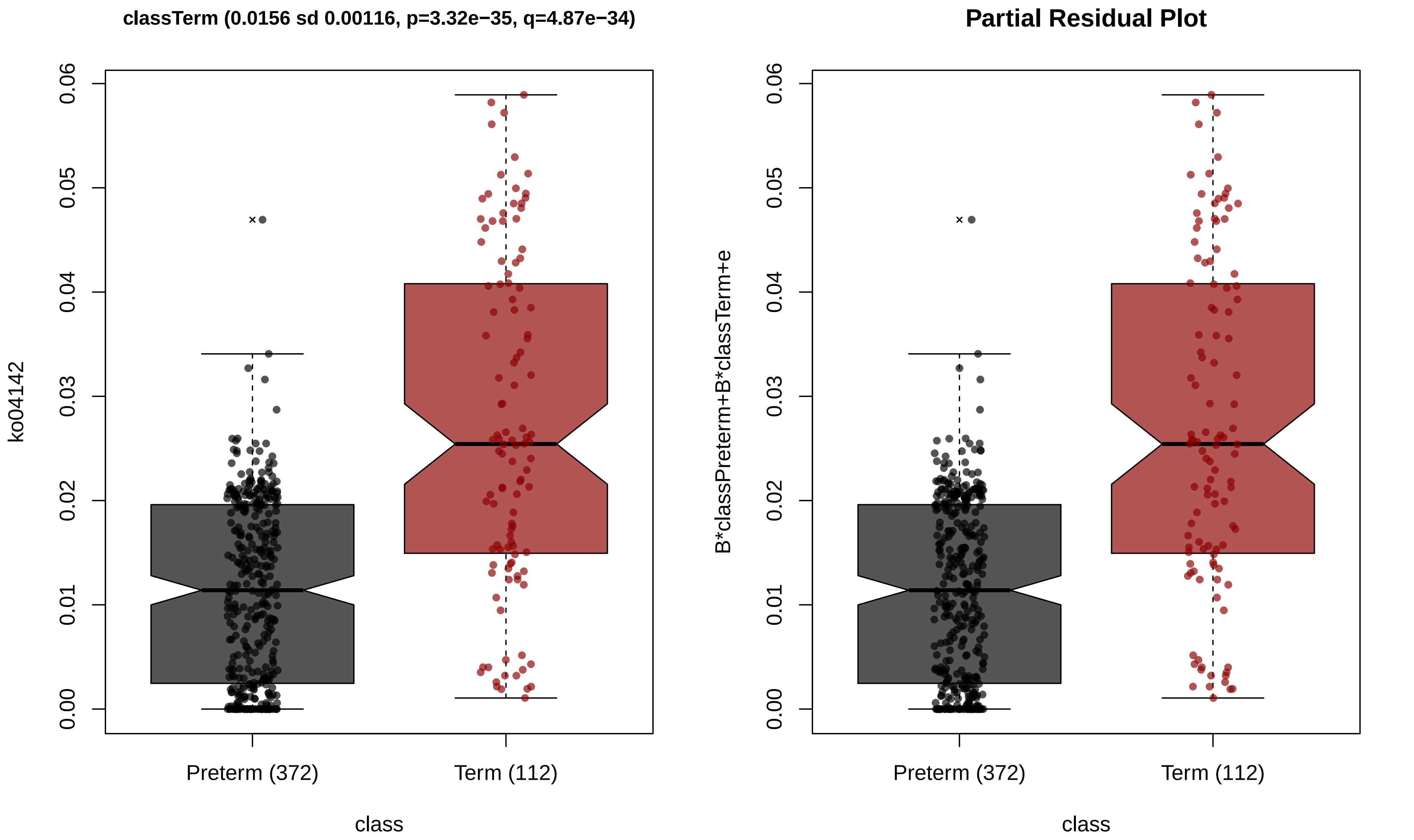

classTerm (0.0156 sd 0.00116, p=3.32e−35, q=4.87e−34)
Partial Residual Plot
ko04142
B*classPreterm+B*classTerm+e
0.00
0.01
0.02
0.03
0.04
0.05
0.06
Preterm (372)
Term (112)
class

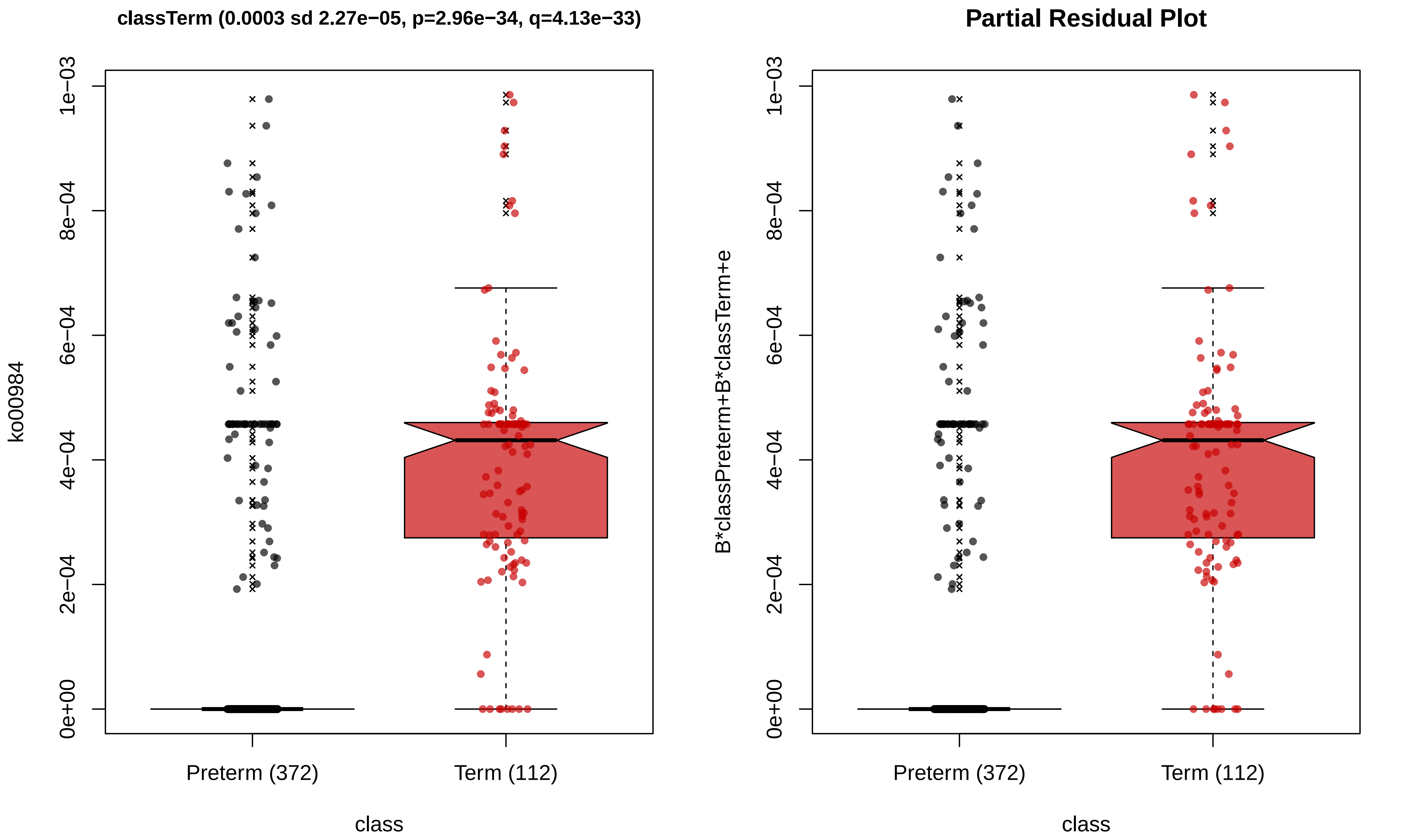

classTerm (0.0003 sd 2.27e−05, p=2.96e−34, q=4.13e−33)
ko00984
0e+00
2e−04
4e−04
6e−04
8e−04
1e−03
Preterm (372)
Term (112)
class
Partial Residual Plot
B*classPreterm+B*classTerm+e
0e+00
2e−04
4e−04
6e−04
8e−04
1e−03
Preterm (372)
Term (112)
class

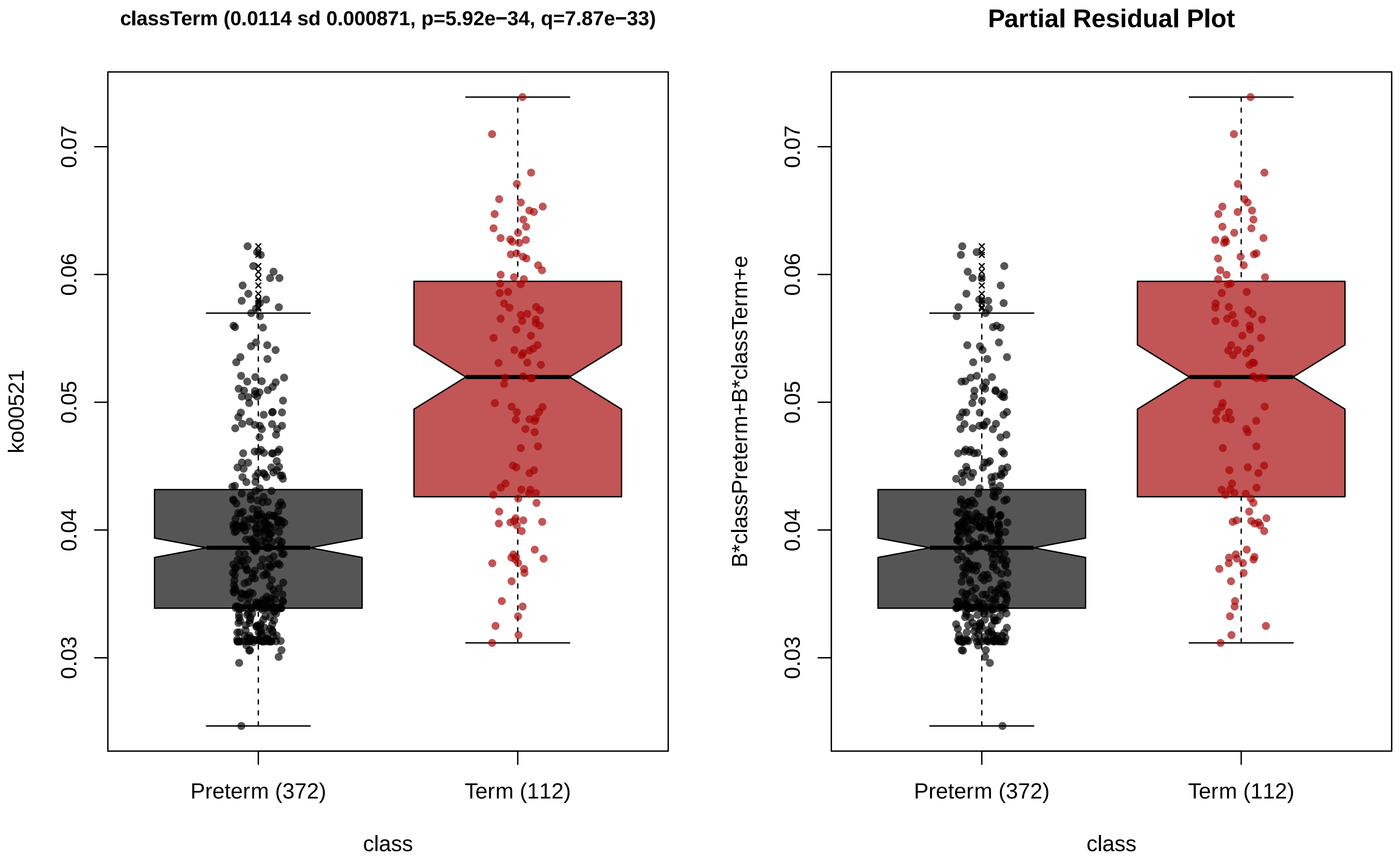
classTerm (0.0114 sd 0.000871, p=5.92e−34, q=7.87e−33)
Partial Residual Plot
ko00521
B*classPreterm+B*classTerm+e
0.03
0.04
0.05
0.06
0.07
Preterm (372)
Term (112)
class

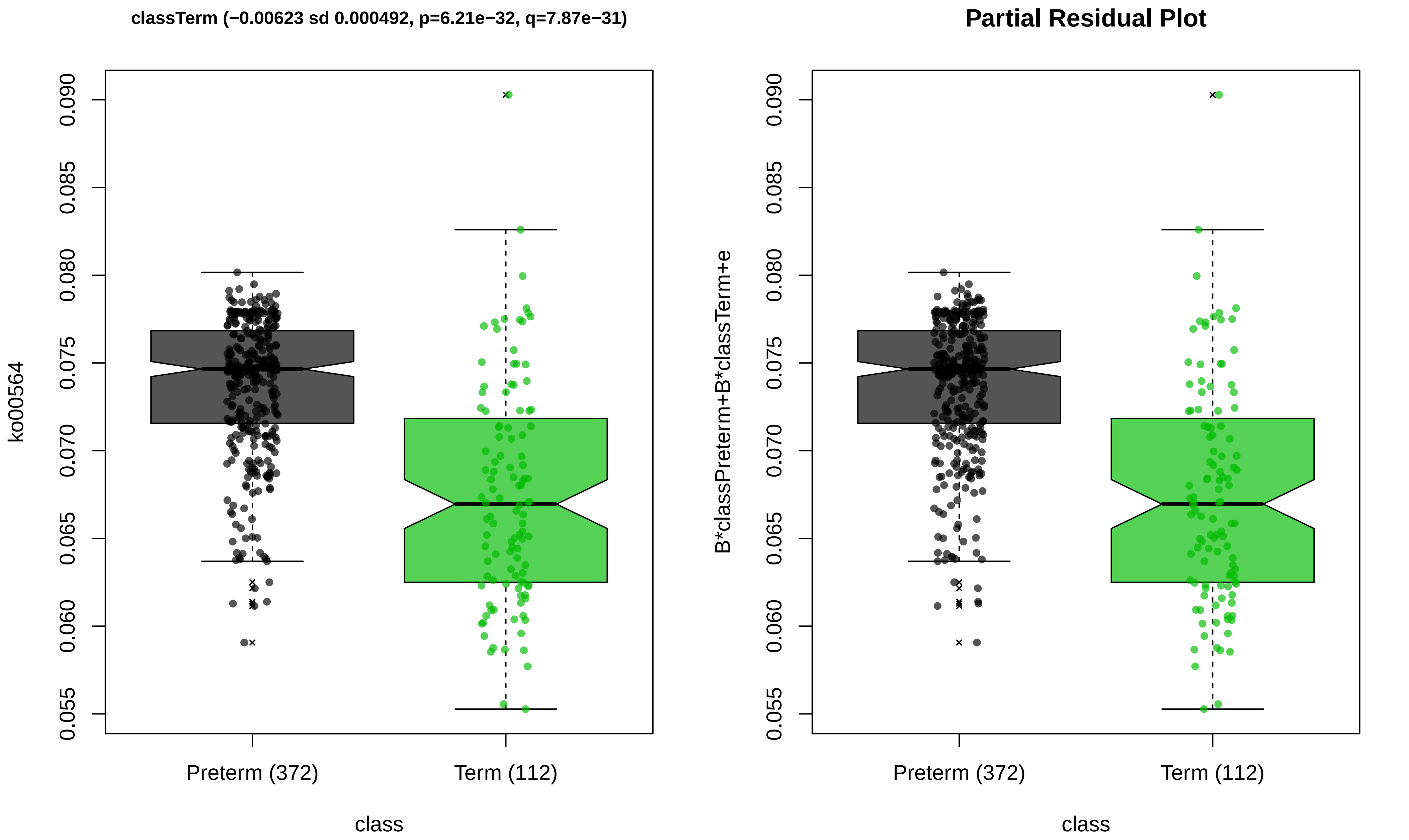

classTerm (−0.00623 sd 0.000492, p=6.21e−32, q=7.87e−31)
Partial Residual Plot
ko00564
B*classPreterm+B*classTerm+e
0.055
0.060
0.065
0.070
0.075
0.080
0.085
0.090
Preterm (372)
Term (112)
class

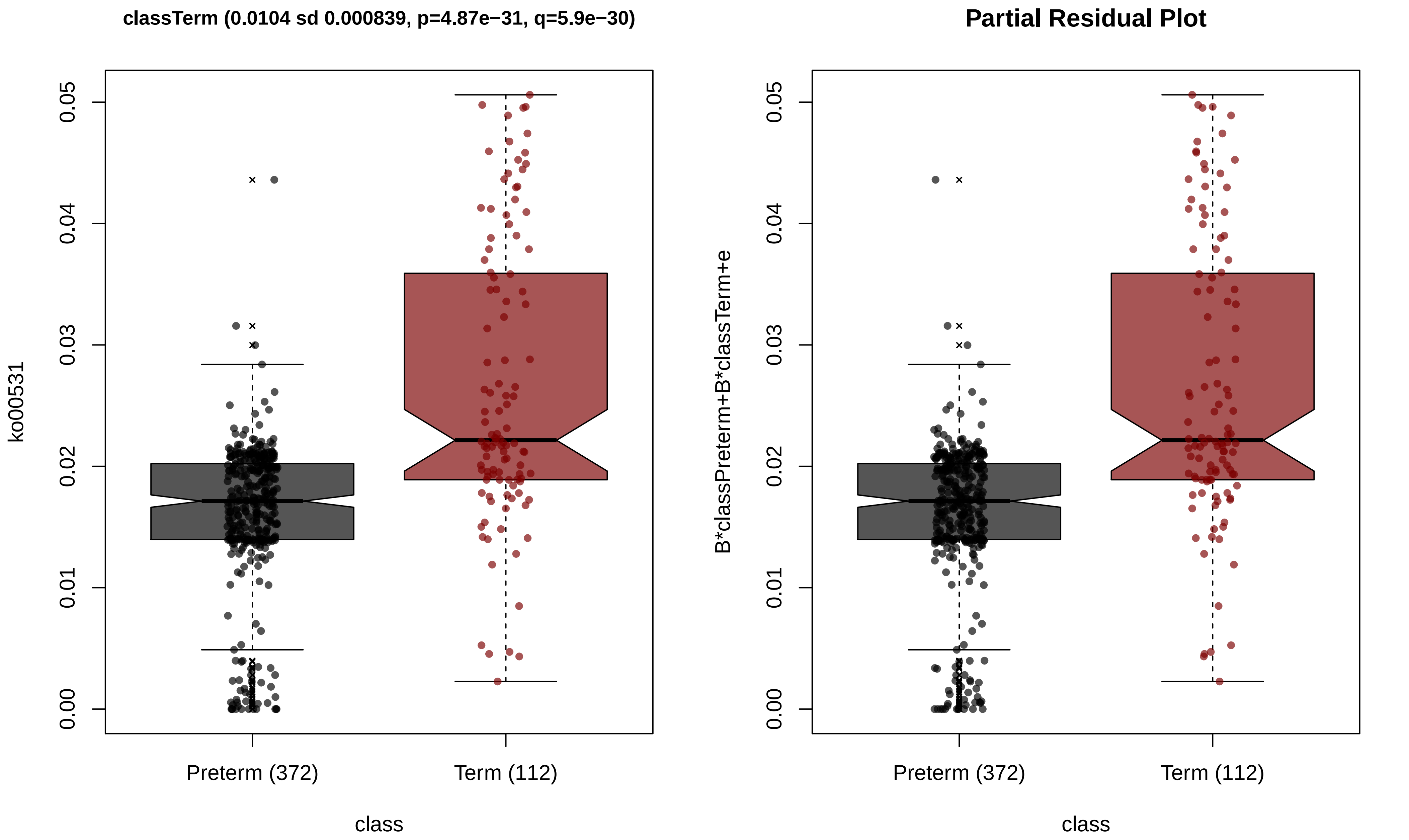

classTerm (0.0104 sd 0.000839, p=4.87e−31, q=5.9e−30)
Partial Residual Plot
ko00531
B*classPreterm+B*classTerm+e
0.00
0.01
0.02
0.03
0.04
0.05
Preterm (372)
Term (112)
class

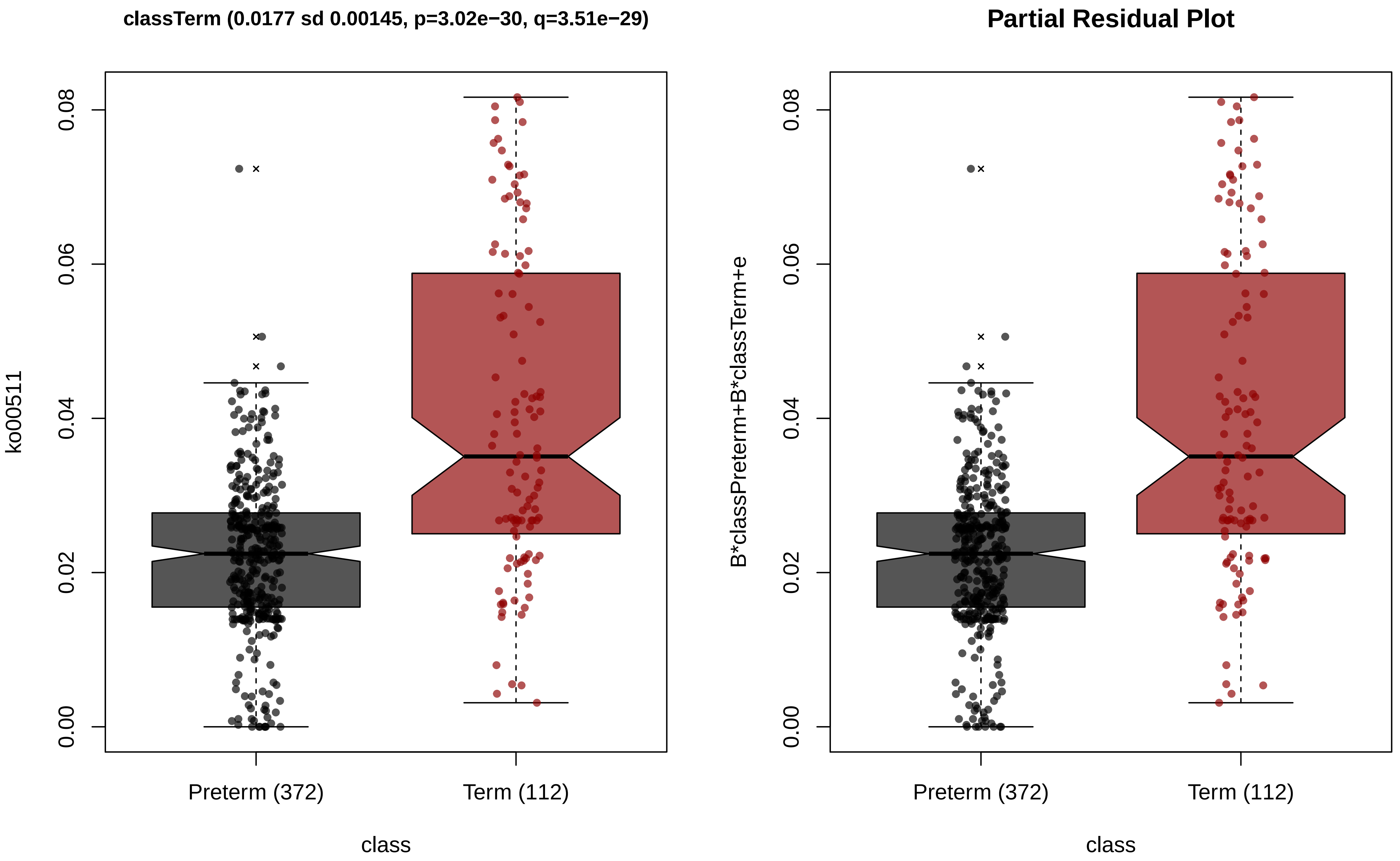

classTerm (0.0177 sd 0.00145, p=3.02e−30, q=3.51e−29)
ko00511
0.00
0.02
0.04
0.06
0.08
Preterm (372)
Term (112)
class
Partial Residual Plot
B*classPreterm+B*classTerm+e
0.00
0.02
0.04
0.06
0.08
Preterm (372)
Term (112)
class

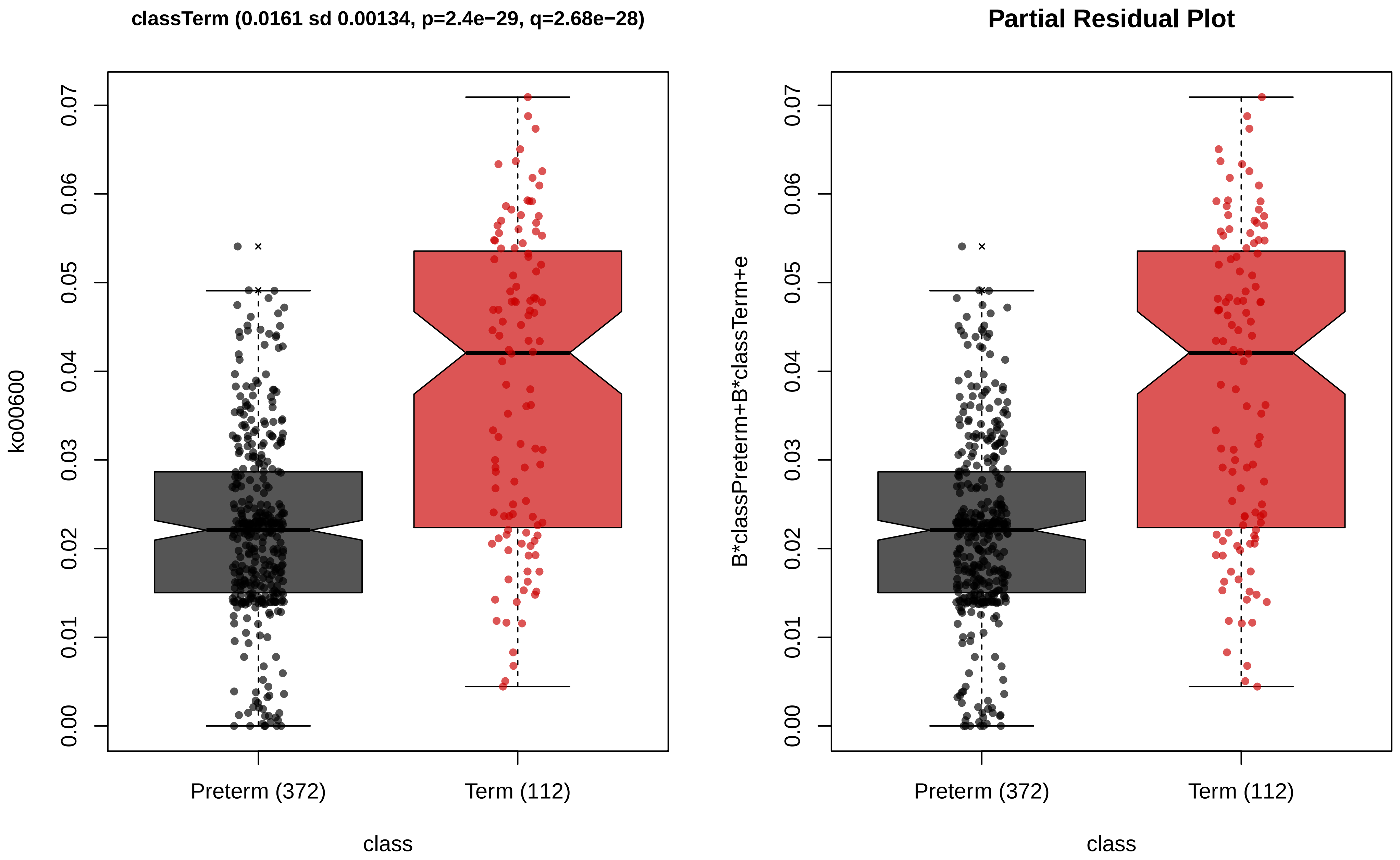

classTerm (0.0161 sd 0.00134, p=2.4e−29, q=2.68e−28)
ko00600
0.00
0.01
0.02
0.03
0.04
0.05
0.06
0.07
Preterm (372)
Term (112)
class
Partial Residual Plot
B*classPreterm+B*classTerm+e
0.00
0.01
0.02
0.03
0.04
0.05
0.06
0.07
Preterm (372)
Term (112)
class

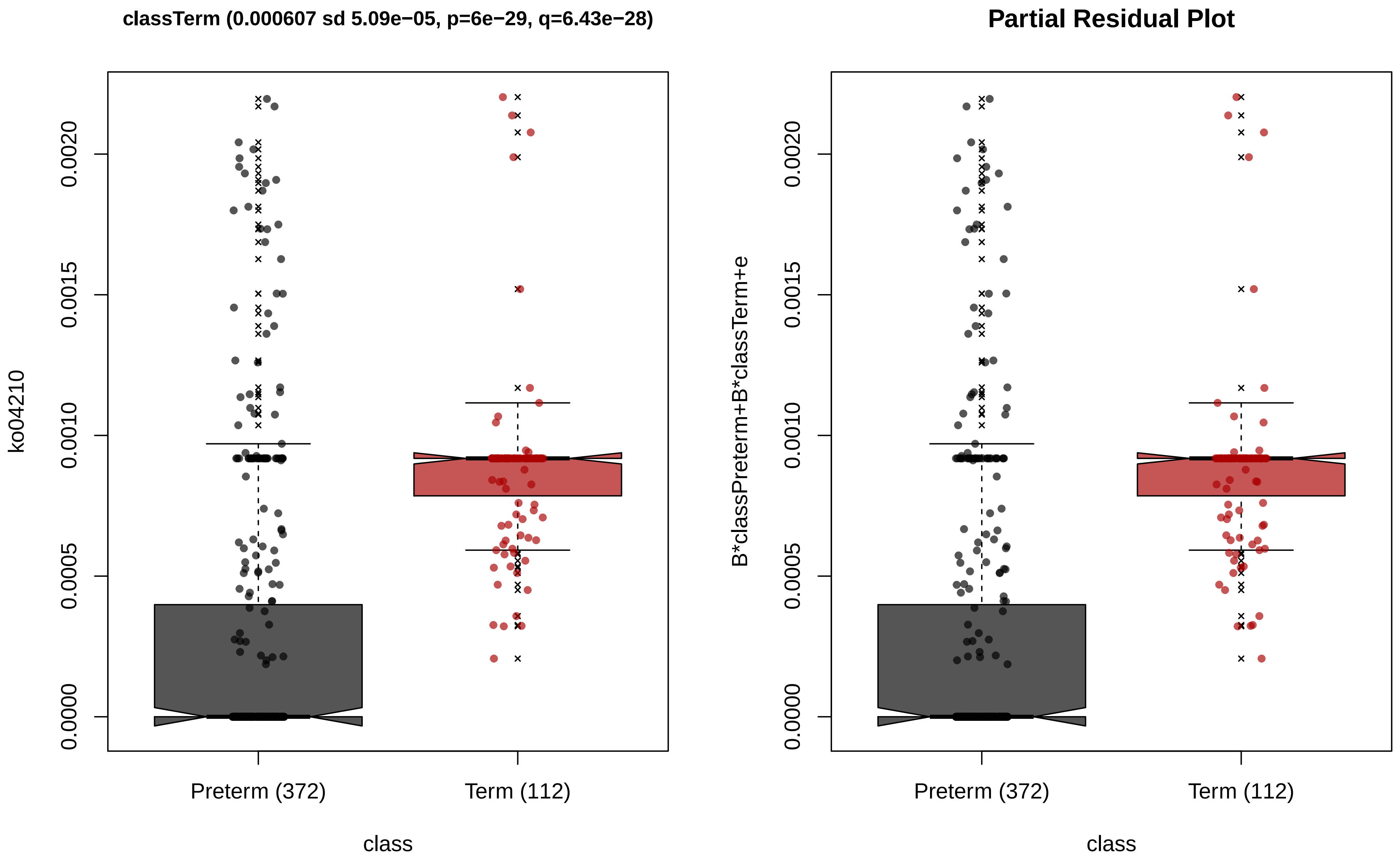

classTerm (0.000607 sd 5.09e−05, p=6e−29, q=6.43e−28)
ko04210
0.0000
0.0005
0.0010
0.0015
0.0020
Preterm (372)
Term (112)
class
Partial Residual Plot
B*classPreterm+B*classTerm+e
0.0000
0.0005
0.0010
0.0015
0.0020
Preterm (372)
Term (112)
class

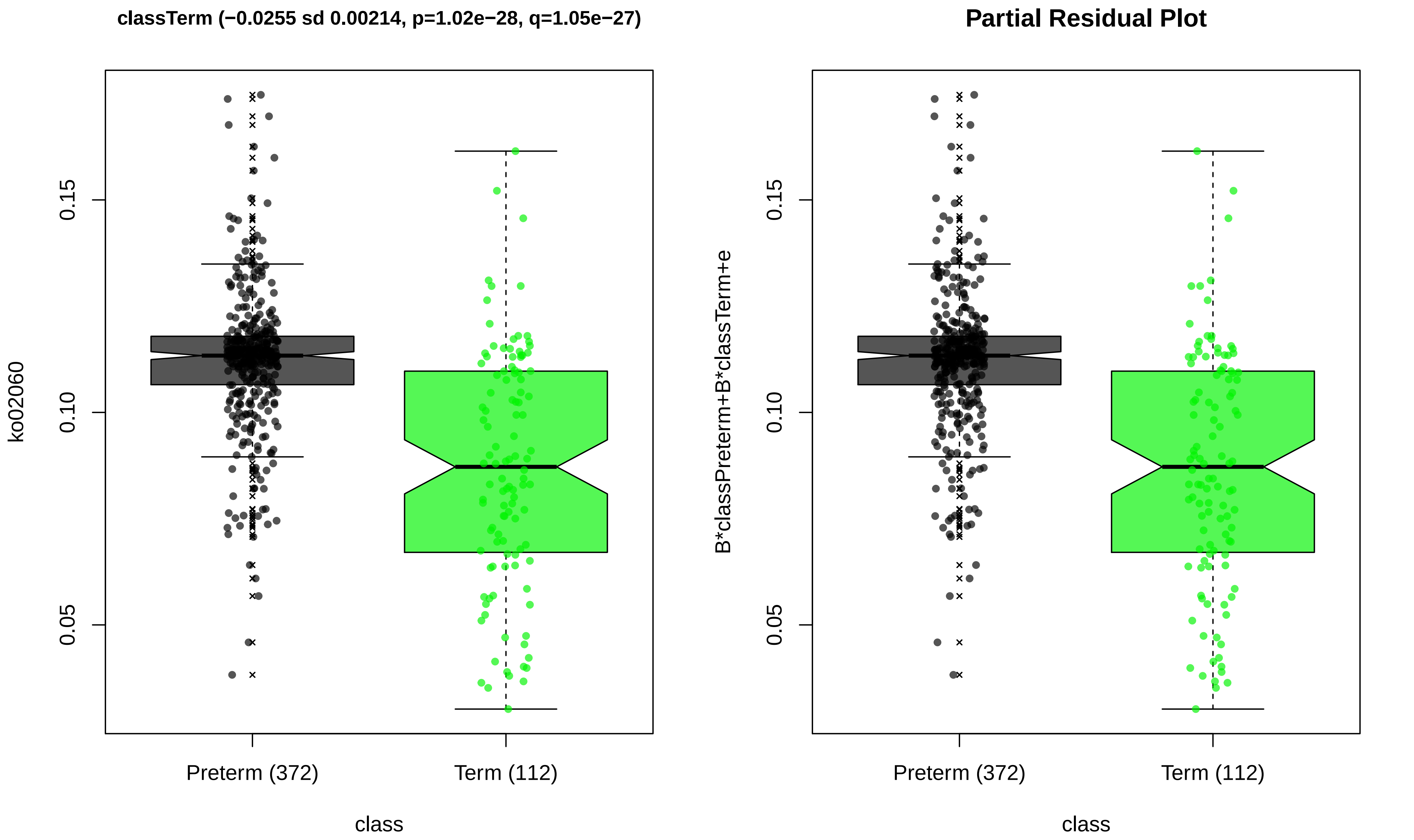

classTerm (−0.0255 sd 0.00214, p=1.02e−28, q=1.05e−27)
ko02060
0.05
0.10
0.15
Preterm (372)
Term (112)
class
Partial Residual Plot
B*classPreterm+B*classTerm+e
0.05
0.10
0.15
Preterm (372)
Term (112)
class

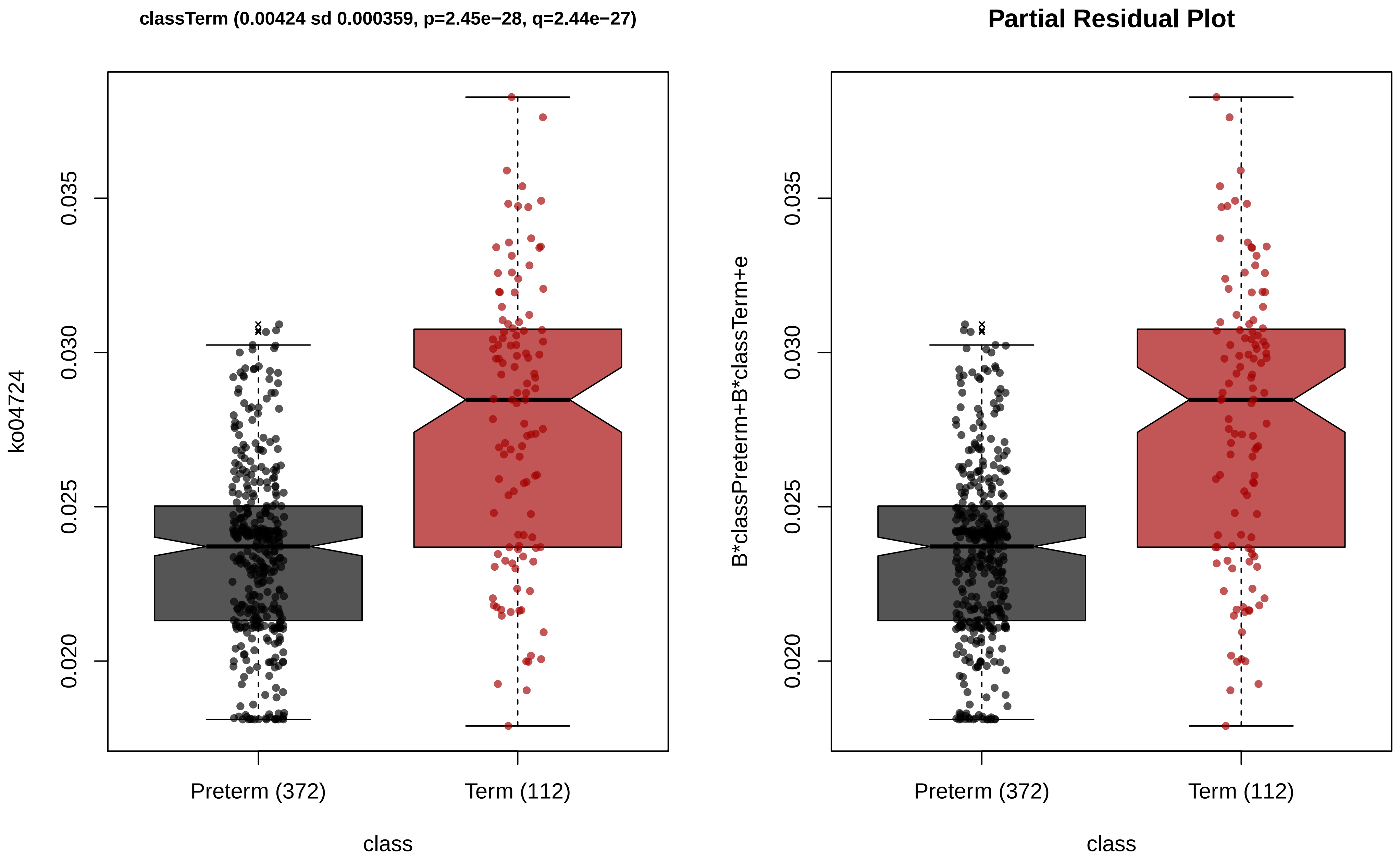

classTerm (0.00424 sd 0.000359, p=2.45e−28, q=2.44e−27)
Partial Residual Plot
ko04724
B*classPreterm+B*classTerm+e
0.020
0.025
0.030
0.035
Preterm (372)
Term (112)
class

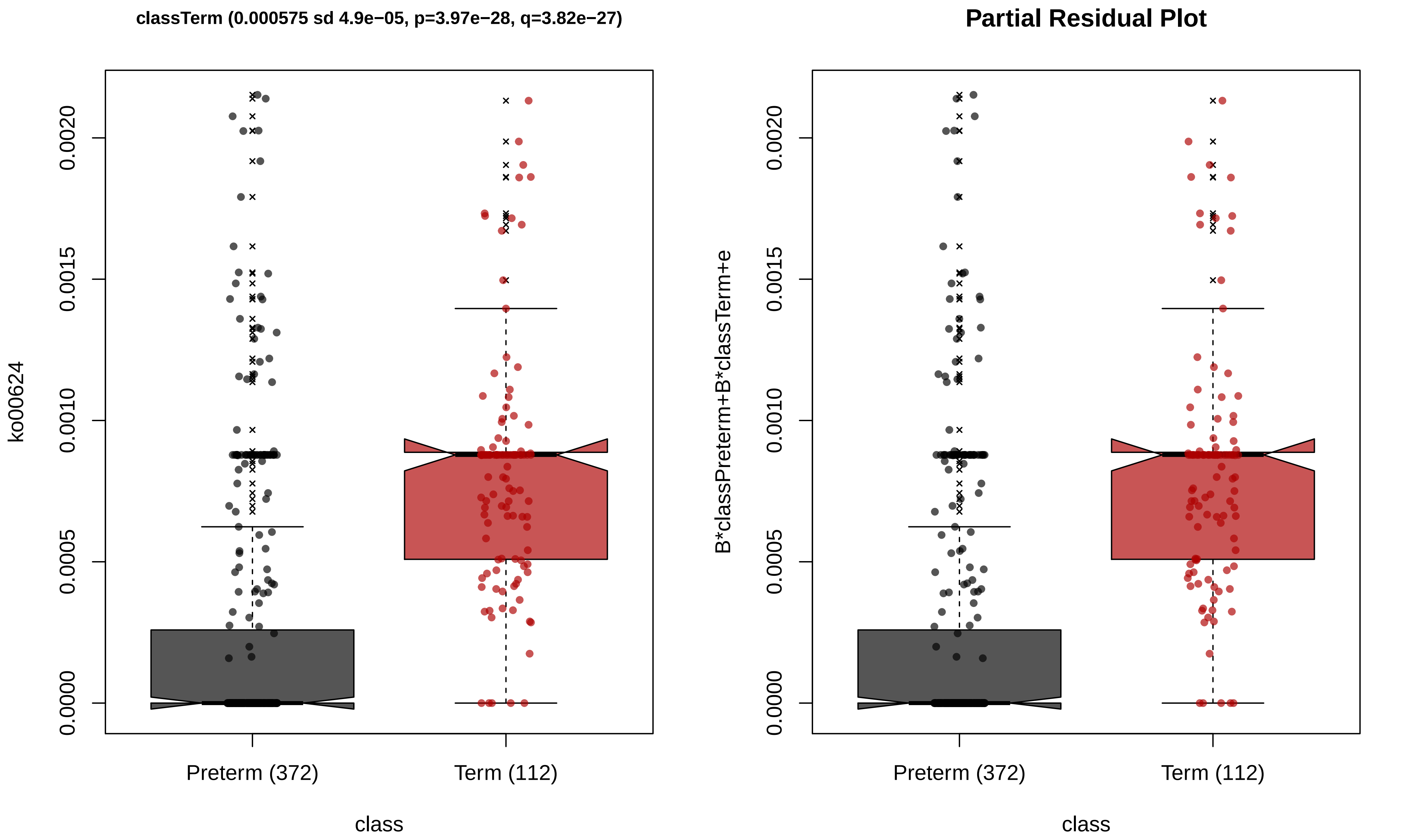

classTerm (0.000575 sd 4.9e−05, p=3.97e−28, q=3.82e−27)
Partial Residual Plot
ko00624
B*classPreterm+B*classTerm+e
0.0000
0.0005
0.0010
0.0015
0.0020
Preterm (372)
Term (112)
class

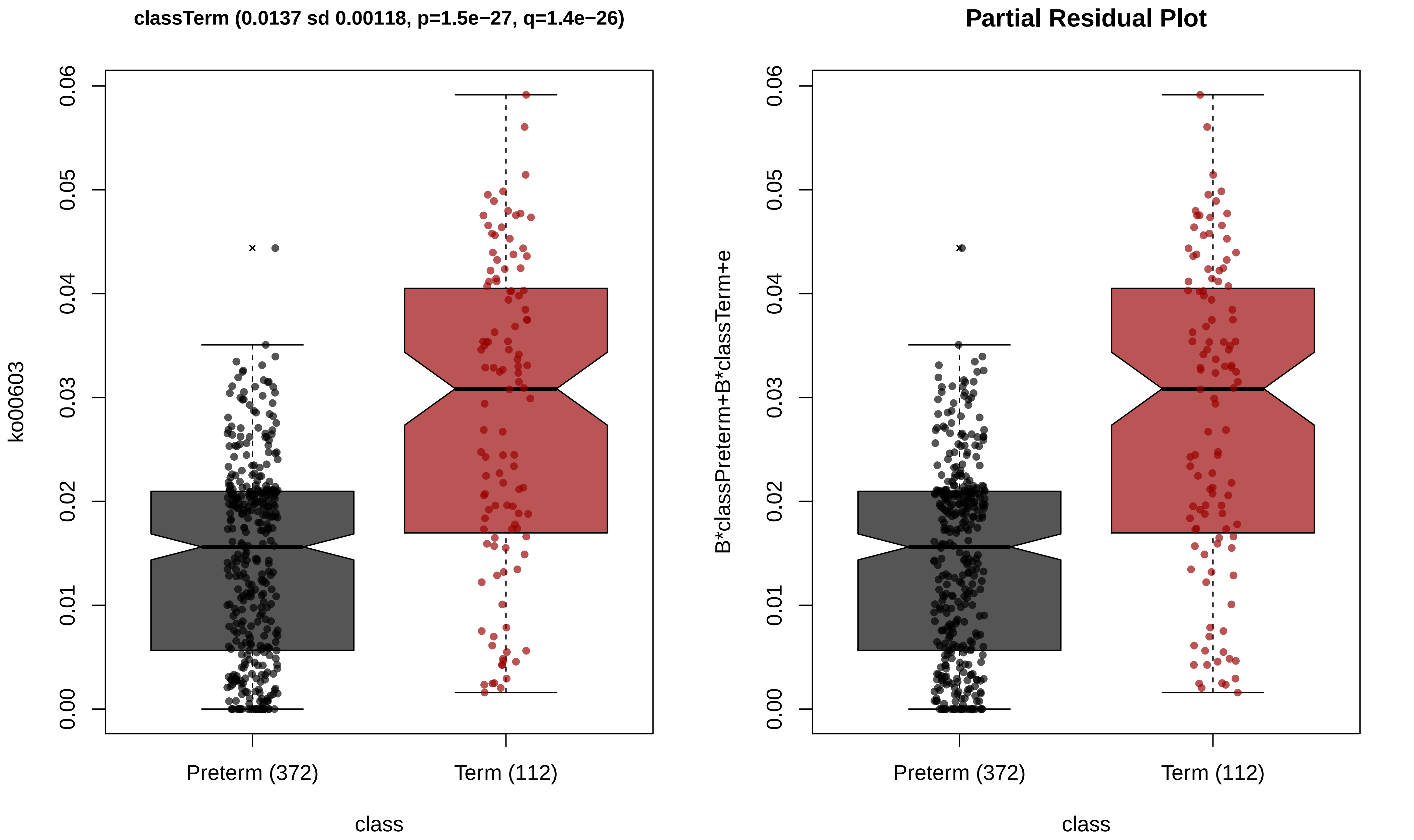
classTerm (0.0137 sd 0.00118, p=1.5e−27, q=1.4e−26)
ko00603
0.00
0.01
0.02
0.03
0.04
0.05
0.06
Preterm (372)
Term (112)
class
Partial Residual Plot
B*classPreterm+B*classTerm+e
0.00
0.01
0.02
0.03
0.04
0.05
0.06
Preterm (372)
Term (112)
class

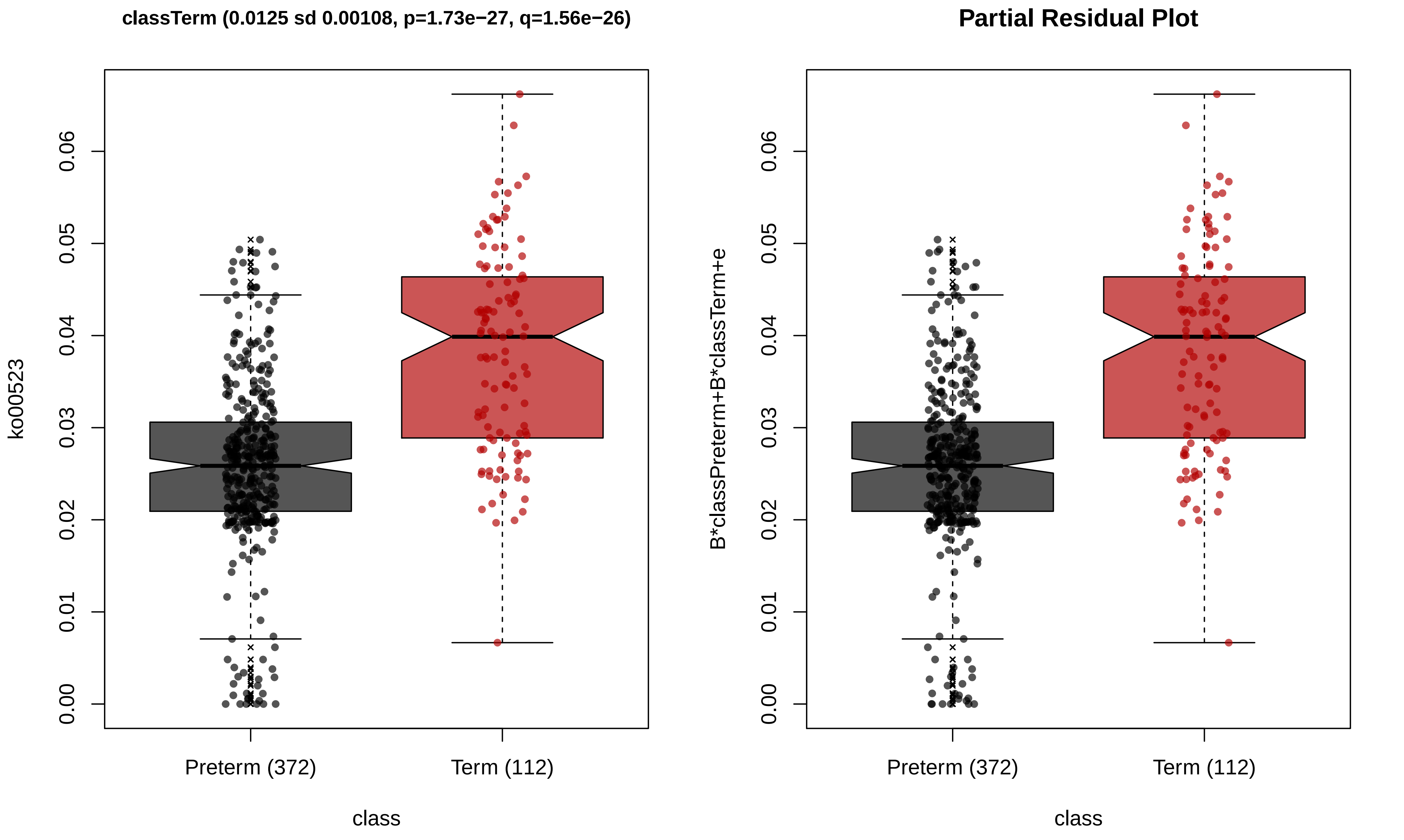
classTerm (0.0125 sd 0.00108, p=1.73e−27, q=1.56e−26)
ko00523
0.00
0.01
0.02
0.03
0.04
0.05
0.06
Preterm (372)
Term (112)
class
Partial Residual Plot
B*classPreterm+B*classTerm+e
0.00
0.01
0.02
0.03
0.04
0.05
0.06
Preterm (372)
Term (112)
class

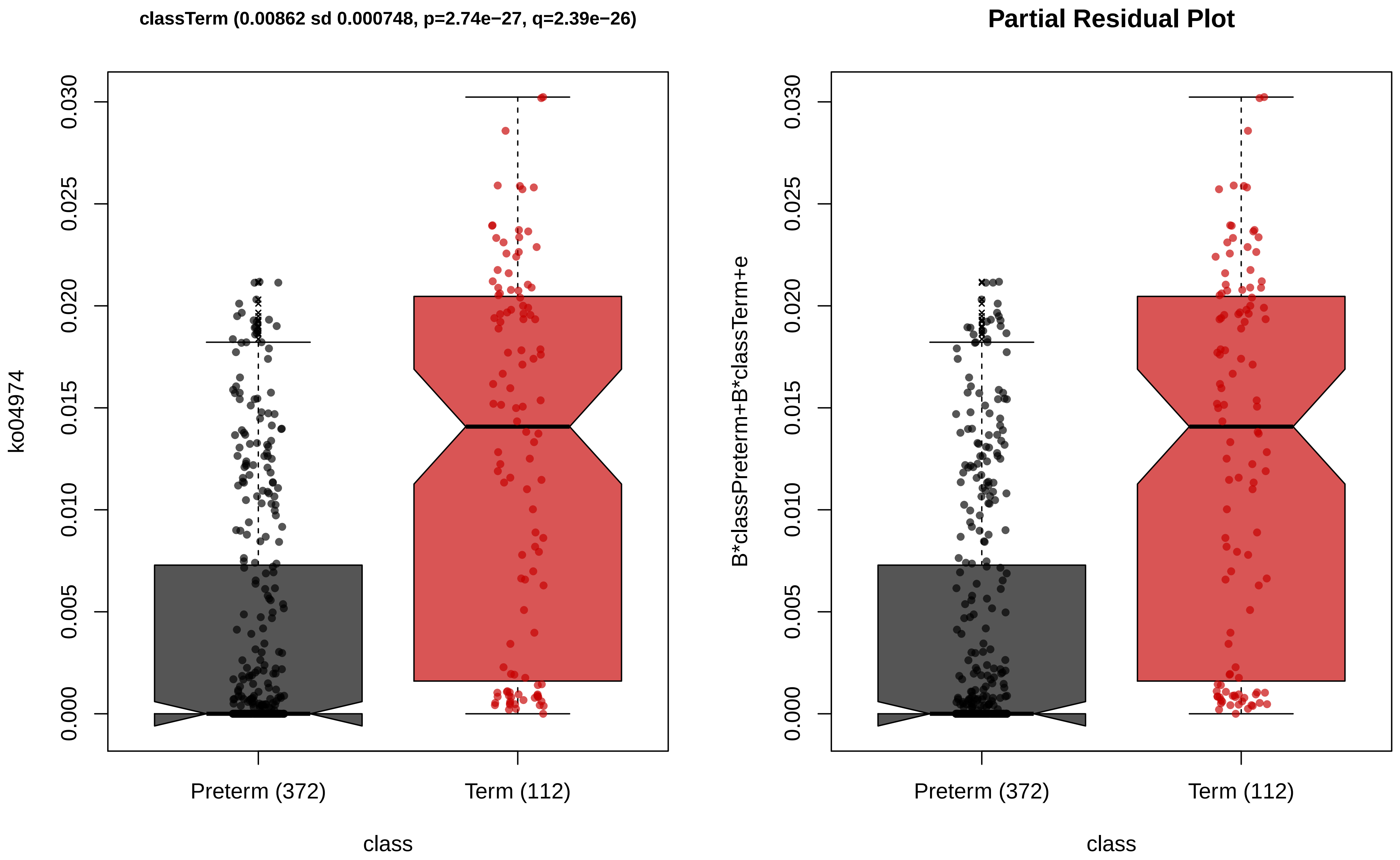
classTerm (0.00862 sd 0.000748, p=2.74e−27, q=2.39e−26)
ko04974
0.000
0.005
0.010
0.015
0.020
0.025
0.030
Preterm (372)
Term (112)
class
Partial Residual Plot
B*classPreterm+B*classTerm+e
0.000
0.005
0.010
0.015
0.020
0.025
0.030
Preterm (372)
Term (112)
class

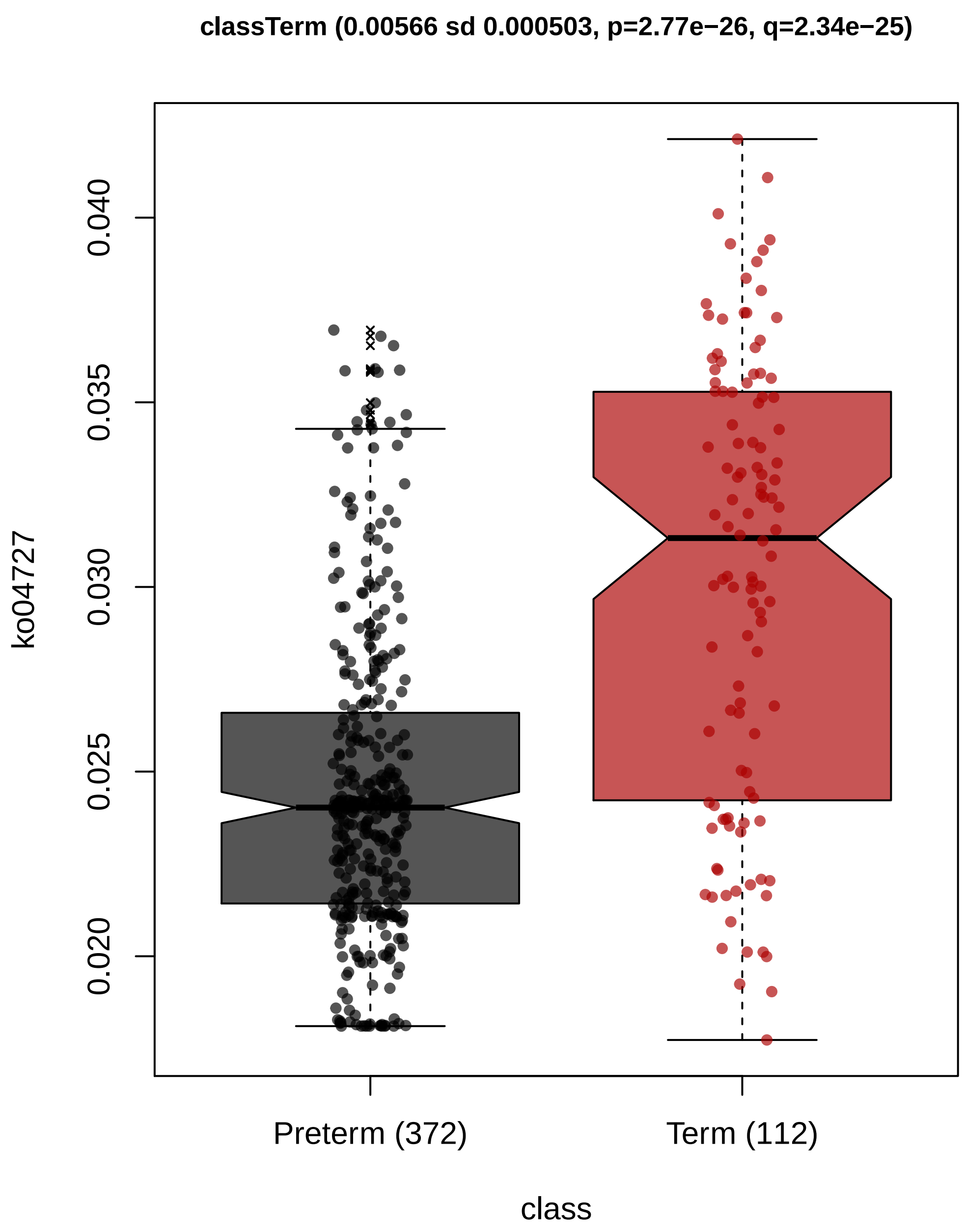
classTerm (0.00566 sd 0.000503, p=2.77e−26, q=2.34e−25)
ko04727
0.020
0.025
0.030
0.035
0.040
Preterm (372)
Term (112)
class

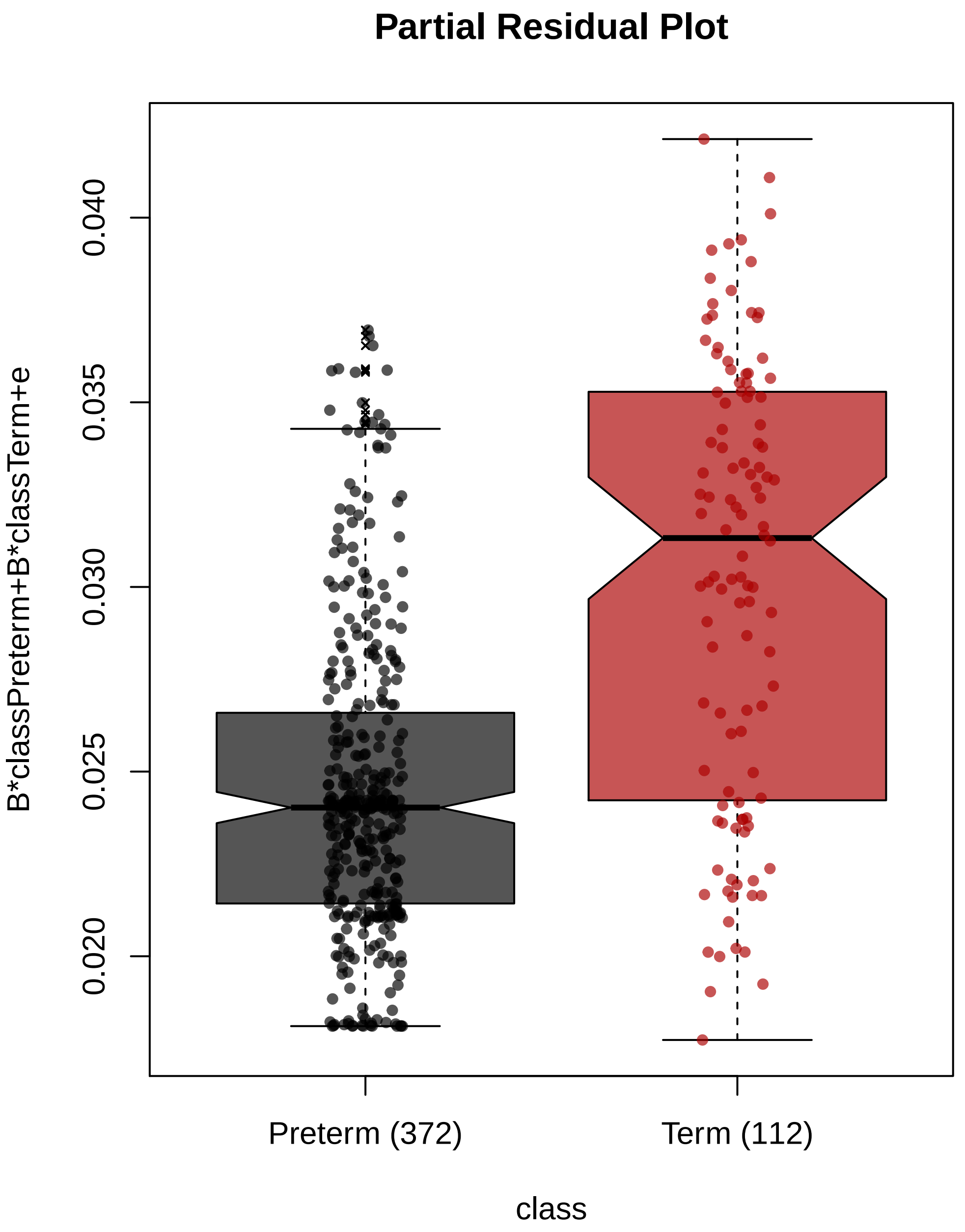
Partial Residual Plot
B*classPreterm+B*classTerm+e
0.020
0.025
0.030
0.035
0.040
Preterm (372)
Term (112)
class

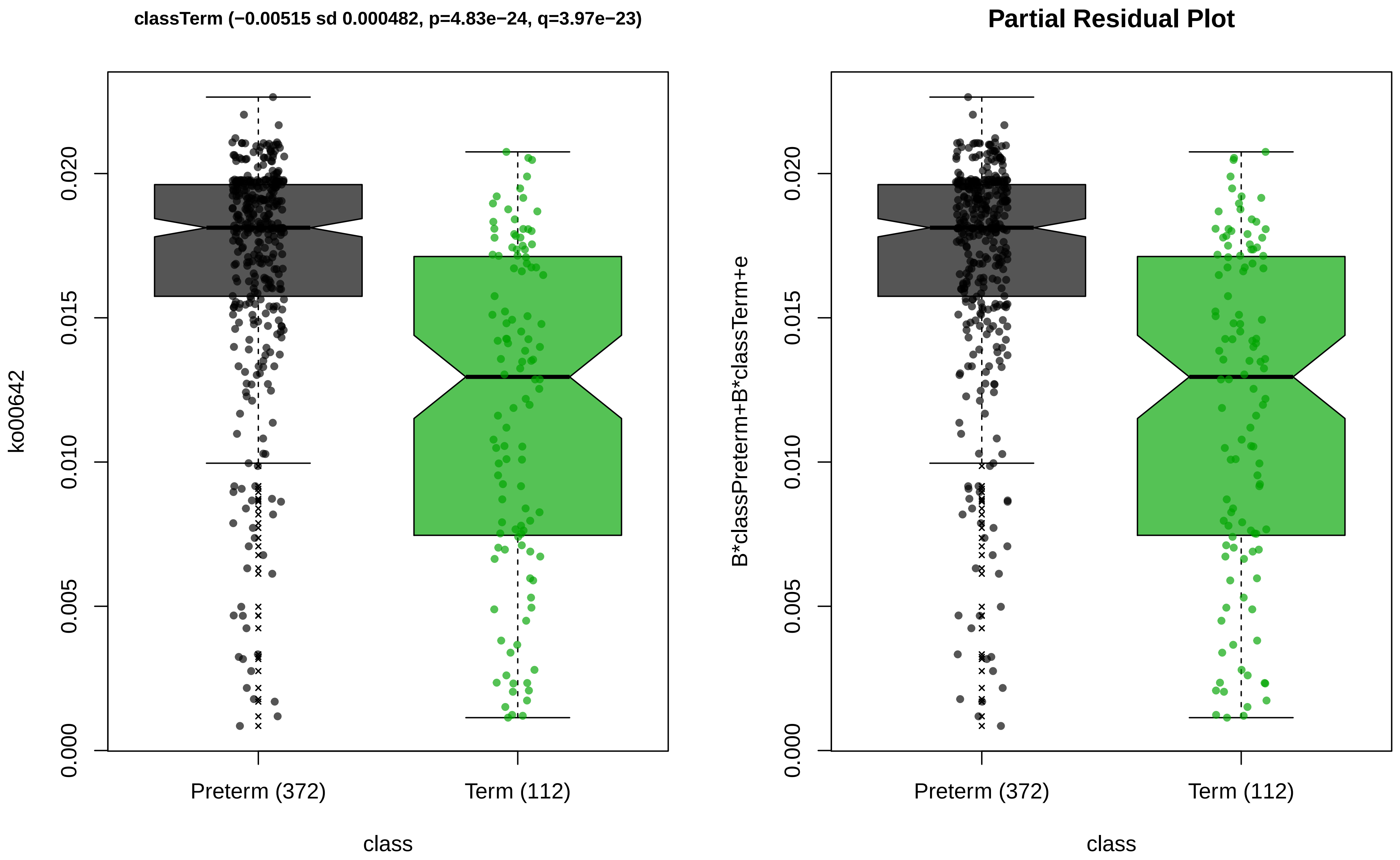

classTerm (−0.00515 sd 0.000482, p=4.83e−24, q=3.97e−23)
ko00642
0.000
0.005
0.010
0.015
0.020
Preterm (372)
Term (112)
class
Partial Residual Plot
B*classPreterm+B*classTerm+e
0.000
0.005
0.010
0.015
0.020
Preterm (372)
Term (112)
class

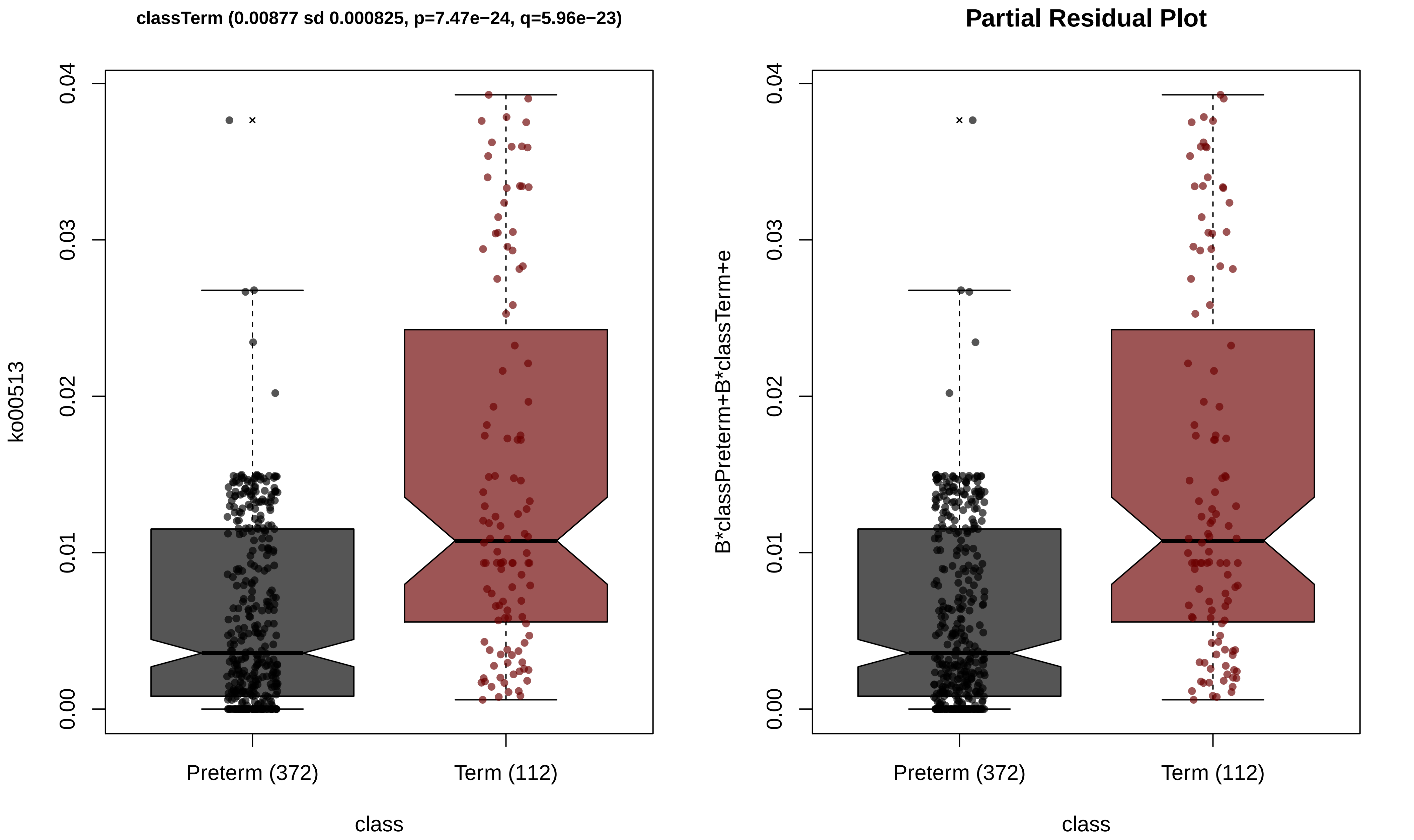
classTerm (0.00877 sd 0.000825, p=7.47e−24, q=5.96e−23)
Partial Residual Plot
ko00513
B*classPreterm+B*classTerm+e
0.00
0.01
0.02
0.03
0.04
Preterm (372)
Term (112)
class

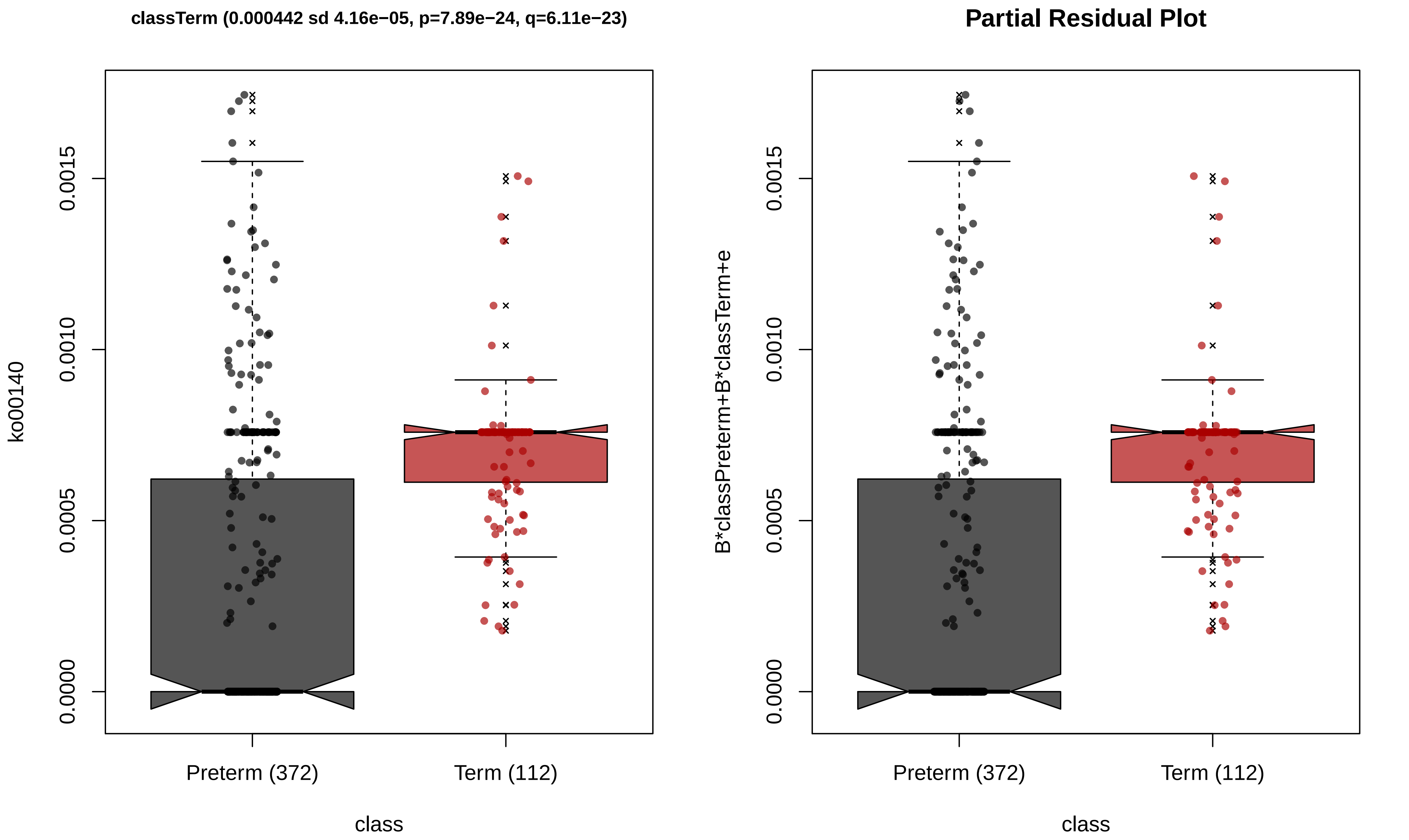

classTerm (0.000442 sd 4.16e−05, p=7.89e−24, q=6.11e−23)
ko00140
0.0000
0.0005
0.0010
0.0015
Preterm (372)
Term (112)
class
Partial Residual Plot
B*classPreterm+B*classTerm+e
0.0000
0.0005
0.0010
0.0015
Preterm (372)
Term (112)
class

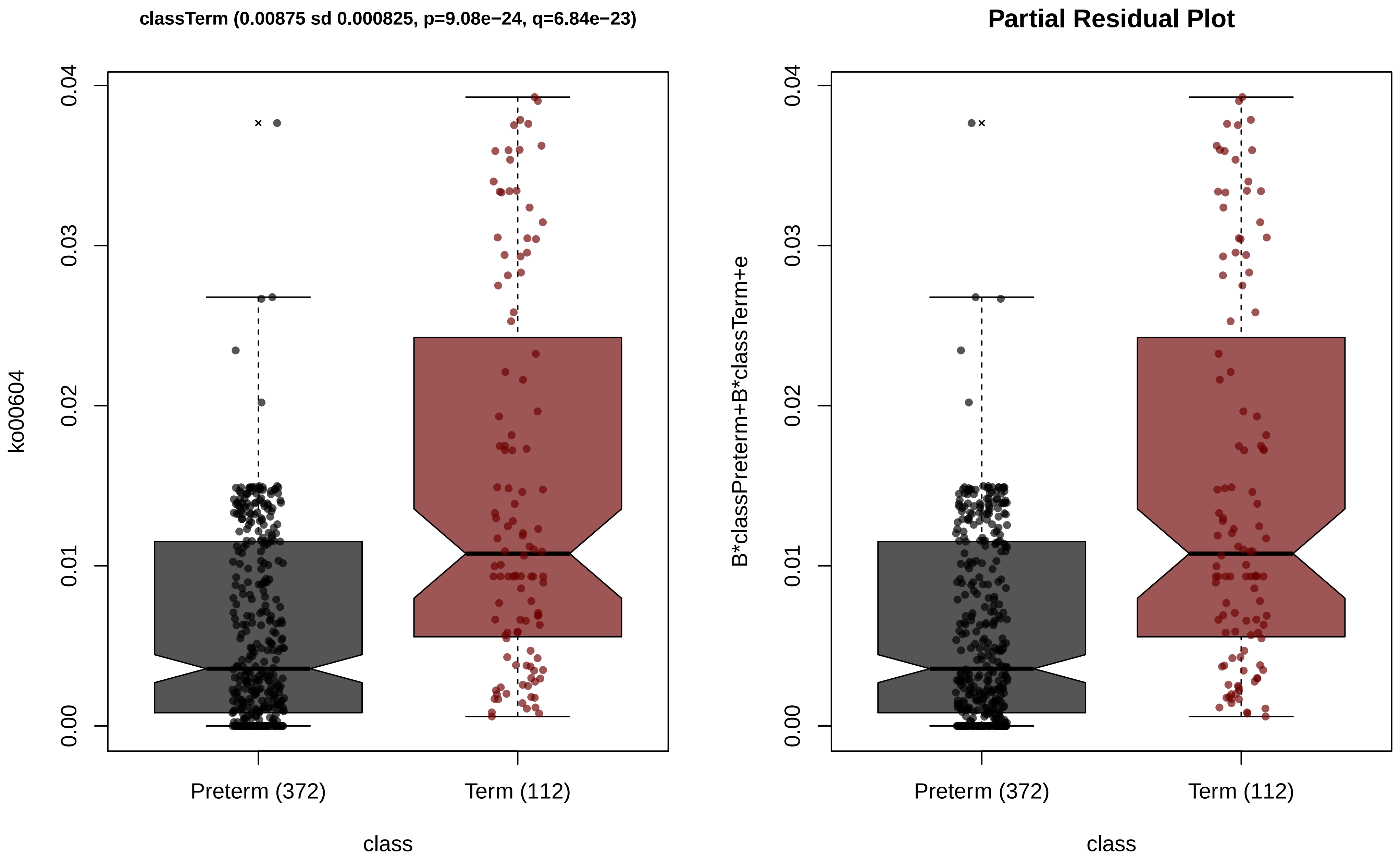
classTerm (0.00875 sd 0.000825, p=9.08e−24, q=6.84e−23)
Partial Residual Plot
ko00604
B*classPreterm+B*classTerm+e
0.00
0.01
0.02
0.03
0.04
Preterm (372)
Term (112)
class

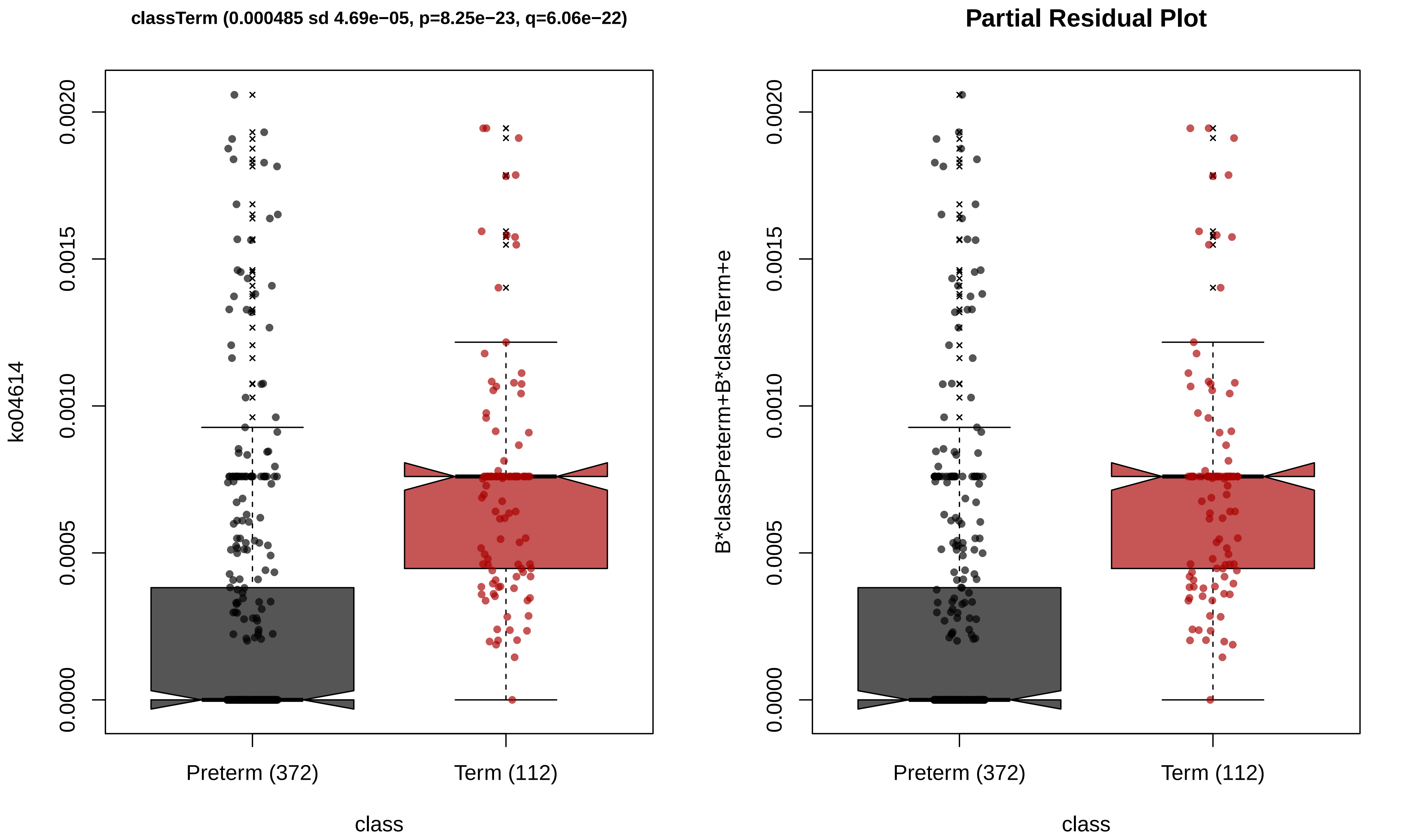

classTerm (0.000485 sd 4.69e−05, p=8.25e−23, q=6.06e−22)
Partial Residual Plot
ko04614
B*classPreterm+B*classTerm+e
0.0000
0.0005
0.0010
0.0015
0.0020
Preterm (372)
Term (112)
class

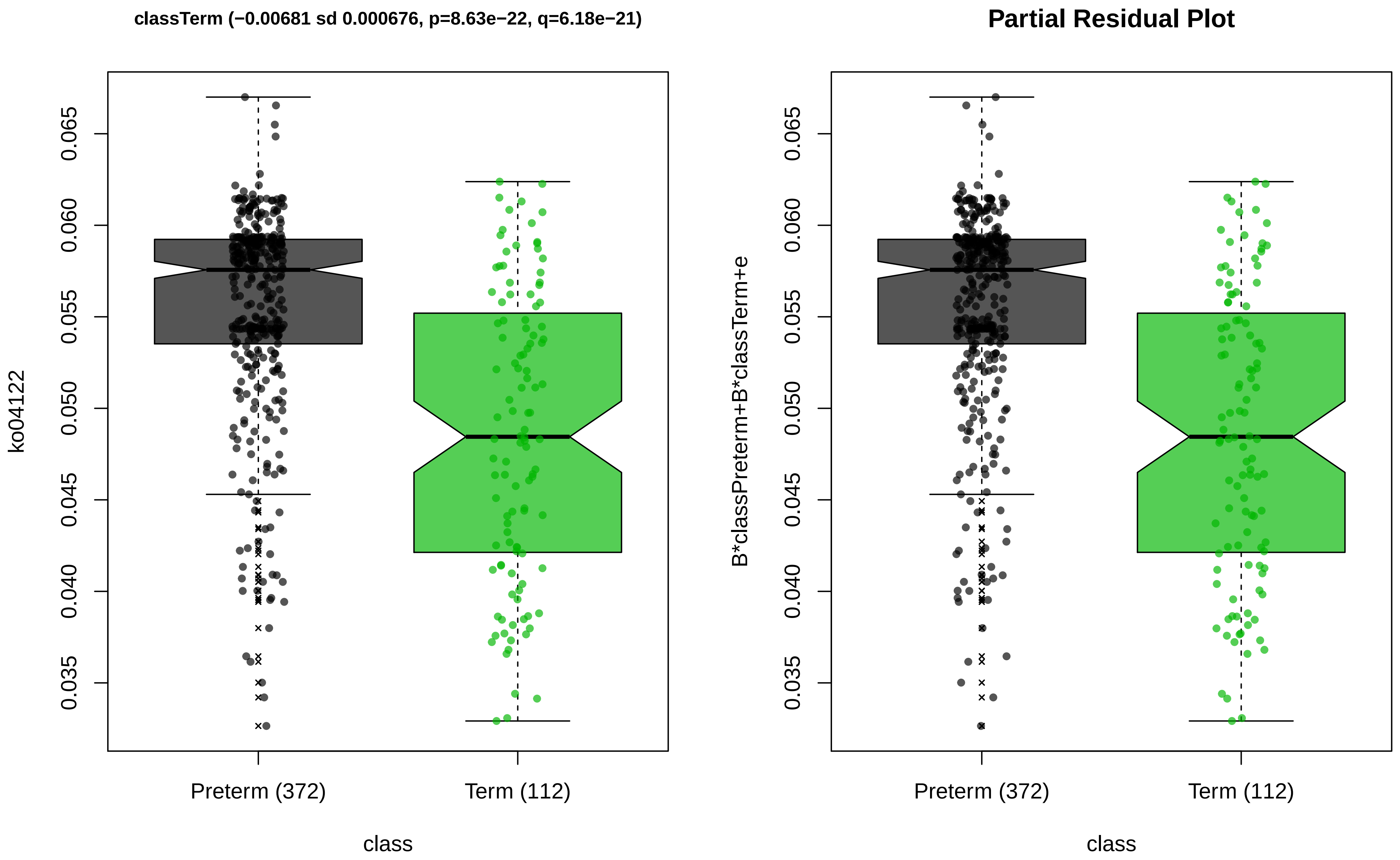

classTerm (−0.00681 sd 0.000676, p=8.63e−22, q=6.18e−21)
Partial Residual Plot
ko04122
B*classPreterm+B*classTerm+e
0.035
0.040
0.045
0.050
0.055
0.060
0.065
Preterm (372)
Term (112)
class

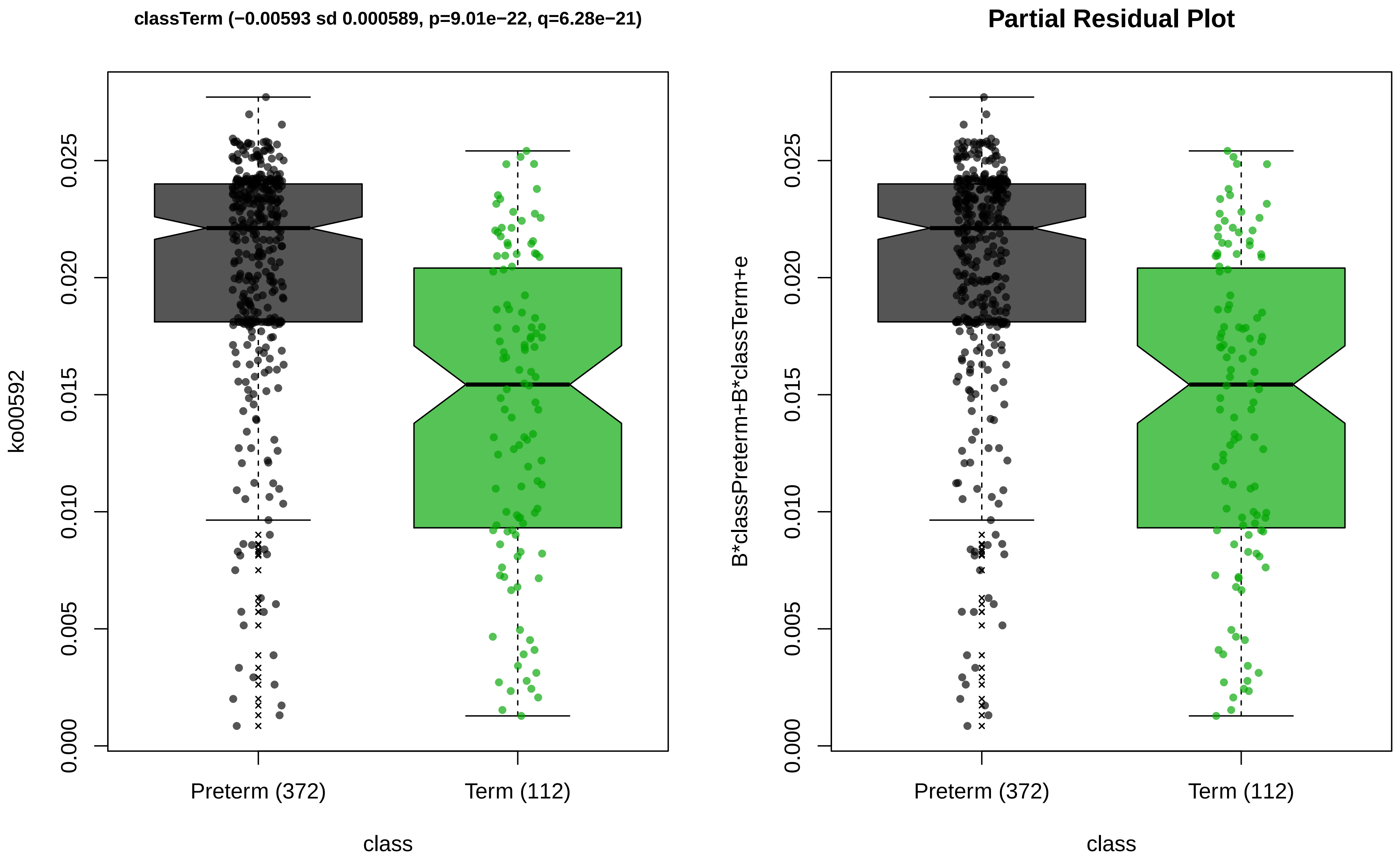

classTerm (−0.00593 sd 0.000589, p=9.01e−22, q=6.28e−21)
ko00592
0.000
0.005
0.010
0.015
0.020
0.025
Preterm (372)
Term (112)
class
Partial Residual Plot
B*classPreterm+B*classTerm+e
0.000
0.005
0.010
0.015
0.020
0.025
Preterm (372)
Term (112)
class

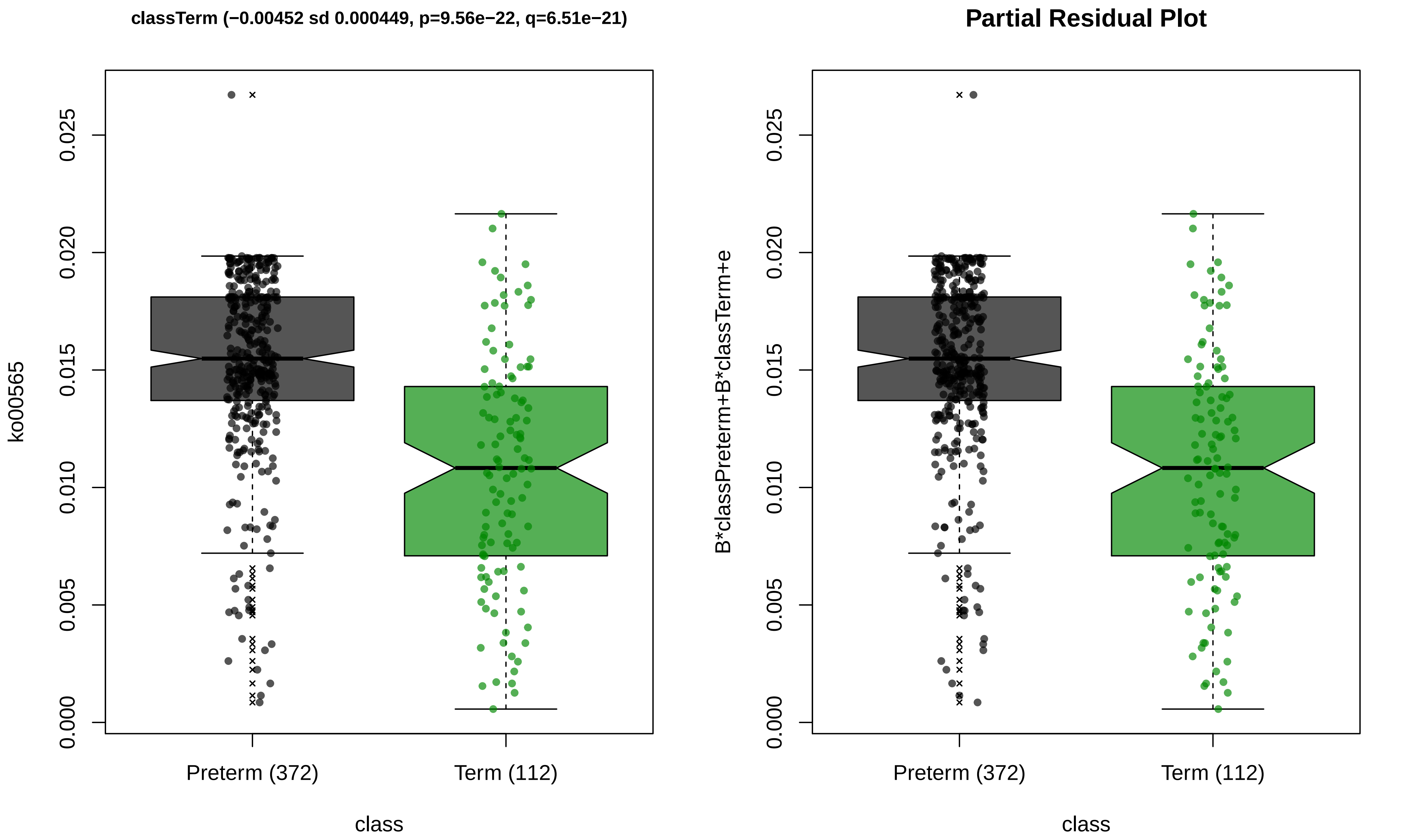

classTerm (−0.00452 sd 0.000449, p=9.56e−22, q=6.51e−21)
Partial Residual Plot
ko00565
B*classPreterm+B*classTerm+e
0.000
0.005
0.010
0.015
0.020
0.025
Preterm (372)
Term (112)
class

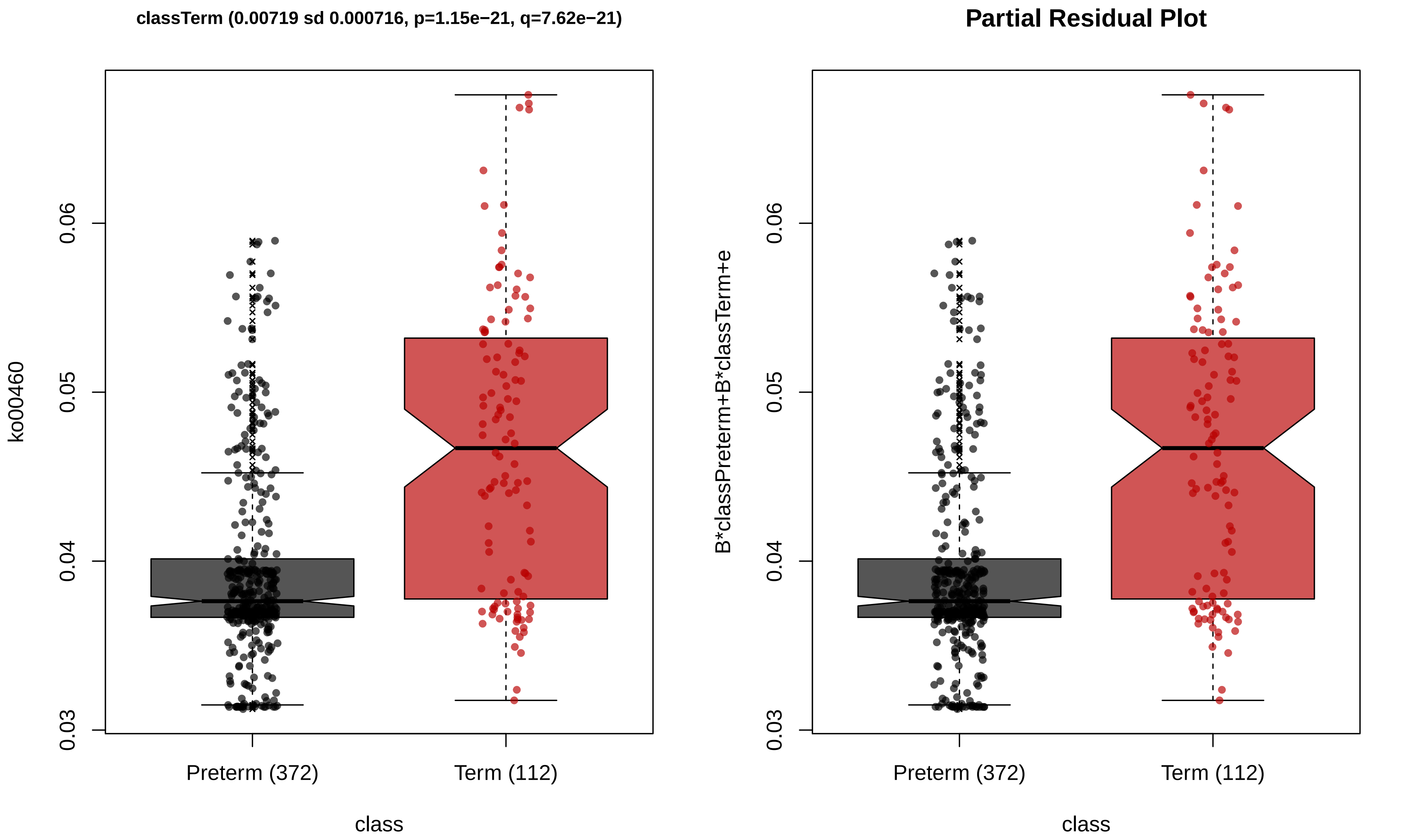

classTerm (0.00719 sd 0.000716, p=1.15e−21, q=7.62e−21)
ko00460
0.03
0.04
0.05
0.06
Preterm (372)
Term (112)
class
Partial Residual Plot
B*classPreterm+B*classTerm+e
0.03
0.04
0.05
0.06
Preterm (372)
Term (112)
class

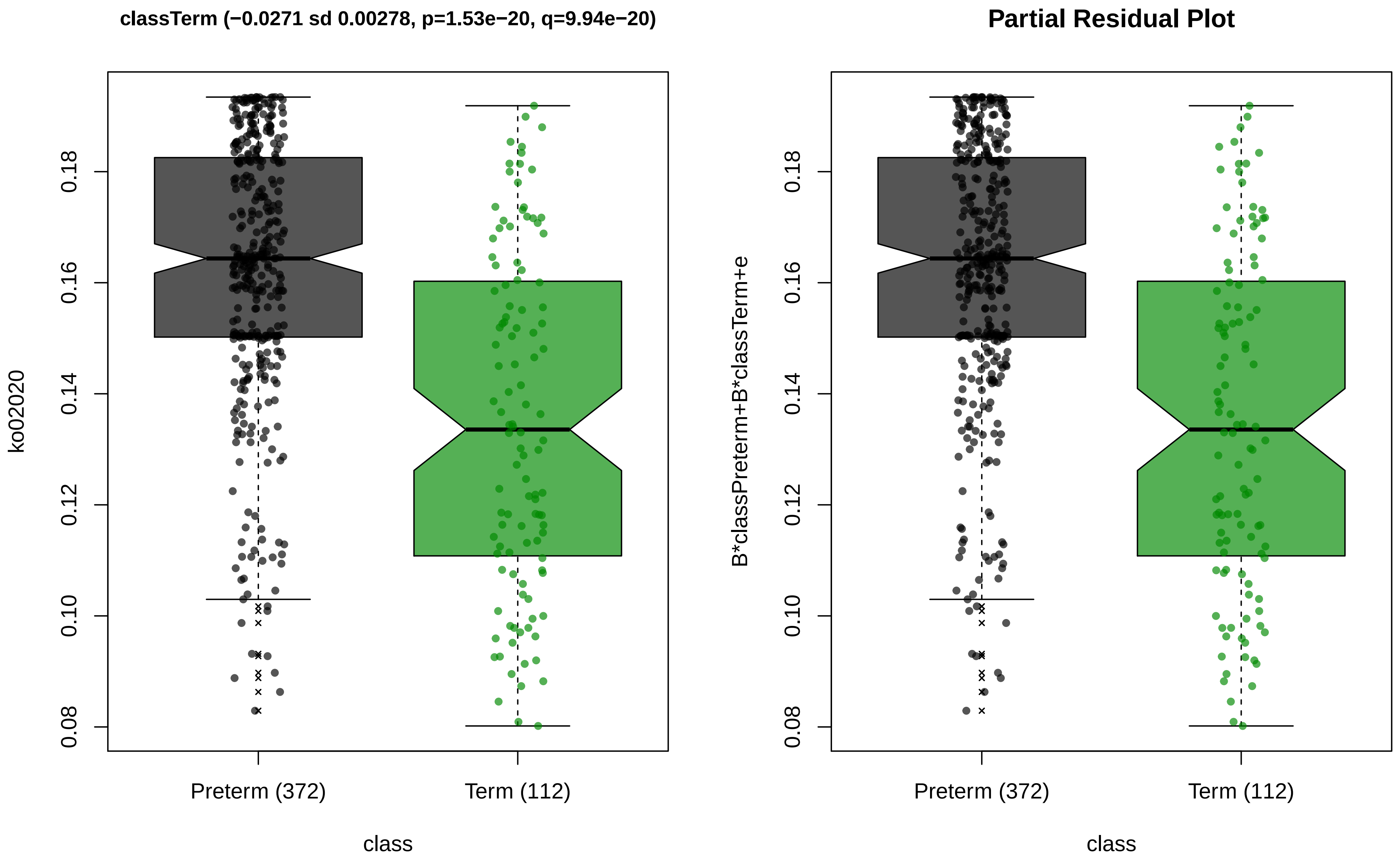

classTerm (−0.0271 sd 0.00278, p=1.53e−20, q=9.94e−20)
Partial Residual Plot
ko02020
B*classPreterm+B*classTerm+e
0.08
0.10
0.12
0.14
0.16
0.18
Preterm (372)
Term (112)
class

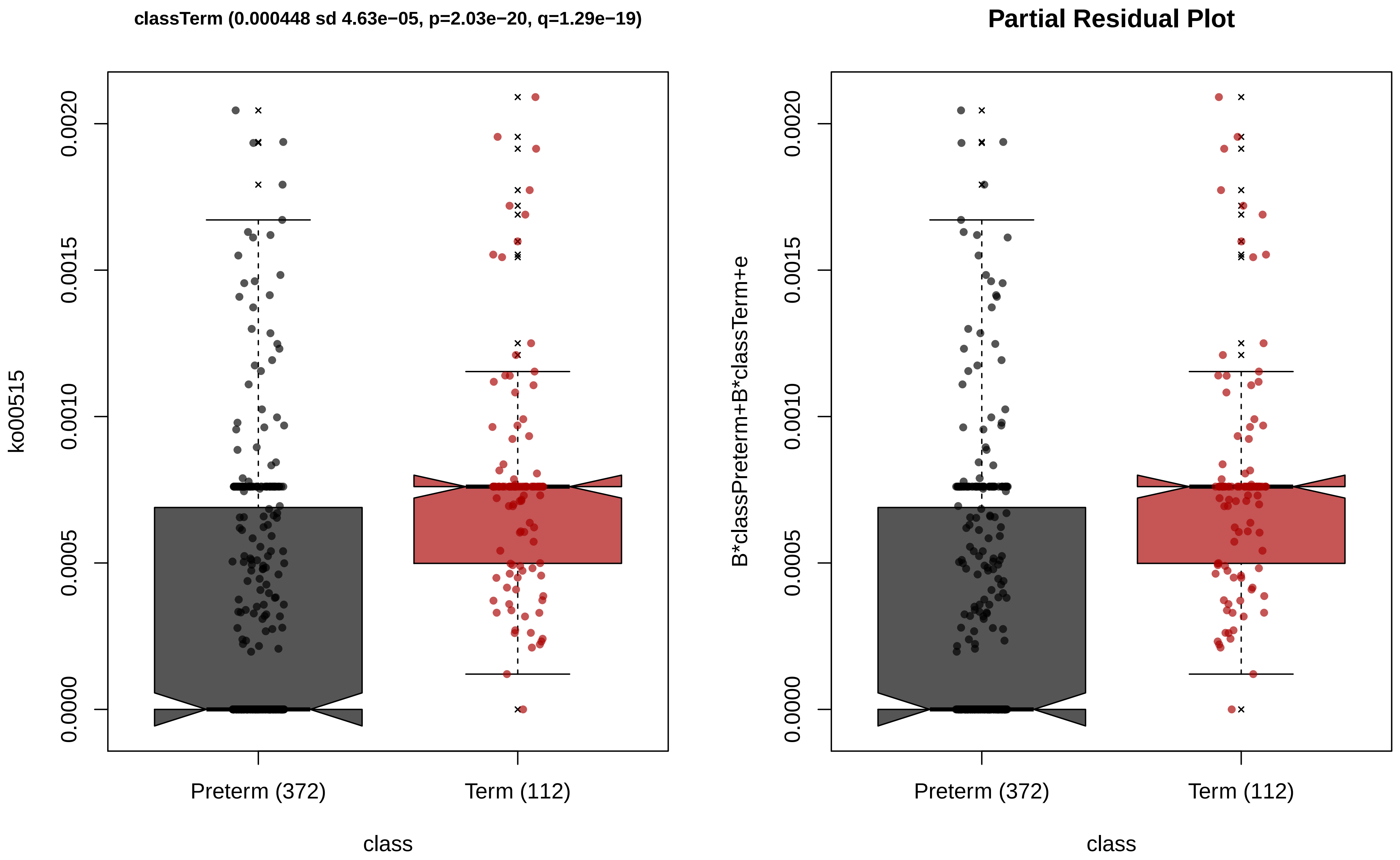

classTerm (0.000448 sd 4.63e−05, p=2.03e−20, q=1.29e−19)
ko00515
0.0000
0.0005
0.0010
0.0015
0.0020
Preterm (372)
Term (112)
class
Partial Residual Plot
B*classPreterm+B*classTerm+e
0.0000
0.0005
0.0010
0.0015
0.0020
Preterm (372)
Term (112)
class

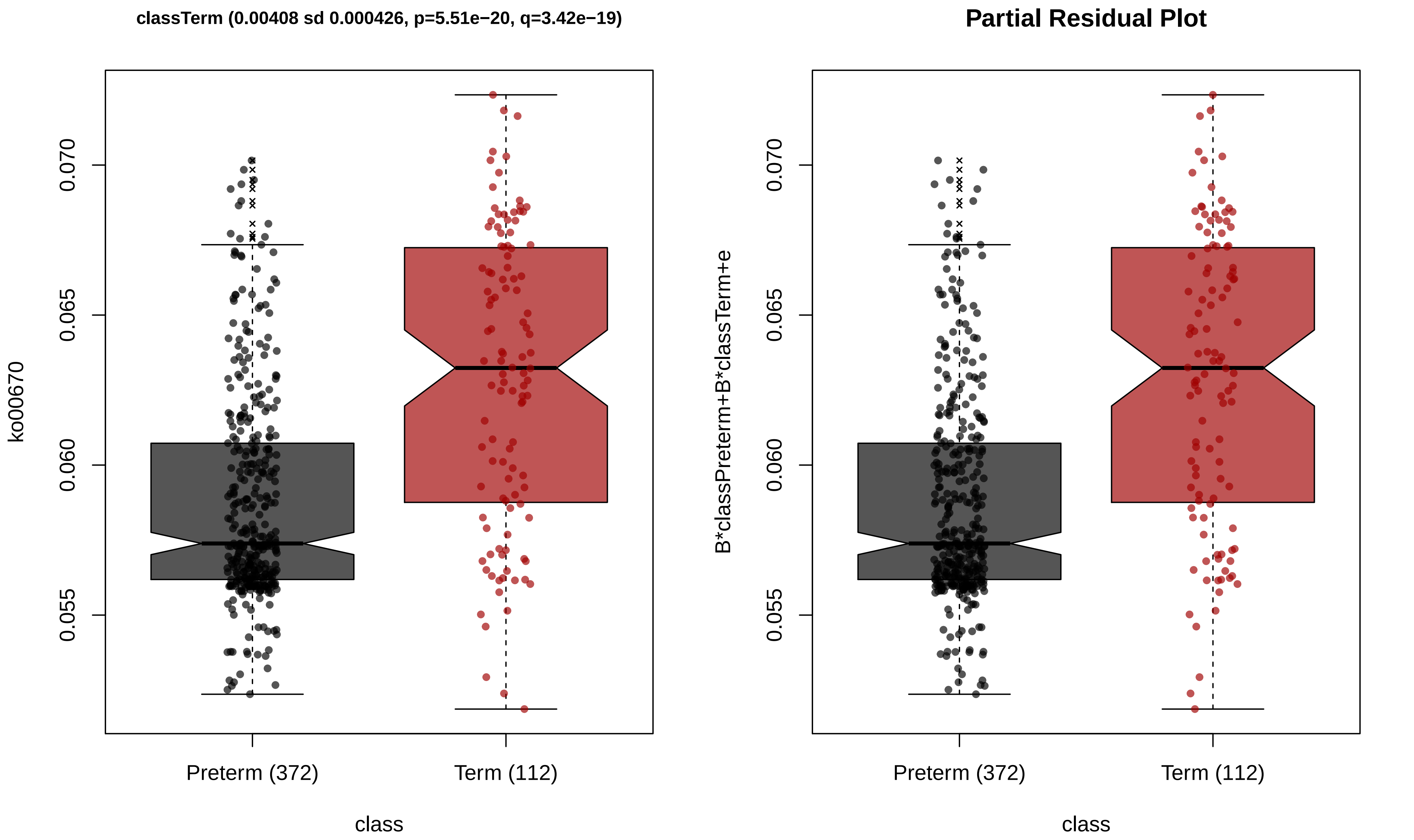

classTerm (0.00408 sd 0.000426, p=5.51e−20, q=3.42e−19)
ko00670
0.055
0.060
0.065
0.070
Preterm (372)
Term (112)
class
Partial Residual Plot
B*classPreterm+B*classTerm+e
0.055
0.060
0.065
0.070
Preterm (372)
Term (112)
class

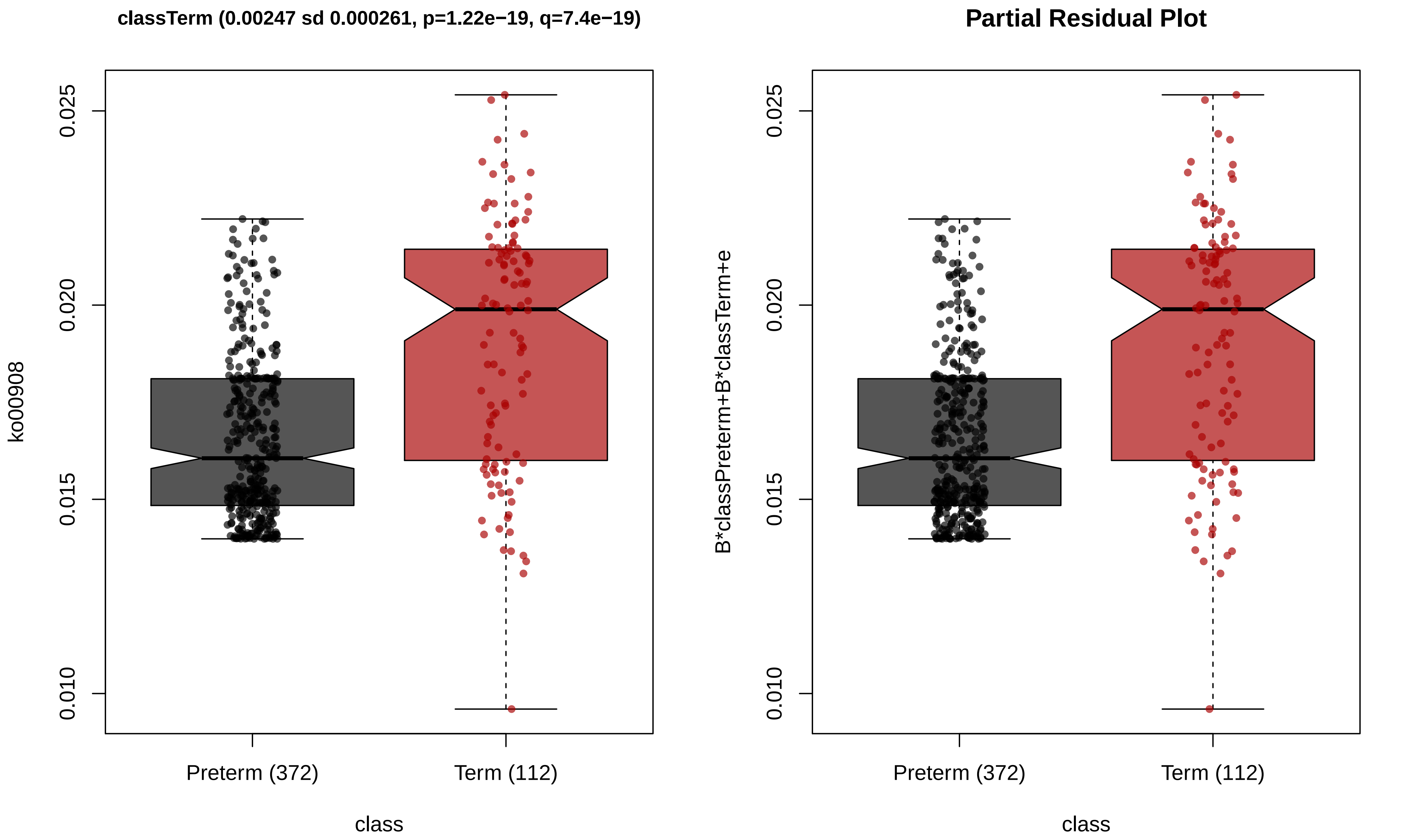
classTerm (0.00247 sd 0.000261, p=1.22e−19, q=7.4e−19)
Partial Residual Plot
ko00908
B*classPreterm+B*classTerm+e
0.010
0.015
0.020
0.025
Preterm (372)
Term (112)
class

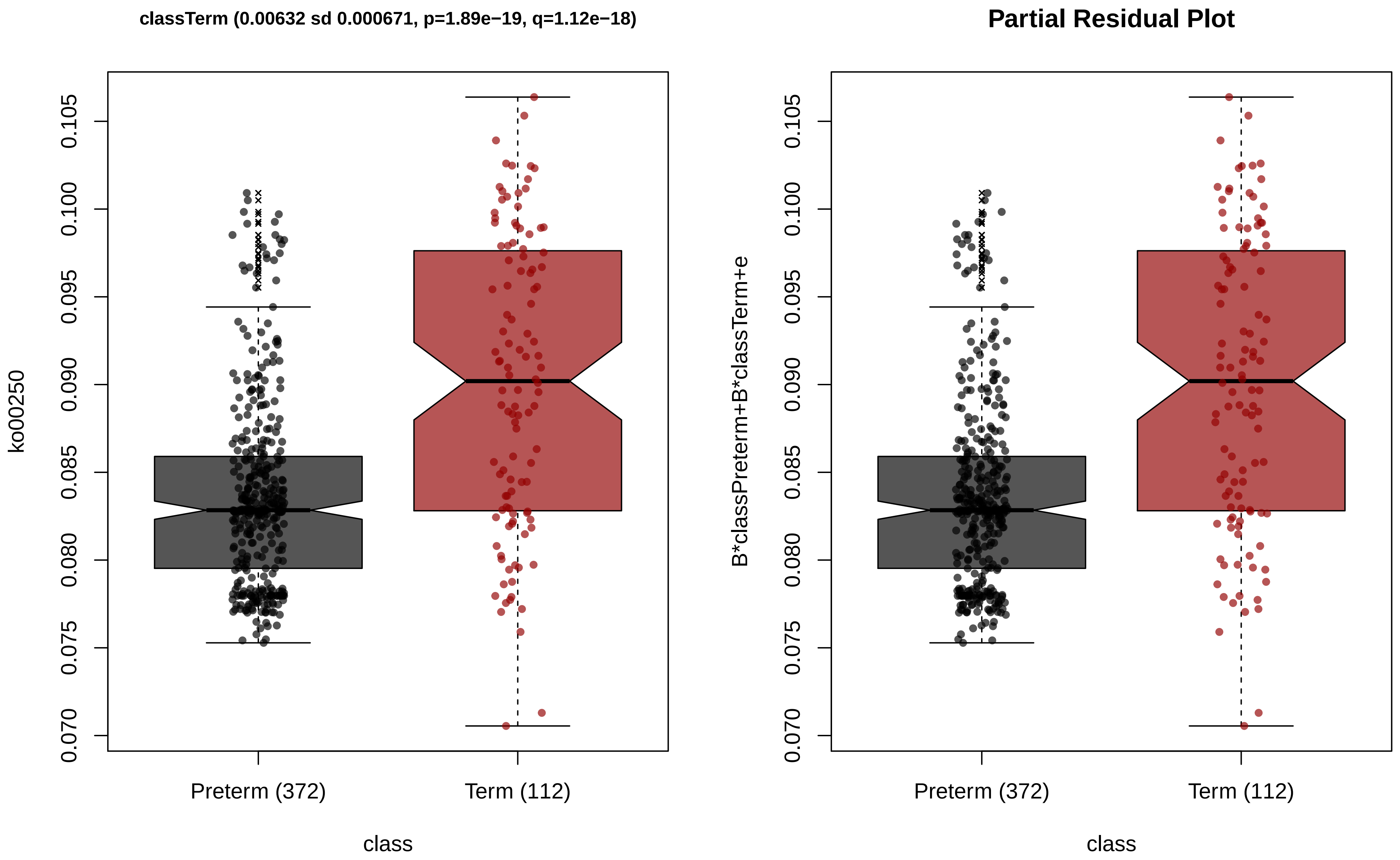
classTerm (0.00632 sd 0.000671, p=1.89e−19, q=1.12e−18)
ko00250
0.070
0.075
0.080
0.085
0.090
0.095
0.100
0.105
Preterm (372)
Term (112)
class
Partial Residual Plot
B*classPreterm+B*classTerm+e
0.070
0.075
0.080
0.085
0.090
0.095
0.100
0.105
Preterm (372)
Term (112)
class

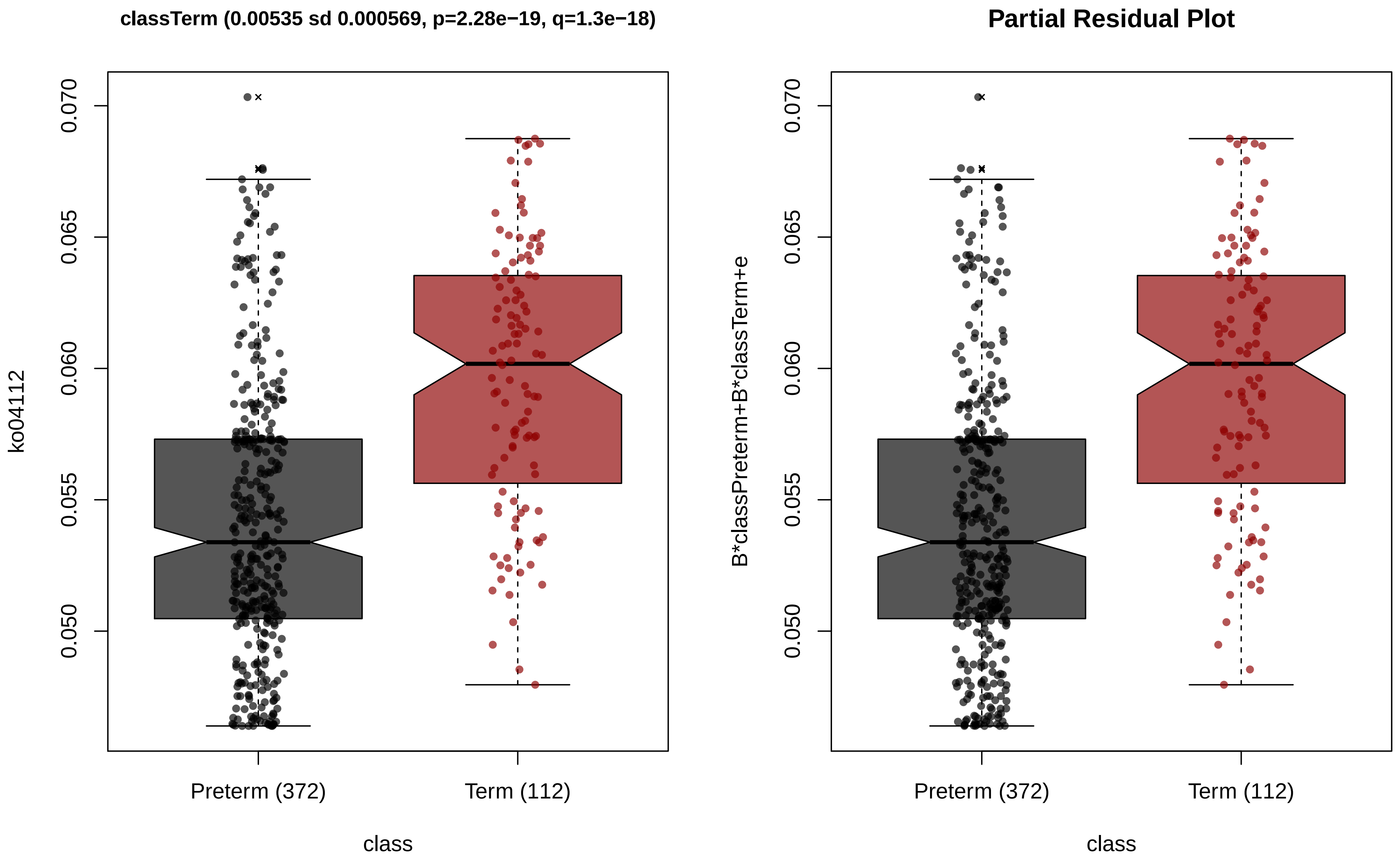

classTerm (0.00535 sd 0.000569, p=2.28e−19, q=1.3e−18)
ko04112
0.070
0.065
0.060
0.055
0.050
Preterm (372)
Term (112)
class
Partial Residual Plot
B*classPreterm+B*classTerm+e
0.070
0.065
0.060
0.055
0.050
Preterm (372)
Term (112)
class

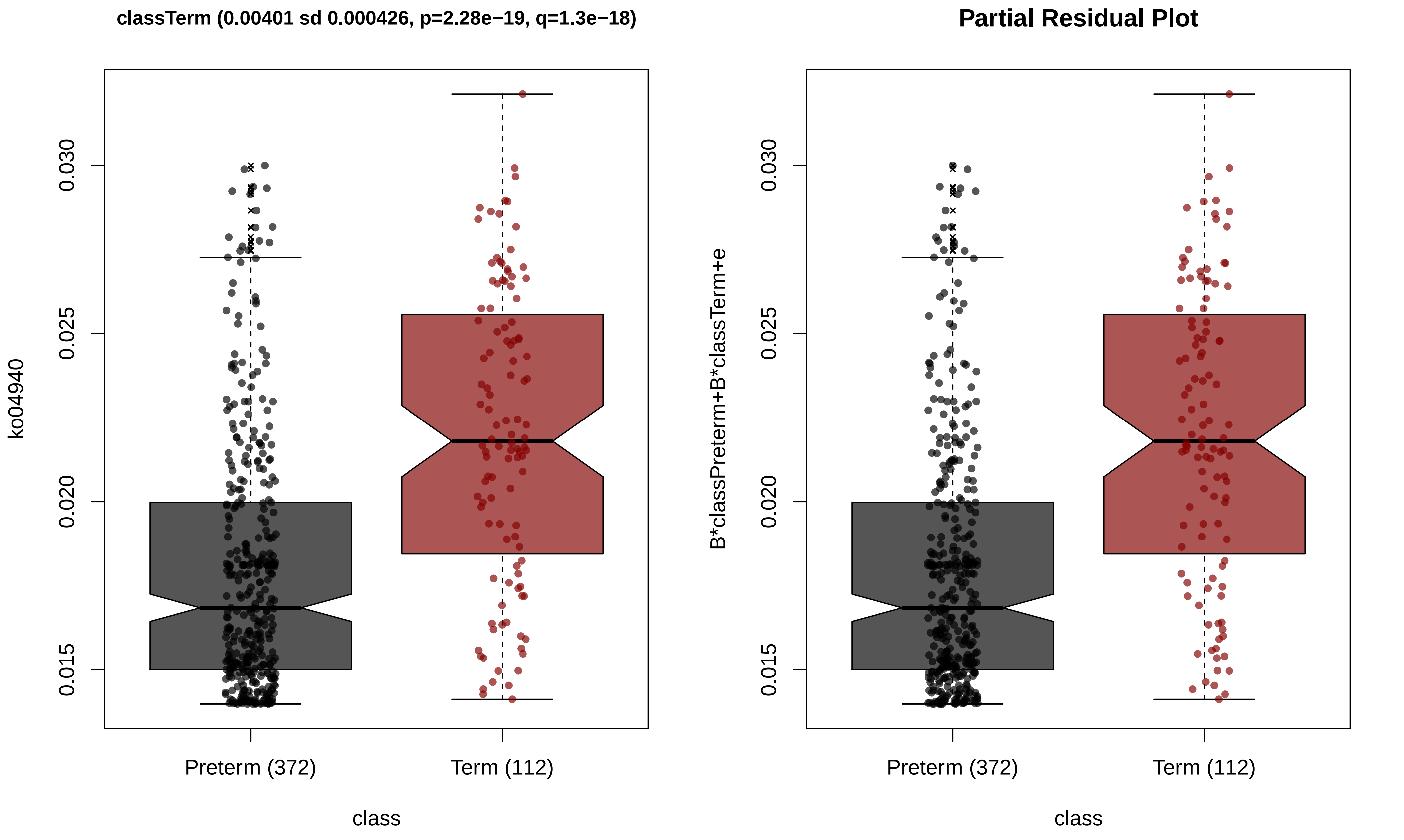

classTerm (0.00401 sd 0.000426, p=2.28e−19, q=1.3e−18)
Partial Residual Plot
ko04940
B*classPreterm+B*classTerm+e
0.030
0.025
0.020
0.015
Preterm (372)
Term (112)
class

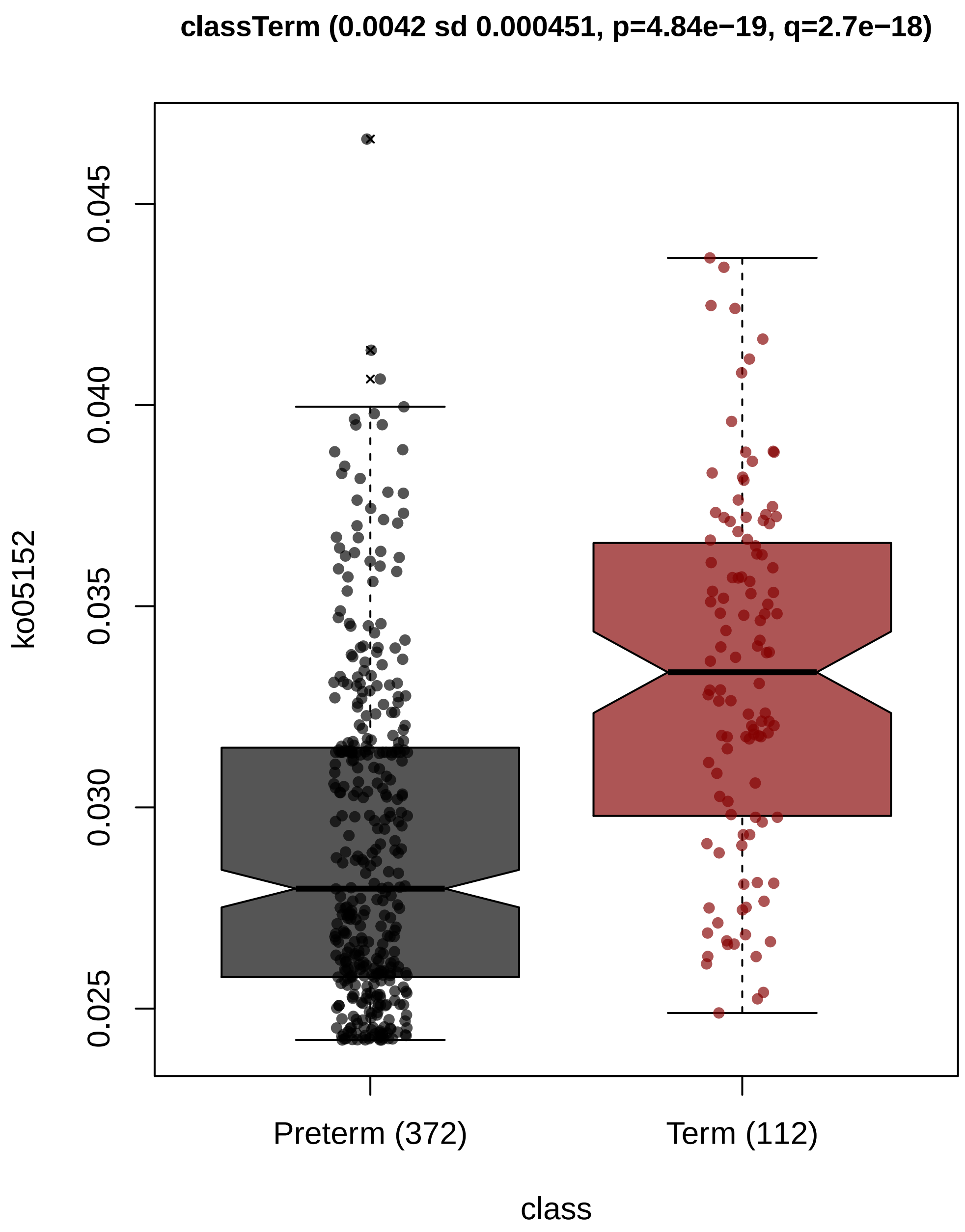
classTerm (0.0042 sd 0.000451, p=4.84e−19, q=2.7e−18)
ko05152
0.025
0.030
0.035
0.040
0.045
Preterm (372)
Term (112)
class

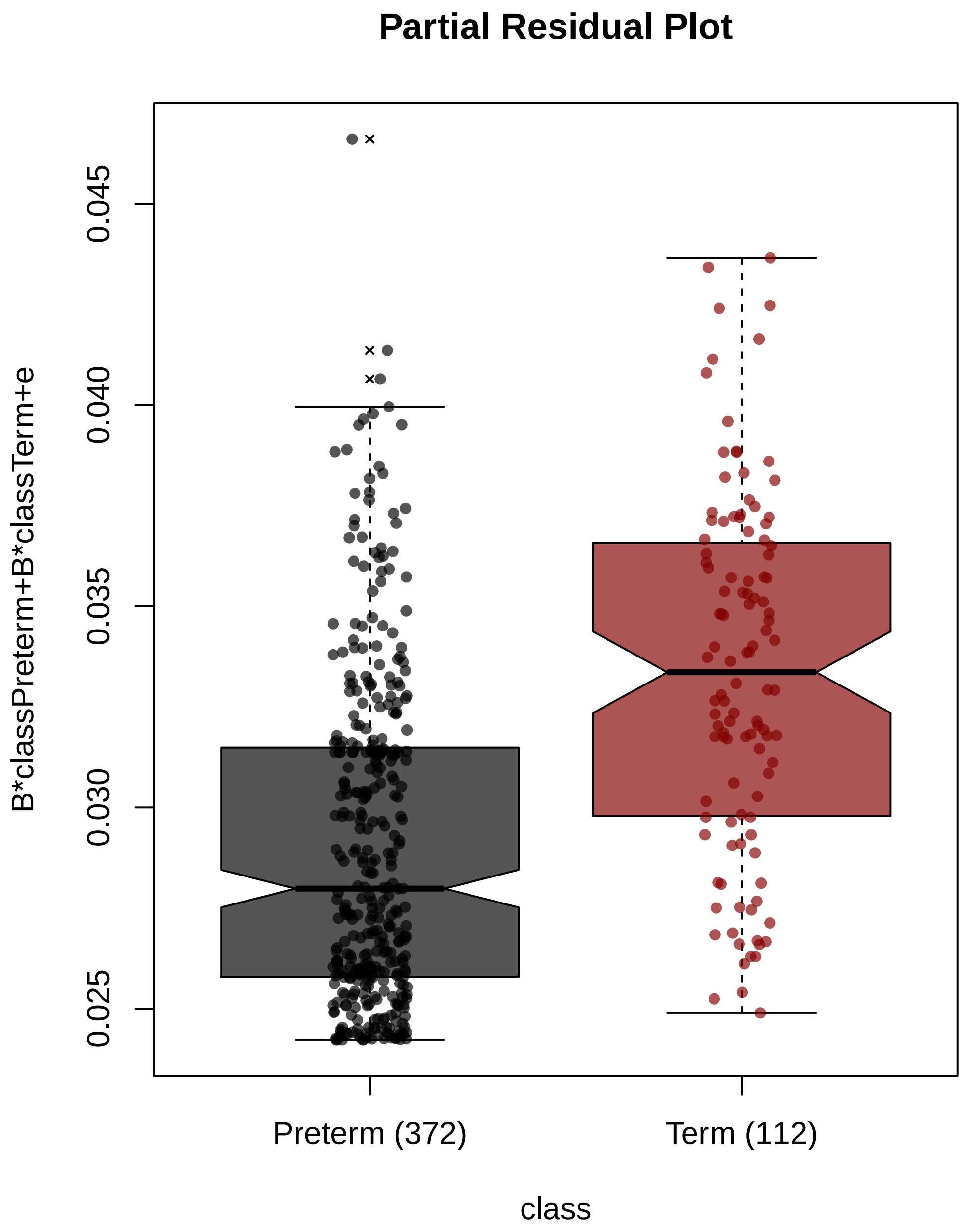
Partial Residual Plot
B*classPreterm+B*classTerm+e
0.025
0.030
0.035
0.040
0.045
Preterm (372)
Term (112)
class

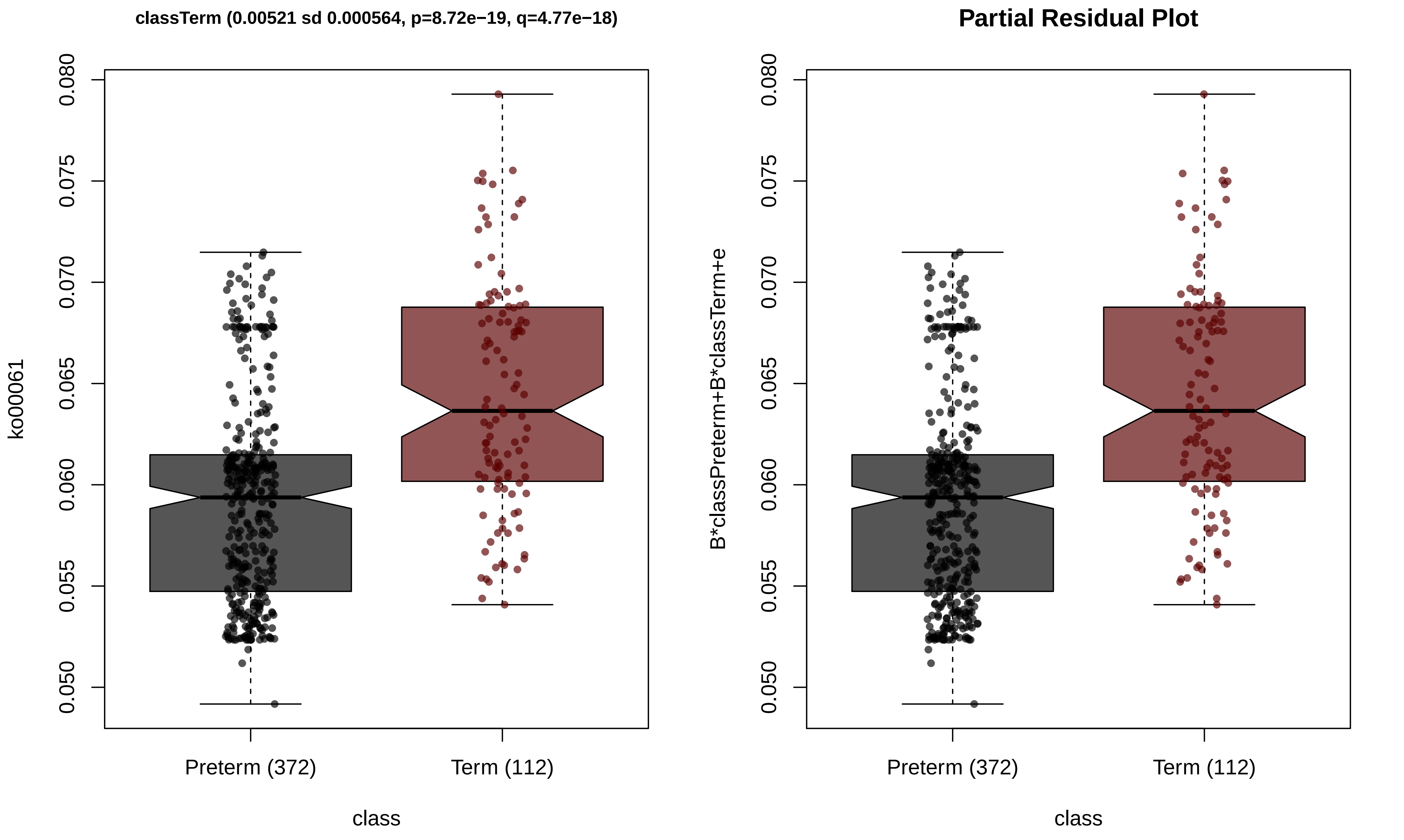

classTerm (0.00521 sd 0.000564, p=8.72e−19, q=4.77e−18)
ko00061
0.080
0.075
0.070
0.065
0.060
0.055
0.050
Preterm (372)
Term (112)
class
Partial Residual Plot
B*classPreterm+B*classTerm+e
0.080
0.075
0.070
0.065
0.060
0.055
0.050
Preterm (372)
Term (112)
class

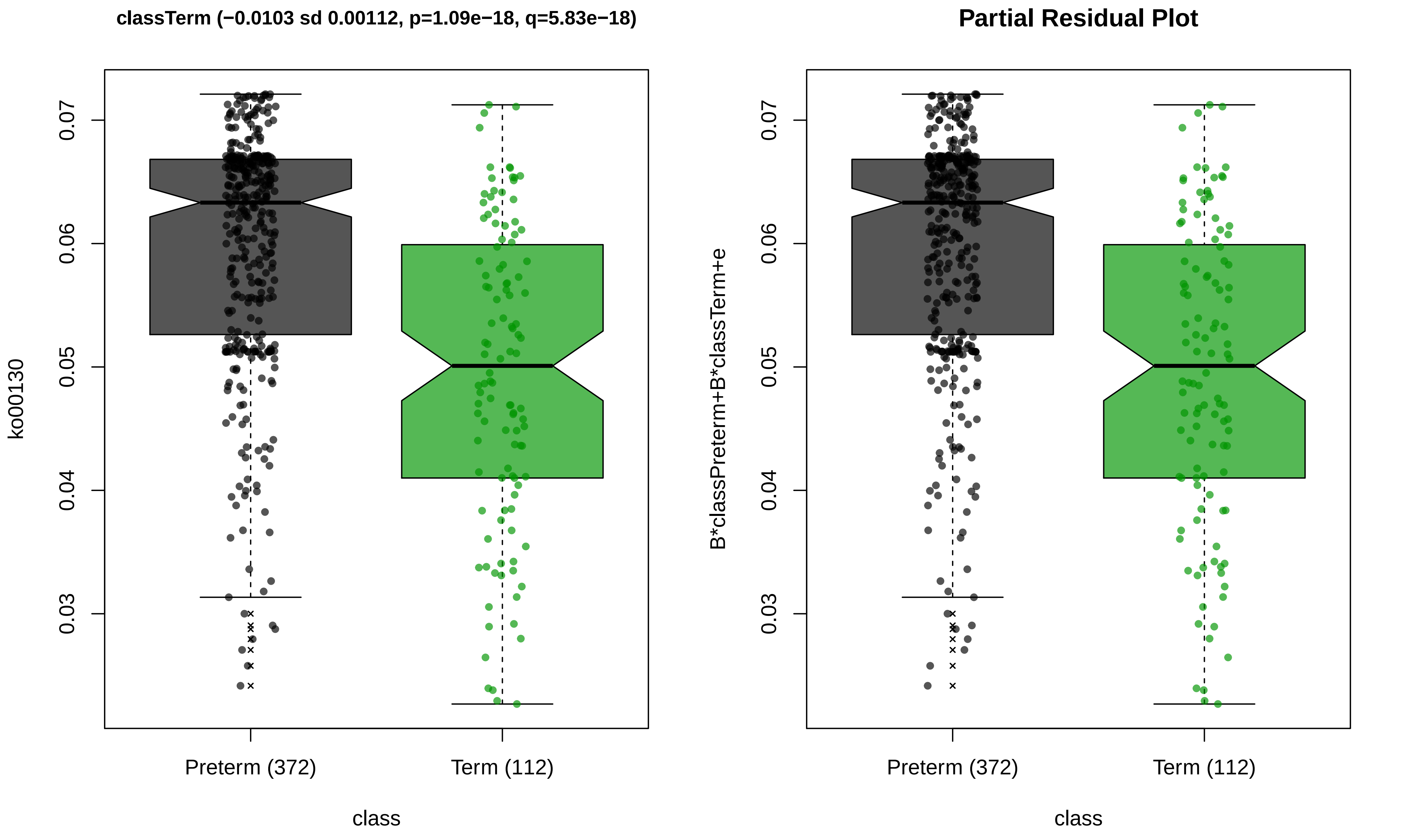
classTerm (−0.0103 sd 0.00112, p=1.09e−18, q=5.83e−18)
Partial Residual Plot
ko00130
B*classPreterm+B*classTerm+e
0.07
0.06
0.05
0.04
0.03
Preterm (372)
Term (112)
class

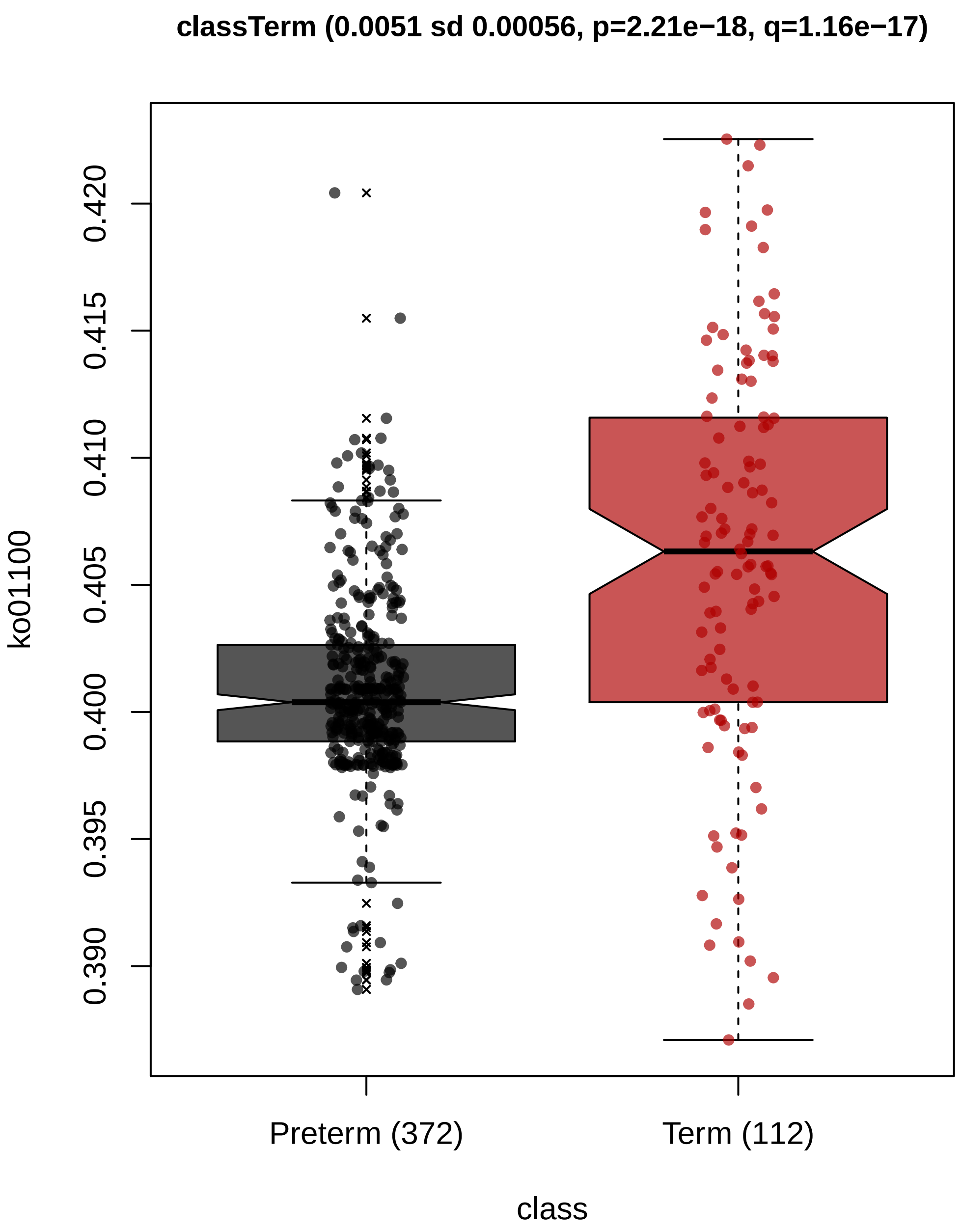

classTerm (0.0051 sd 0.00056, p=2.21e−18, q=1.16e−17)
ko01100
0.390
0.395
0.400
0.405
0.410
0.415
0.420
Preterm (372)
Term (112)
class

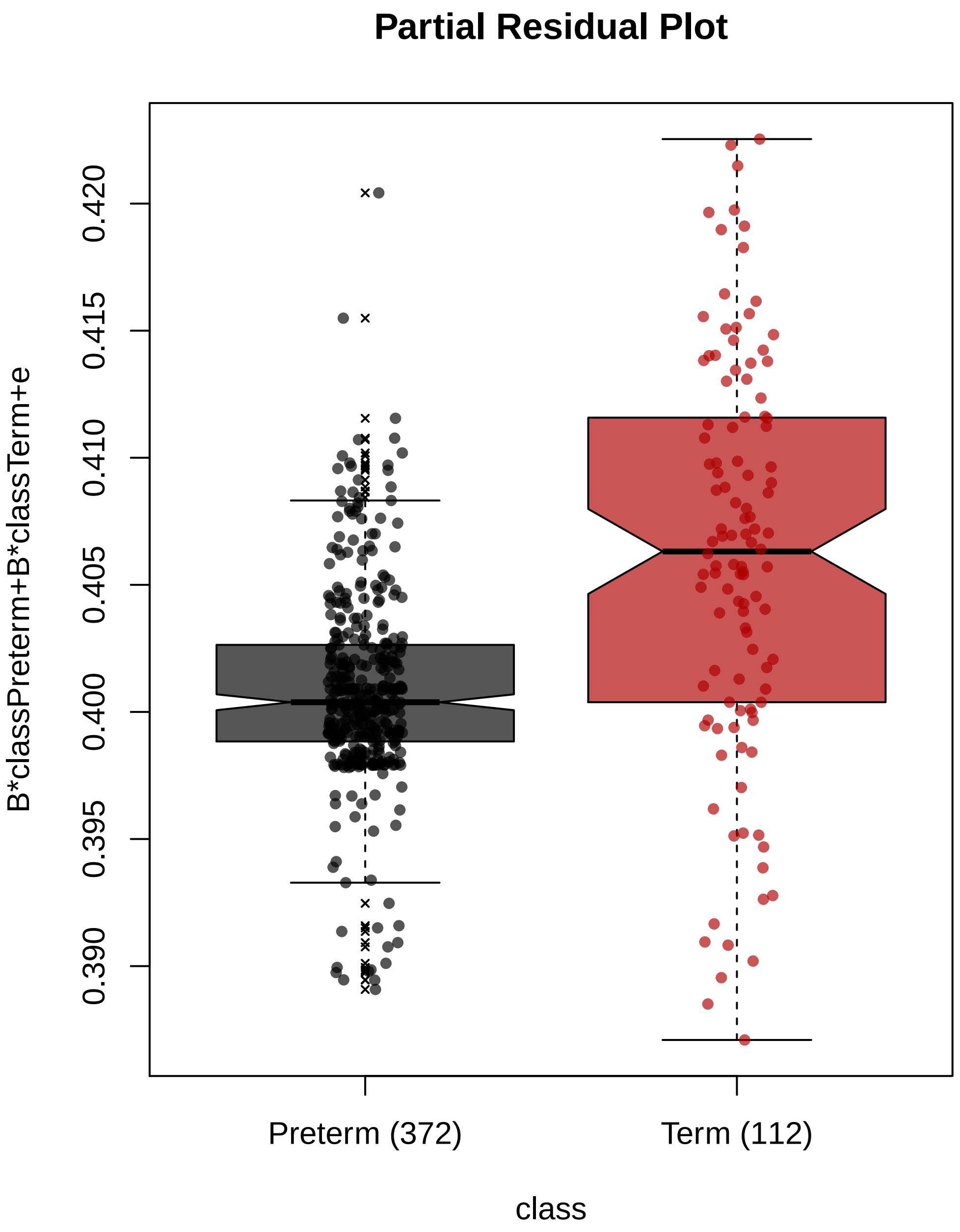

Partial Residual Plot
B*classPreterm+B*classTerm+e
0.390
0.395
0.400
0.405
0.410
0.415
0.420
Preterm (372)
Term (112)
class

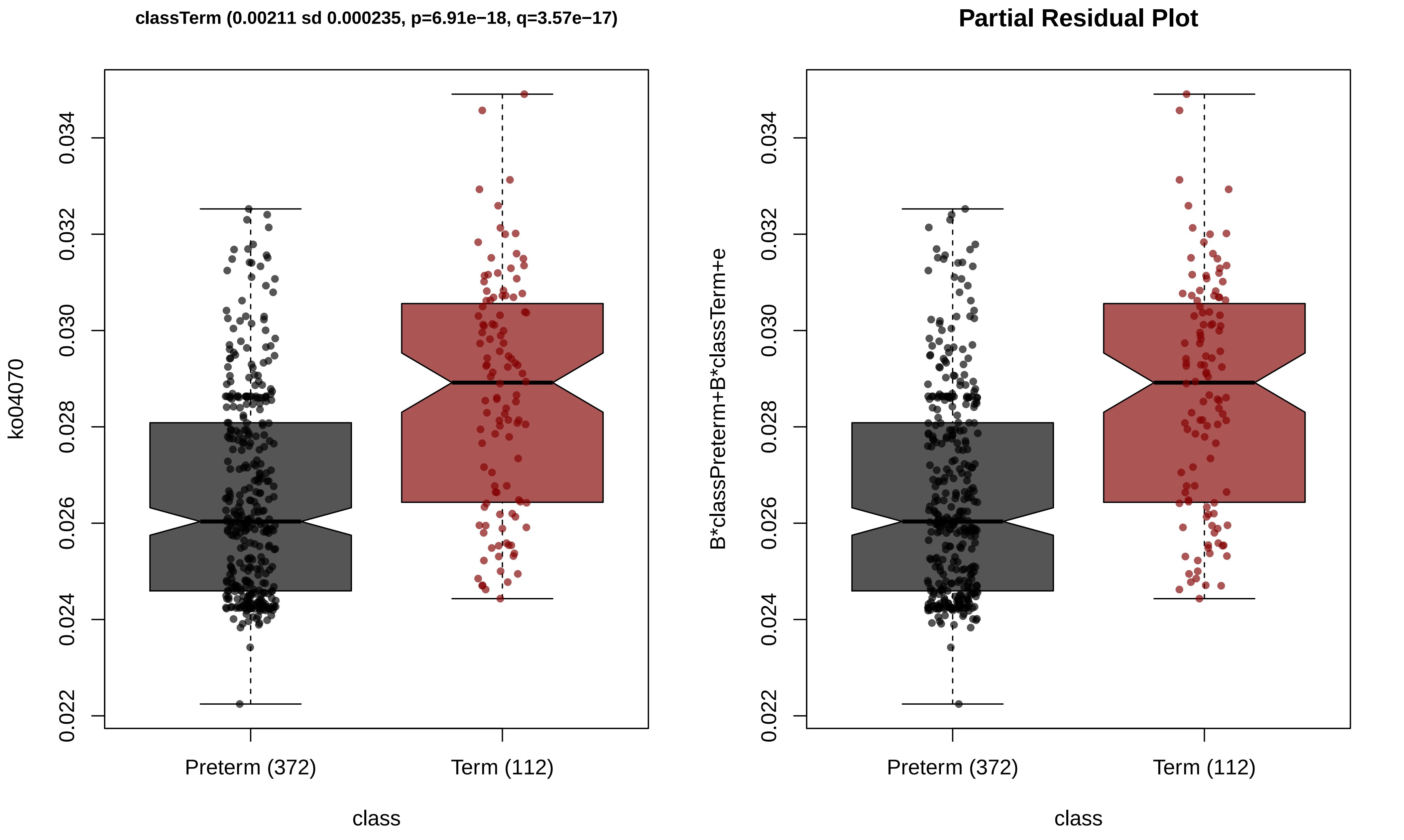
classTerm (0.00211 sd 0.000235, p=6.91e−18, q=3.57e−17)
Partial Residual Plot
ko04070
B*classPreterm+B*classTerm+e
0.022
0.024
0.026
0.028
0.030
0.032
0.034
Preterm (372)
Term (112)
class

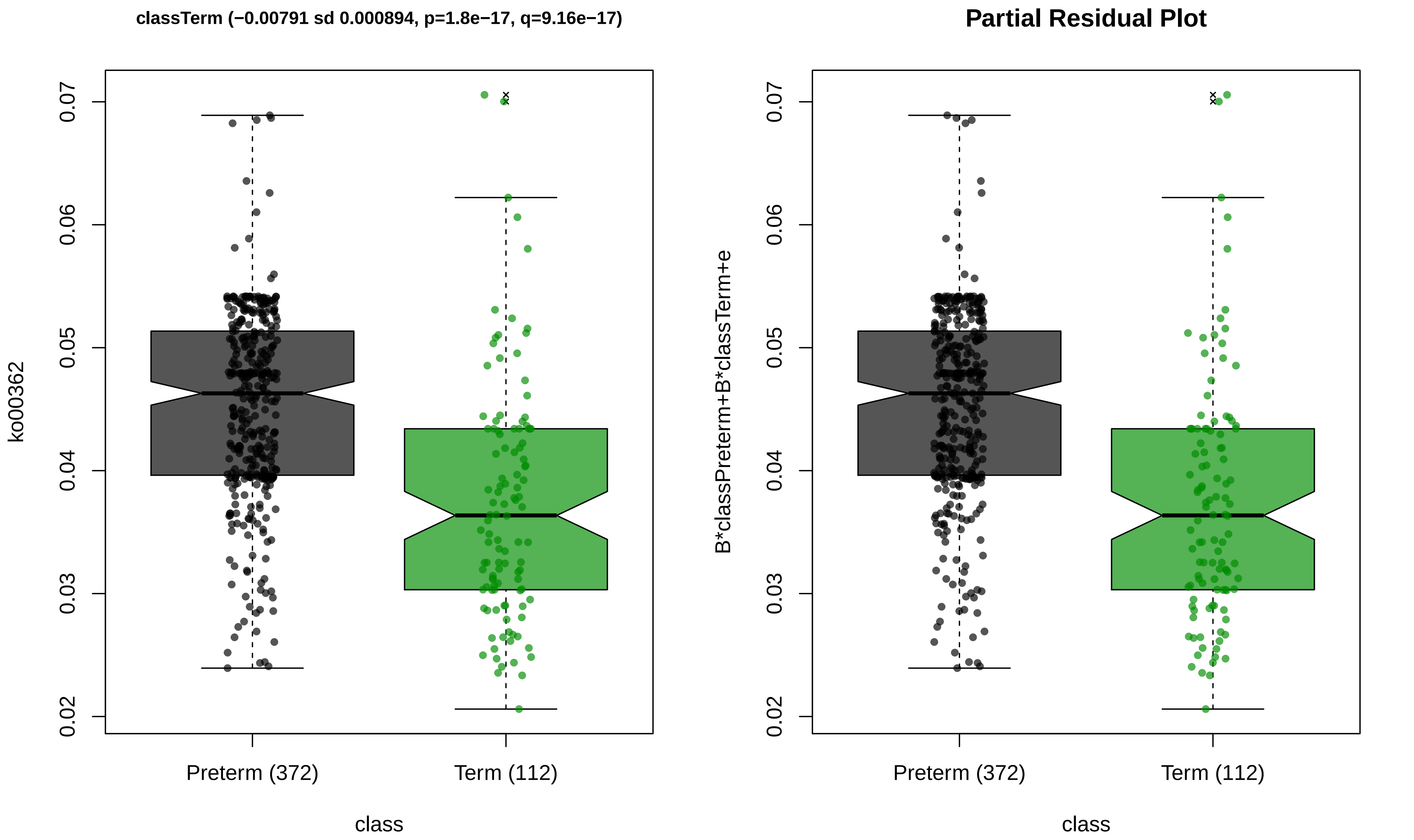
classTerm (−0.00791 sd 0.000894, p=1.8e−17, q=9.16e−17)
Partial Residual Plot
ko00362
B*classPreterm+B*classTerm+e
0.02
0.03
0.04
0.05
0.06
0.07
Preterm (372)
Term (112)
class

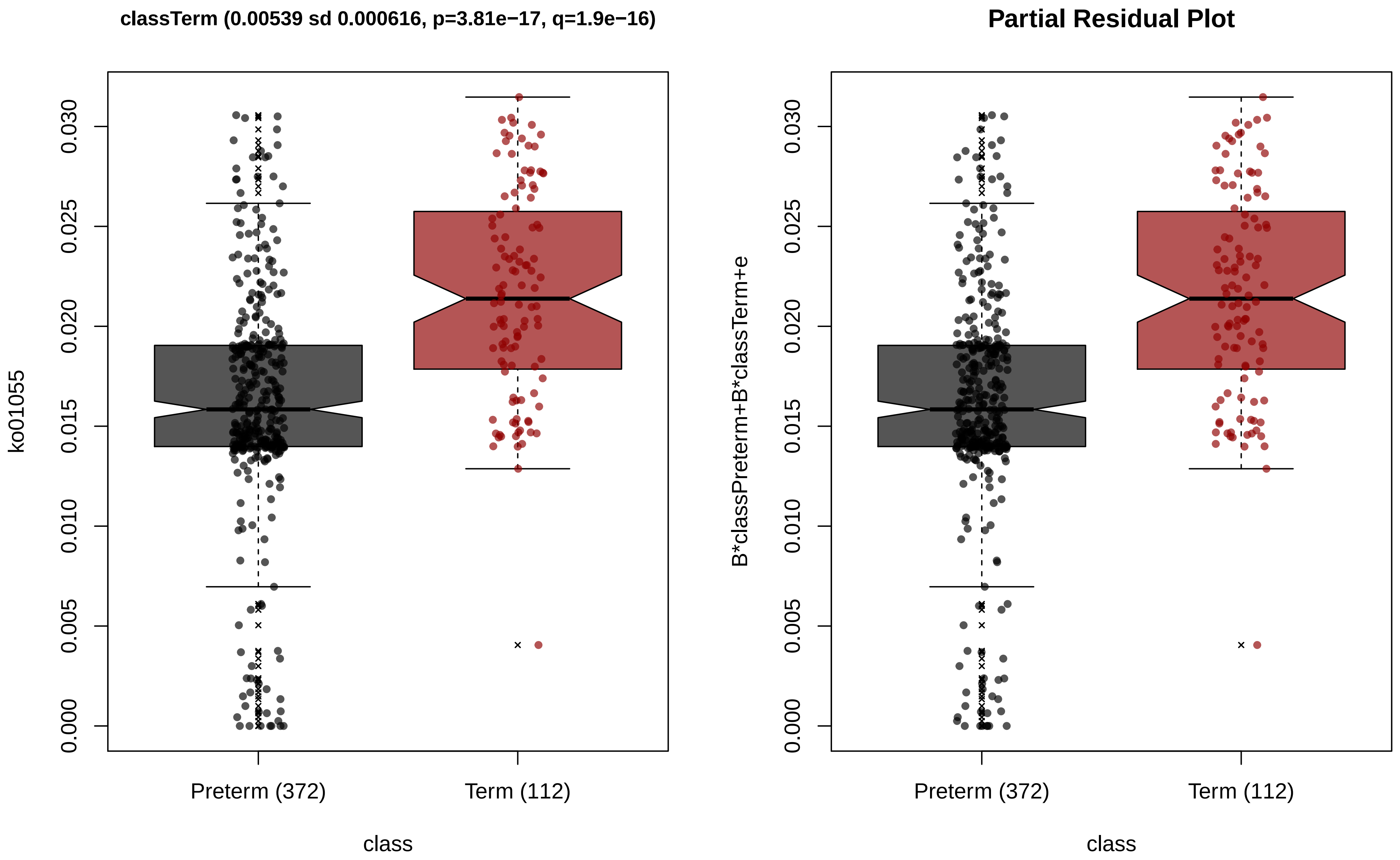

classTerm (0.00539 sd 0.000616, p=3.81e−17, q=1.9e−16)
ko01055
0.000
0.005
0.010
0.015
0.020
0.025
0.030
Preterm (372)
Term (112)
class
Partial Residual Plot
B*classPreterm+B*classTerm+e
0.000
0.005
0.010
0.015
0.020
0.025
0.030
Preterm (372)
Term (112)
class

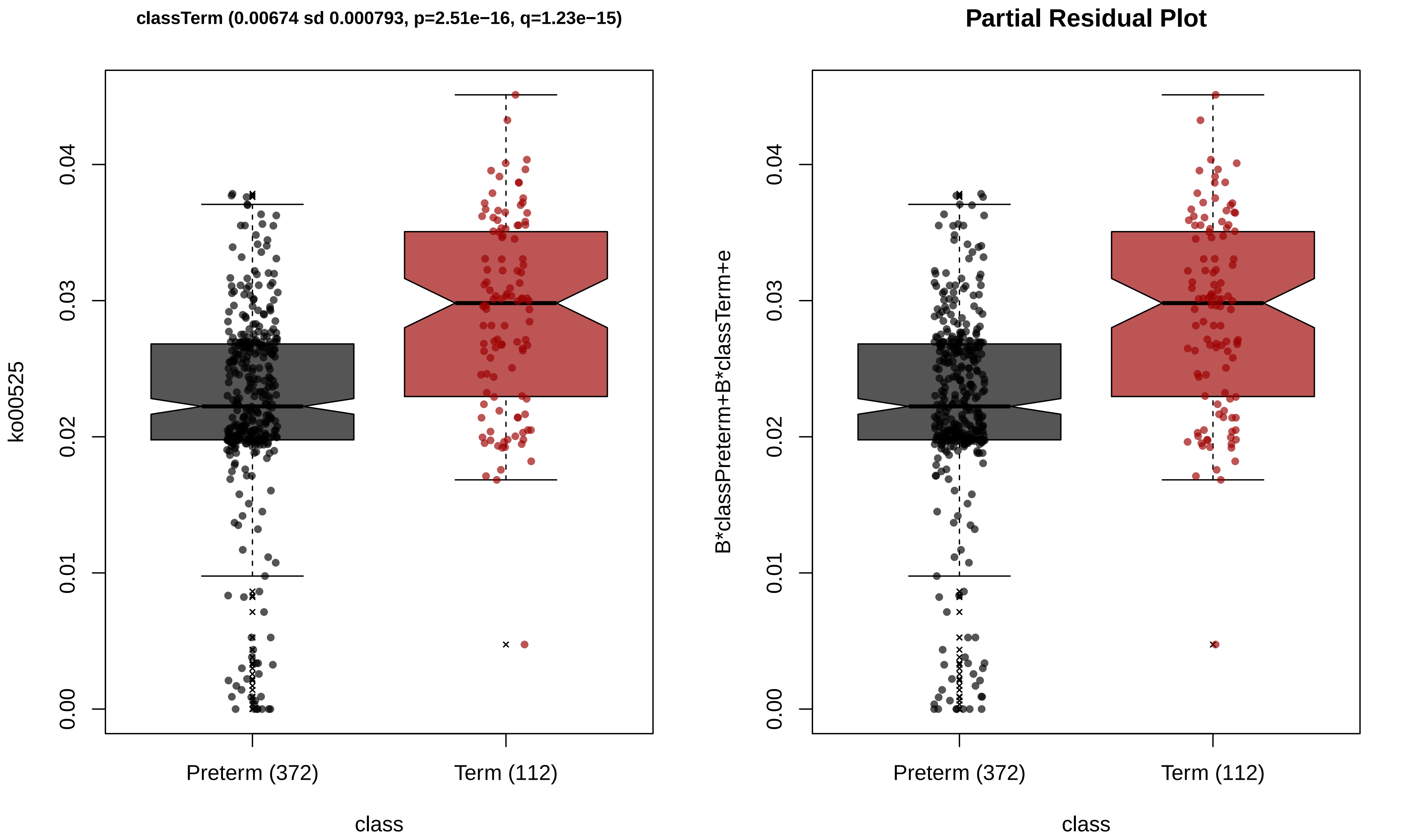

classTerm (0.00674 sd 0.000793, p=2.51e−16, q=1.23e−15)
Partial Residual Plot
ko00525
B*classPreterm+B*classTerm+e
0.00
0.01
0.02
0.03
0.04
Preterm (372)
Term (112)
class

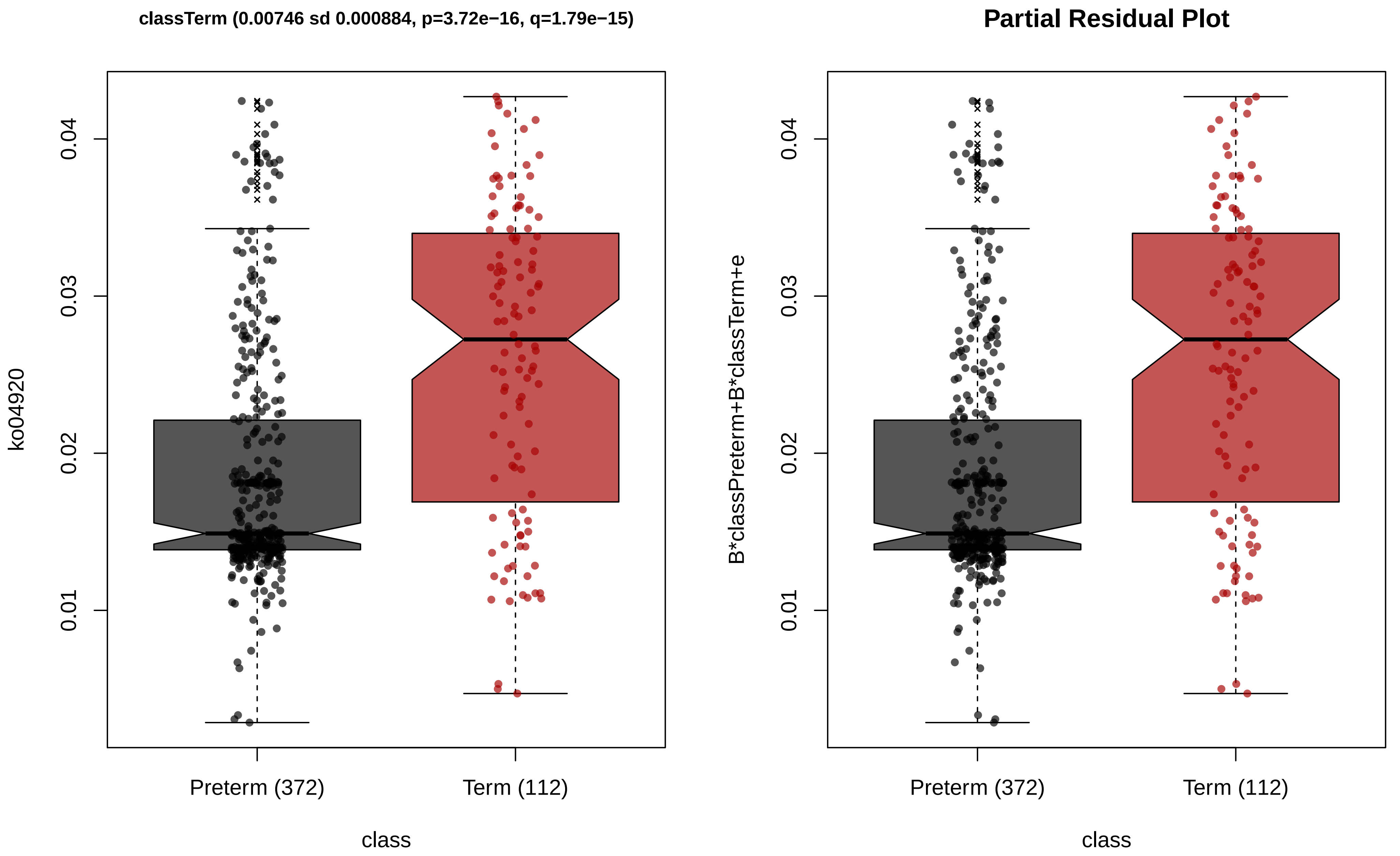

classTerm (0.00746 sd 0.000884, p=3.72e−16, q=1.79e−15)
ko04920
0.01
0.02
0.03
0.04
Preterm (372)
Term (112)
class
Partial Residual Plot
B*classPreterm+B*classTerm+e
0.01
0.02
0.03
0.04
Preterm (372)
Term (112)
class

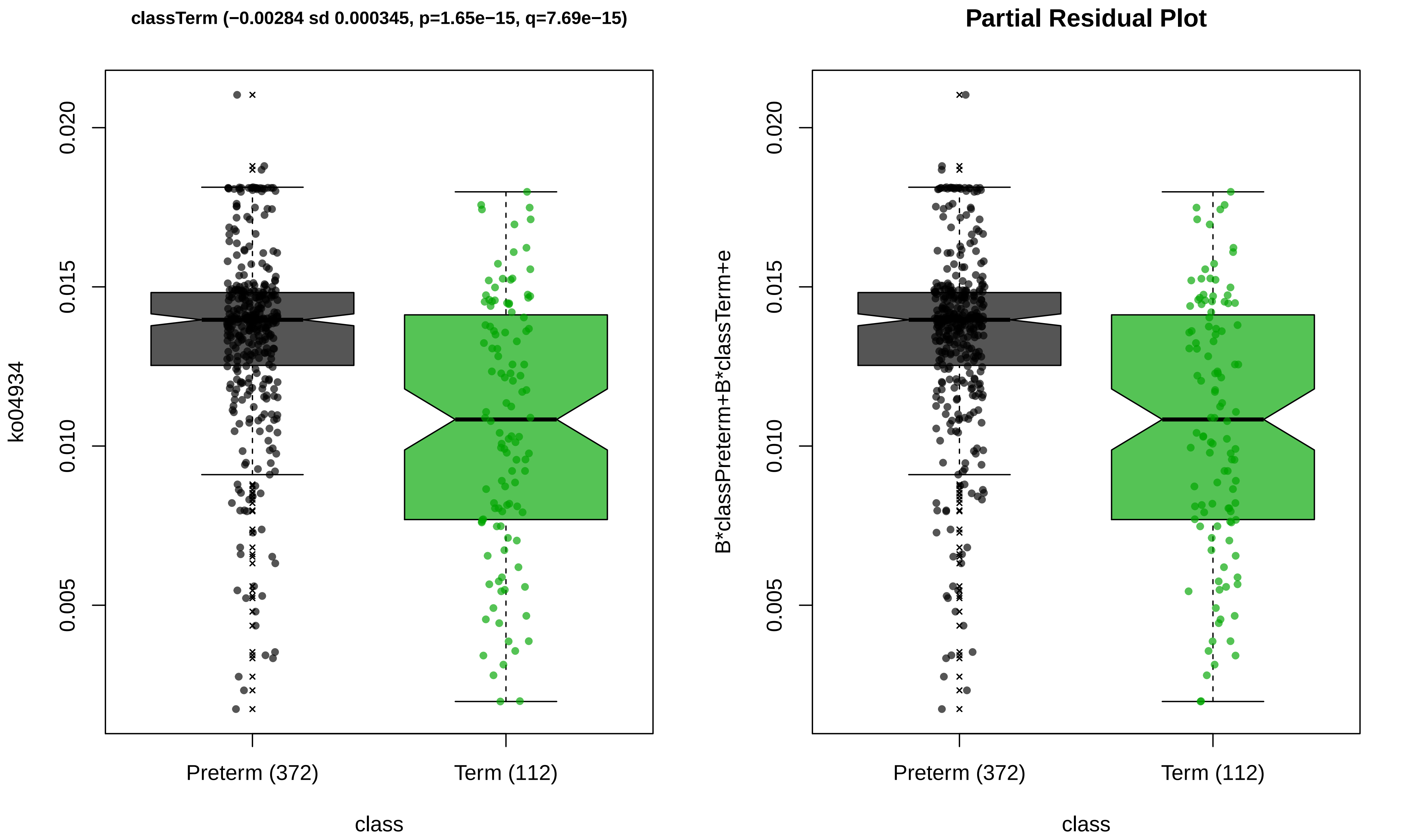
classTerm (−0.00284 sd 0.000345, p=1.65e−15, q=7.69e−15)
ko04934
0.020
0.015
0.010
0.005
Preterm (372)
Term (112)
class
Partial Residual Plot
B*classPreterm+B*classTerm+e
0.020
0.015
0.010
0.005
Preterm (372)
Term (112)
class

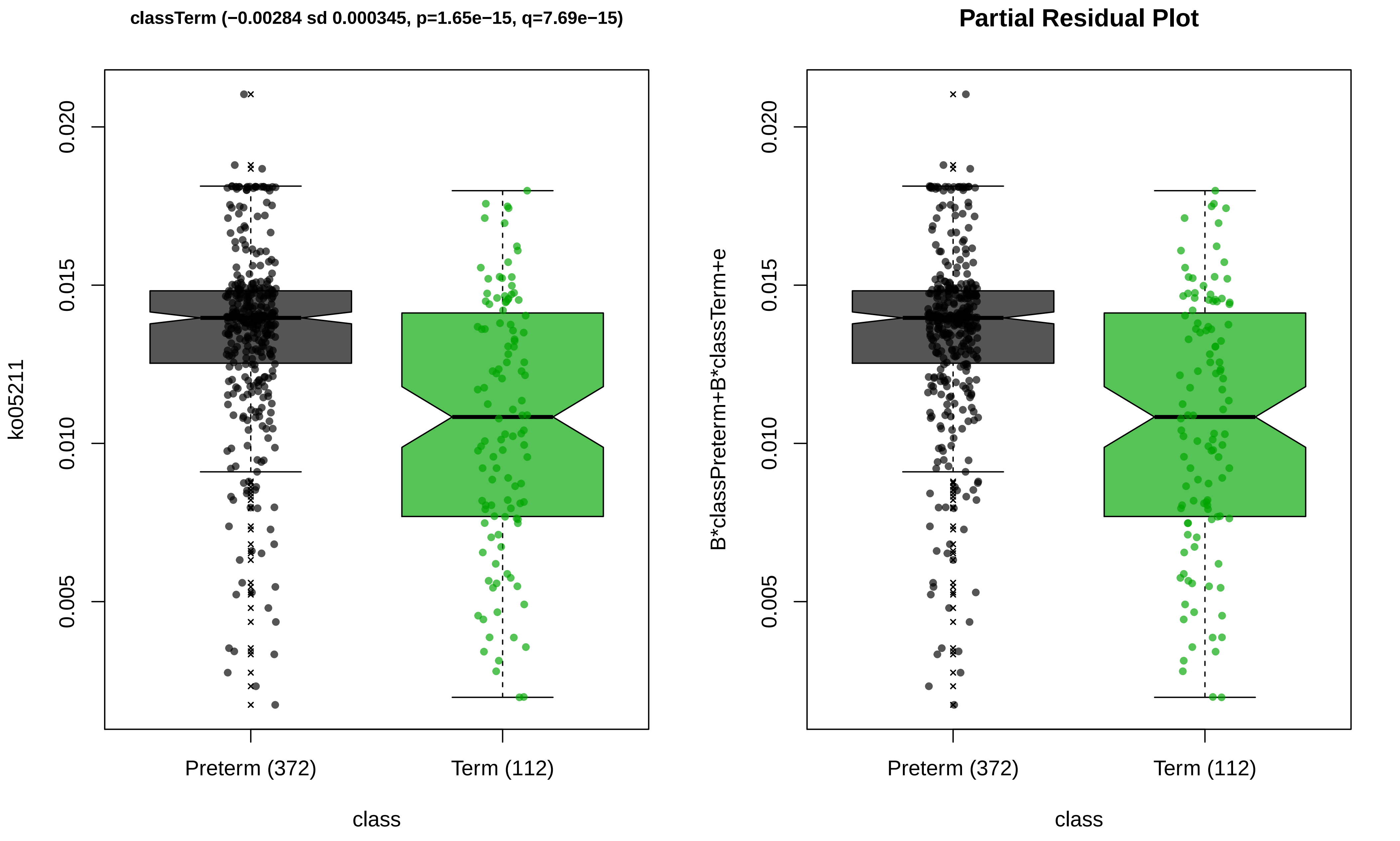

classTerm (−0.00284 sd 0.000345, p=1.65e−15, q=7.69e−15)
ko05211
0.005
0.010
0.015
0.020
Preterm (372)
Term (112)
class
Partial Residual Plot
B*classPreterm+B*classTerm+e
0.005
0.010
0.015
0.020
Preterm (372)
Term (112)
class

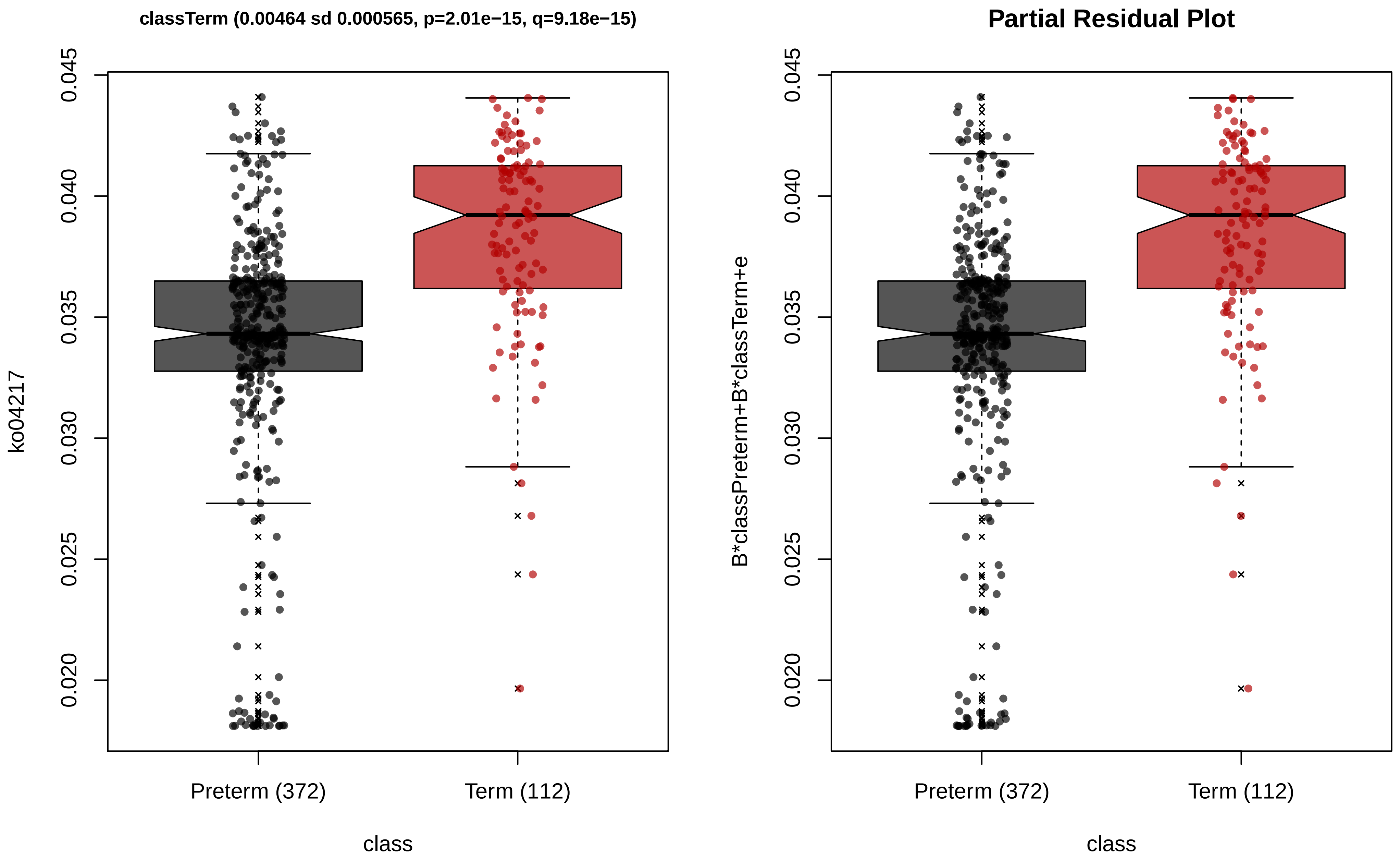
classTerm (0.00464 sd 0.000565, p=2.01e−15, q=9.18e−15)
Partial Residual Plot
ko04217
B*classPreterm+B*classTerm+e
0.045
0.040
0.035
0.030
0.025
0.020
Preterm (372)
Term (112)
class

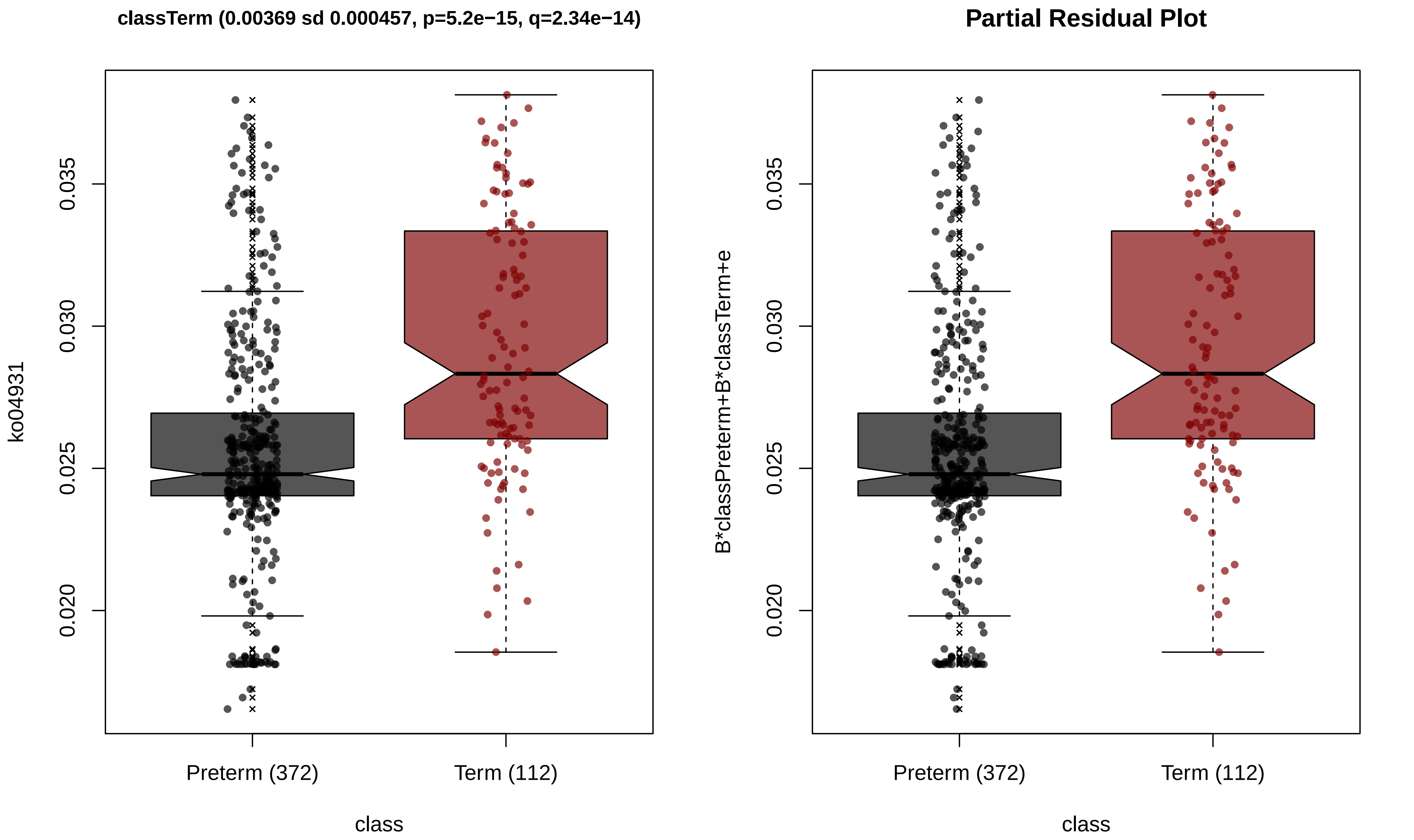

classTerm (0.00369 sd 0.000457, p=5.2e−15, q=2.34e−14)
ko04931
0.020
0.025
0.030
0.035
Preterm (372)
Term (112)
class
Partial Residual Plot
B*classPreterm+B*classTerm+e
0.020
0.025
0.030
0.035
Preterm (372)
Term (112)
class

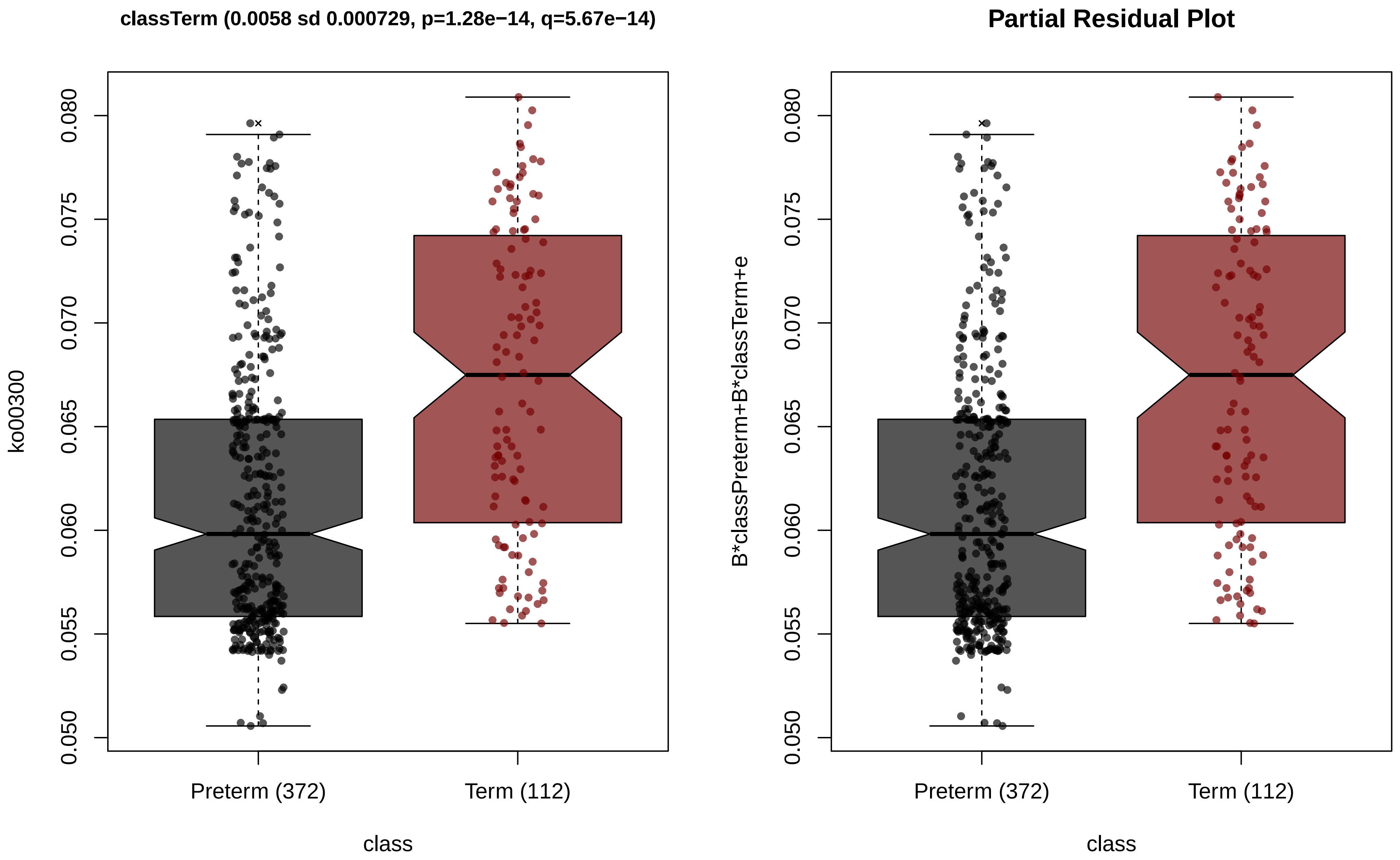

classTerm (0.0058 sd 0.000729, p=1.28e−14, q=5.67e−14)
Partial Residual Plot
ko00300
B*classPreterm+B*classTerm+e
0.050
0.055
0.060
0.065
0.070
0.075
0.080
Preterm (372)
Term (112)
class

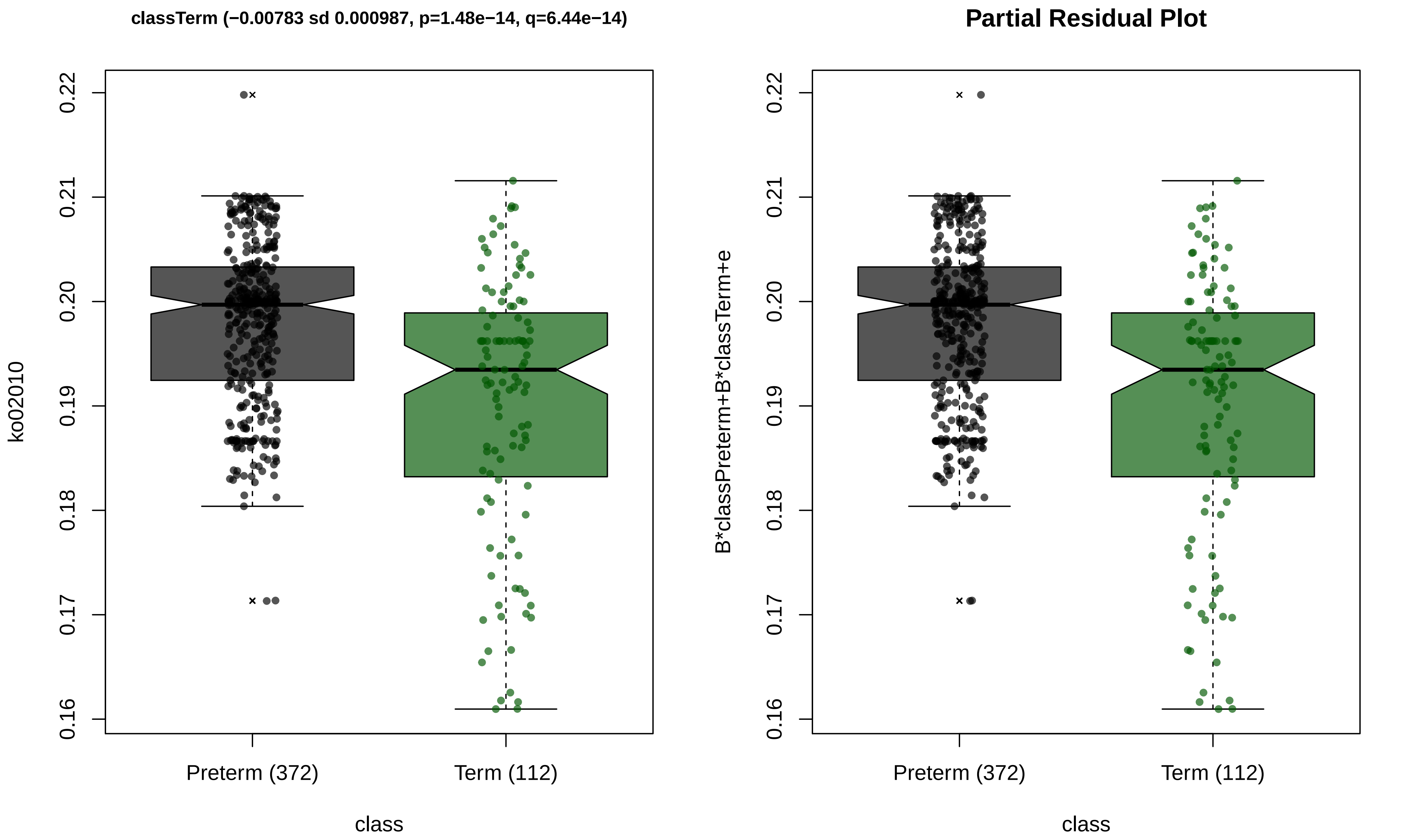
classTerm (−0.00783 sd 0.000987, p=1.48e−14, q=6.44e−14)
Partial Residual Plot
ko02010
B*classPreterm+B*classTerm+e
0.16
0.17
0.18
0.19
0.20
0.21
0.22
Preterm (372)
Term (112)
class

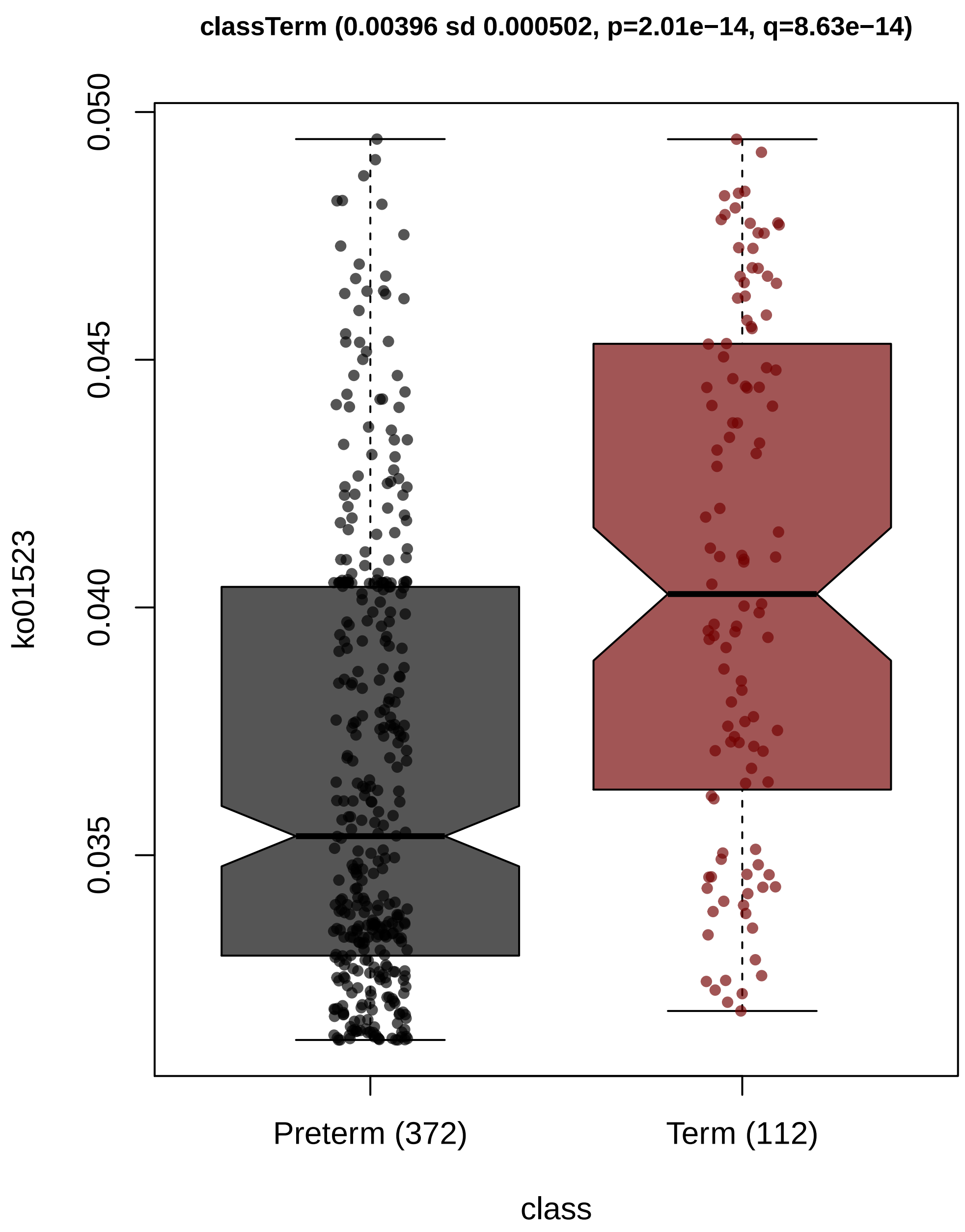

classTerm (0.00396 sd 0.000502, p=2.01e−14, q=8.63e−14)
ko01523
0.050
0.045
0.040
0.035
Preterm (372)
Term (112)
class

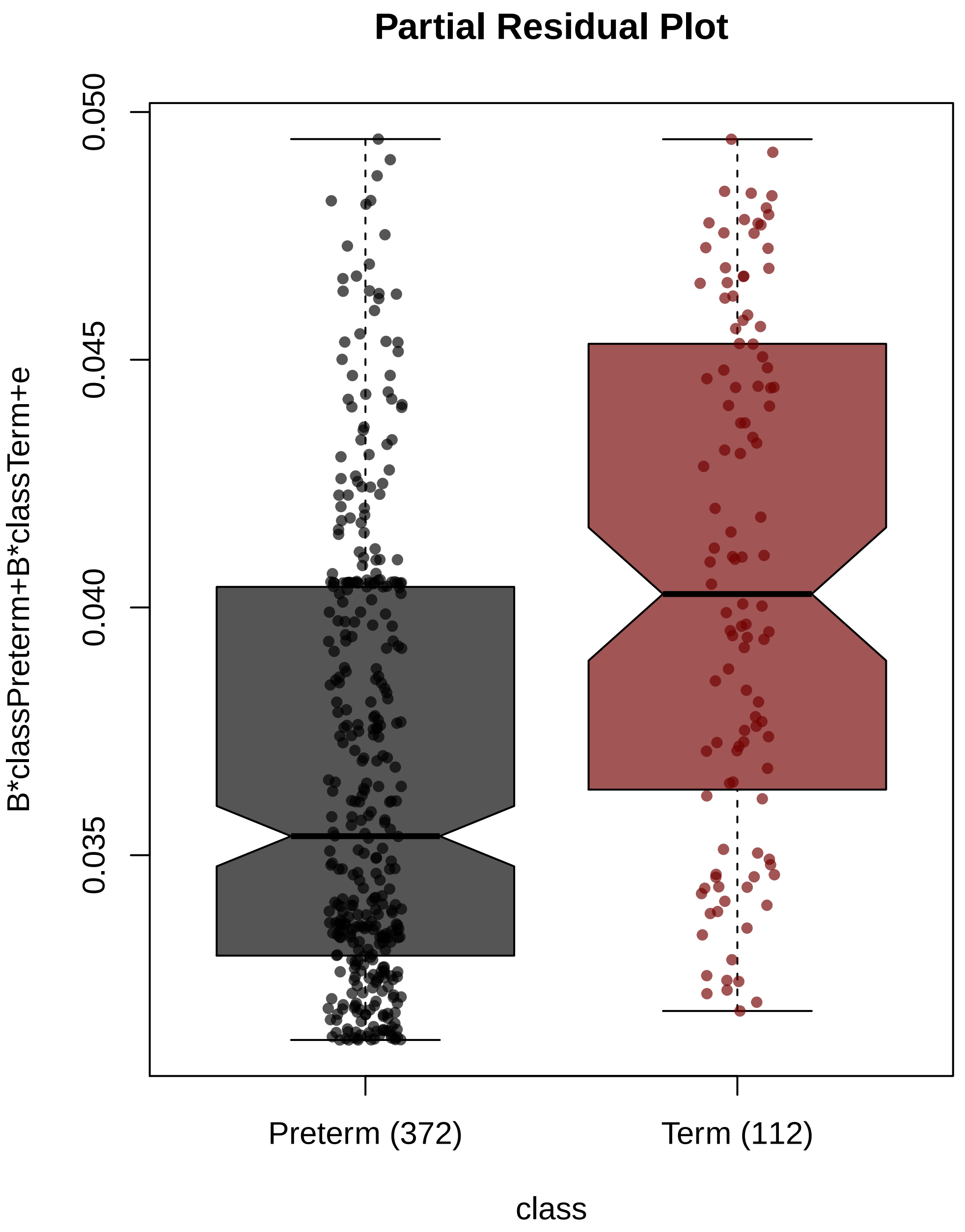

Partial Residual Plot
B*classPreterm+B*classTerm+e
0.050
0.045
0.040
0.035
Preterm (372)
Term (112)
class

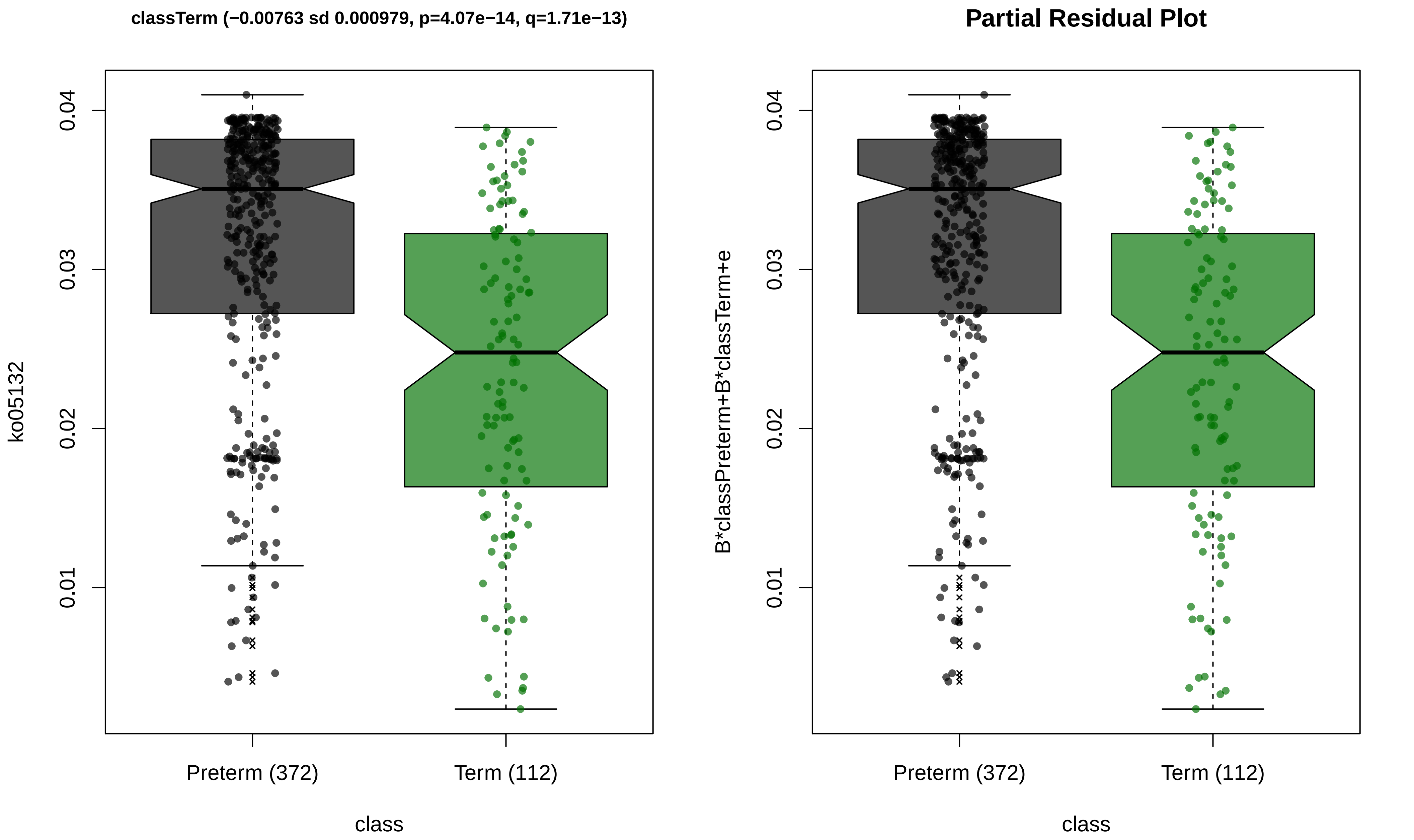
classTerm (−0.00763 sd 0.000979, p=4.07e−14, q=1.71e−13)
ko05132
0.04
0.03
0.02
0.01
Preterm (372)
Term (112)
class
Partial Residual Plot
B*classPreterm+B*classTerm+e
0.04
0.03
0.02
0.01
Preterm (372)
Term (112)
class

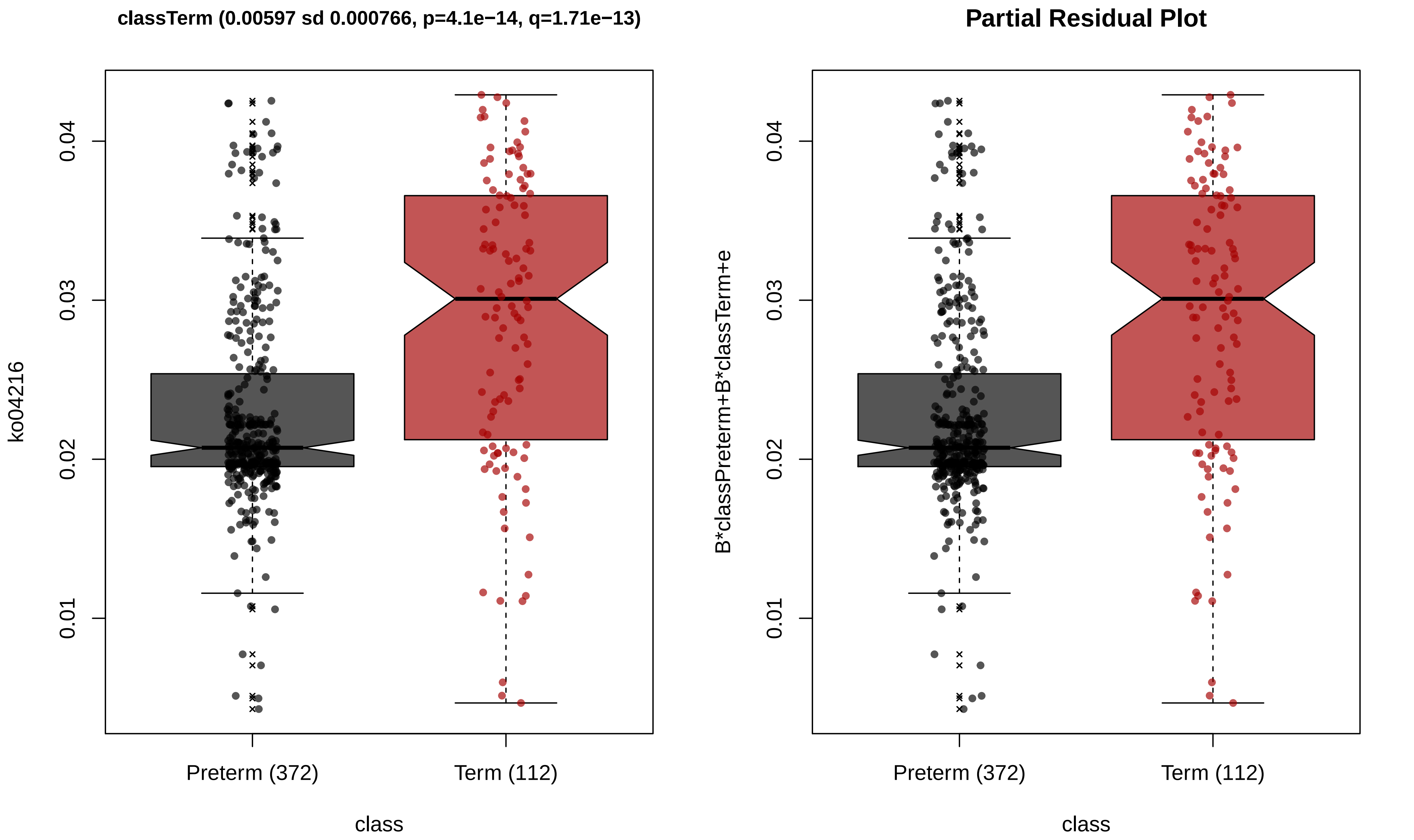

classTerm (0.00597 sd 0.000766, p=4.1e−14, q=1.71e−13)
Partial Residual Plot
ko04216
B*classPreterm+B*classTerm+e
0.01
0.02
0.03
0.04
Preterm (372)
Term (112)
class

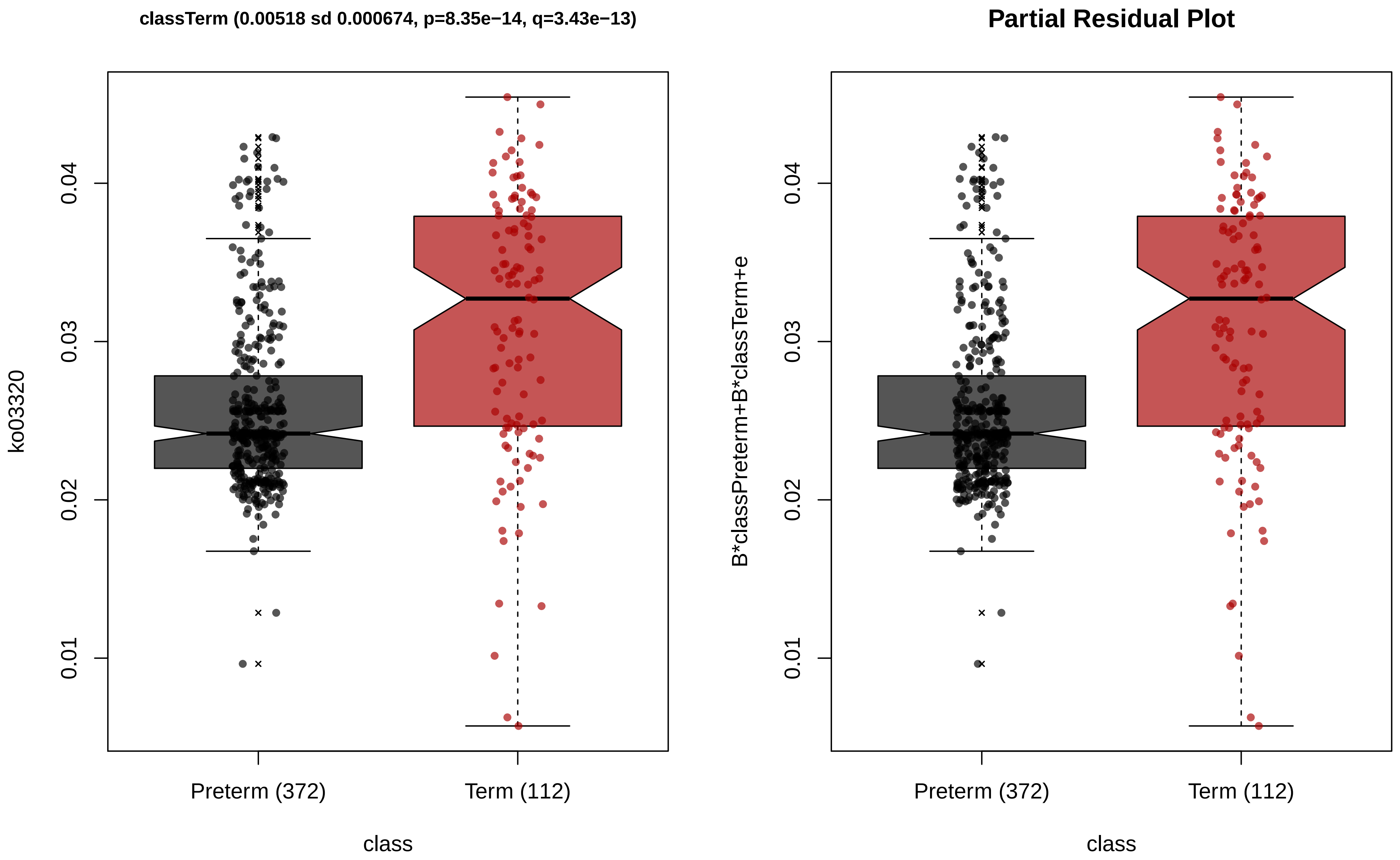
classTerm (0.00518 sd 0.000674, p=8.35e−14, q=3.43e−13)
ko03320
0.01
0.02
0.03
0.04
Preterm (372)
Term (112)
class
Partial Residual Plot
B*classPreterm+B*classTerm+e
0.01
0.02
0.03
0.04
Preterm (372)
Term (112)
class

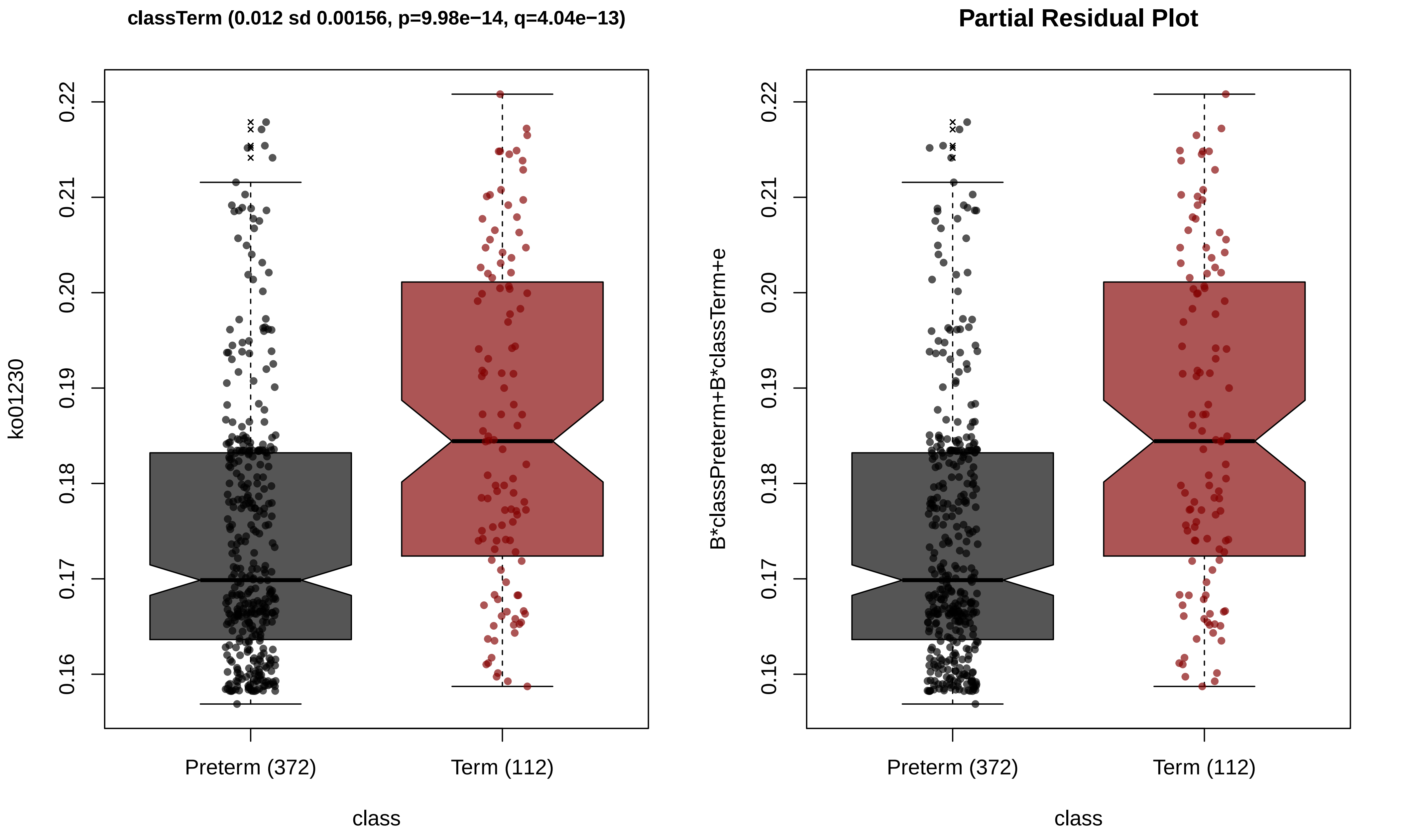
classTerm (0.012 sd 0.00156, p=9.98e−14, q=4.04e−13)
ko01230
0.16
0.17
0.18
0.19
0.20
0.21
0.22
Preterm (372)
Term (112)
class
Partial Residual Plot
B*classPreterm+B*classTerm+e
class

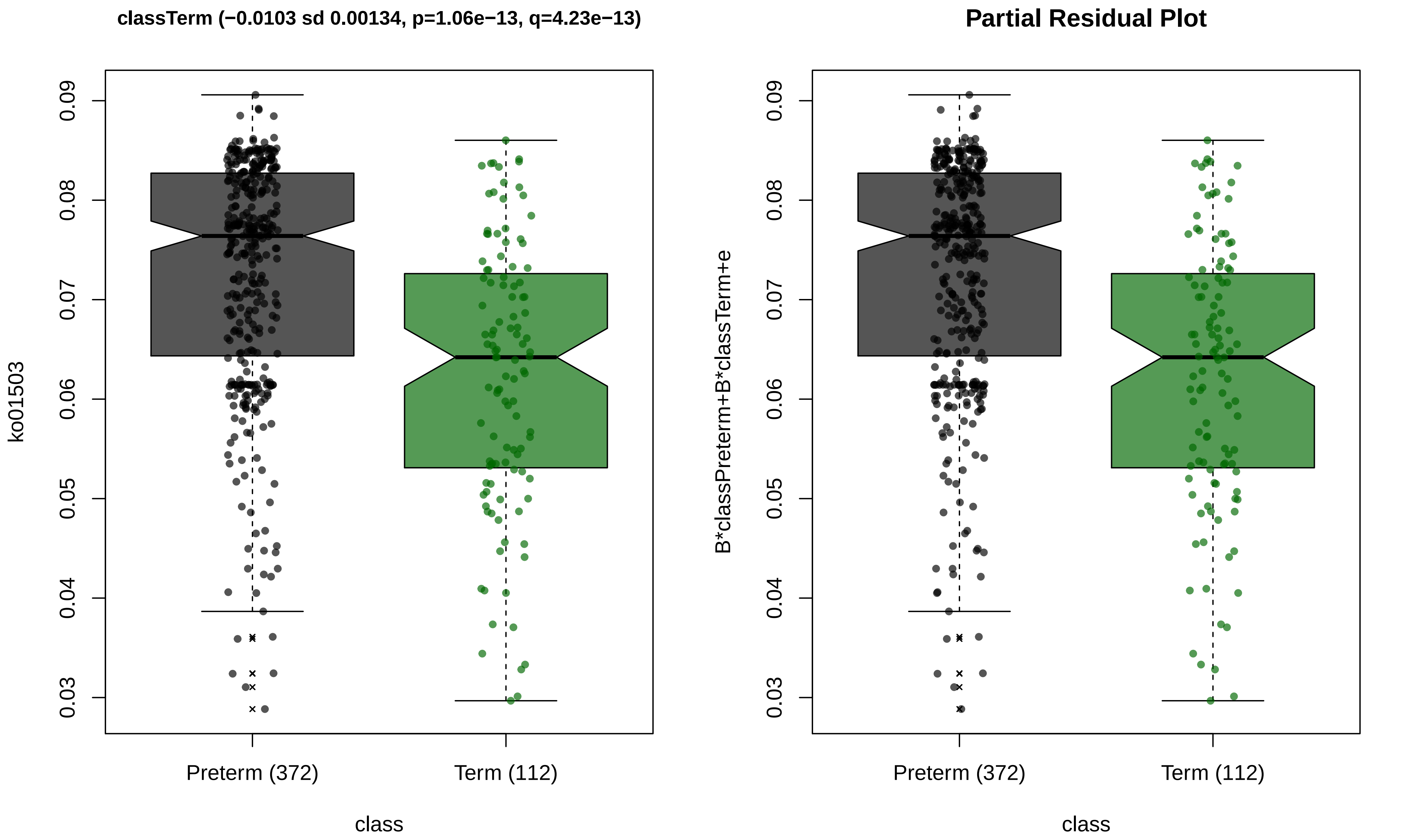
classTerm (−0.0103 sd 0.00134, p=1.06e−13, q=4.23e−13)
Partial Residual Plot
ko01503
B*classPreterm+B*classTerm+e
0.09
0.08
0.07
0.06
0.05
0.04
0.03
Preterm (372)
Term (112)
class

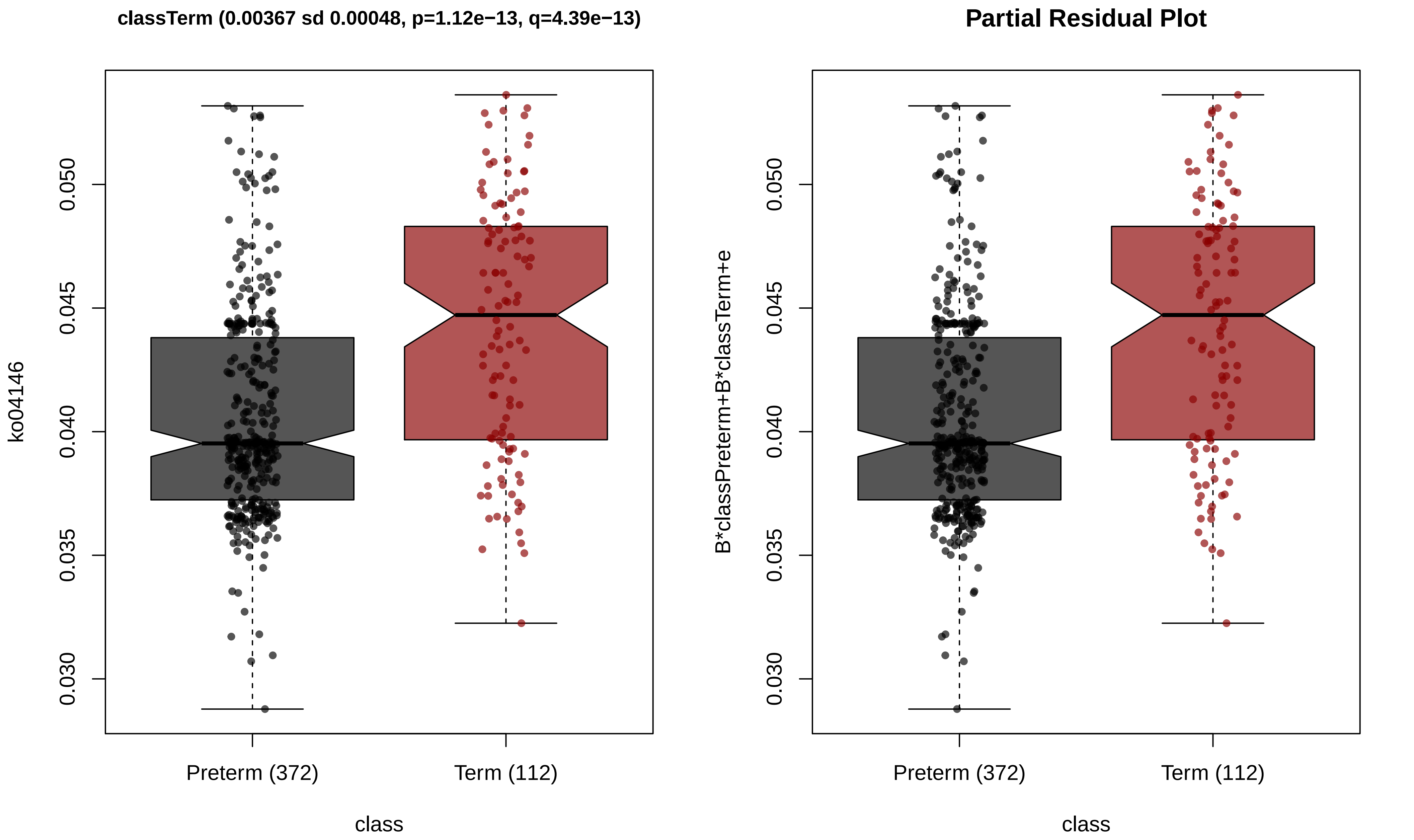

classTerm (0.00367 sd 0.00048, p=1.12e−13, q=4.39e−13)
ko04146
0.030
0.035
0.040
0.045
0.050
Preterm (372)
Term (112)
class
Partial Residual Plot
B*classPreterm+B*classTerm+e
0.030
0.035
0.040
0.045
0.050
Preterm (372)
Term (112)
class

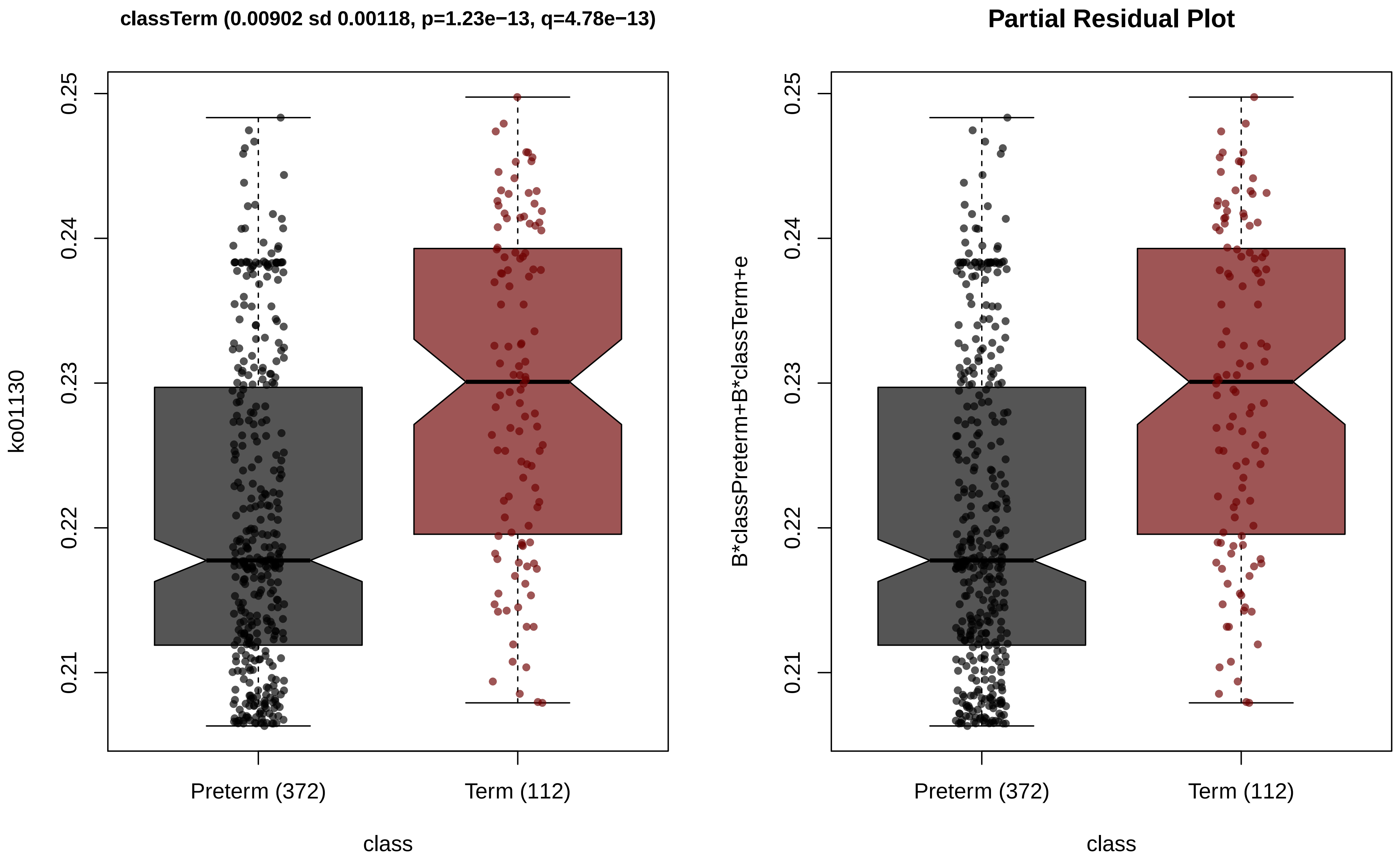

classTerm (0.00902 sd 0.00118, p=1.23e−13, q=4.78e−13)
ko01130
0.21
0.22
0.23
0.24
0.25
Preterm (372)
Term (112)
class
Partial Residual Plot
B*classPreterm+B*classTerm+e
0.21
0.22
0.23
0.24
0.25
Preterm (372)
Term (112)
class

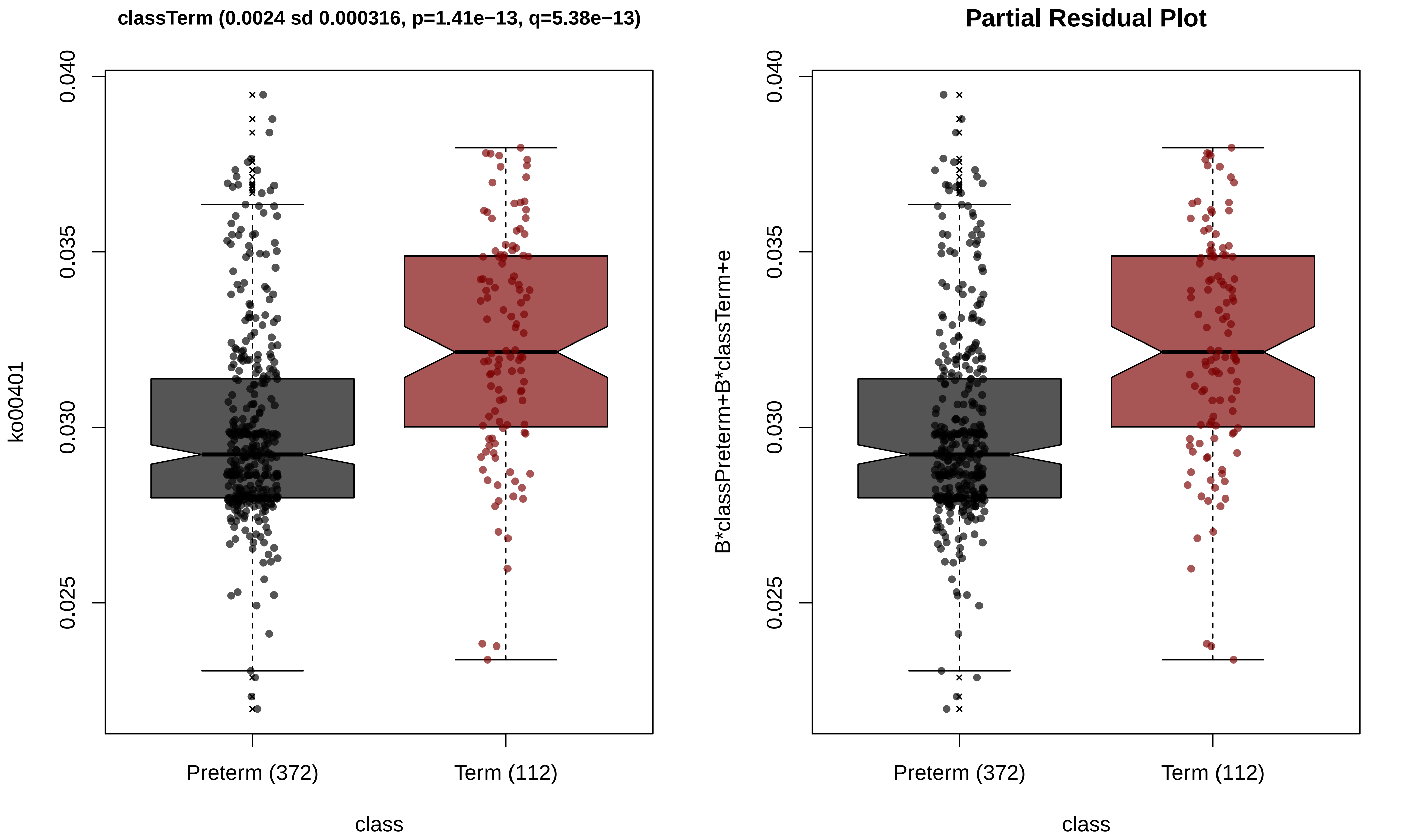
classTerm (0.0024 sd 0.000316, p=1.41e−13, q=5.38e−13)
ko00401
0.040
0.035
0.030
0.025
Preterm (372)
Term (112)
class
Partial Residual Plot
B*classPreterm+B*classTerm+e
0.040
0.035
0.030
0.025
Preterm (372)
Term (112)
class

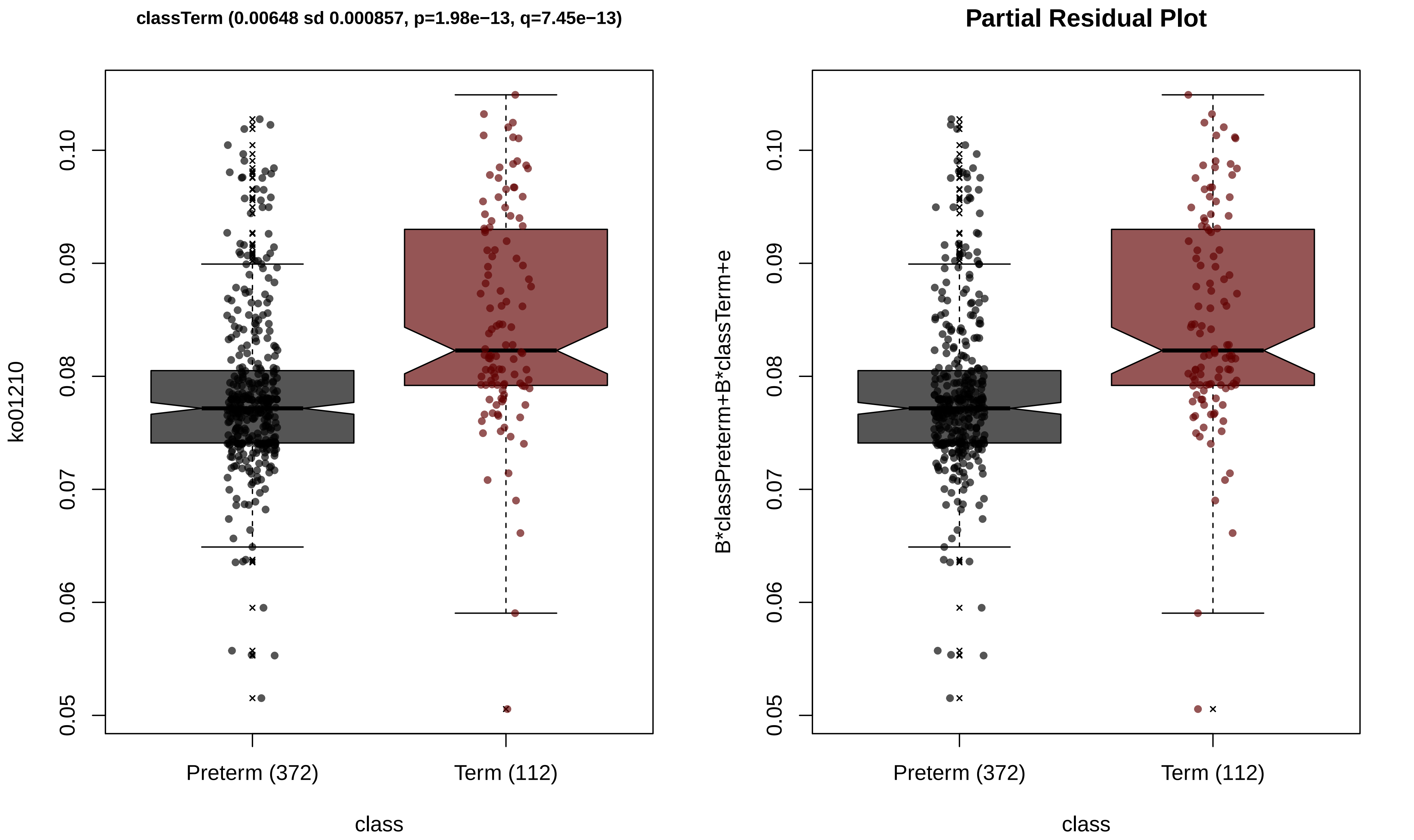

classTerm (0.00648 sd 0.000857, p=1.98e−13, q=7.45e−13)
Partial Residual Plot
ko01210
B*classPreterm+B*classTerm+e
0.05
0.06
0.07
0.08
0.09
0.10
Preterm (372)
Term (112)
class

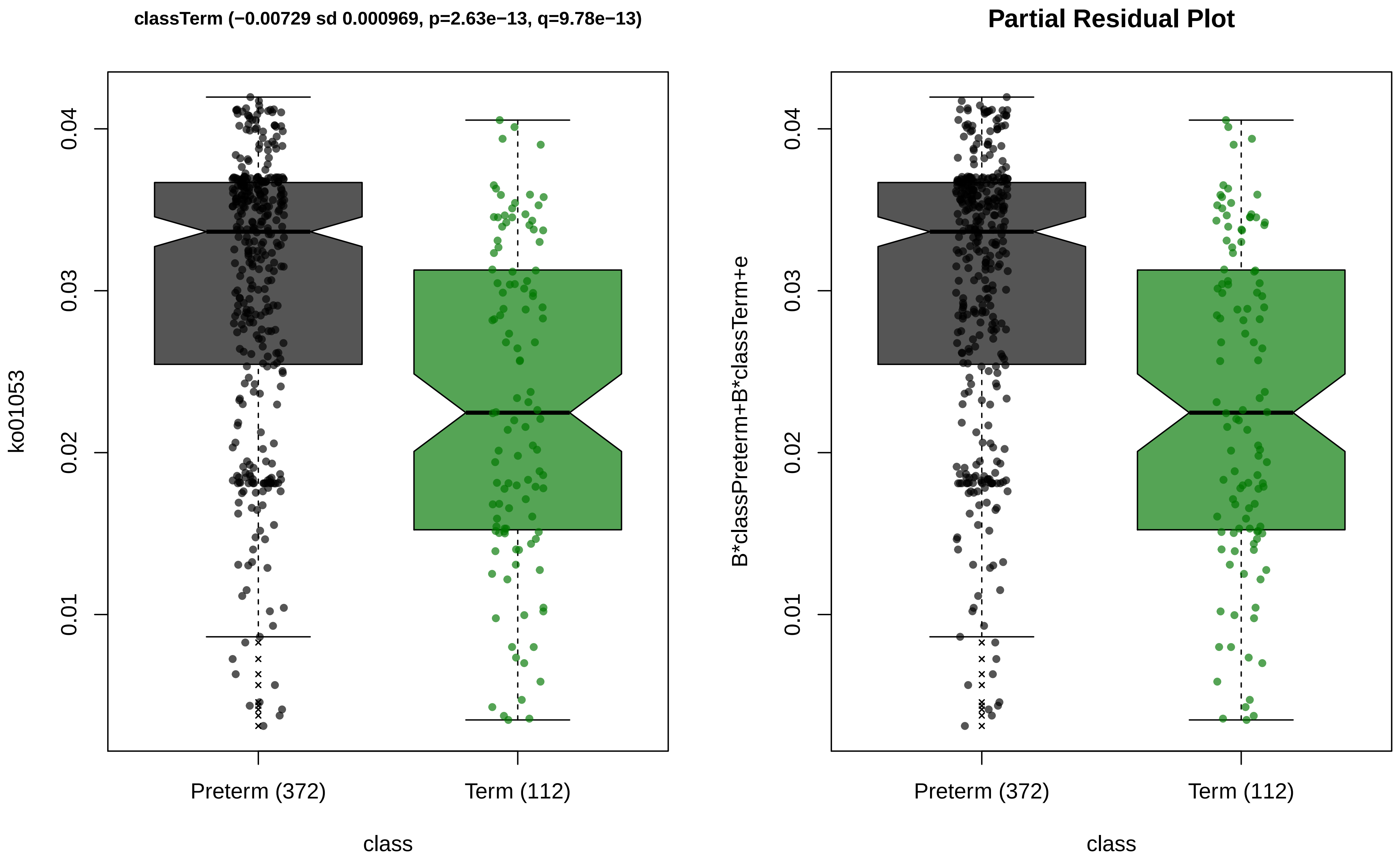
classTerm (−0.00729 sd 0.000969, p=2.63e−13, q=9.78e−13)
Partial Residual Plot
ko01053
B*classPreterm+B*classTerm+e
0.01
0.02
0.03
0.04
Preterm (372)
Term (112)
class

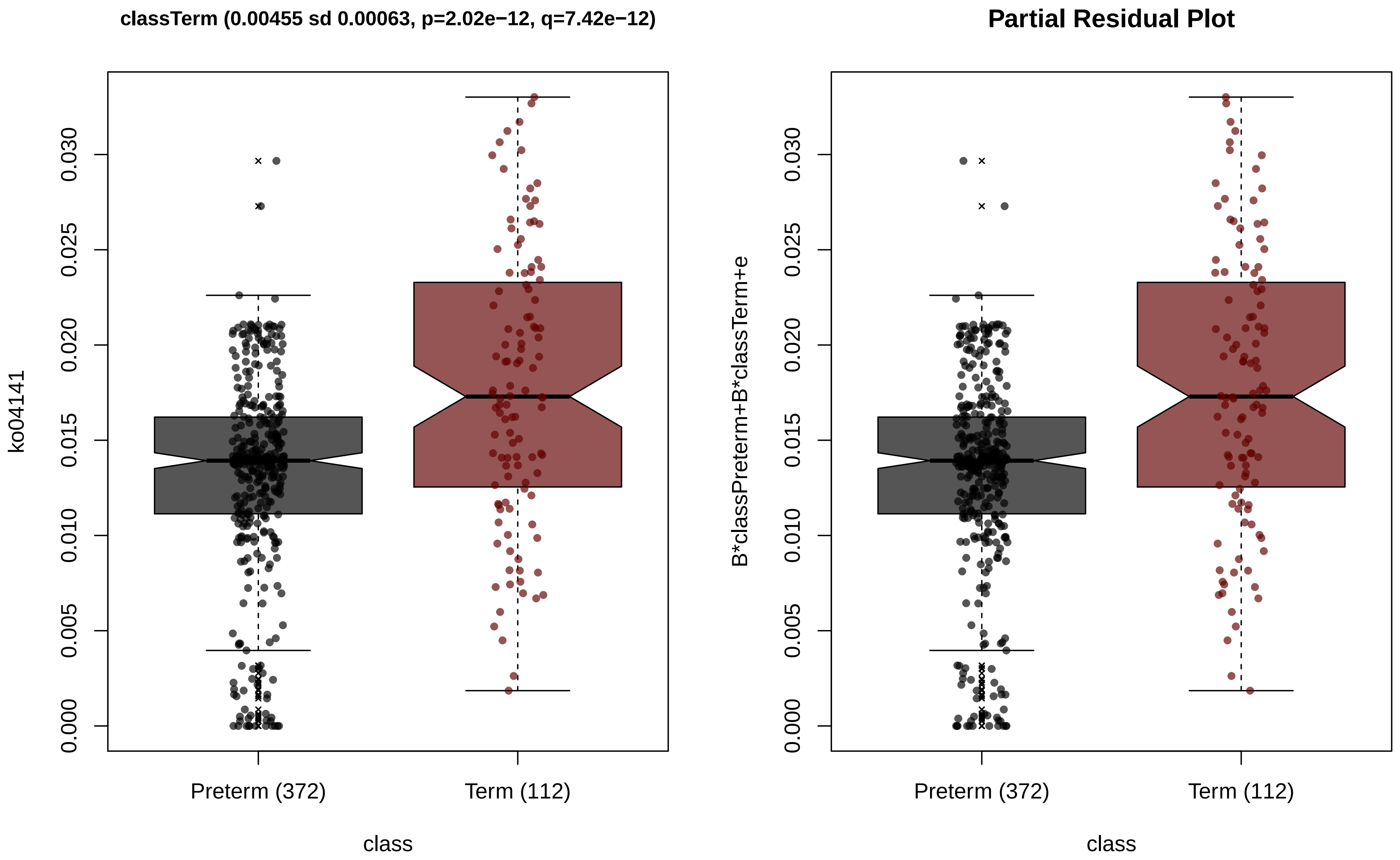
classTerm (0.00455 sd 0.00063, p=2.02e−12, q=7.42e−12)
ko04141
0.000
0.005
0.010
0.015
0.020
0.025
0.030
Preterm (372)
Term (112)
class
Partial Residual Plot
B*classPreterm+B*classTerm+e
0.000
0.005
0.010
0.015
0.020
0.025
0.030
Preterm (372)
Term (112)
class

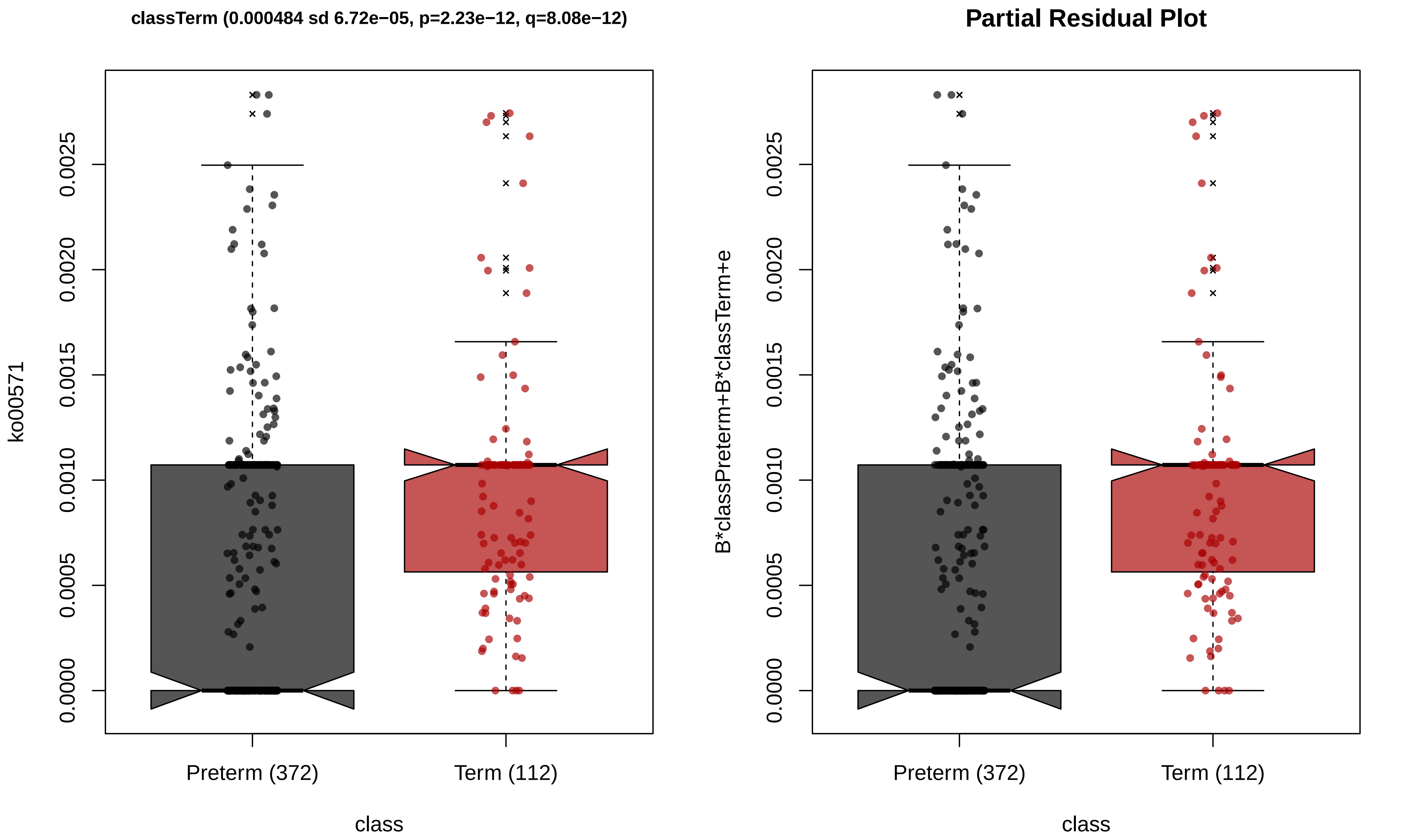

classTerm (0.000484 sd 6.72e−05, p=2.23e−12, q=8.08e−12)
ko00571
0.0000
0.0005
0.0010
0.0015
0.0020
0.0025
Preterm (372)
Term (112)
class
Partial Residual Plot
B*classPreterm+B*classTerm+e
0.0000
0.0005
0.0010
0.0015
0.0020
0.0025
Preterm (372)
Term (112)
class

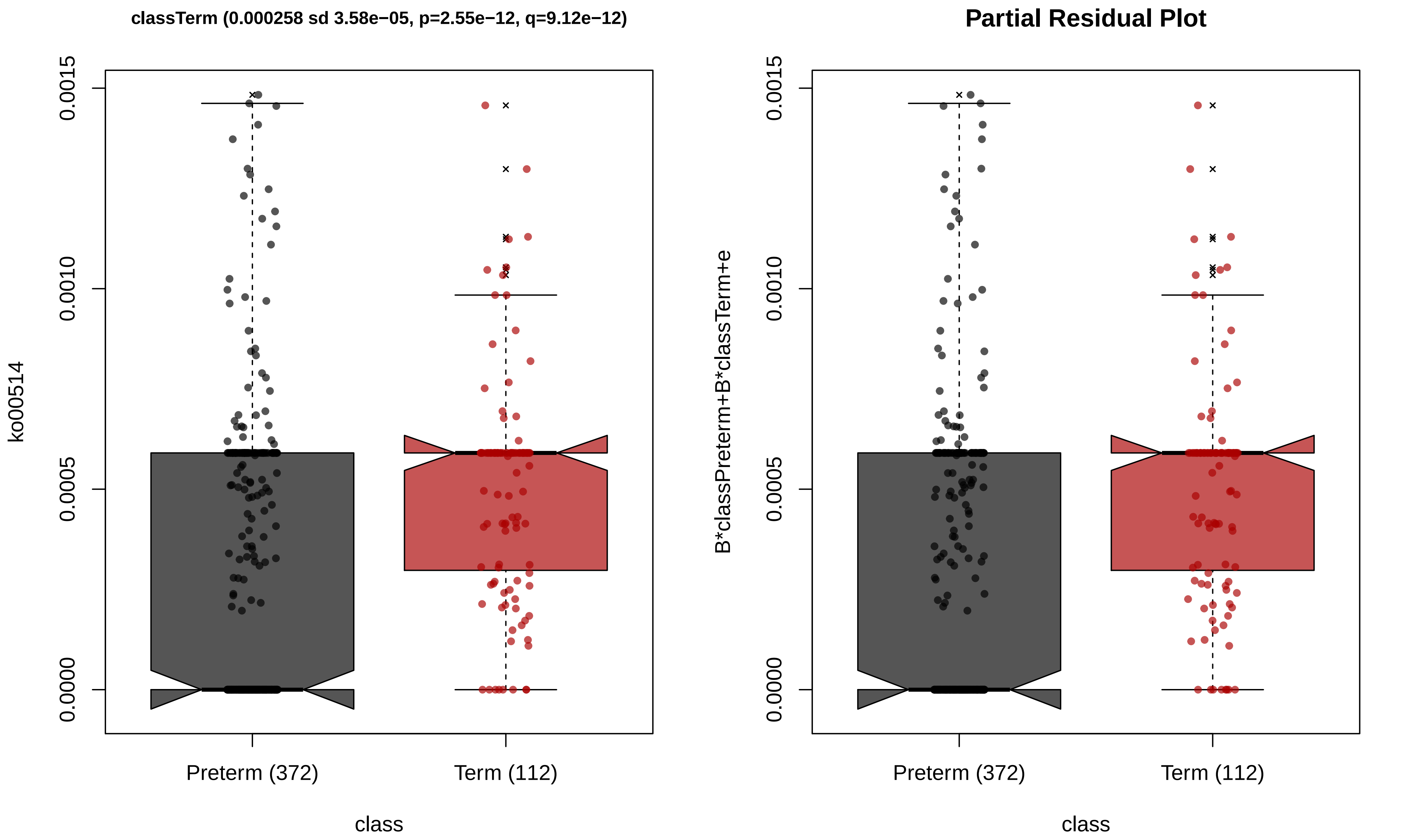

classTerm (0.000258 sd 3.58e−05, p=2.55e−12, q=9.12e−12)
ko00514
0.0000
0.0005
0.0010
0.0015
Preterm (372)
Term (112)
class
Partial Residual Plot
B*classPreterm+B*classTerm+e
0.0000
0.0005
0.0010
0.0015
Preterm (372)
Term (112)
class

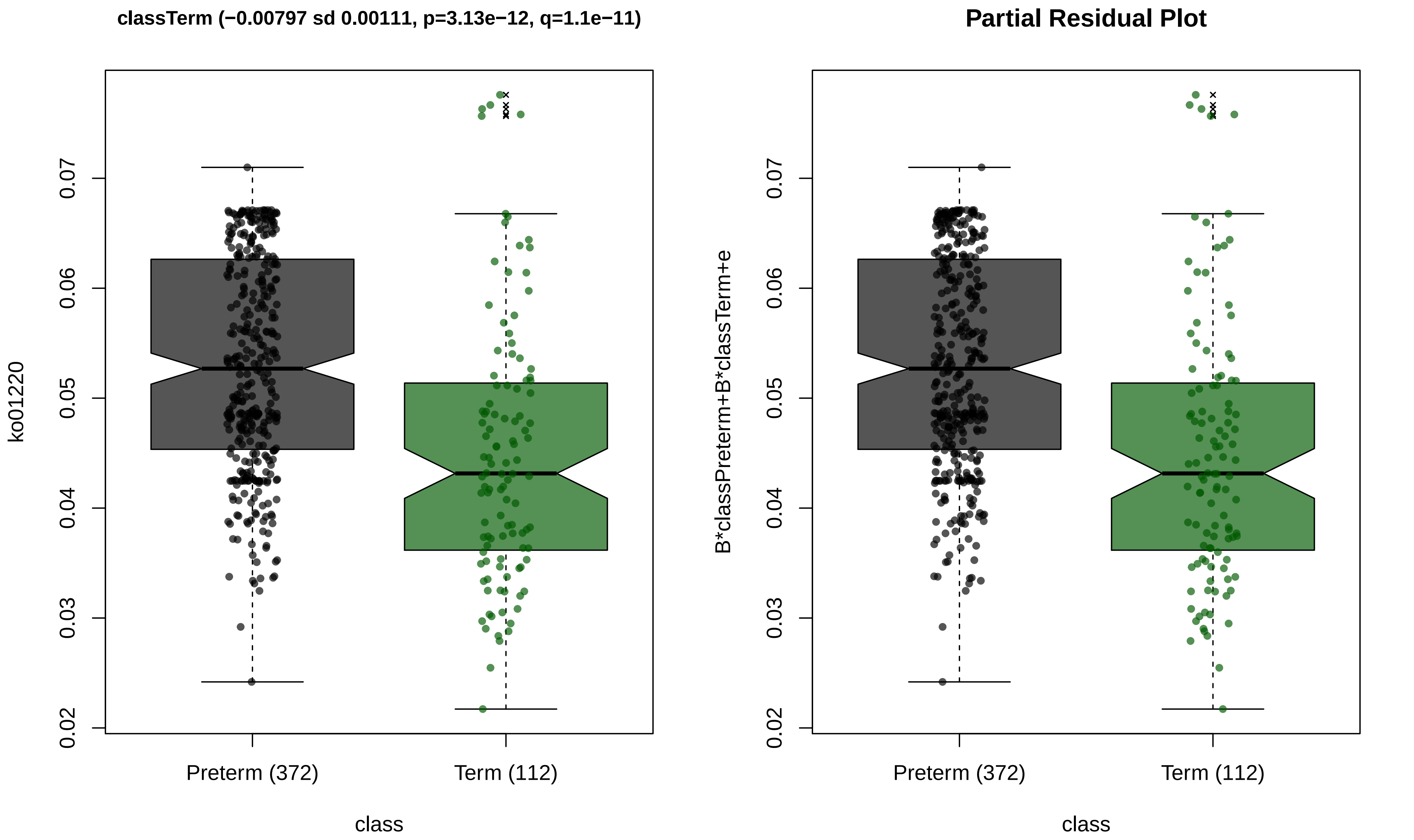

classTerm (−0.00797 sd 0.00111, p=3.13e−12, q=1.1e−11)
Partial Residual Plot
ko01220
B*classPreterm+B*classTerm+e
0.02
0.03
0.04
0.05
0.06
0.07
Preterm (372)
Term (112)
class

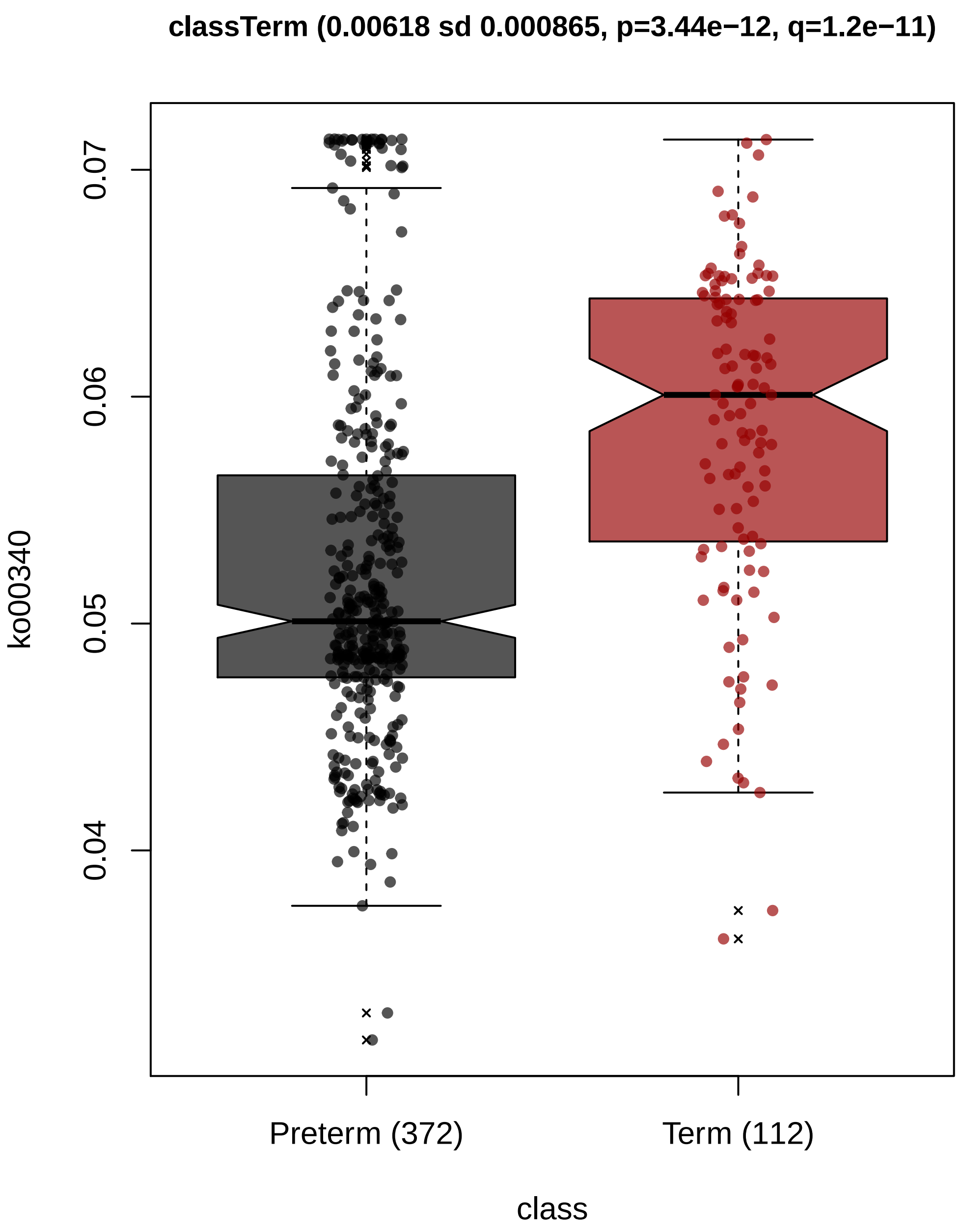

classTerm (0.00618 sd 0.000865, p=3.44e−12, q=1.2e−11)
ko00340
0.04
0.05
0.06
0.07
Preterm (372)
Term (112)
class

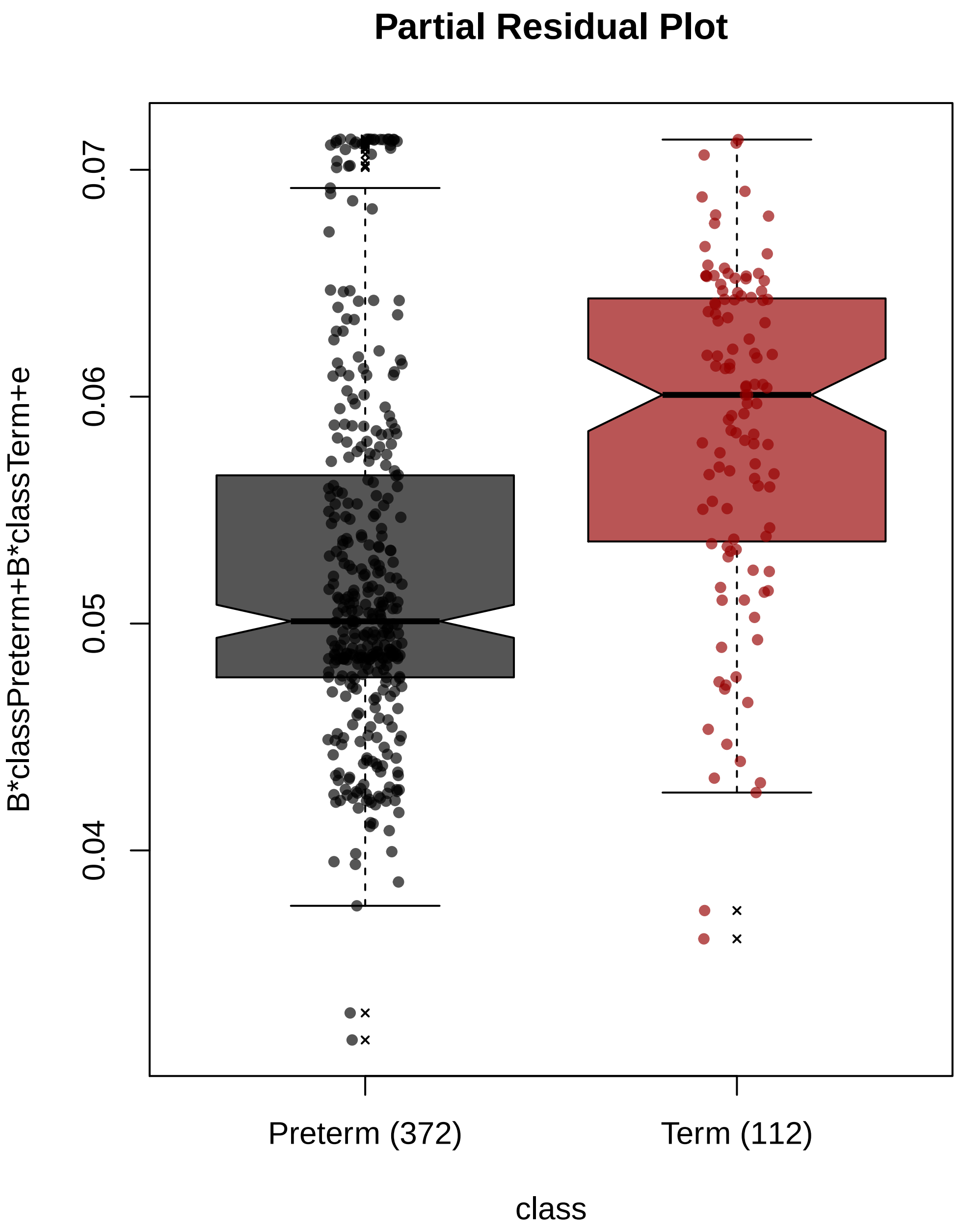

Partial Residual Plot
B*classPreterm+B*classTerm+e
0.04
0.05
0.06
0.07
Preterm (372)
Term (112)
class

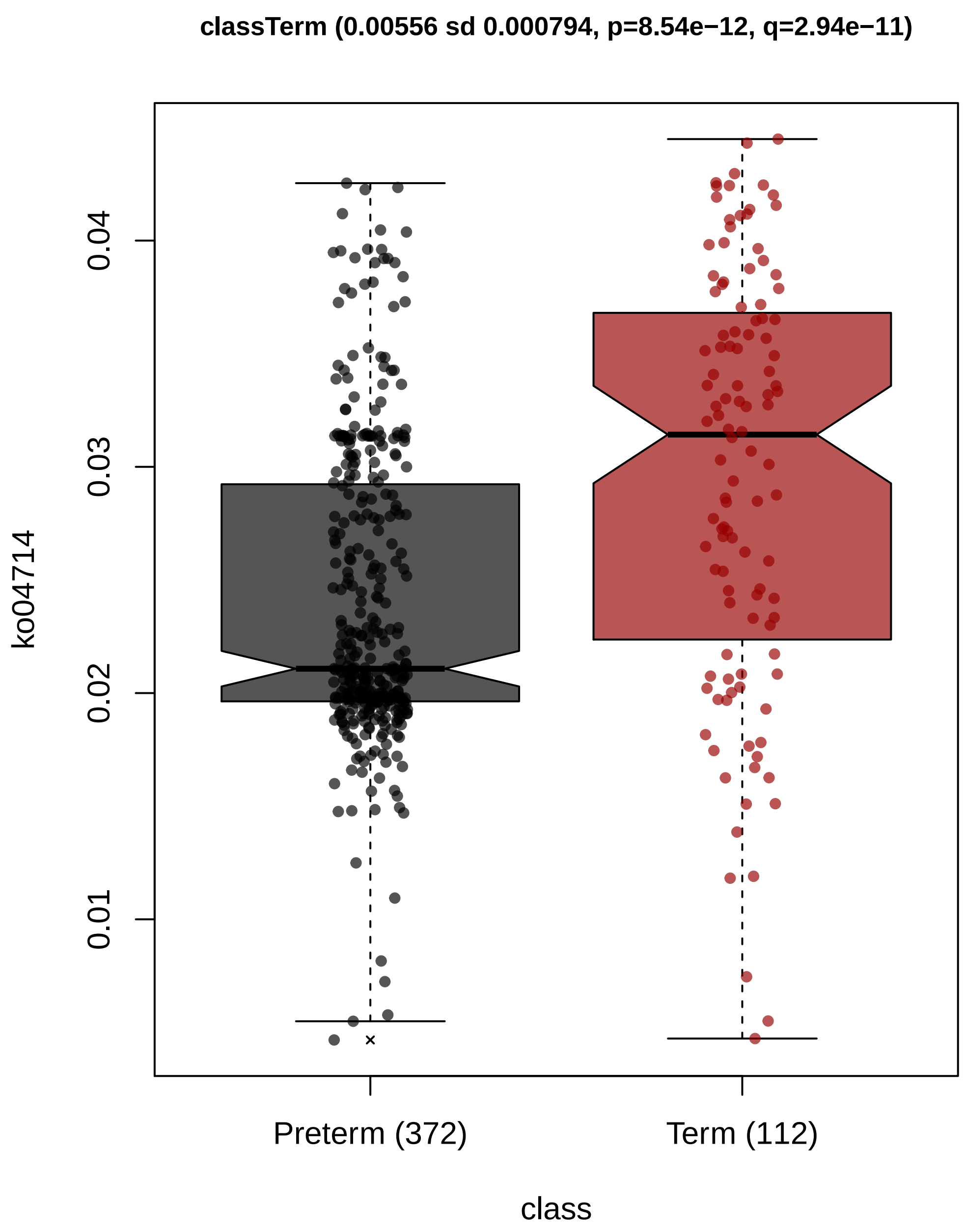
classTerm (0.00556 sd 0.000794, p=8.54e−12, q=2.94e−11)
ko04714
0.01
0.02
0.03
0.04
Preterm (372)
Term (112)
class

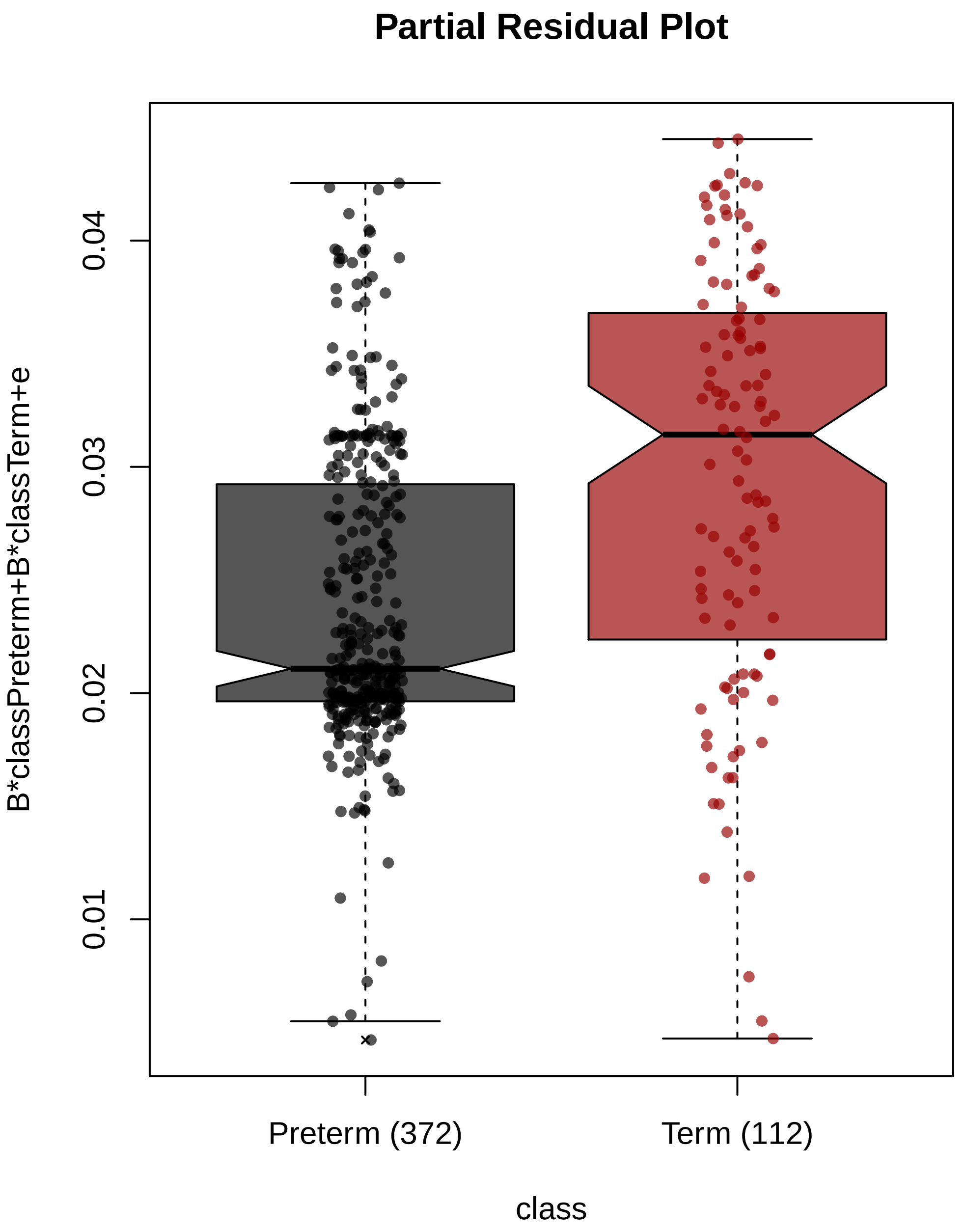
Partial Residual Plot
B*classPreterm+B*classTerm+e
0.01
0.02
0.03
0.04
Preterm (372)
Term (112)
class

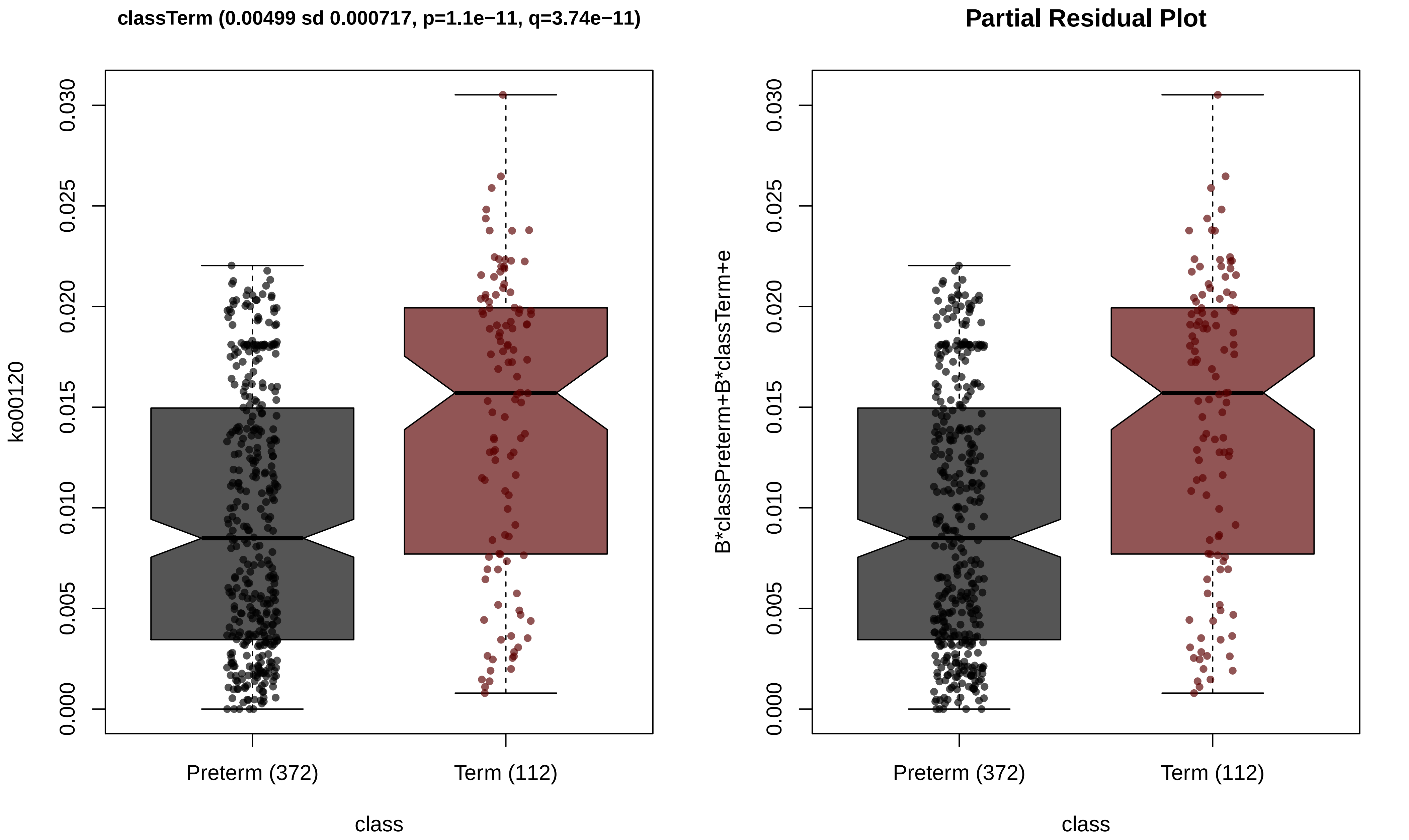

classTerm (0.00499 sd 0.000717, p=1.1e−11, q=3.74e−11)
Partial Residual Plot
ko00120
B*classPreterm+B*classTerm+e
0.000
0.005
0.010
0.015
0.020
0.025
0.030
Preterm (372)
Term (112)
class

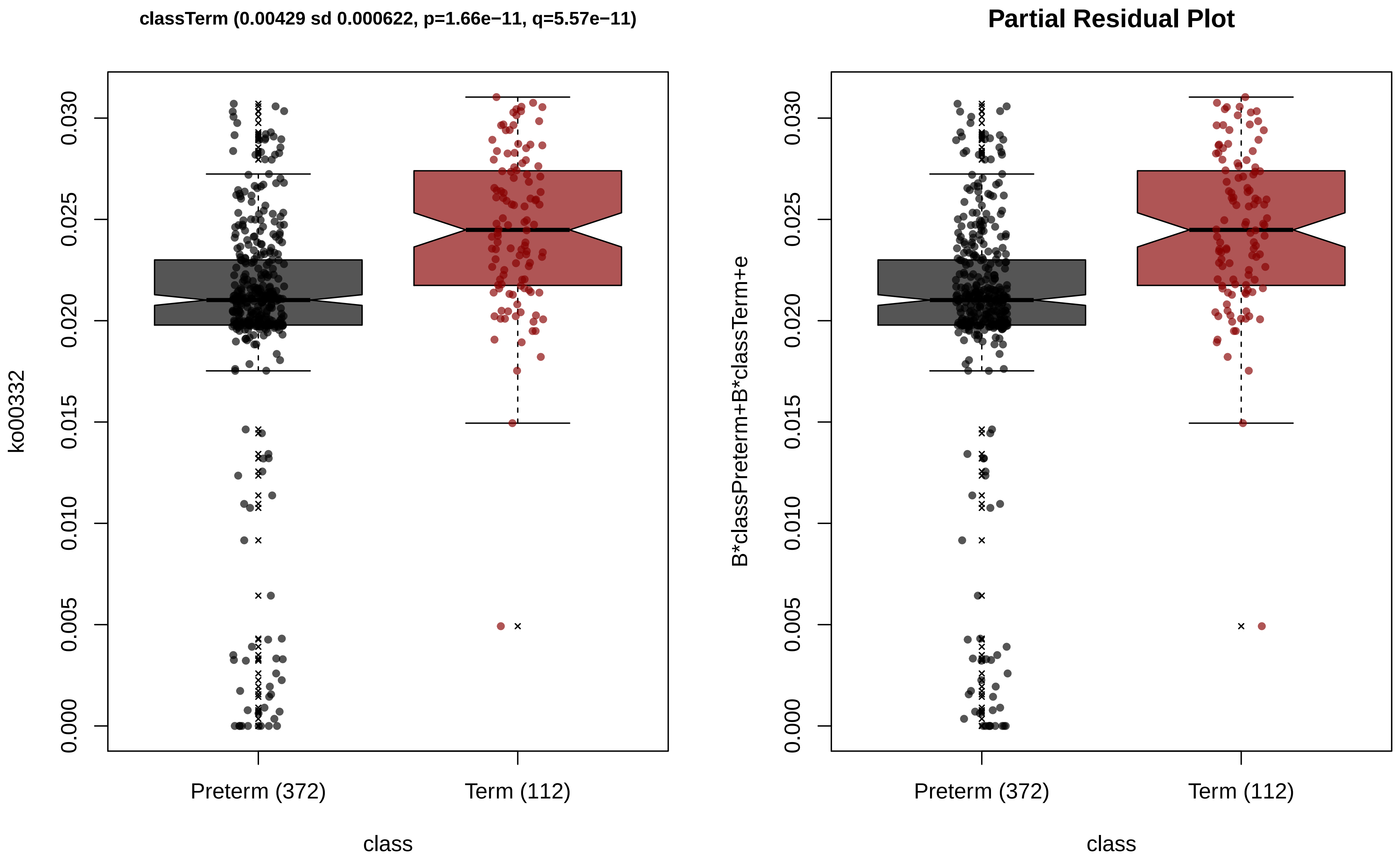

classTerm (0.00429 sd 0.000622, p=1.66e−11, q=5.57e−11)
Partial Residual Plot
ko00332
B*classPreterm+B*classTerm+e
0.000
0.005
0.010
0.015
0.020
0.025
0.030
Preterm (372)
Term (112)
class

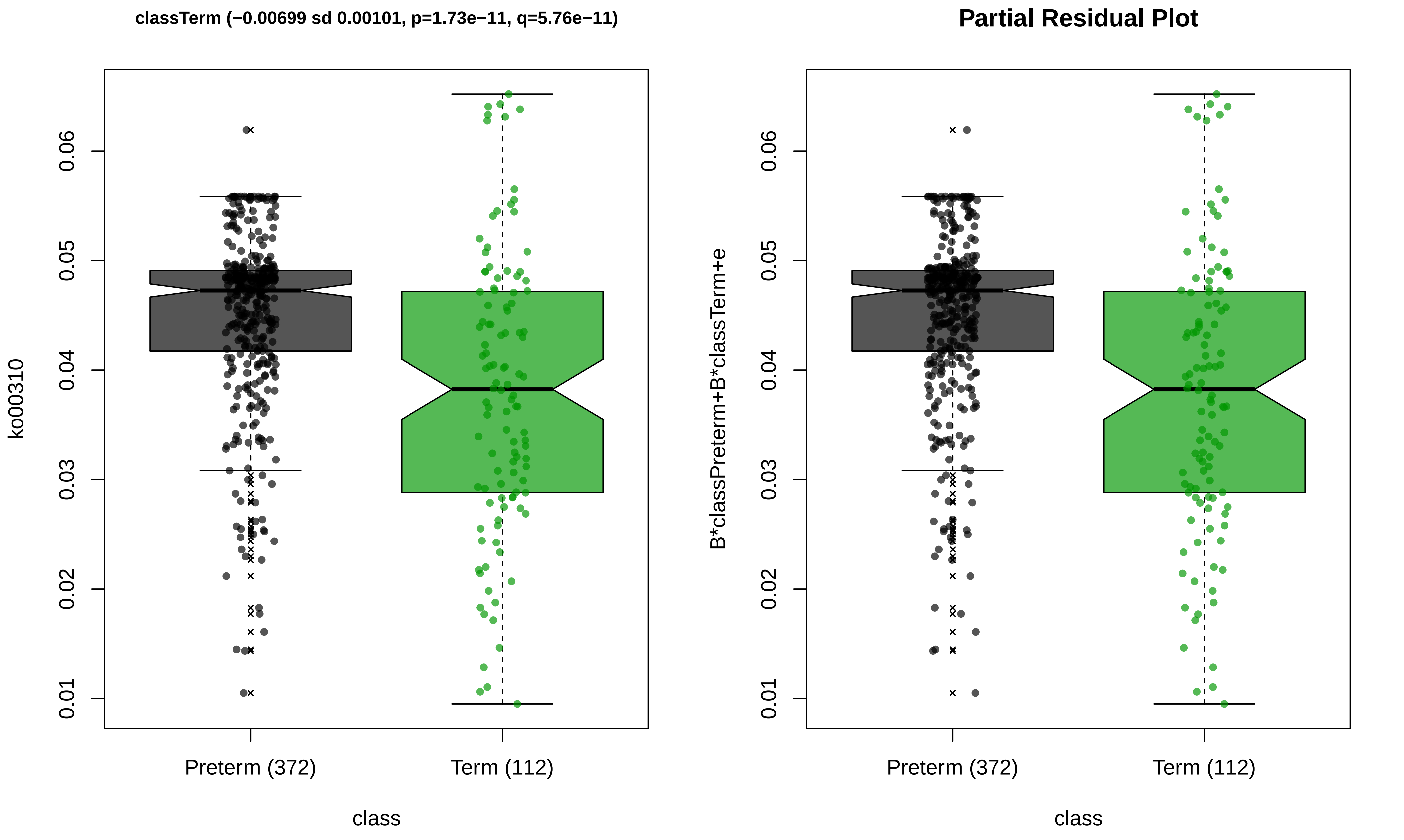

classTerm (−0.00699 sd 0.00101, p=1.73e−11, q=5.76e−11)
Partial Residual Plot
ko00310
B*classPreterm+B*classTerm+e
0.01
0.02
0.03
0.04
0.05
0.06
Preterm (372)
Term (112)
class

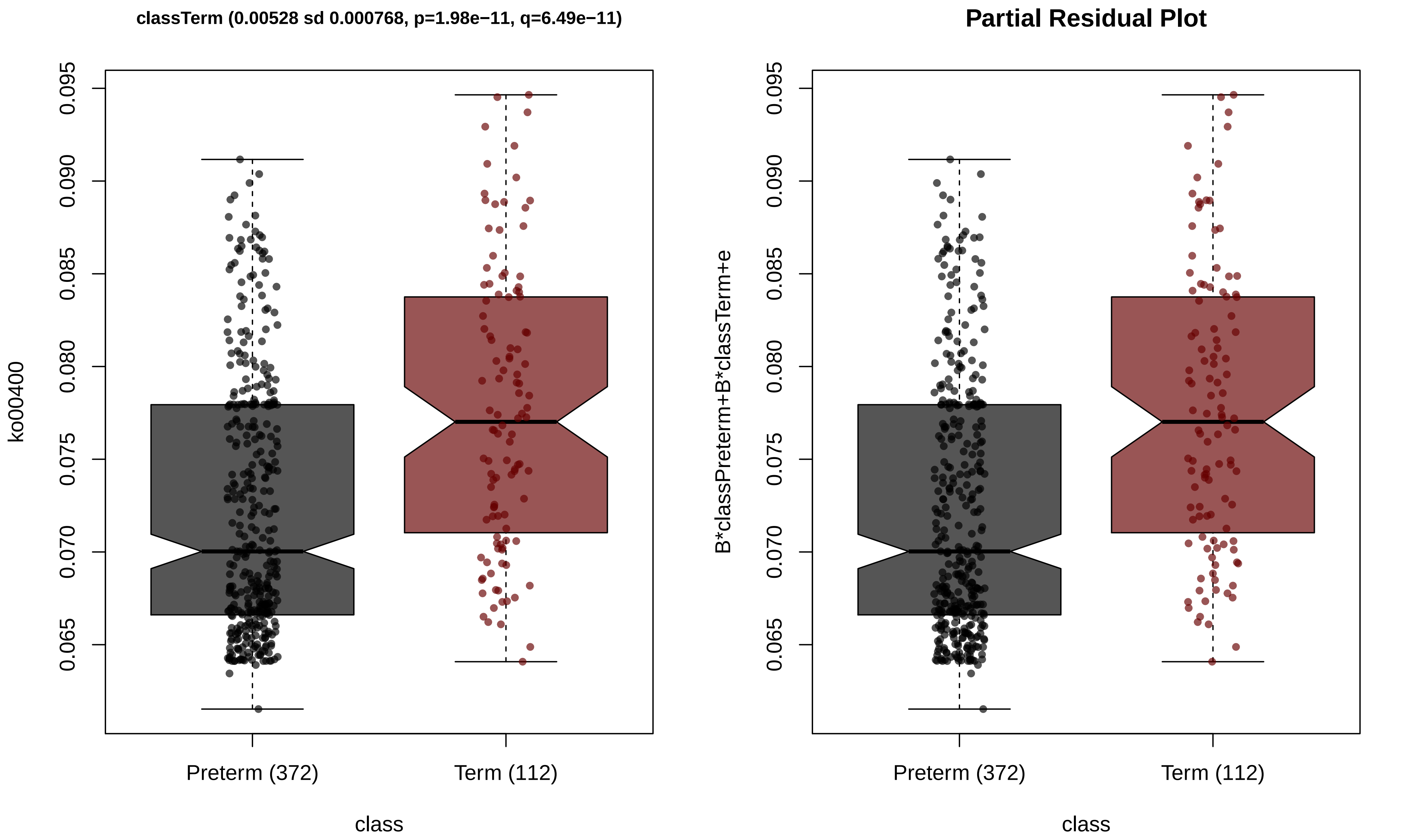
classTerm (0.00528 sd 0.000768, p=1.98e−11, q=6.49e−11)
Partial Residual Plot
ko00400
B*classPreterm+B*classTerm+e
0.065
0.070
0.075
0.080
0.085
0.090
0.095
Preterm (372)
Term (112)
class

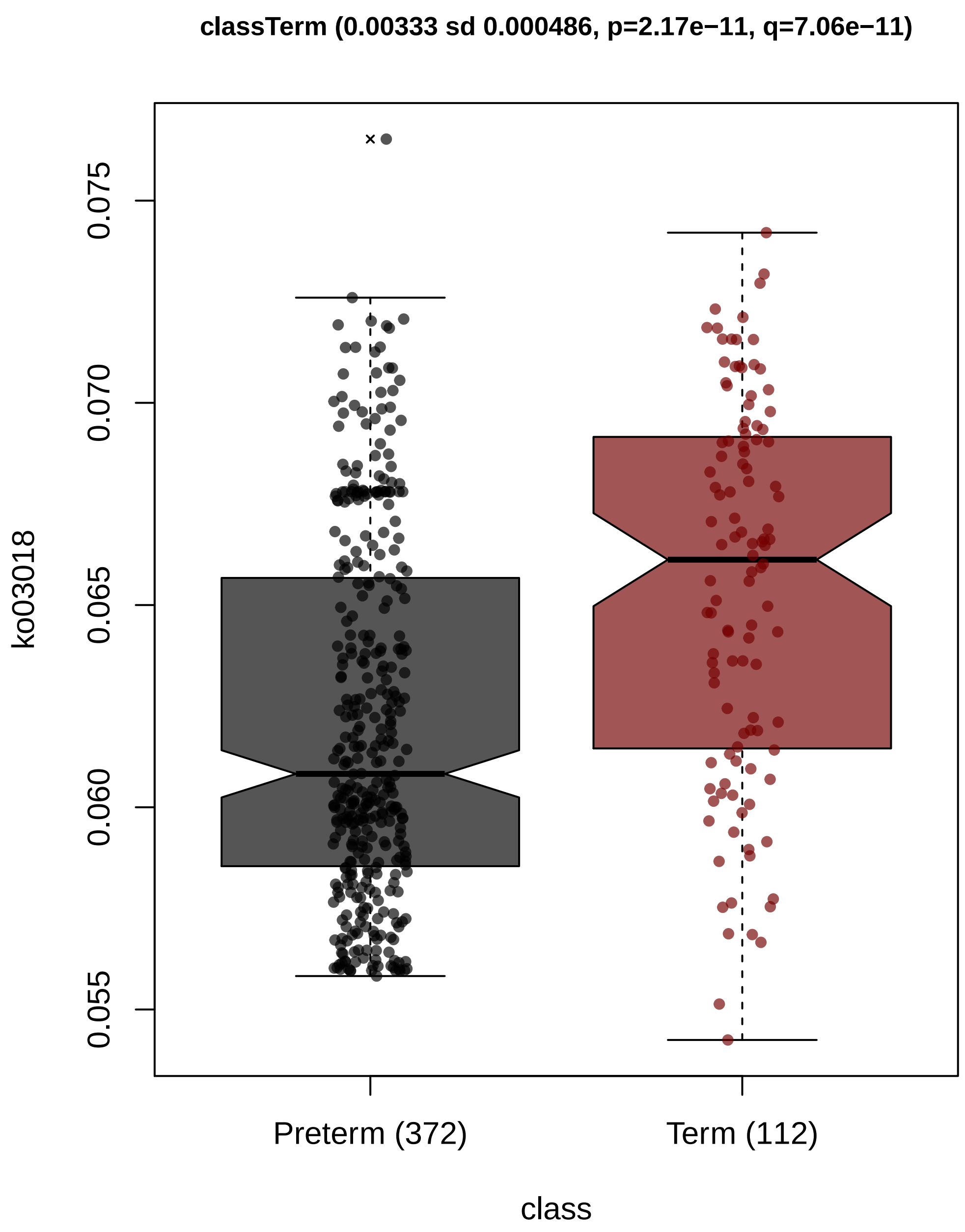

classTerm (0.00333 sd 0.000486, p=2.17e−11, q=7.06e−11)
ko03018
0.055
0.060
0.065
0.070
0.075
Preterm (372)
Term (112)
class

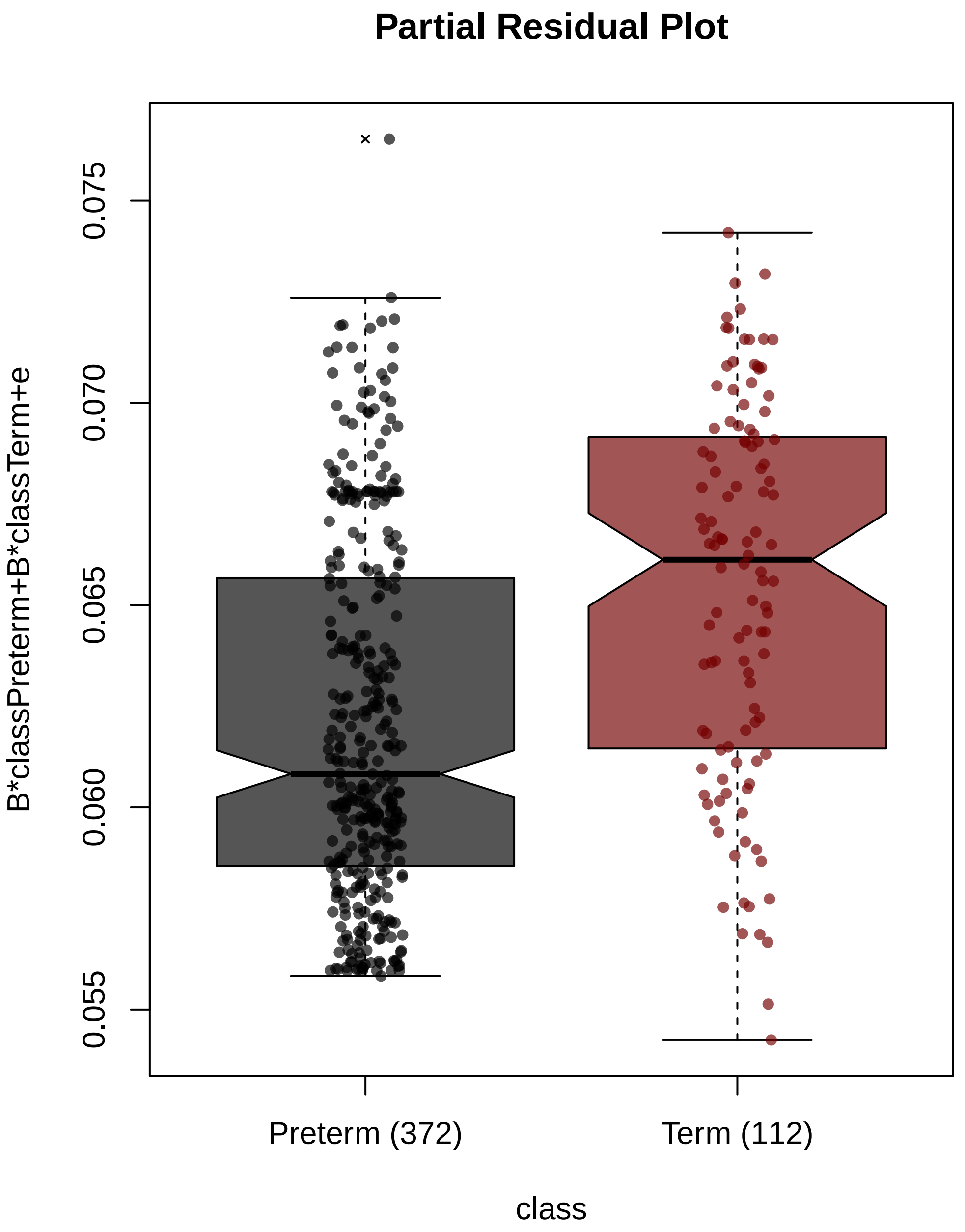

Partial Residual Plot
B*classPreterm+B*classTerm+e
0.055
0.060
0.065
0.070
0.075
Preterm (372)
Term (112)
class

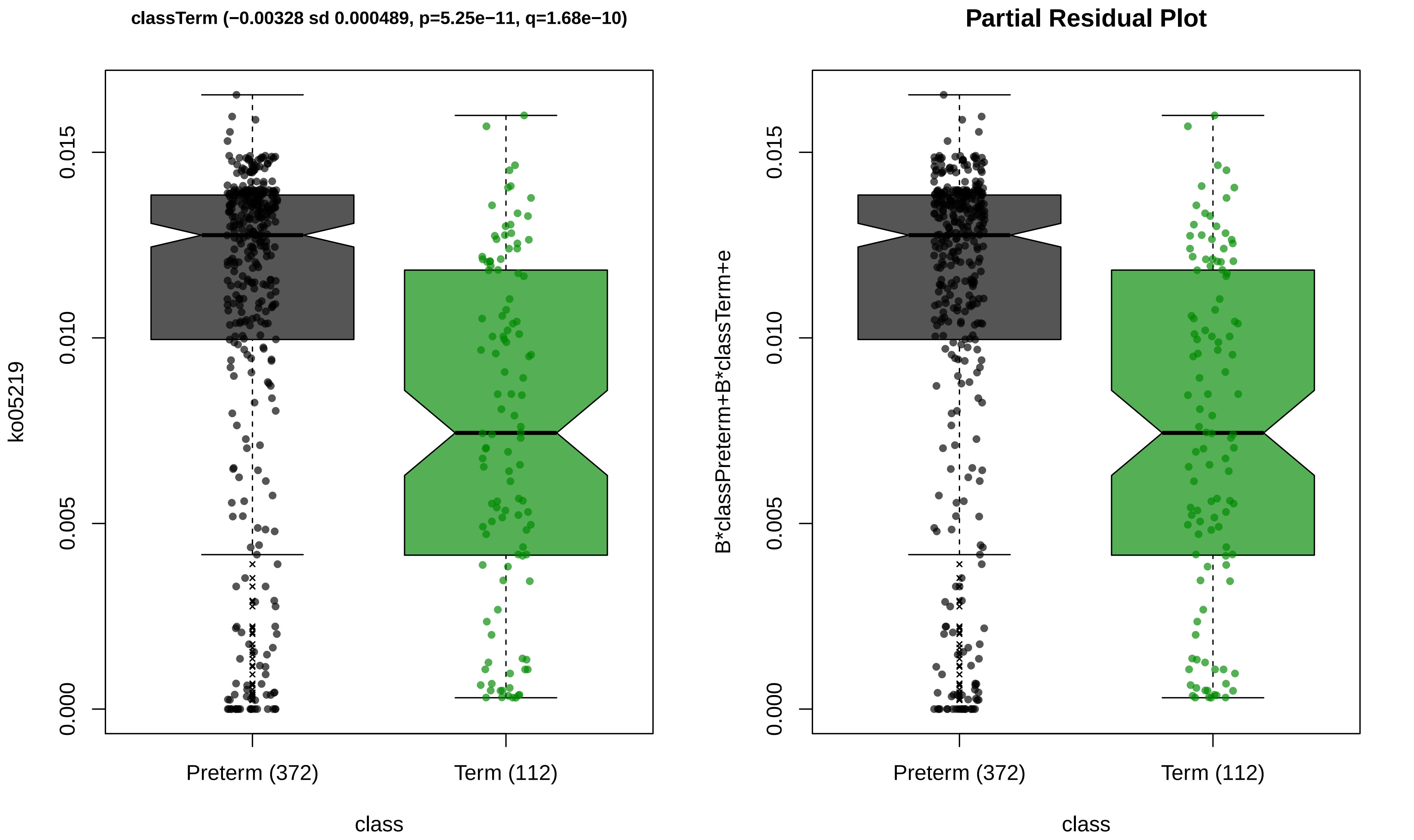
classTerm (−0.00328 sd 0.000489, p=5.25e−11, q=1.68e−10)
ko05219
0.000
0.005
0.010
0.015
Preterm (372)
Term (112)
class
Partial Residual Plot
B*classPreterm+B*classTerm+e
0.000
0.005
0.010
0.015
Preterm (372)
Term (112)
class

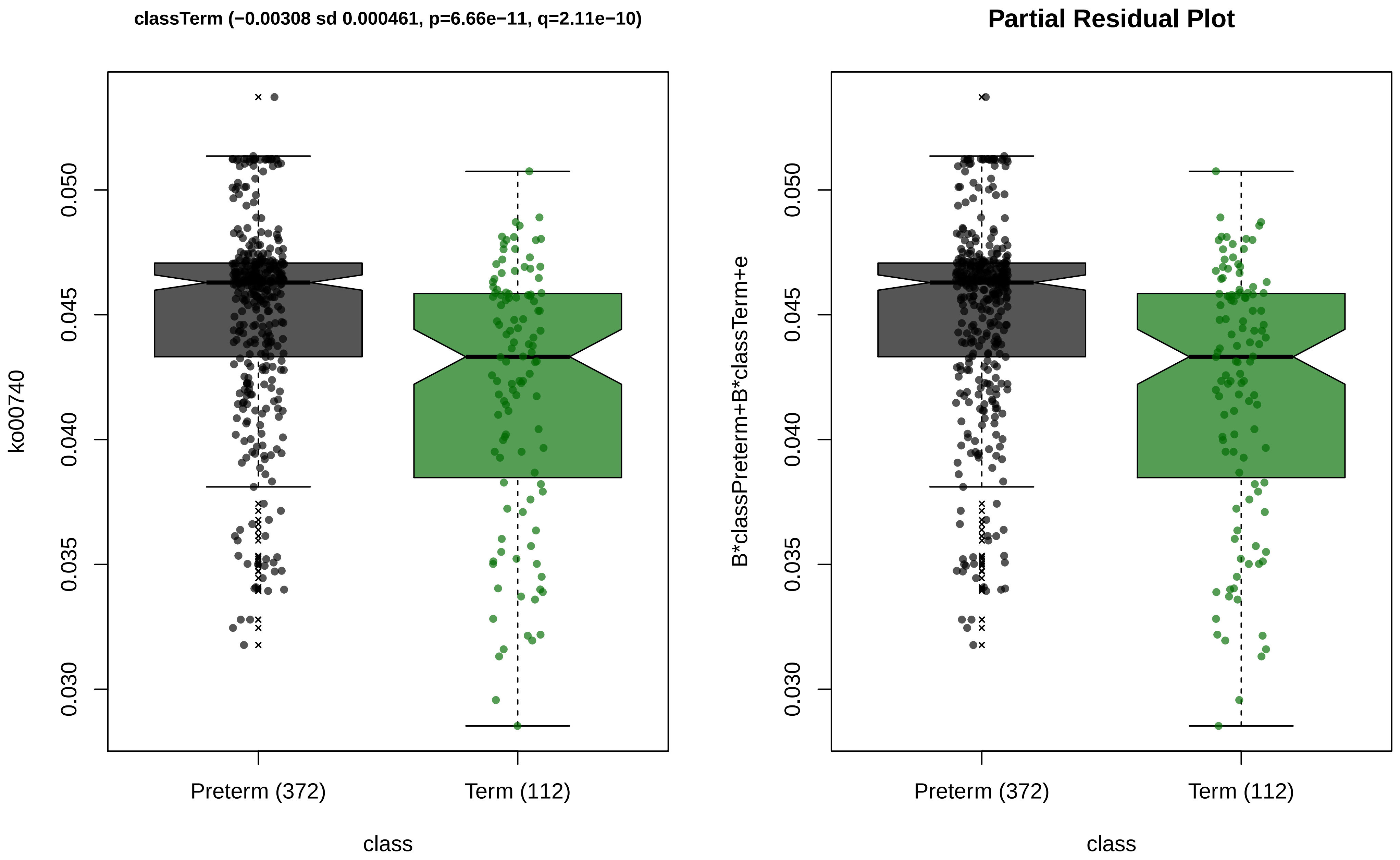

classTerm (−0.00308 sd 0.000461, p=6.66e−11, q=2.11e−10)
ko00740
0.030
0.035
0.040
0.045
0.050
Preterm (372)
Term (112)
class
Partial Residual Plot
B*classPreterm+B*classTerm+e
0.030
0.035
0.040
0.045
0.050
Preterm (372)
Term (112)
class

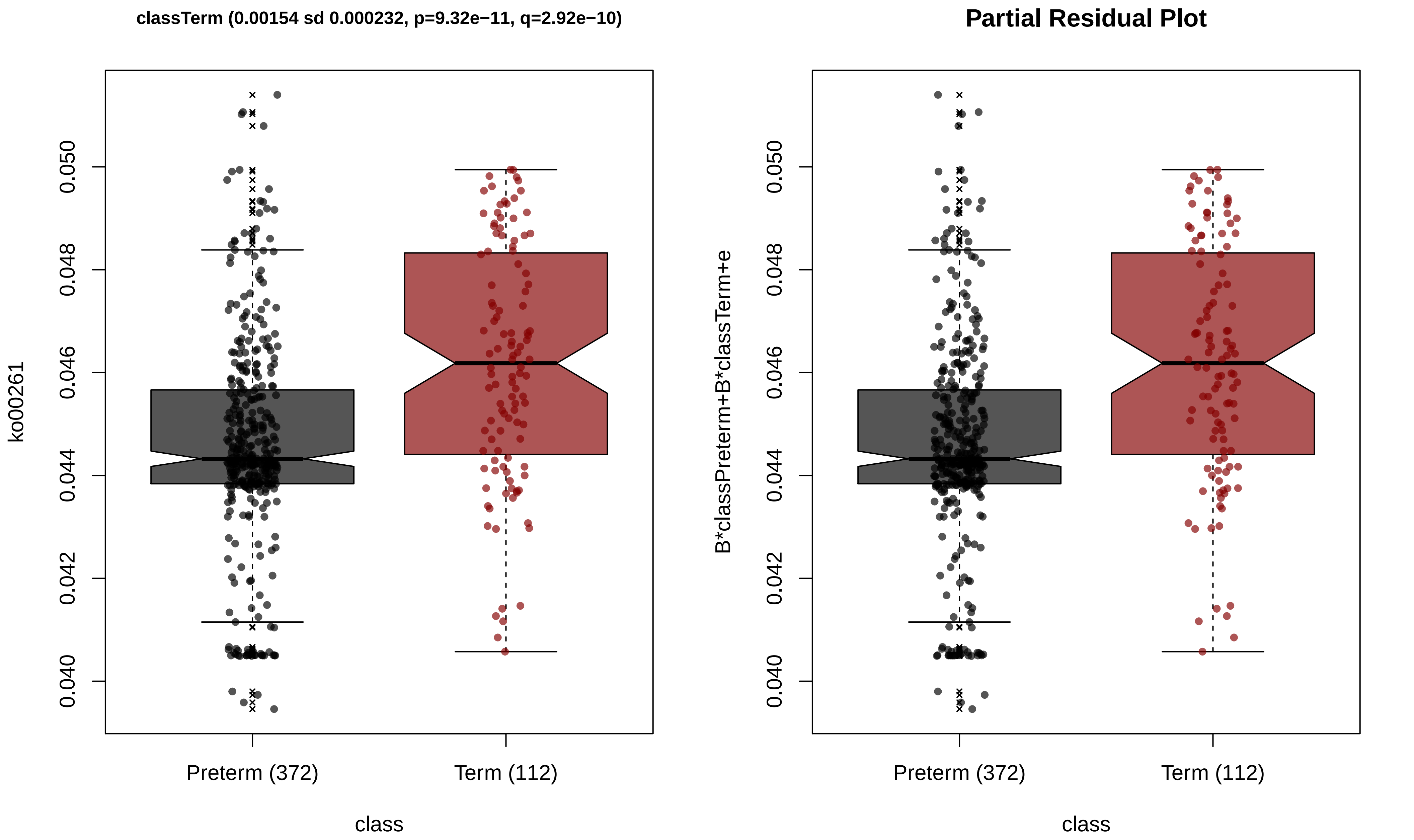

classTerm (0.00154 sd 0.000232, p=9.32e−11, q=2.92e−10)
Partial Residual Plot
ko00261
B*classPreterm+B*classTerm+e
0.040
0.042
0.044
0.046
0.048
0.050
Preterm (372)
Term (112)
class

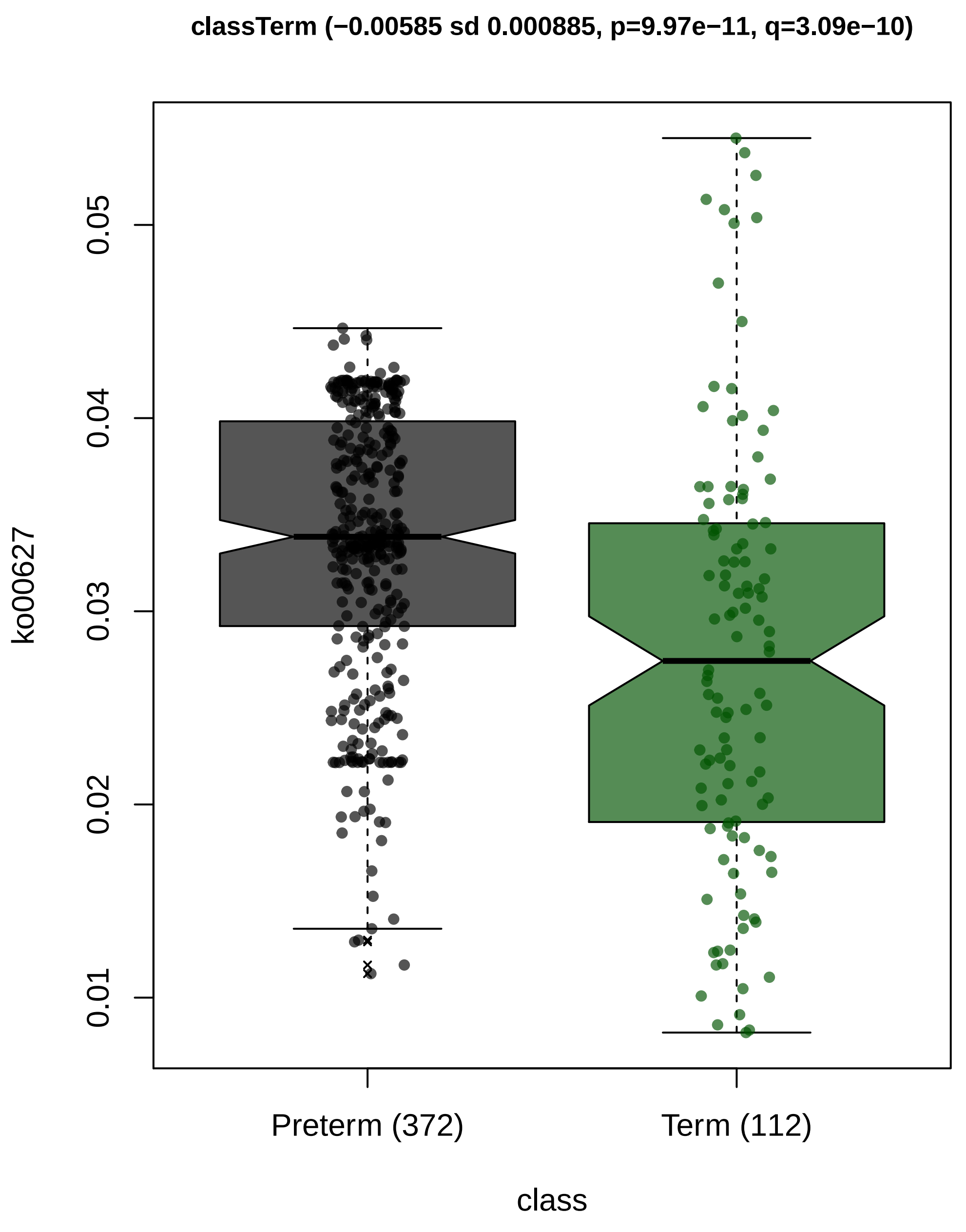

classTerm (−0.00585 sd 0.000885, p=9.97e−11, q=3.09e−10)
ko00627
0.01
0.02
0.03
0.04
0.05
Preterm (372)
Term (112)
class

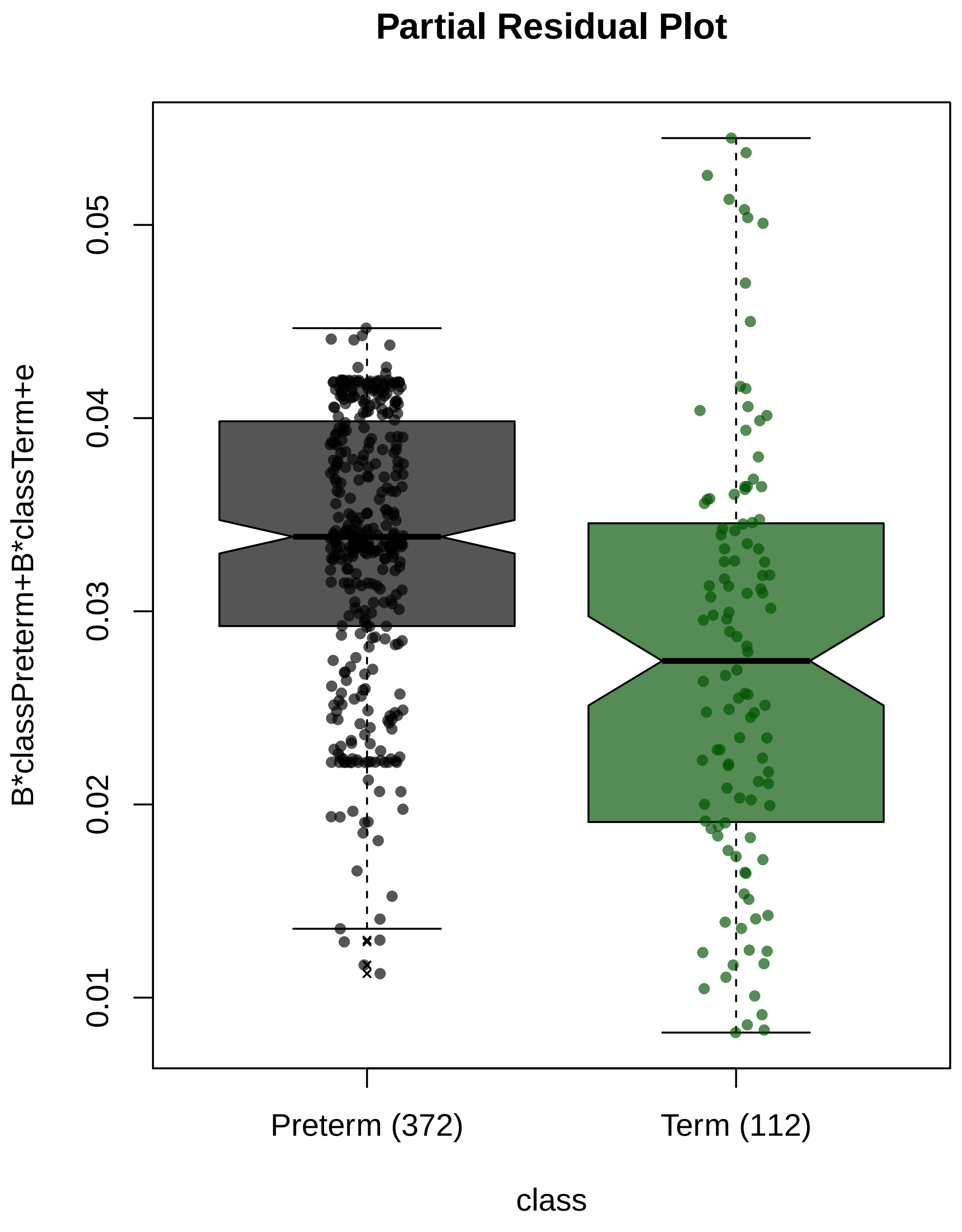

Partial Residual Plot
B*classPreterm+B*classTerm+e
0.01
0.02
0.03
0.04
0.05
Preterm (372)
Term (112)
class

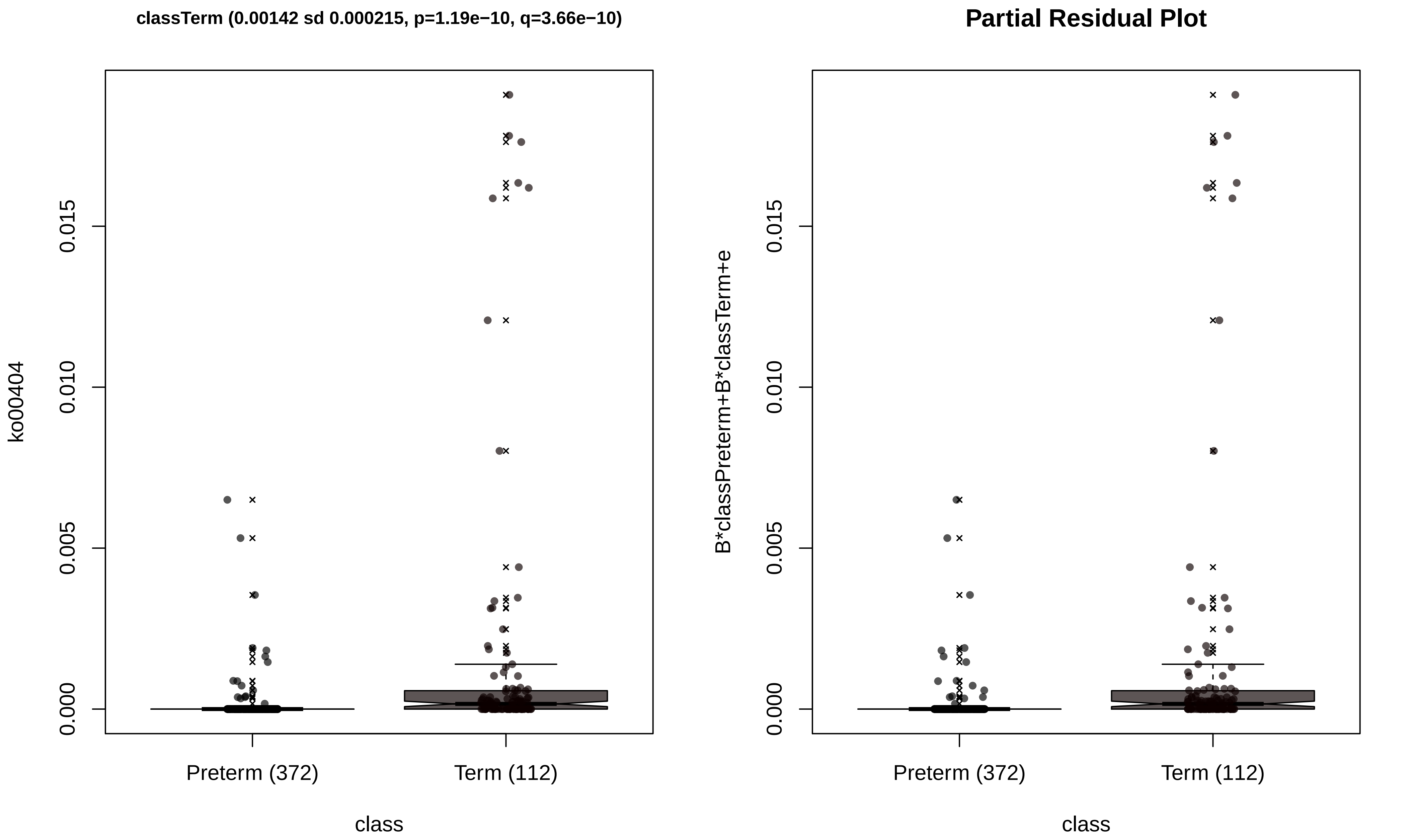

classTerm (0.00142 sd 0.000215, p=1.19e−10, q=3.66e−10)
ko00404
0.000
0.005
0.010
0.015
Preterm (372)
Term (112)
class
Partial Residual Plot
B*classPreterm+B*classTerm+e
0.000
0.005
0.010
0.015
Preterm (372)
Term (112)
class

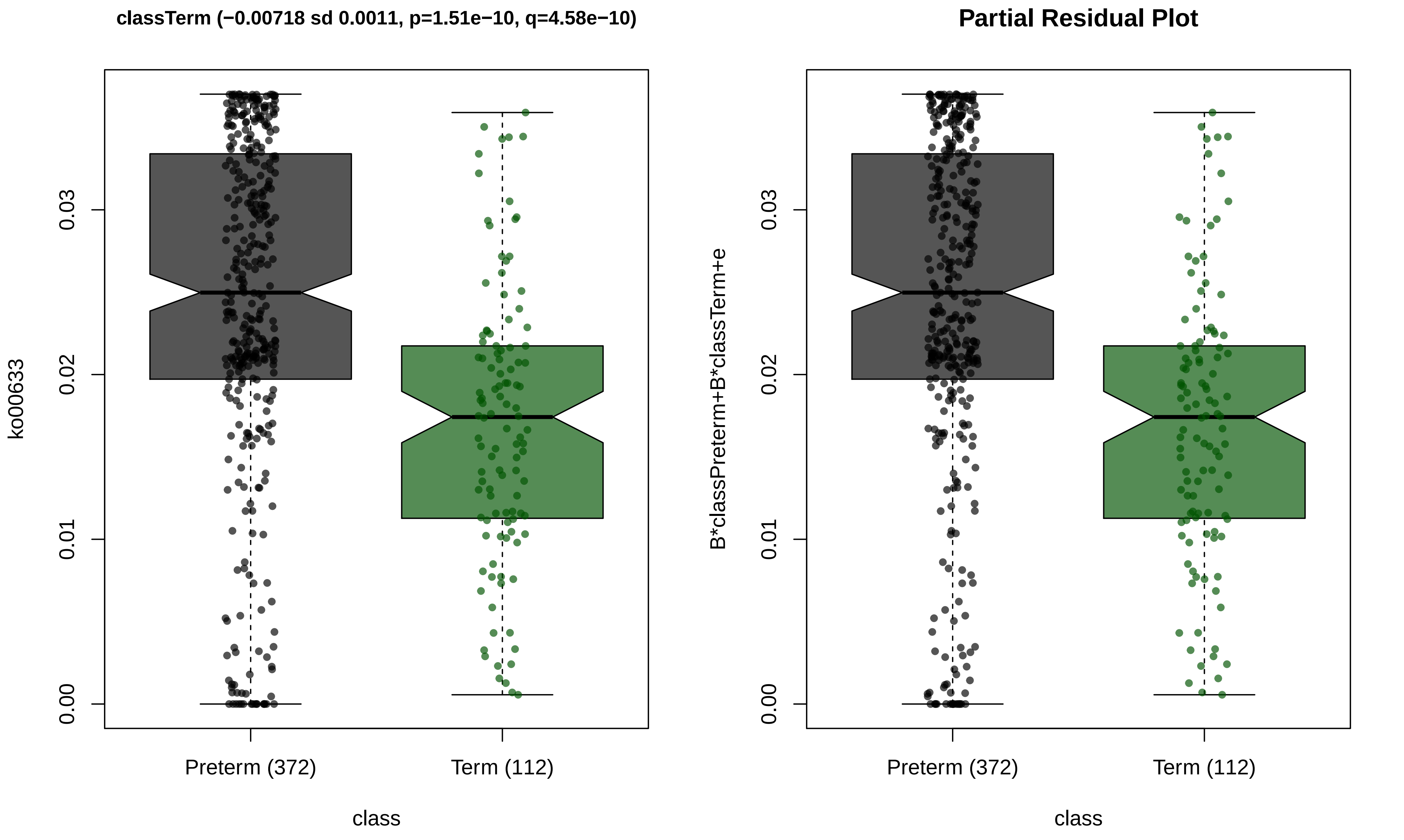
classTerm (−0.00718 sd 0.0011, p=1.51e−10, q=4.58e−10)
ko00633
0.00
0.01
0.02
0.03
Preterm (372)
Term (112)
class
Partial Residual Plot
B*classPreterm+B*classTerm+e
0.00
0.01
0.02
0.03
Preterm (372)
Term (112)
class

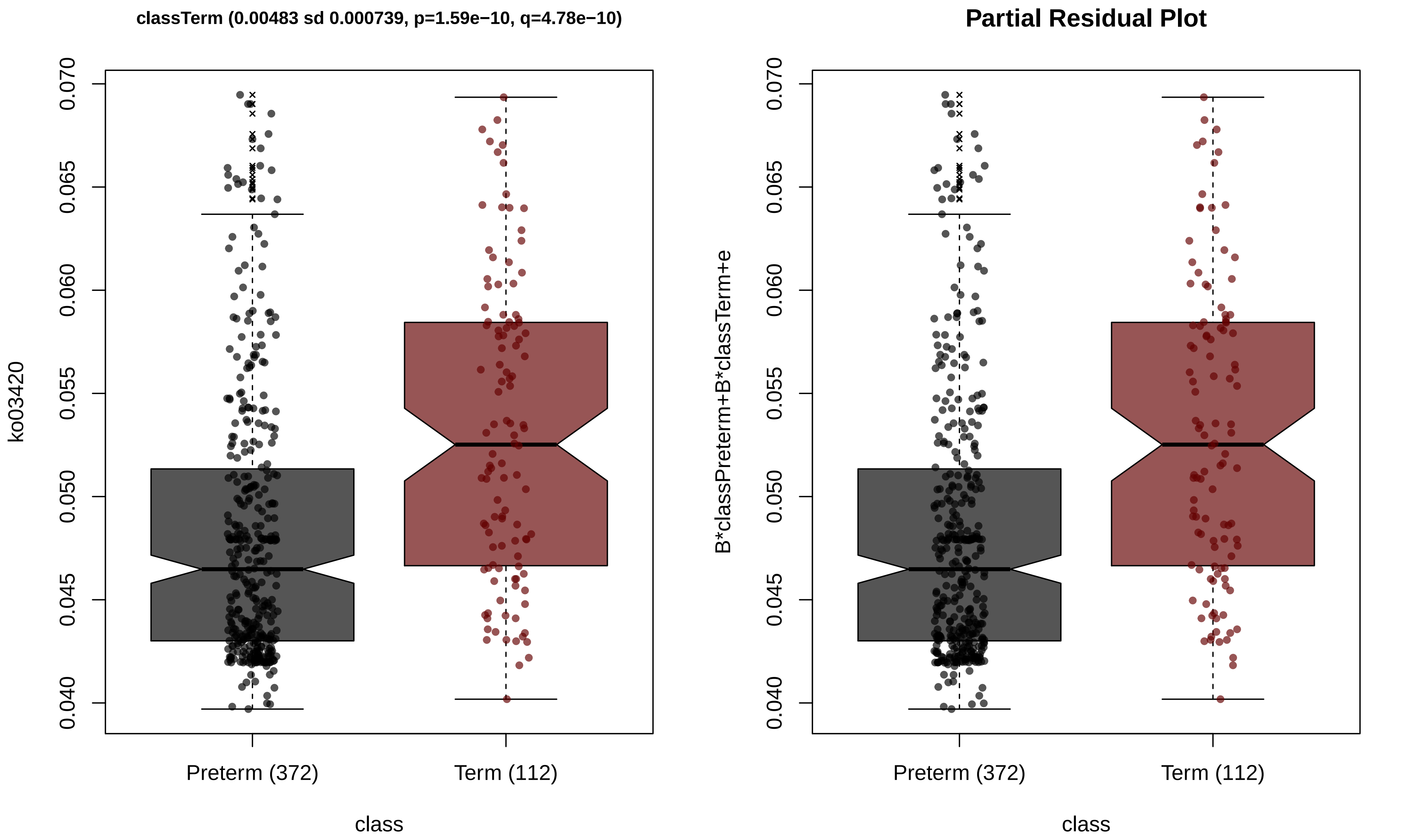

classTerm (0.00483 sd 0.000739, p=1.59e−10, q=4.78e−10)
ko03420
0.040
0.045
0.050
0.055
0.060
0.065
0.070
Preterm (372)
Term (112)
class
Partial Residual Plot
B*classPreterm+B*classTerm+e
0.040
0.045
0.050
0.055
0.060
0.065
0.070
Preterm (372)
Term (112)
class

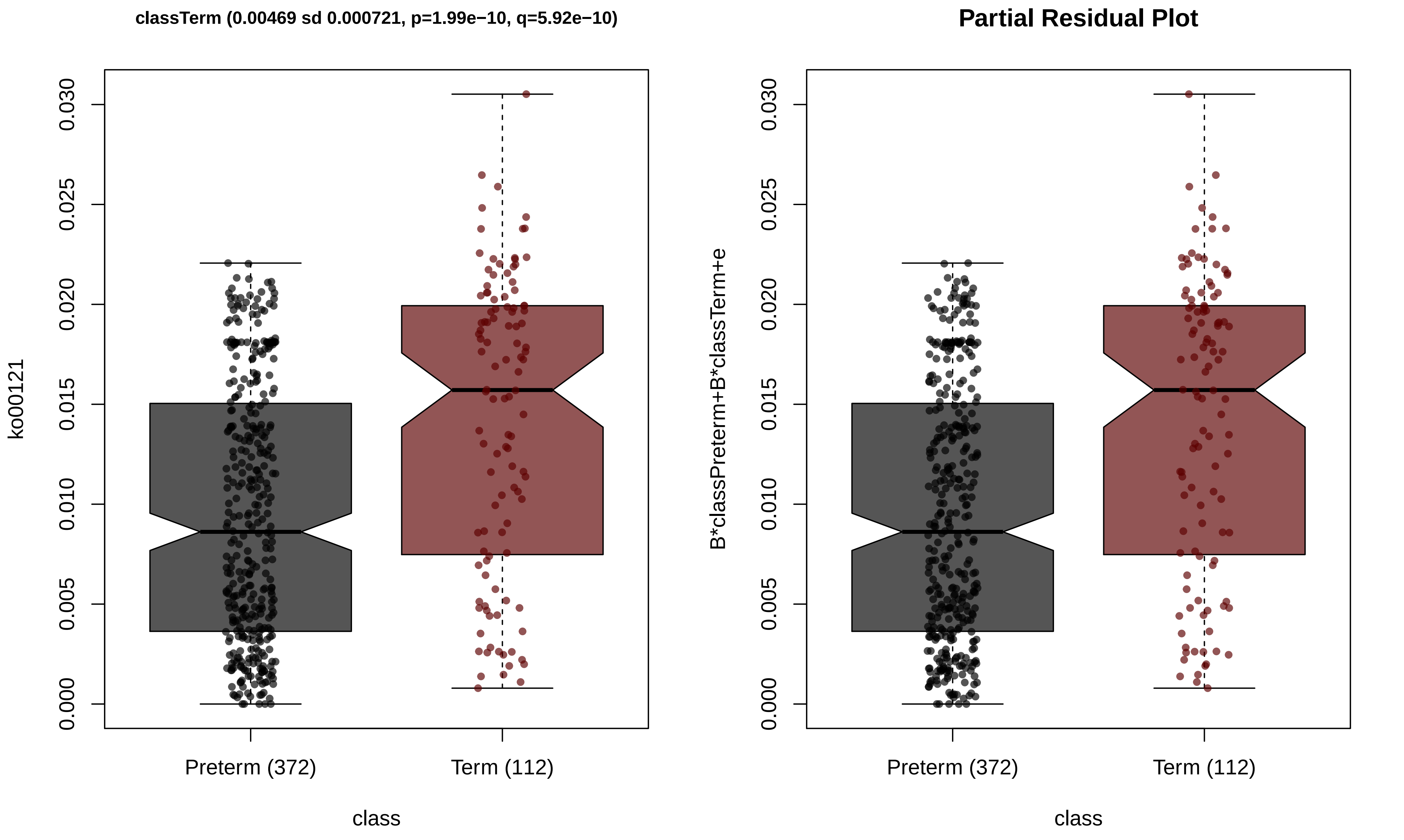

classTerm (0.00469 sd 0.000721, p=1.99e−10, q=5.92e−10)
ko00121
0.000
0.005
0.010
0.015
0.020
0.025
0.030
Preterm (372)
Term (112)
class
Partial Residual Plot
B*classPreterm+B*classTerm+e
0.000
0.005
0.010
0.015
0.020
0.025
0.030
Preterm (372)
Term (112)
class

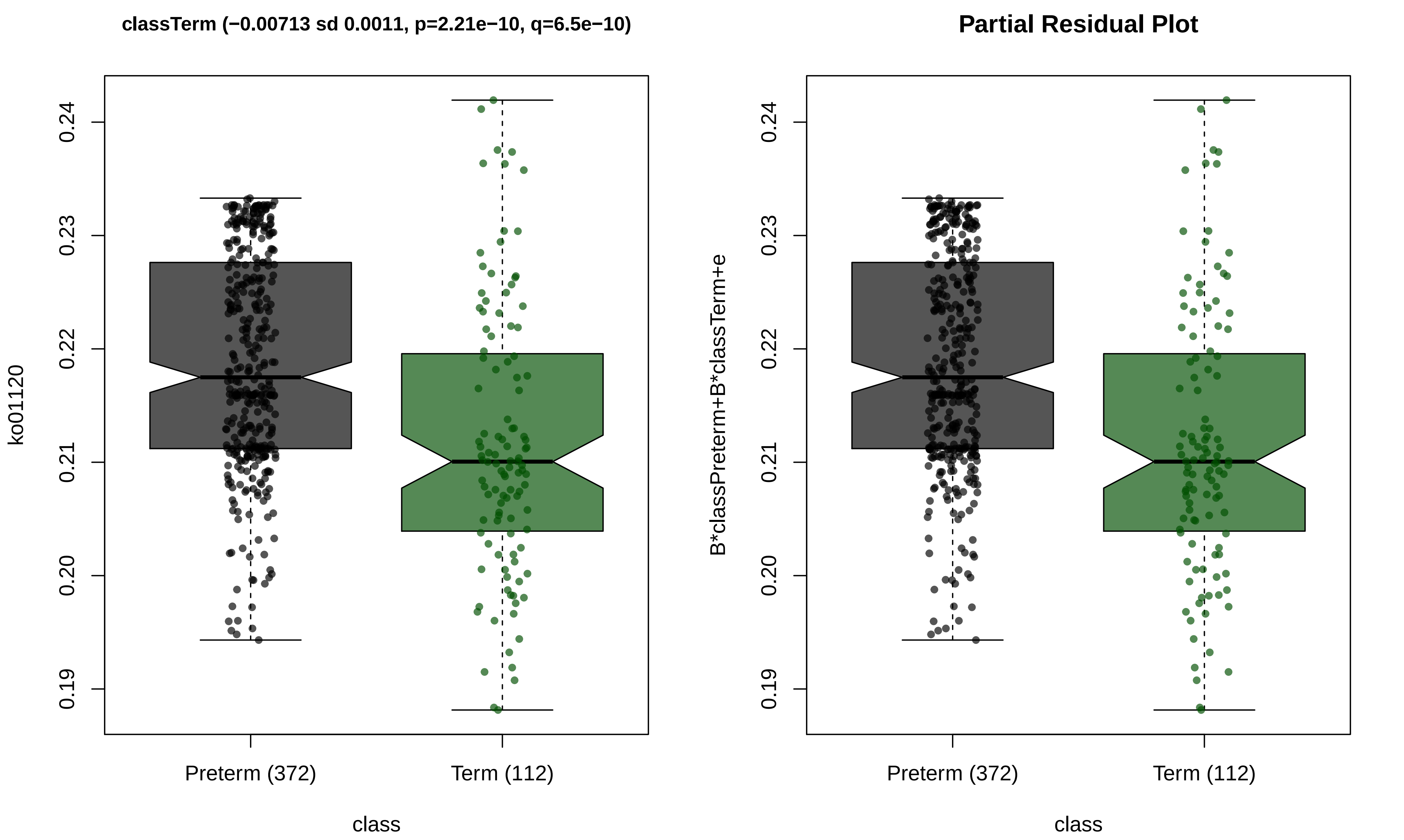
classTerm (−0.00713 sd 0.0011, p=2.21e−10, q=6.5e−10)
Partial Residual Plot
ko01120
B*classPreterm+B*classTerm+e
0.19
0.20
0.21
0.22
0.23
0.24
Preterm (372)
Term (112)
class

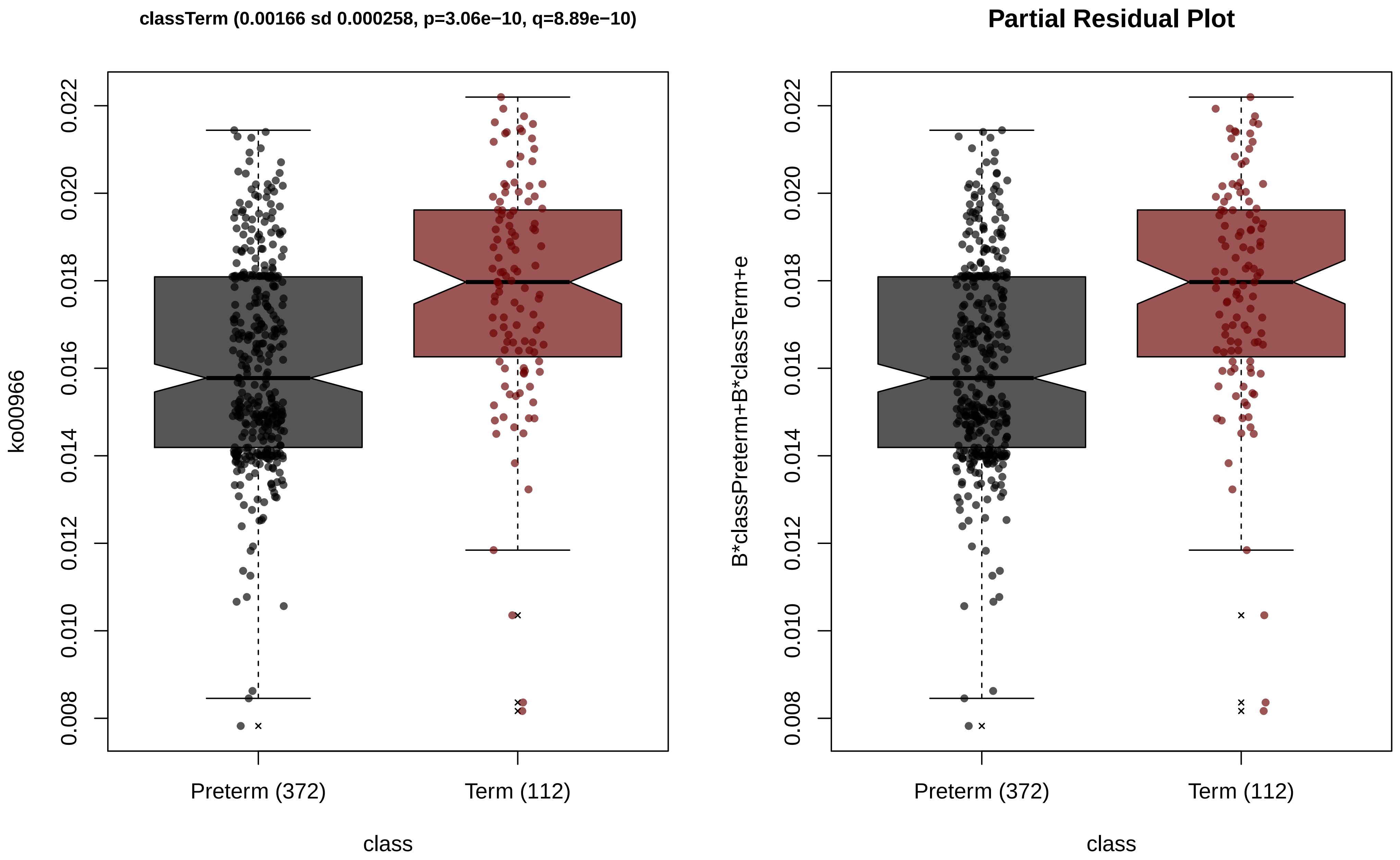

classTerm (0.00166 sd 0.000258, p=3.06e−10, q=8.89e−10)
Partial Residual Plot
ko00966
B*classPreterm+B*classTerm+e
0.008
0.010
0.012
0.014
0.016
0.018
0.020
0.022
Preterm (372)
Term (112)
class

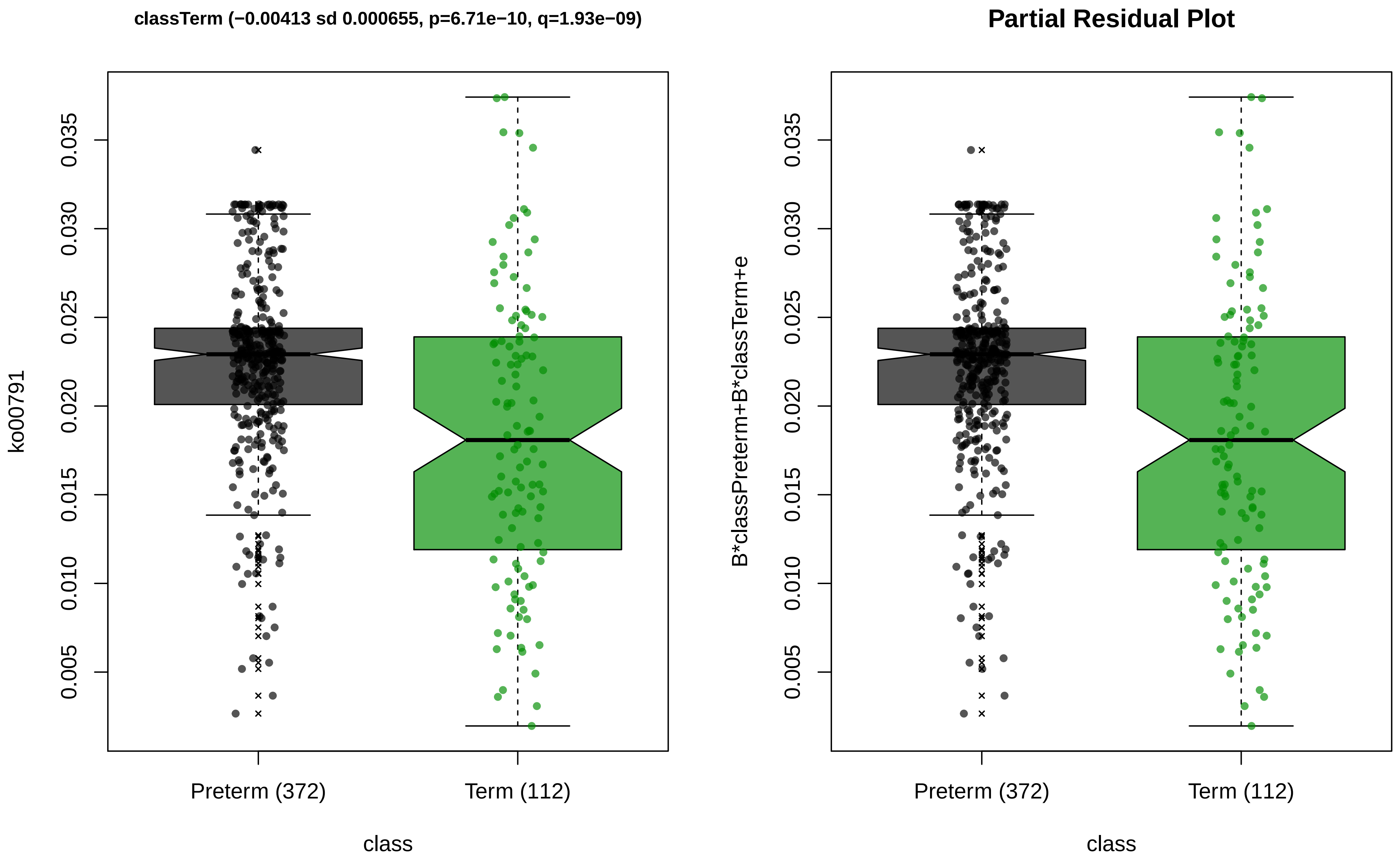

classTerm (−0.00413 sd 0.000655, p=6.71e−10, q=1.93e−09)
ko00791
0.005
0.010
0.015
0.020
0.025
0.030
0.035
Preterm (372)
Term (112)
class
Partial Residual Plot
B*classPreterm+B*classTerm+e
0.005
0.010
0.015
0.020
0.025
0.030
0.035
Preterm (372)
Term (112)
class

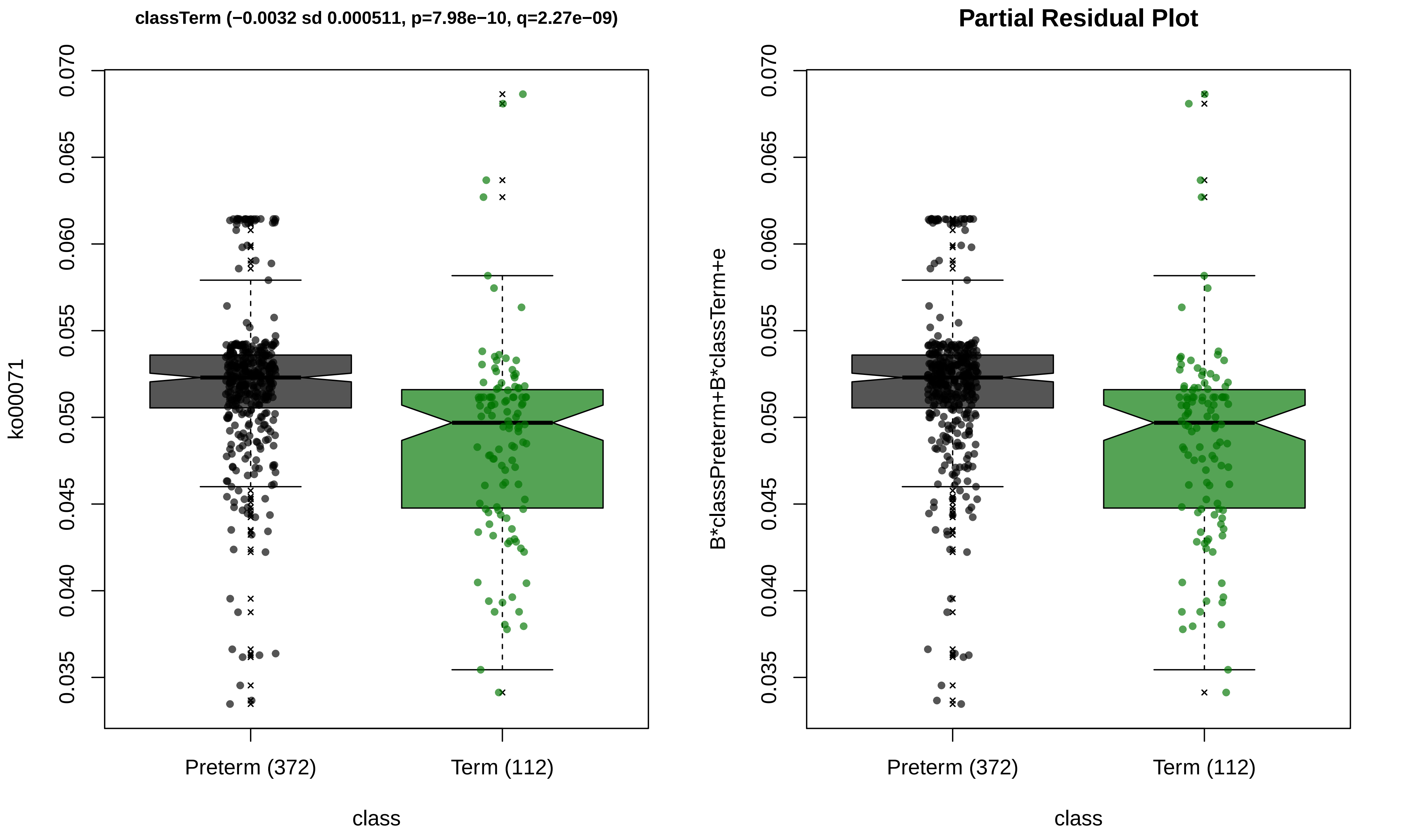

classTerm (−0.0032 sd 0.000511, p=7.98e−10, q=2.27e−09)
Partial Residual Plot
ko00071
B*classPreterm+B*classTerm+e
0.035
0.040
0.045
0.050
0.055
0.060
0.065
0.070
Preterm (372)
Term (112)
class

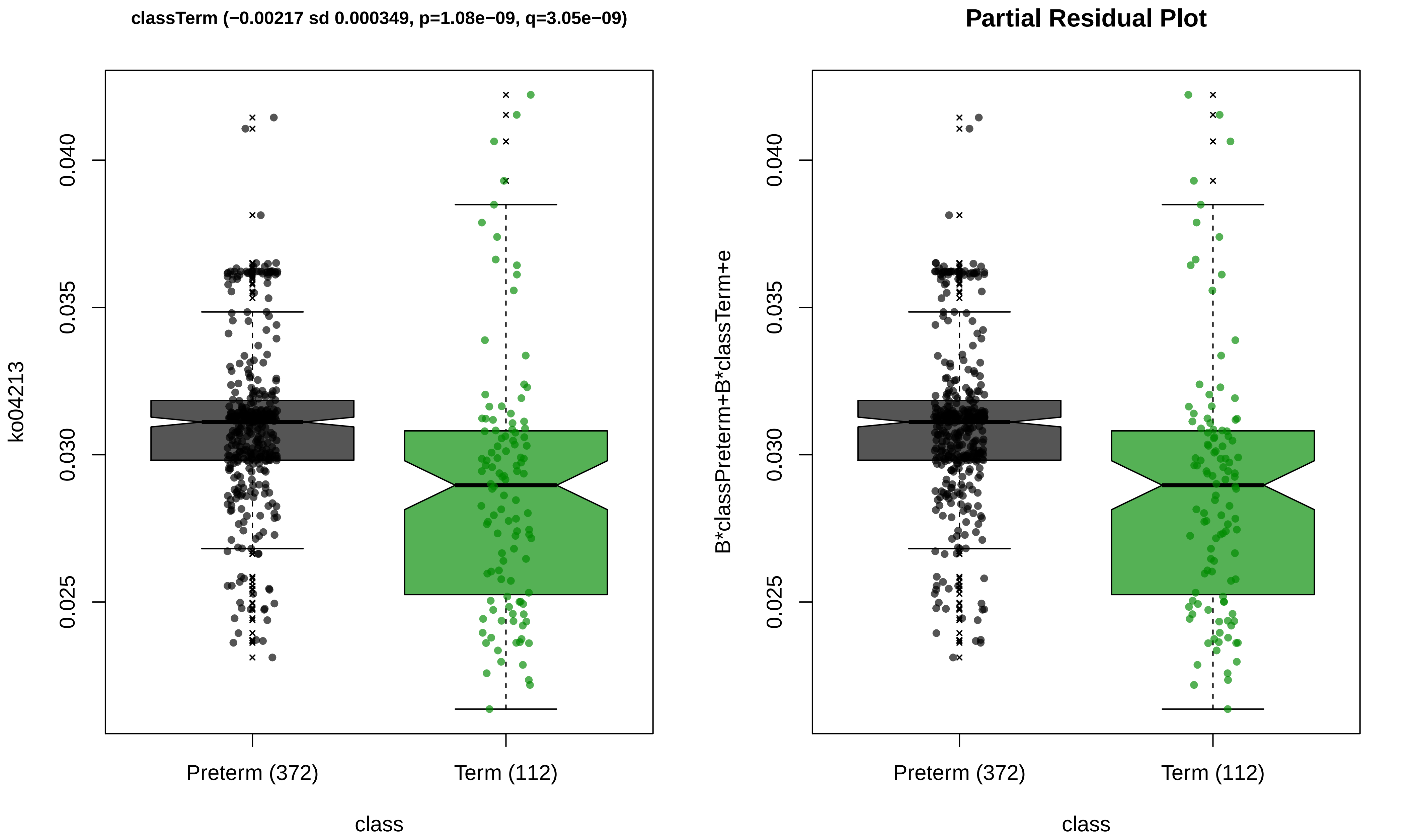
classTerm (−0.00217 sd 0.000349, p=1.08e−09, q=3.05e−09)
Partial Residual Plot
ko04213
B*classPreterm+B*classTerm+e
0.025
0.030
0.035
0.040
Preterm (372)
Term (112)
class

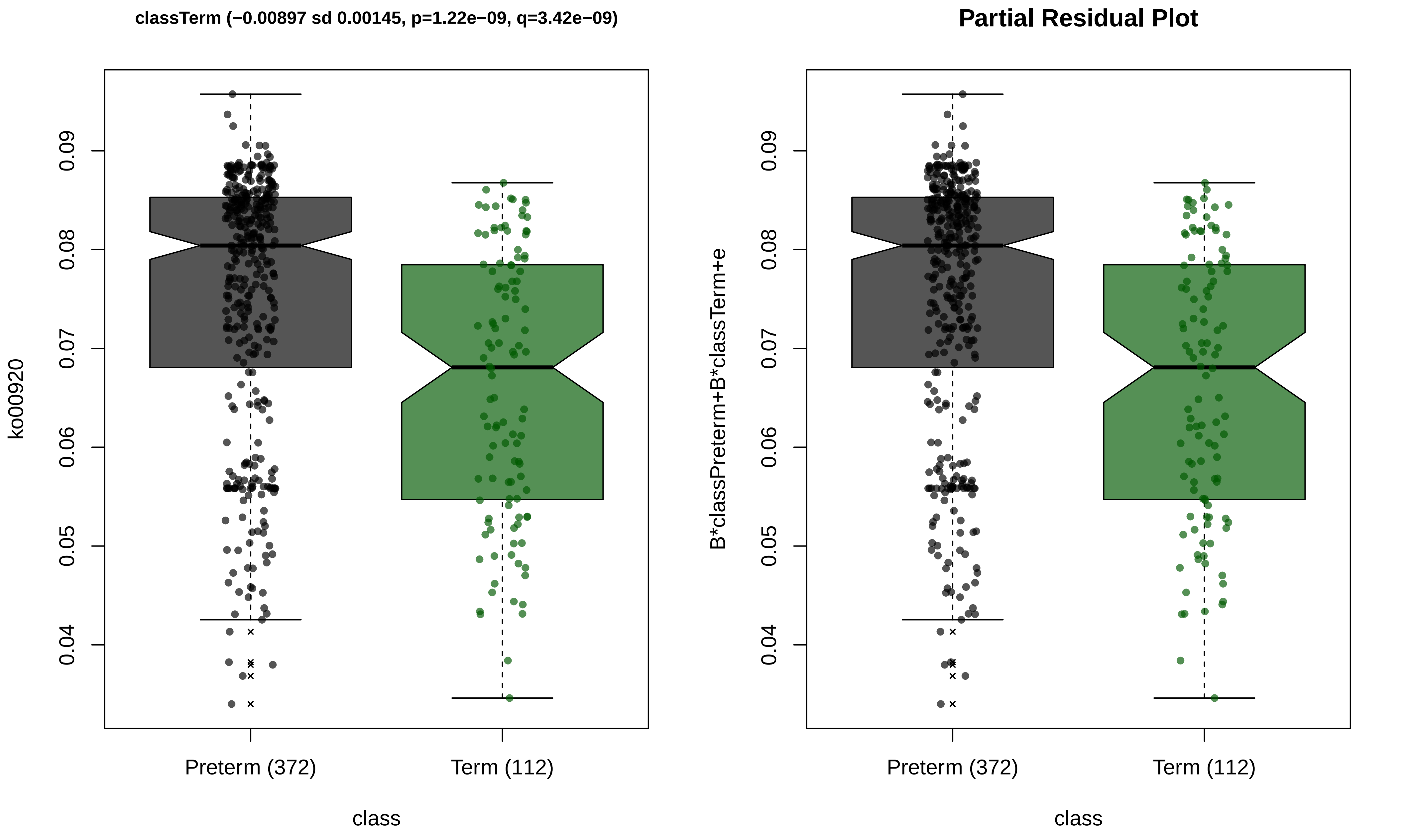

classTerm (−0.00897 sd 0.00145, p=1.22e−09, q=3.42e−09)
Partial Residual Plot
ko00920
B*classPreterm+B*classTerm+e
0.04
0.05
0.06
0.07
0.08
0.09
Preterm (372)
Term (112)
class

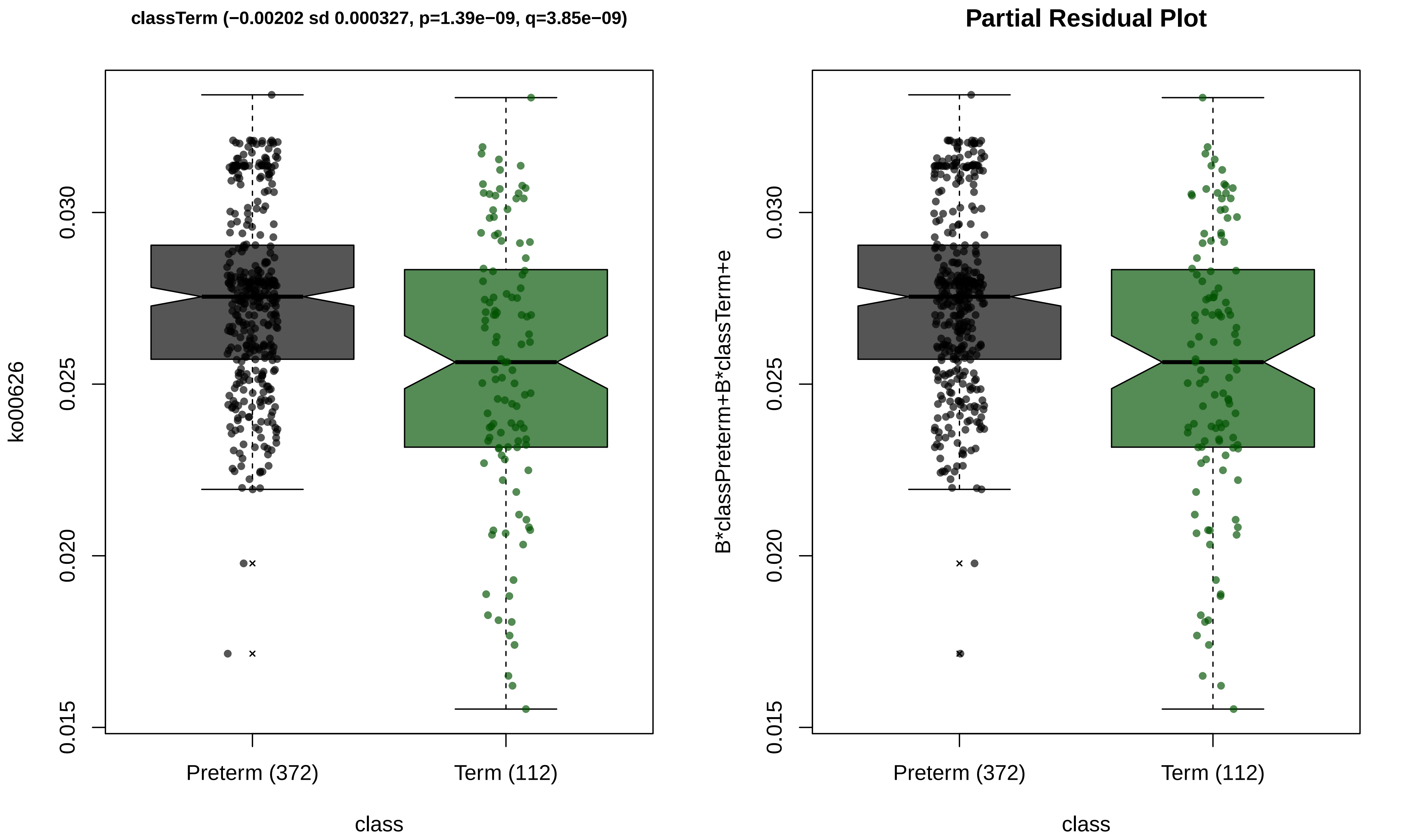

classTerm (−0.00202 sd 0.000327, p=1.39e−09, q=3.85e−09)
Partial Residual Plot
ko00626
B*classPreterm+B*classTerm+e
0.015
0.020
0.025
0.030
Preterm (372)
Term (112)
class

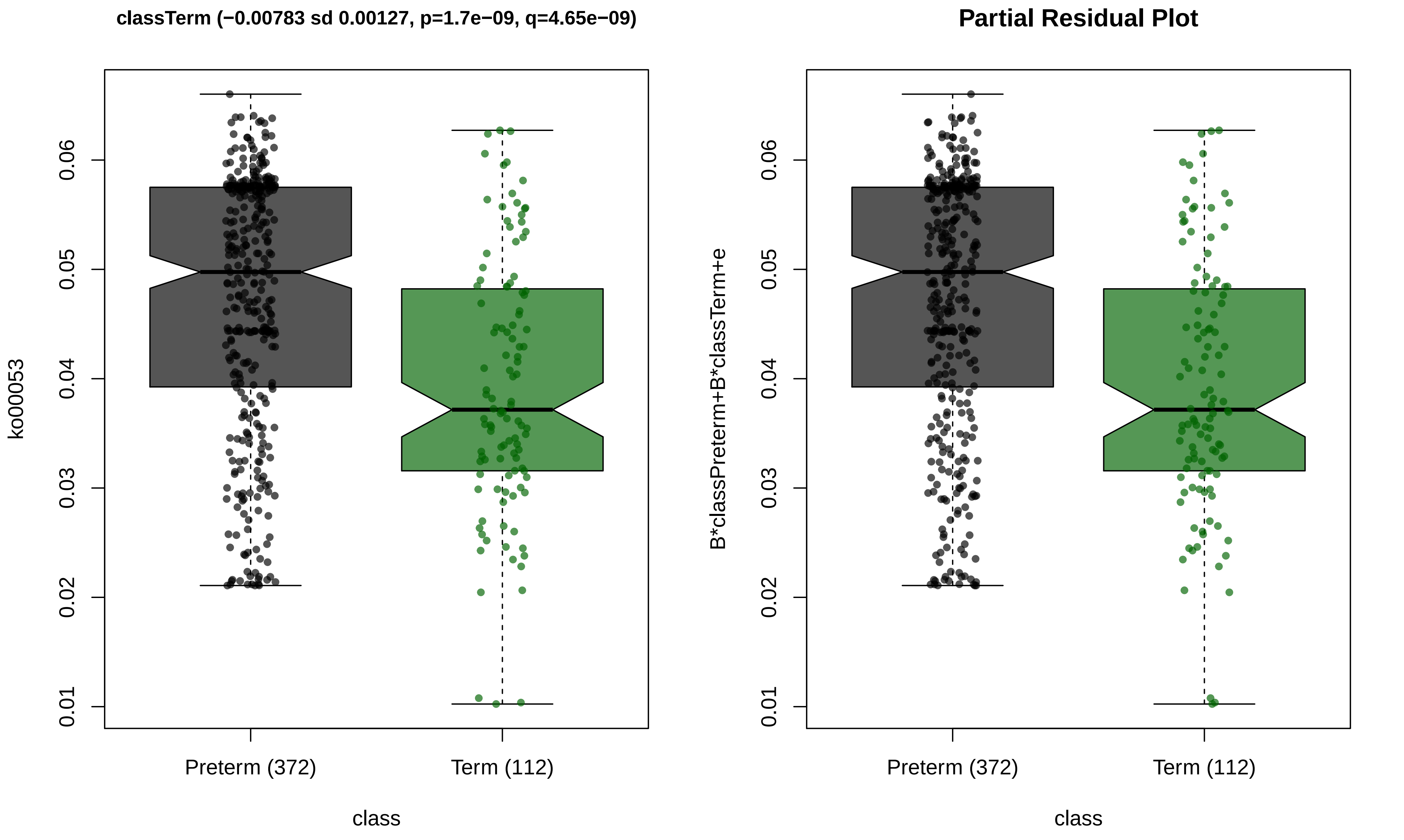

classTerm (−0.00783 sd 0.00127, p=1.7e−09, q=4.65e−09)
Partial Residual Plot
ko00053
B*classPreterm+B*classTerm+e
0.01
0.02
0.03
0.04
0.05
0.06
Preterm (372)
Term (112)
class

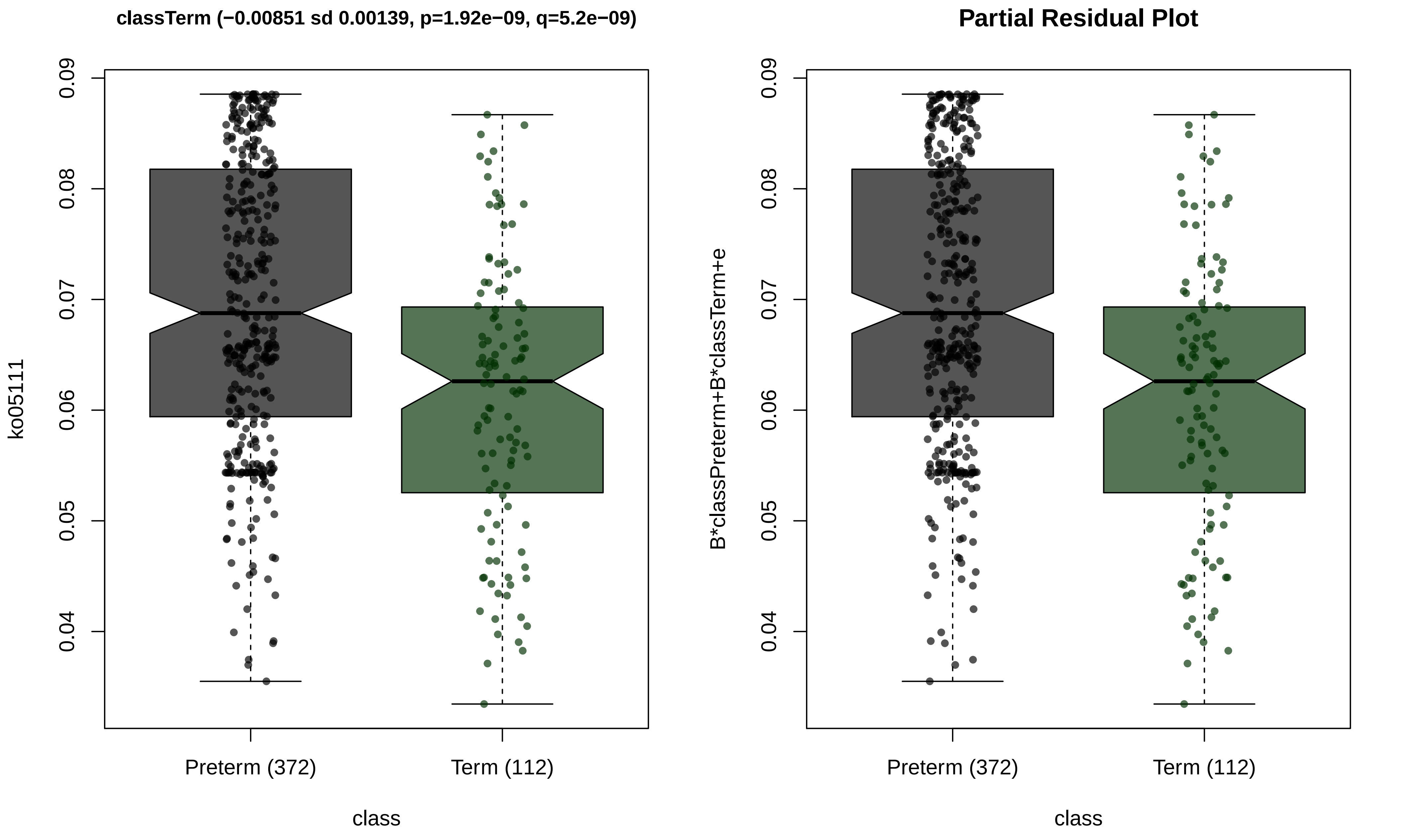

classTerm (−0.00851 sd 0.00139, p=1.92e−09, q=5.2e−09)
Partial Residual Plot
ko05111
B*classPreterm+B*classTerm+e
0.09
0.08
0.07
0.06
0.05
0.04
Preterm (372)
Term (112)
class

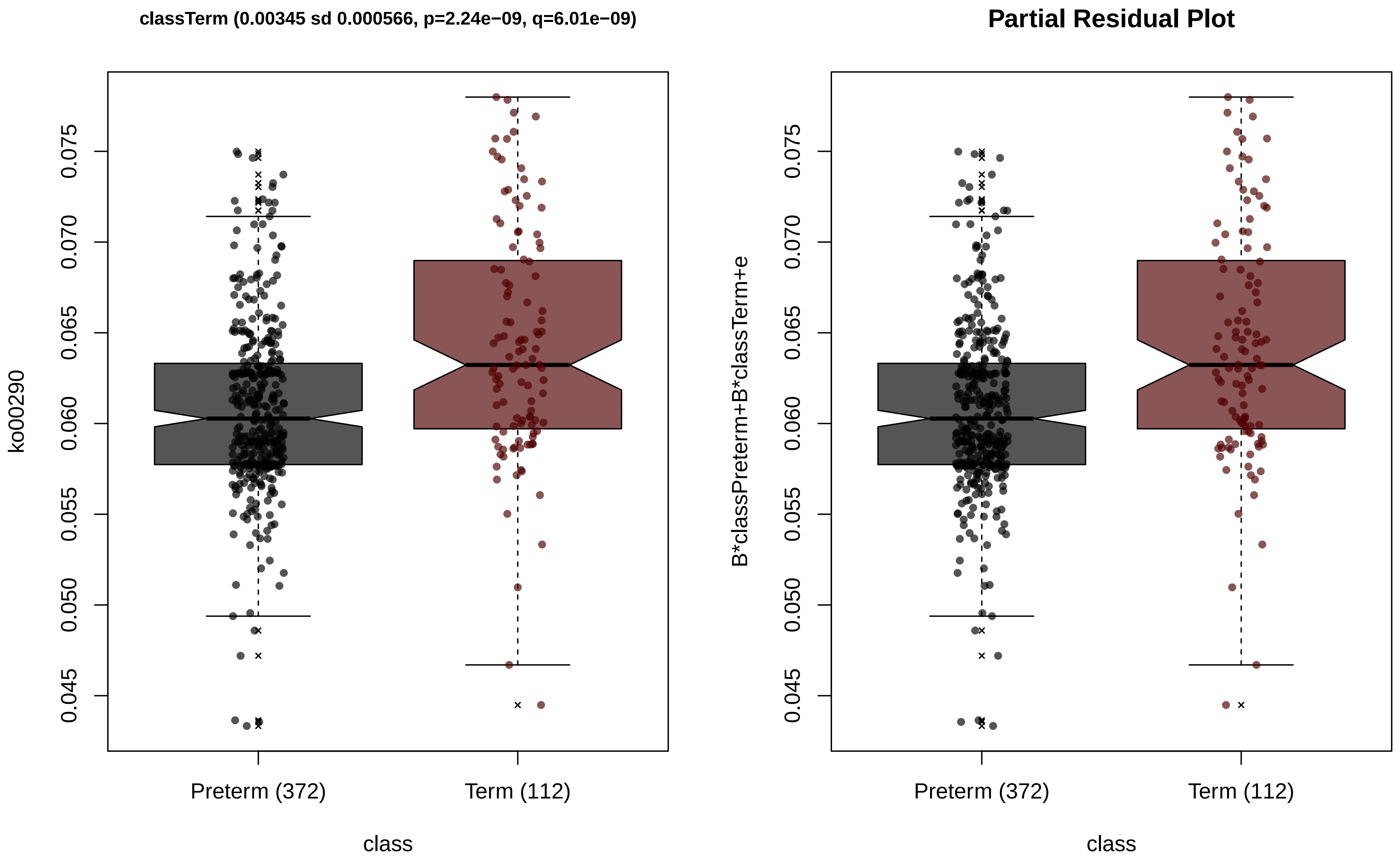
classTerm (0.00345 sd 0.000566, p=2.24e−09, q=6.01e−09)
ko00290
0.045
0.050
0.055
0.060
0.065
0.070
0.075
Preterm (372)
Term (112)
class
Partial Residual Plot
B*classPreterm+B*classTerm+e
0.045
0.050
0.055
0.060
0.065
0.070
0.075
Preterm (372)
Term (112)
class

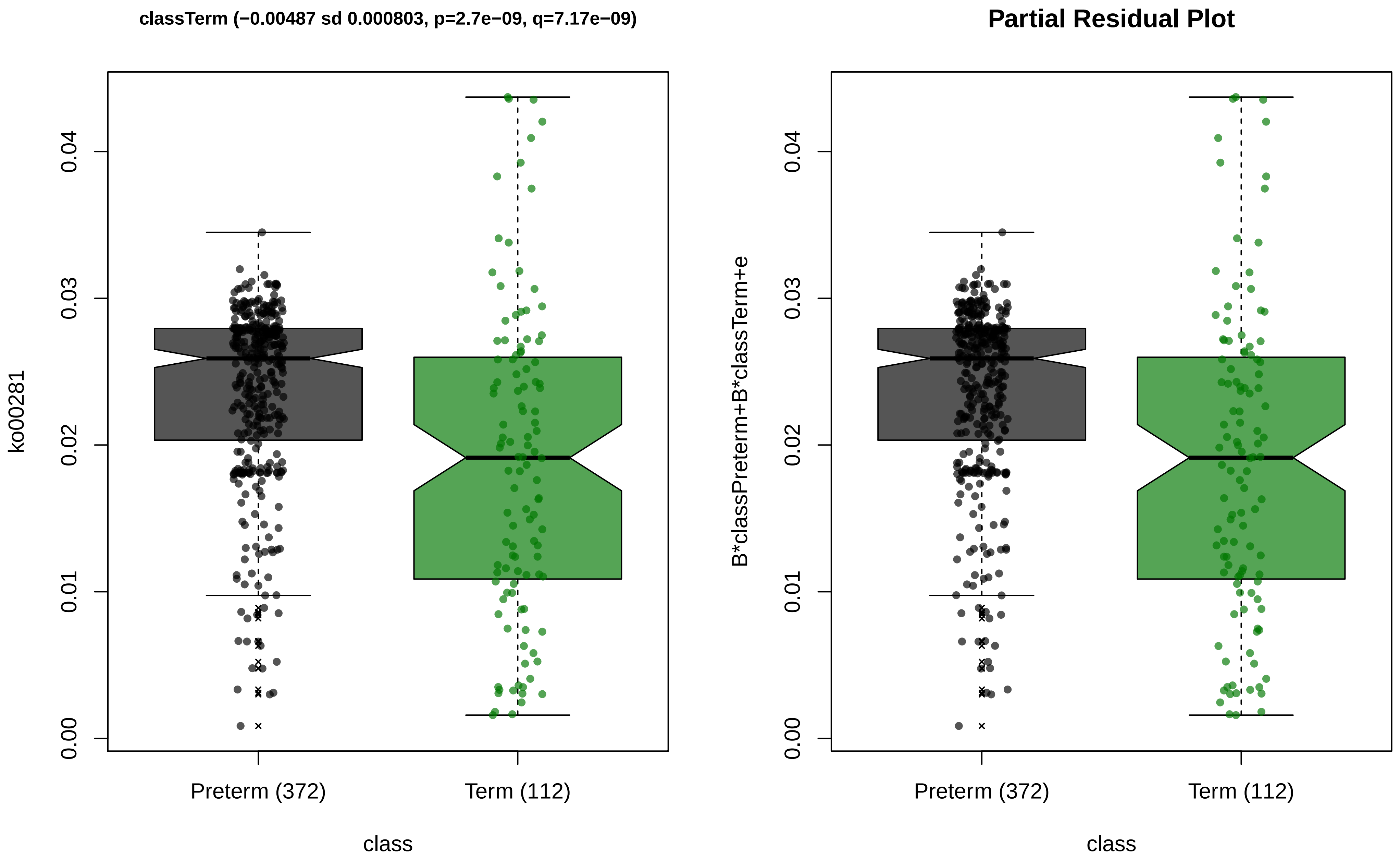

classTerm (−0.00487 sd 0.000803, p=2.7e−09, q=7.17e−09)
Partial Residual Plot
ko00281
B*classPreterm+B*classTerm+e
0.00
0.01
0.02
0.03
0.04
Preterm (372)
Term (112)
class

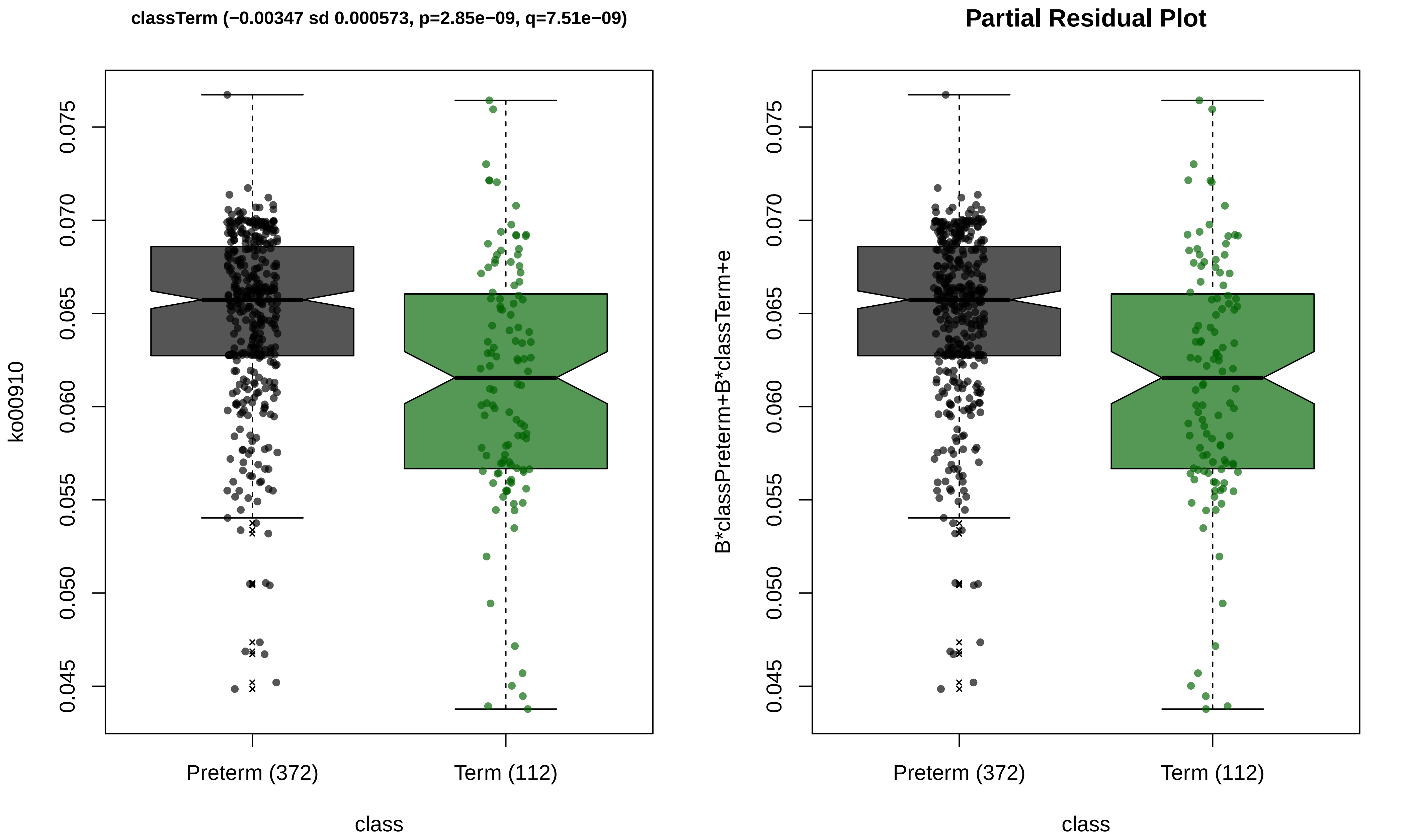

classTerm (−0.00347 sd 0.000573, p=2.85e−09, q=7.51e−09)
Partial Residual Plot
ko00910
B*classPreterm+B*classTerm+e
0.045
0.050
0.055
0.060
0.065
0.070
0.075
Preterm (372)
Term (112)
class

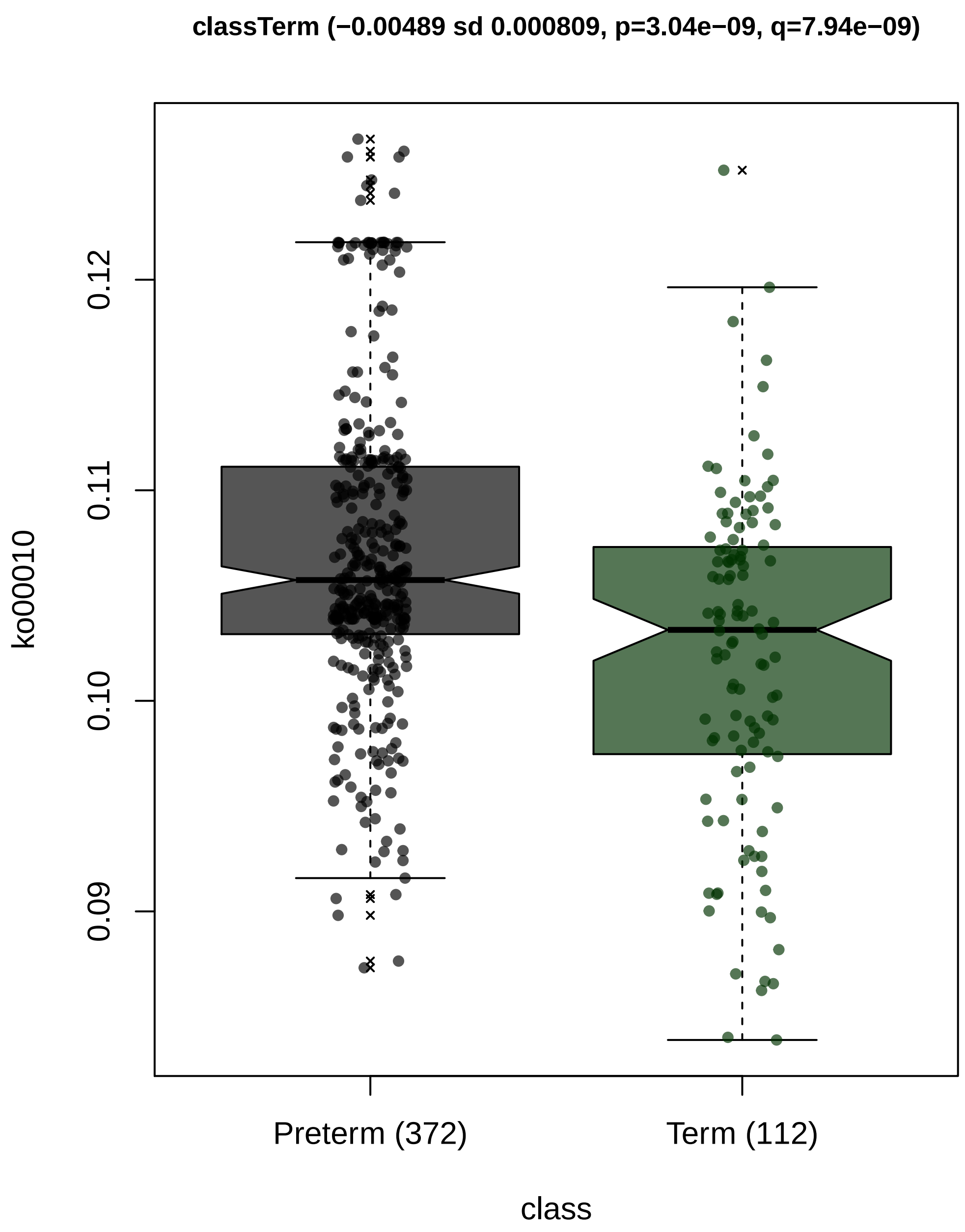
classTerm (−0.00489 sd 0.000809, p=3.04e−09, q=7.94e−09)
ko00010
0.09
0.10
0.11
0.12
Preterm (372)
Term (112)
class

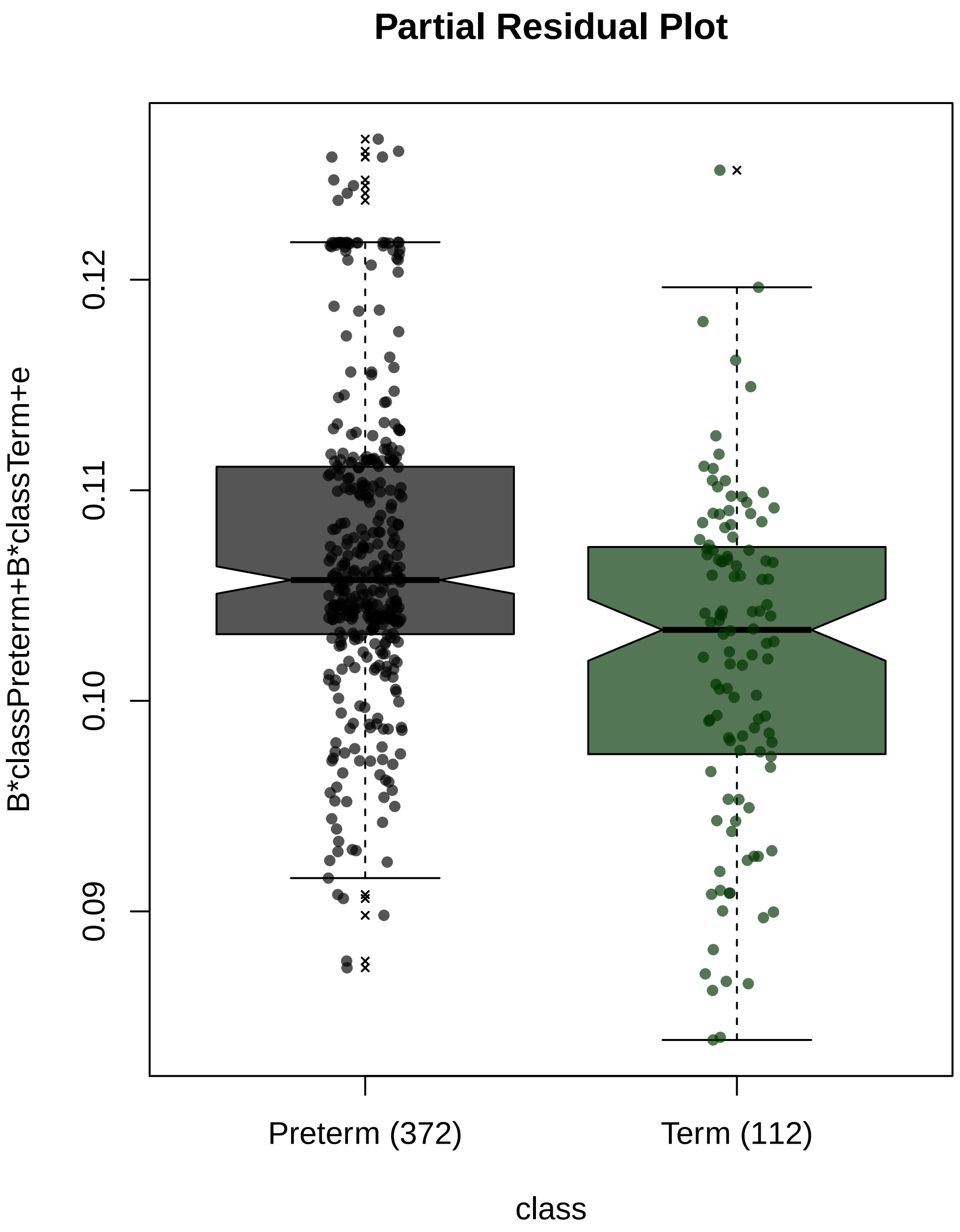
Partial Residual Plot
B*classPreterm+B*classTerm+e
0.09
0.10
0.11
0.12
Preterm (372)
Term (112)
class

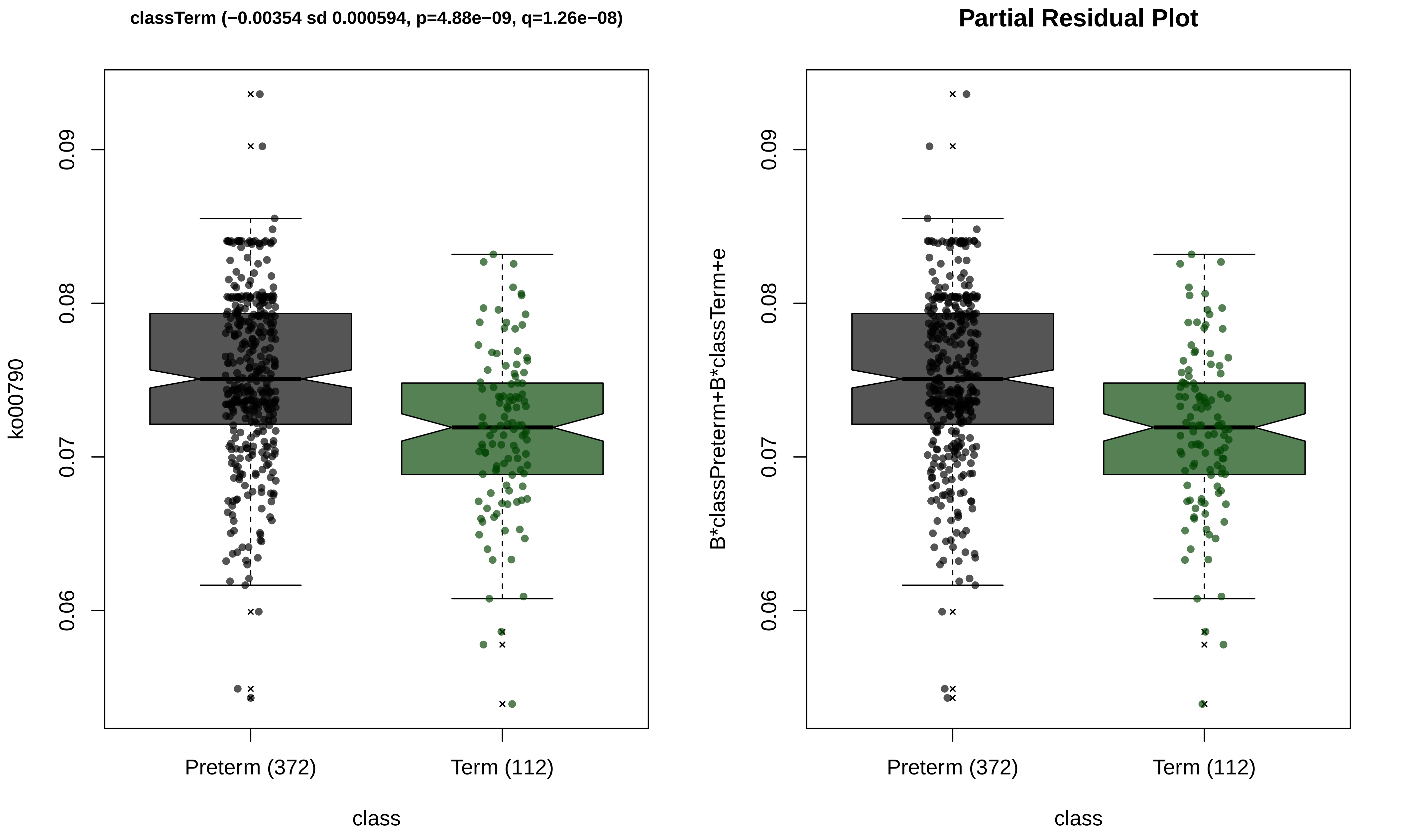
classTerm (−0.00354 sd 0.000594, p=4.88e−09, q=1.26e−08)
Partial Residual Plot
ko00790
B*classPreterm+B*classTerm+e
0.06
0.07
0.08
0.09
Preterm (372)
Term (112)
class

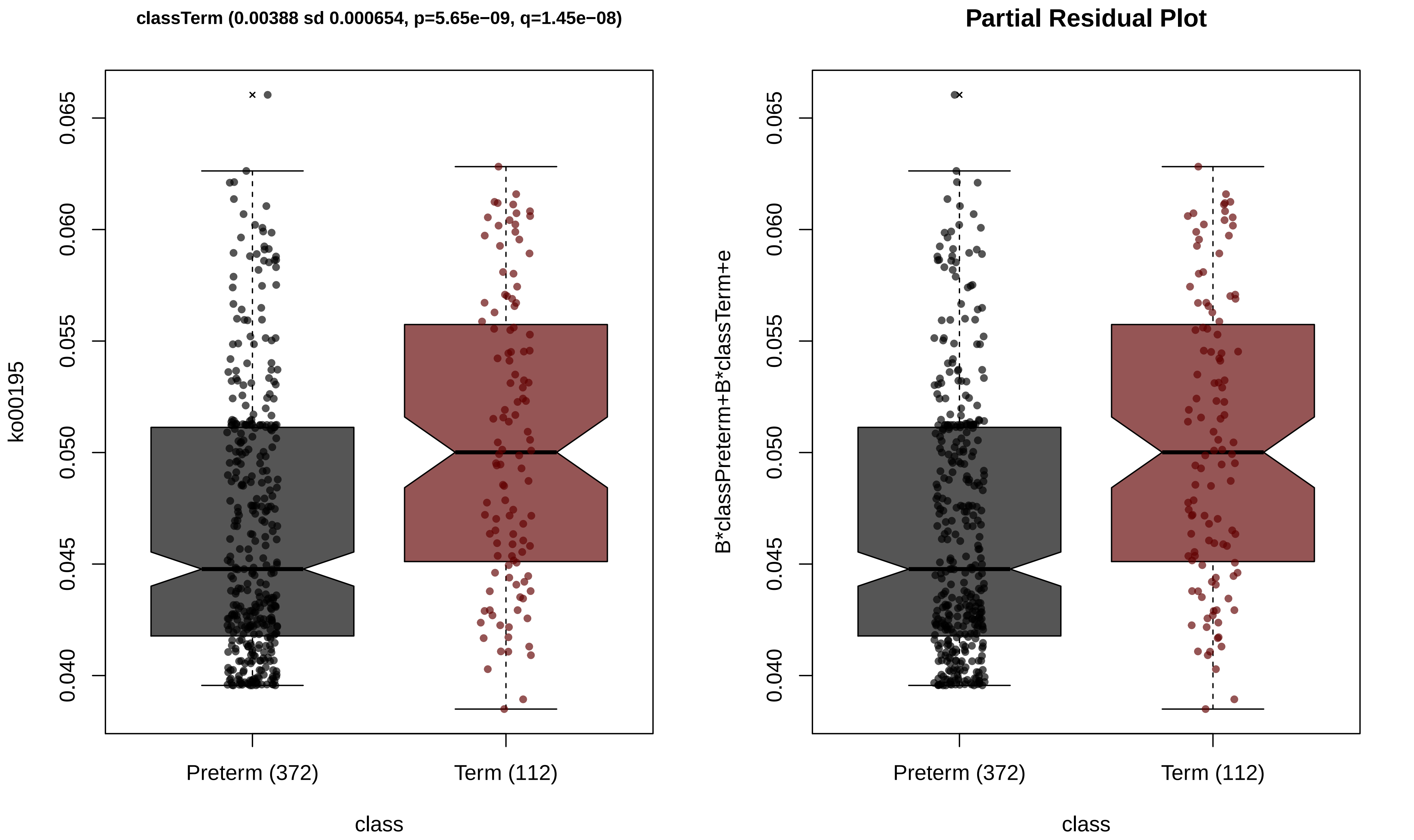

classTerm (0.00388 sd 0.000654, p=5.65e−09, q=1.45e−08)
ko00195
0.040
0.045
0.050
0.055
0.060
0.065
Preterm (372)
Term (112)
class
Partial Residual Plot
B*classPreterm+B*classTerm+e
0.040
0.045
0.050
0.055
0.060
0.065
Preterm (372)
Term (112)
class

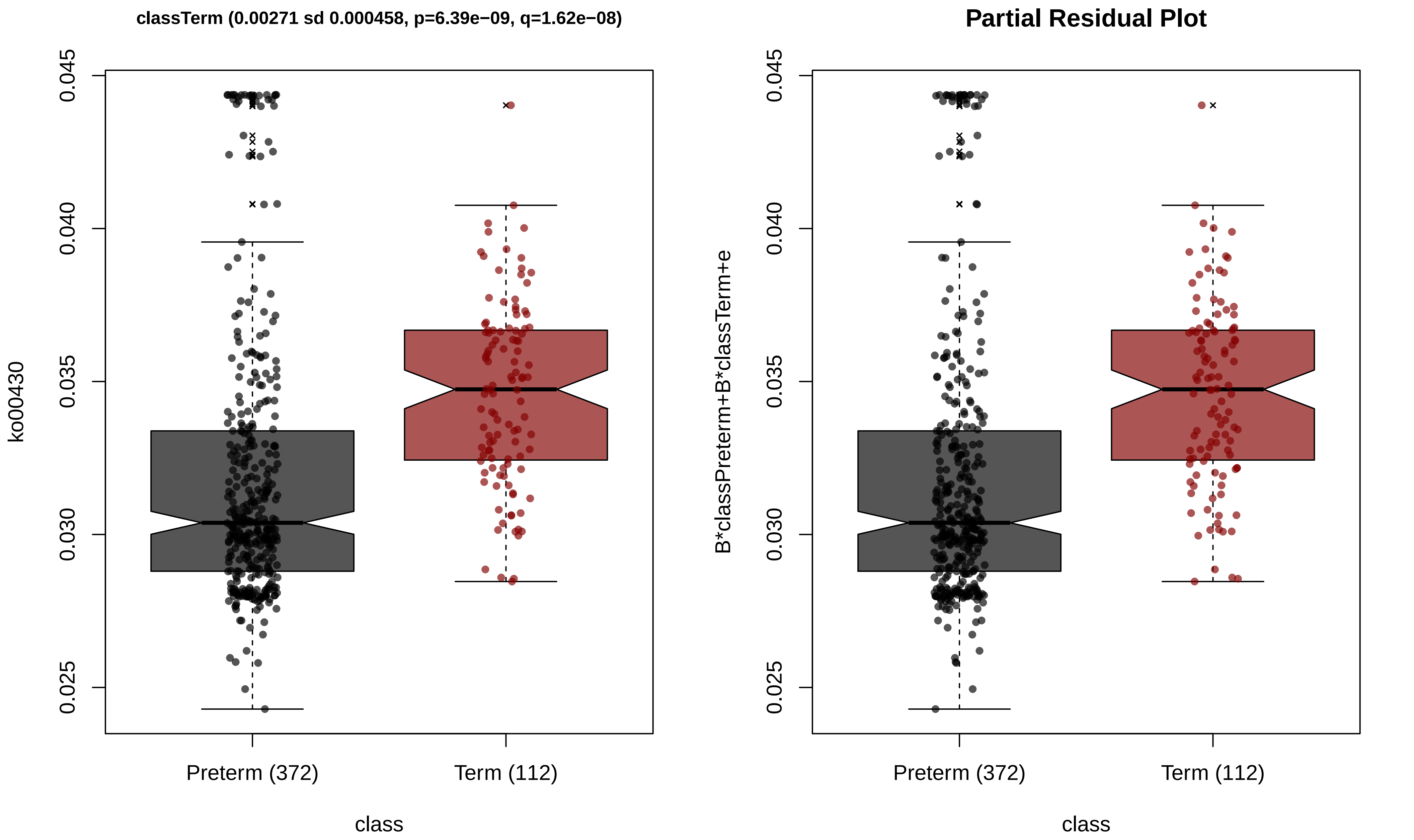
classTerm (0.00271 sd 0.000458, p=6.39e−09, q=1.62e−08)
Partial Residual Plot
ko00430
B*classPreterm+B*classTerm+e
0.025
0.030
0.035
0.040
0.045
Preterm (372)
Term (112)
class

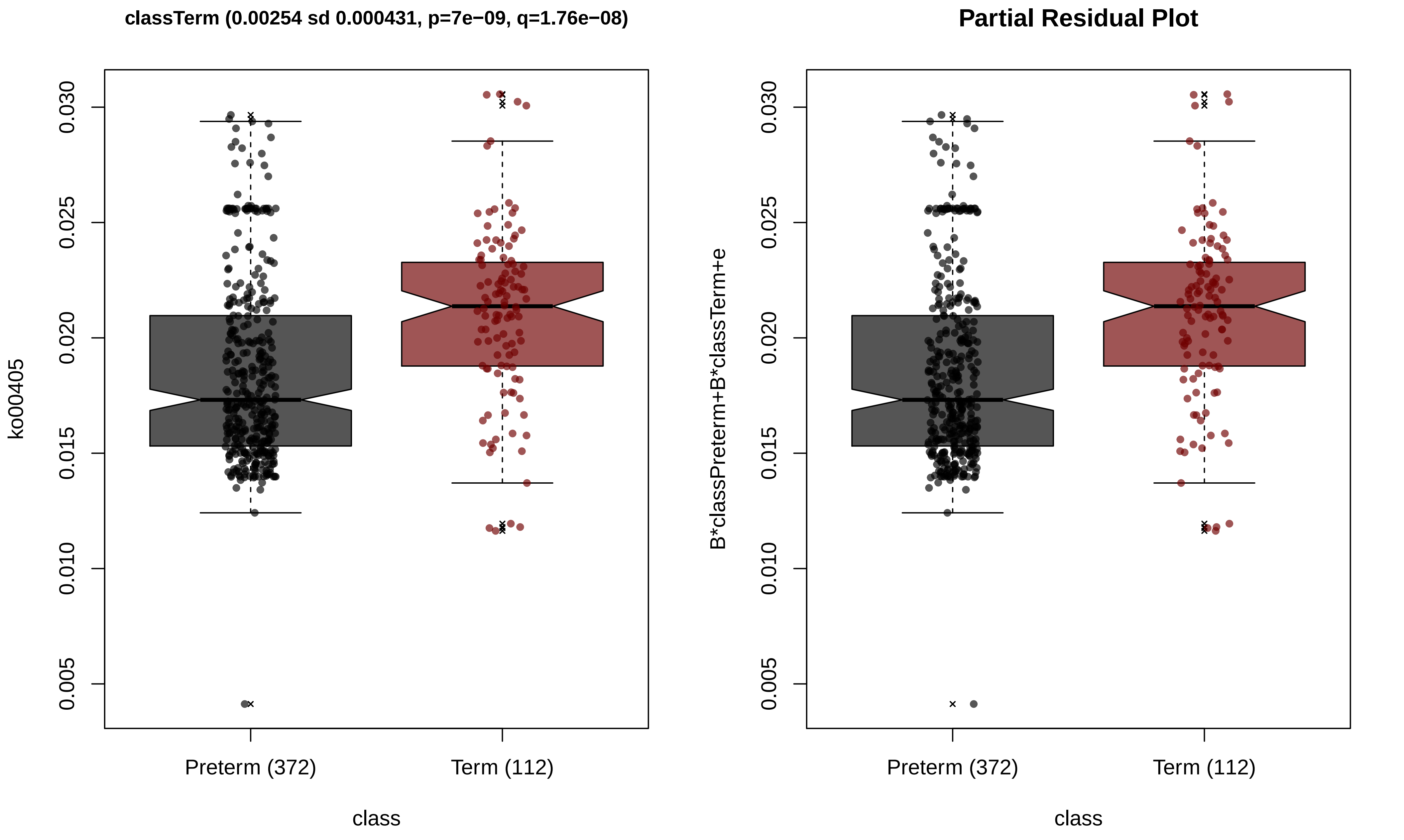

classTerm (0.00254 sd 0.000431, p=7e−09, q=1.76e−08)
Partial Residual Plot
ko00405
B*classPreterm+B*classTerm+e
0.005
0.010
0.015
0.020
0.025
0.030
Preterm (372)
Term (112)
class

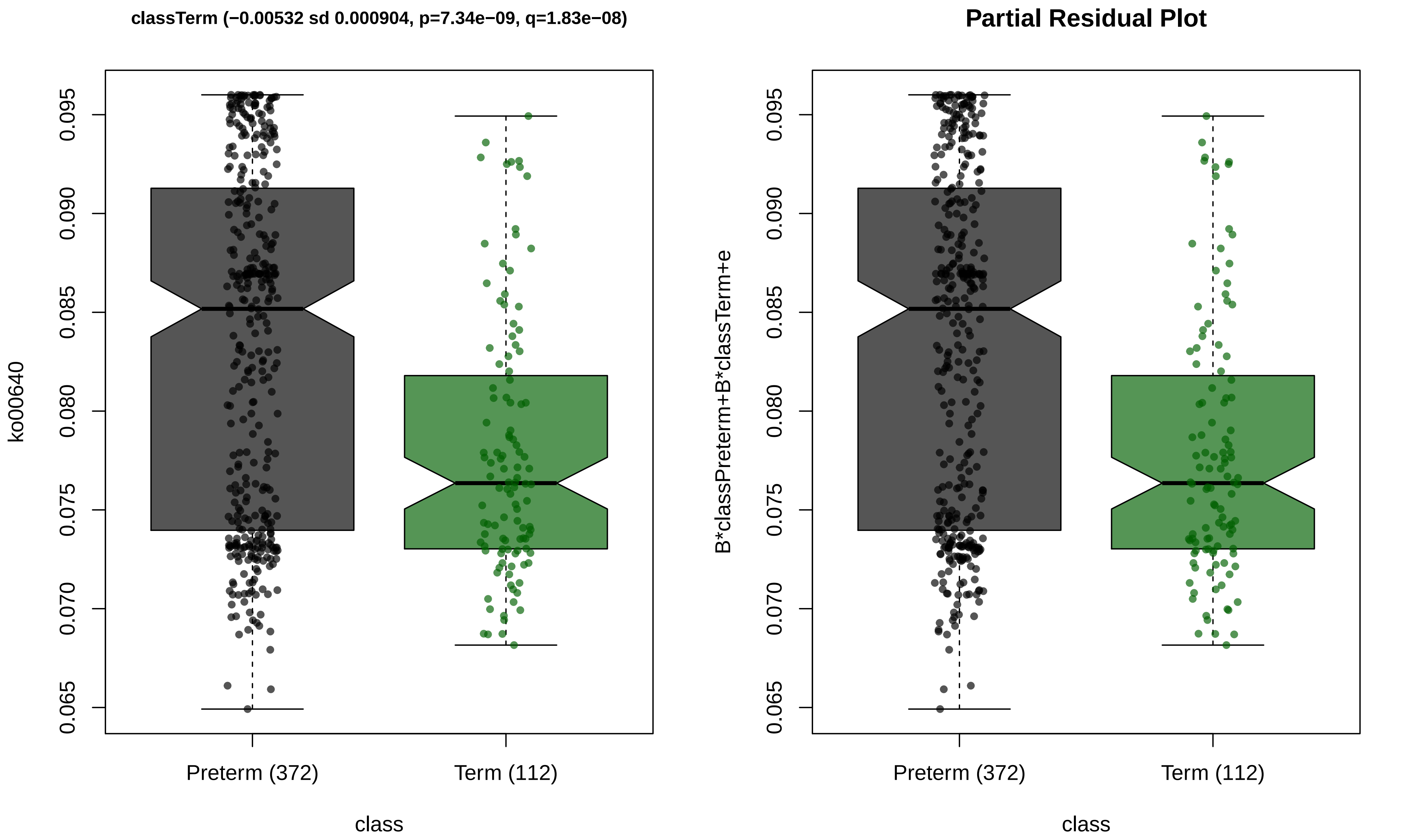

classTerm (−0.00532 sd 0.000904, p=7.34e−09, q=1.83e−08)
Partial Residual Plot
ko00640
B*classPreterm+B*classTerm+e
0.065
0.070
0.075
0.080
0.085
0.090
0.095
Preterm (372)
Term (112)
class

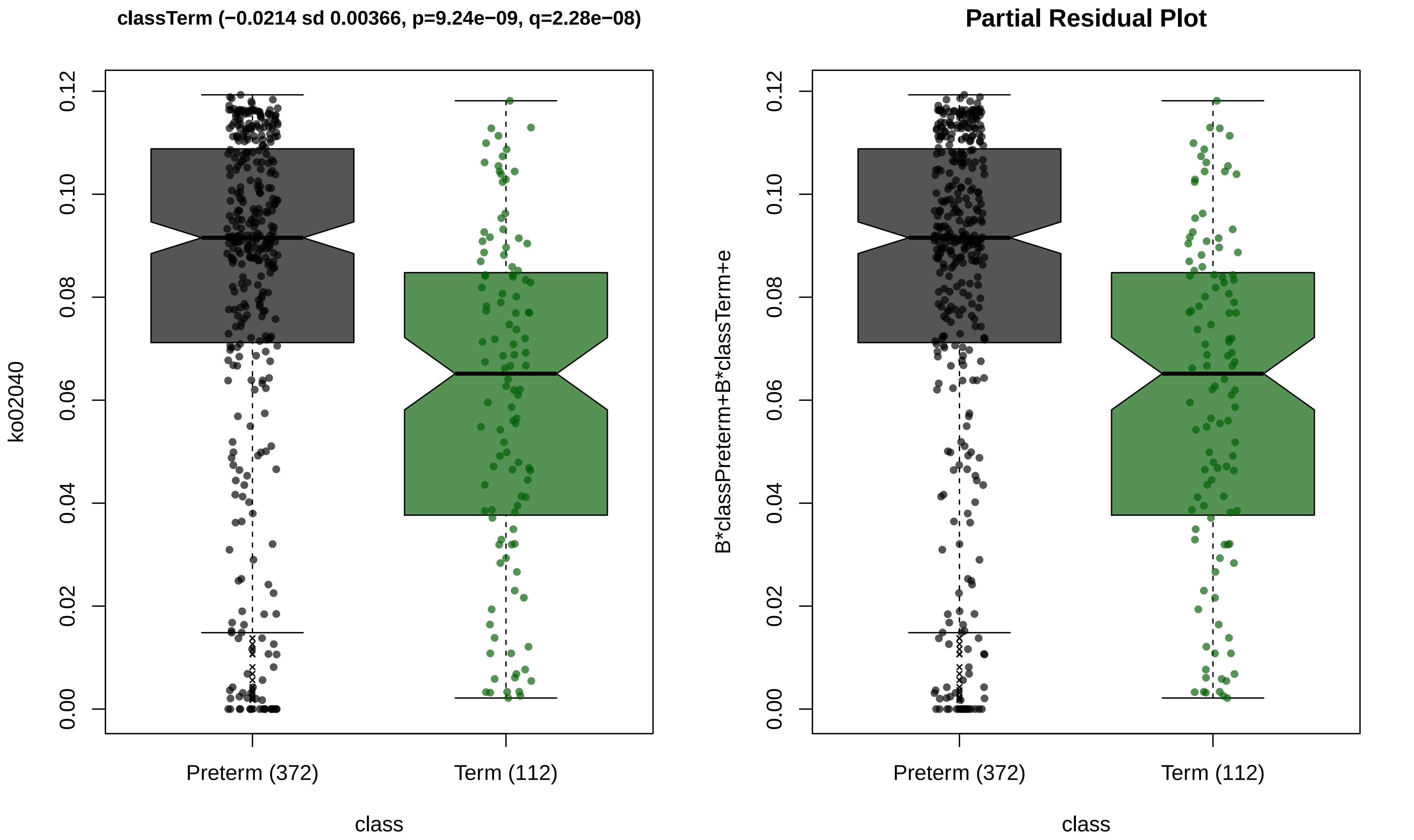
classTerm (−0.0214 sd 0.00366, p=9.24e−09, q=2.28e−08)
Partial Residual Plot
ko02040
B*classPreterm+B*classTerm+e
0.00
0.02
0.04
0.06
0.08
0.10
0.12
Preterm (372)
Term (112)
class

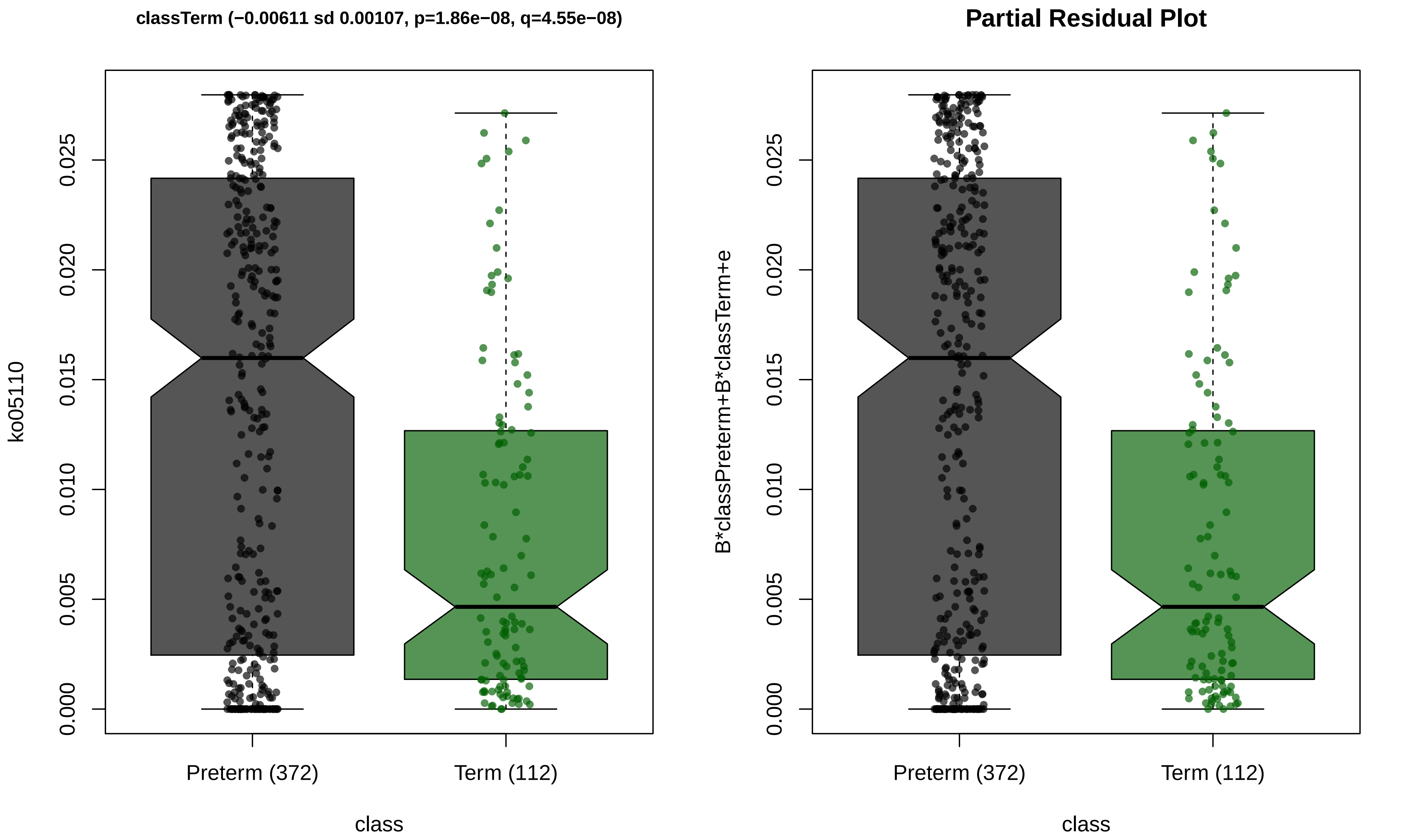

classTerm (−0.00611 sd 0.00107, p=1.86e−08, q=4.55e−08)
Partial Residual Plot
ko05110
B*classPreterm+B*classTerm+e
0.000
0.005
0.010
0.015
0.020
0.025
Preterm (372)
Term (112)
class

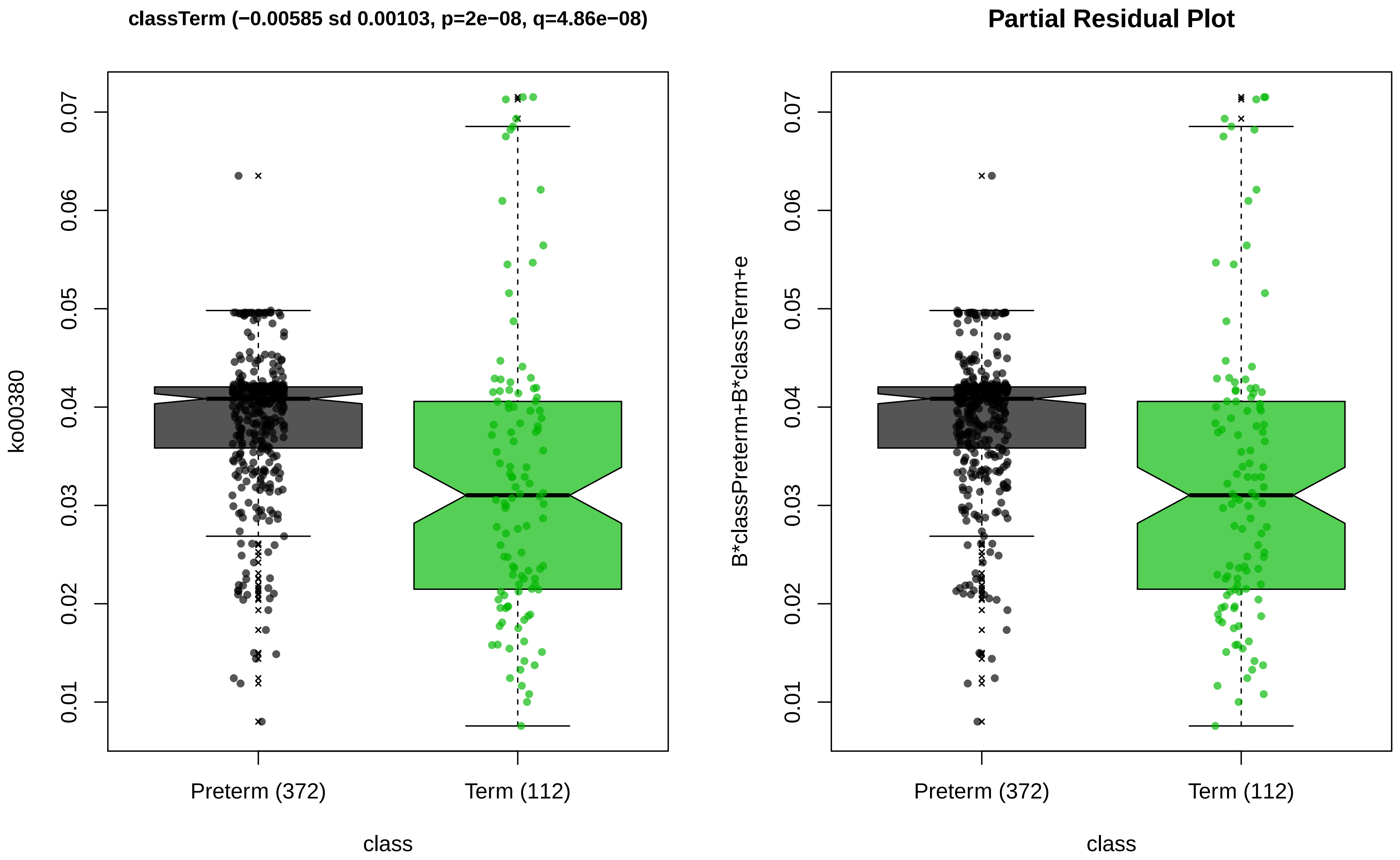

classTerm (−0.00585 sd 0.00103, p=2e−08, q=4.86e−08)
ko00380
0.01
0.02
0.03
0.04
0.05
0.06
0.07
Preterm (372)
Term (112)
class
Partial Residual Plot
B*classPreterm+B*classTerm+e
0.01
0.02
0.03
0.04
0.05
0.06
0.07
Preterm (372)
Term (112)
class

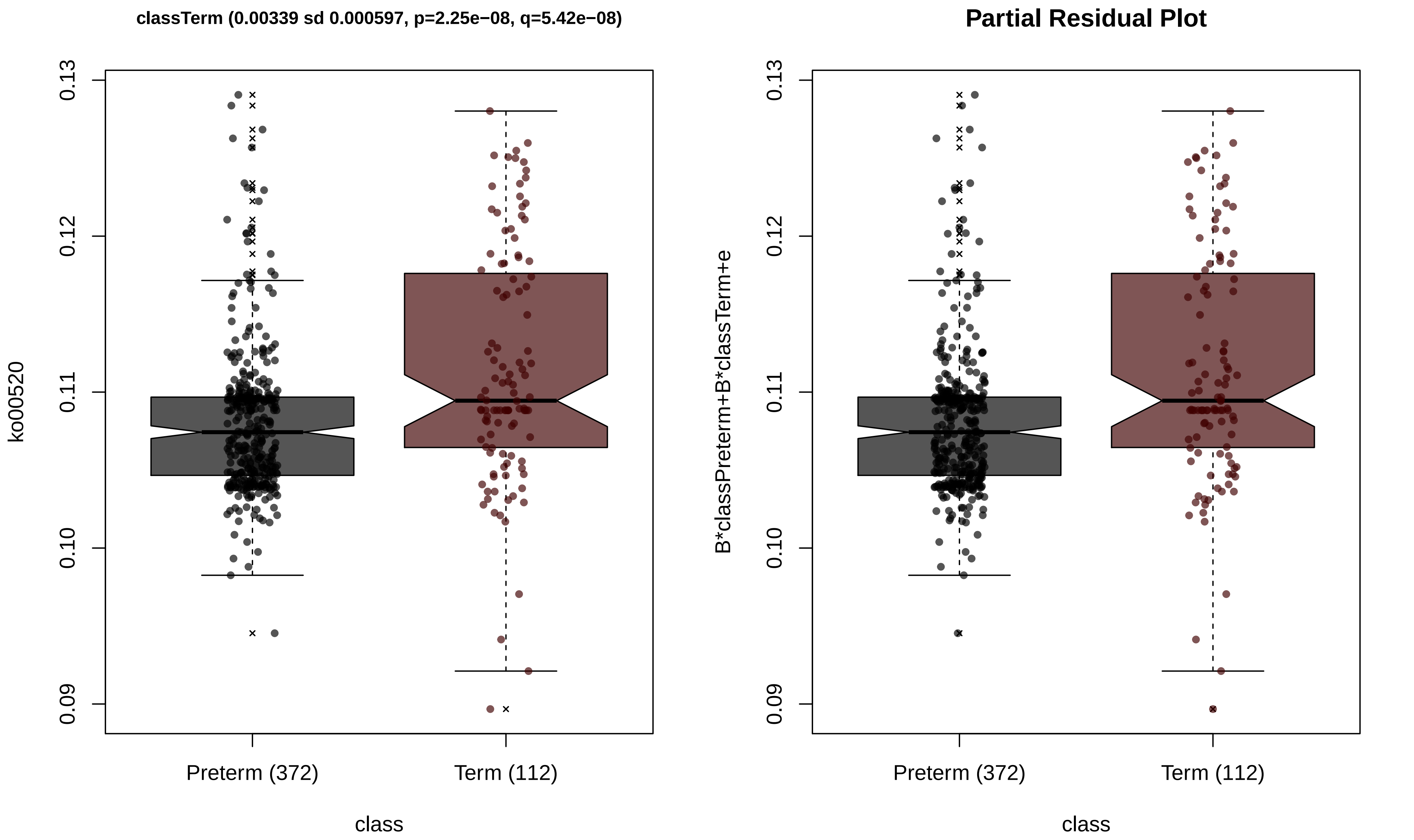
classTerm (0.00339 sd 0.000597, p=2.25e−08, q=5.42e−08)
Partial Residual Plot
ko00520
B*classPreterm+B*classTerm+e
0.13
0.12
0.11
0.10
0.09
Preterm (372)
Term (112)
class

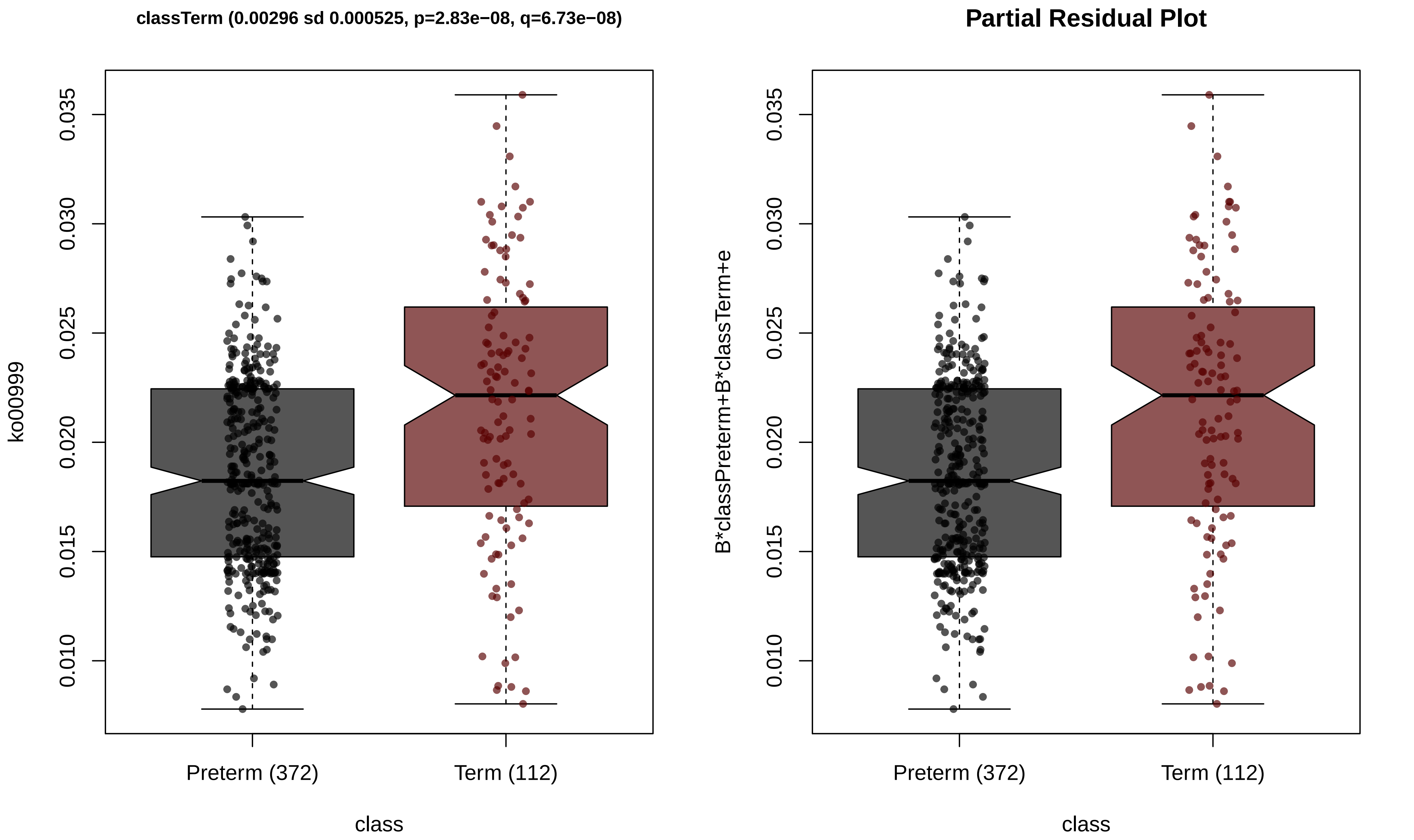
classTerm (0.00296 sd 0.000525, p=2.83e−08, q=6.73e−08)
ko00999
0.010
0.015
0.020
0.025
0.030
0.035
Preterm (372)
Term (112)
class
Partial Residual Plot
B*classPreterm+B*classTerm+e
0.010
0.015
0.020
0.025
0.030
0.035
Preterm (372)
Term (112)
class

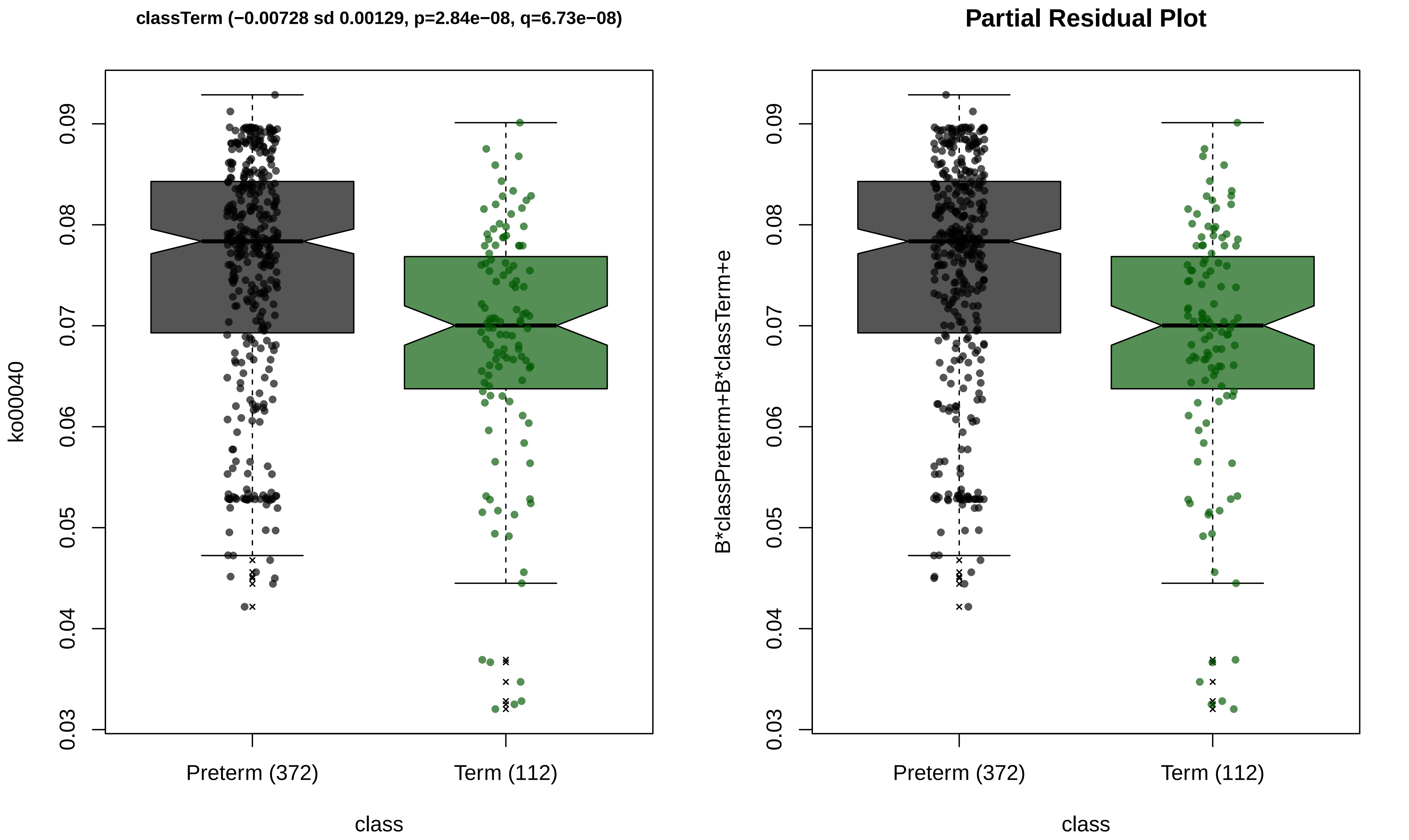

classTerm (−0.00728 sd 0.00129, p=2.84e−08, q=6.73e−08)
ko00040
0.03
0.04
0.05
0.06
0.07
0.08
0.09
Preterm (372)
Term (112)
class
Partial Residual Plot
B*classPreterm+B*classTerm+e
0.03
0.04
0.05
0.06
0.07
0.08
0.09
Preterm (372)
Term (112)
class

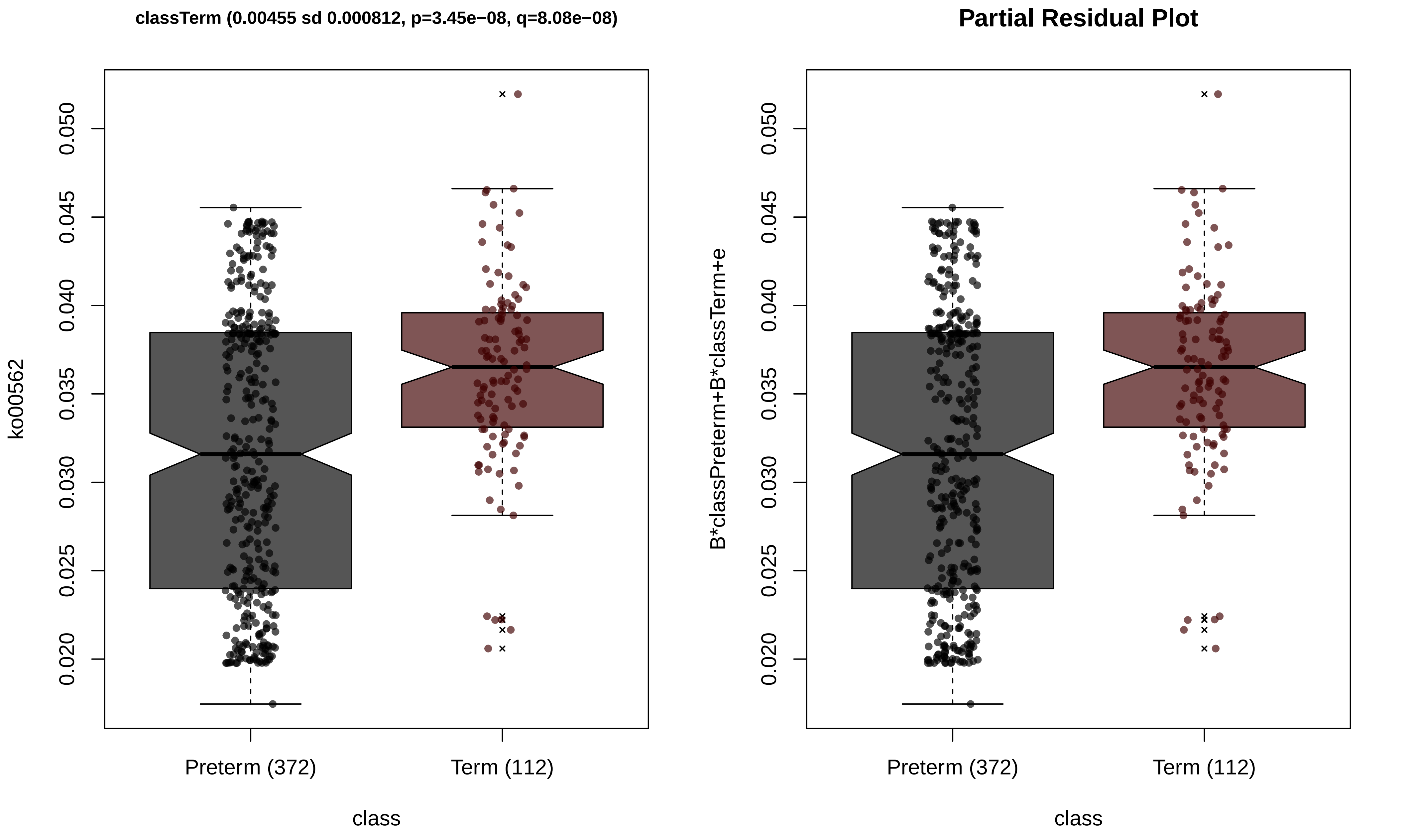
classTerm (0.00455 sd 0.000812, p=3.45e−08, q=8.08e−08)
Partial Residual Plot
ko00562
B*classPreterm+B*classTerm+e
0.020
0.025
0.030
0.035
0.040
0.045
0.050
Preterm (372)
Term (112)
class

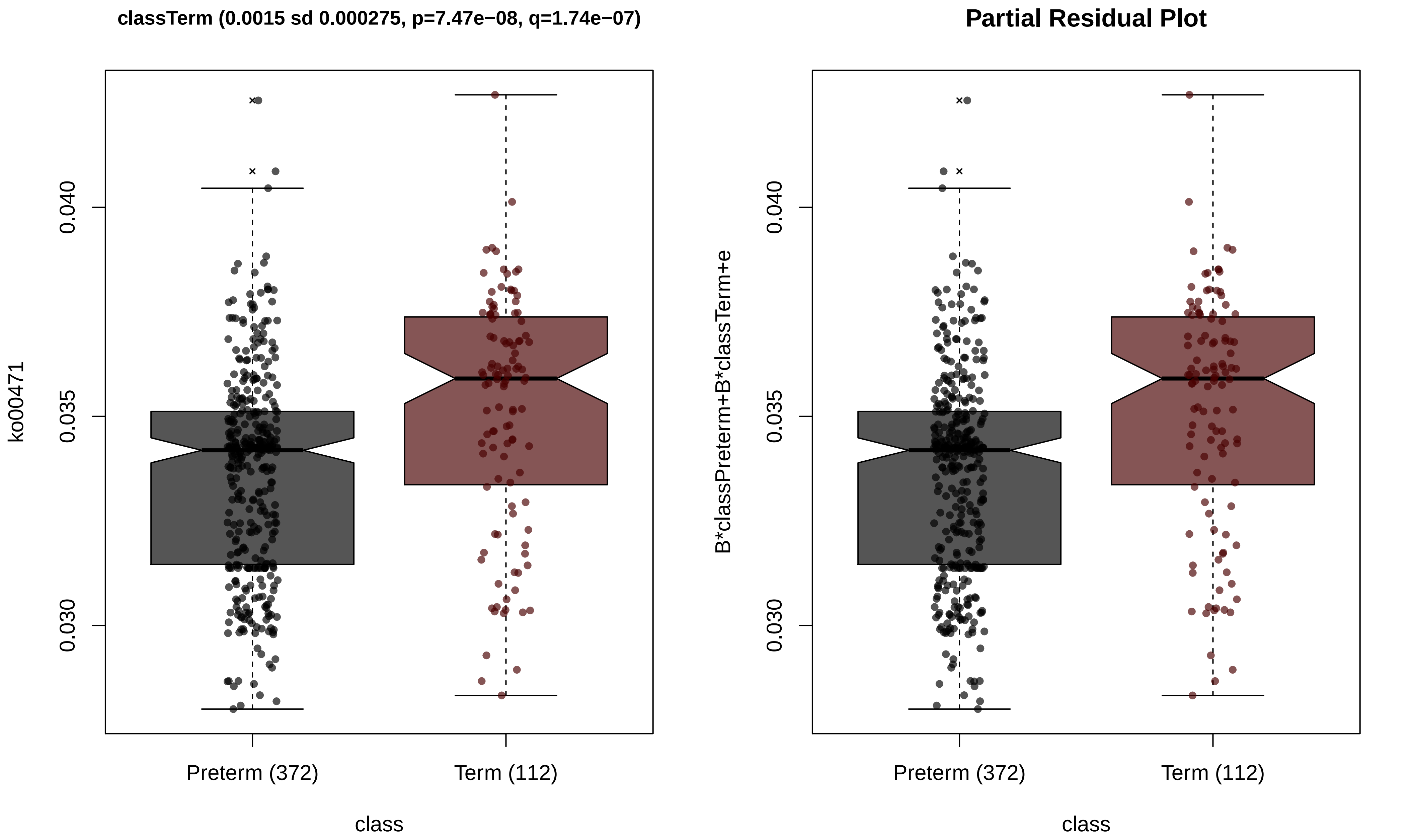

classTerm (0.0015 sd 0.000275, p=7.47e−08, q=1.74e−07)
ko00471
0.040
0.035
0.030
Preterm (372)
Term (112)
class
Partial Residual Plot
B*classPreterm+B*classTerm+e
0.040
0.035
0.030
Preterm (372)
Term (112)
class

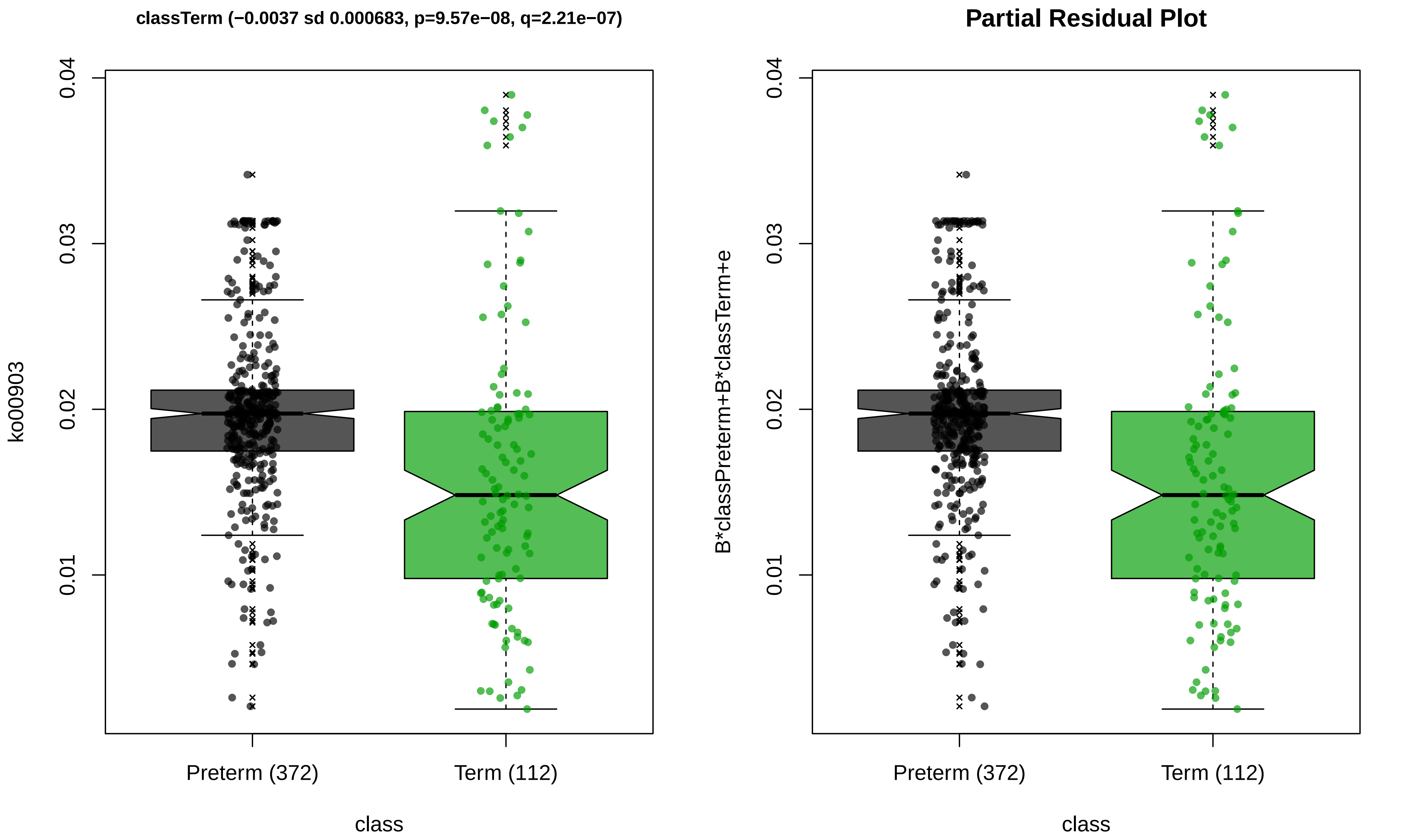
classTerm (−0.0037 sd 0.000683, p=9.57e−08, q=2.21e−07)
ko00903
0.04
0.03
0.02
0.01
Preterm (372)
Term (112)
class
Partial Residual Plot
B*classPreterm+B*classTerm+e
0.04
0.03
0.02
0.01
Preterm (372)
Term (112)
class

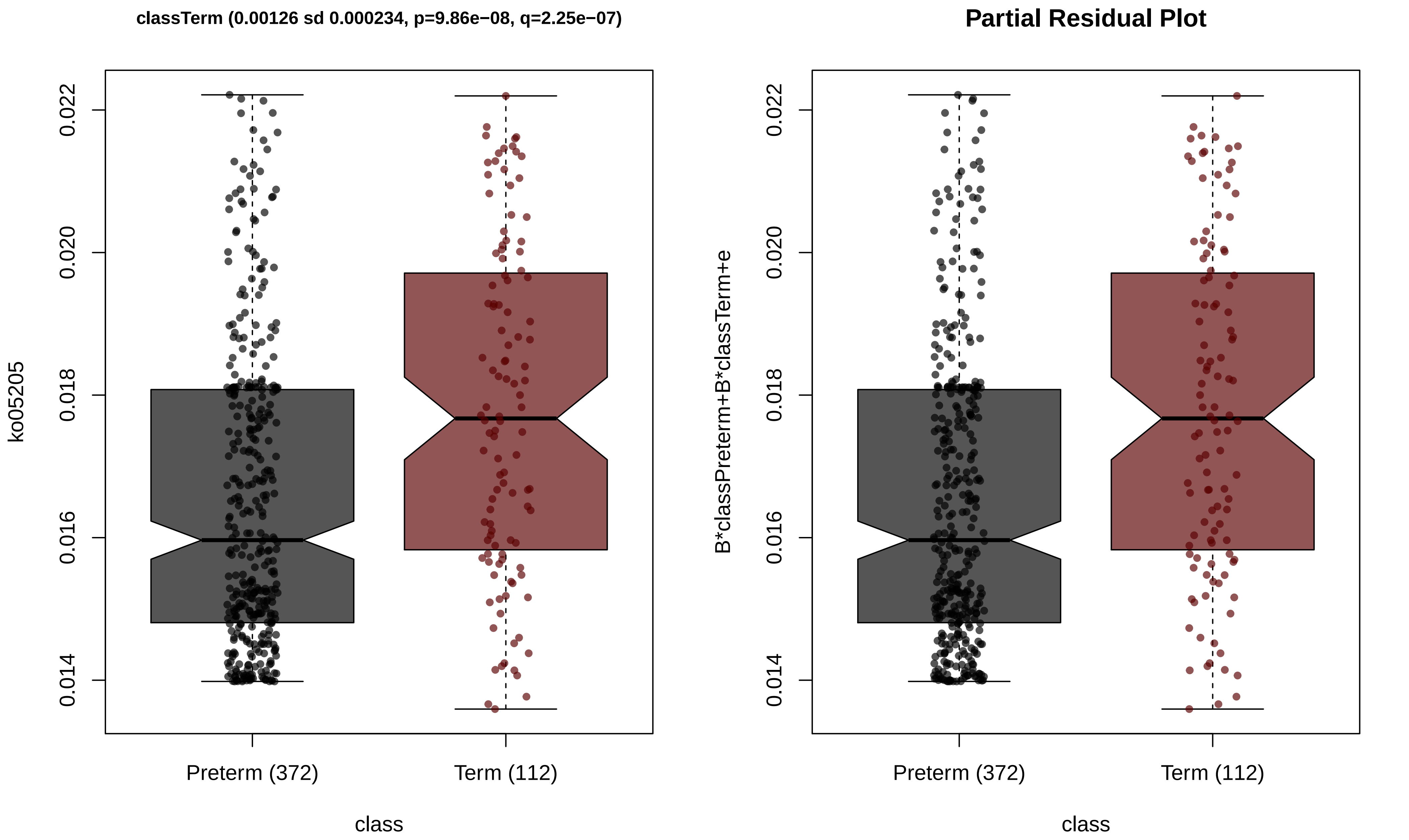

classTerm (0.00126 sd 0.000234, p=9.86e−08, q=2.25e−07)
Partial Residual Plot
ko05205
B*classPreterm+B*classTerm+e
0.014
0.016
0.018
0.020
0.022
Preterm (372)
Term (112)
class

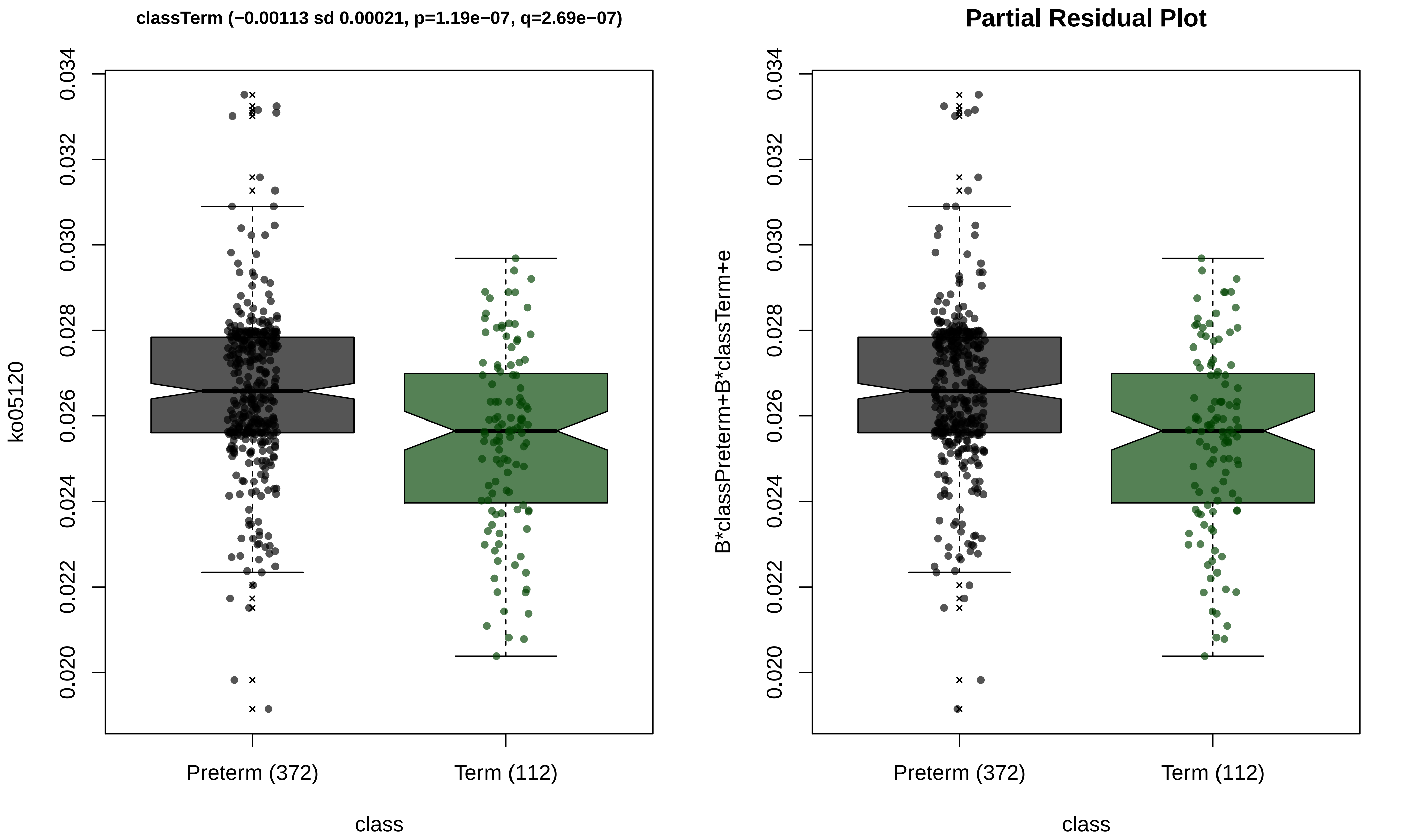
classTerm (−0.00113 sd 0.00021, p=1.19e−07, q=2.69e−07)
Partial Residual Plot
ko05120
B*classPreterm+B*classTerm+e
0.020
0.022
0.024
0.026
0.028
0.030
0.032
0.034
Preterm (372)
Term (112)
class

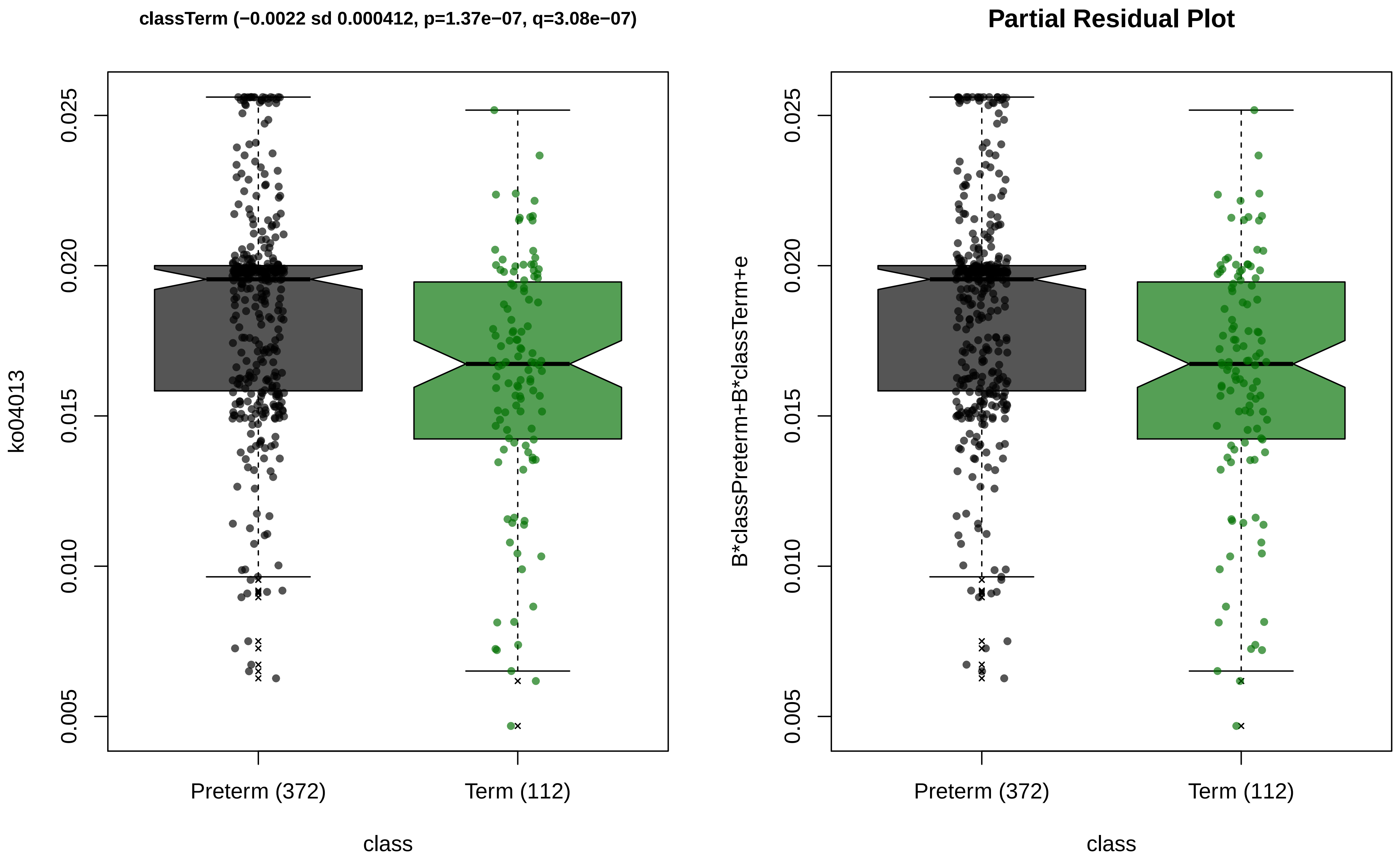

classTerm (−0.0022 sd 0.000412, p=1.37e−07, q=3.08e−07)
Partial Residual Plot
ko04013
B*classPreterm+B*classTerm+e
0.005
0.010
0.015
0.020
0.025
Preterm (372)
Term (112)
class

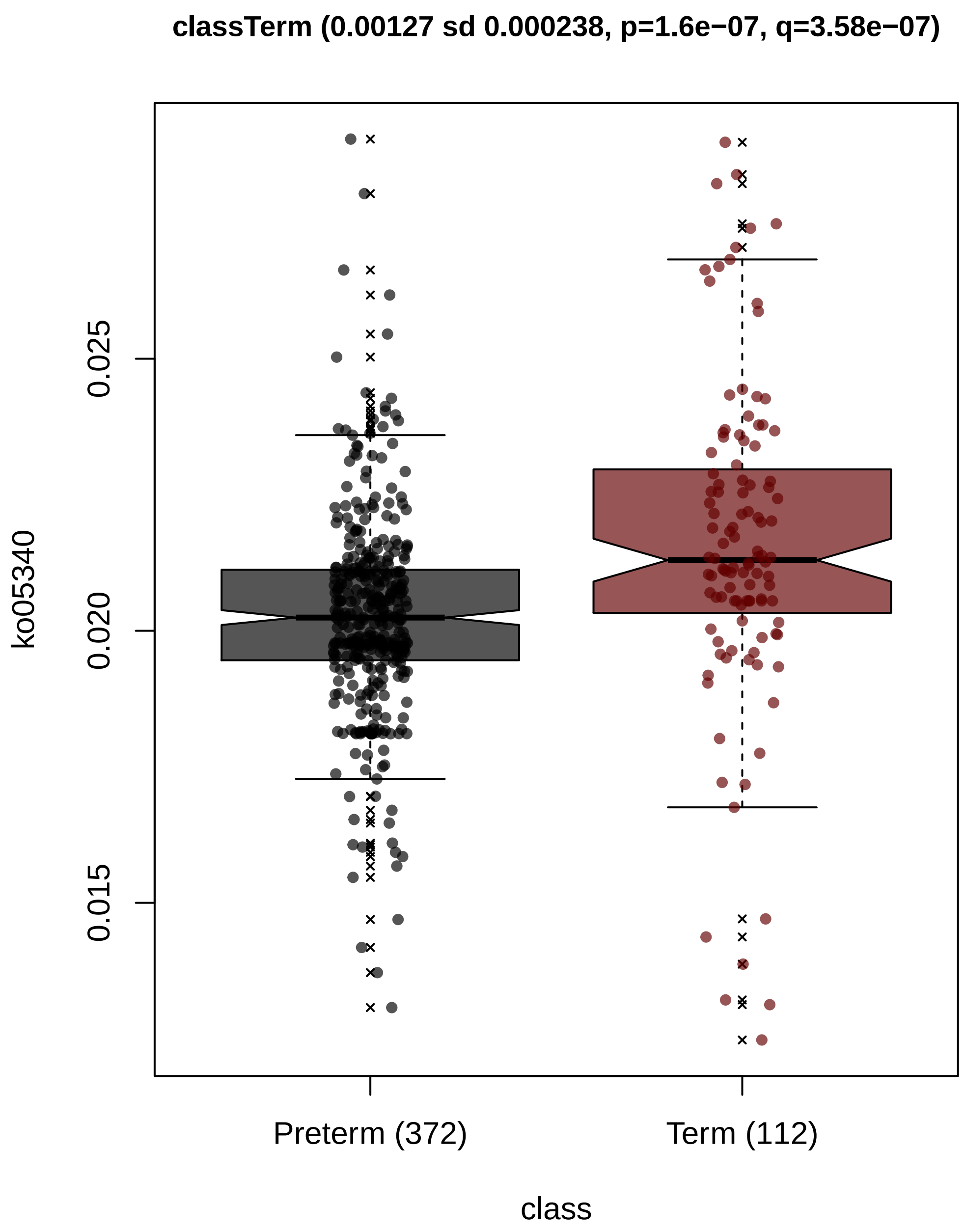
classTerm (0.00127 sd 0.000238, p=1.6e−07, q=3.58e−07)
ko05340
0.015
0.020
0.025
Preterm (372)
Term (112)
class

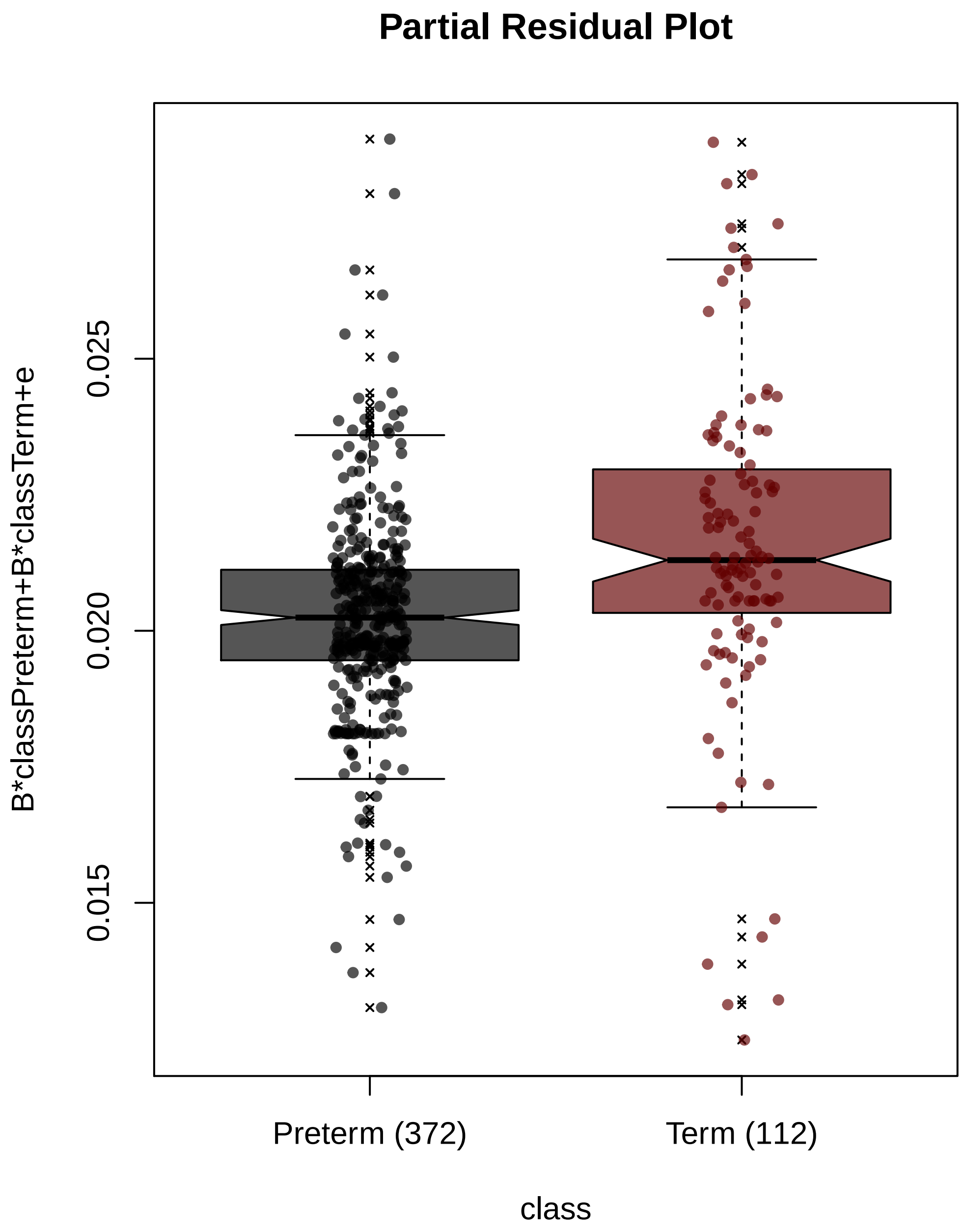
Partial Residual Plot
B*classPreterm+B*classTerm+e
0.015
0.020
0.025
Preterm (372)
Term (112)
class

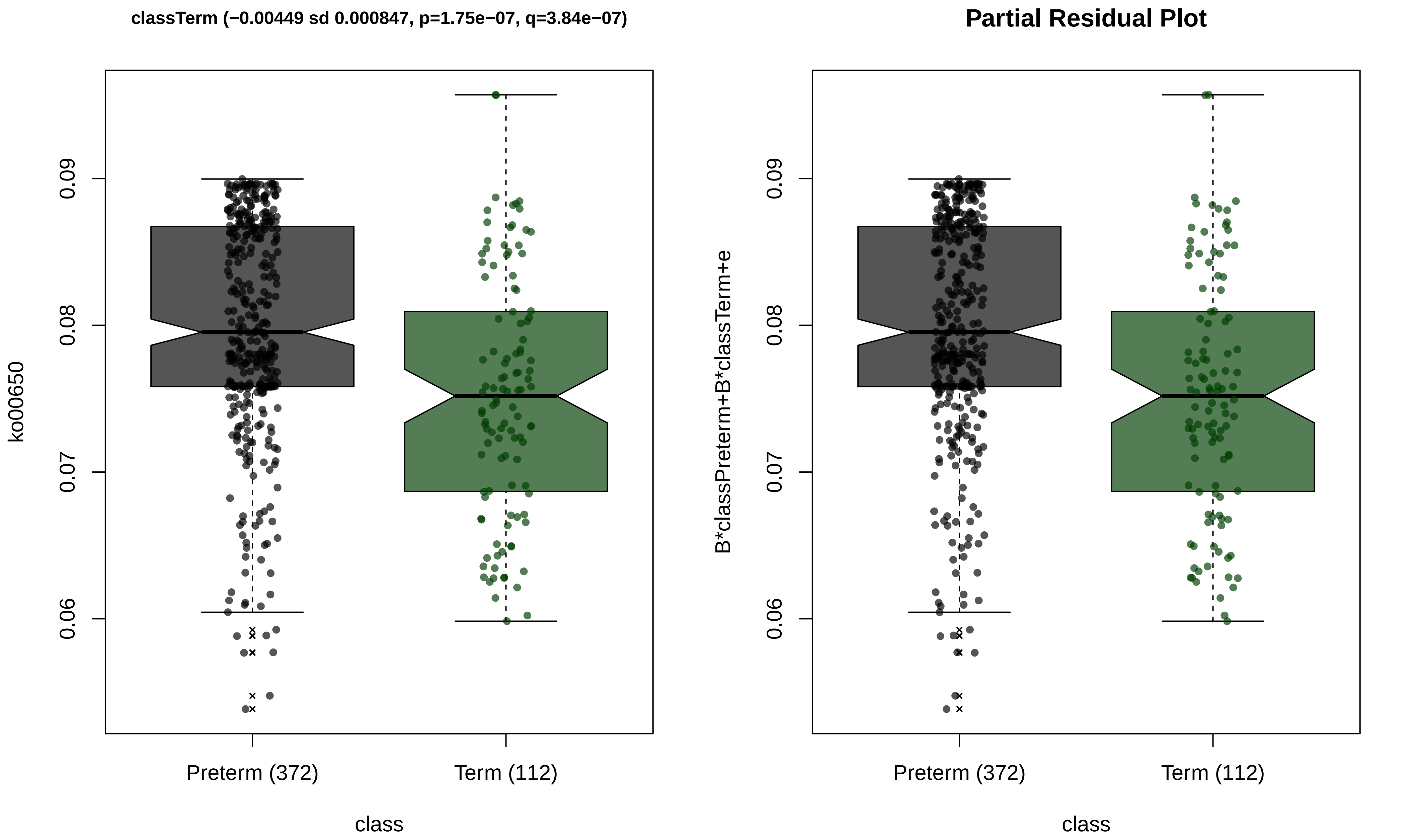
classTerm (−0.00449 sd 0.000847, p=1.75e−07, q=3.84e−07)
Partial Residual Plot
ko00650
B*classPreterm+B*classTerm+e
0.06
0.07
0.08
0.09
Preterm (372)
Term (112)
class

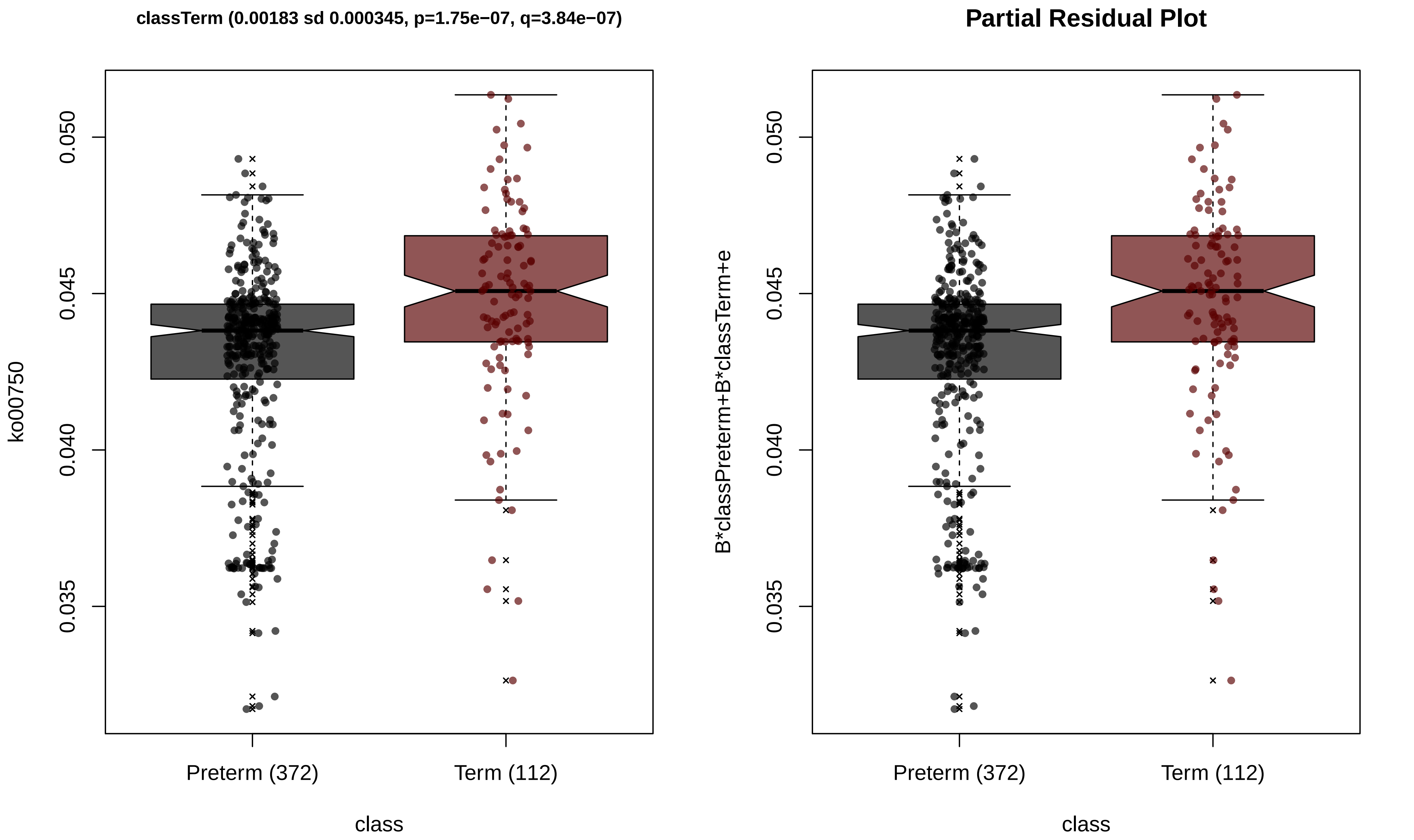
classTerm (0.00183 sd 0.000345, p=1.75e−07, q=3.84e−07)
ko00750
0.035
0.040
0.045
0.050
Preterm (372)
Term (112)
class
Partial Residual Plot
B*classPreterm+B*classTerm+e
0.035
0.040
0.045
0.050
Preterm (372)
Term (112)
class

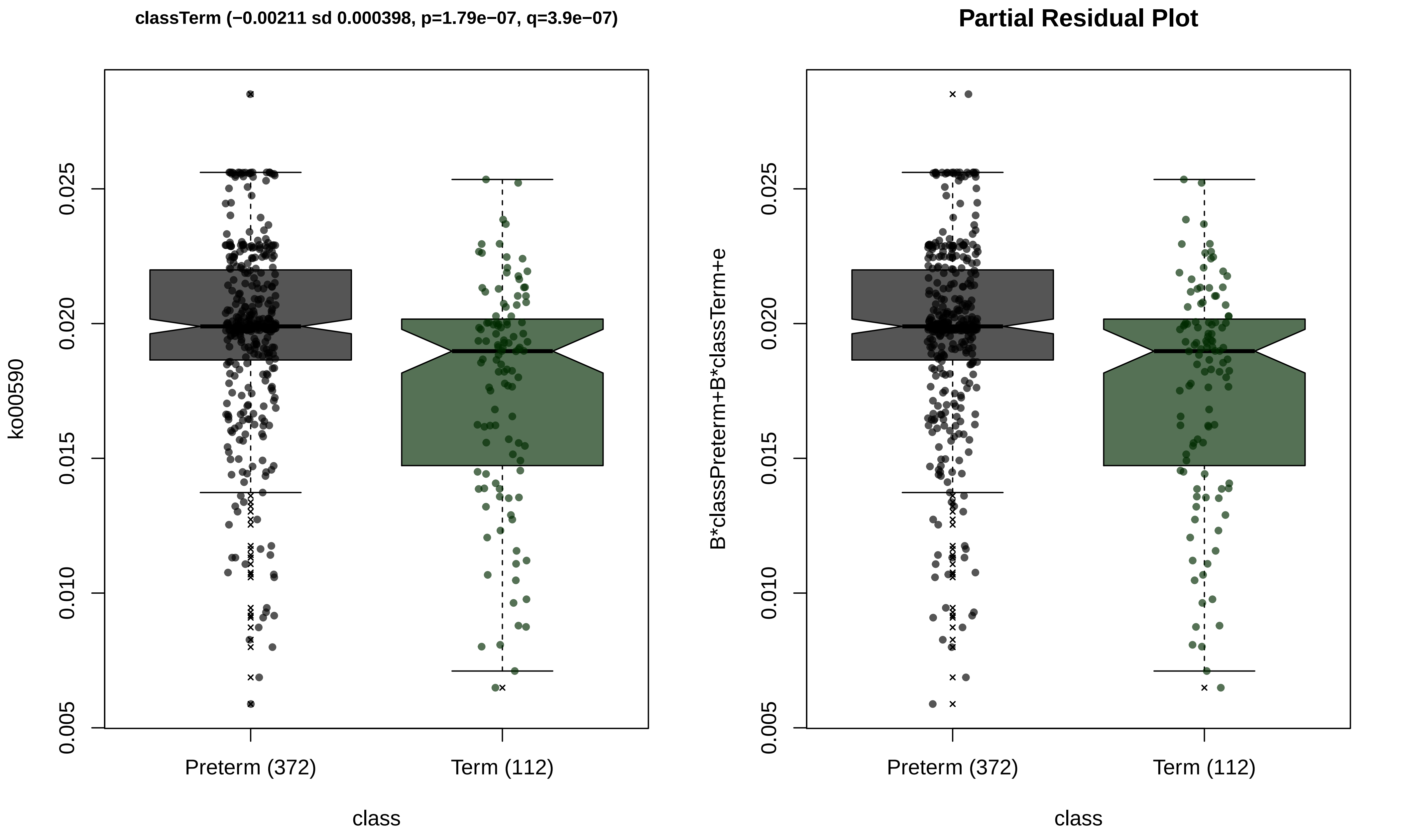

classTerm (−0.00211 sd 0.000398, p=1.79e−07, q=3.9e−07)
ko00590
0.005
0.010
0.015
0.020
0.025
Preterm (372)
Term (112)
class
Partial Residual Plot
B*classPreterm+B*classTerm+e
0.005
0.010
0.015
0.020
0.025
Preterm (372)
Term (112)
class

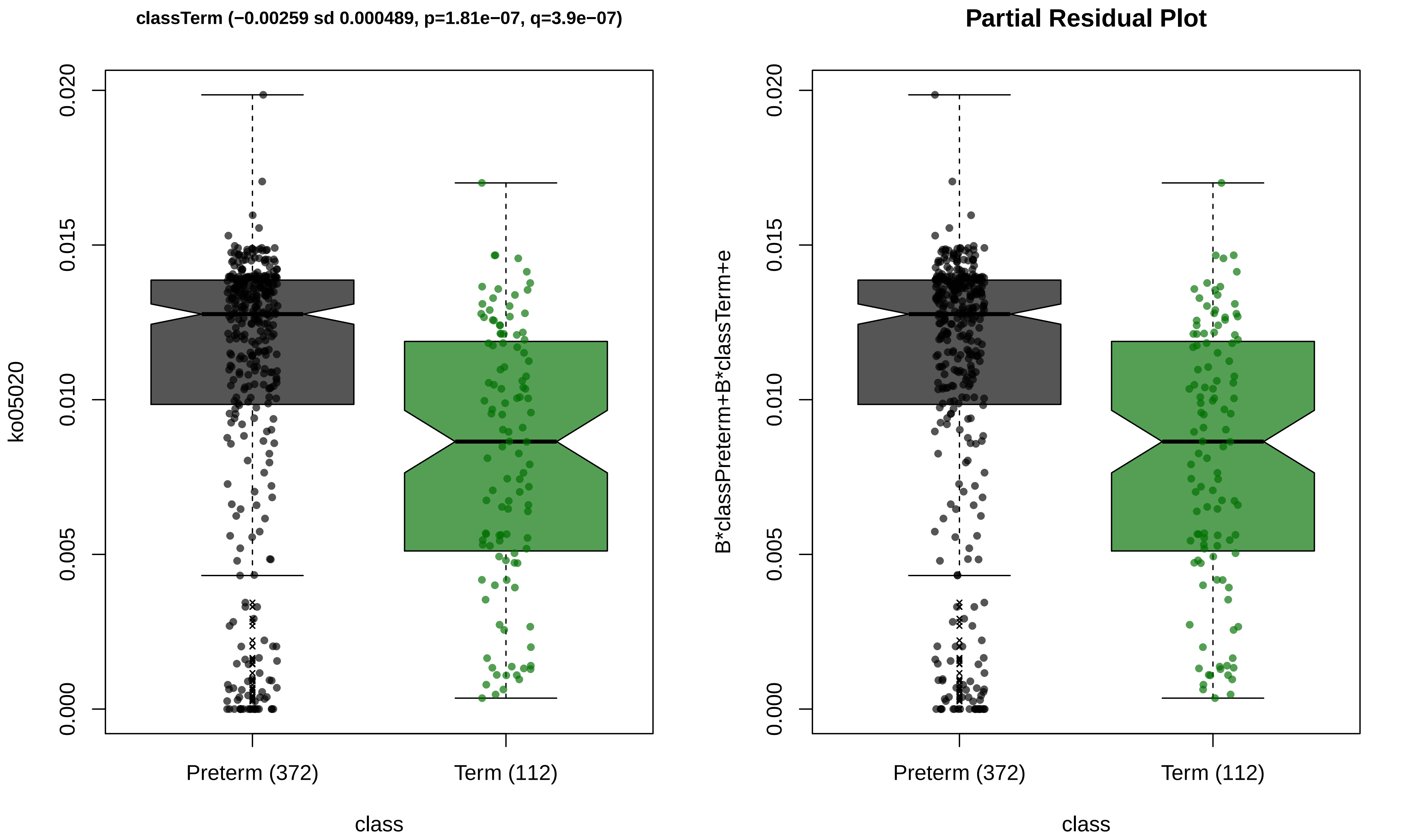

classTerm (−0.00259 sd 0.000489, p=1.81e−07, q=3.9e−07)
ko05020
0.000
0.005
0.010
0.015
0.020
Preterm (372)
Term (112)
class
Partial Residual Plot
B*classPreterm+B*classTerm+e
0.000
0.005
0.010
0.015
0.020
Preterm (372)
Term (112)
class

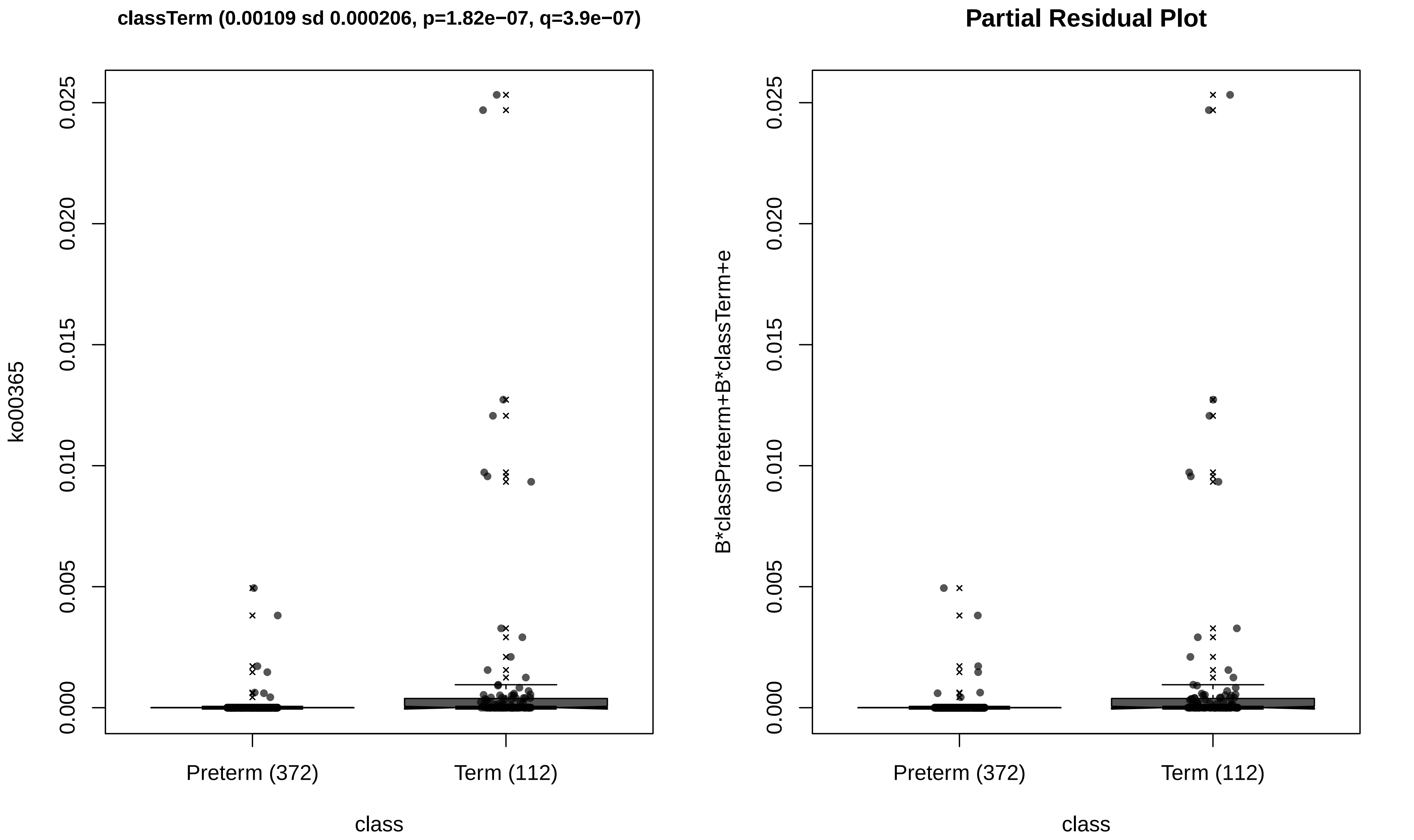

classTerm (0.00109 sd 0.000206, p=1.82e−07, q=3.9e−07)
Partial Residual Plot
ko00365
B*classPreterm+B*classTerm+e
0.025
0.020
0.015
0.010
0.005
0.000
Preterm (372)
Term (112)
class

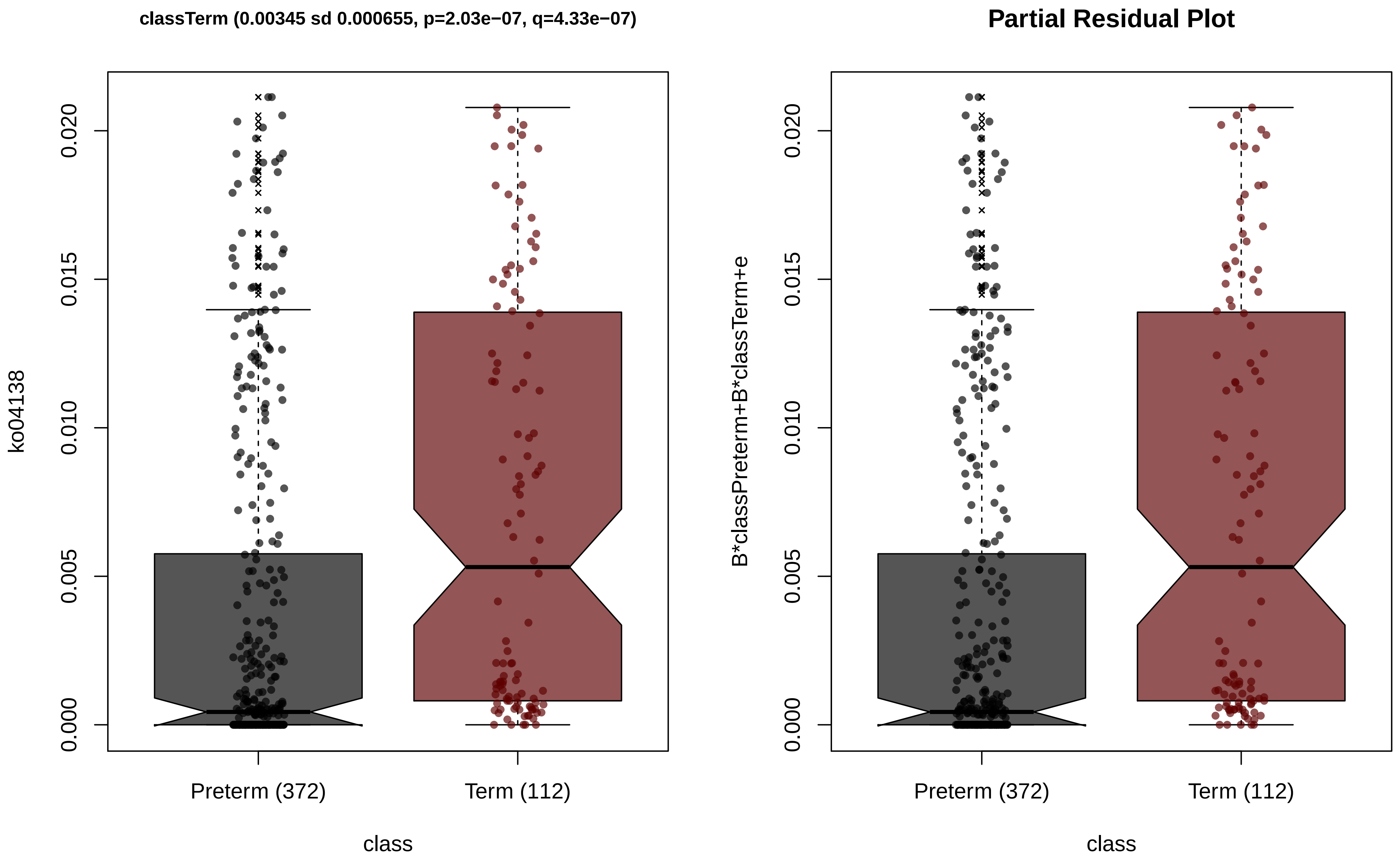
classTerm (0.00345 sd 0.000655, p=2.03e−07, q=4.33e−07)
ko04138
0.000
0.005
0.010
0.015
0.020
Preterm (372)
Term (112)
class
Partial Residual Plot
B*classPreterm+B*classTerm+e
0.000
0.005
0.010
0.015
0.020
Preterm (372)
Term (112)
class

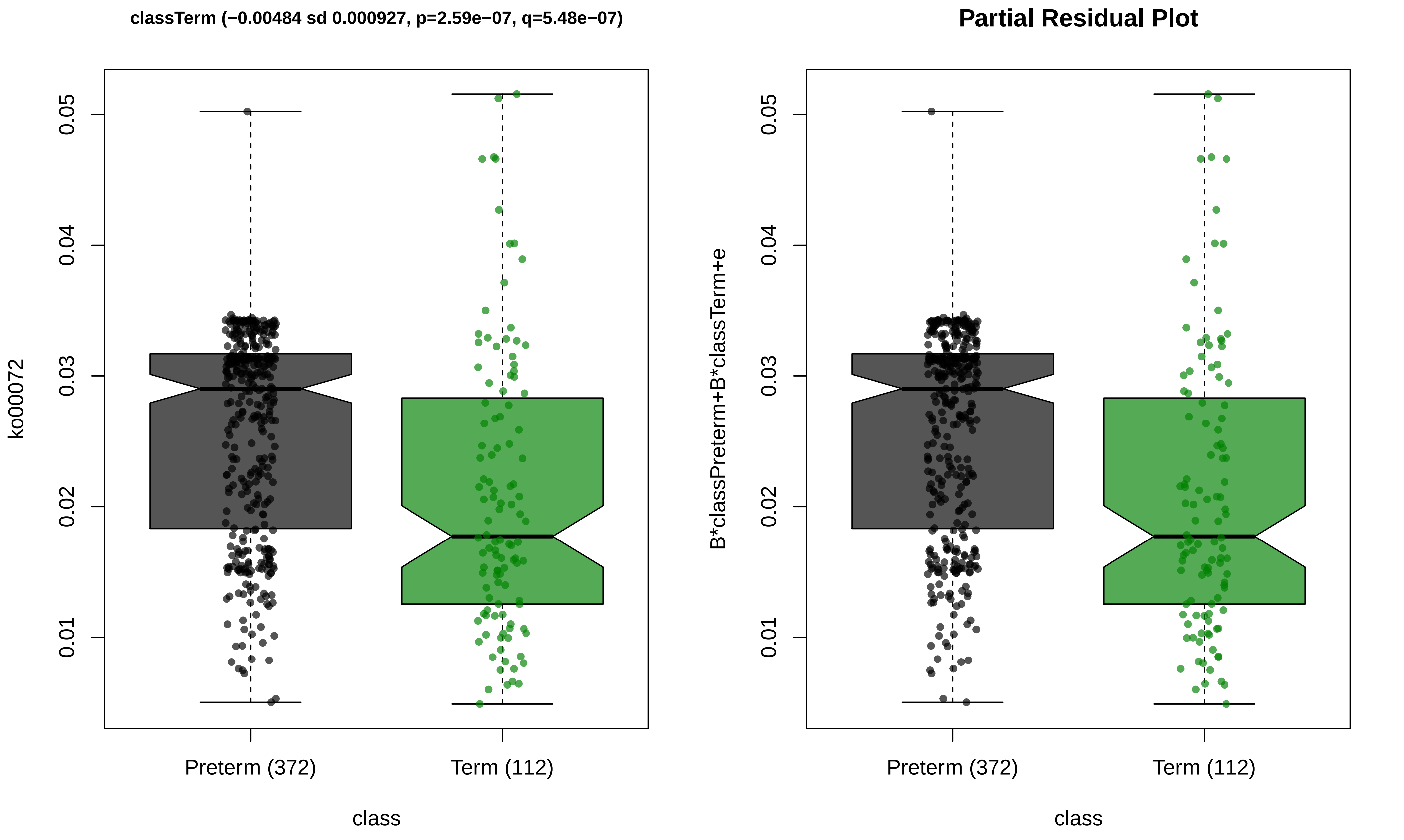
classTerm (−0.00484 sd 0.000927, p=2.59e−07, q=5.48e−07)
Partial Residual Plot
ko00072
B*classPreterm+B*classTerm+e
0.01
0.02
0.03
0.04
0.05
Preterm (372)
Term (112)
class

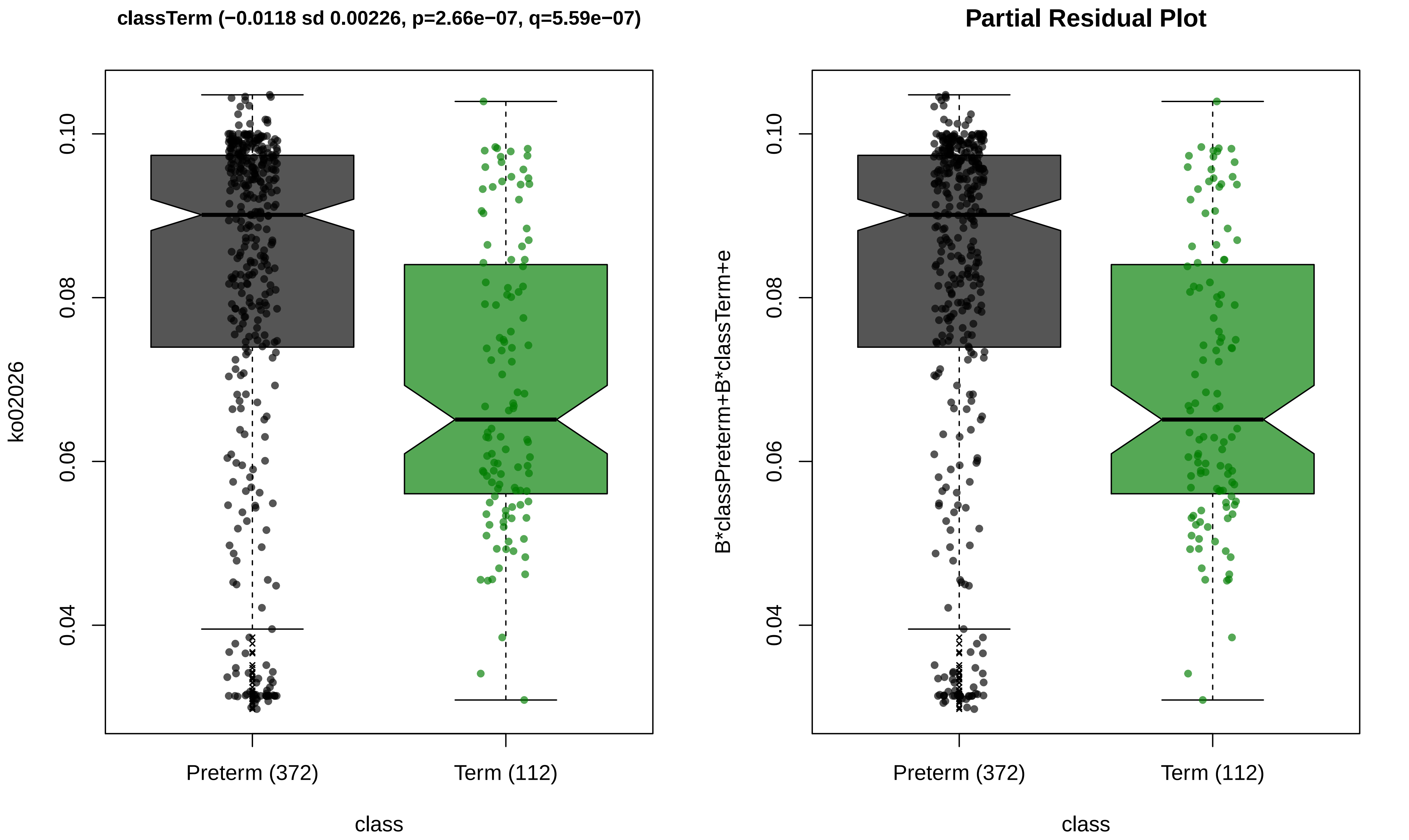

classTerm (−0.0118 sd 0.00226, p=2.66e−07, q=5.59e−07)
Partial Residual Plot
ko02026
B*classPreterm+B*classTerm+e
0.04
0.06
0.08
0.10
Preterm (372)
Term (112)
class

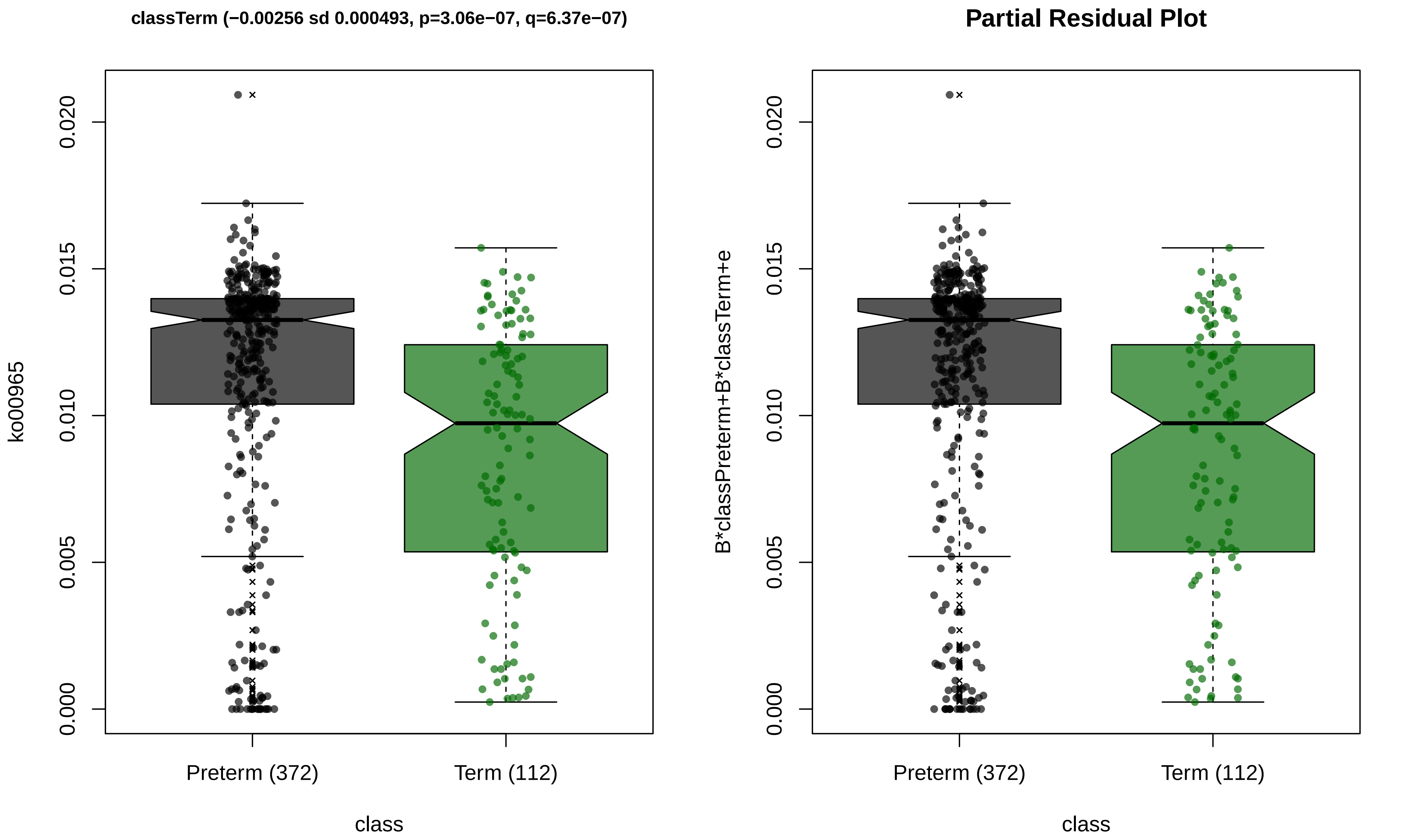

classTerm (−0.00256 sd 0.000493, p=3.06e−07, q=6.37e−07)
Partial Residual Plot
ko00965
B*classPreterm+B*classTerm+e
0.000
0.005
0.010
0.015
0.020
Preterm (372)
Term (112)
class

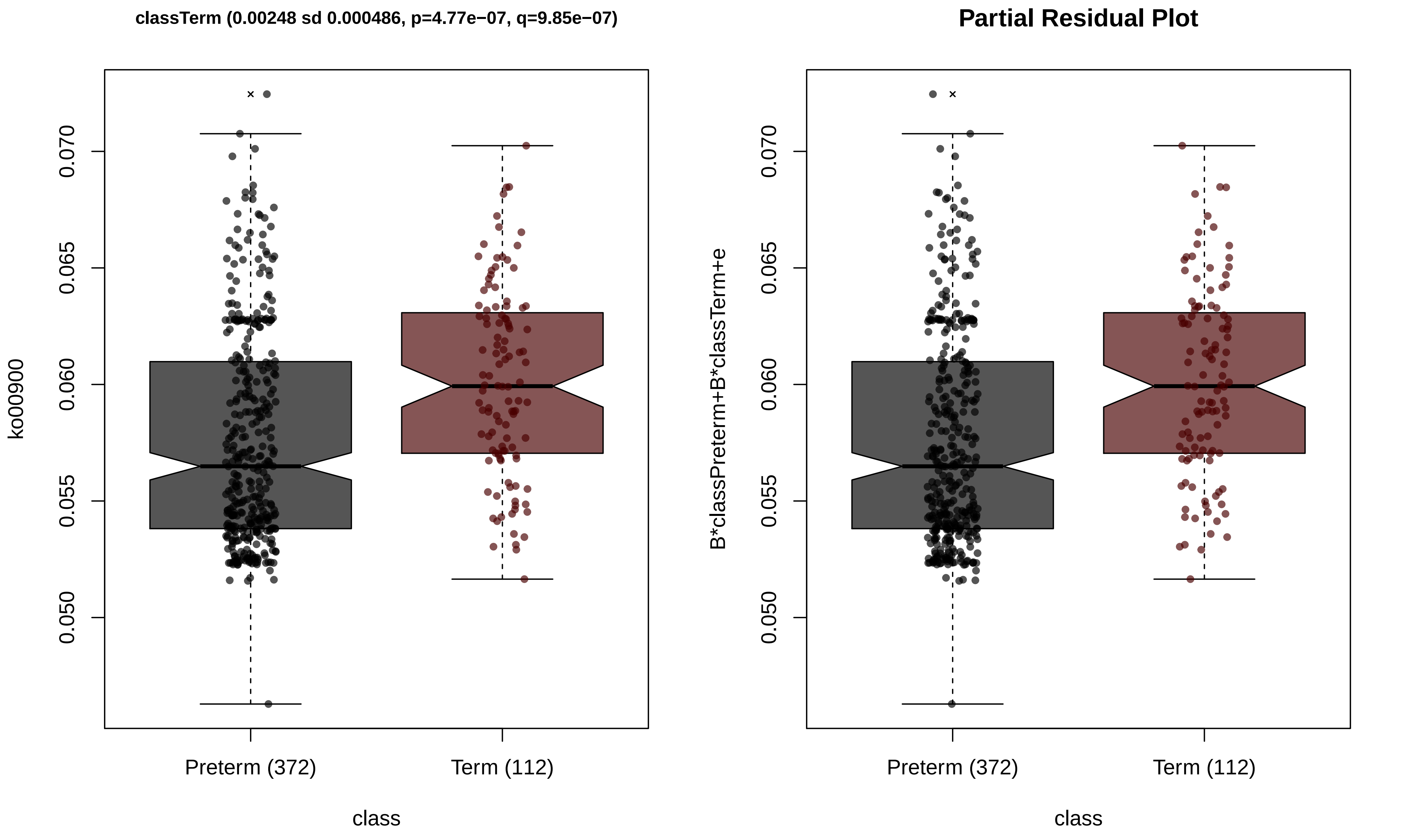
classTerm (0.00248 sd 0.000486, p=4.77e−07, q=9.85e−07)
ko00900
0.050
0.055
0.060
0.065
0.070
Preterm (372)
Term (112)
class
Partial Residual Plot
B*classPreterm+B*classTerm+e
0.050
0.055
0.060
0.065
0.070
Preterm (372)
Term (112)
class

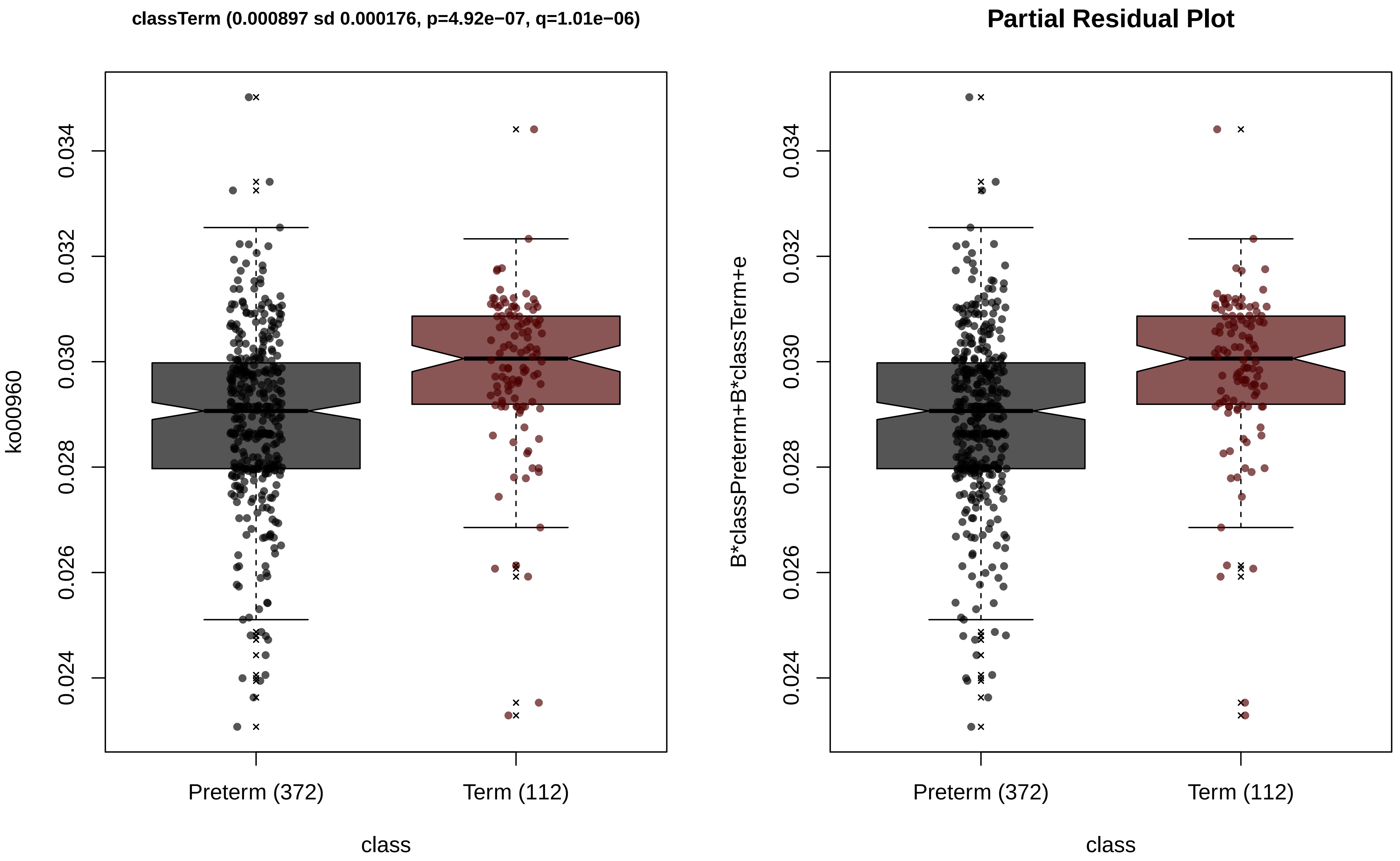
classTerm (0.000897 sd 0.000176, p=4.92e−07, q=1.01e−06)
ko00960
0.024
0.026
0.028
0.030
0.032
0.034
Preterm (372)
Term (112)
class
Partial Residual Plot
B*classPreterm+B*classTerm+e
0.024
0.026
0.028
0.030
0.032
0.034
Preterm (372)
Term (112)
class

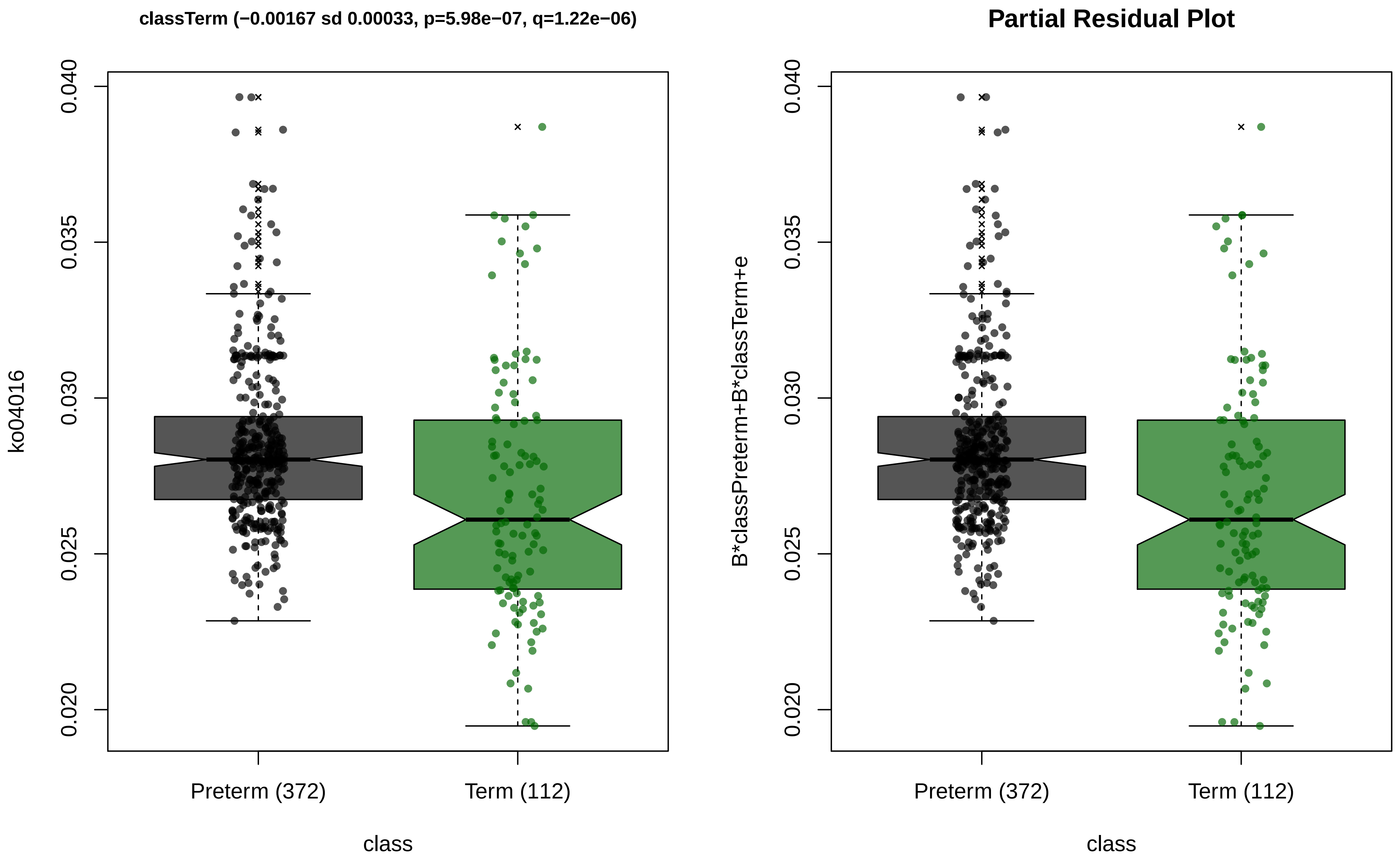
classTerm (−0.00167 sd 0.00033, p=5.98e−07, q=1.22e−06)
Partial Residual Plot
ko04016
B*classPreterm+B*classTerm+e
0.040
0.035
0.030
0.025
0.020
Preterm (372)
Term (112)
class

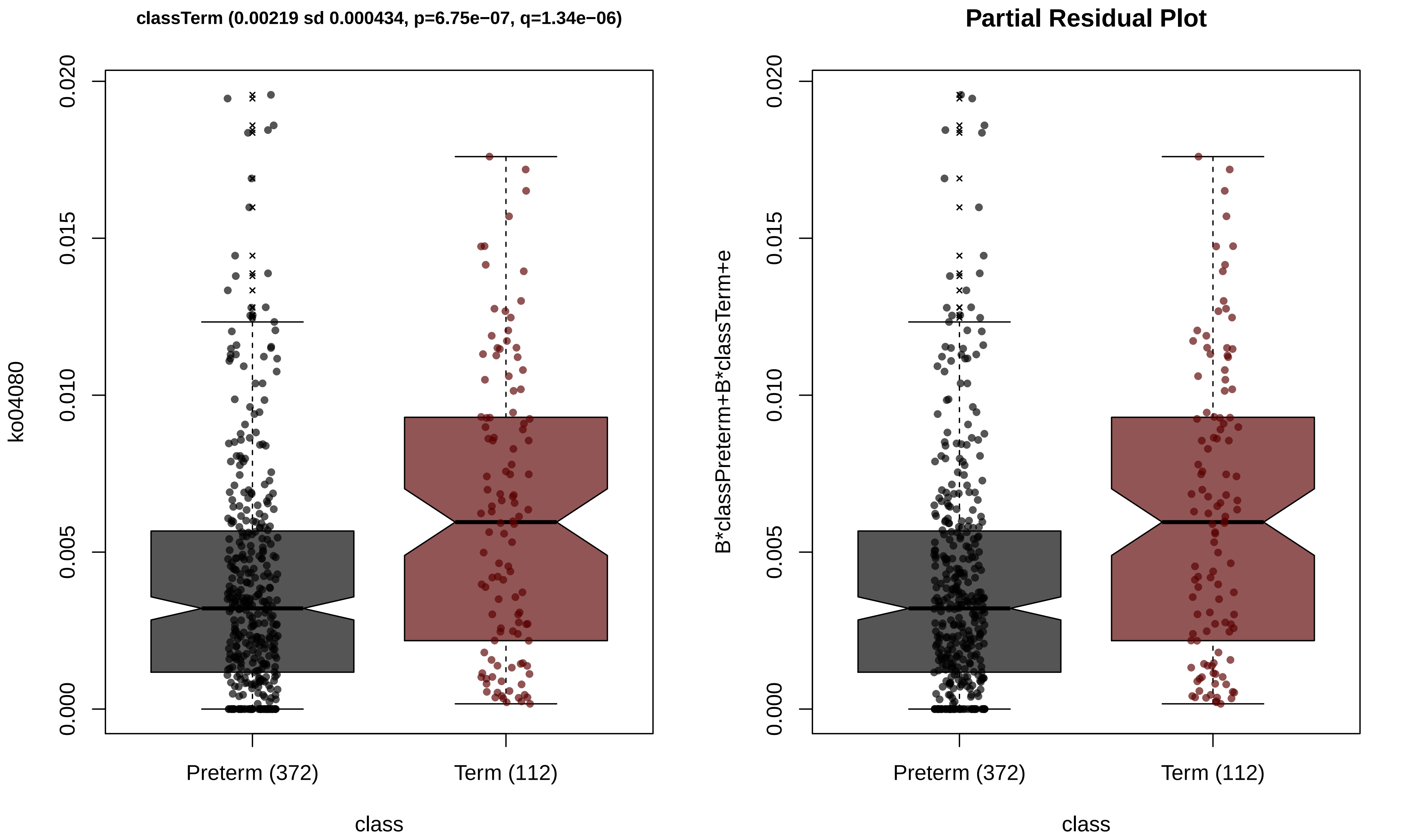

classTerm (0.00219 sd 0.000434, p=6.75e−07, q=1.34e−06)
Partial Residual Plot
ko04080
B*classPreterm+B*classTerm+e
0.000
0.005
0.010
0.015
0.020
Preterm (372)
Term (112)
class

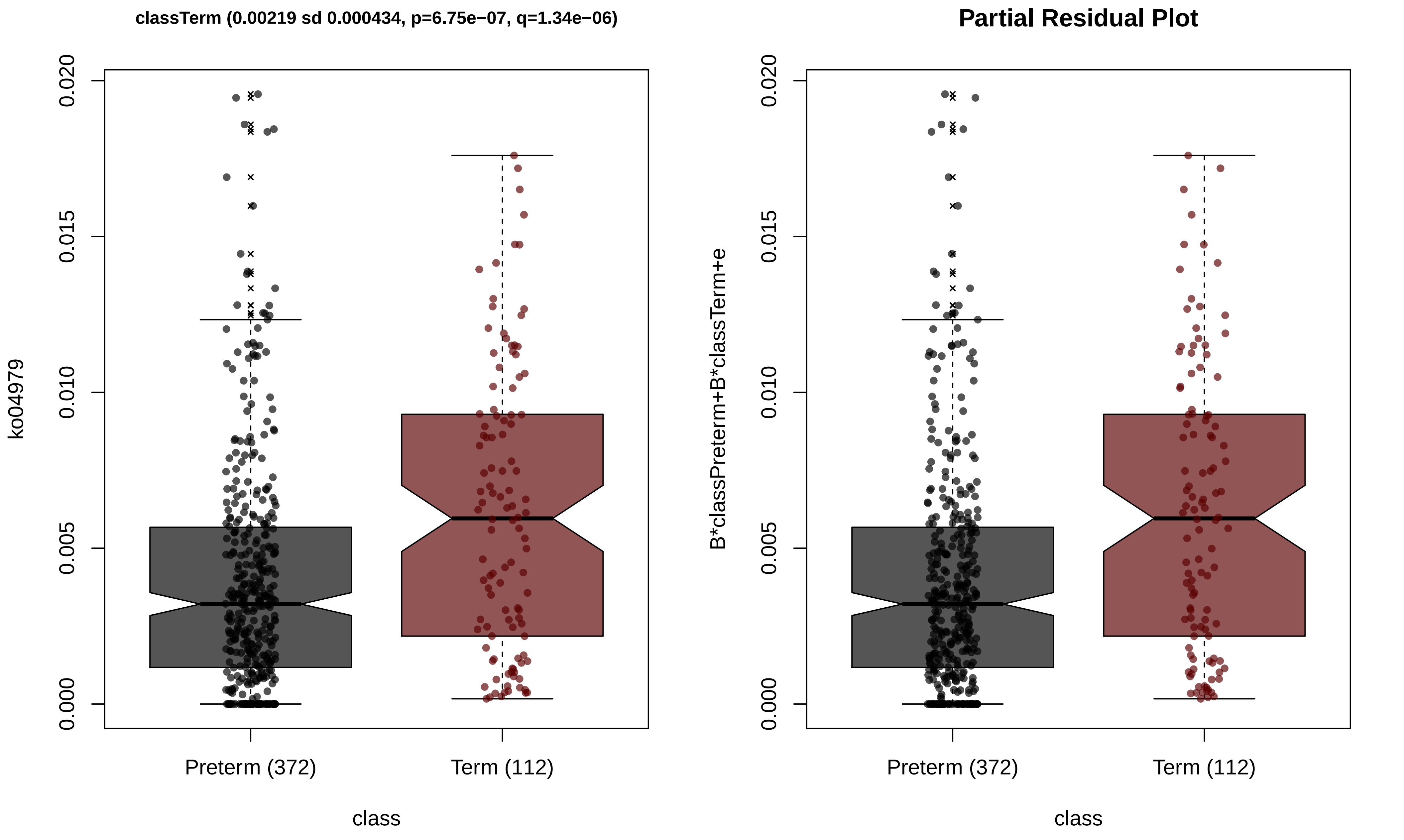

classTerm (0.00219 sd 0.000434, p=6.75e−07, q=1.34e−06)
Partial Residual Plot
ko04979
B*classPreterm+B*classTerm+e
0.000
0.005
0.010
0.015
0.020
Preterm (372)
Term (112)
class

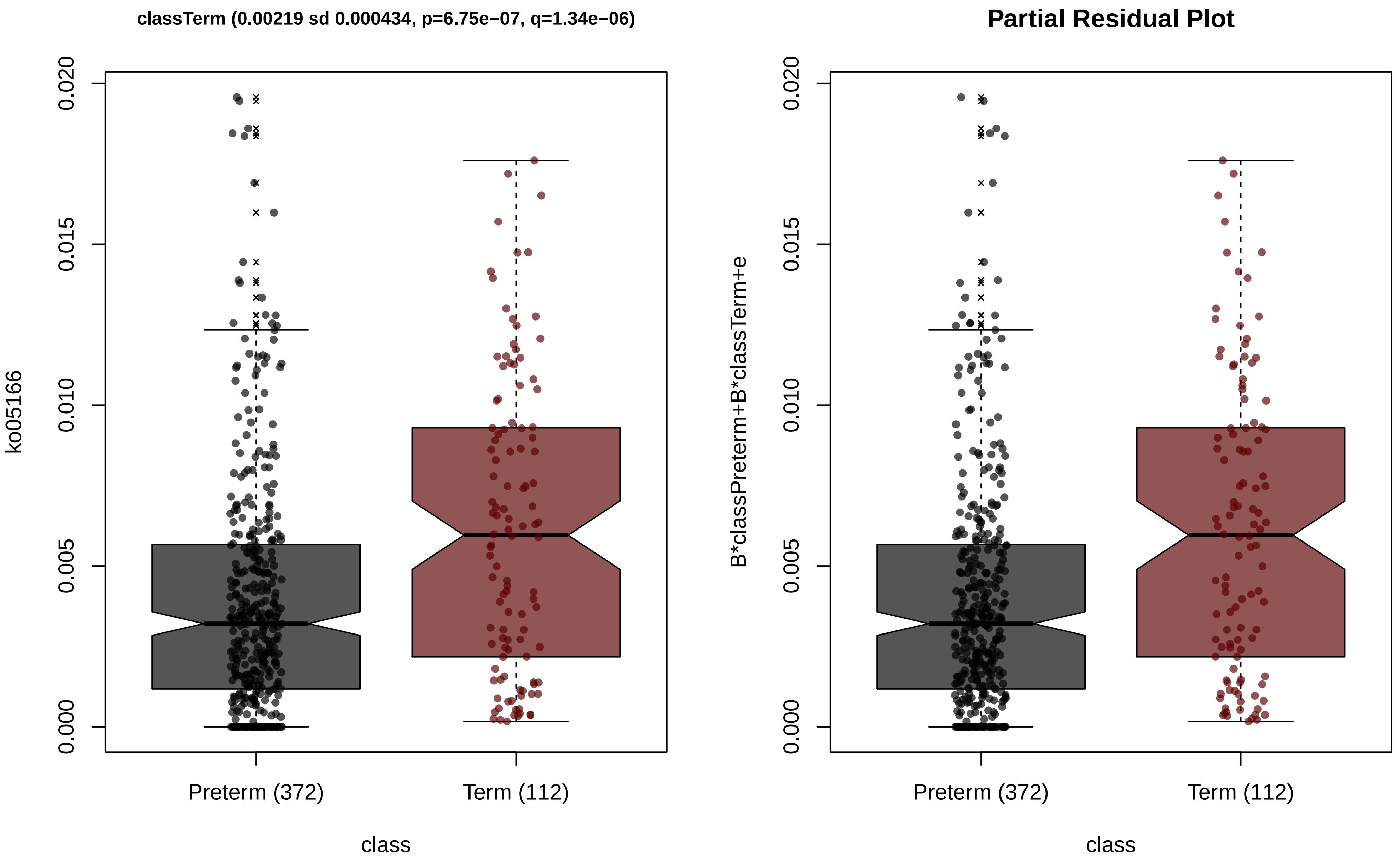

classTerm (0.00219 sd 0.000434, p=6.75e−07, q=1.34e−06)
Partial Residual Plot
ko05166
B*classPreterm+B*classTerm+e
0.000
0.005
0.010
0.015
0.020
Preterm (372)
Term (112)
class

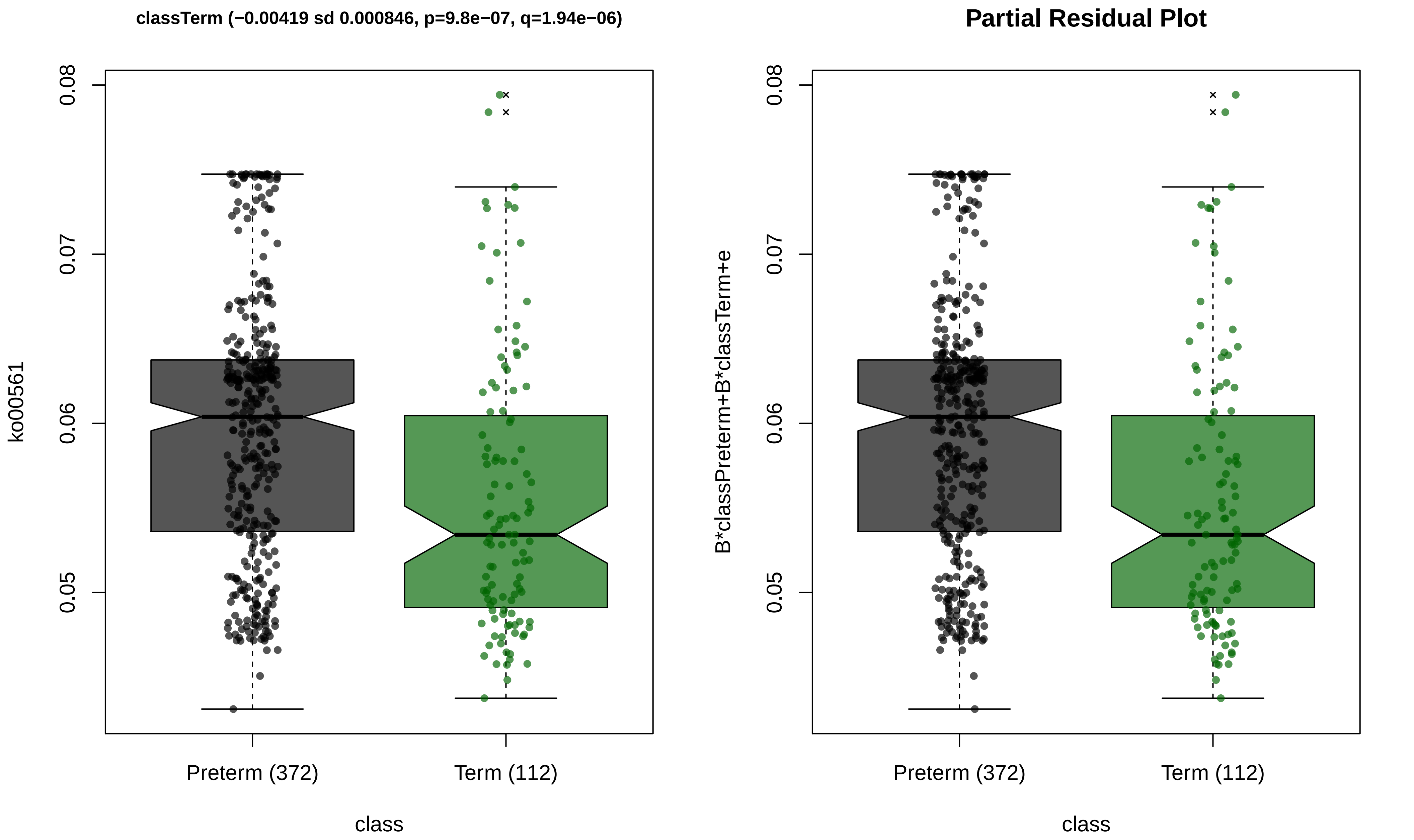
classTerm (−0.00419 sd 0.000846, p=9.8e−07, q=1.94e−06)
Partial Residual Plot
ko00561
B*classPreterm+B*classTerm+e
0.08
0.07
0.06
0.05
Preterm (372)
Term (112)
class

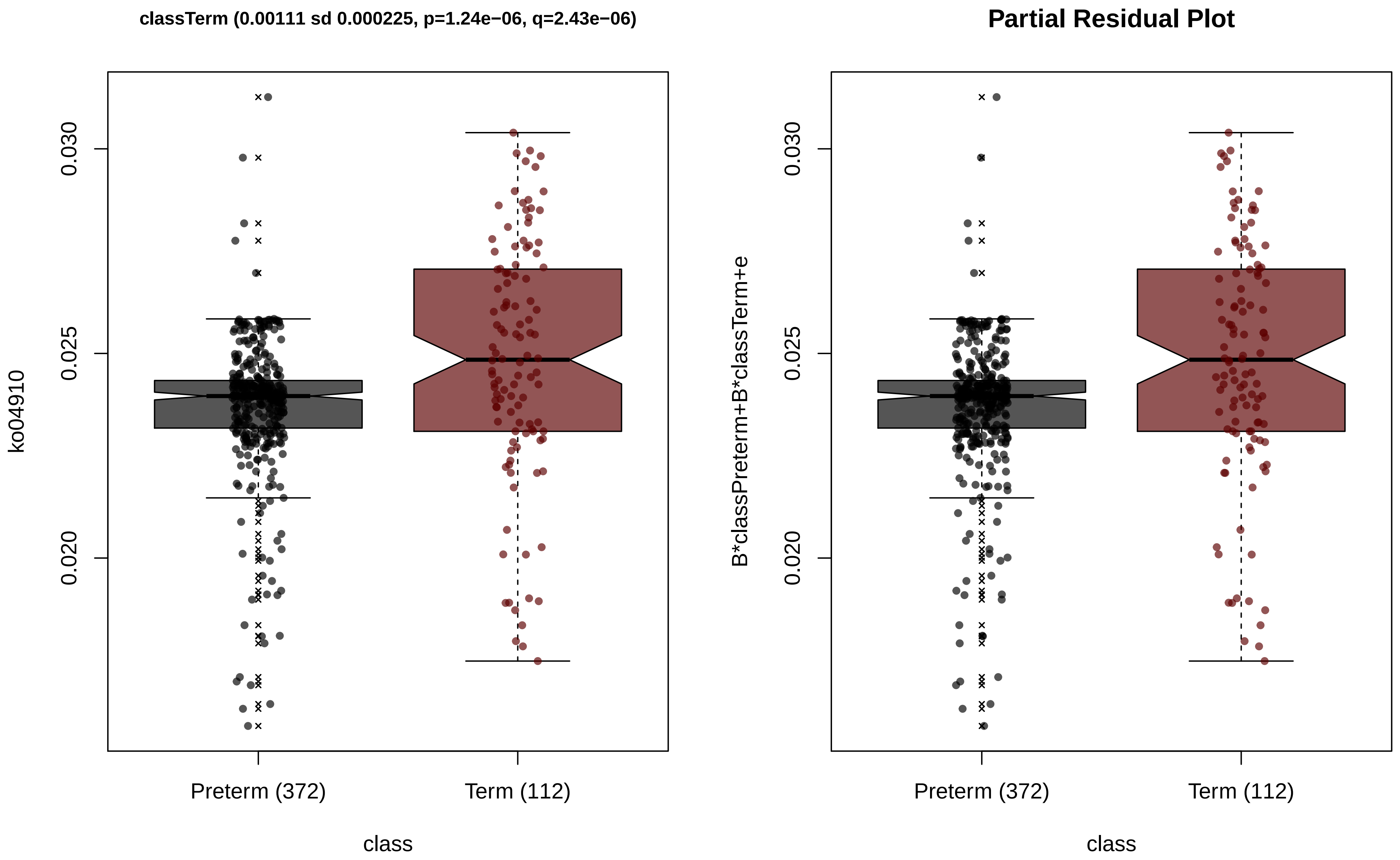

classTerm (0.00111 sd 0.000225, p=1.24e−06, q=2.43e−06)
ko04910
0.030
0.025
0.020
Preterm (372)
Term (112)
class
Partial Residual Plot
B*classPreterm+B*classTerm+e
0.030
0.025
0.020
Preterm (372)
Term (112)
class

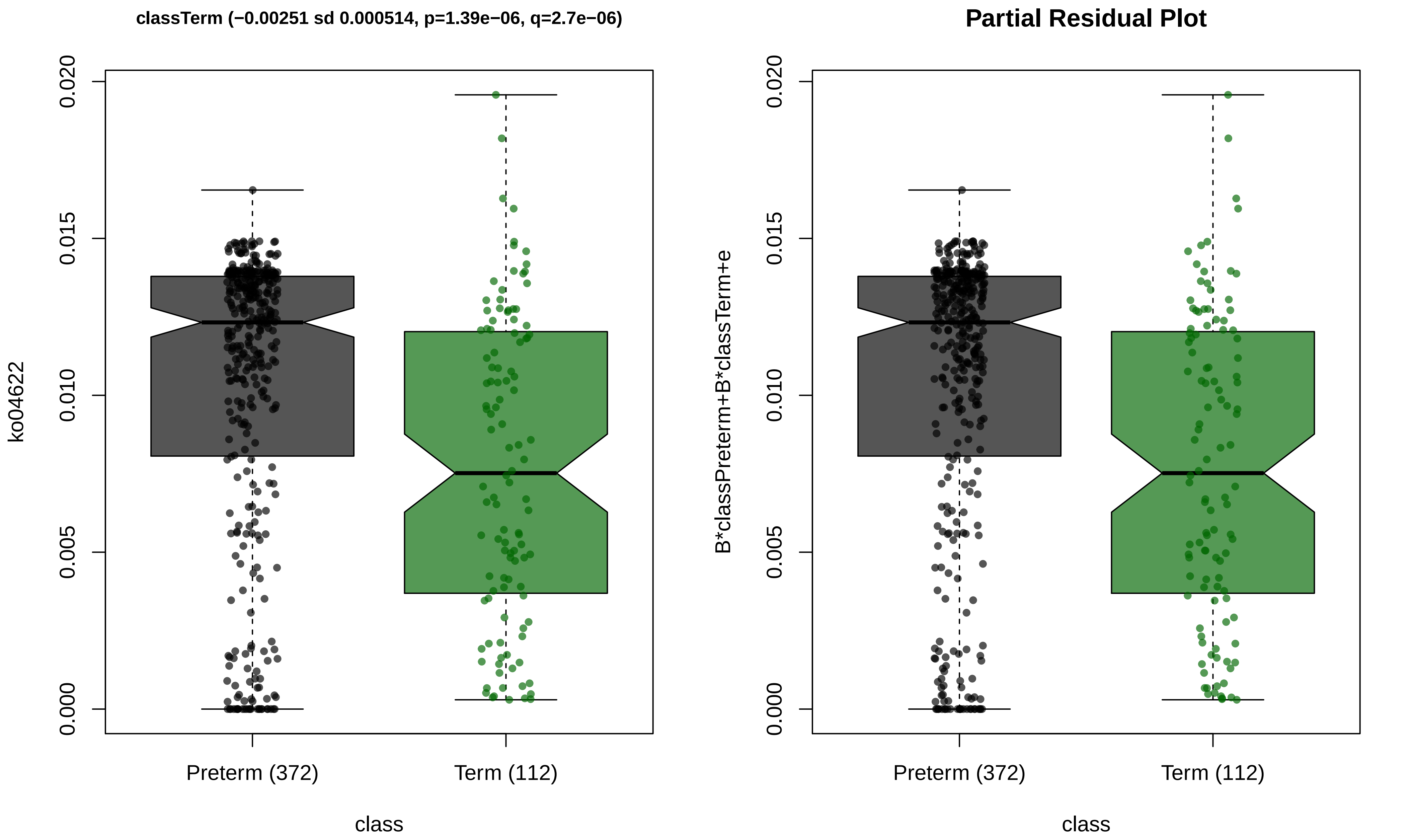
classTerm (−0.00251 sd 0.000514, p=1.39e−06, q=2.7e−06)
Partial Residual Plot
ko04622
B*classPreterm+B*classTerm+e
0.000
0.005
0.010
0.015
0.020
Preterm (372)
Term (112)
class

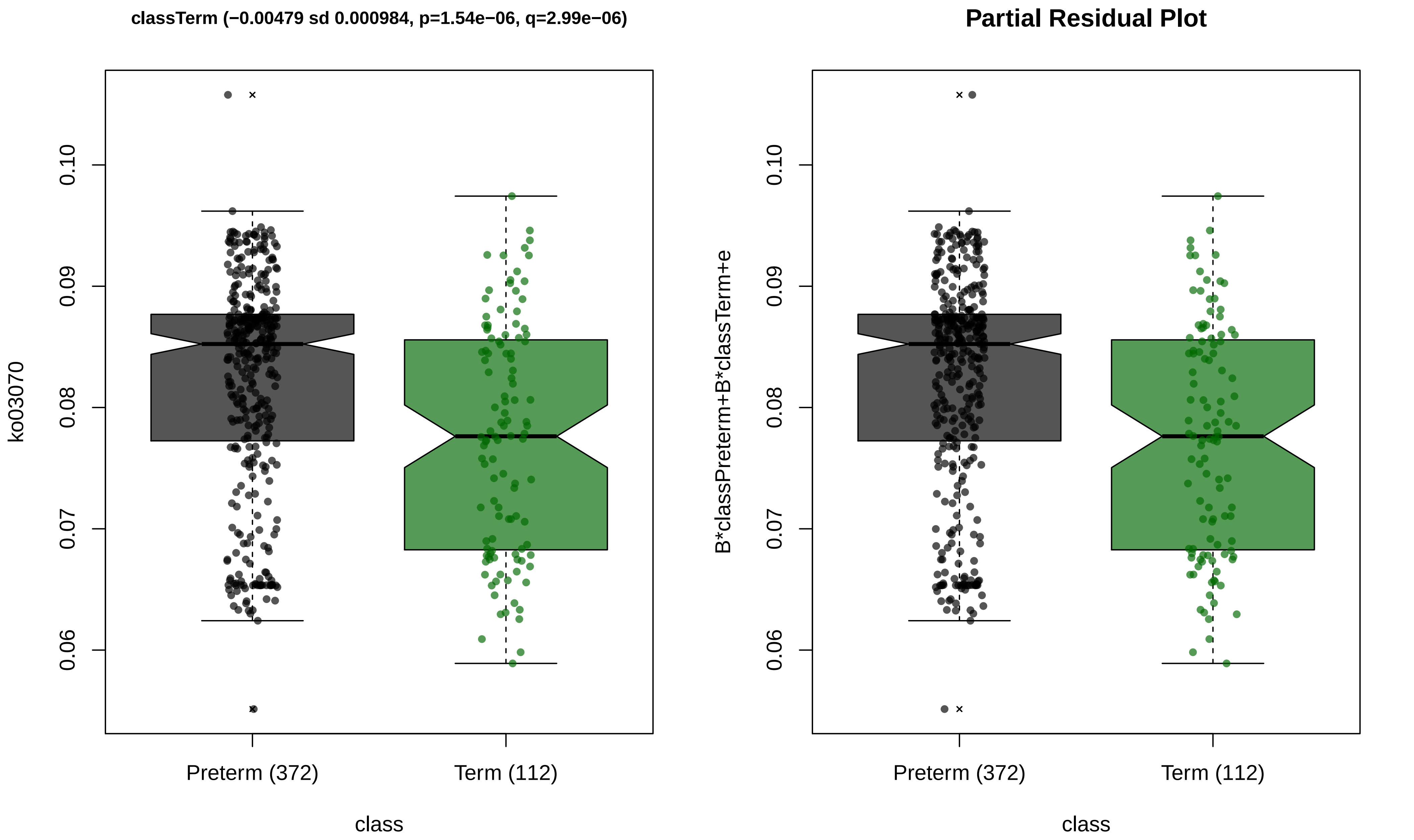

classTerm (−0.00479 sd 0.000984, p=1.54e−06, q=2.99e−06)
Partial Residual Plot
ko03070
B*classPreterm+B*classTerm+e
0.06
0.07
0.08
0.09
0.10
Preterm (372)
Term (112)
class

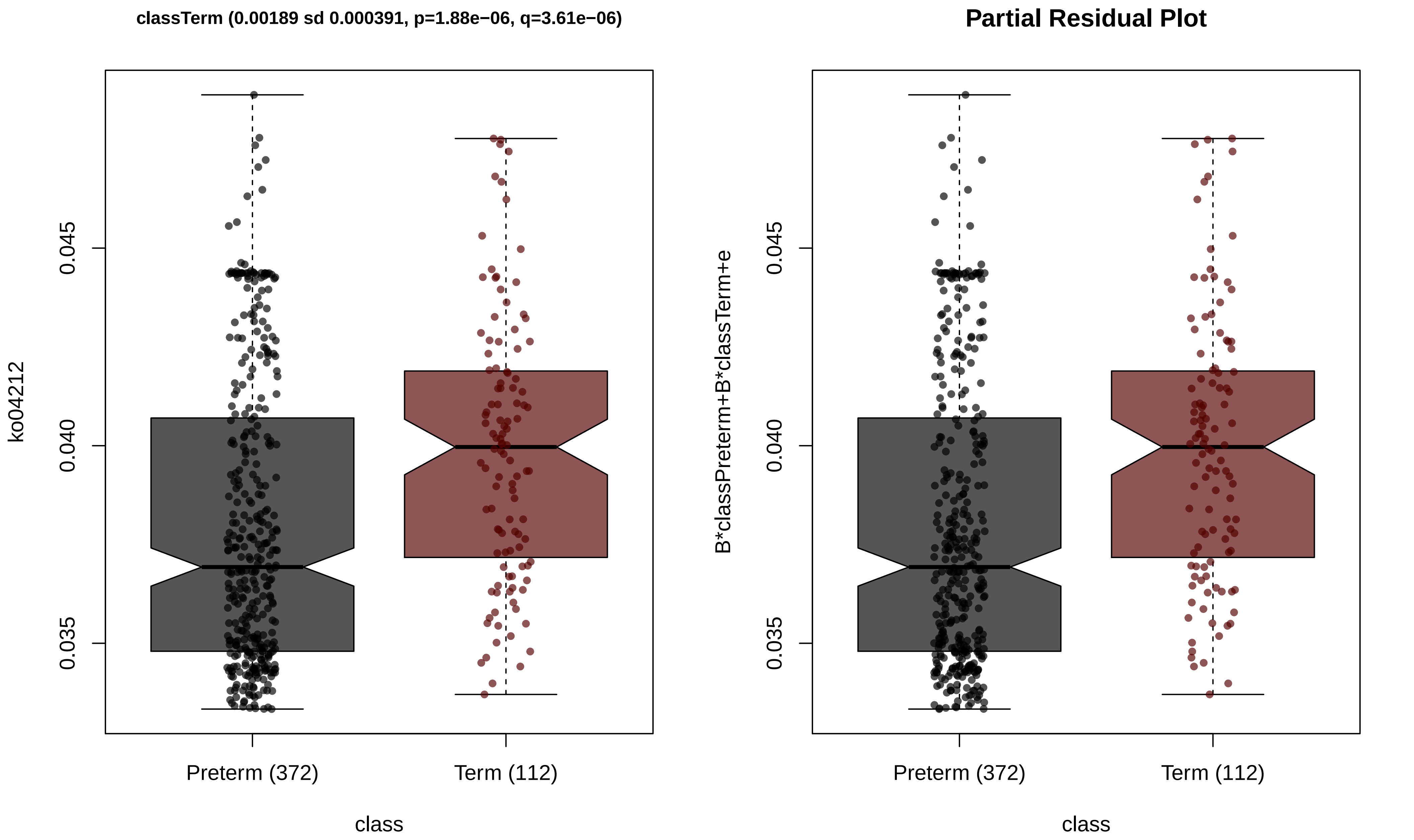

classTerm (0.00189 sd 0.000391, p=1.88e−06, q=3.61e−06)
Partial Residual Plot
ko04212
B*classPreterm+B*classTerm+e
0.035
0.040
0.045
Preterm (372)
Term (112)
class

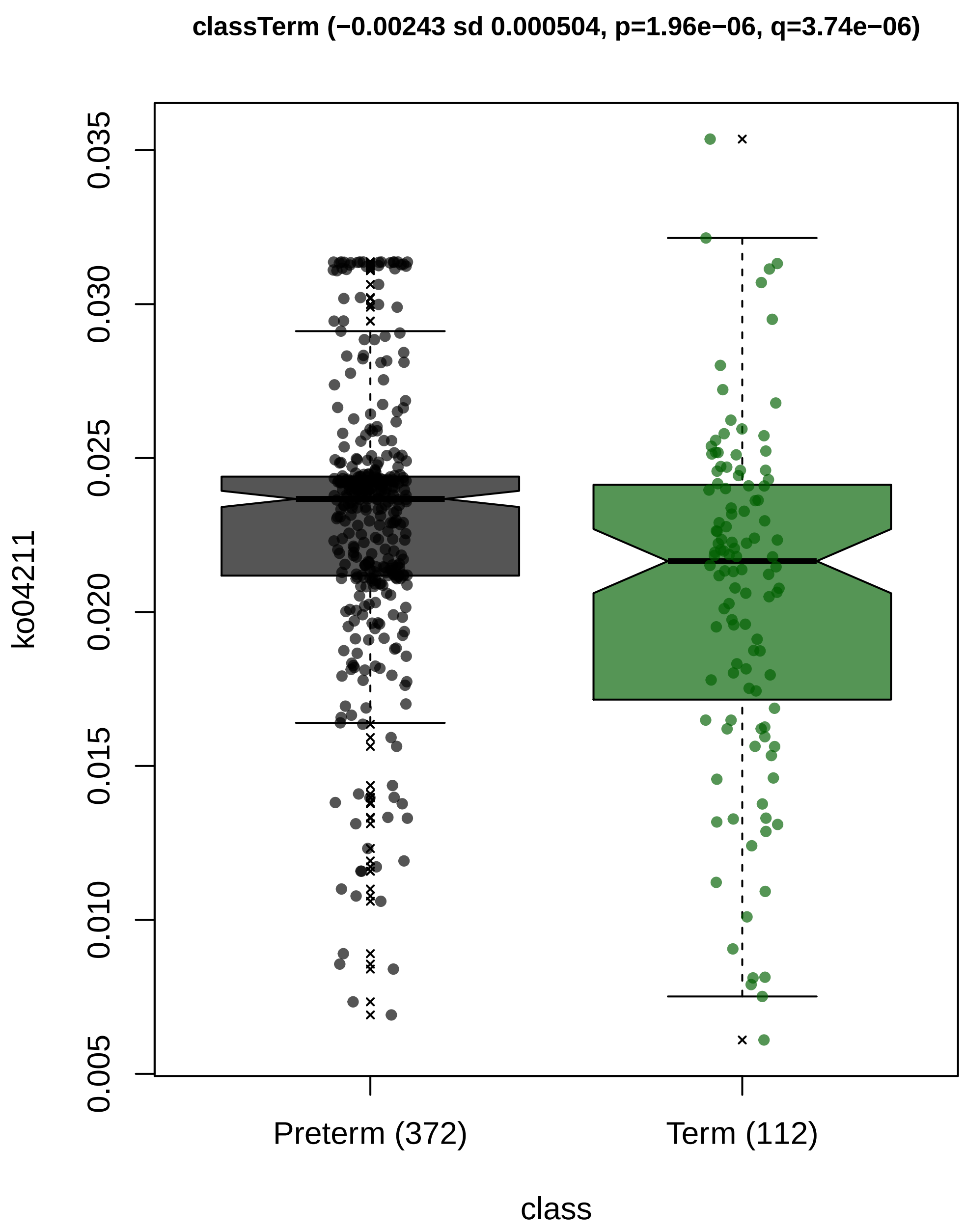

classTerm (−0.00243 sd 0.000504, p=1.96e−06, q=3.74e−06)
ko04211
0.005
0.010
0.015
0.020
0.025
0.030
0.035
Preterm (372)
Term (112)
class

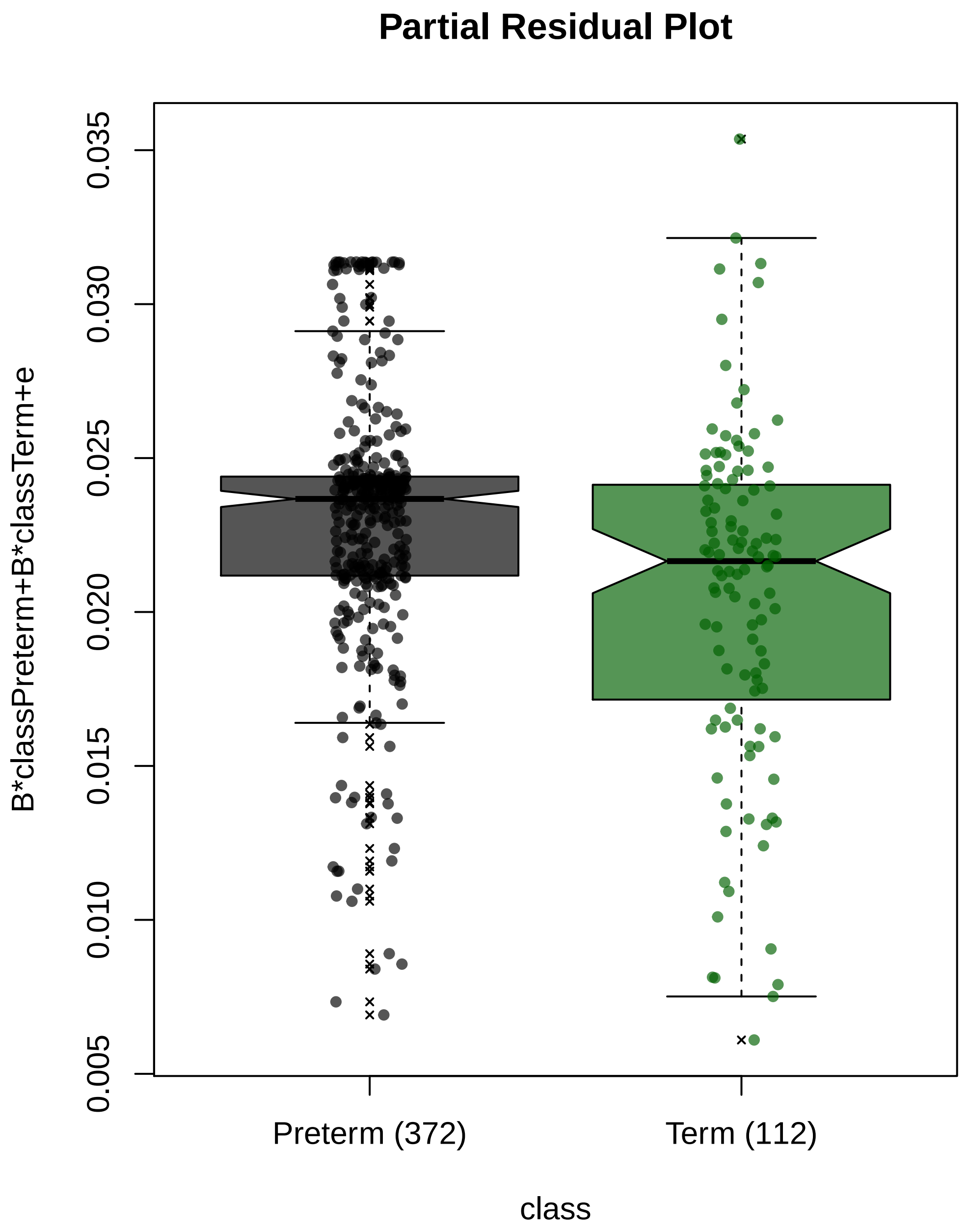

Partial Residual Plot
B*classPreterm+B*classTerm+e
0.005
0.010
0.015
0.020
0.025
0.030
0.035
Preterm (372)
Term (112)
class

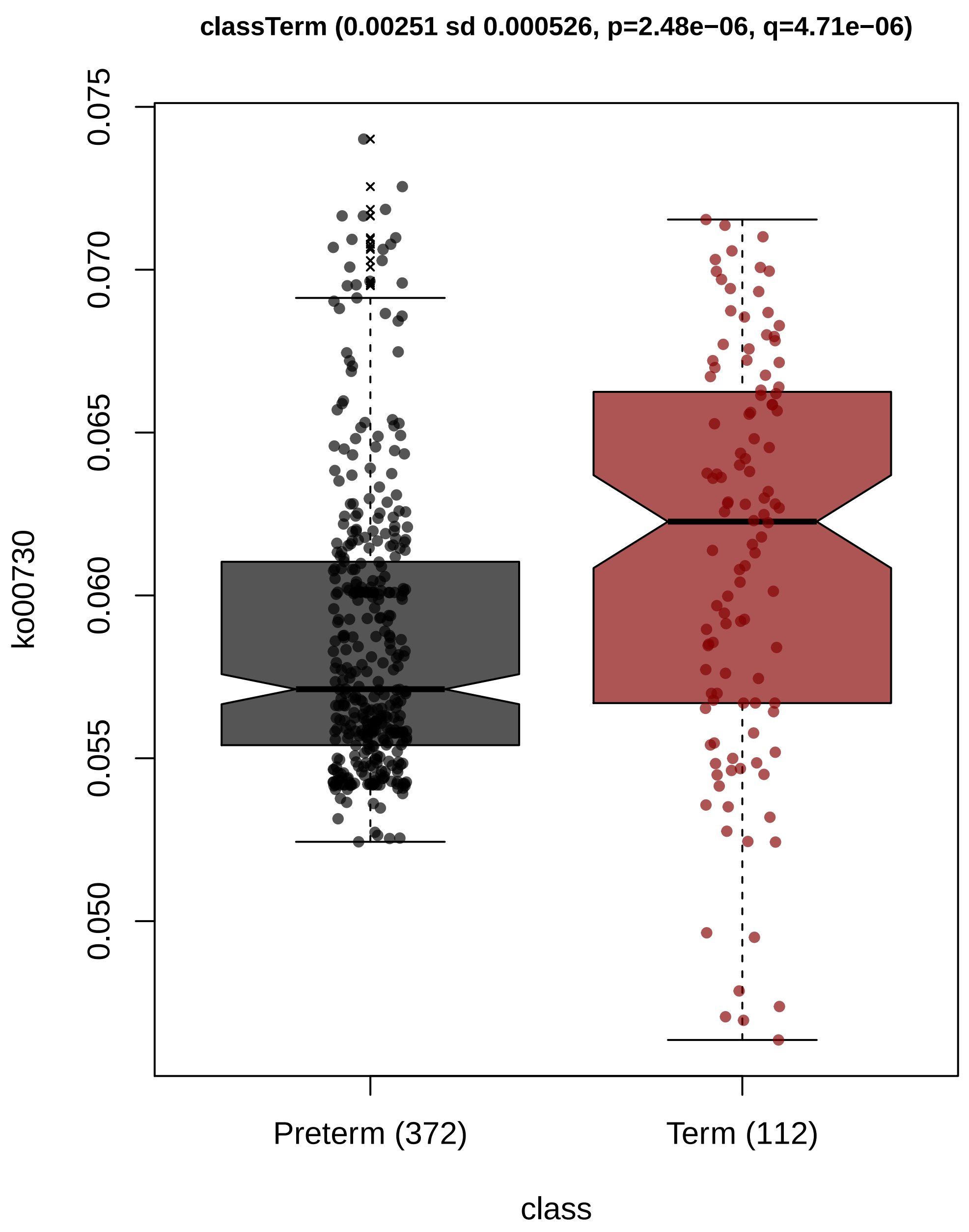

classTerm (0.00251 sd 0.000526, p=2.48e−06, q=4.71e−06)
ko00730
0.050
0.055
0.060
0.065
0.070
0.075
Preterm (372)
Term (112)
class

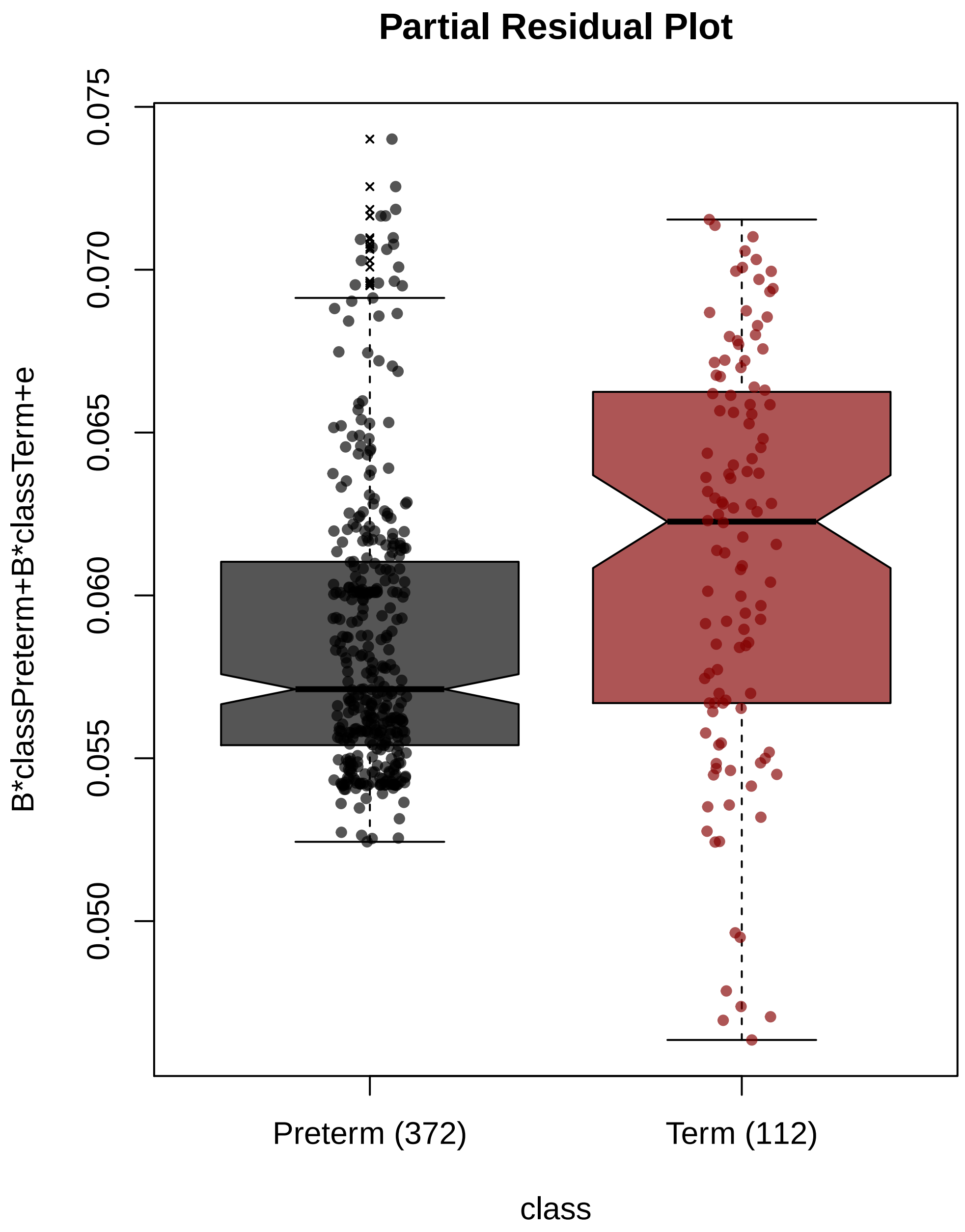

Partial Residual Plot
B*classPreterm+B*classTerm+e
0.050
0.055
0.060
0.065
0.070
0.075
Preterm (372)
Term (112)
class

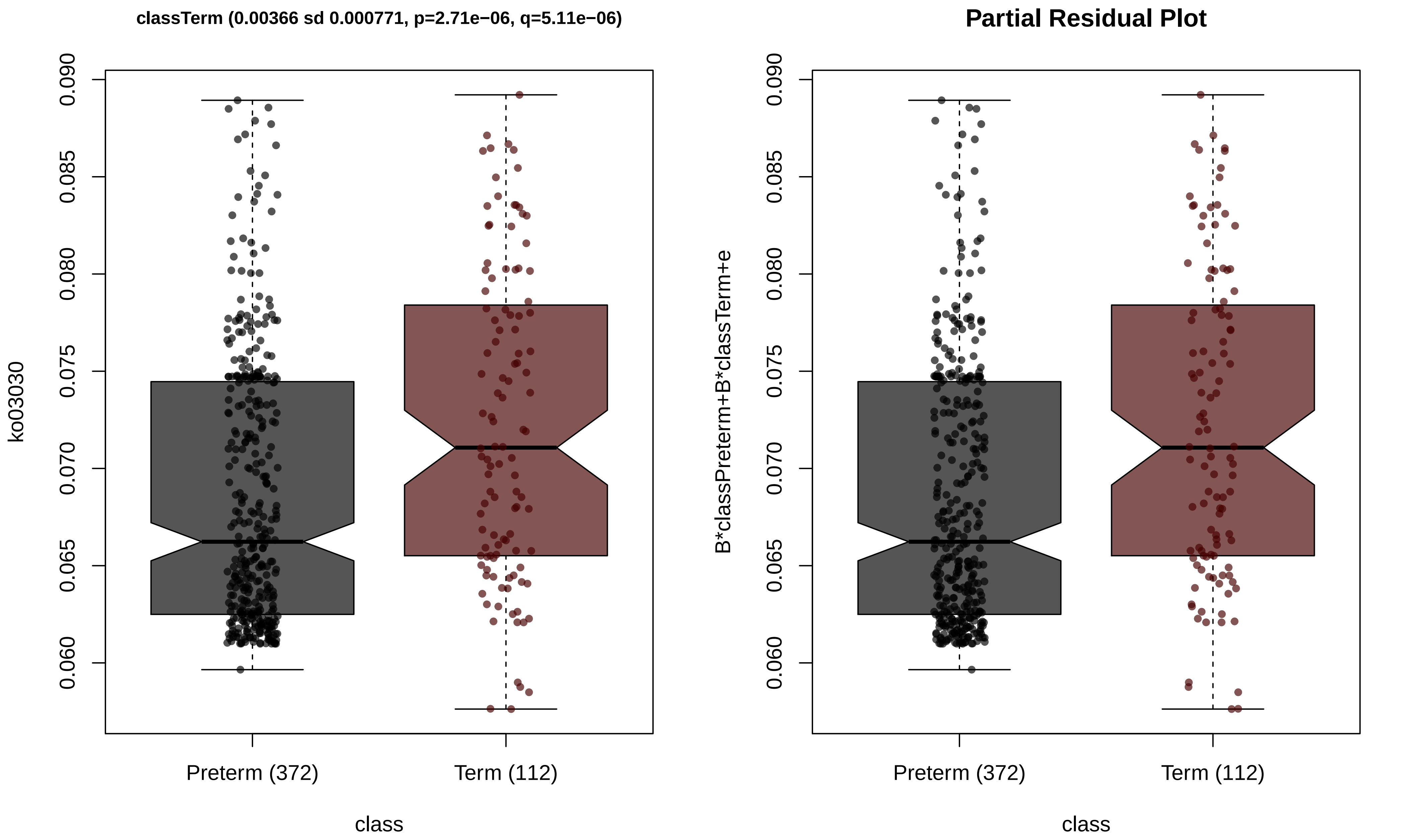

classTerm (0.00366 sd 0.000771, p=2.71e−06, q=5.11e−06)
Partial Residual Plot
ko03030
B*classPreterm+B*classTerm+e
0.060
0.065
0.070
0.075
0.080
0.085
0.090
Preterm (372)
Term (112)
class

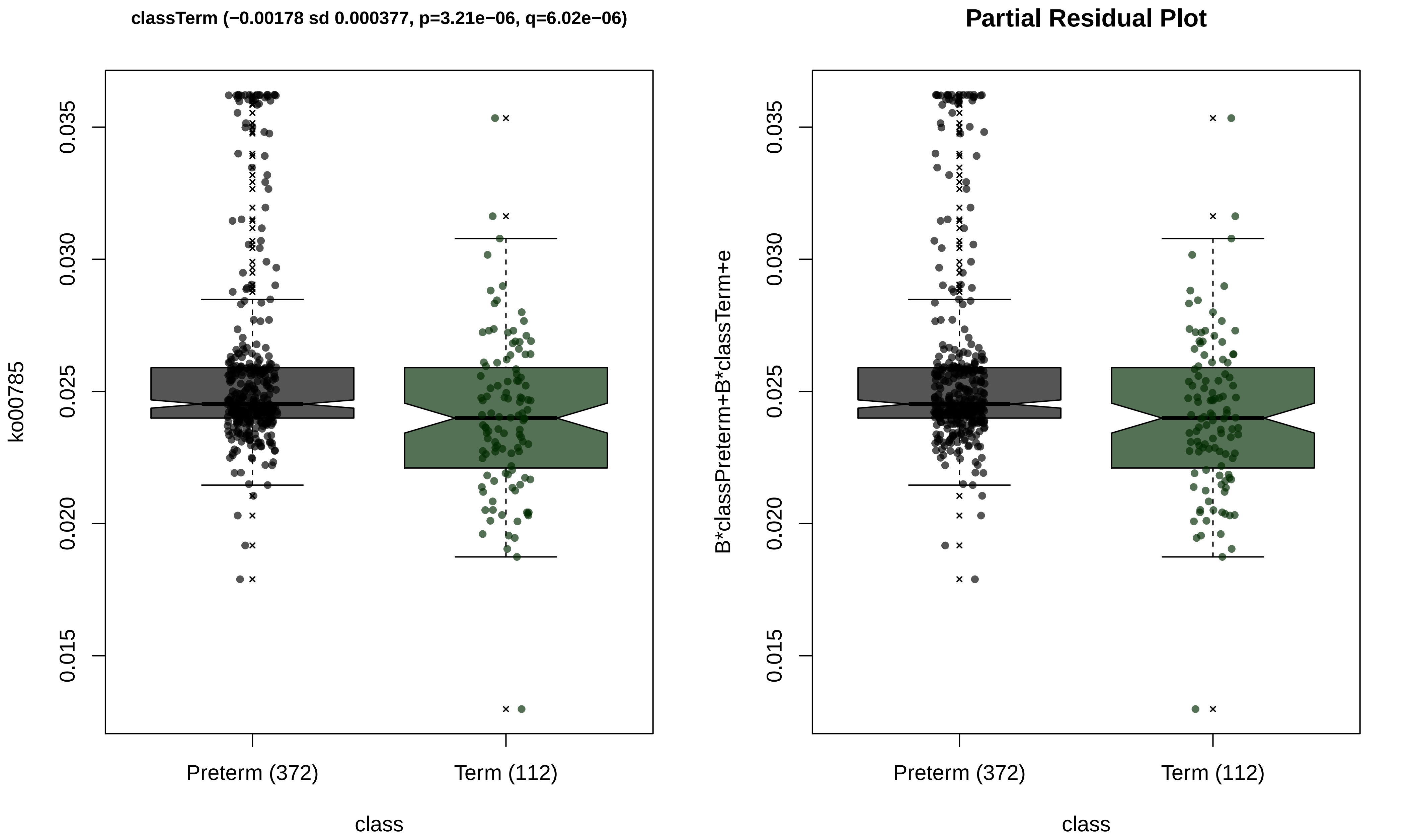

classTerm (−0.00178 sd 0.000377, p=3.21e−06, q=6.02e−06)
Partial Residual Plot
ko00785
B*classPreterm+B*classTerm+e
0.015
0.020
0.025
0.030
0.035
Preterm (372)
Term (112)
class

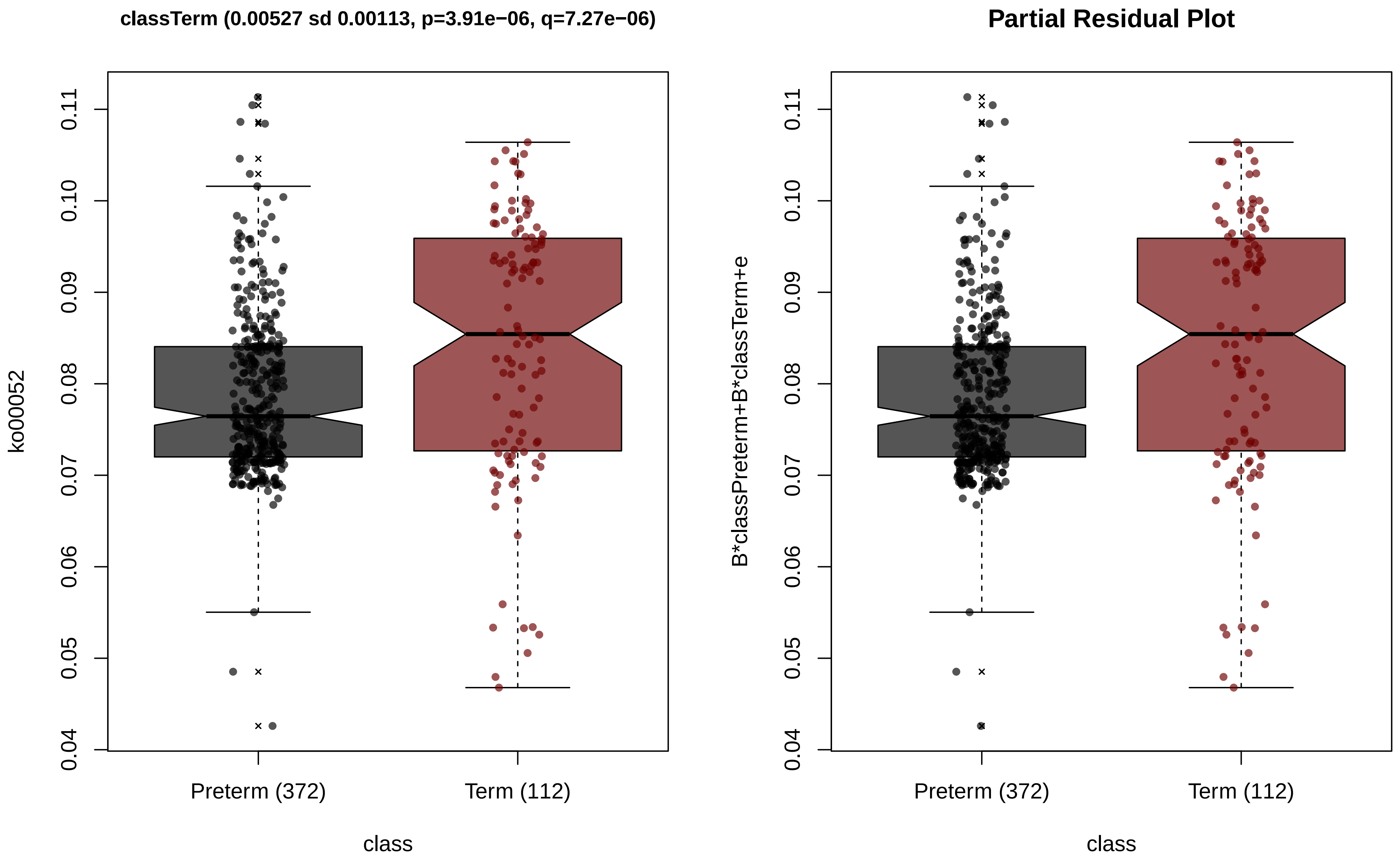

classTerm (0.00527 sd 0.00113, p=3.91e−06, q=7.27e−06)
ko00052
0.04
0.05
0.06
0.07
0.08
0.09
0.10
0.11
Preterm (372)
Term (112)
class
Partial Residual Plot
B*classPreterm+B*classTerm+e
0.04
0.05
0.06
0.07
0.08
0.09
0.10
0.11
Preterm (372)
Term (112)
class

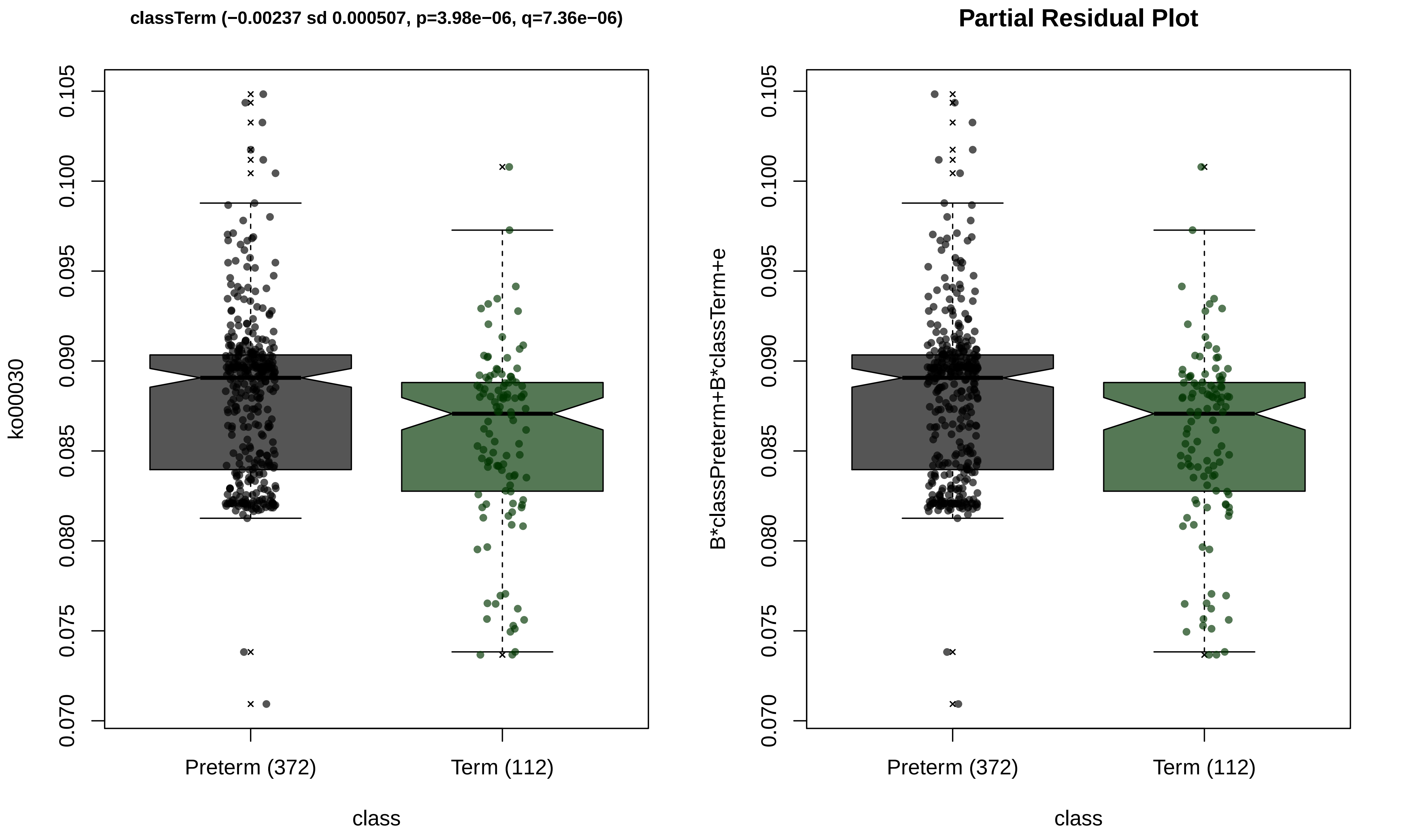

classTerm (−0.00237 sd 0.000507, p=3.98e−06, q=7.36e−06)
ko00030
0.070
0.075
0.080
0.085
0.090
0.095
0.100
0.105
Preterm (372)
Term (112)
class
Partial Residual Plot
B*classPreterm+B*classTerm+e
0.070
0.075
0.080
0.085
0.090
0.095
0.100
0.105
Preterm (372)
Term (112)
class

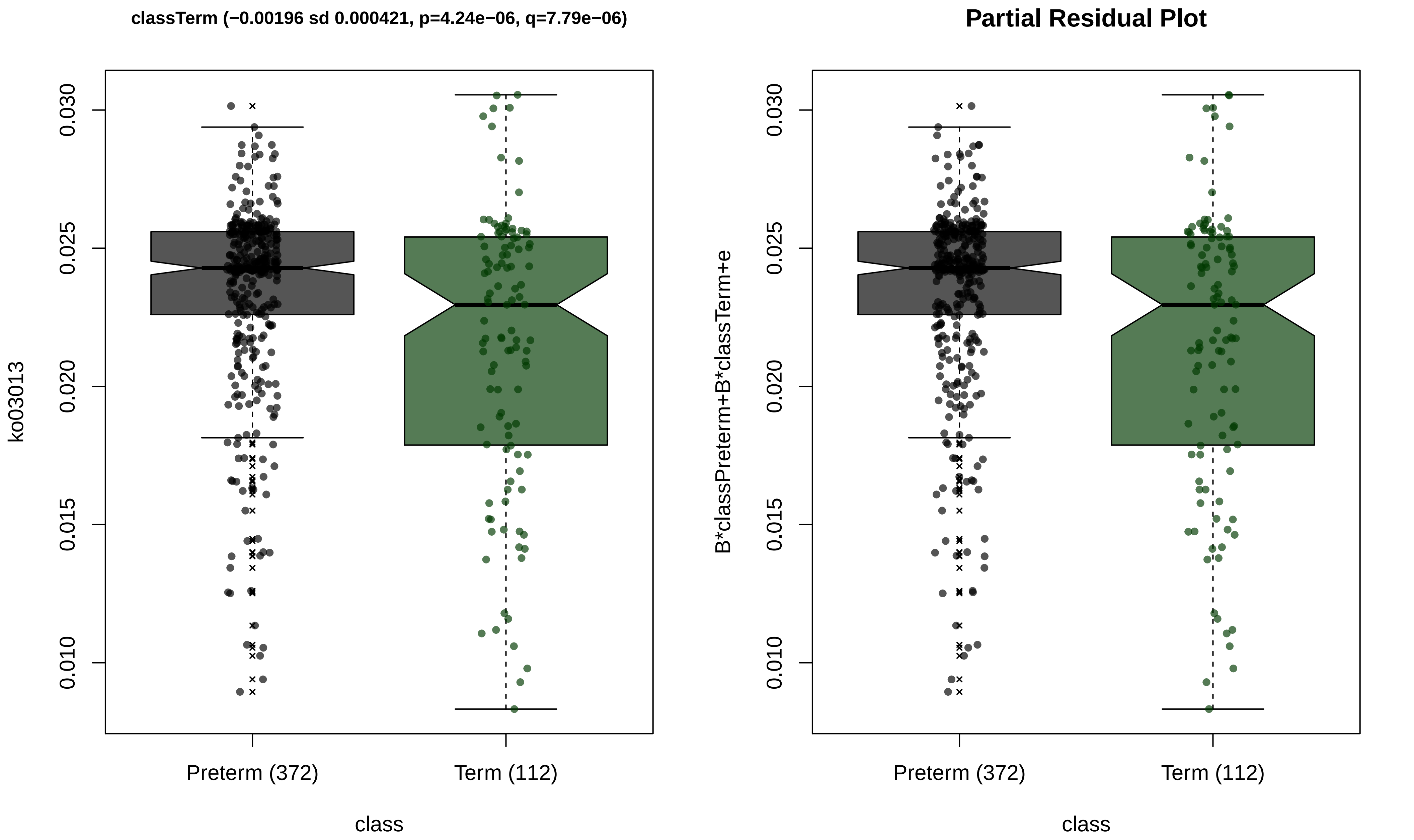

classTerm (−0.00196 sd 0.000421, p=4.24e−06, q=7.79e−06)
Partial Residual Plot
ko03013
B*classPreterm+B*classTerm+e
0.030
0.025
0.020
0.015
0.010
Preterm (372)
Term (112)
class

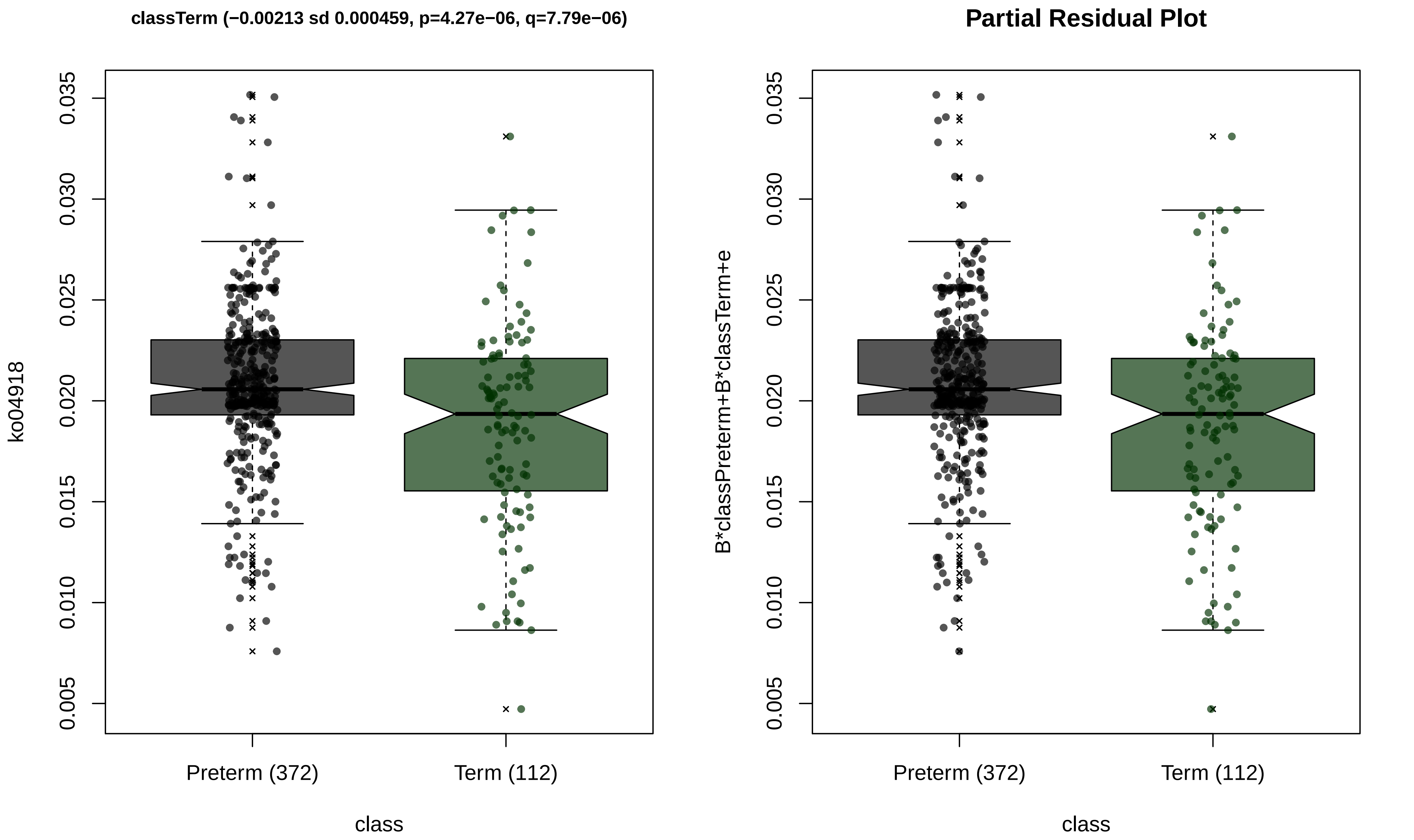
classTerm (−0.00213 sd 0.000459, p=4.27e−06, q=7.79e−06)
Partial Residual Plot
ko04918
B*classPreterm+B*classTerm+e
0.005
0.010
0.015
0.020
0.025
0.030
0.035
Preterm (372)
Term (112)
class

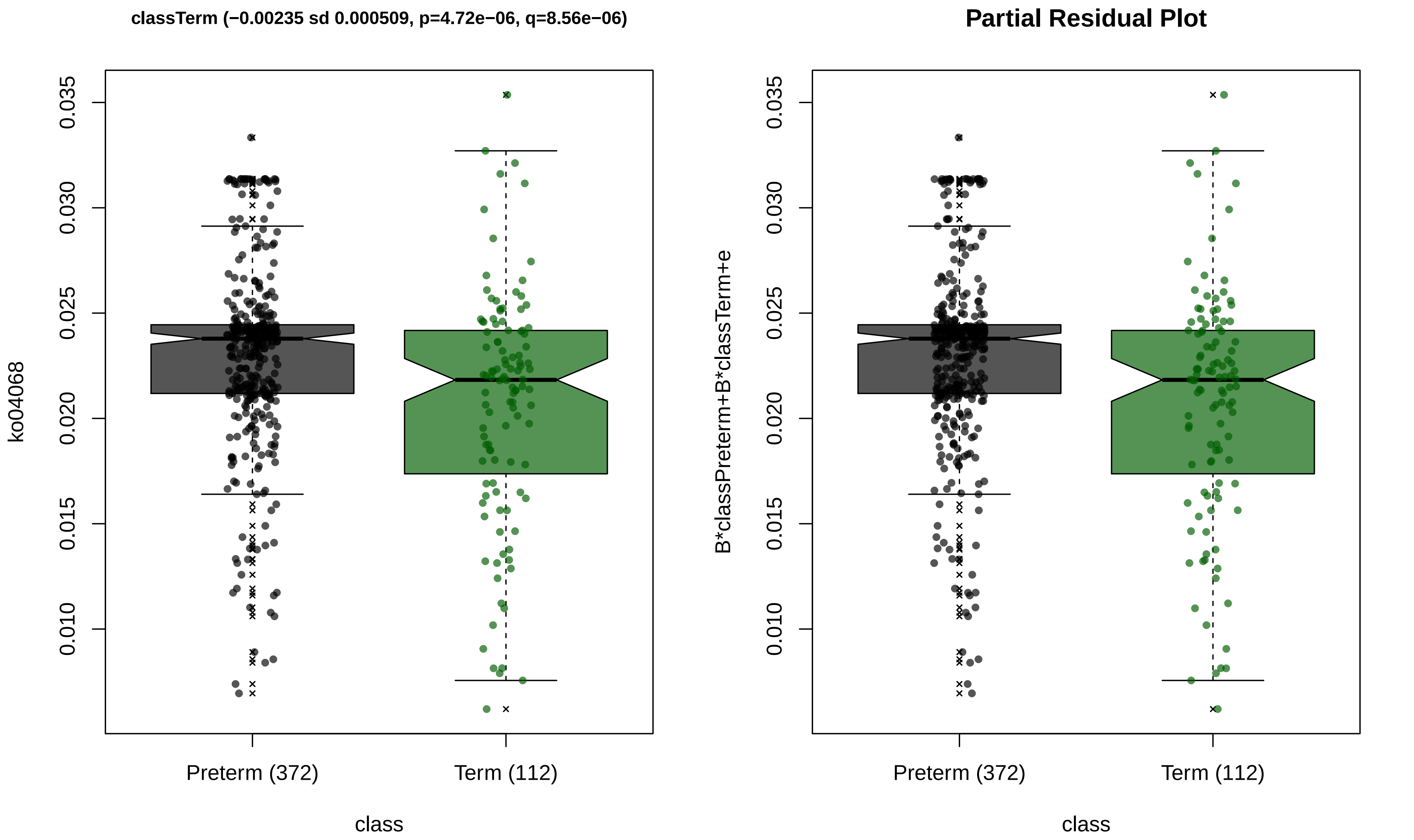

classTerm (−0.00235 sd 0.000509, p=4.72e−06, q=8.56e−06)
ko04068
0.010
0.015
0.020
0.025
0.030
0.035
Preterm (372)
Term (112)
class
Partial Residual Plot
B*classPreterm+B*classTerm+e
0.010
0.015
0.020
0.025
0.030
0.035
Preterm (372)
Term (112)
class

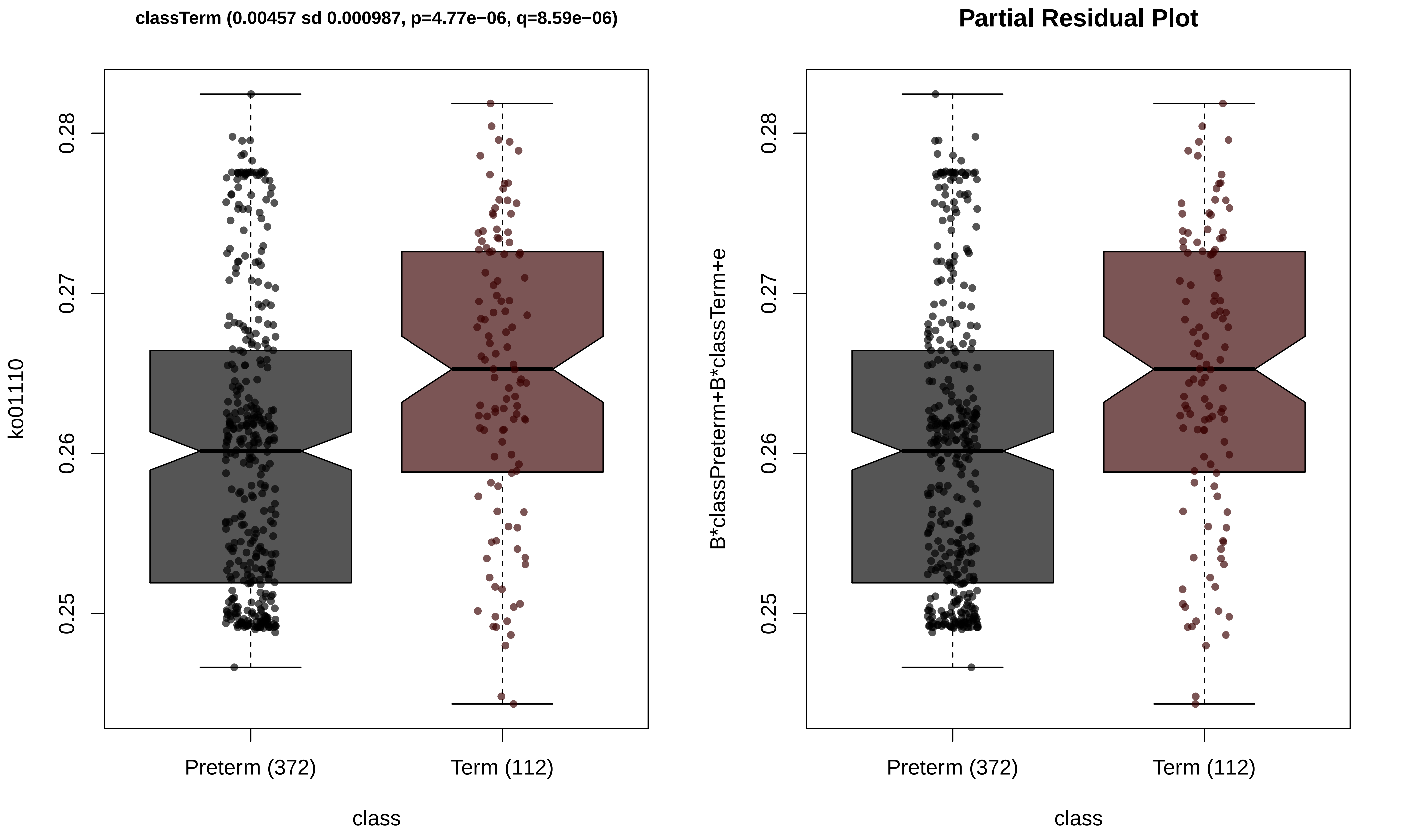

classTerm (0.00457 sd 0.000987, p=4.77e−06, q=8.59e−06)
Partial Residual Plot
ko01110
B*classPreterm+B*classTerm+e
0.25
0.26
0.27
0.28
Preterm (372)
Term (112)
class

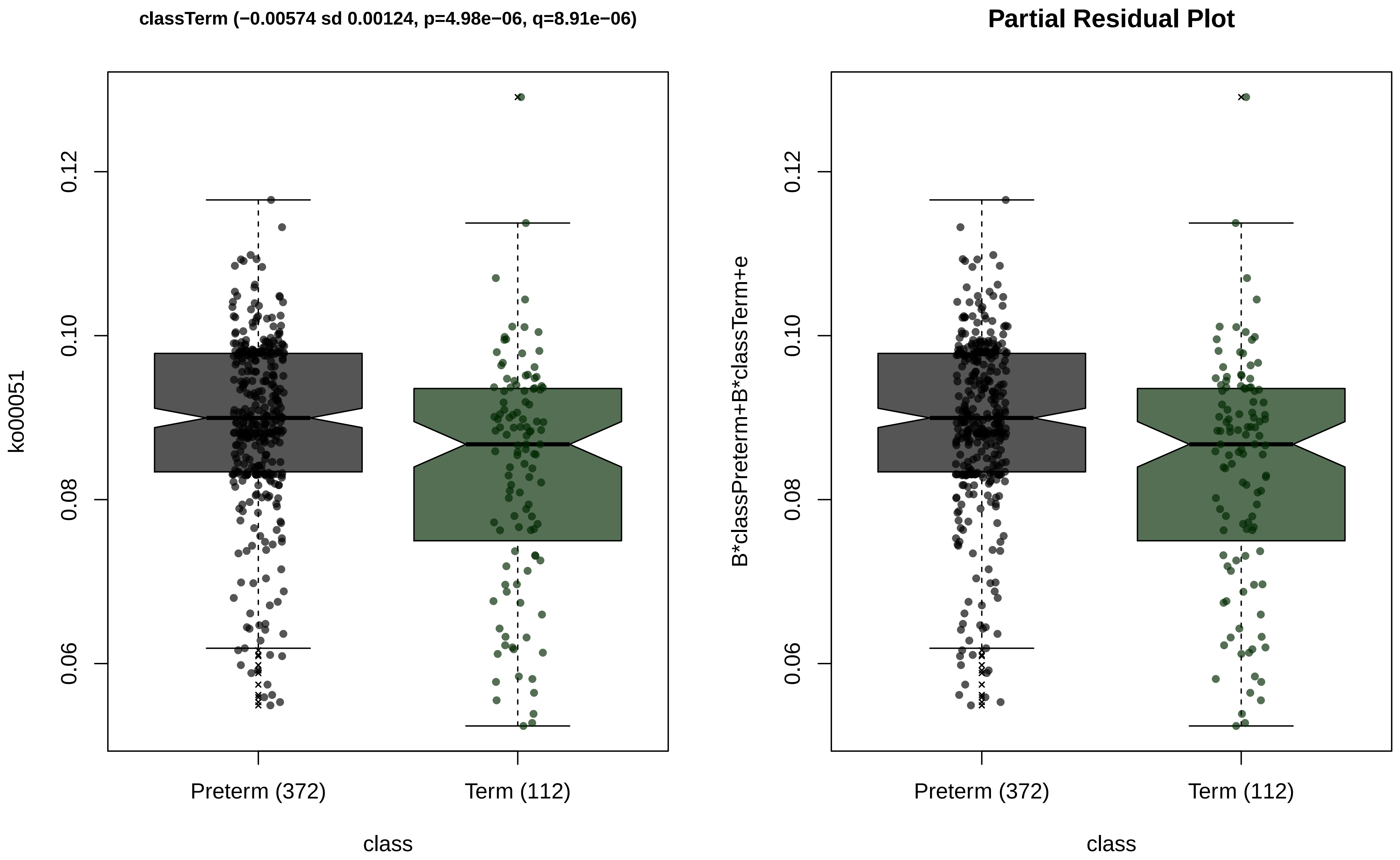
classTerm (−0.00574 sd 0.00124, p=4.98e−06, q=8.91e−06)
ko00051
0.06
0.08
0.10
0.12
Preterm (372)
Term (112)
class
Partial Residual Plot
B*classPreterm+B*classTerm+e
0.06
0.08
0.10
0.12
Preterm (372)
Term (112)
class

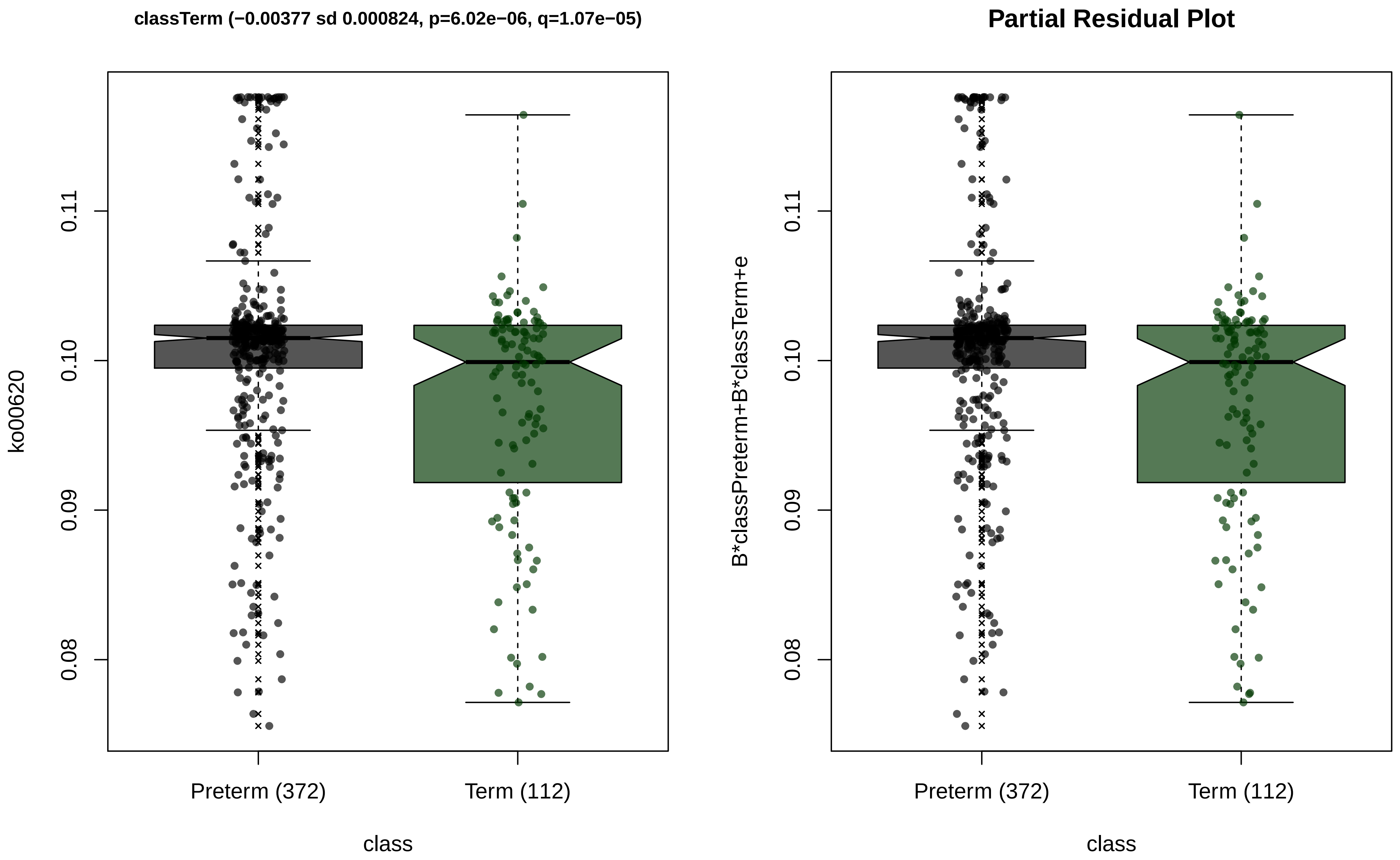
classTerm (−0.00377 sd 0.000824, p=6.02e−06, q=1.07e−05)
Partial Residual Plot
ko00620
B*classPreterm+B*classTerm+e
0.08
0.09
0.10
0.11
Preterm (372)
Term (112)
class

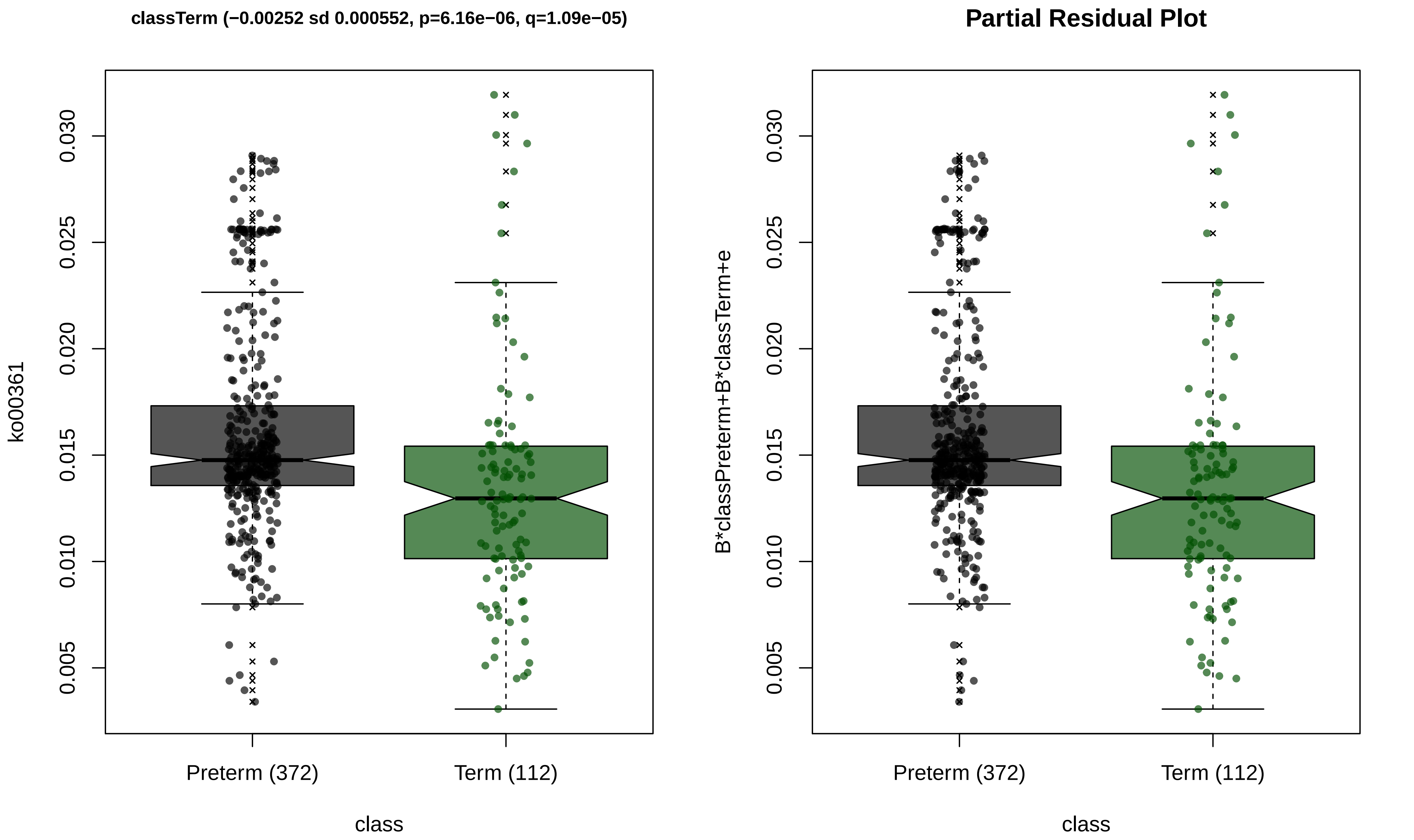

classTerm (−0.00252 sd 0.000552, p=6.16e−06, q=1.09e−05)
Partial Residual Plot
ko00361
B*classPreterm+B*classTerm+e
0.005
0.010
0.015
0.020
0.025
0.030
Preterm (372)
Term (112)
class

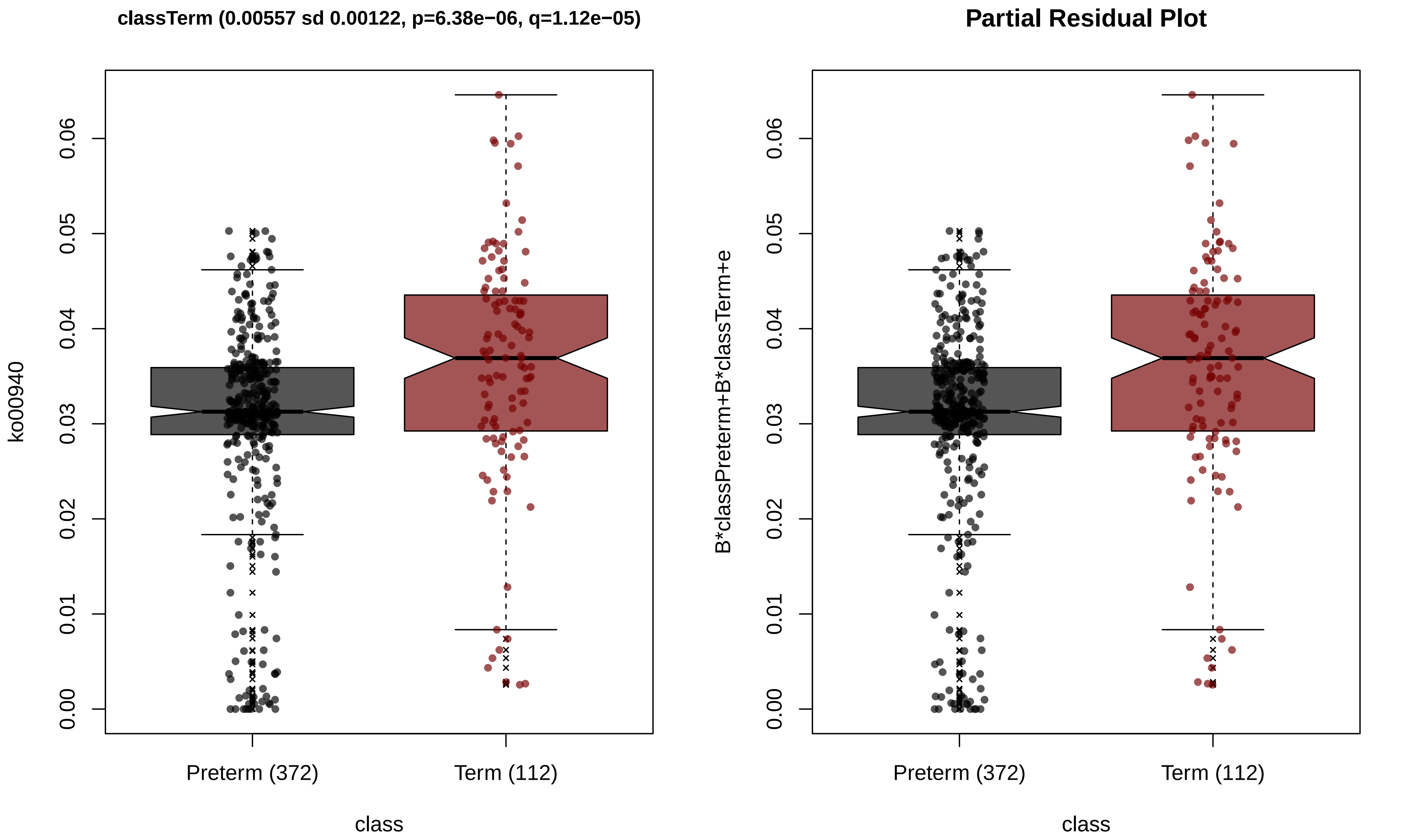

classTerm (0.00557 sd 0.00122, p=6.38e−06, q=1.12e−05)
Partial Residual Plot
ko00940
B*classPreterm+B*classTerm+e
0.00
0.01
0.02
0.03
0.04
0.05
0.06
Preterm (372)
Term (112)
class

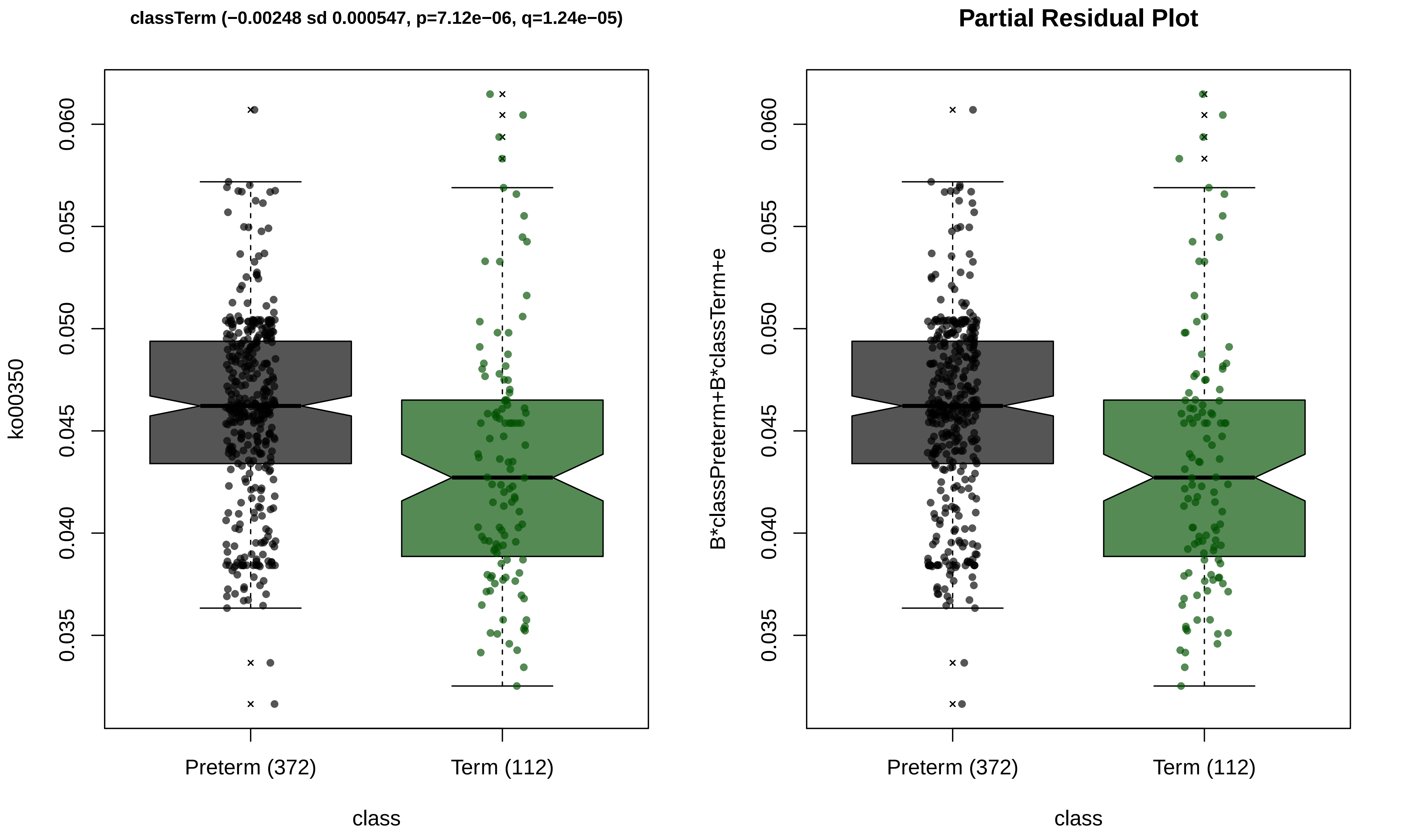
classTerm (−0.00248 sd 0.000547, p=7.12e−06, q=1.24e−05)
Partial Residual Plot
ko00350
B*classPreterm+B*classTerm+e
0.035
0.040
0.045
0.050
0.055
0.060
Preterm (372)
Term (112)
class

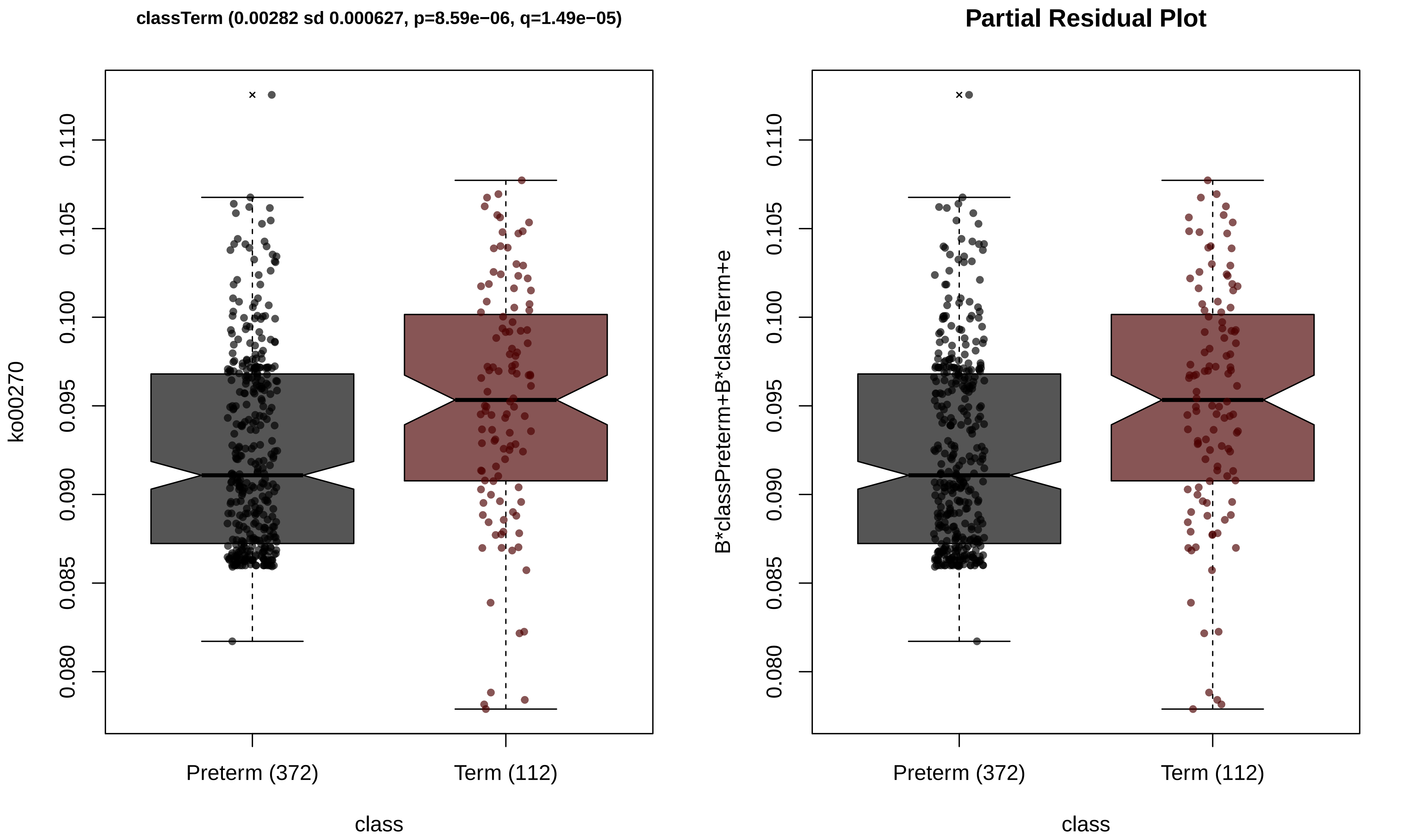

classTerm (0.00282 sd 0.000627, p=8.59e−06, q=1.49e−05)
Partial Residual Plot
ko00270
B*classPreterm+B*classTerm+e
0.080
0.085
0.090
0.095
0.100
0.105
0.110
Preterm (372)
Term (112)
class

classTerm (0.00263 sd 0.00059, p=1.04e−05, q=1.8e−05)

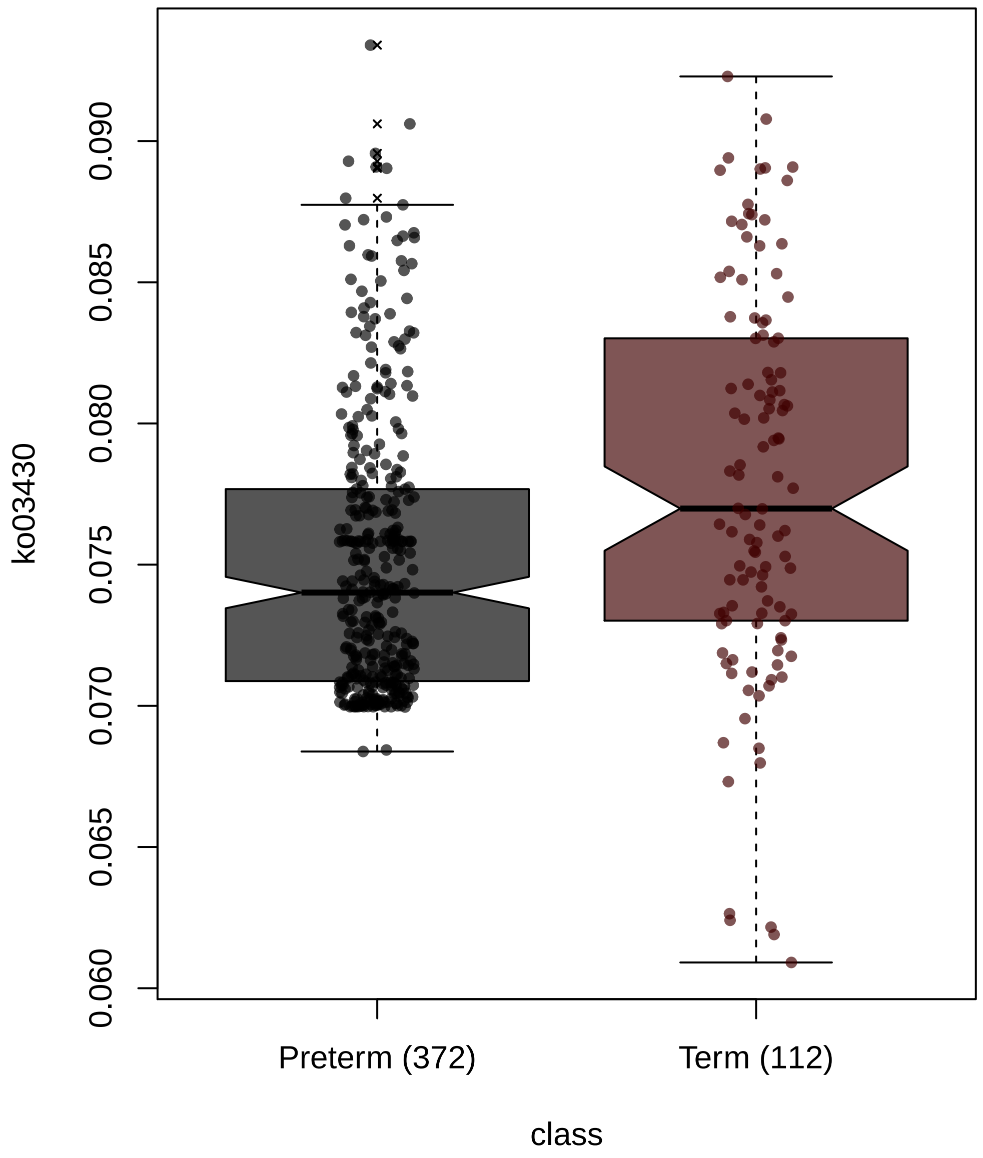

Partial Residual Plot

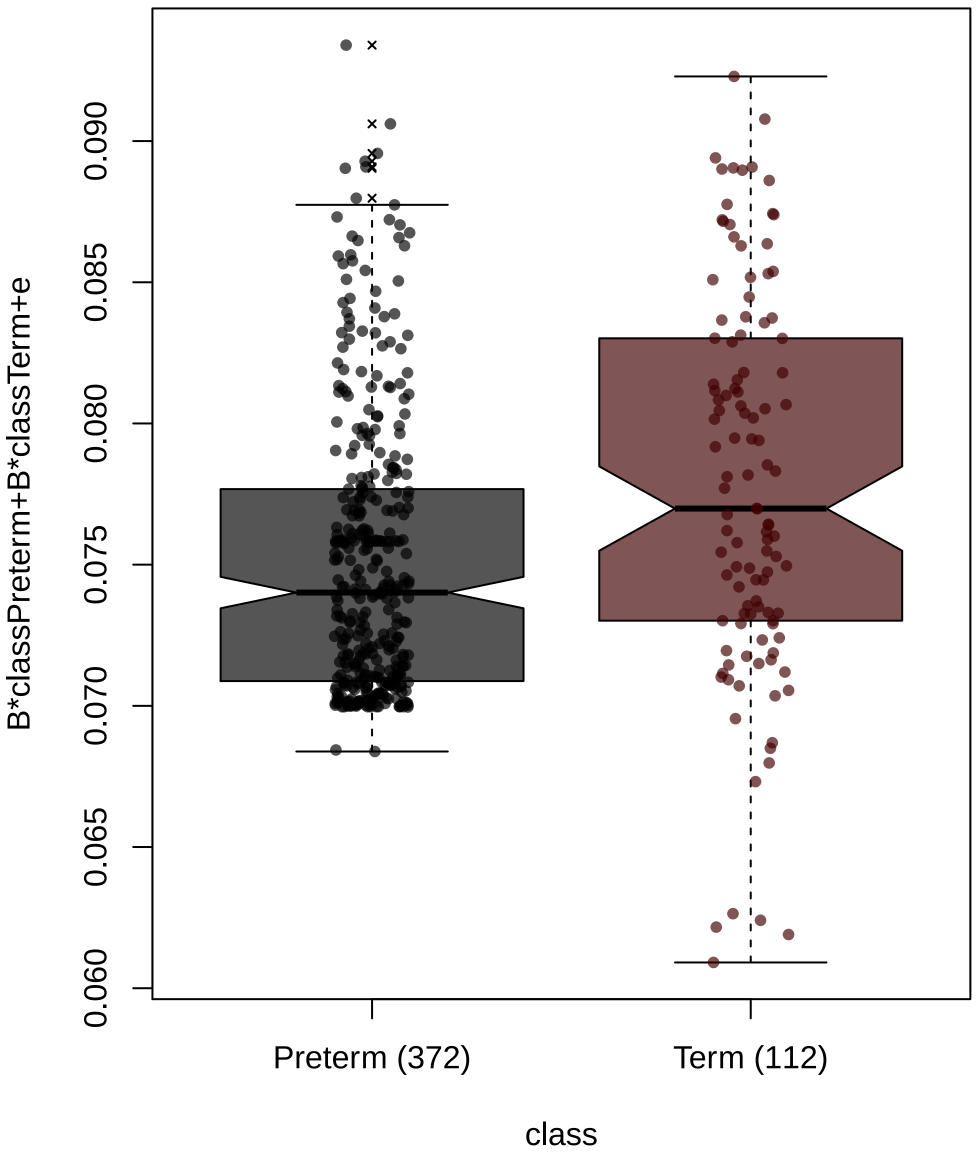

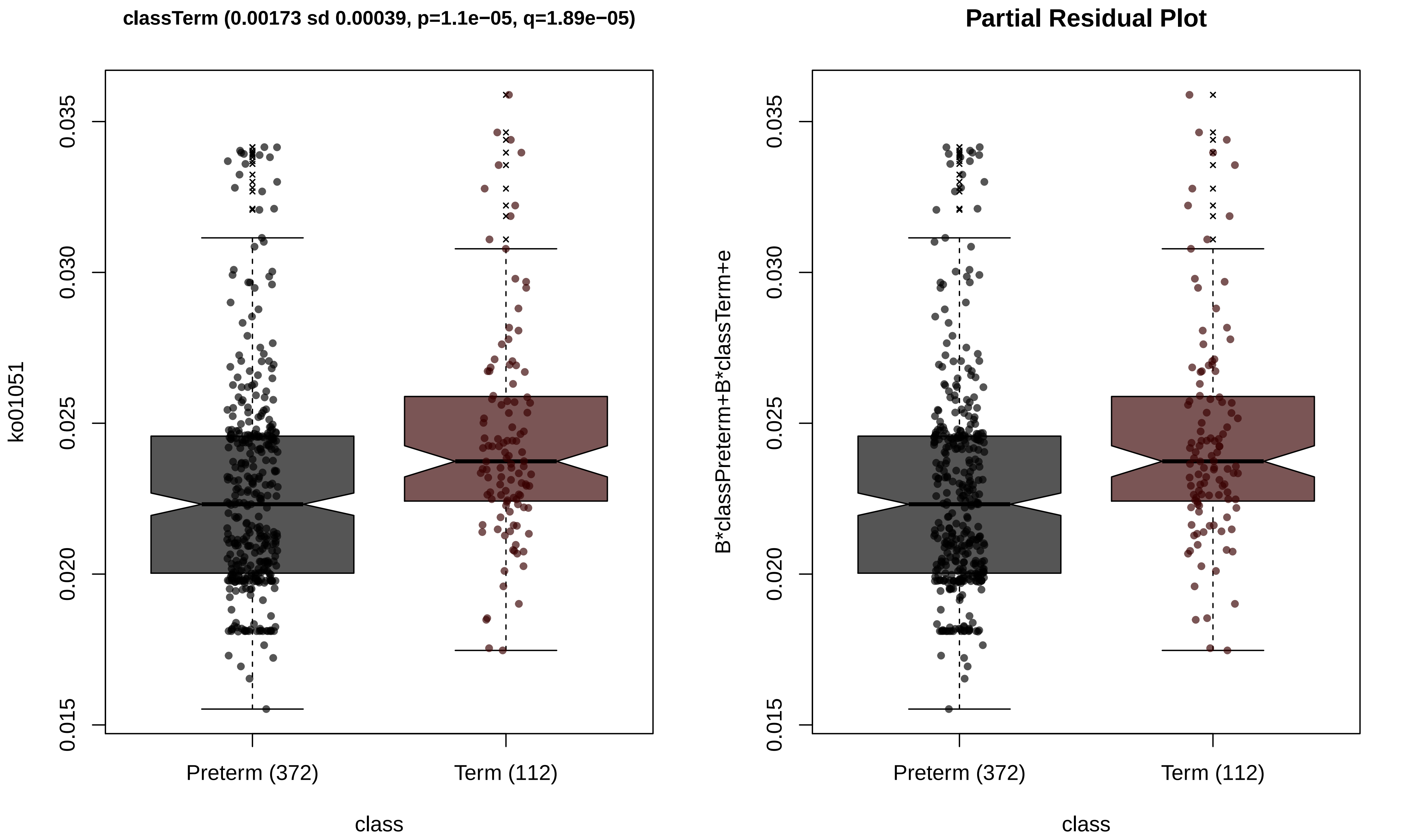
classTerm (0.00173 sd 0.00039, p=1.1e−05, q=1.89e−05)
ko01051
0.035
0.030
0.025
0.020
0.015
Preterm (372)
Term (112)
class
Partial Residual Plot
B*classPreterm+B*classTerm+e
0.035
0.030
0.025
0.020
0.015
Preterm (372)
Term (112)
class

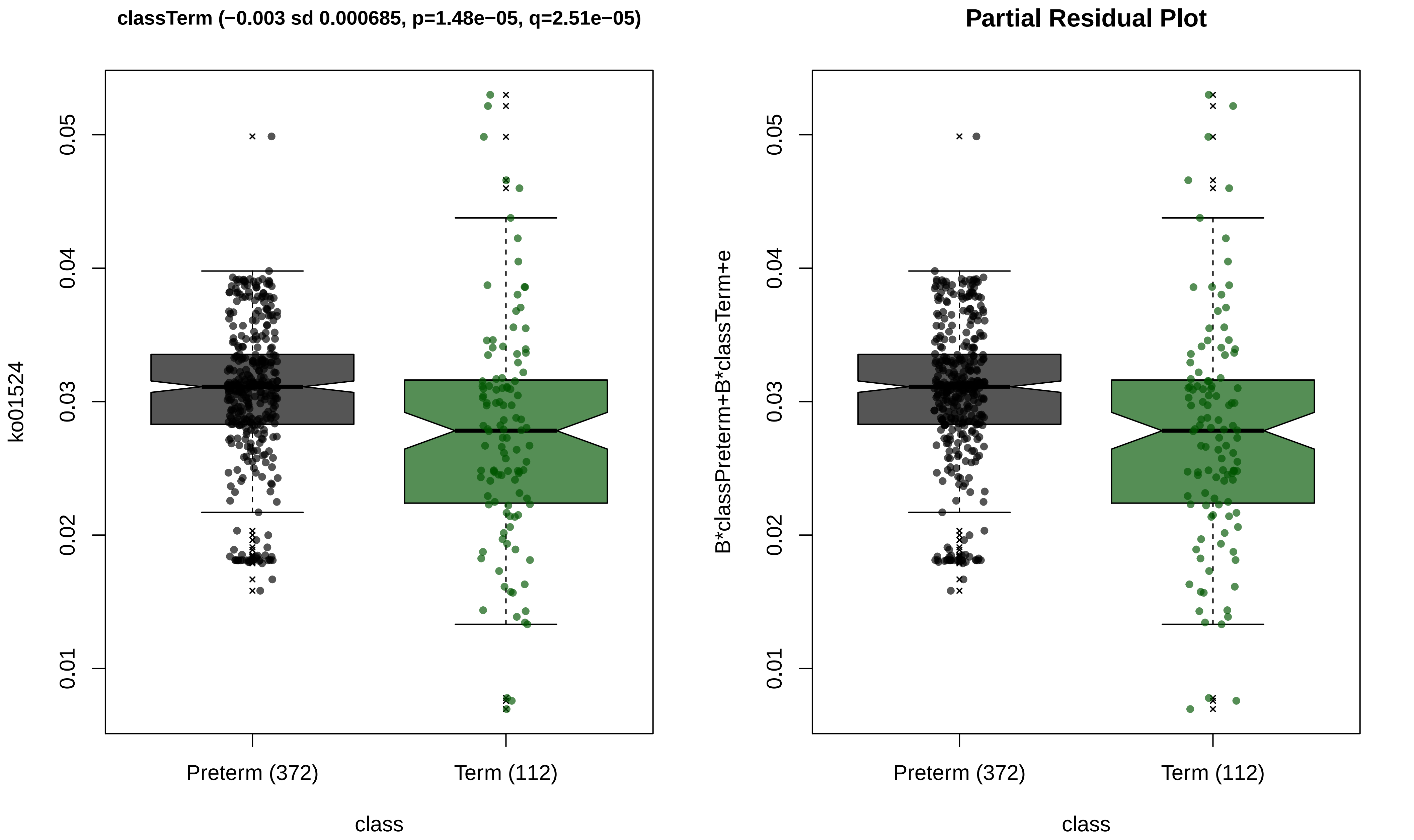

classTerm (−0.003 sd 0.000685, p=1.48e−05, q=2.51e−05)
ko01524
0.01
0.02
0.03
0.04
0.05
Preterm (372)
Term (112)
class
Partial Residual Plot
B*classPreterm+B*classTerm+e
0.01
0.02
0.03
0.04
0.05
Preterm (372)
Term (112)
class

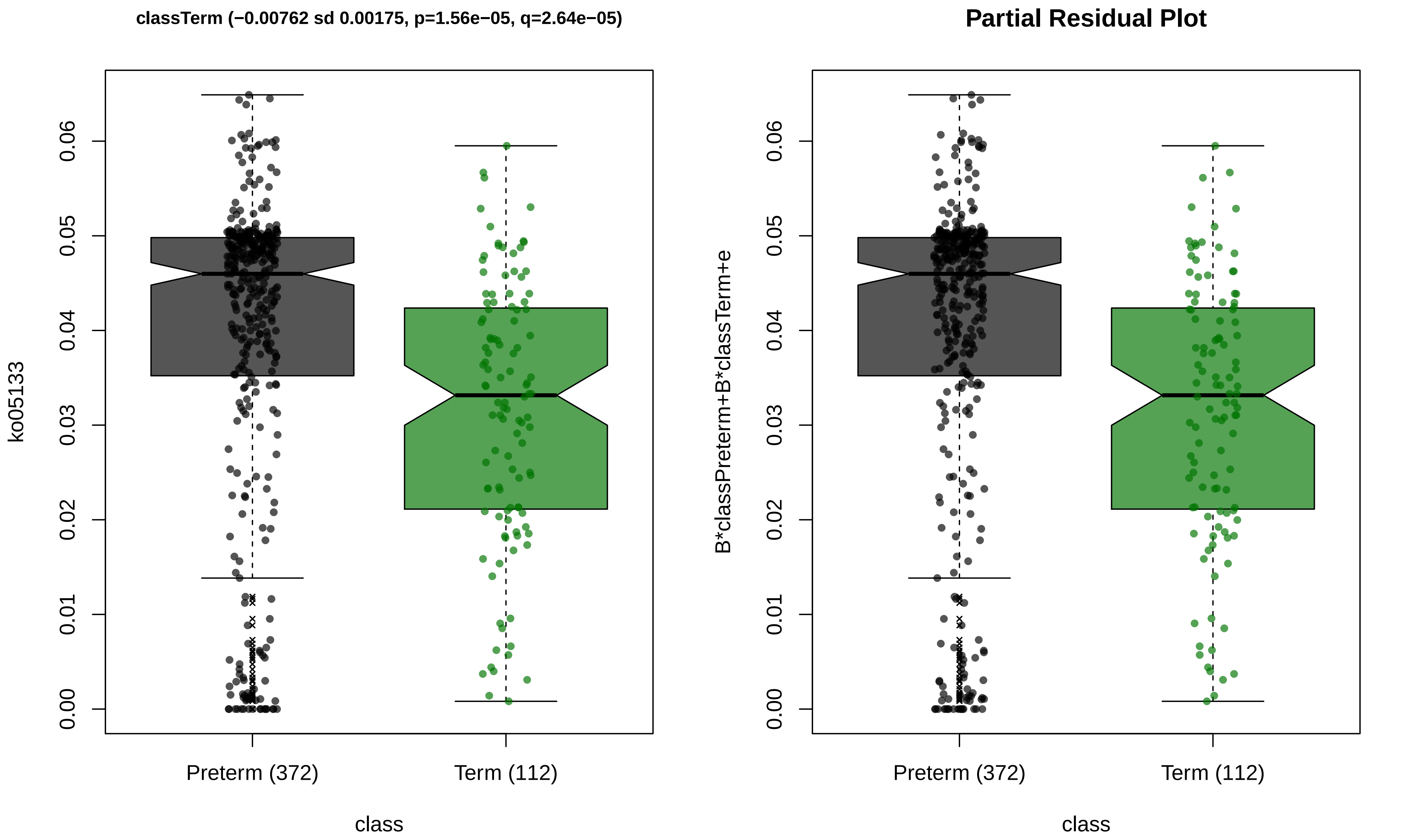

classTerm (−0.00762 sd 0.00175, p=1.56e−05, q=2.64e−05)
Partial Residual Plot
ko05133
B*classPreterm+B*classTerm+e
0.00
0.01
0.02
0.03
0.04
0.05
0.06
Preterm (372)
Term (112)
class

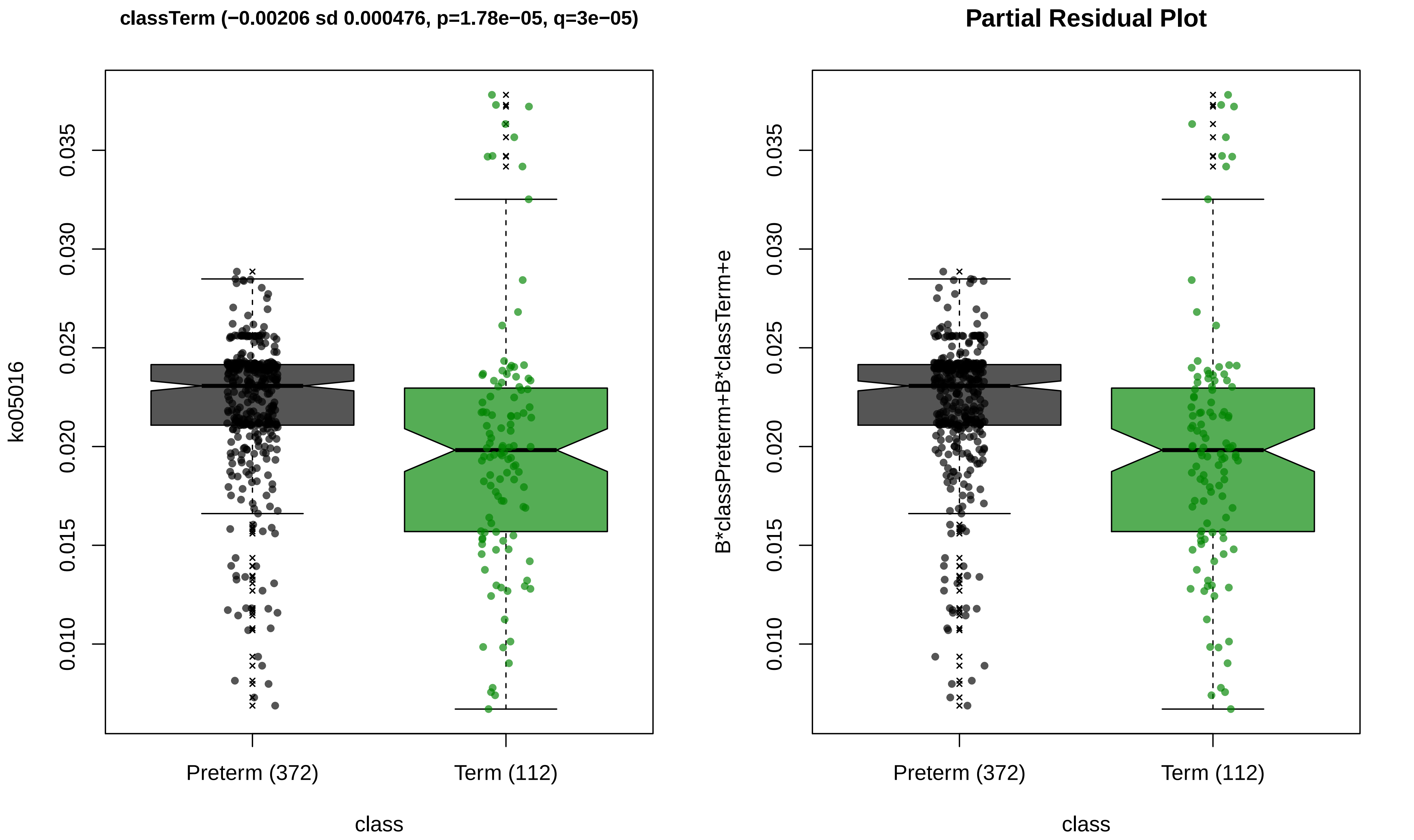

classTerm (−0.00206 sd 0.000476, p=1.78e−05, q=3e−05)
Partial Residual Plot
ko05016
B*classPreterm+B*classTerm+e
0.010
0.015
0.020
0.025
0.030
0.035
Preterm (372)
Term (112)
class

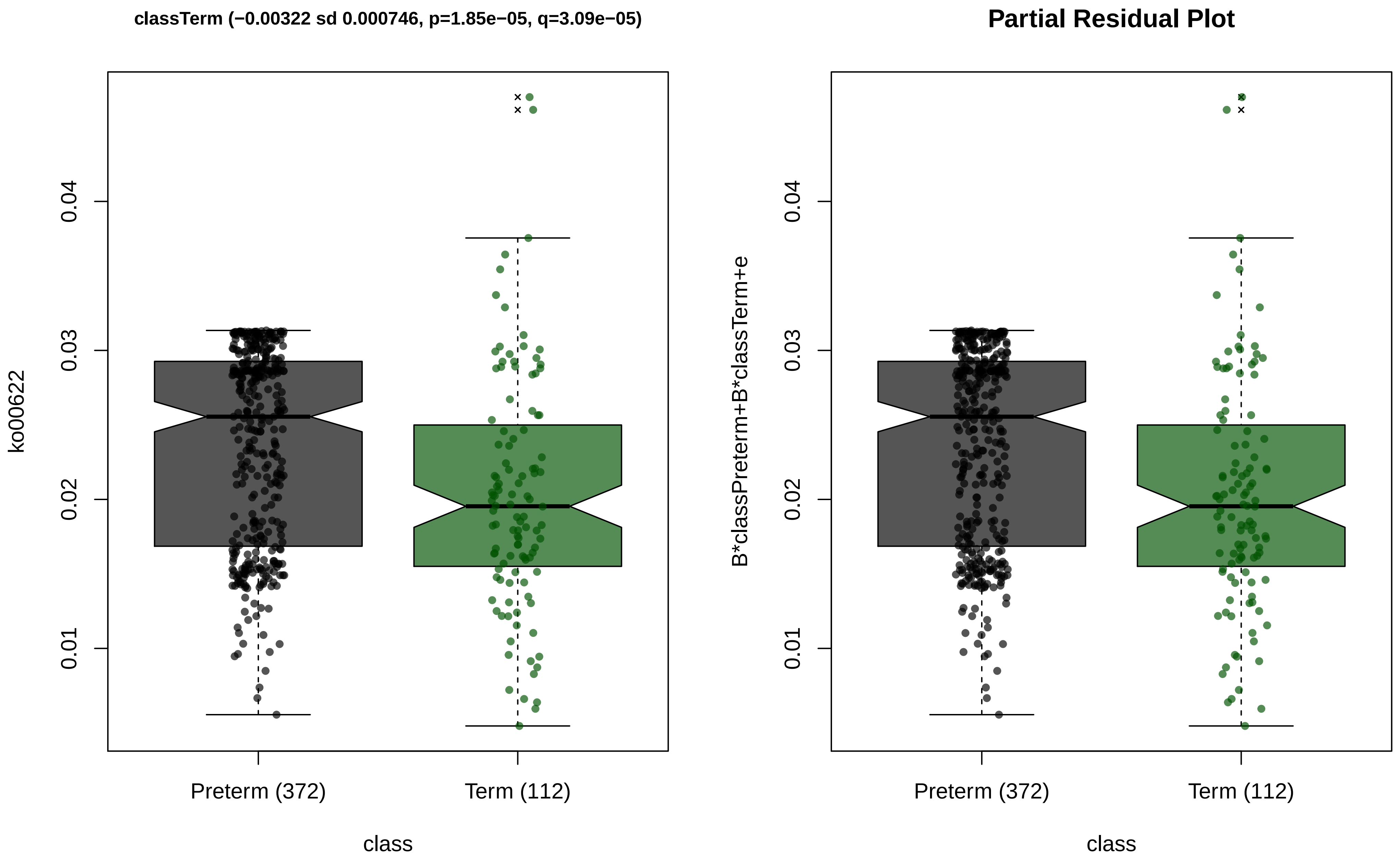
classTerm (−0.00322 sd 0.000746, p=1.85e−05, q=3.09e−05)
Partial Residual Plot
ko00622
B*classPreterm+B*classTerm+e
0.01
0.02
0.03
0.04
Preterm (372)
Term (112)
class

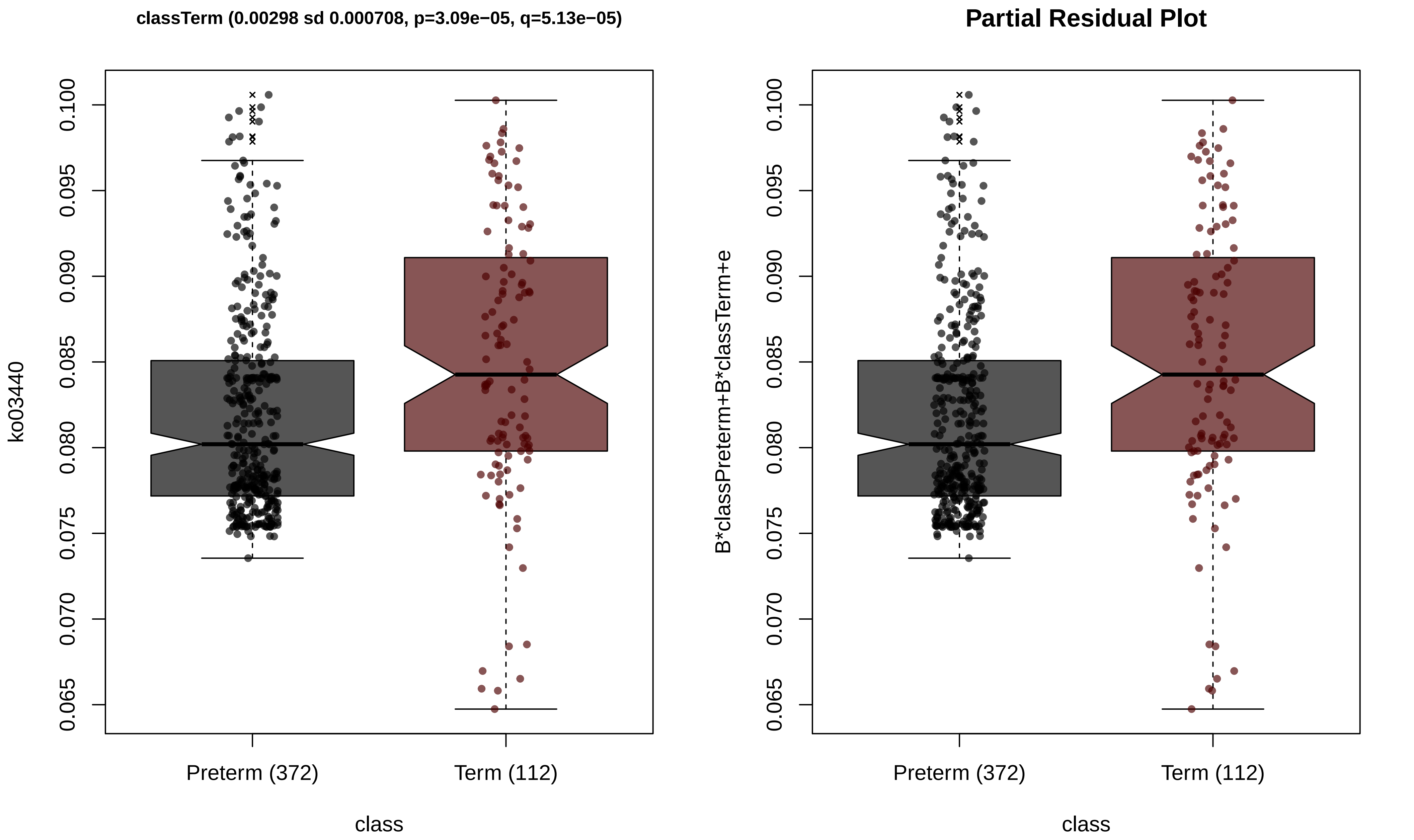

classTerm (0.00298 sd 0.000708, p=3.09e−05, q=5.13e−05)
Partial Residual Plot
ko03440
B*classPreterm+B*classTerm+e
0.065
0.070
0.075
0.080
0.085
0.090
0.095
0.100
Preterm (372)
Term (112)
class

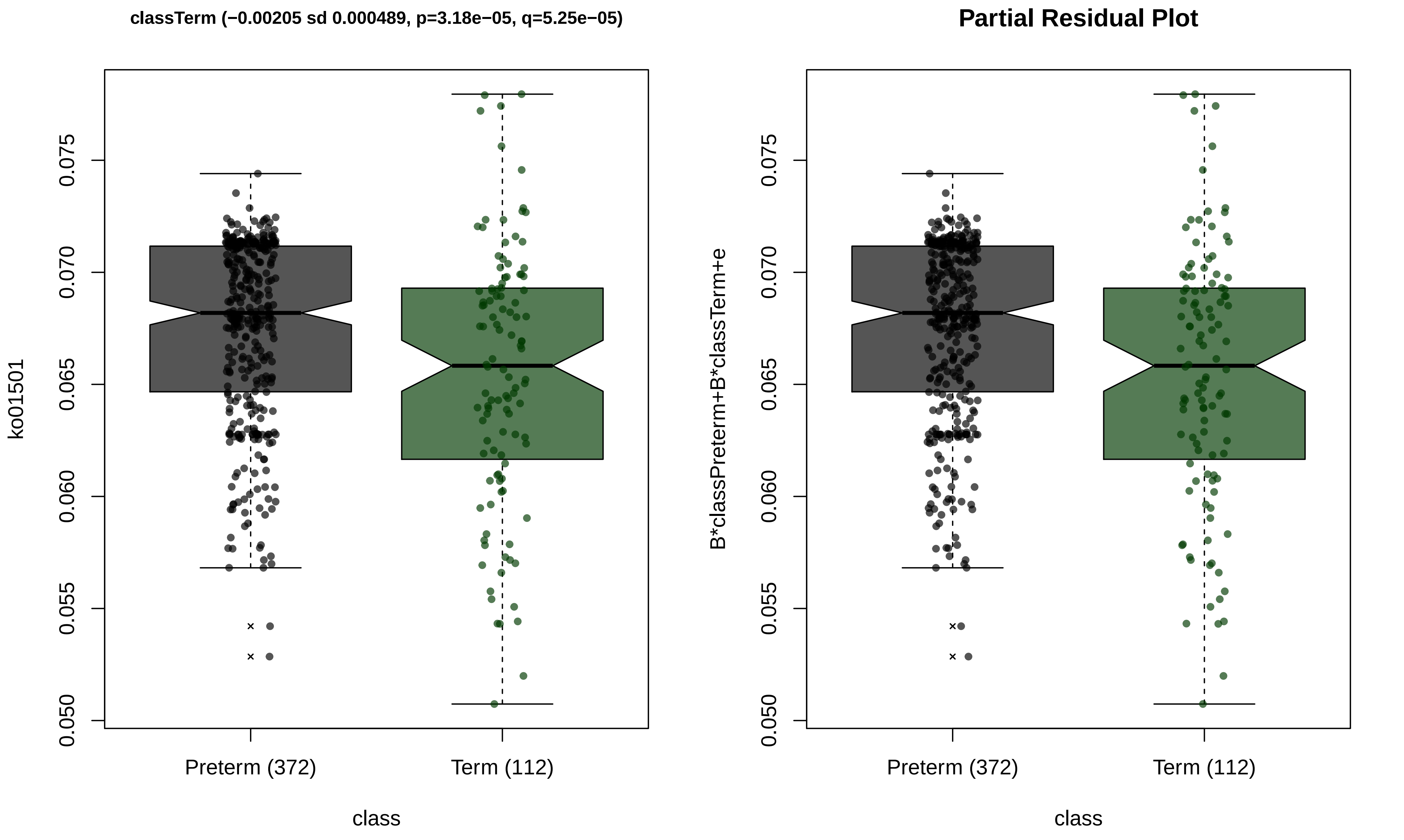
classTerm (−0.00205 sd 0.000489, p=3.18e−05, q=5.25e−05)
Partial Residual Plot
ko01501
B*classPreterm+B*classTerm+e
0.050
0.055
0.060
0.065
0.070
0.075
Preterm (372)
Term (112)
class

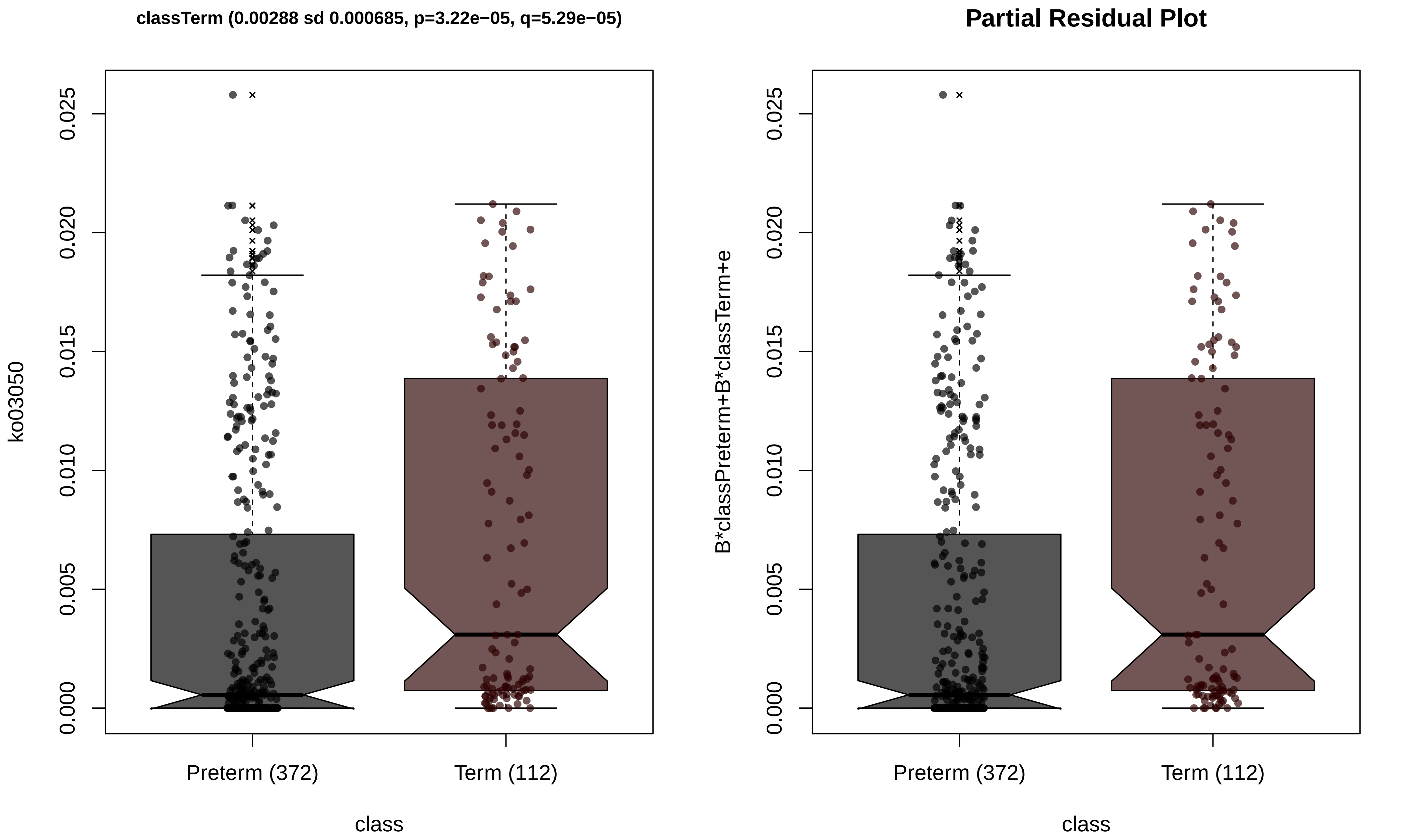

classTerm (0.00288 sd 0.000685, p=3.22e−05, q=5.29e−05)
Partial Residual Plot
ko03050
B*classPreterm+B*classTerm+e
0.000
0.005
0.010
0.015
0.020
0.025
Preterm (372)
Term (112)
class

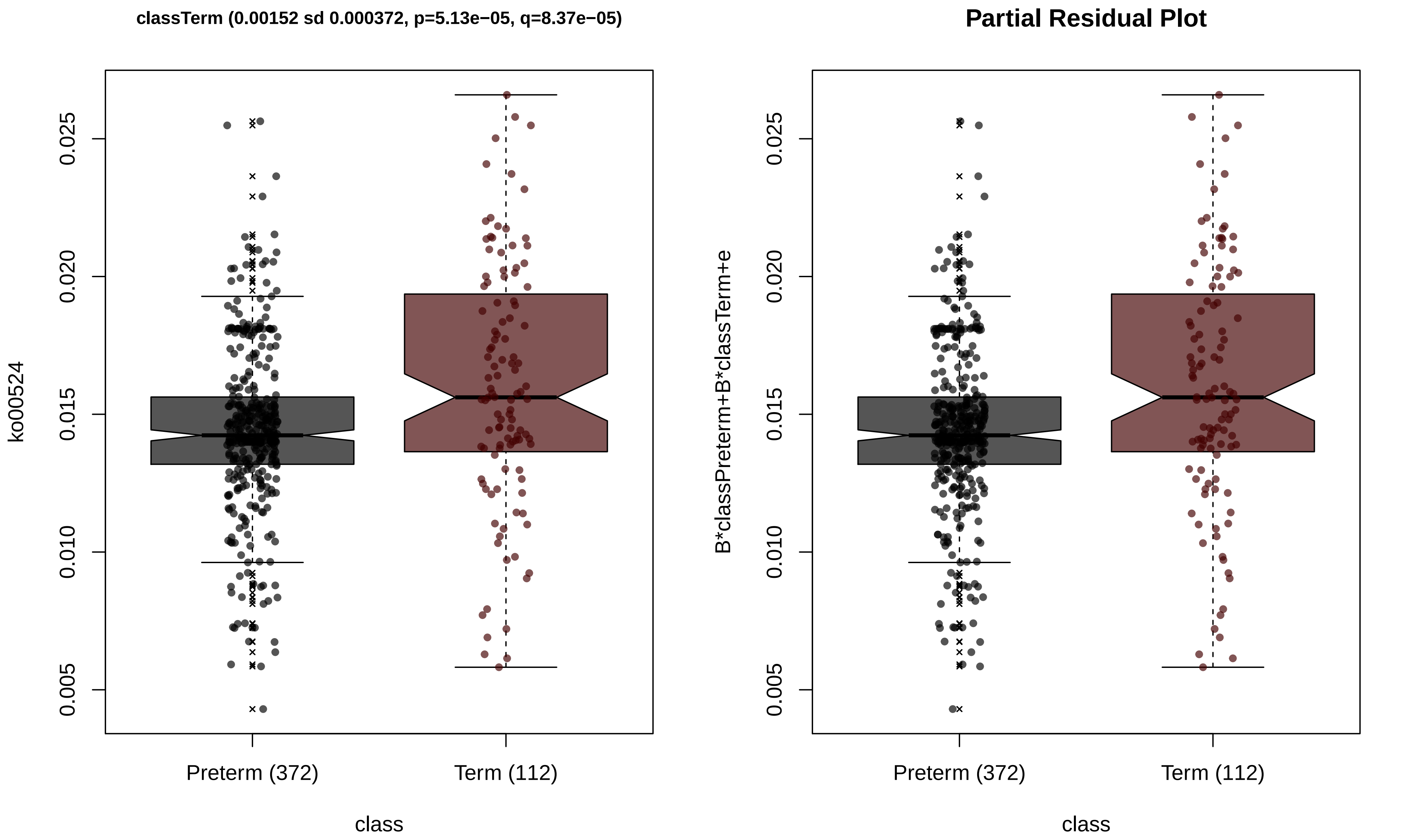

classTerm (0.00152 sd 0.000372, p=5.13e−05, q=8.37e−05)
Partial Residual Plot
ko00524
B*classPreterm+B*classTerm+e
0.005
0.010
0.015
0.020
0.025
Preterm (372)
Term (112)
class

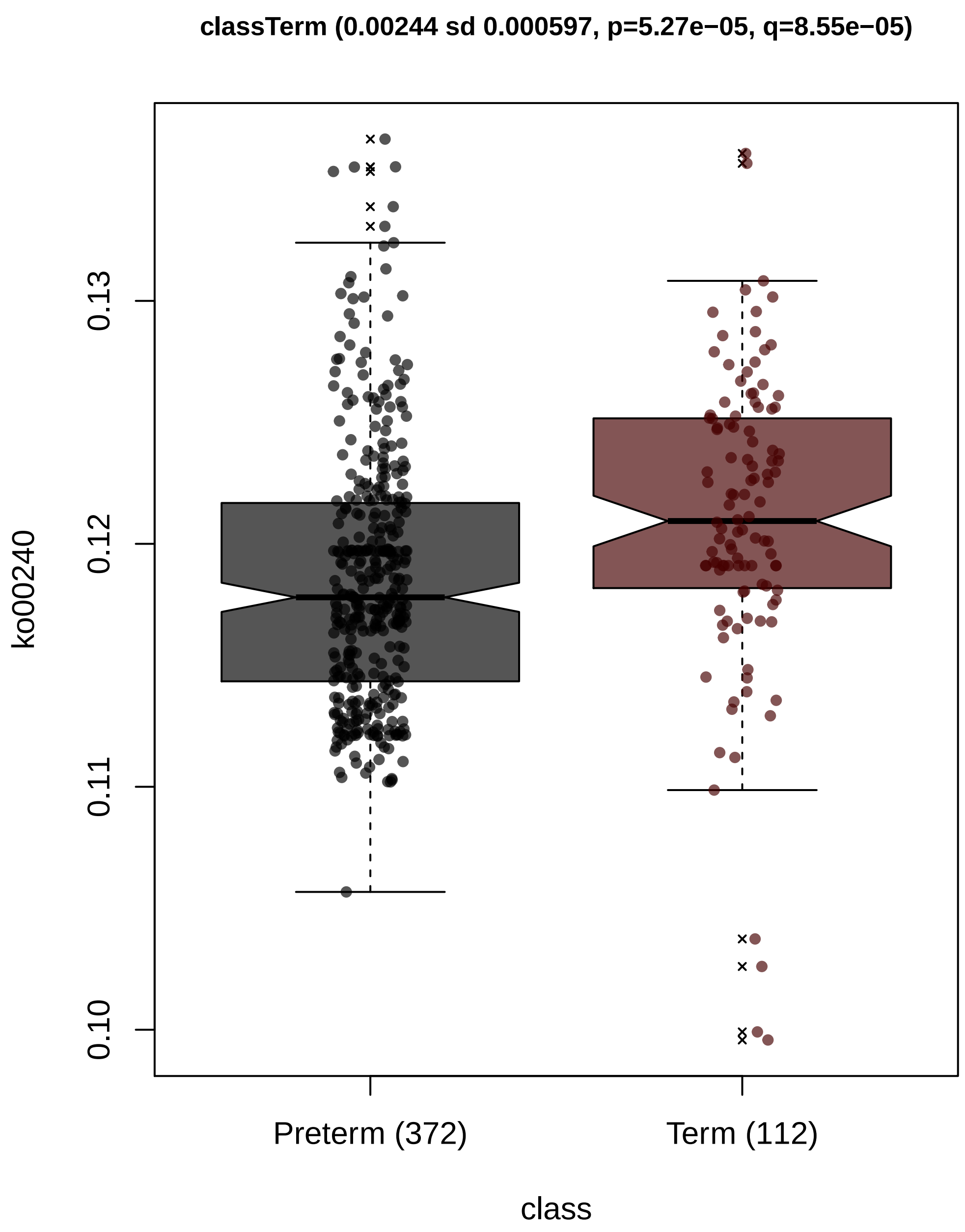

classTerm (0.00244 sd 0.000597, p=5.27e−05, q=8.55e−05)
ko00240
0.10
0.11
0.12
0.13
Preterm (372)
Term (112)
class

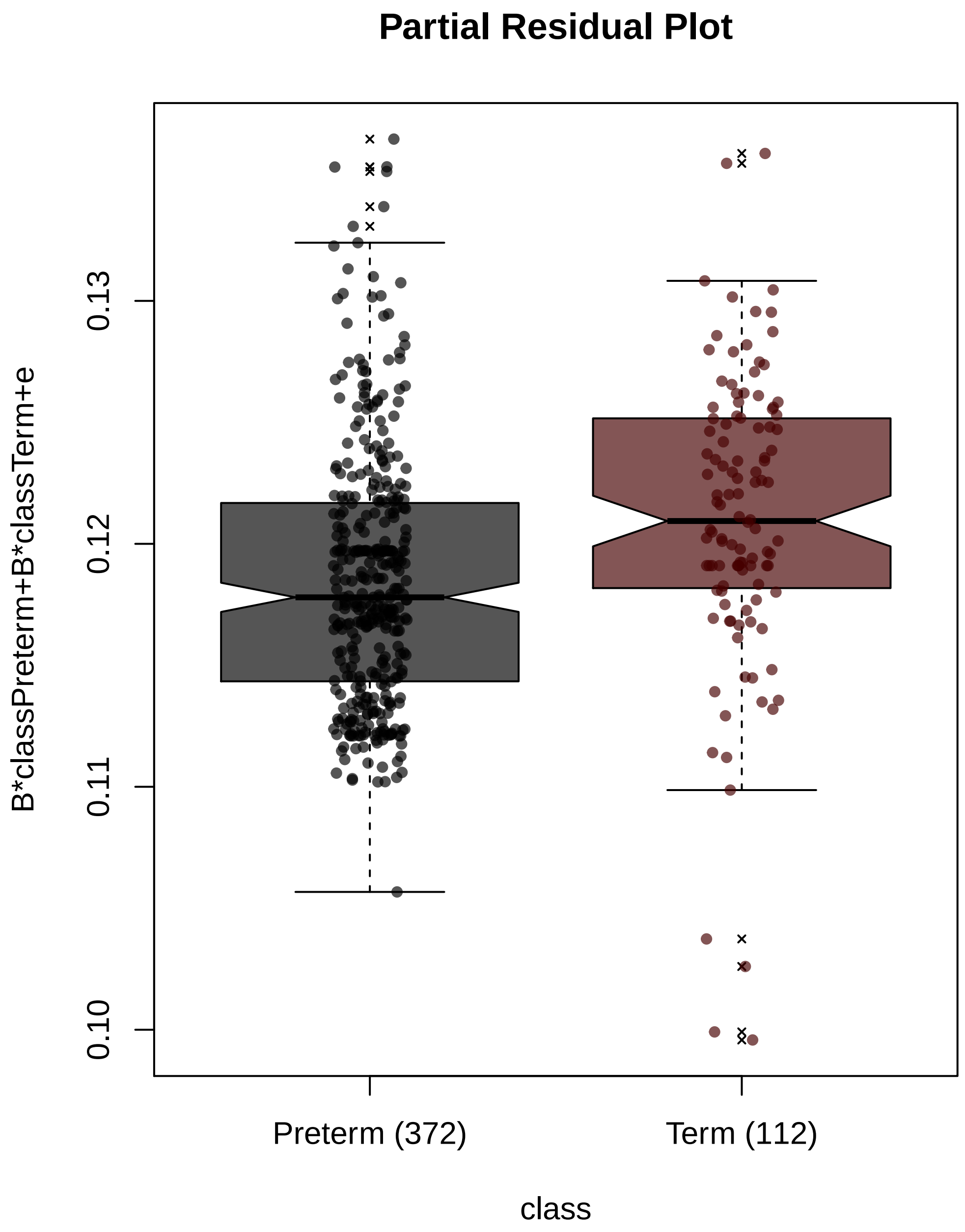

Partial Residual Plot
B*classPreterm+B*classTerm+e
0.10
0.11
0.12
0.13
Preterm (372)
Term (112)
class

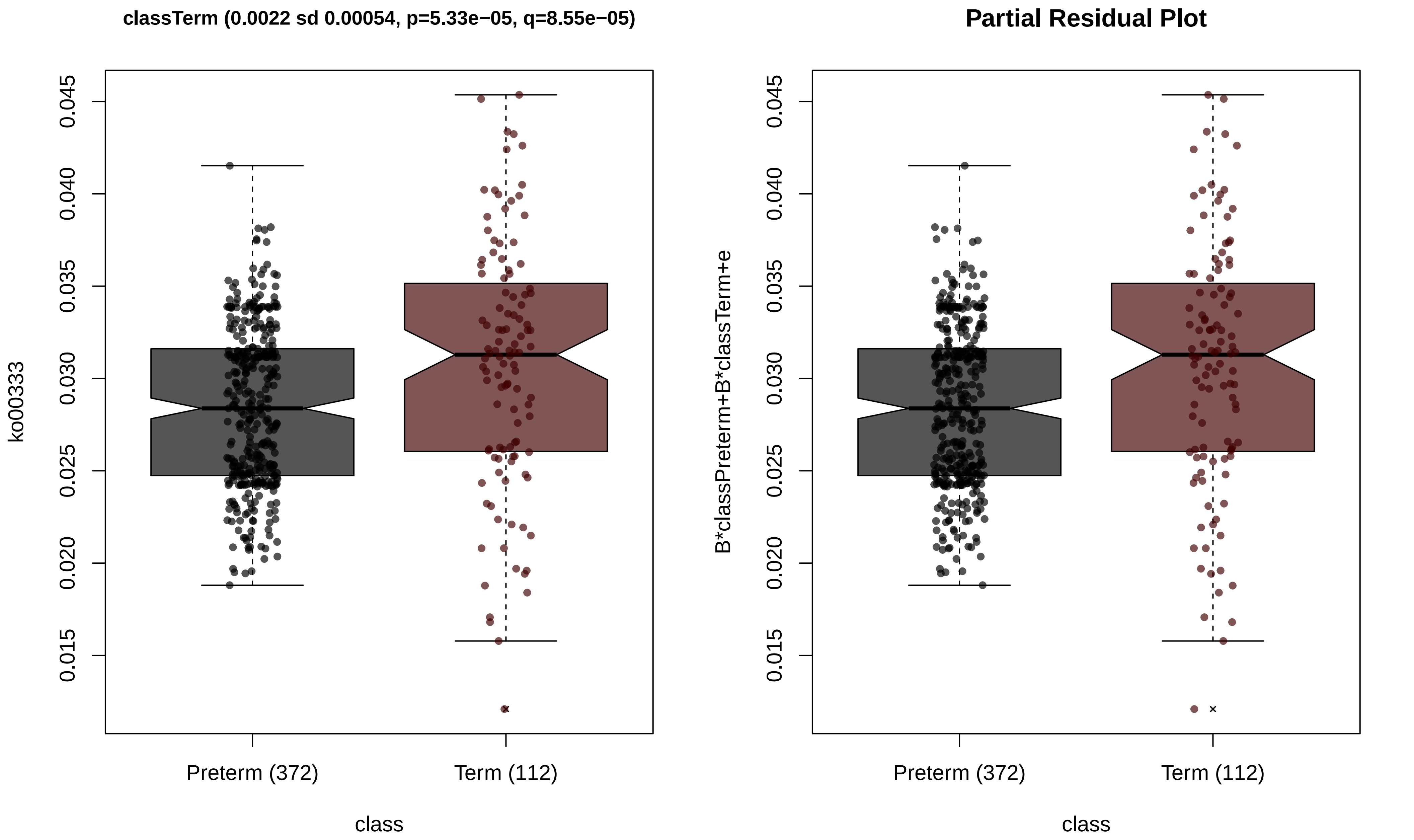
classTerm (0.0022 sd 0.00054, p=5.33e−05, q=8.55e−05)
Partial Residual Plot
ko00333
B*classPreterm+B*classTerm+e
0.015
0.020
0.025
0.030
0.035
0.040
0.045
Preterm (372)
Term (112)
class

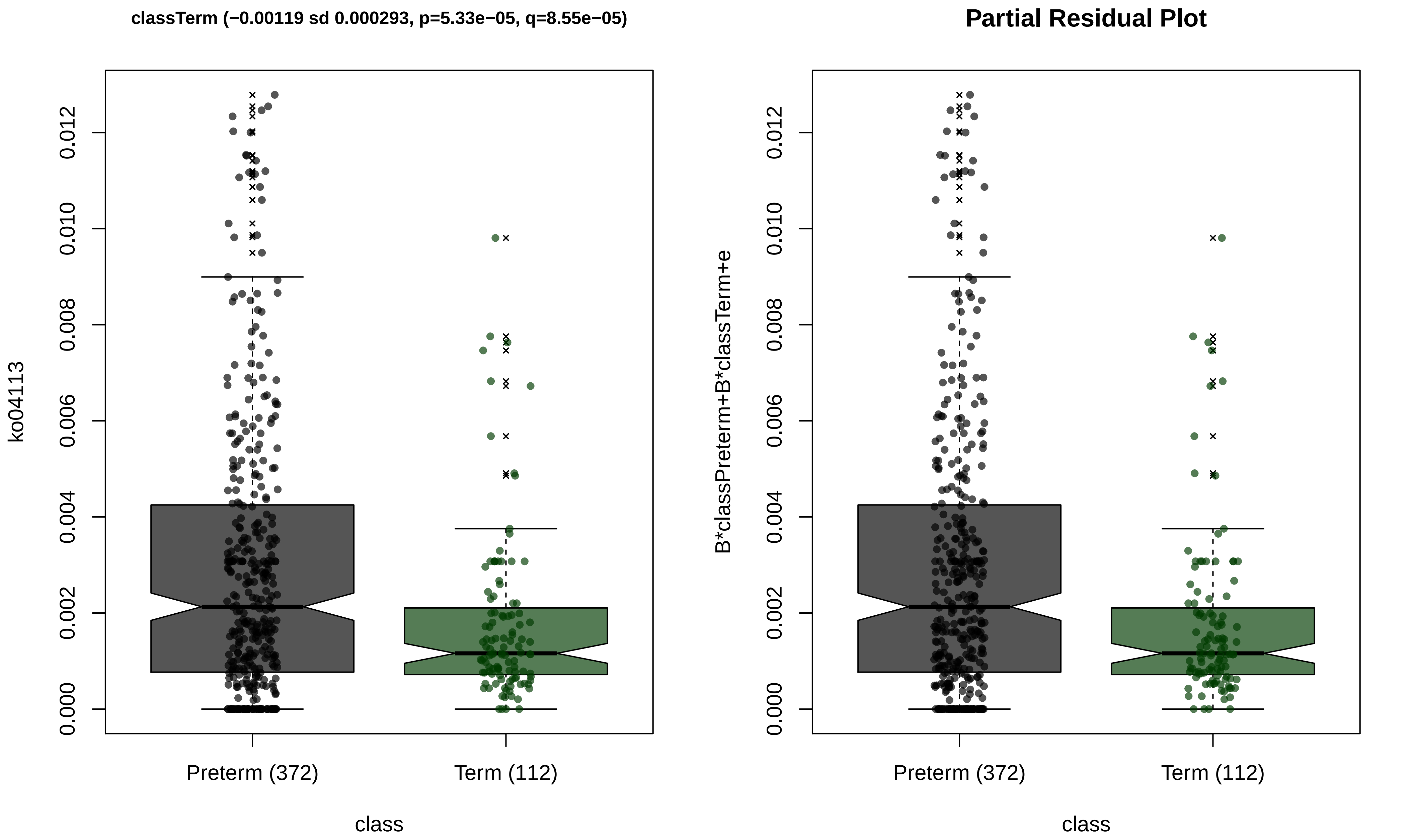
classTerm (−0.00119 sd 0.000293, p=5.33e−05, q=8.55e−05)
Partial Residual Plot
ko04113
B*classPreterm+B*classTerm+e
0.000
0.002
0.004
0.006
0.008
0.010
0.012
Preterm (372)
Term (112)
class

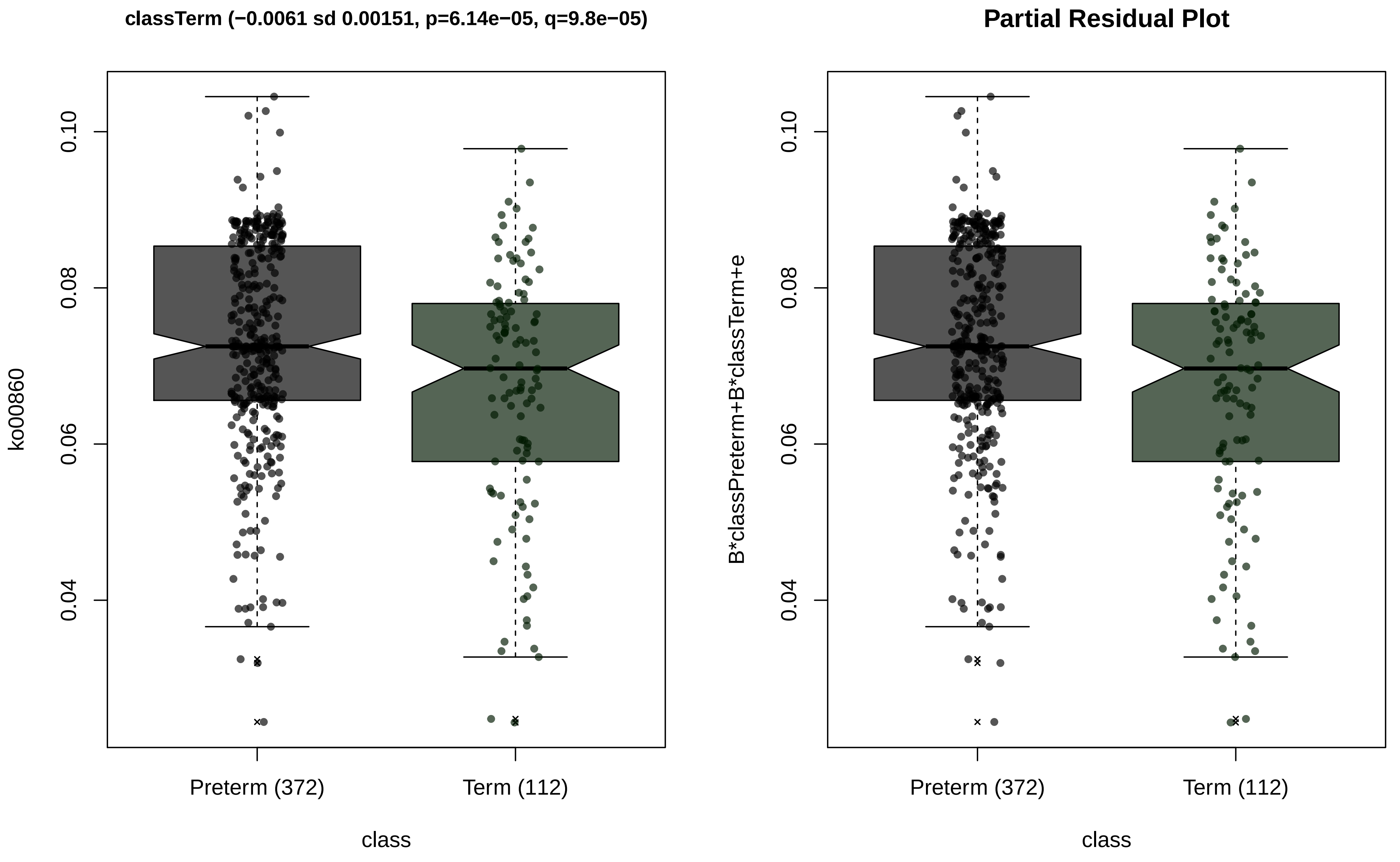
classTerm (−0.0061 sd 0.00151, p=6.14e−05, q=9.8e−05)
ko00860
0.04
0.06
0.08
0.10
Preterm (372)
Term (112)
class
Partial Residual Plot
B*classPreterm+B*classTerm+e
0.04
0.06
0.08
0.10
Preterm (372)
Term (112)
class

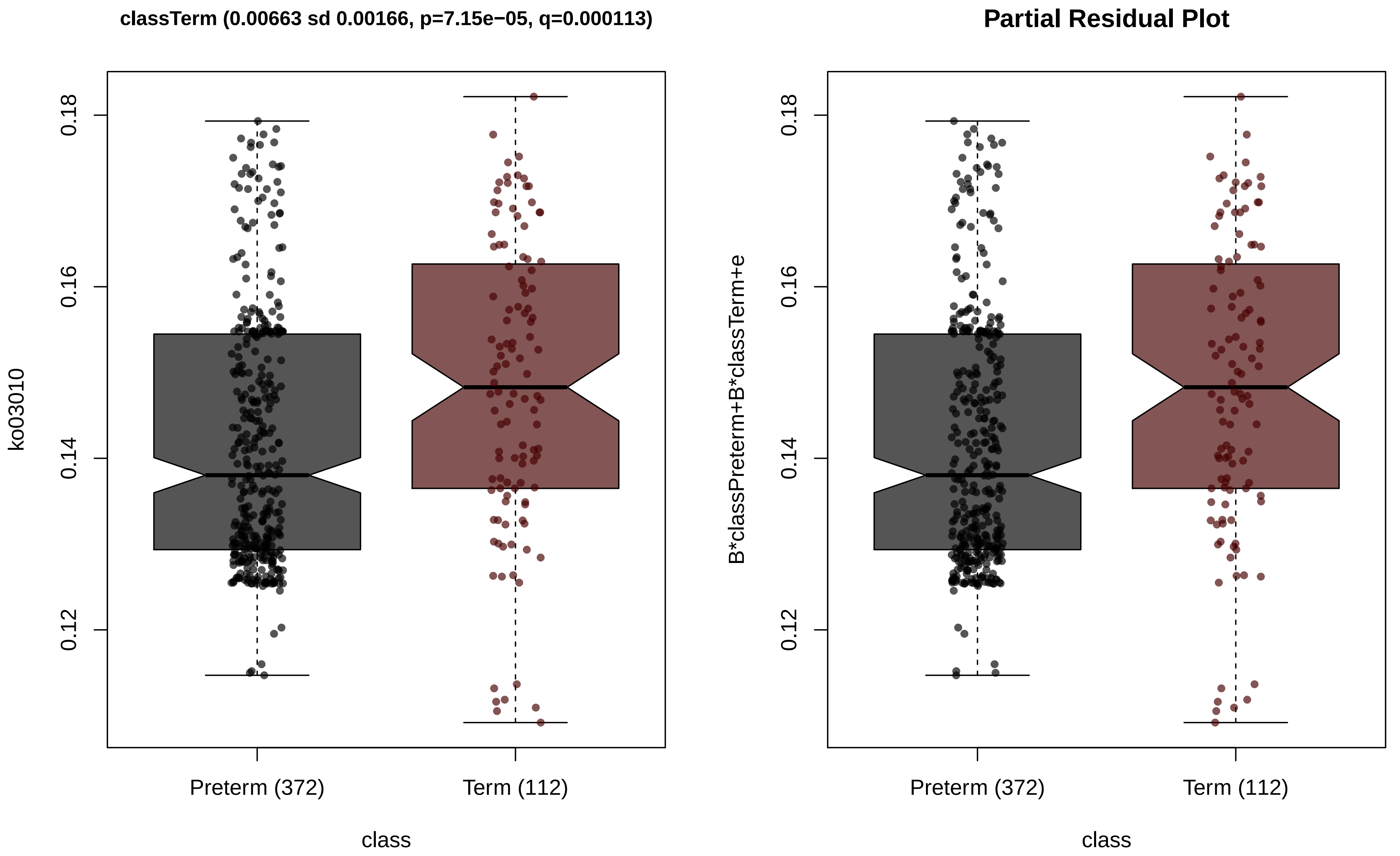

classTerm (0.00663 sd 0.00166, p=7.15e−05, q=0.000113)
ko03010
0.12
0.14
0.16
0.18
Preterm (372)
Term (112)
class
Partial Residual Plot
B*classPreterm+B*classTerm+e
0.12
0.14
0.16
0.18
Preterm (372)
Term (112)
class

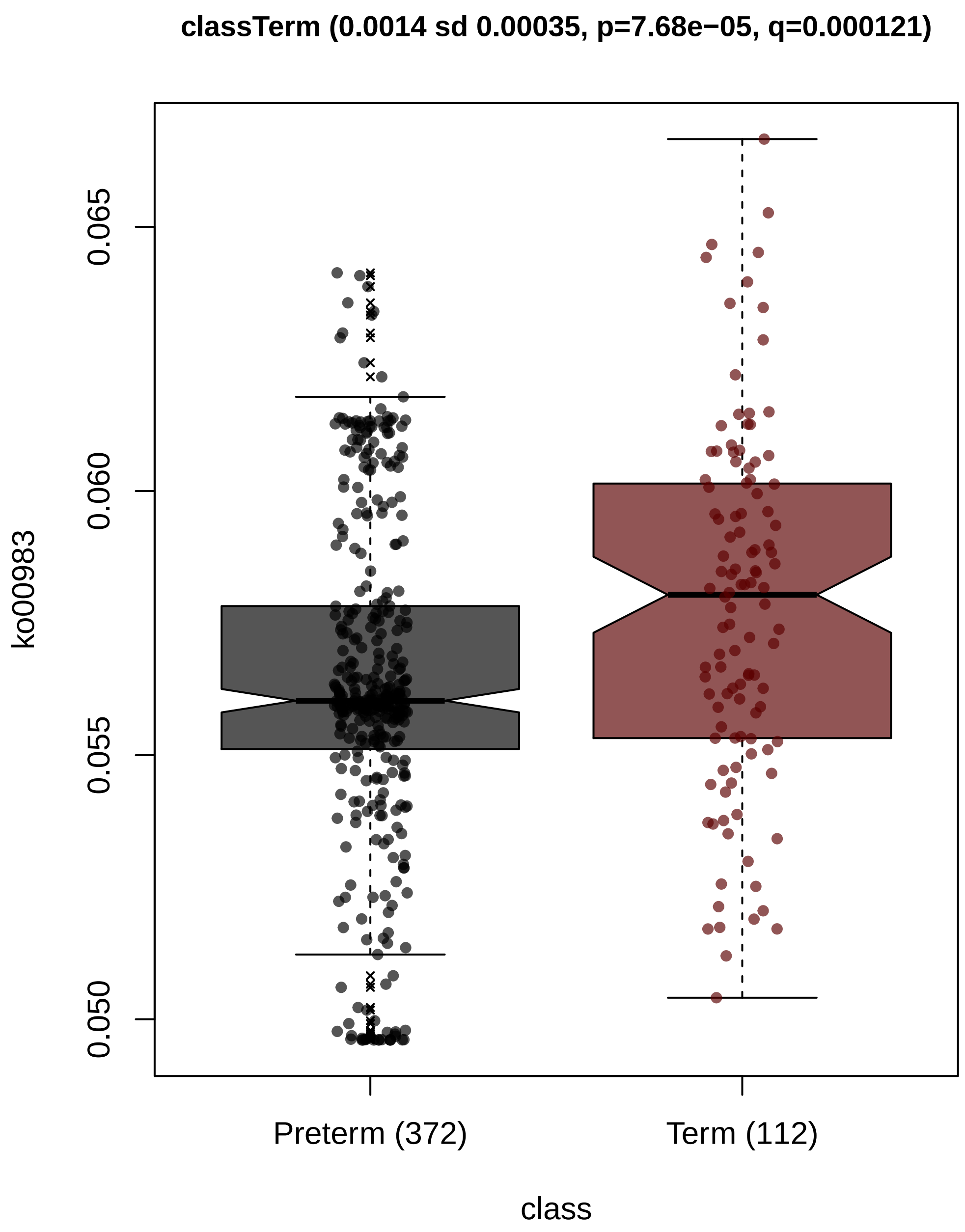

classTerm (0.0014 sd 0.00035, p=7.68e−05, q=0.000121)
ko00983
0.050
0.055
0.060
0.065
Preterm (372)
Term (112)
class

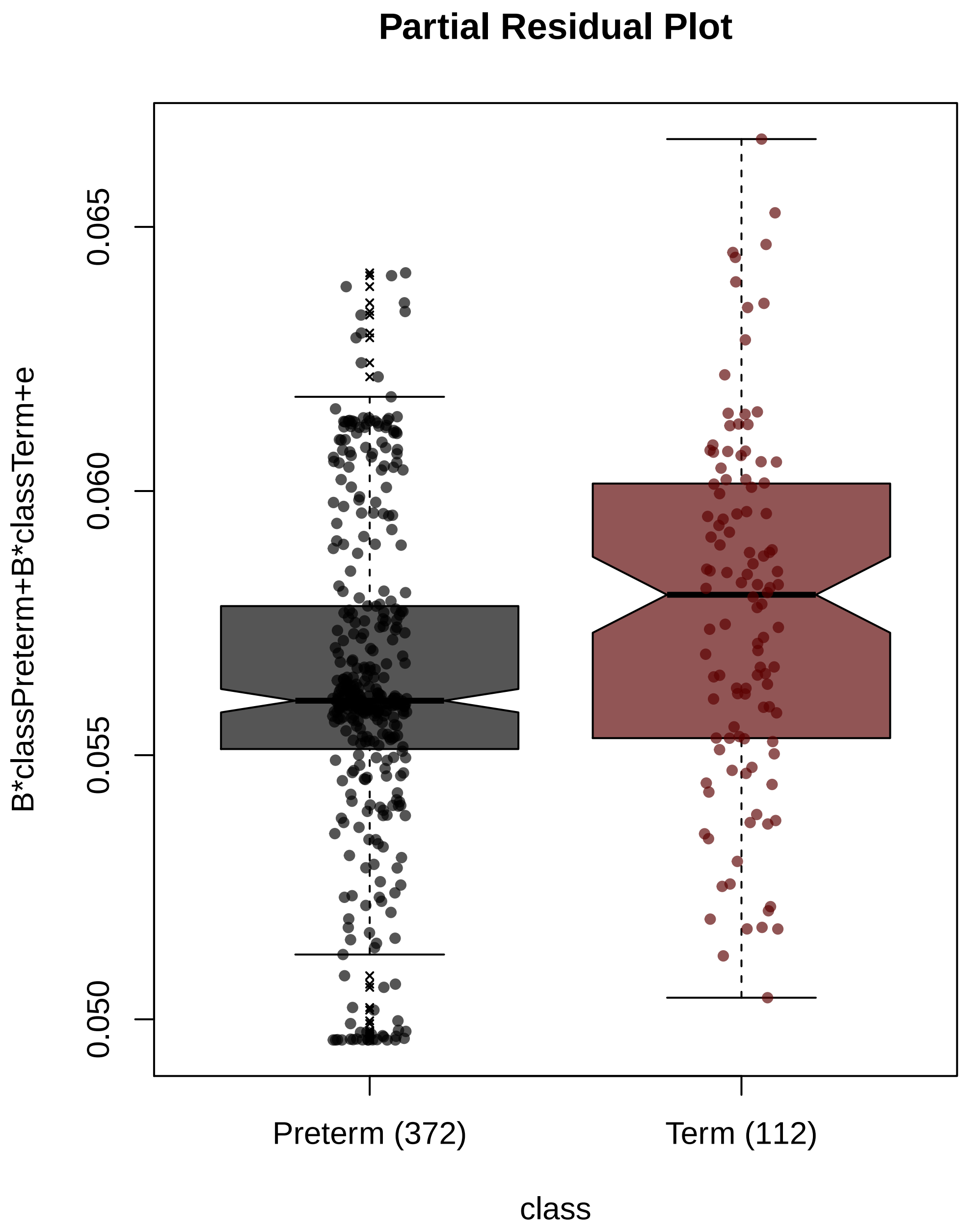

Partial Residual Plot
B*classPreterm+B*classTerm+e
0.050
0.055
0.060
0.065
Preterm (372)
Term (112)
class

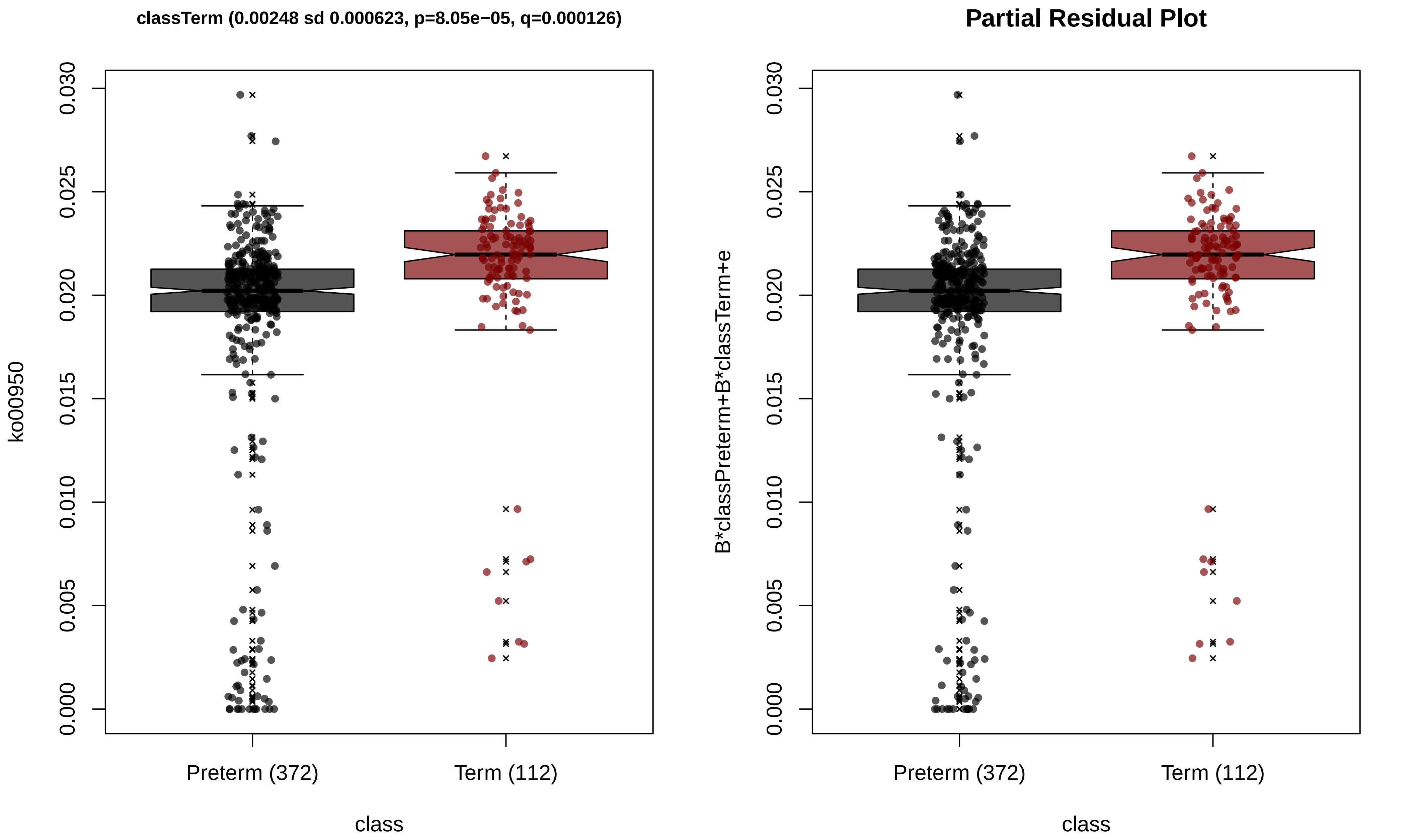
classTerm (0.00248 sd 0.000623, p=8.05e−05, q=0.000126)
Partial Residual Plot
ko00950
B*classPreterm+B*classTerm+e
0.000
0.005
0.010
0.015
0.020
0.025
0.030
Preterm (372)
Term (112)
class

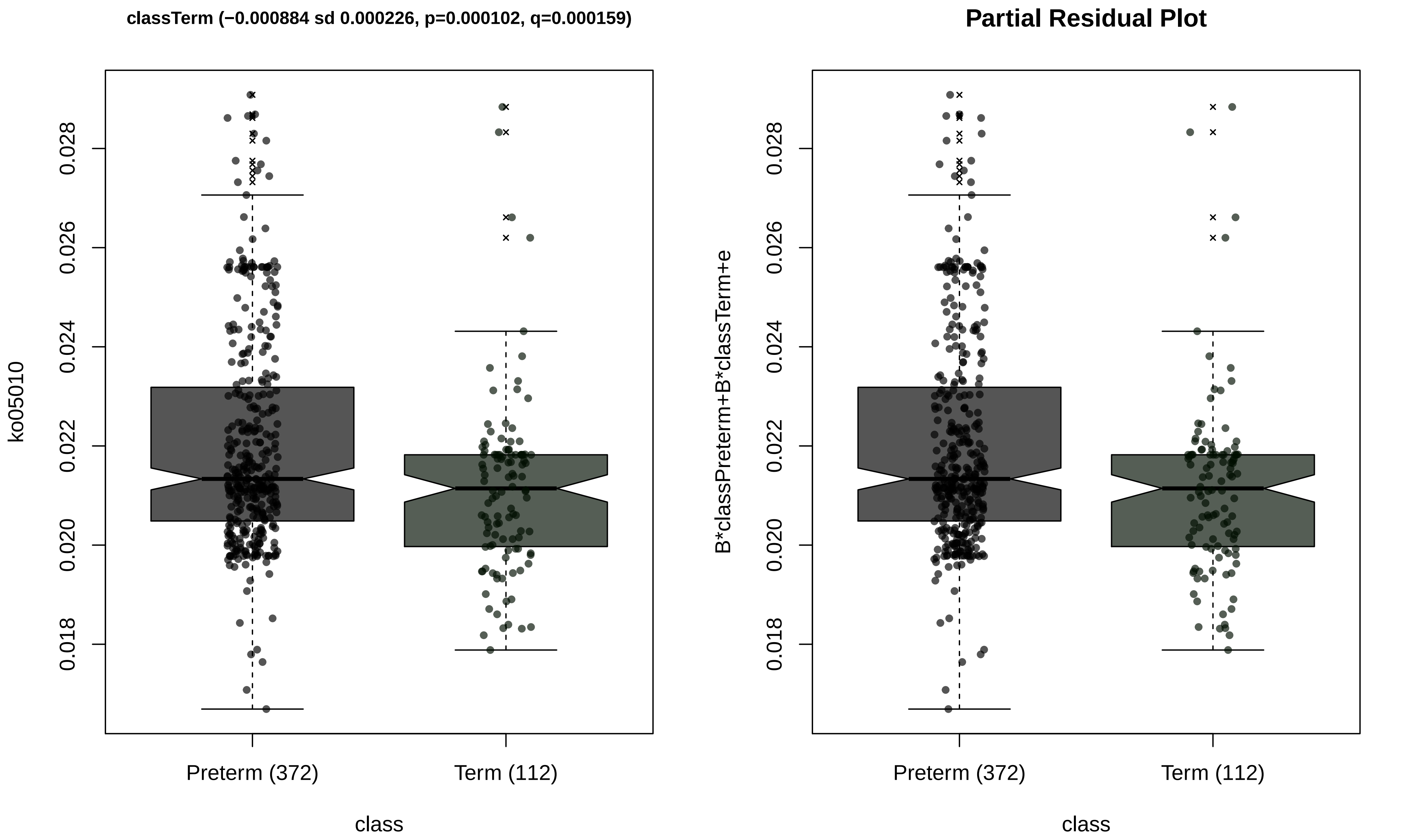

classTerm (−0.000884 sd 0.000226, p=0.000102, q=0.000159)
ko05010
0.018
0.020
0.022
0.024
0.026
0.028
Preterm (372)
Term (112)
class
Partial Residual Plot
B*classPreterm+B*classTerm+e
0.018
0.020
0.022
0.024
0.026
0.028
Preterm (372)
Term (112)
class

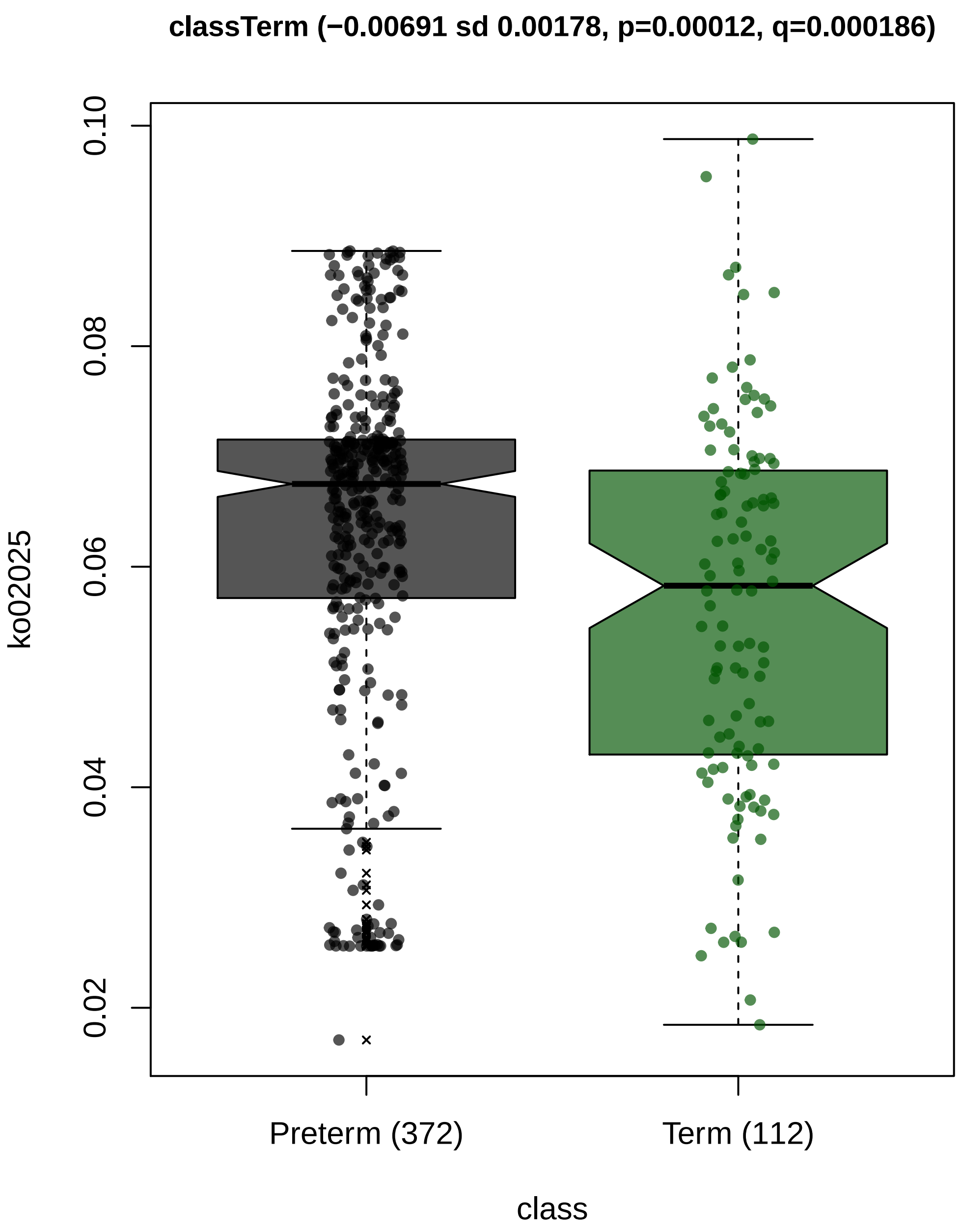

classTerm (−0.00691 sd 0.00178, p=0.00012, q=0.000186)
ko02025
0.02
0.04
0.06
0.08
0.10
Preterm (372)
Term (112)
class

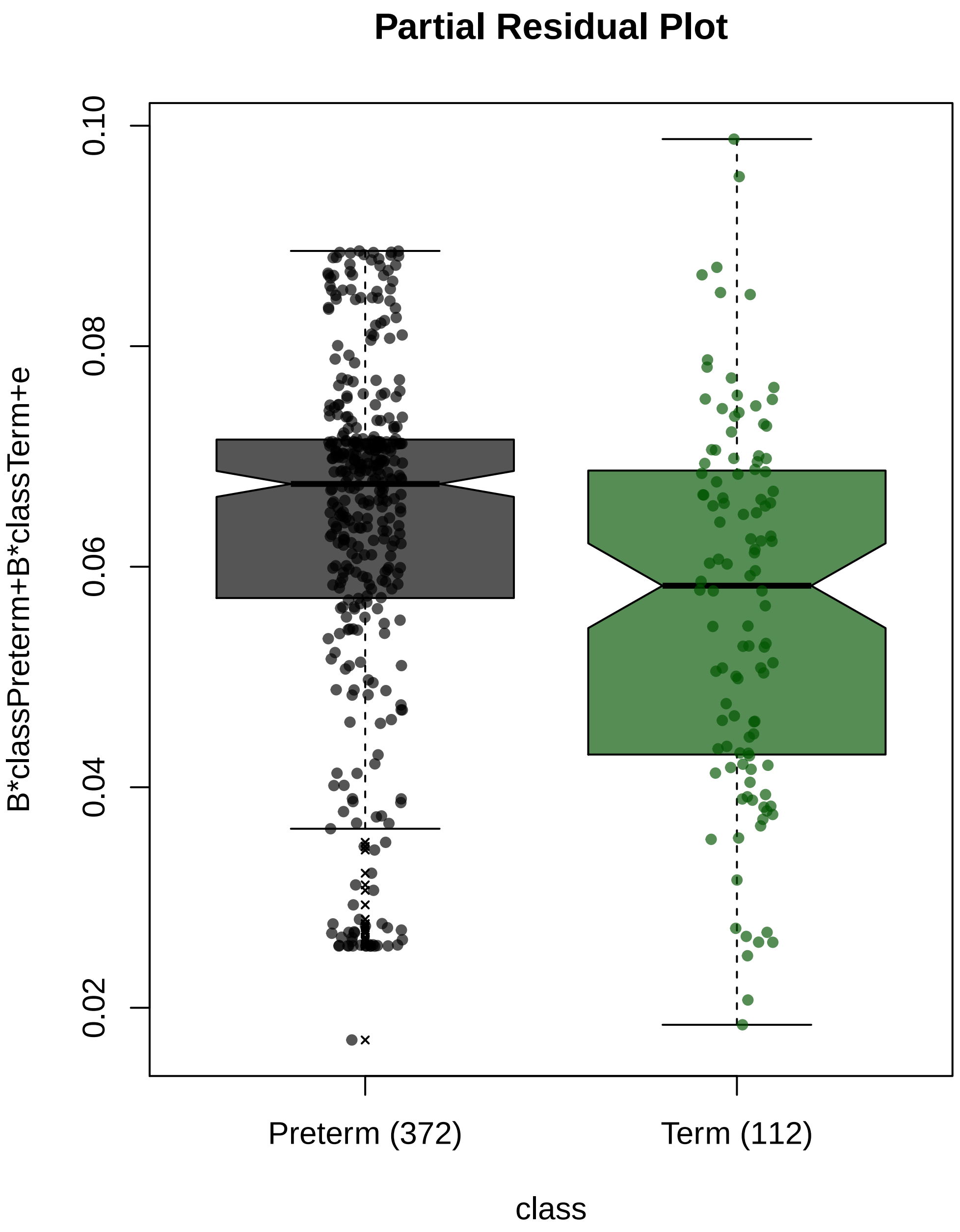

Partial Residual Plot
B*classPreterm+B*classTerm+e
0.02
0.04
0.06
0.08
0.10
Preterm (372)
Term (112)
class

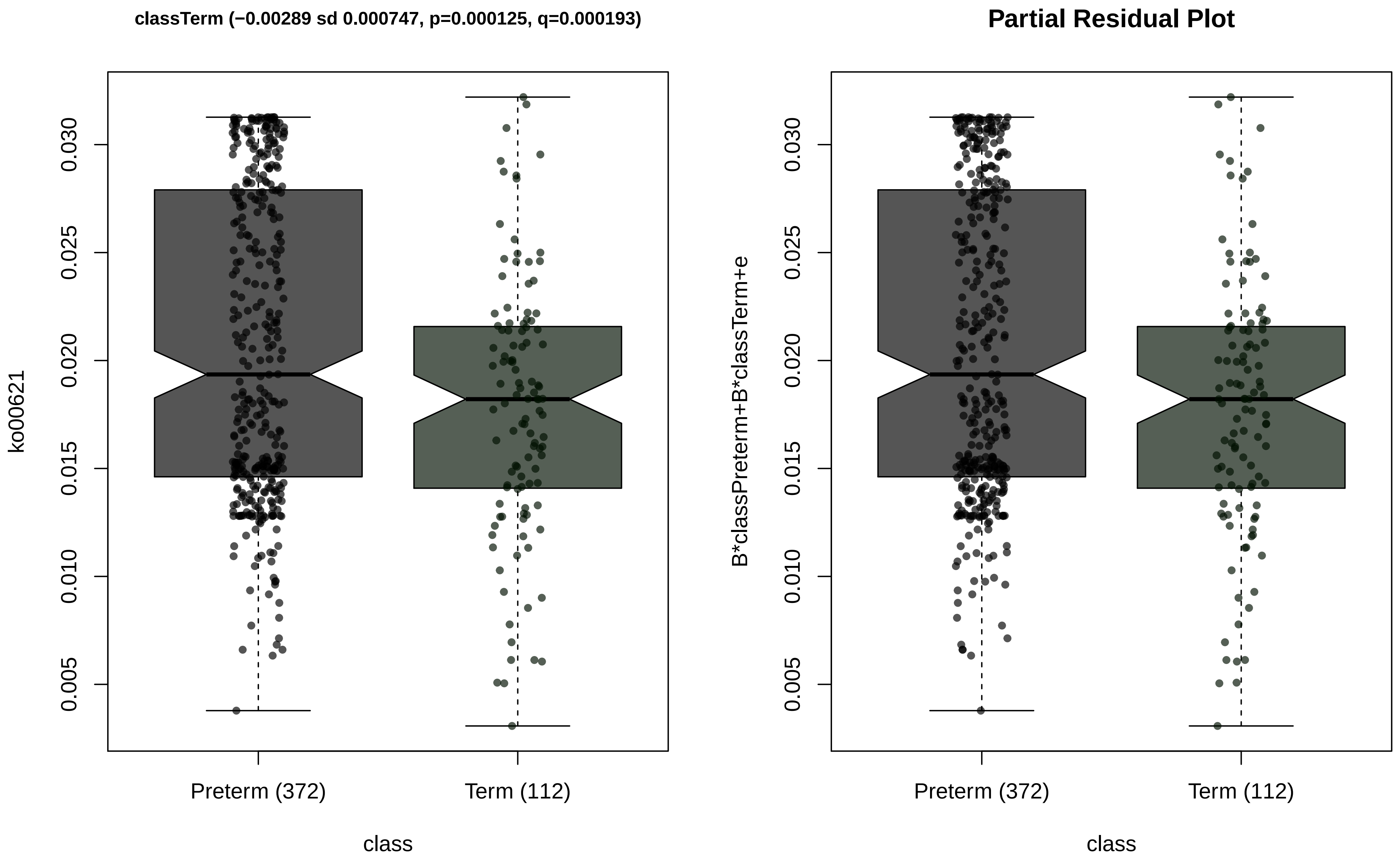
classTerm (−0.00289 sd 0.000747, p=0.000125, q=0.000193)
Partial Residual Plot
ko00621
B*classPreterm+B*classTerm+e
0.030
0.025
0.020
0.015
0.010
0.005
Preterm (372)
Term (112)
class

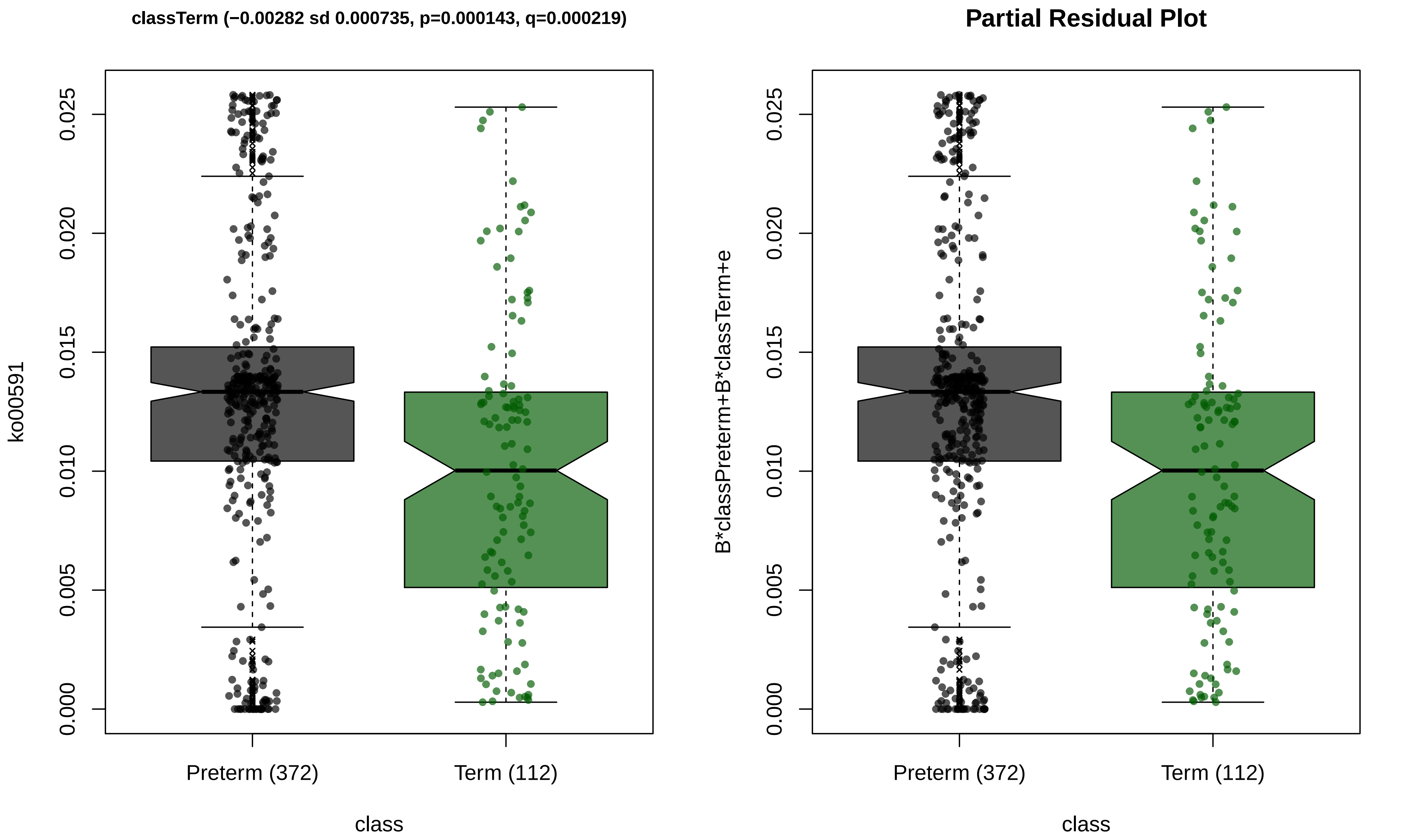

classTerm (−0.00282 sd 0.000735, p=0.000143, q=0.000219)
ko00591
0.000
0.005
0.010
0.015
0.020
0.025
Preterm (372)
Term (112)
class
Partial Residual Plot
B*classPreterm+B*classTerm+e
0.000
0.005
0.010
0.015
0.020
0.025
Preterm (372)
Term (112)
class

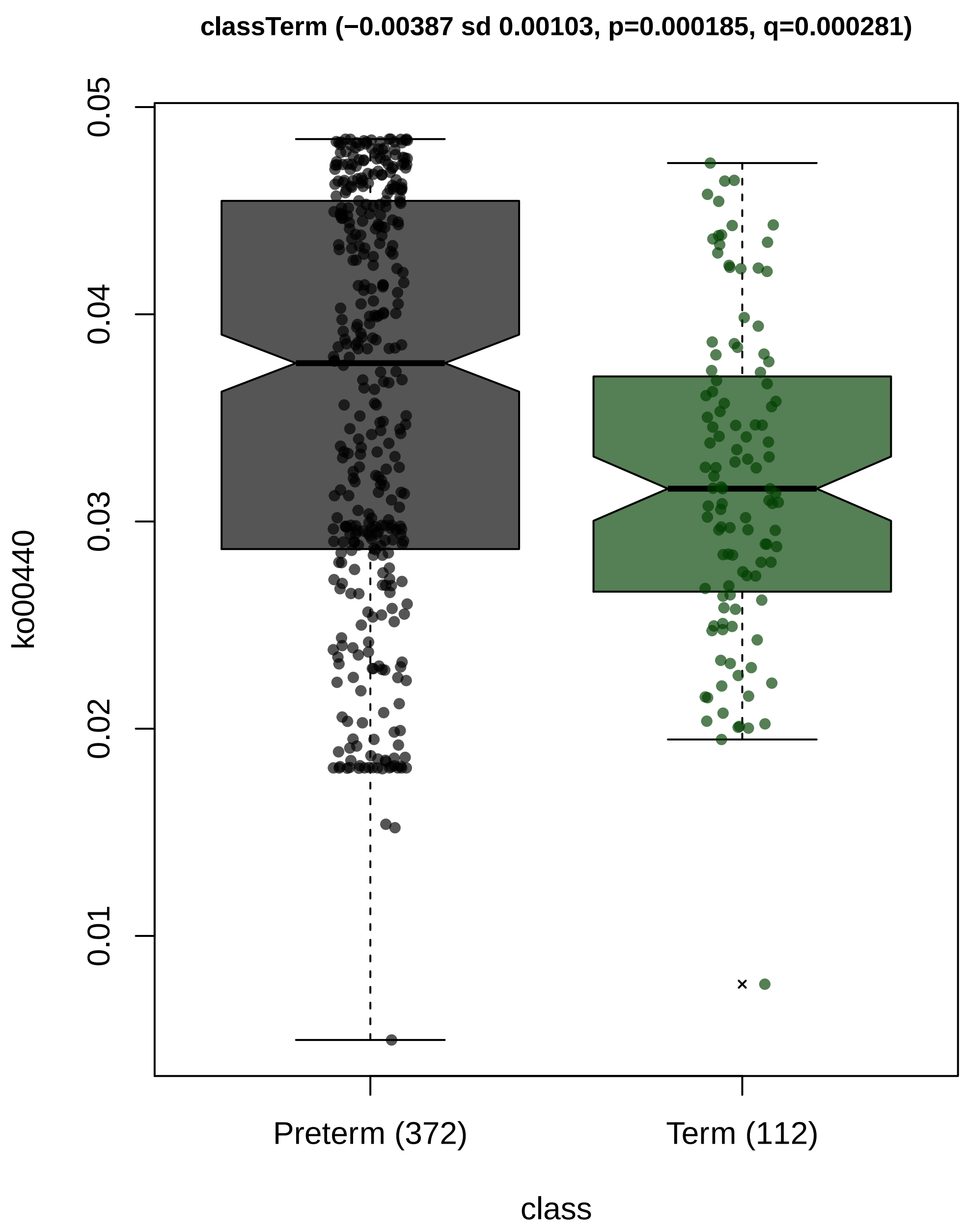

classTerm (−0.00387 sd 0.00103, p=0.000185, q=0.000281)
ko00440
0.05
0.04
0.03
0.02
0.01
Preterm (372)
Term (112)
class

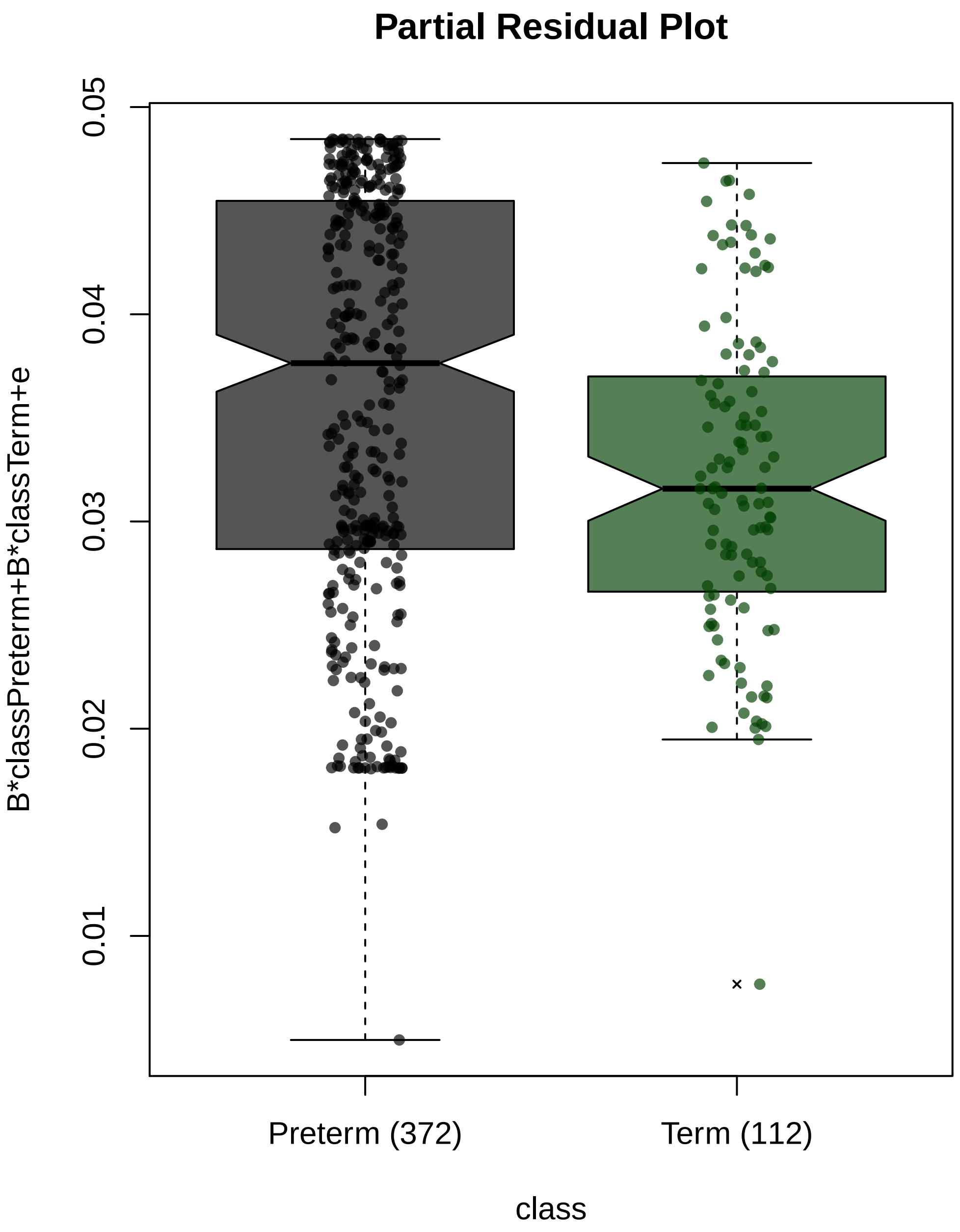

Partial Residual Plot
B*classPreterm+B*classTerm+e
0.05
0.04
0.03
0.02
0.01
Preterm (372)
Term (112)
class

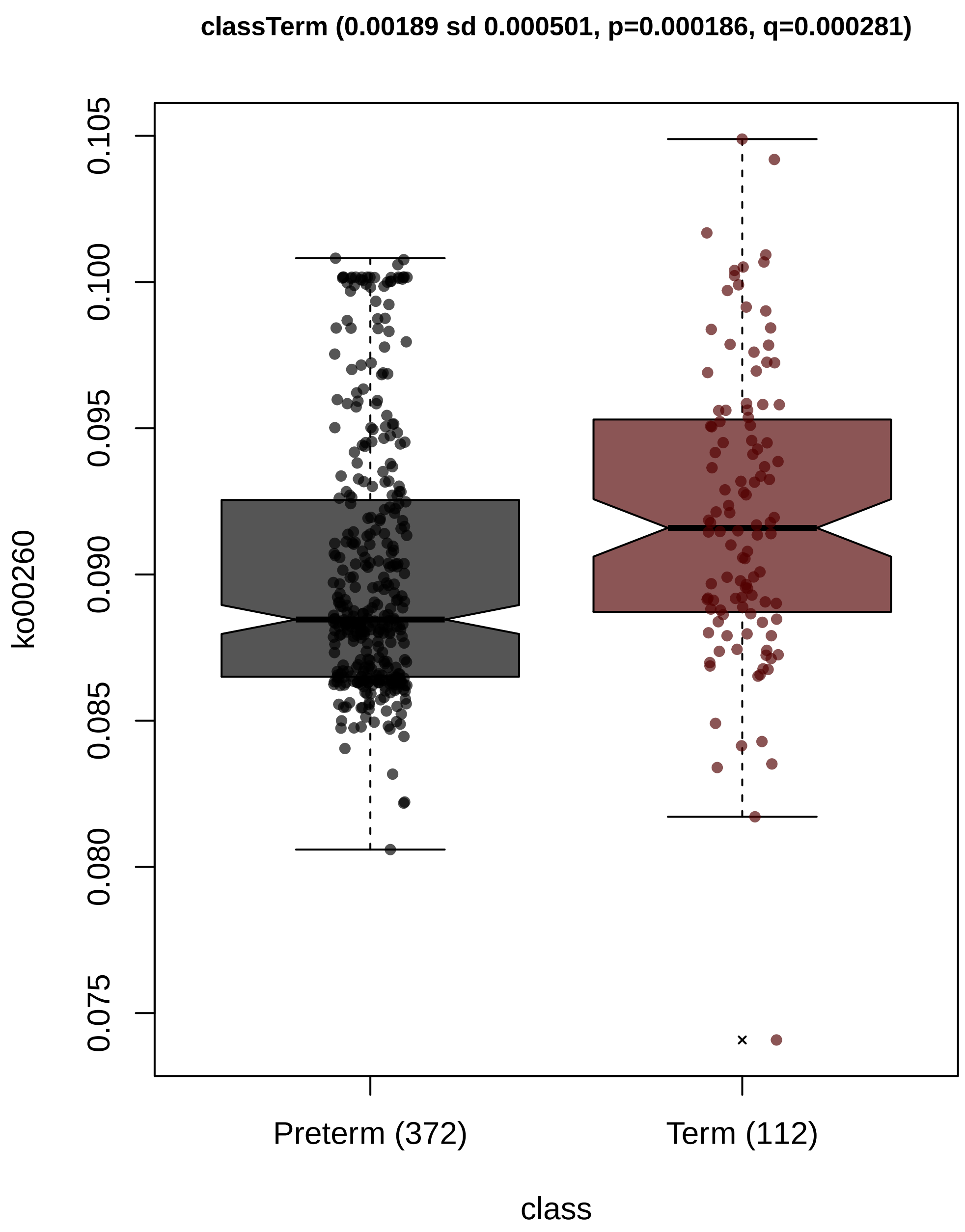

classTerm (0.00189 sd 0.000501, p=0.000186, q=0.000281)
ko00260
0.075
0.080
0.085
0.090
0.095
0.100
0.105
Preterm (372)
Term (112)
class

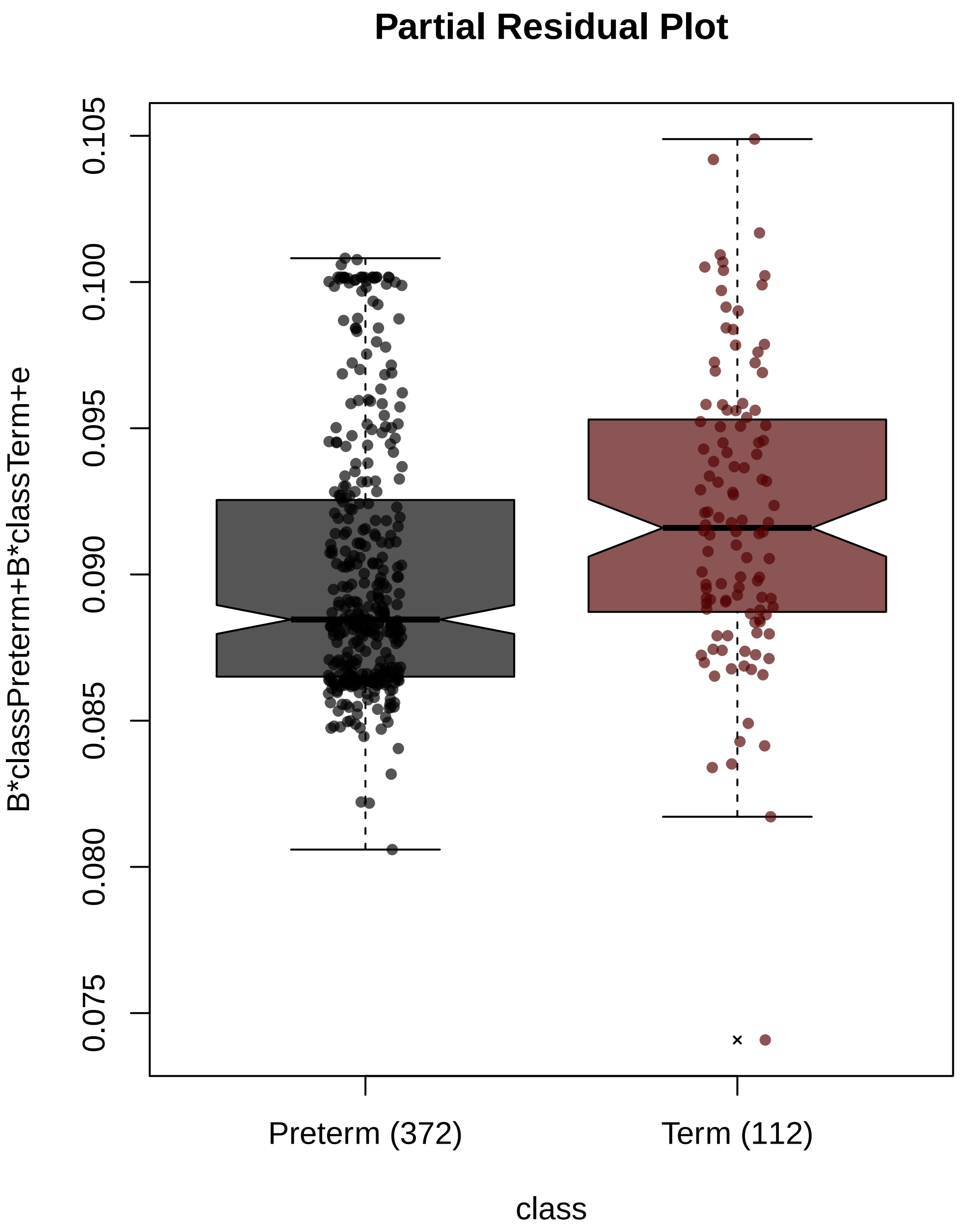

Partial Residual Plot
B*classPreterm+B*classTerm+e
0.075
0.080
0.085
0.090
0.095
0.100
0.105
Preterm (372)
Term (112)
class

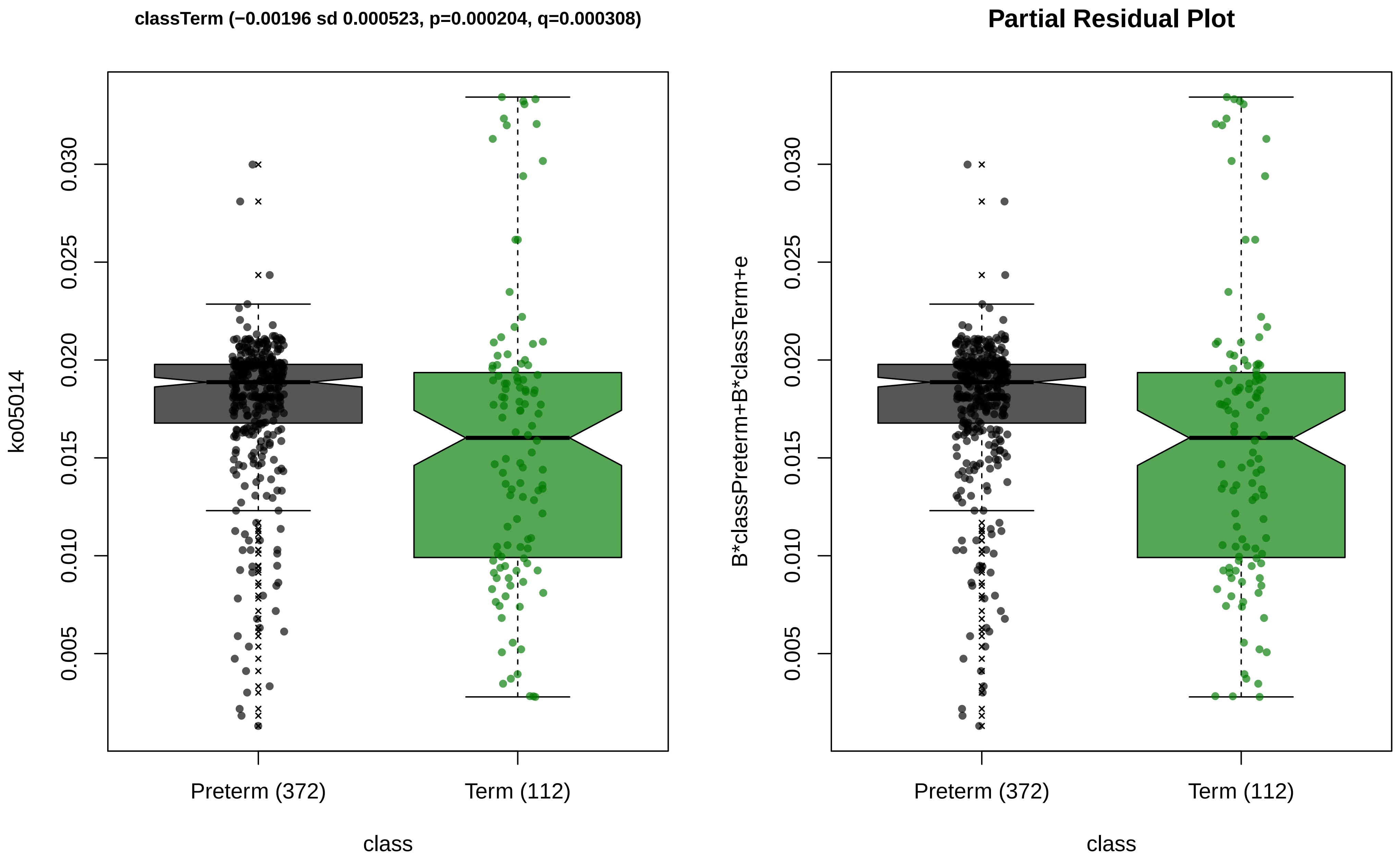

classTerm (−0.00196 sd 0.000523, p=0.000204, q=0.000308)
Partial Residual Plot
ko05014
B*classPreterm+B*classTerm+e
0.005
0.010
0.015
0.020
0.025
0.030
Preterm (372)
Term (112)
class

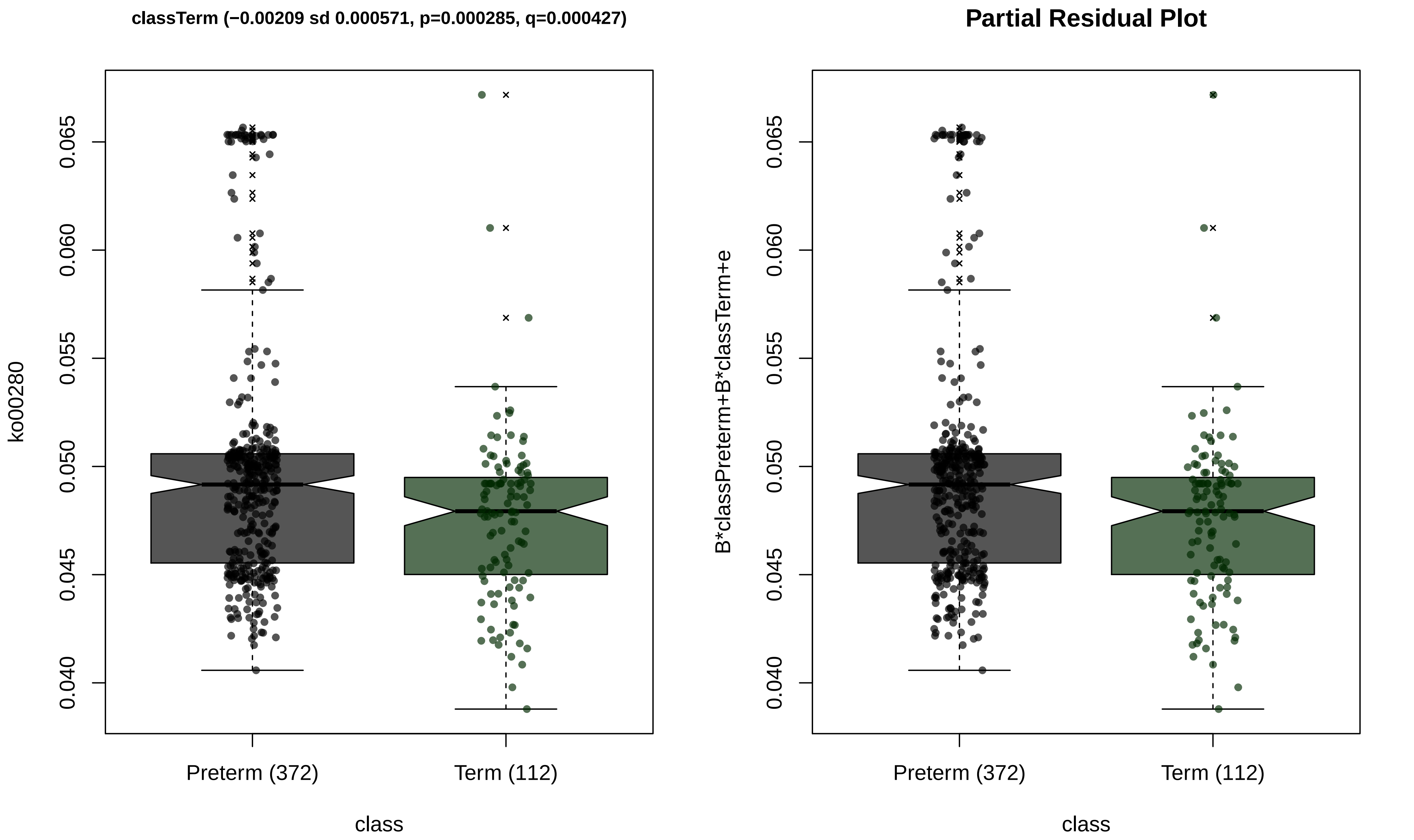
classTerm (−0.00209 sd 0.000571, p=0.000285, q=0.000427)
Partial Residual Plot
ko00280
B*classPreterm+B*classTerm+e
0.040
0.045
0.050
0.055
0.060
0.065
Preterm (372)
Term (112)
class

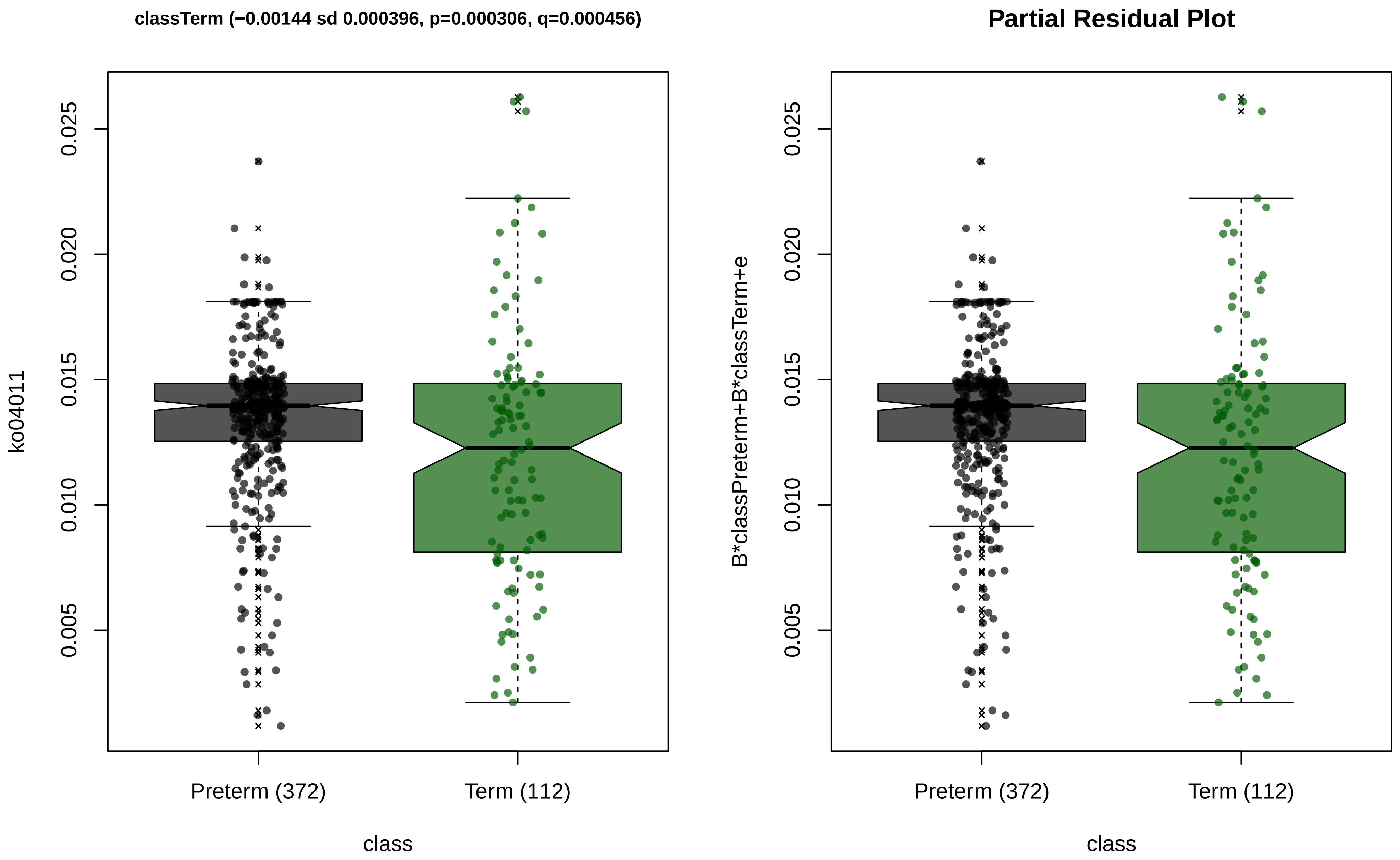
classTerm (−0.00144 sd 0.000396, p=0.000306, q=0.000456)
ko04011
0.005
0.010
0.015
0.020
0.025
Preterm (372)
Term (112)
class
Partial Residual Plot
B*classPreterm+B*classTerm+e
0.005
0.010
0.015
0.020
0.025
Preterm (372)
Term (112)
class

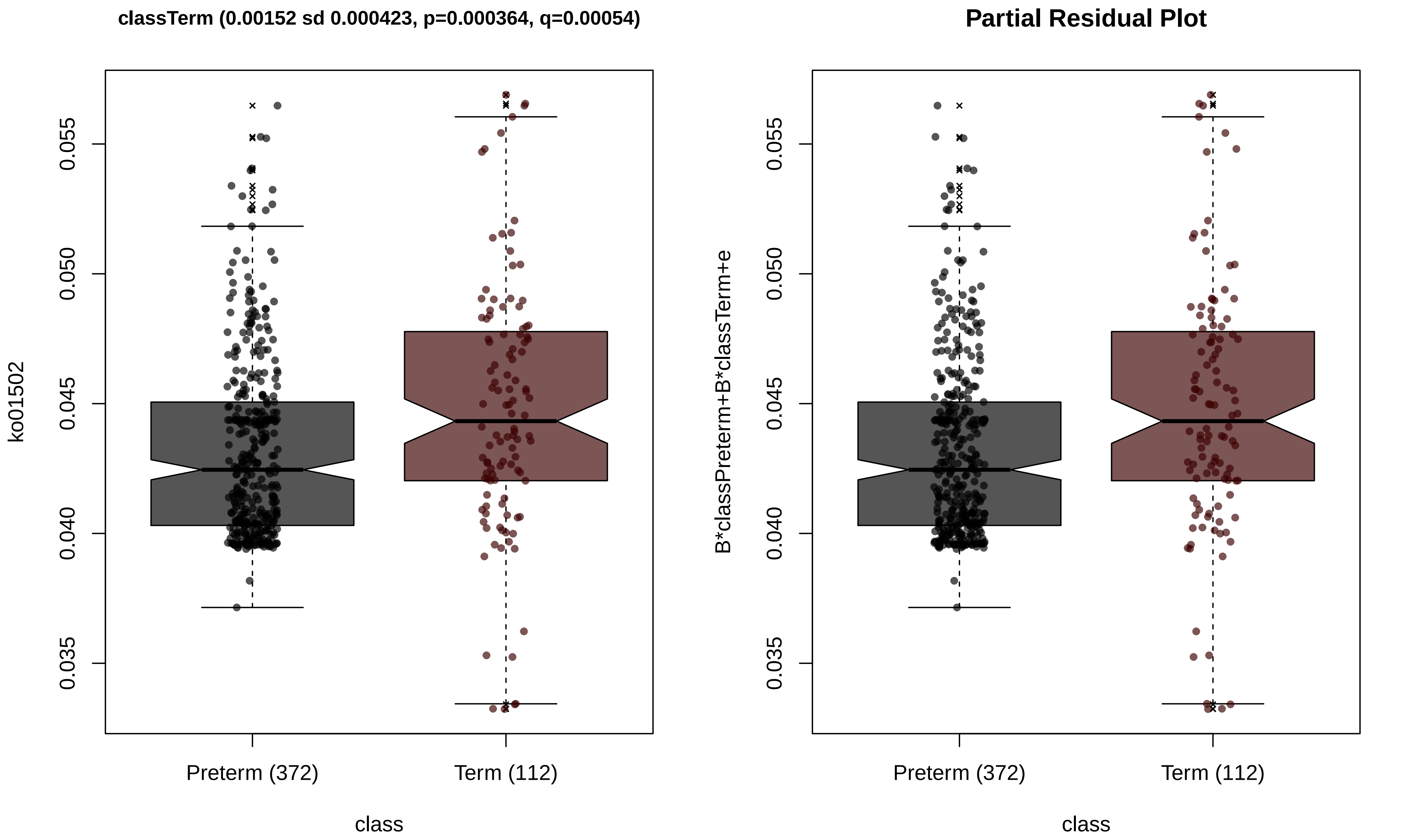

classTerm (0.00152 sd 0.000423, p=0.000364, q=0.00054)
ko01502
0.035
0.040
0.045
0.050
0.055
Preterm (372)
Term (112)
class
Partial Residual Plot
B*classPreterm+B*classTerm+e
0.035
0.040
0.045
0.050
0.055
Preterm (372)
Term (112)
class

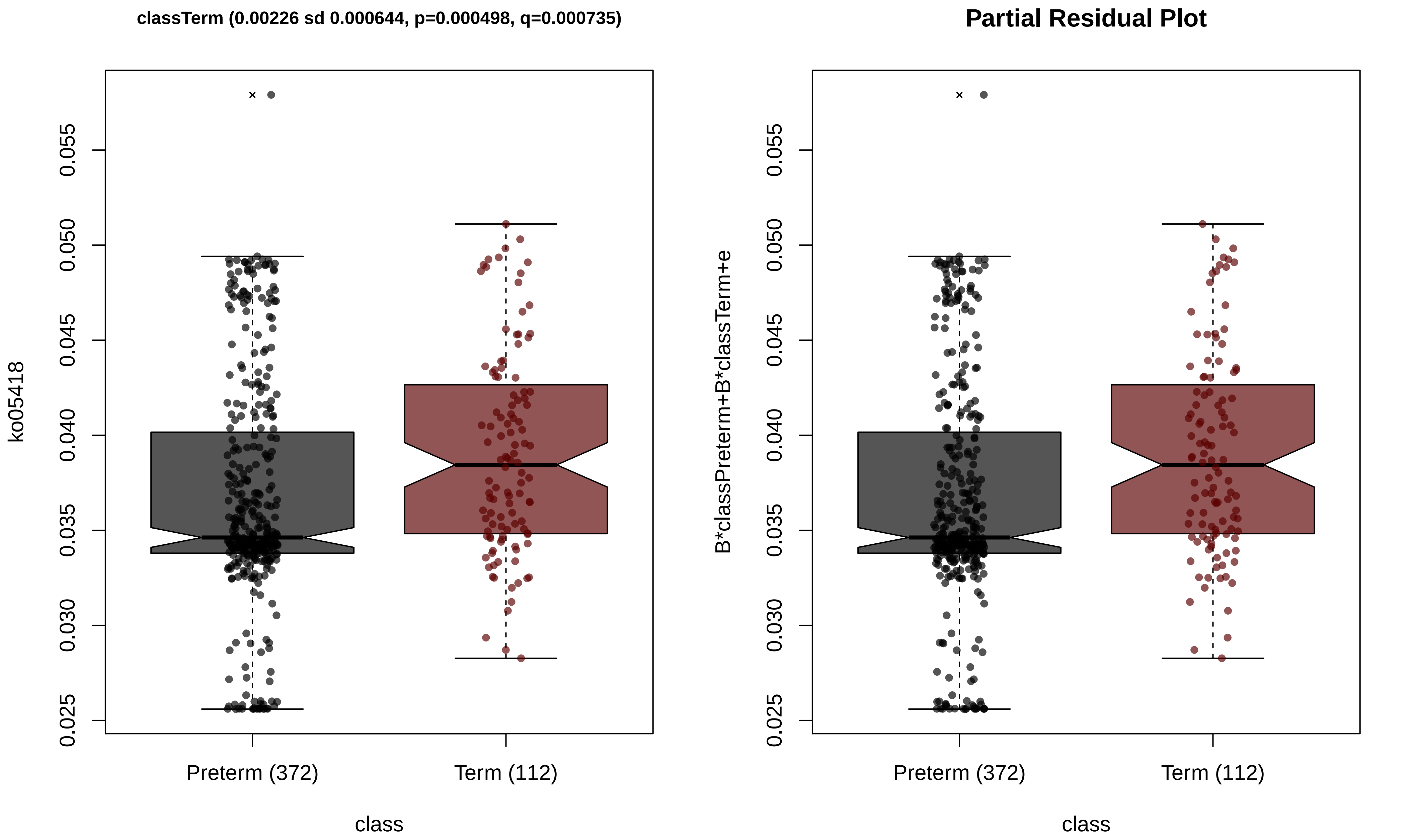
classTerm (0.00226 sd 0.000644, p=0.000498, q=0.000735)
Partial Residual Plot
ko05418
B*classPreterm+B*classTerm+e
0.025
0.030
0.035
0.040
0.045
0.050
0.055
Preterm (372)
Term (112)
class

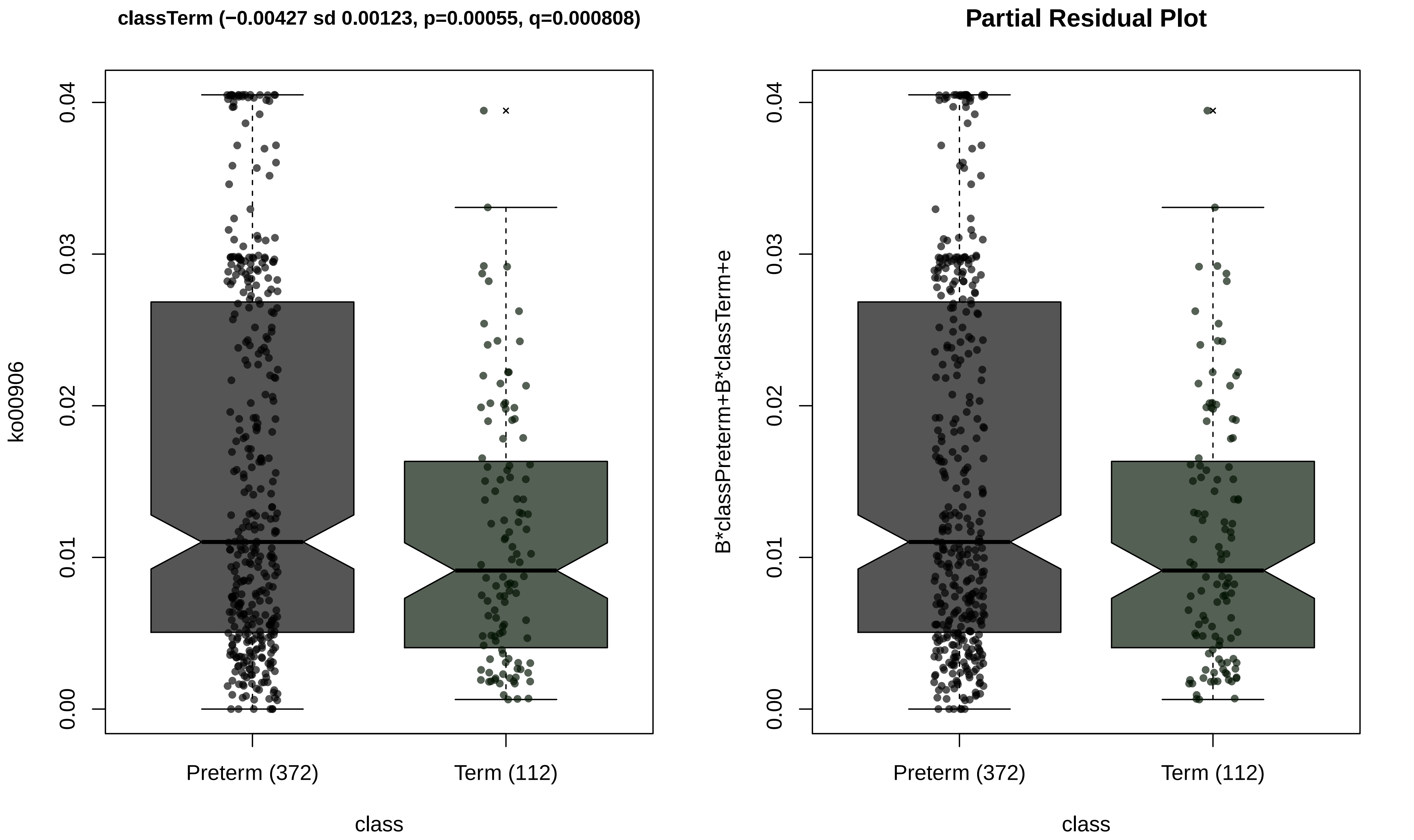
classTerm (−0.00427 sd 0.00123, p=0.00055, q=0.000808)
ko00906
0.00
0.01
0.02
0.03
0.04
Preterm (372)
Term (112)
class
Partial Residual Plot
B*classPreterm+B*classTerm+e
0.00
0.01
0.02
0.03
0.04
Preterm (372)
Term (112)
class

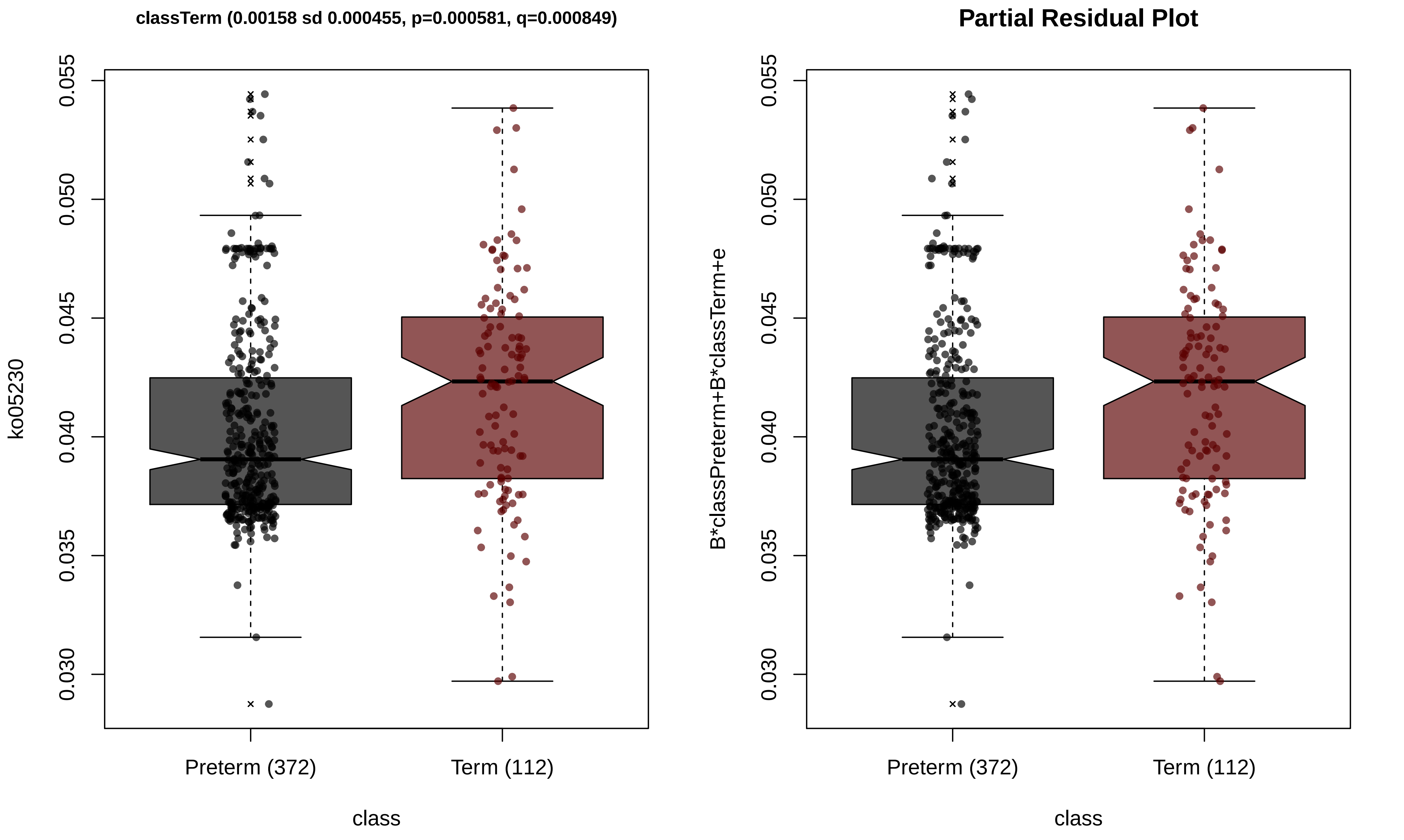

classTerm (0.00158 sd 0.000455, p=0.000581, q=0.000849)
ko05230
0.030
0.035
0.040
0.045
0.050
0.055
Preterm (372)
Term (112)
class
Partial Residual Plot
B*classPreterm+B*classTerm+e
0.030
0.035
0.040
0.045
0.050
0.055
Preterm (372)
Term (112)
class

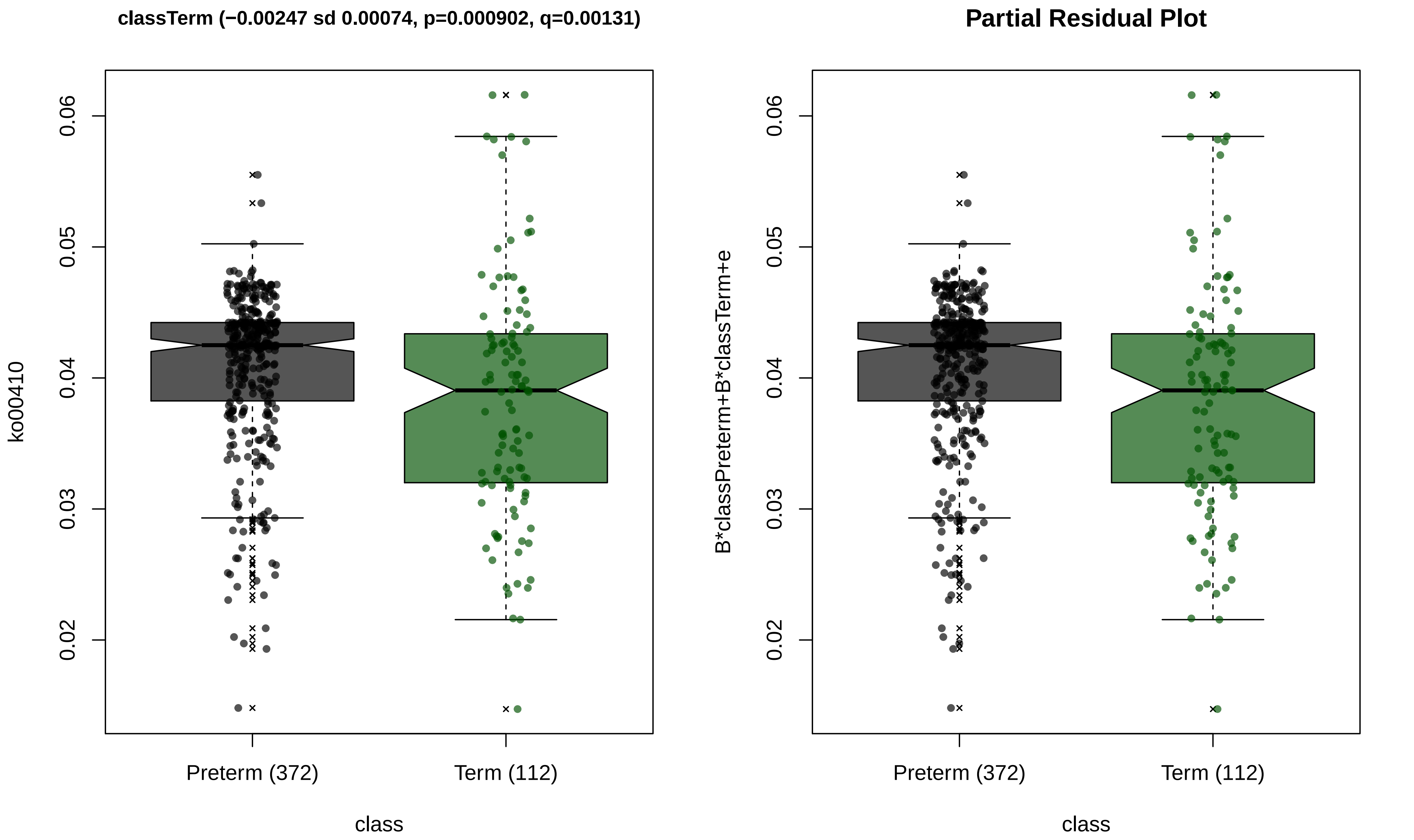

classTerm (−0.00247 sd 0.00074, p=0.000902, q=0.00131)
ko00410
0.02
0.03
0.04
0.05
0.06
Preterm (372)
Term (112)
class
Partial Residual Plot
B*classPreterm+B*classTerm+e
0.02
0.03
0.04
0.05
0.06
Preterm (372)
Term (112)
class

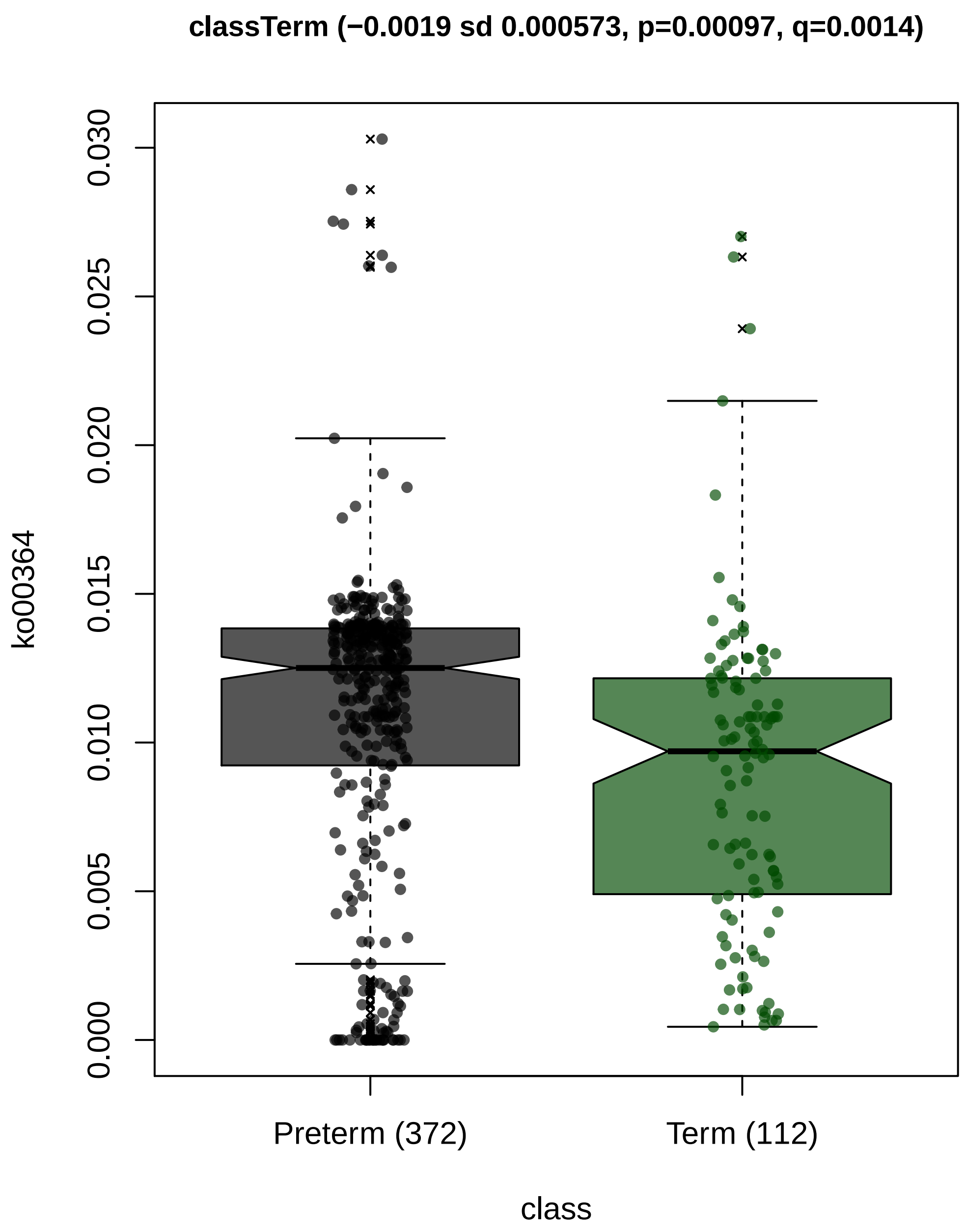
classTerm (−0.0019 sd 0.000573, p=0.00097, q=0.0014)
ko00364
0.000
0.005
0.010
0.015
0.020
0.025
0.030
Preterm (372)
Term (112)
class
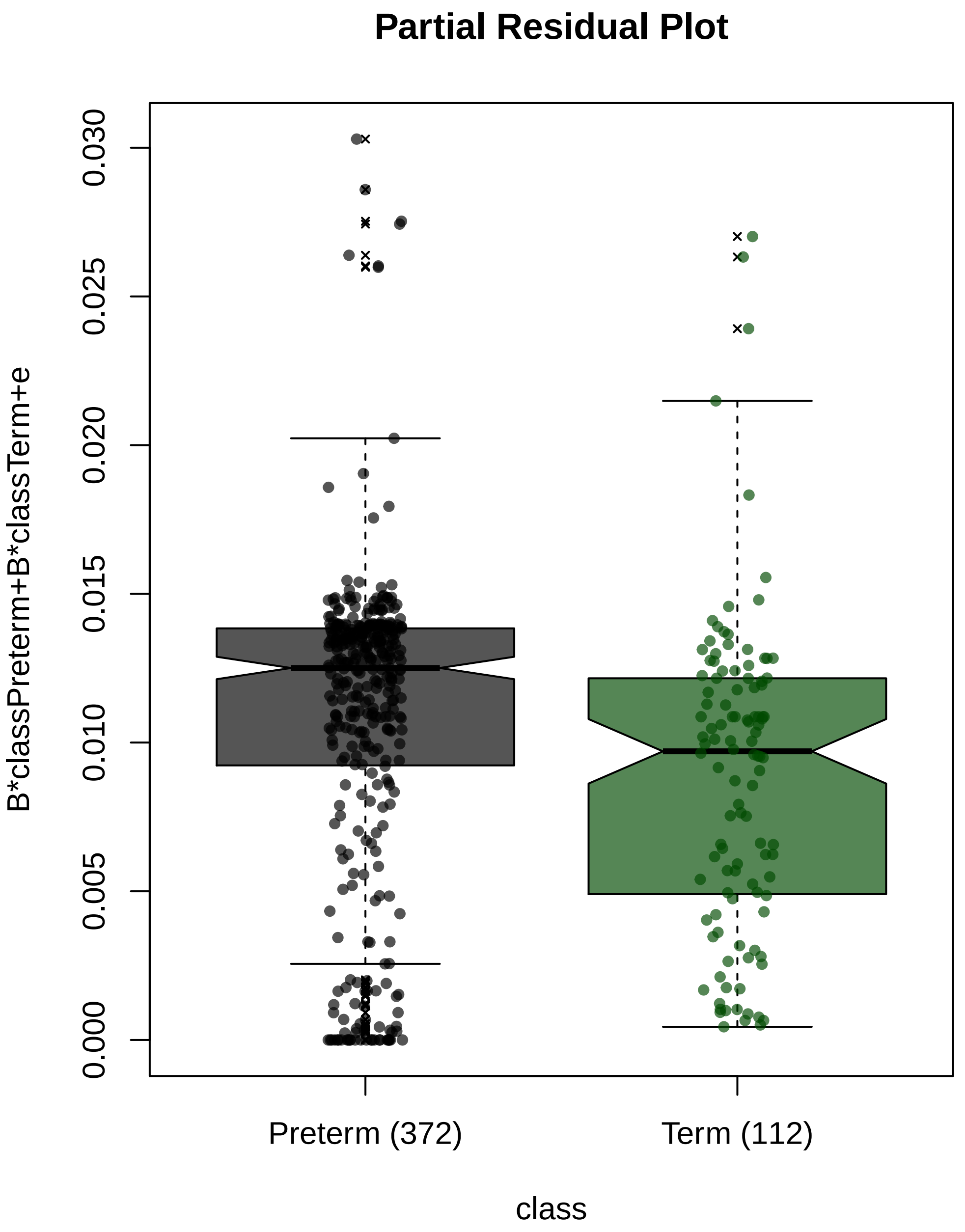
Partial Residual Plot
B*classPreterm+B*classTerm+e
0.000
0.005
0.010
0.015
0.020
0.025
0.030
Preterm (372)
Term (112)
class

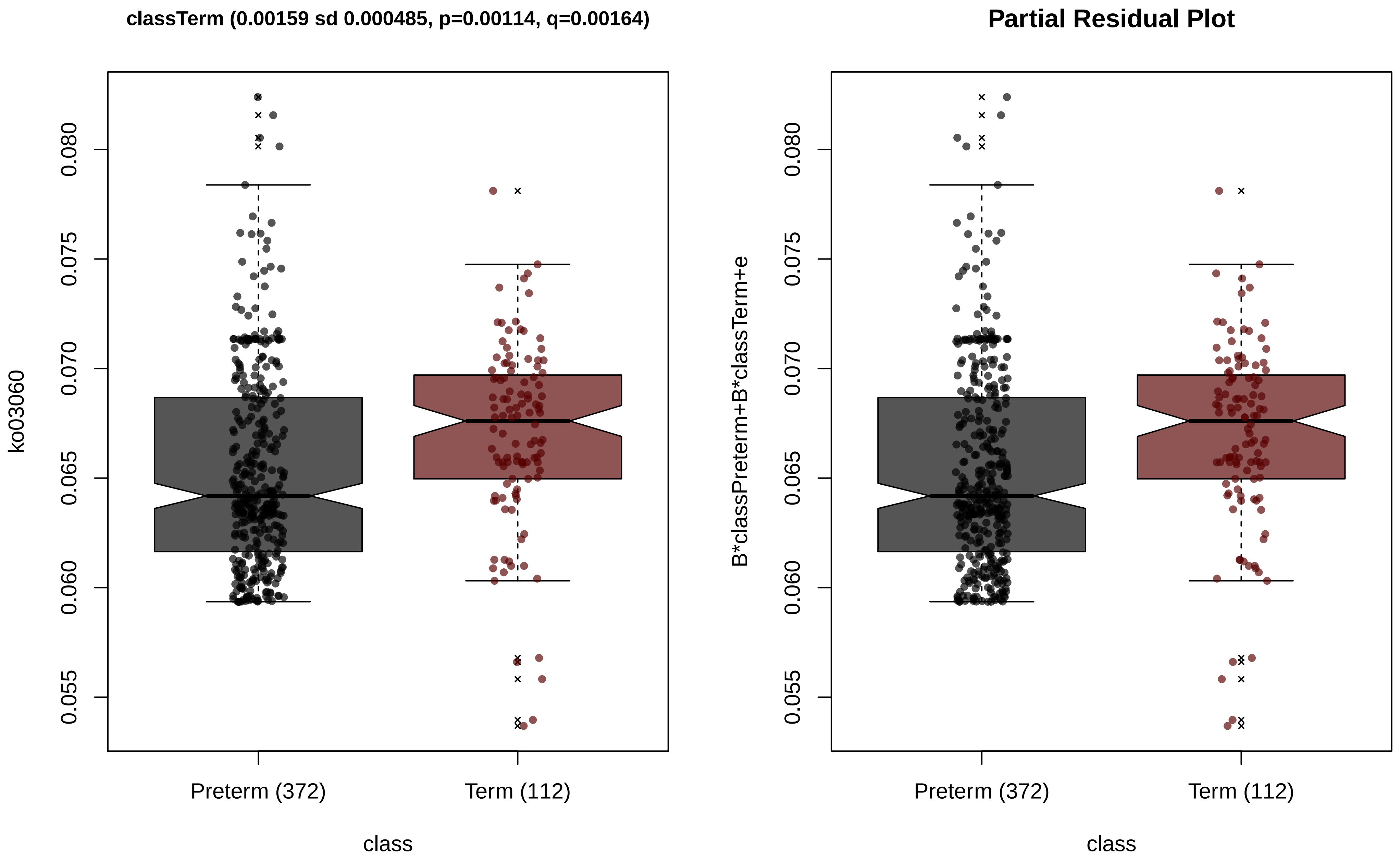

classTerm (0.00159 sd 0.000485, p=0.00114, q=0.00164)
ko03060
0.055
0.060
0.065
0.070
0.075
0.080
Preterm (372)
Term (112)
class
Partial Residual Plot
B*classPreterm+B*classTerm+e
0.055
0.060
0.065
0.070
0.075
0.080
Preterm (372)
Term (112)
class

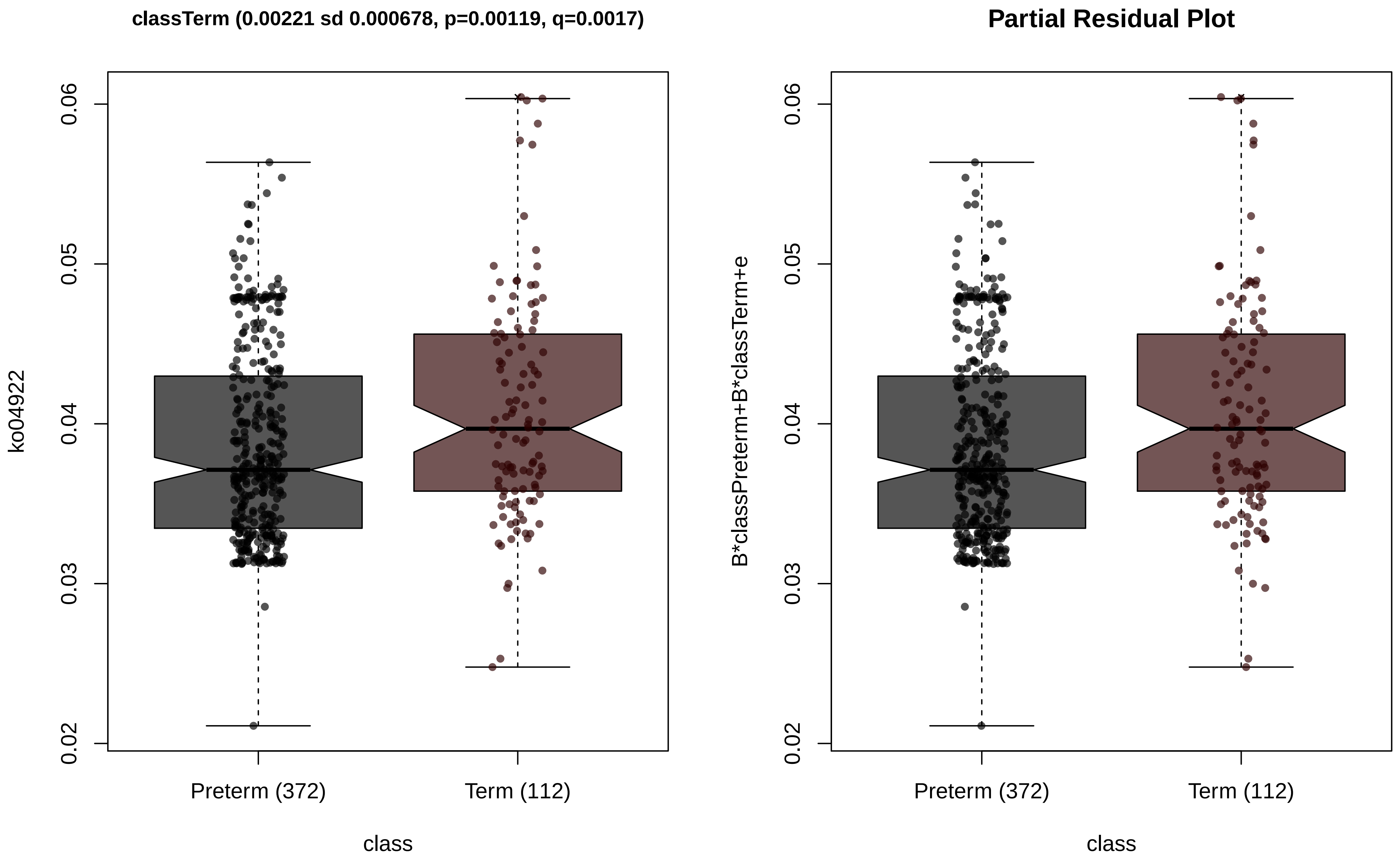

classTerm (0.00221 sd 0.000678, p=0.00119, q=0.0017)
ko04922
0.02
0.03
0.04
0.05
0.06
Preterm (372)
Term (112)
class
Partial Residual Plot
B*classPreterm+B*classTerm+e
0.02
0.03
0.04
0.05
0.06
Preterm (372)
Term (112)
class

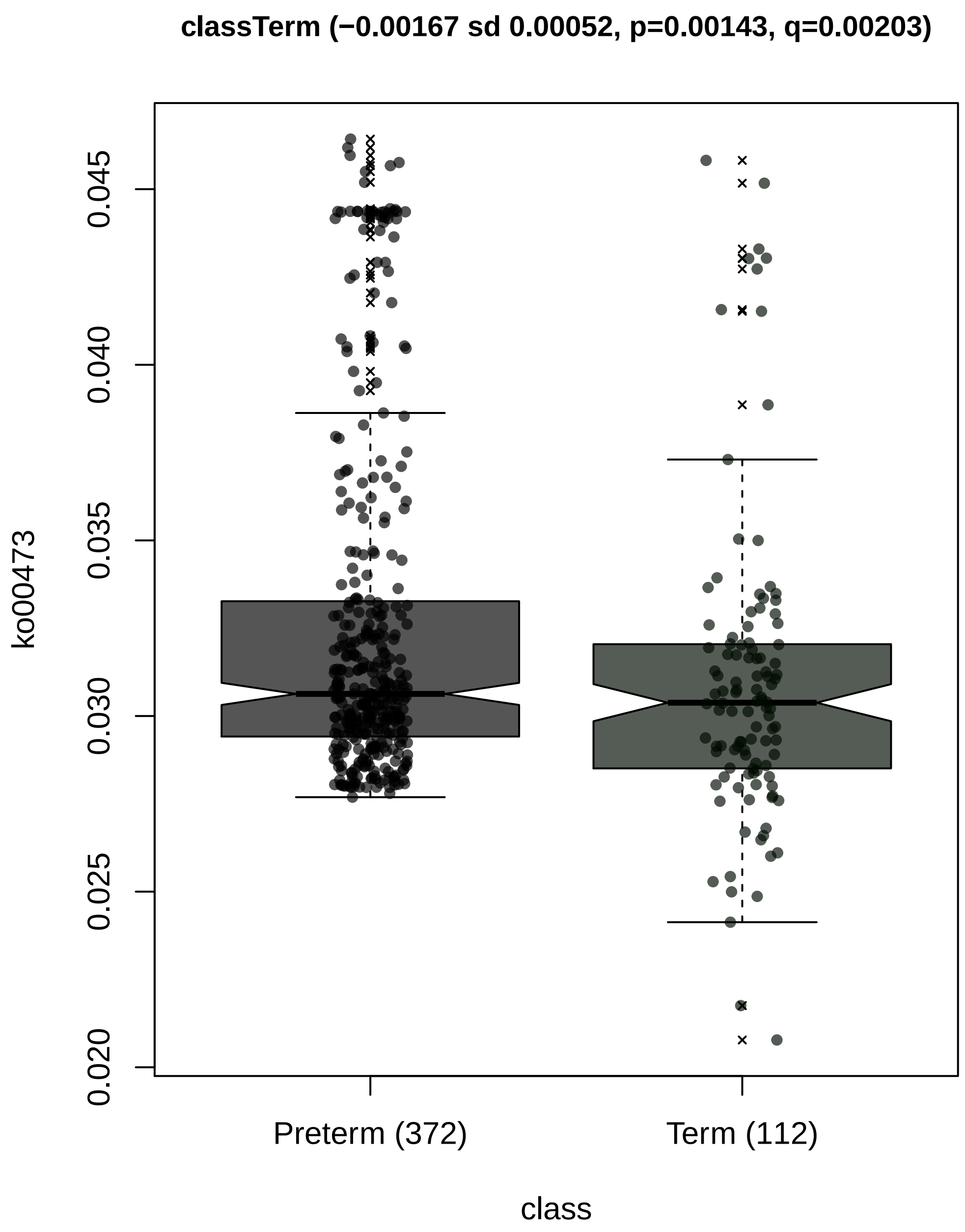
classTerm (−0.00167 sd 0.00052, p=0.00143, q=0.00203)
ko00473
0.020
0.025
0.030
0.035
0.040
0.045
Preterm (372)
Term (112)
class

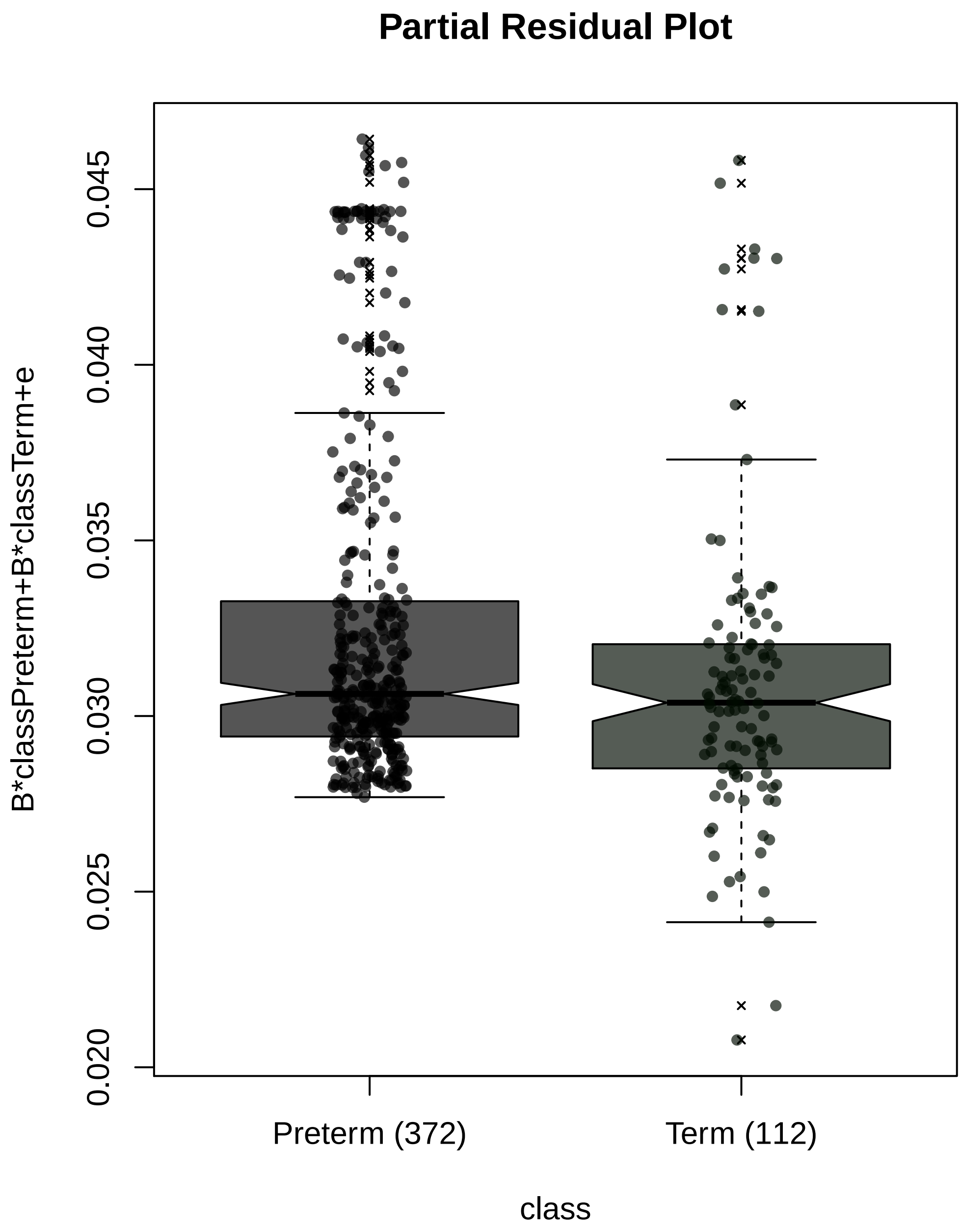
Partial Residual Plot
B*classPreterm+B*classTerm+e
0.020
0.025
0.030
0.035
0.040
0.045
Preterm (372)
Term (112)
class

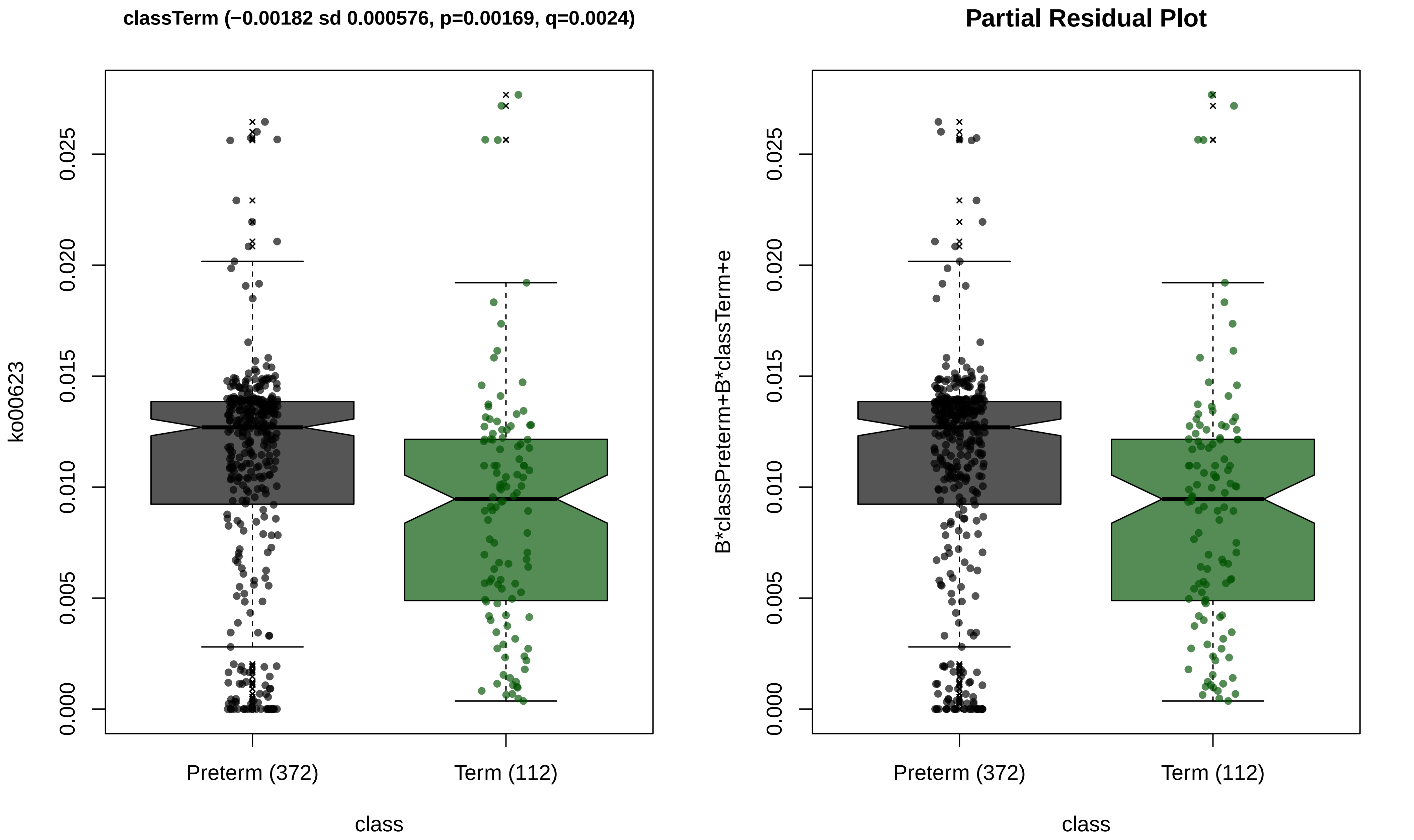

classTerm (−0.00182 sd 0.000576, p=0.00169, q=0.0024)
Partial Residual Plot
ko00623
B*classPreterm+B*classTerm+e
0.000
0.005
0.010
0.015
0.020
0.025
Preterm (372)
Term (112)
class

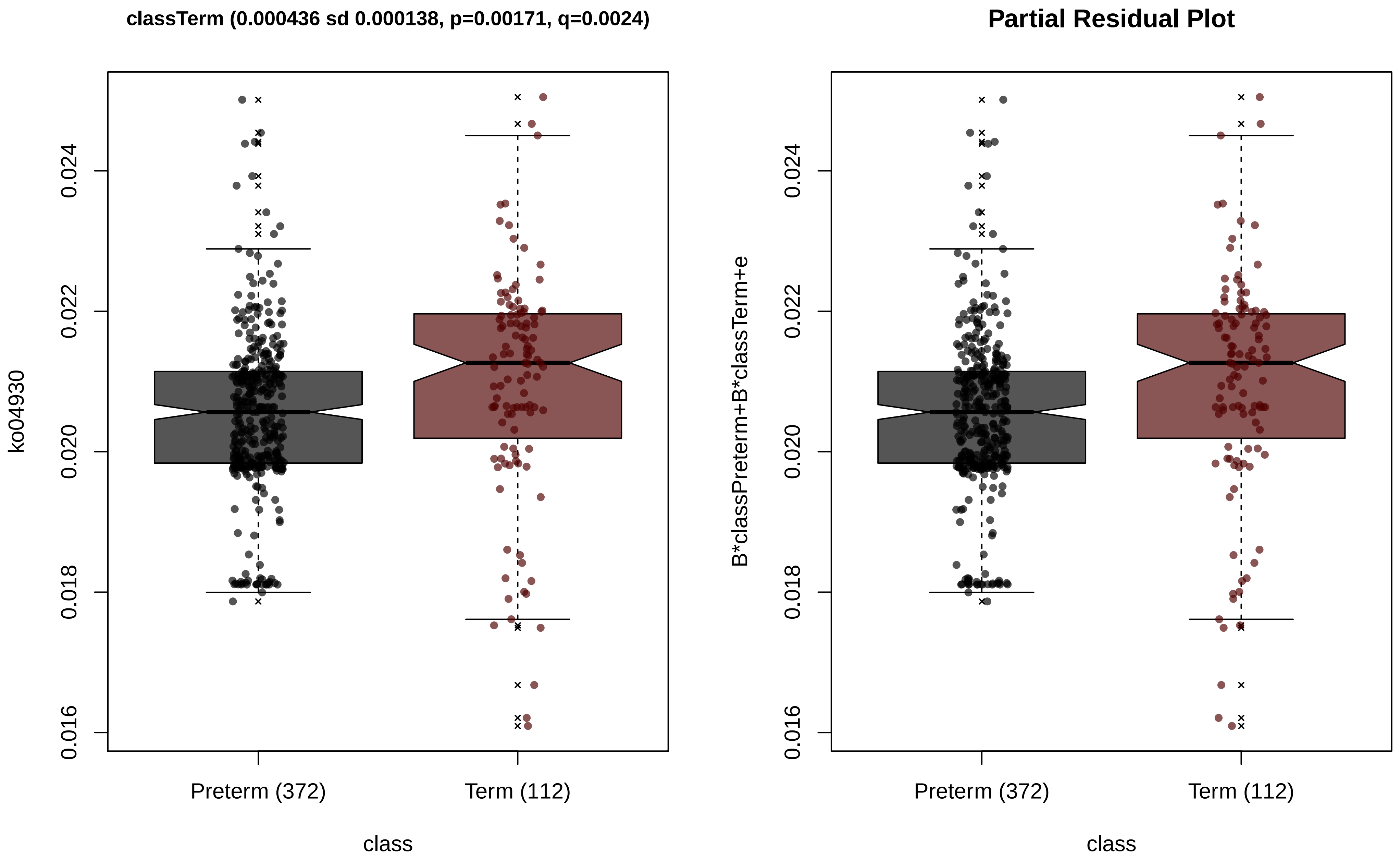

classTerm (0.000436 sd 0.000138, p=0.00171, q=0.0024)
ko04930
0.016
0.018
0.020
0.022
0.024
Preterm (372)
Term (112)
class
Partial Residual Plot
B*classPreterm+B*classTerm+e
0.016
0.018
0.020
0.022
0.024
Preterm (372)
Term (112)
class

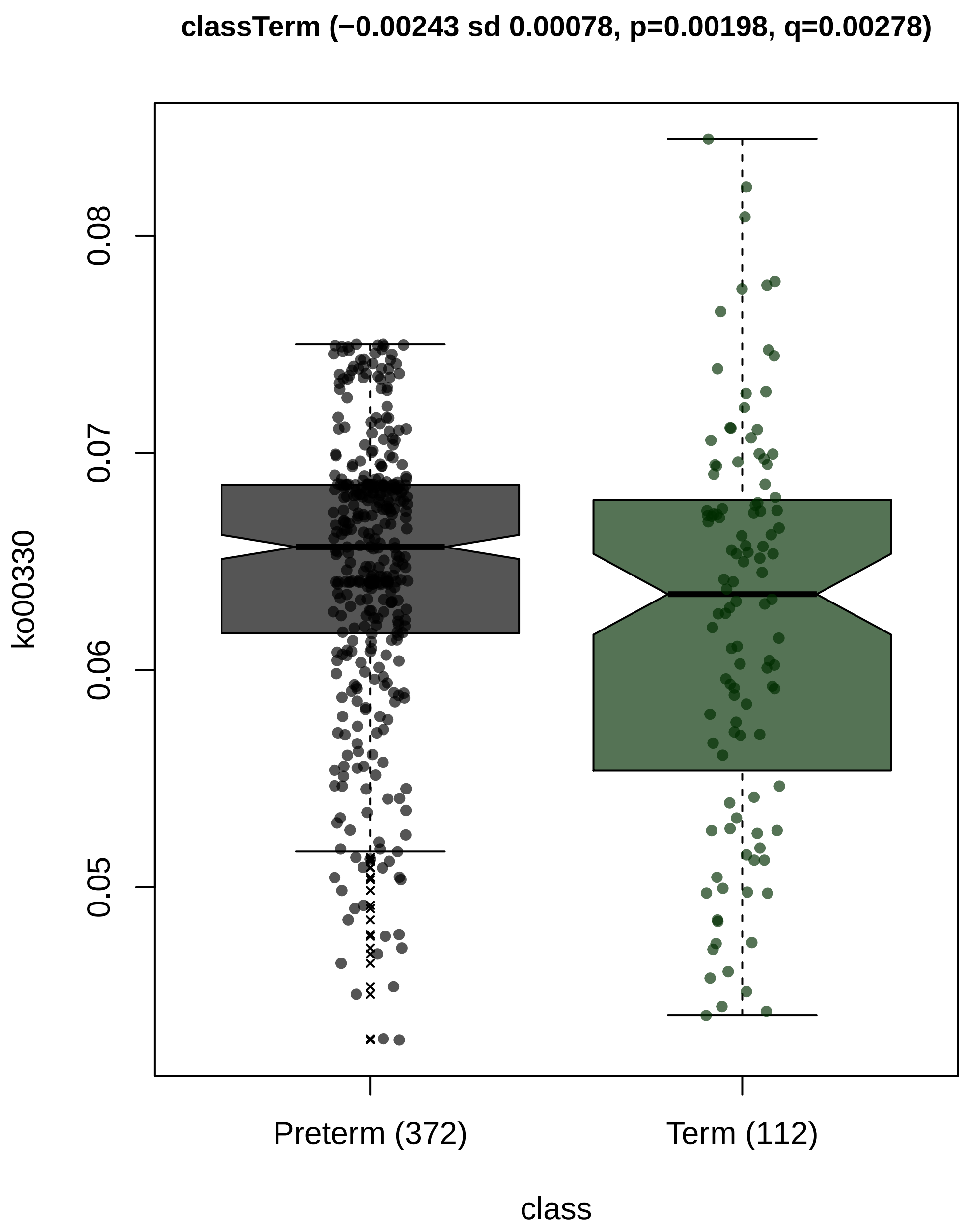

classTerm (−0.00243 sd 0.00078, p=0.00198, q=0.00278)
ko00330
0.08
0.07
0.06
0.05
Preterm (372)
Term (112)
class

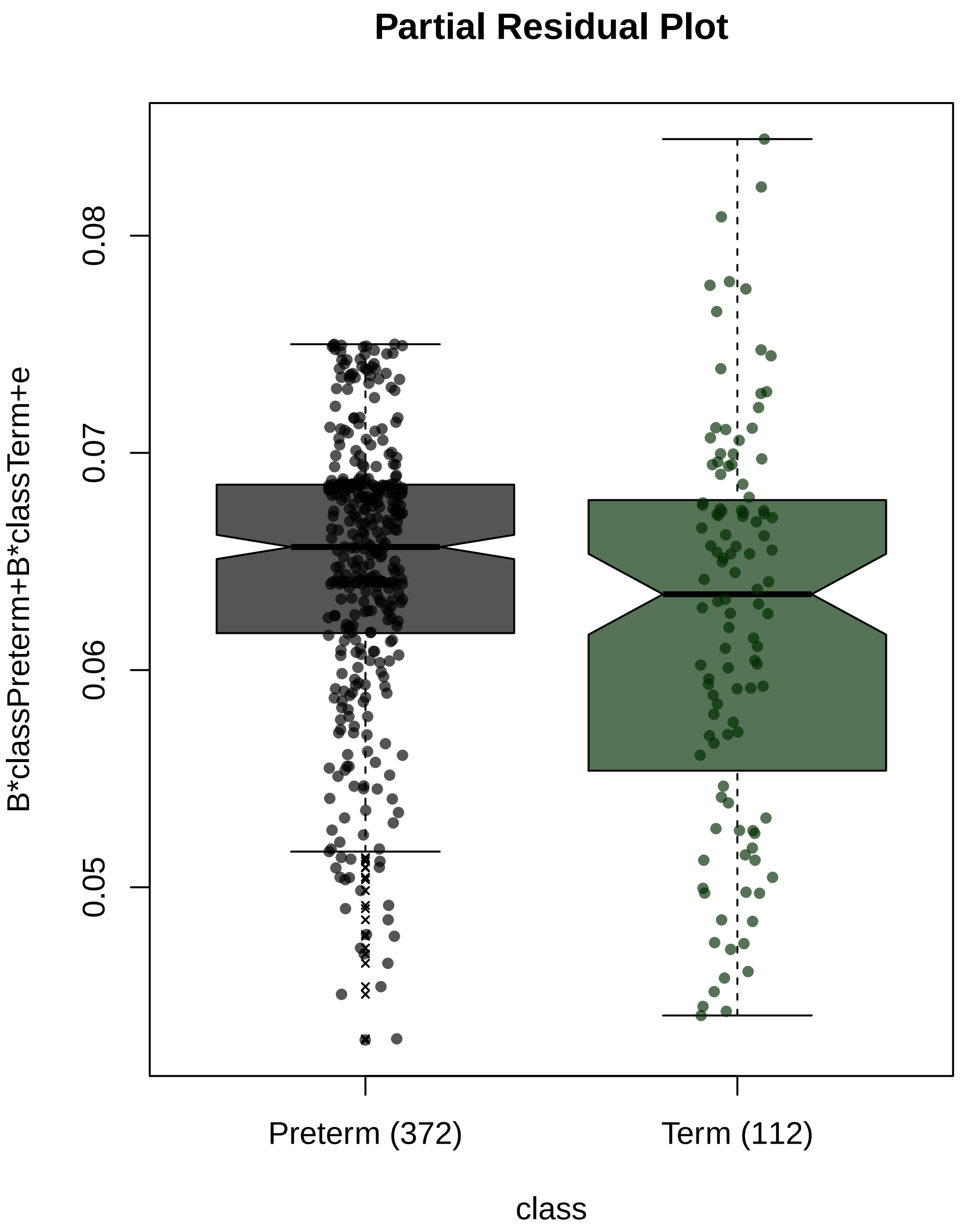

Partial Residual Plot
B*classPreterm+B*classTerm+e
0.08
0.07
0.06
0.05
Preterm (372)
Term (112)
class

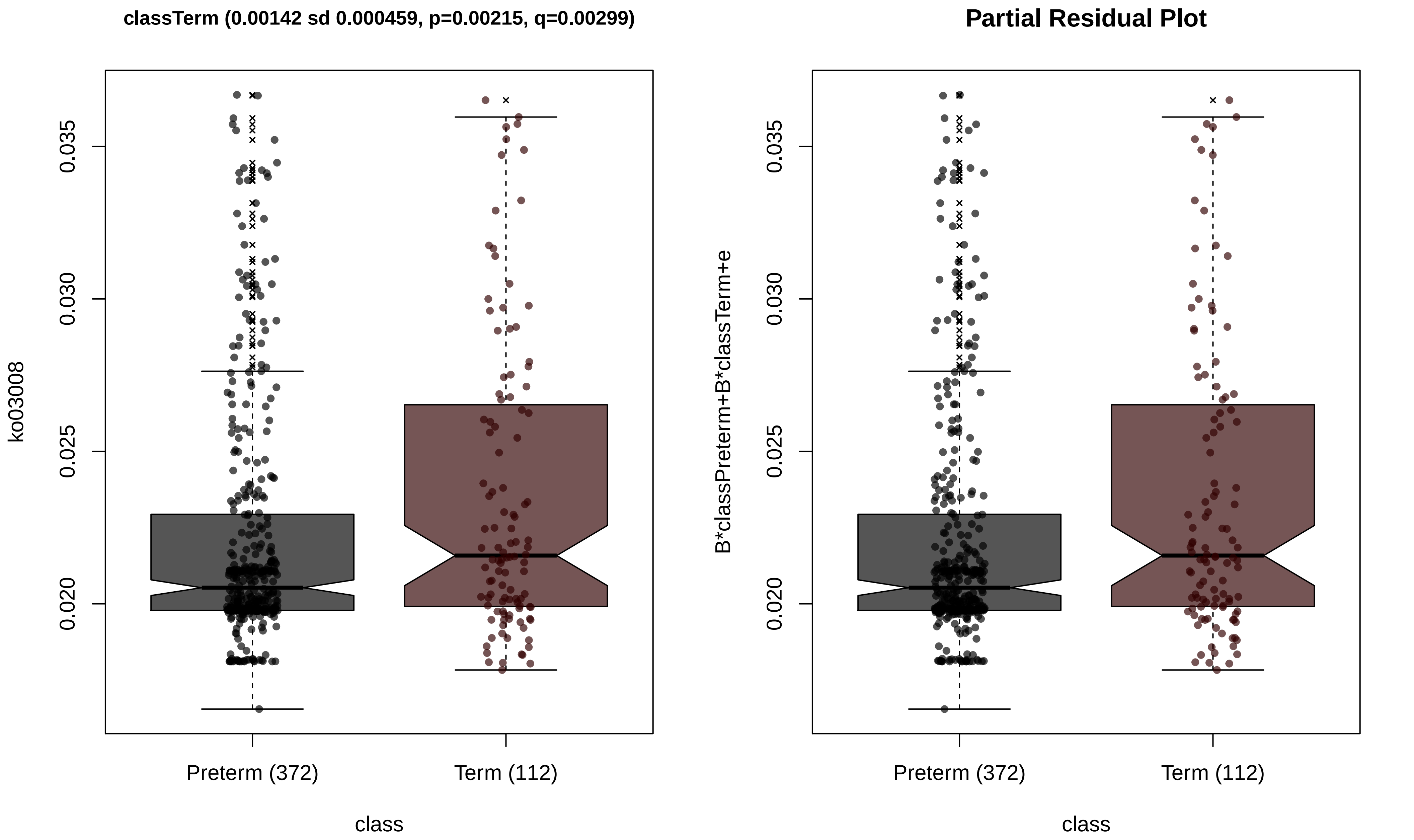

classTerm (0.00142 sd 0.000459, p=0.00215, q=0.00299)
ko03008
0.020
0.025
0.030
0.035
Preterm (372)
Term (112)
class
Partial Residual Plot
B*classPreterm+B*classTerm+e
0.020
0.025
0.030
0.035
Preterm (372)
Term (112)
class

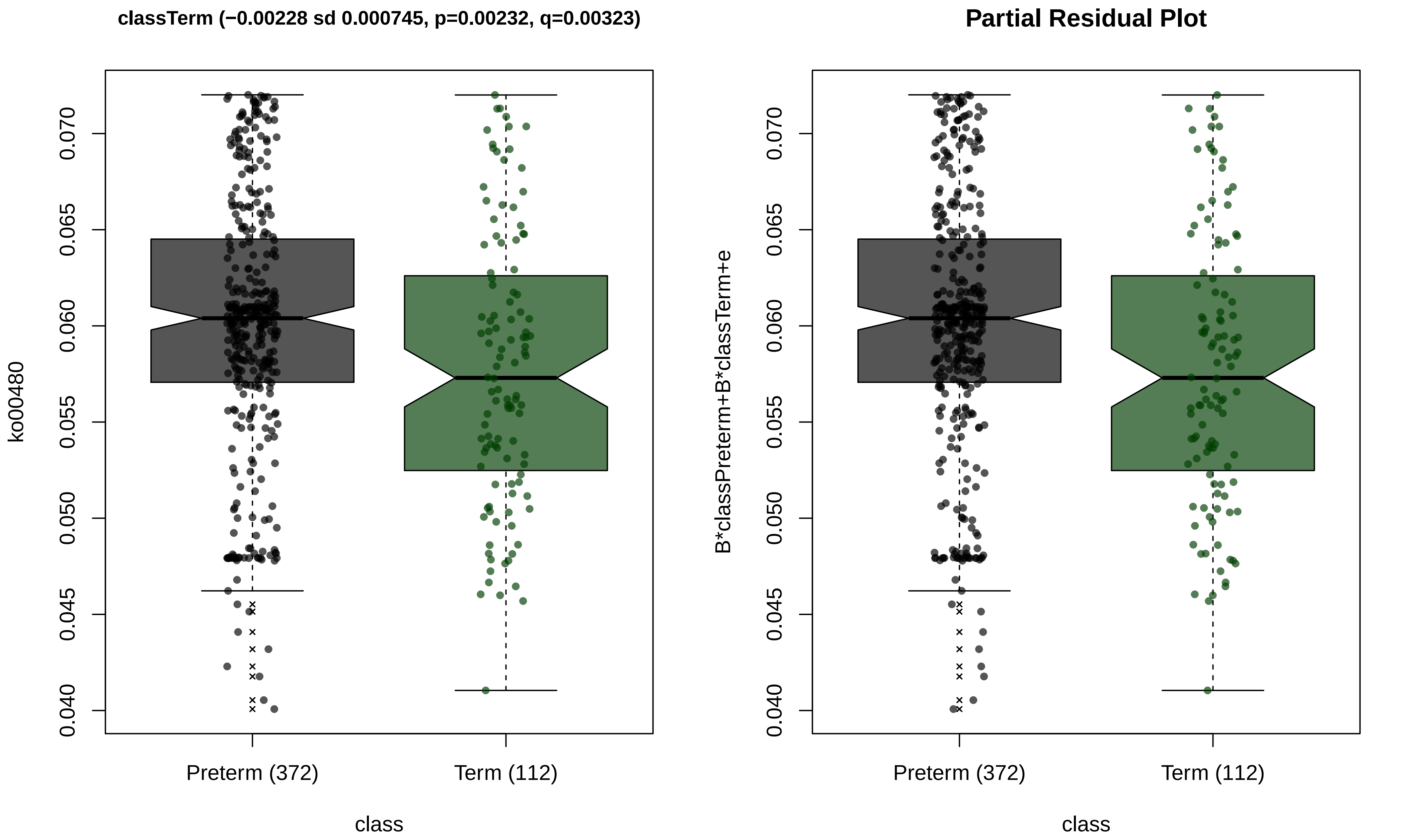
classTerm (−0.00228 sd 0.000745, p=0.00232, q=0.00323)
Partial Residual Plot
ko00480
B*classPreterm+B*classTerm+e
0.040
0.045
0.050
0.055
0.060
0.065
0.070
Preterm (372)
Term (112)
class

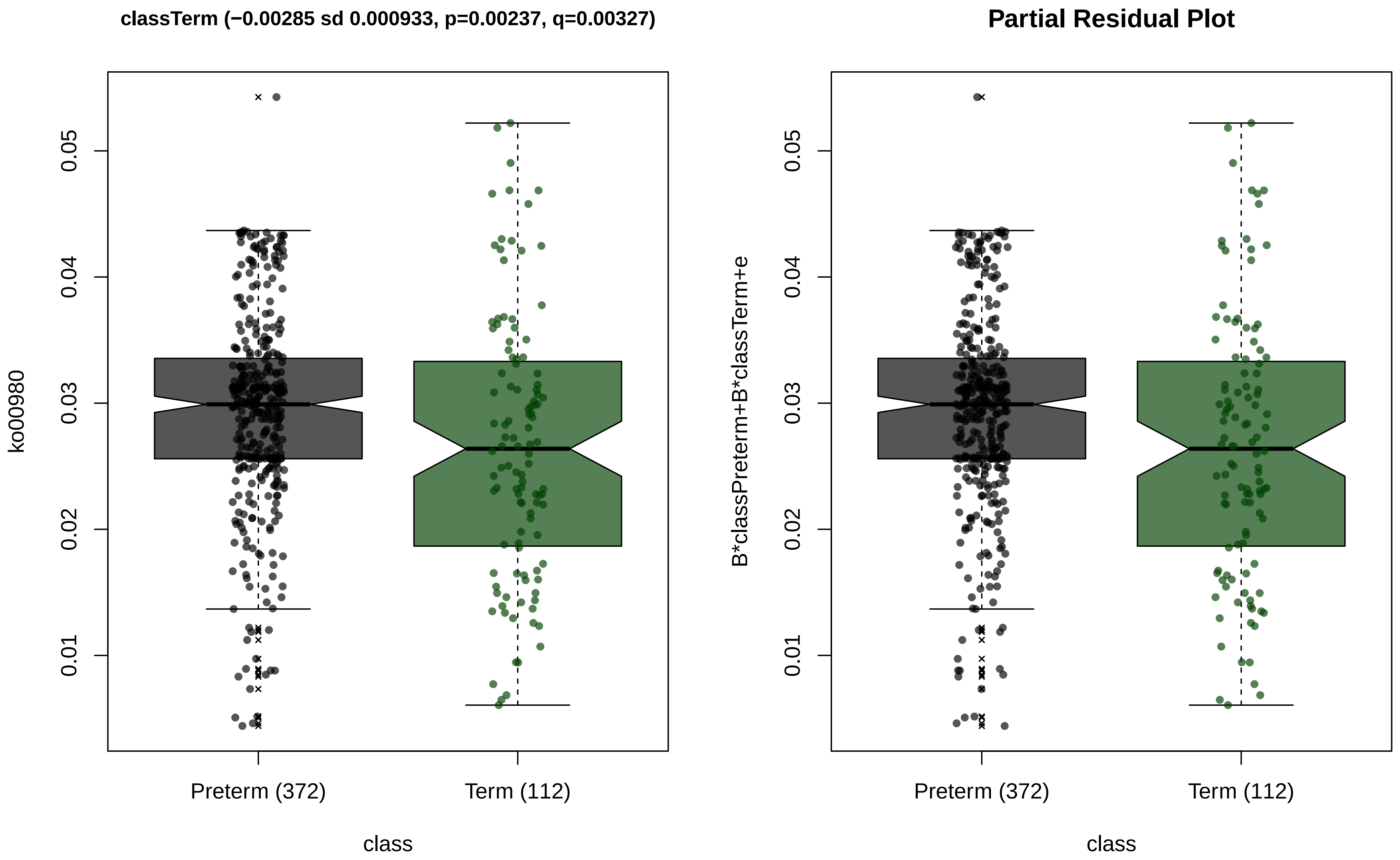

classTerm (−0.00285 sd 0.000933, p=0.00237, q=0.00327)
Partial Residual Plot
ko00980
B*classPreterm+B*classTerm+e
0.01
0.02
0.03
0.04
0.05
Preterm (372)
Term (112)
class

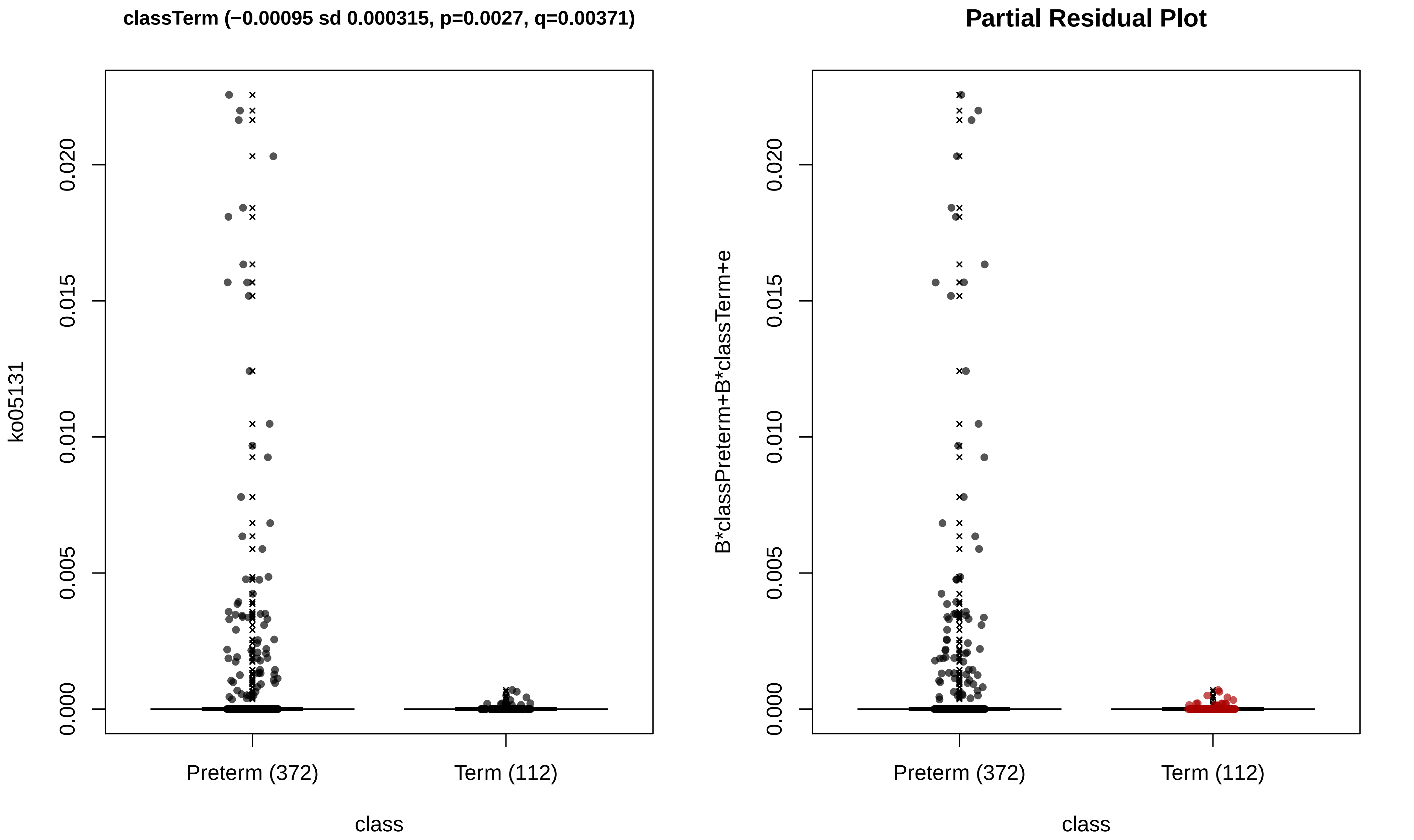
classTerm (−0.00095 sd 0.000315, p=0.0027, q=0.00371)
ko05131
0.000
0.005
0.010
0.015
0.020
Preterm (372)
Term (112)
class
Partial Residual Plot
B*classPreterm+B*classTerm+e
0.000
0.005
0.010
0.015
0.020
Preterm (372)
Term (112)
class

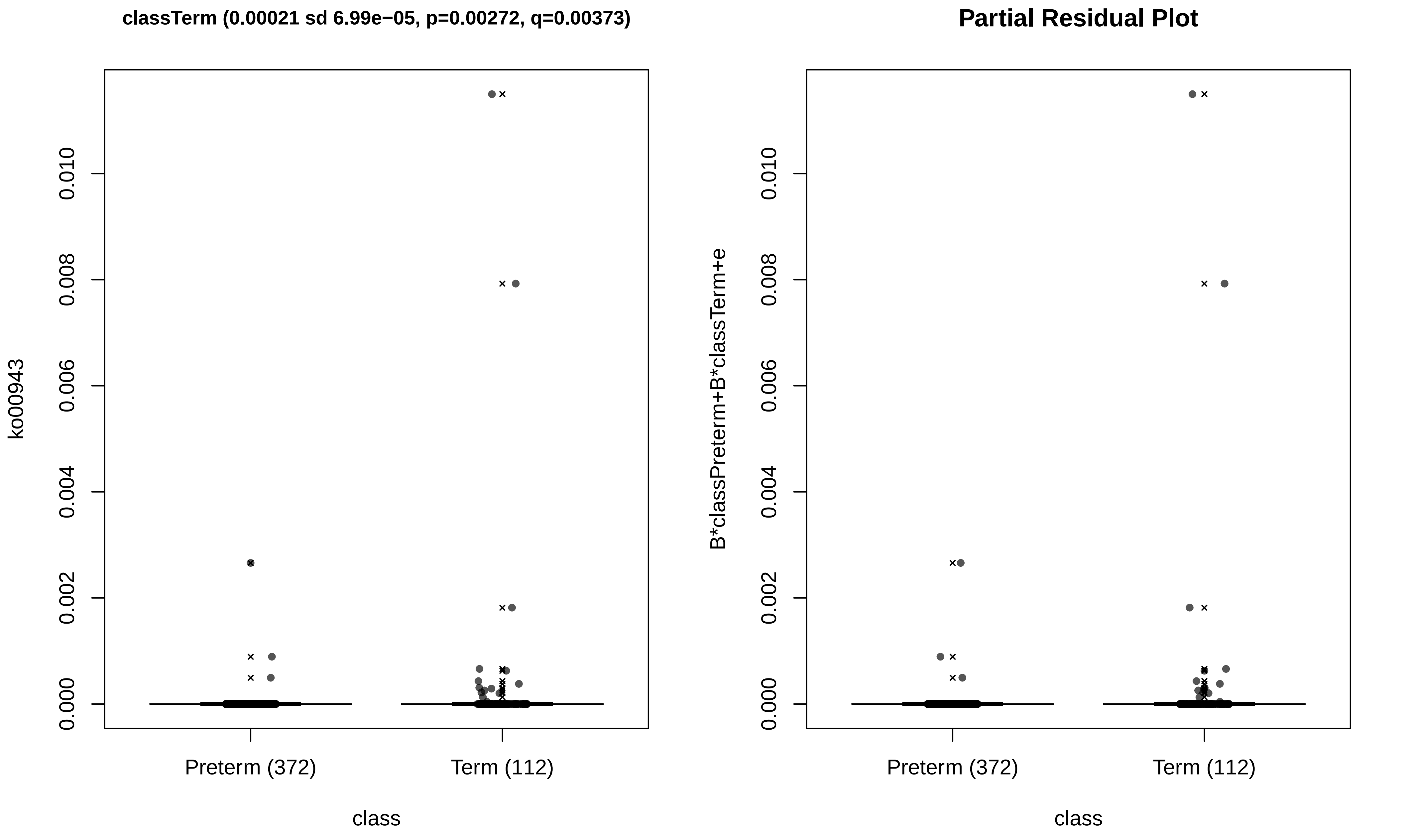
classTerm (0.00021 sd 6.99e−05, p=0.00272, q=0.00373)
Partial Residual Plot
ko00943
B*classPreterm+B*classTerm+e
0.000
0.002
0.004
0.006
0.008
0.010
Preterm (372)
Term (112)
class

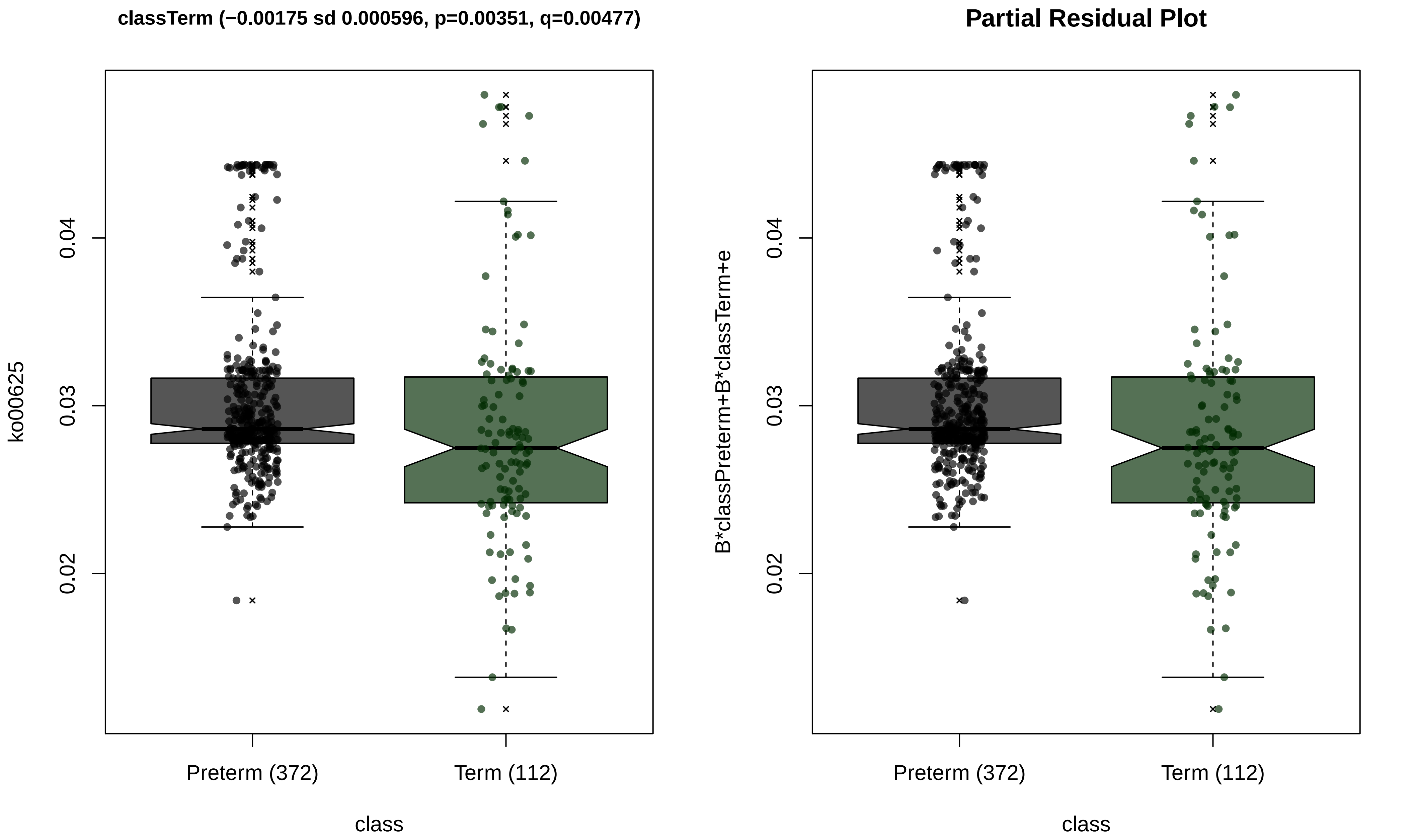

classTerm (−0.00175 sd 0.000596, p=0.00351, q=0.00477)
ko00625
0.02
0.03
0.04
Preterm (372)
Term (112)
class
Partial Residual Plot
B*classPreterm+B*classTerm+e
0.02
0.03
0.04
Preterm (372)
Term (112)
class

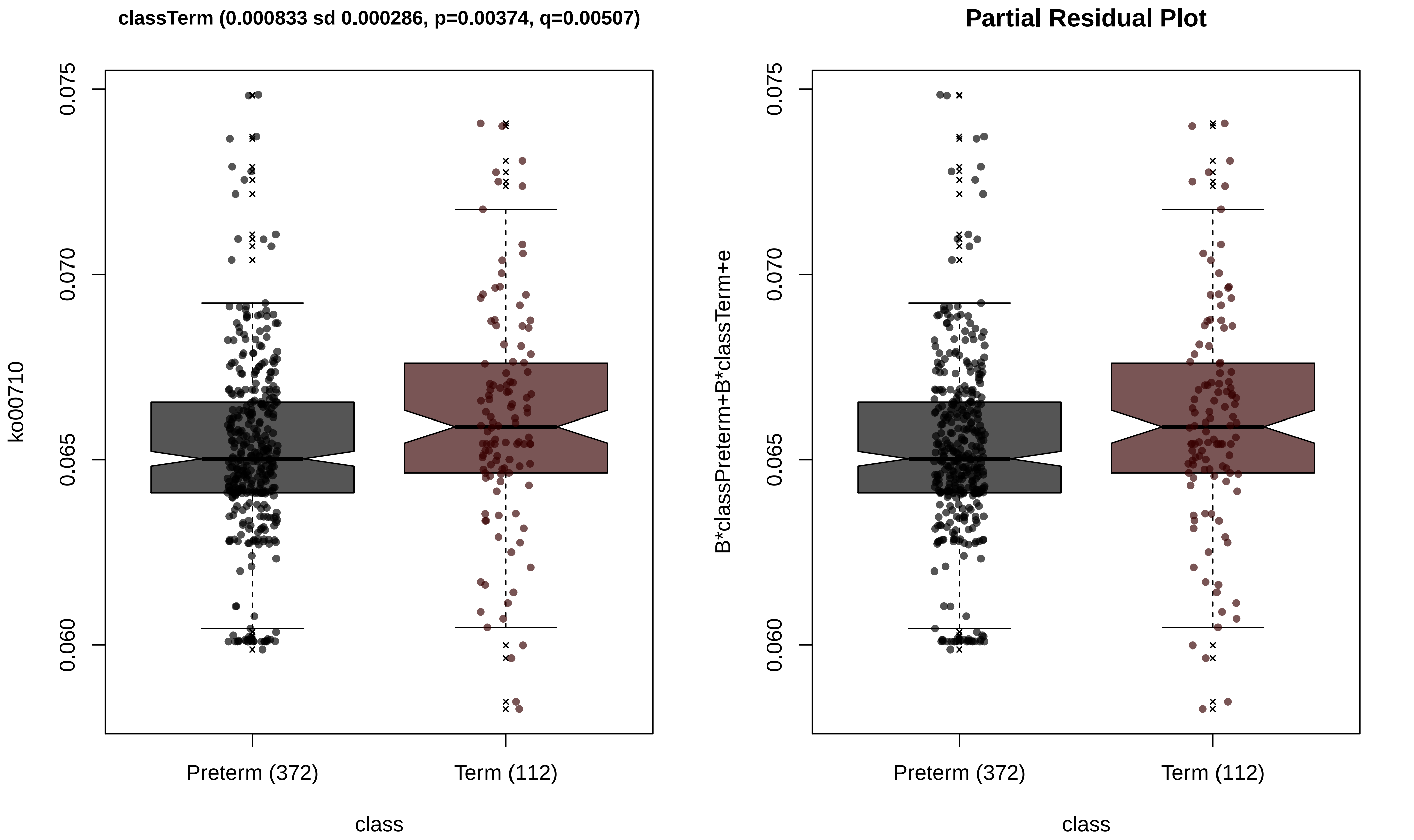
classTerm (0.000833 sd 0.000286, p=0.00374, q=0.00507)
Partial Residual Plot
ko00710
B*classPreterm+B*classTerm+e
0.075
0.070
0.065
0.060
Preterm (372)
Term (112)
class

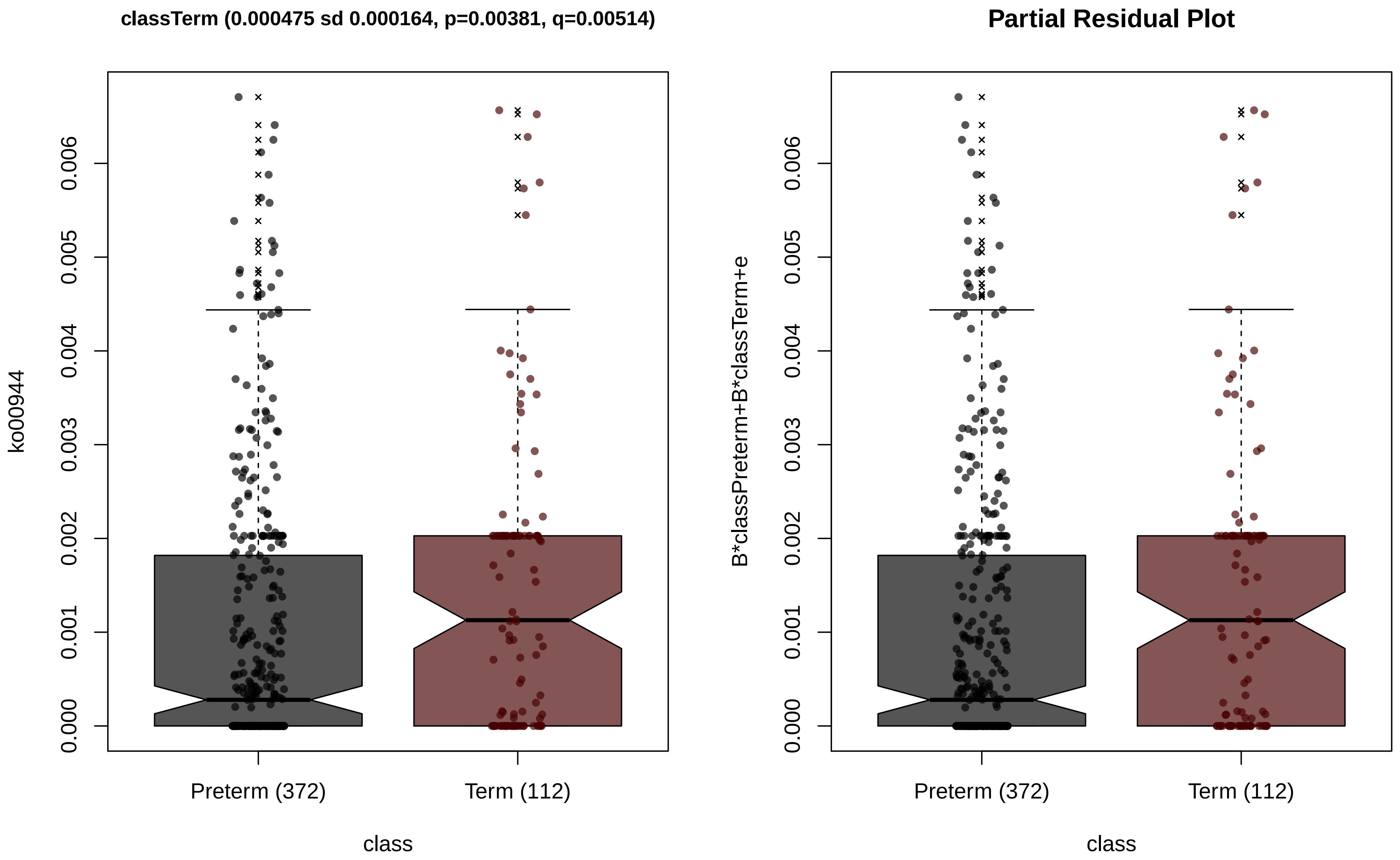

classTerm (0.000475 sd 0.000164, p=0.00381, q=0.00514)
ko00944
0.000
0.001
0.002
0.003
0.004
0.005
0.006
Preterm (372)
Term (112)
class
Partial Residual Plot
B*classPreterm+B*classTerm+e
0.000
0.001
0.002
0.003
0.004
0.005
0.006
Preterm (372)
Term (112)
class

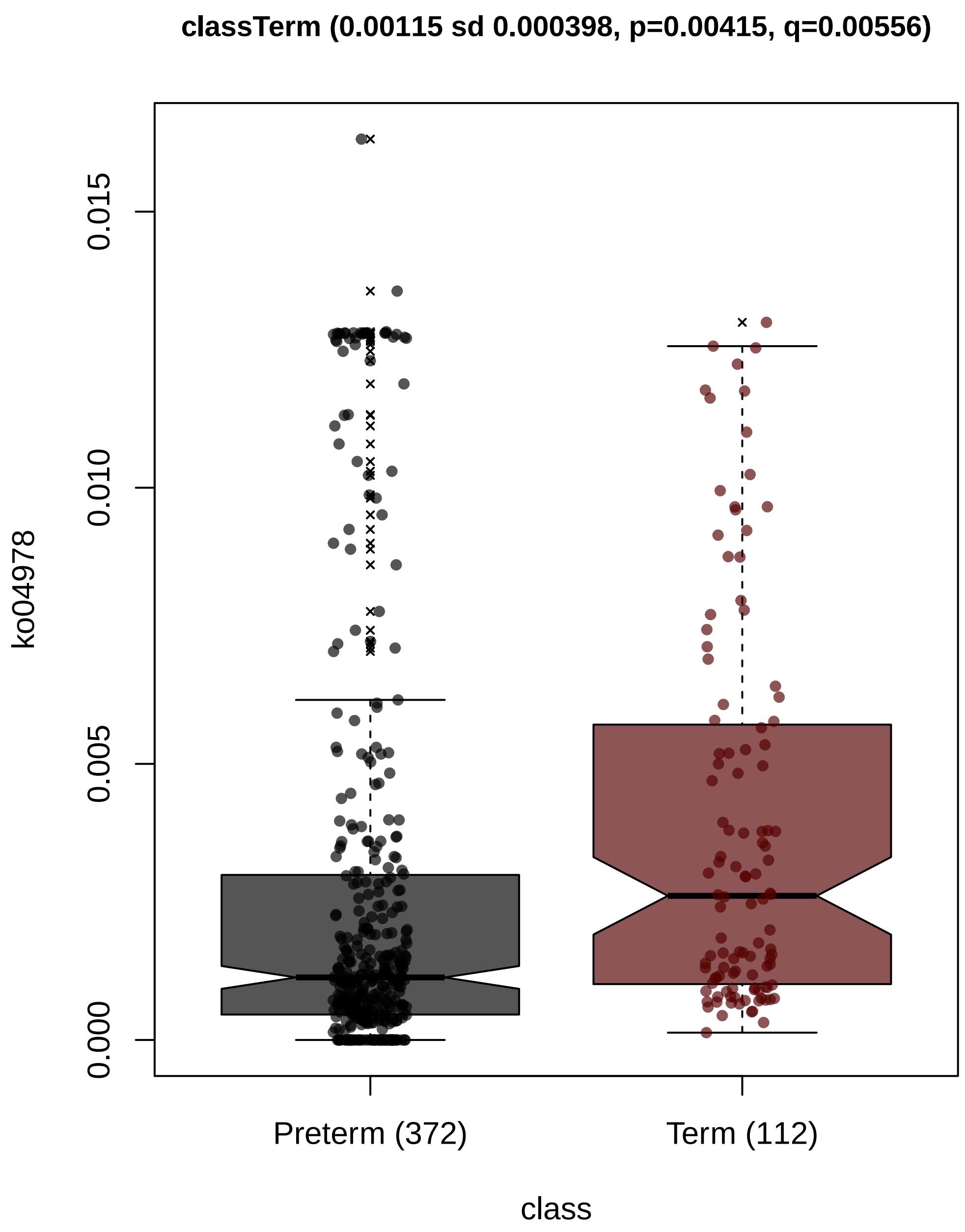

classTerm (0.00115 sd 0.000398, p=0.00415, q=0.00556)
ko04978
0.000
0.005
0.010
0.015
Preterm (372)
Term (112)
class

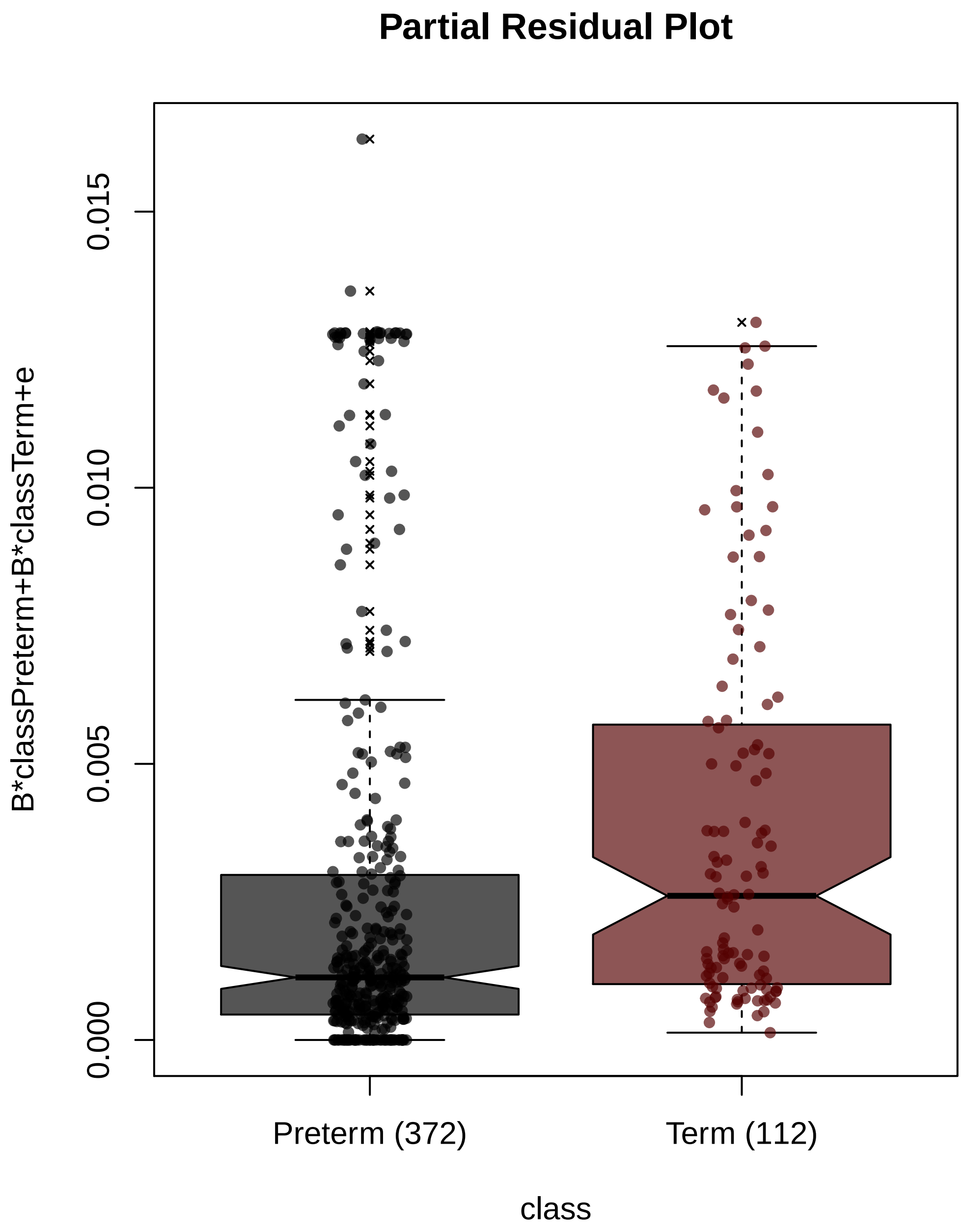

Partial Residual Plot
B*classPreterm+B*classTerm+e
0.000
0.005
0.010
0.015
Preterm (372)
Term (112)
class

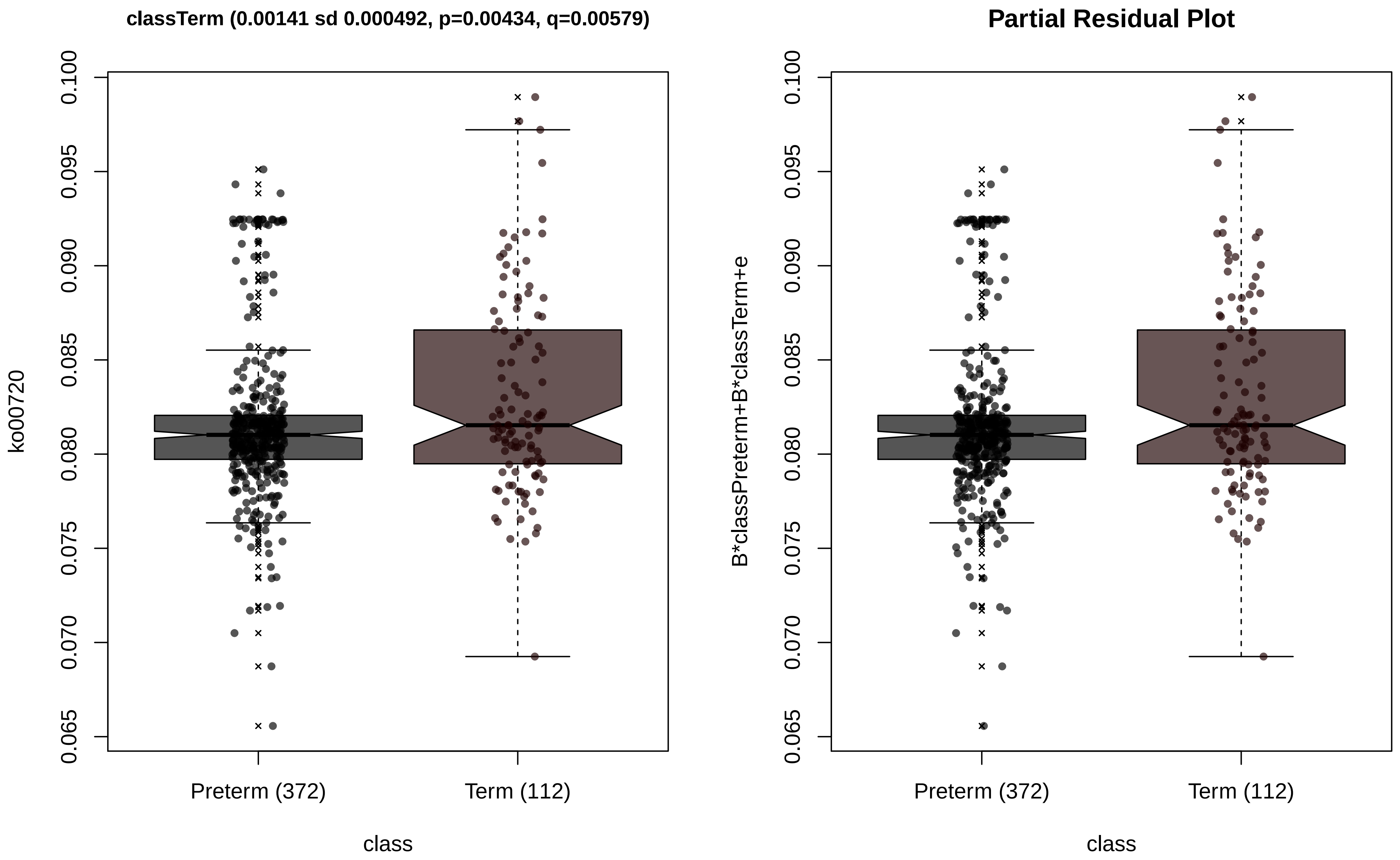

classTerm (0.00141 sd 0.000492, p=0.00434, q=0.00579)
Partial Residual Plot
ko00720
B*classPreterm+B*classTerm+e
0.065
0.070
0.075
0.080
0.085
0.090
0.095
0.100
Preterm (372)
Term (112)
class

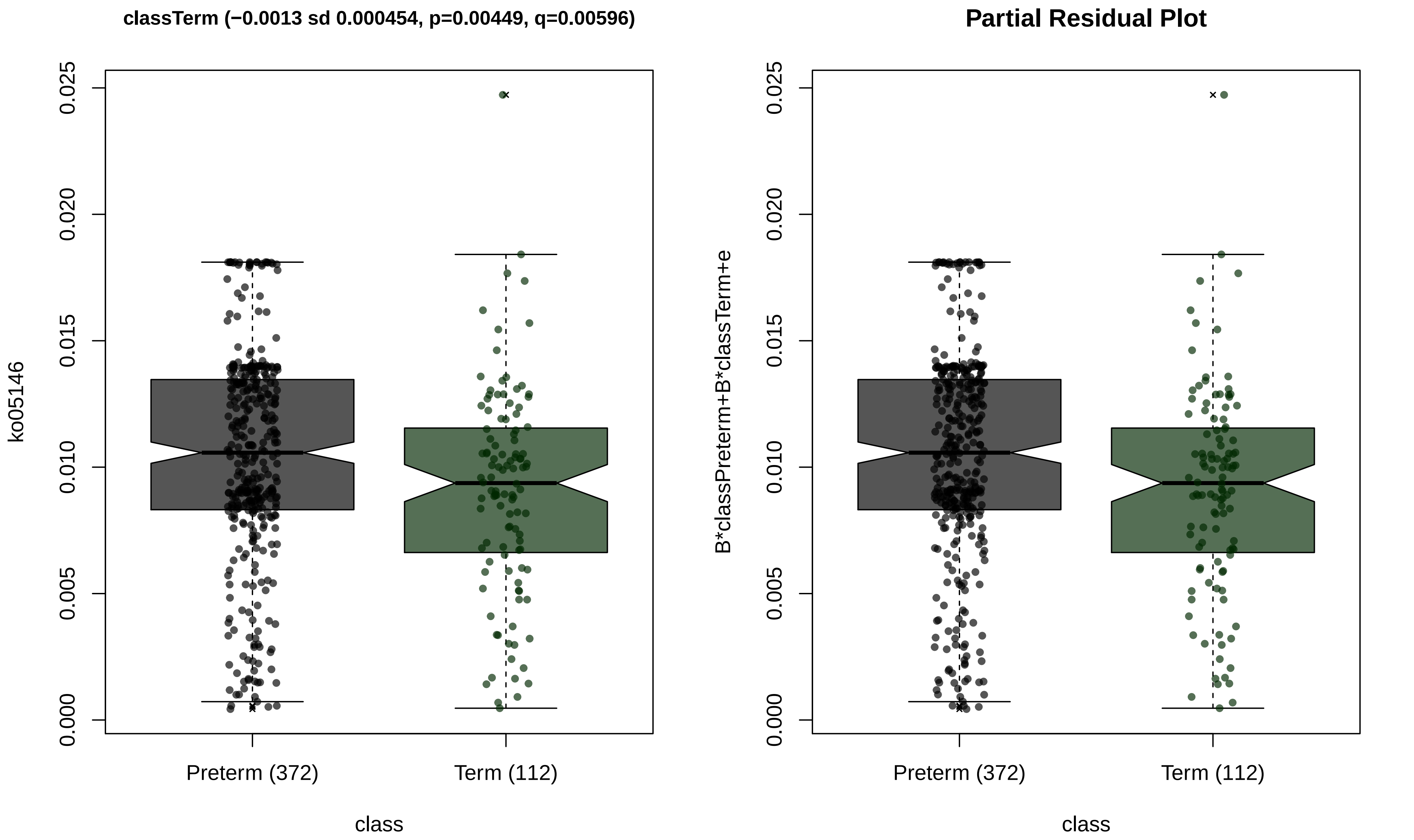
classTerm (−0.0013 sd 0.000454, p=0.00449, q=0.00596)
Partial Residual Plot
ko05146
B*classPreterm+B*classTerm+e
0.000
0.005
0.010
0.015
0.020
0.025
Preterm (372)
Term (112)
class

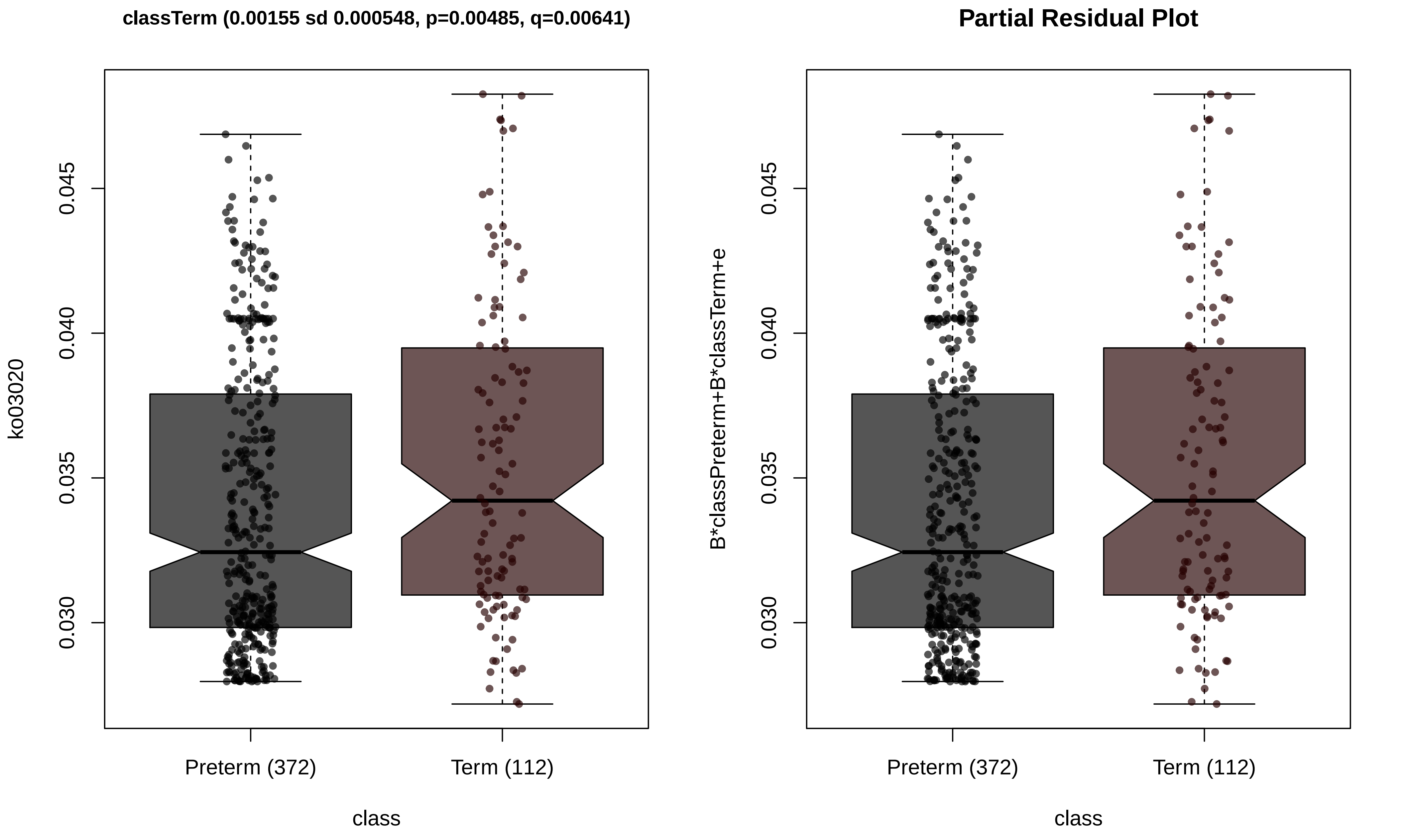
classTerm (0.00155 sd 0.000548, p=0.00485, q=0.00641)
ko03020
0.030
0.035
0.040
0.045
Preterm (372)
Term (112)
class
Partial Residual Plot
B*classPreterm+B*classTerm+e
0.030
0.035
0.040
0.045
Preterm (372)
Term (112)
class

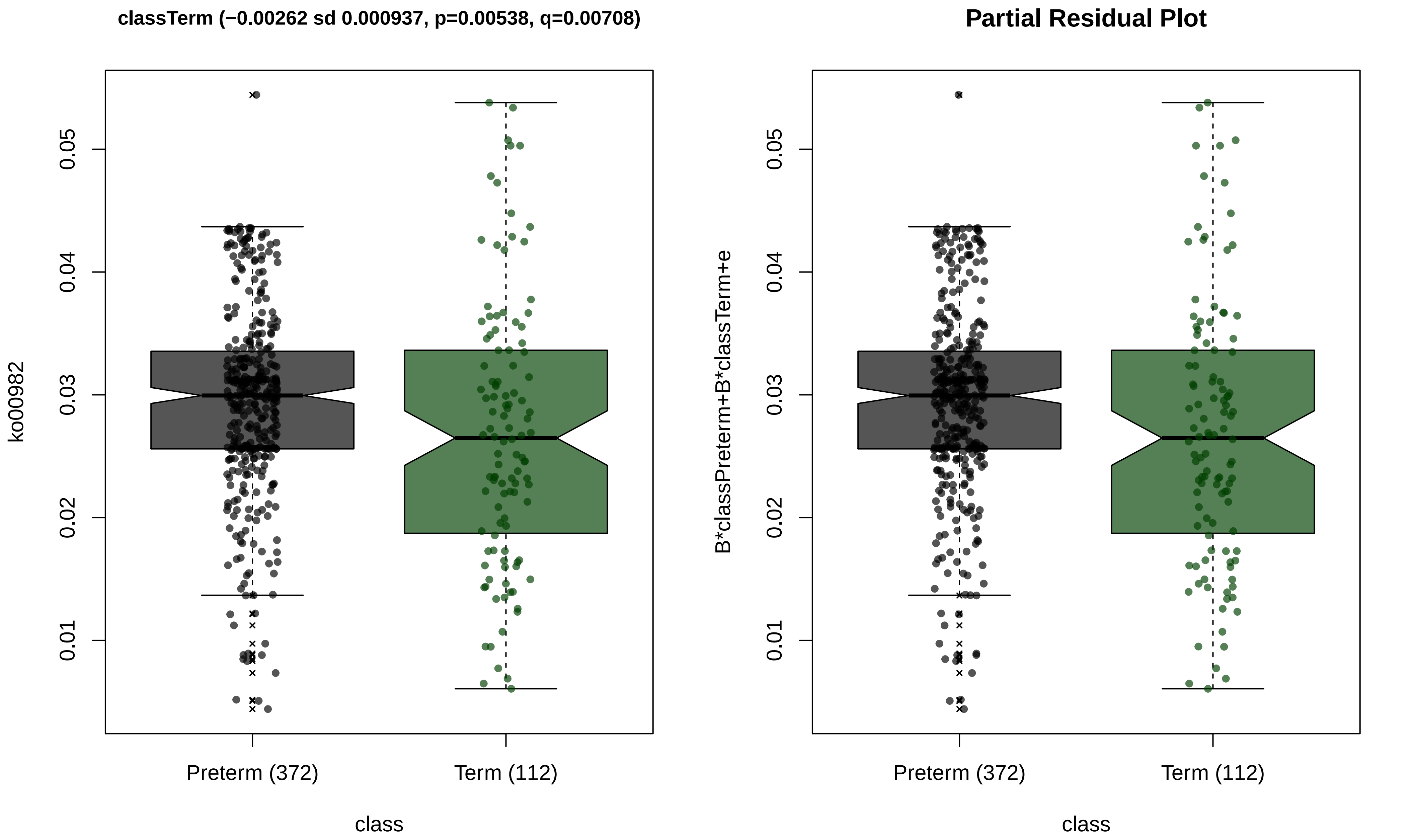

classTerm (−0.00262 sd 0.000937, p=0.00538, q=0.00708)
Partial Residual Plot
ko00982
B*classPreterm+B*classTerm+e
0.01
0.02
0.03
0.04
0.05
Preterm (372)
Term (112)
class

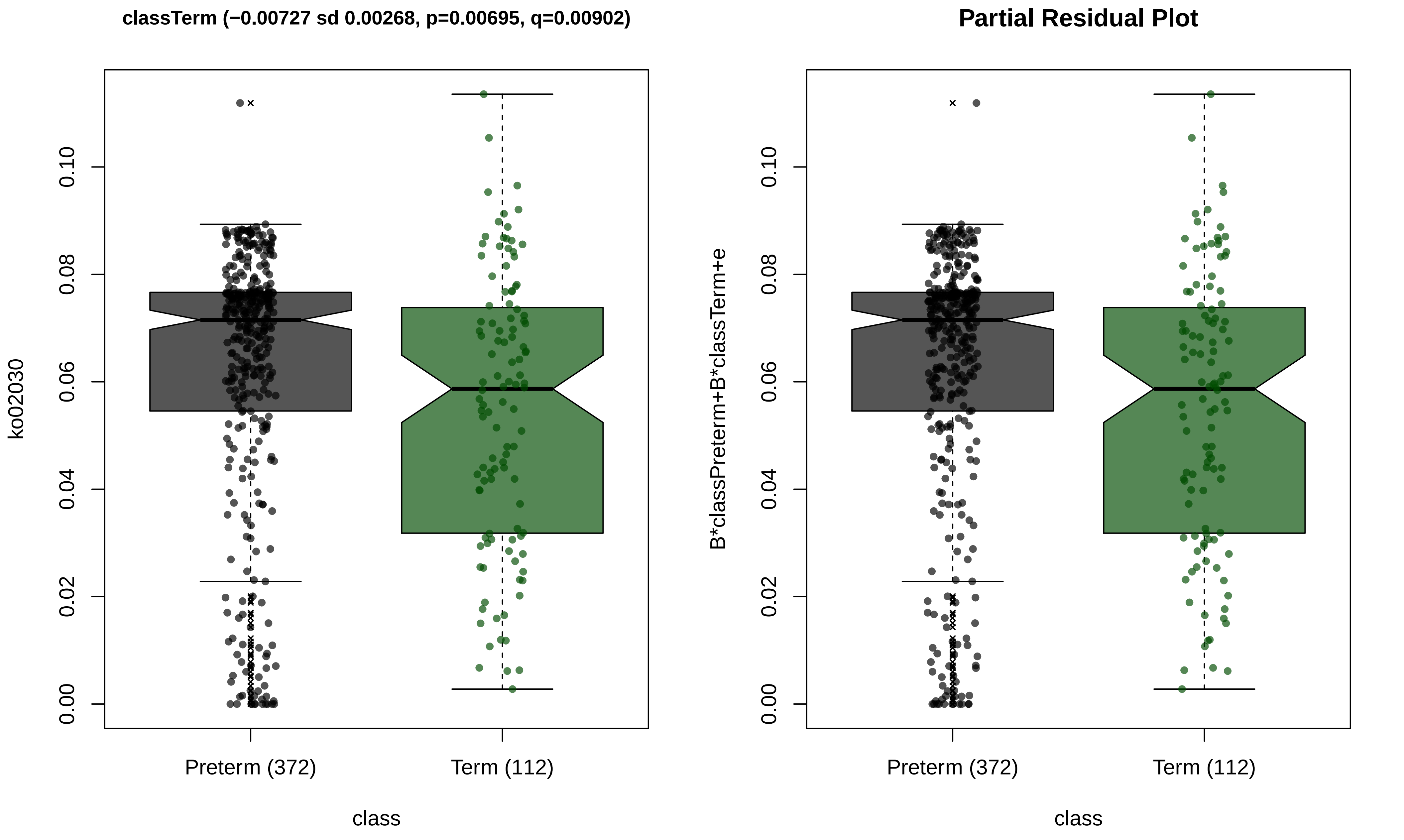

classTerm (−0.00727 sd 0.00268, p=0.00695, q=0.00902)
Partial Residual Plot
ko02030
B*classPreterm+B*classTerm+e
0.00
0.02
0.04
0.06
0.08
0.10
Preterm (372)
Term (112)
class

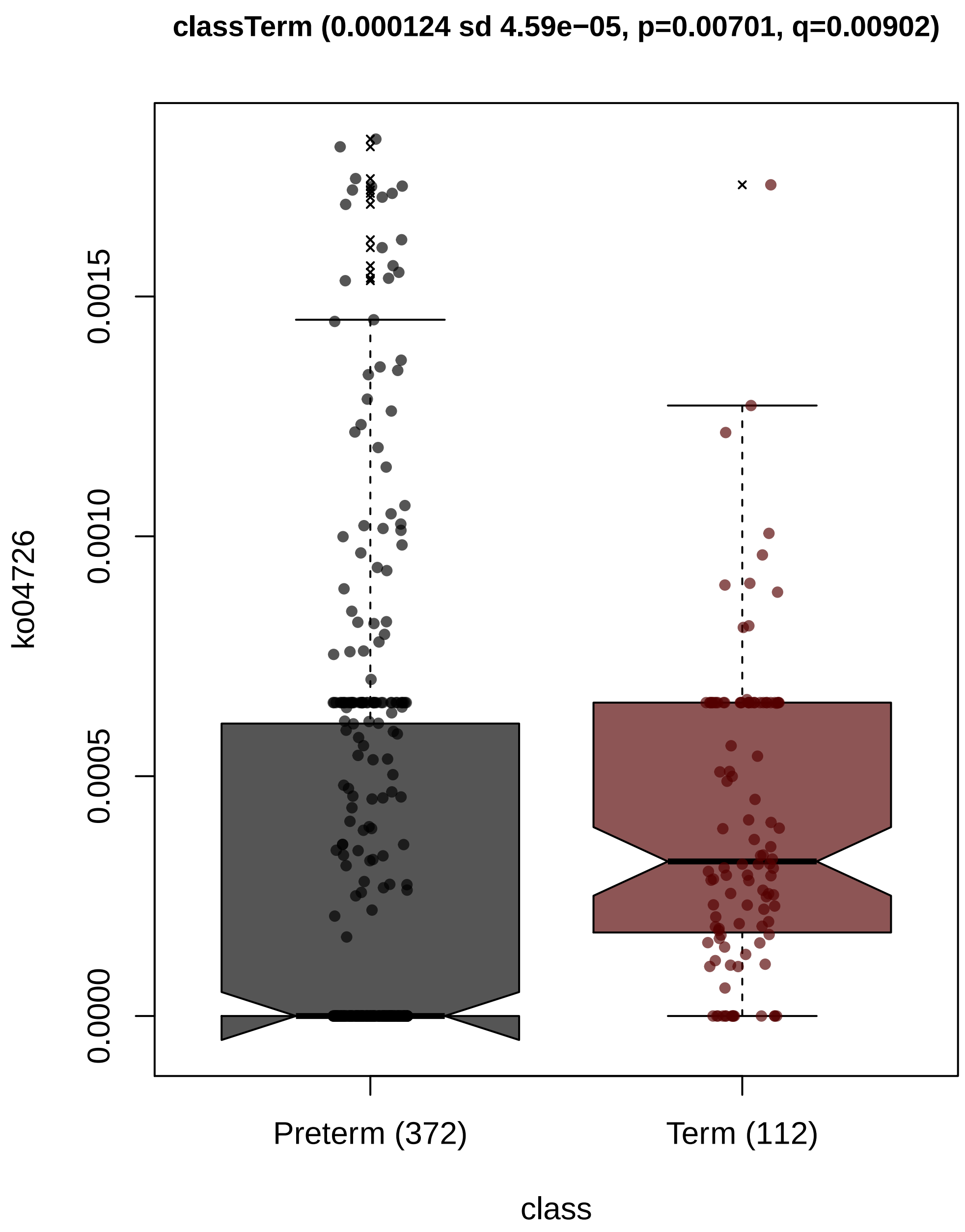
classTerm (0.000124 sd 4.59e−05, p=0.00701, q=0.00902)
ko04726
0.0000
0.0005
0.0010
0.0015
Preterm (372)
Term (112)
class

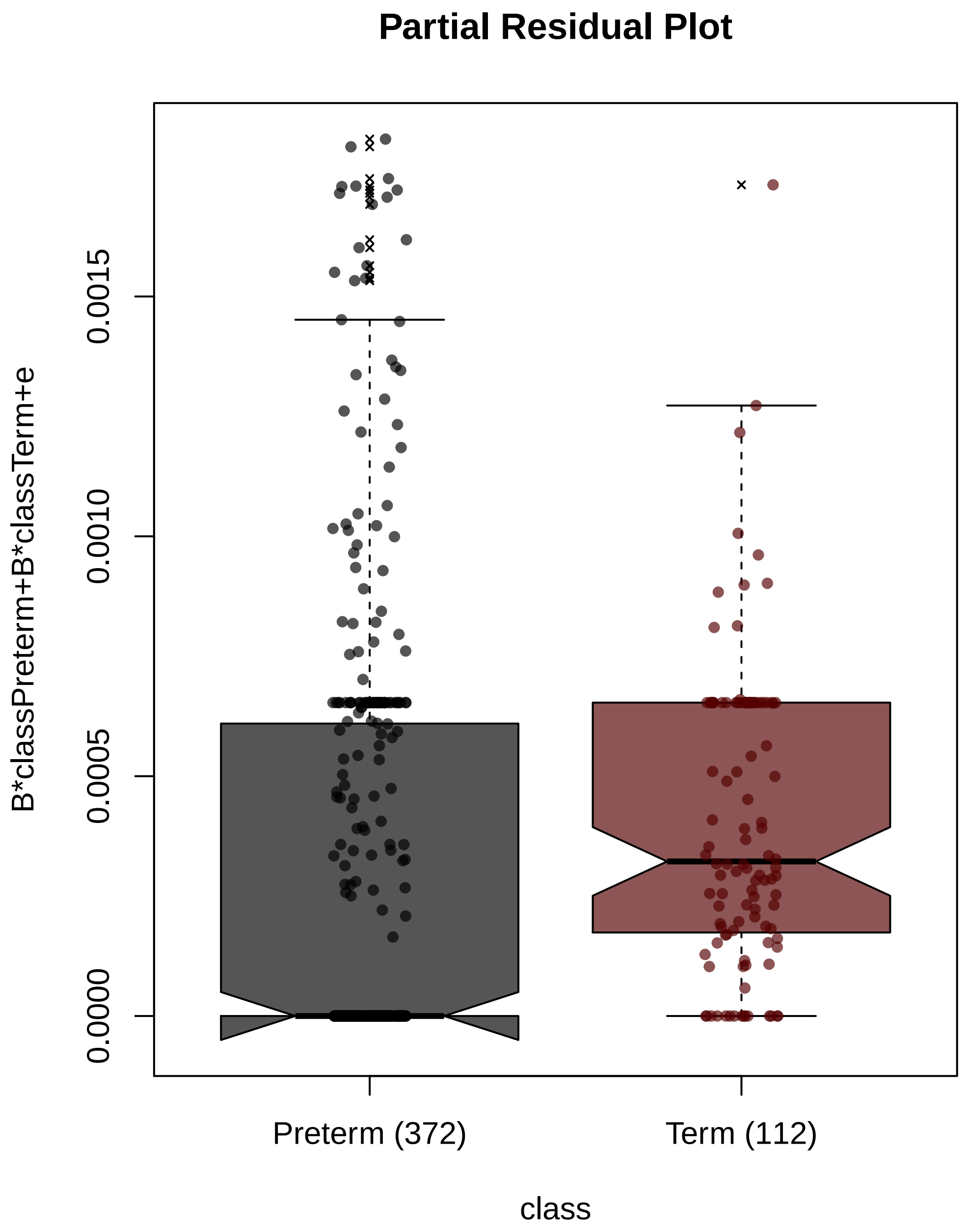
Partial Residual Plot
B*classPreterm+B*classTerm+e
0.0000
0.0005
0.0010
0.0015
Preterm (372)
Term (112)
class

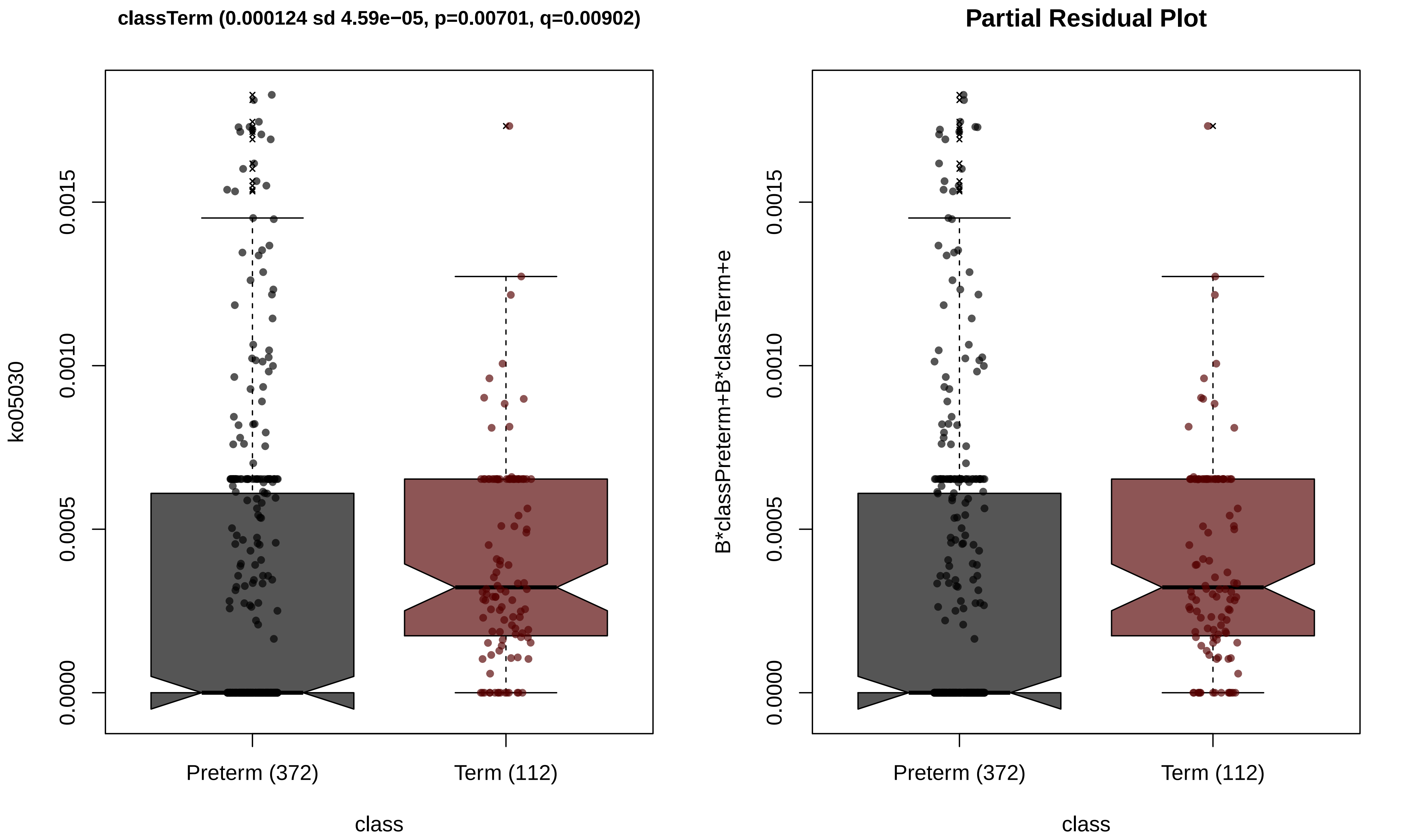
classTerm (0.000124 sd 4.59e−05, p=0.00701, q=0.00902)
ko05030
0.0000
0.0005
0.0010
0.0015
Preterm (372)
Term (112)
class
Partial Residual Plot
B*classPreterm+B*classTerm+e
0.0000
0.0005
0.0010
0.0015
Preterm (372)
Term (112)
class

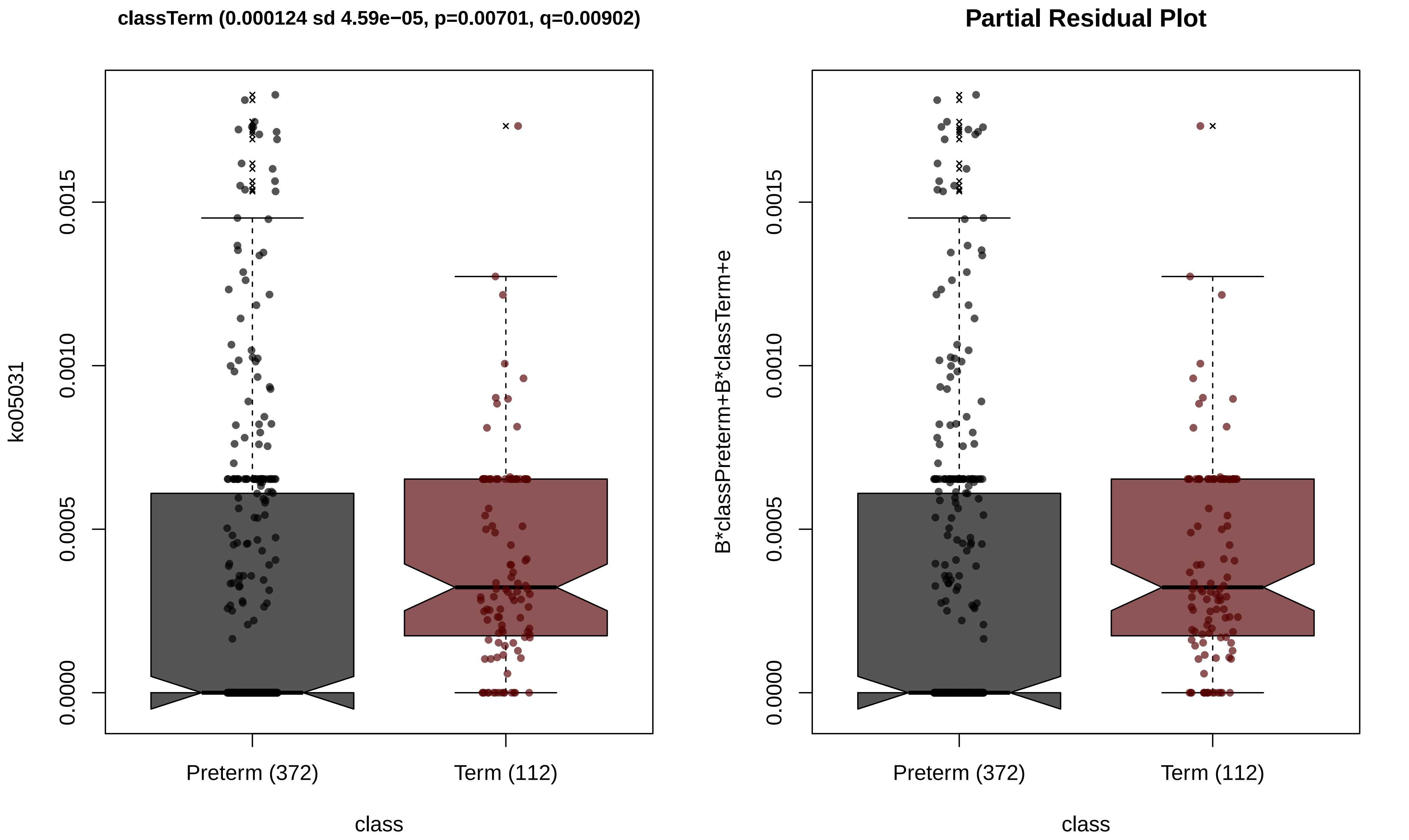

classTerm (0.000124 sd 4.59e−05, p=0.00701, q=0.00902)
ko05031
0.0000
0.0005
0.0010
0.0015
Preterm (372)
Term (112)
class
Partial Residual Plot
B*classPreterm+B*classTerm+e
0.0000
0.0005
0.0010
0.0015
Preterm (372)
Term (112)
class

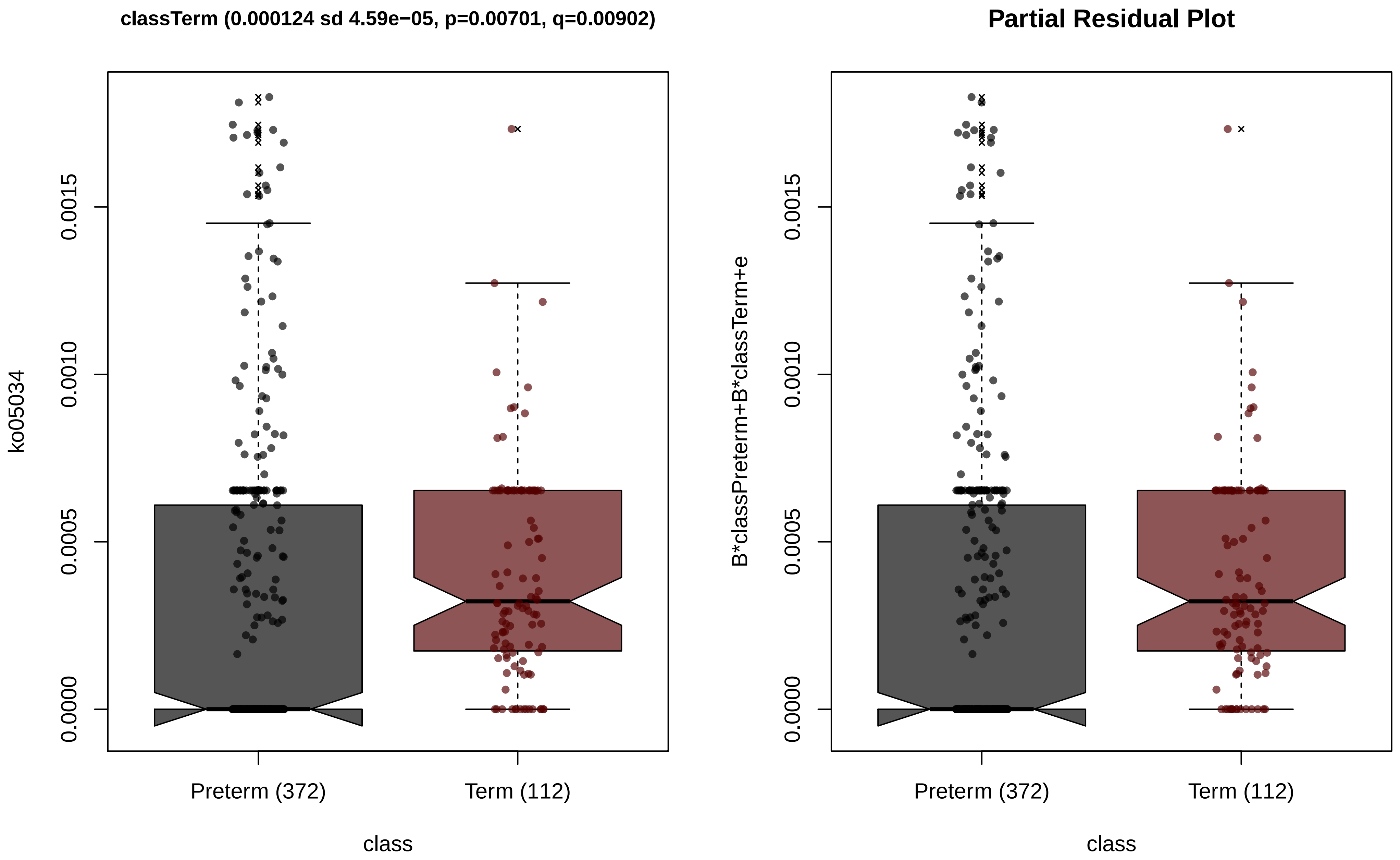

classTerm (0.000124 sd 4.59e−05, p=0.00701, q=0.00902)
Partial Residual Plot
ko05034
B*classPreterm+B*classTerm+e
0.0000
0.0005
0.0010
0.0015
Preterm (372)
Term (112)
class

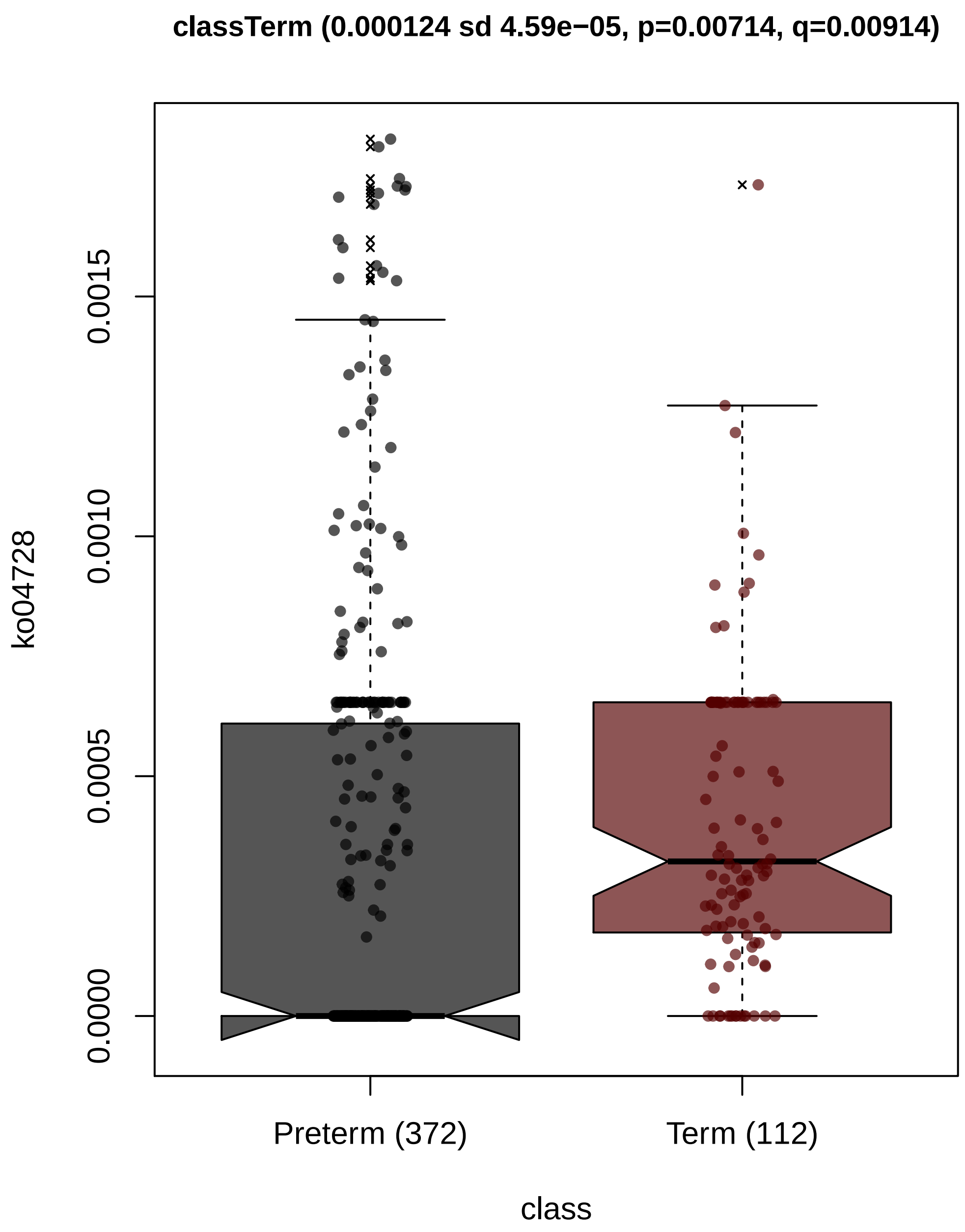

classTerm (0.000124 sd 4.59e−05, p=0.00714, q=0.00914)
ko04728
0.0000
0.0005
0.0010
0.0015
Preterm (372)
Term (112)
class

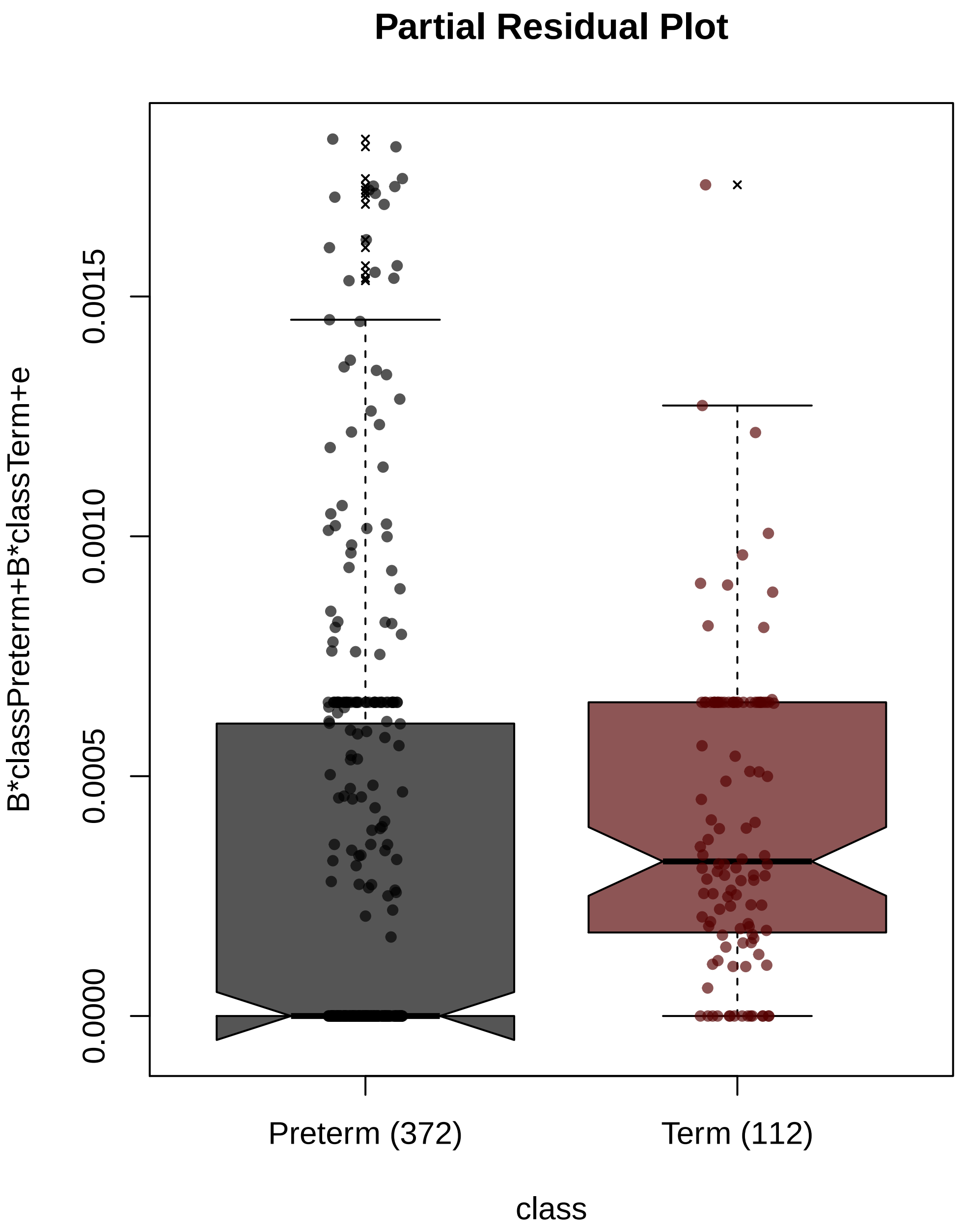

Partial Residual Plot
B*classPreterm+B*classTerm+e
0.0000
0.0005
0.0010
0.0015
Preterm (372)
Term (112)
class

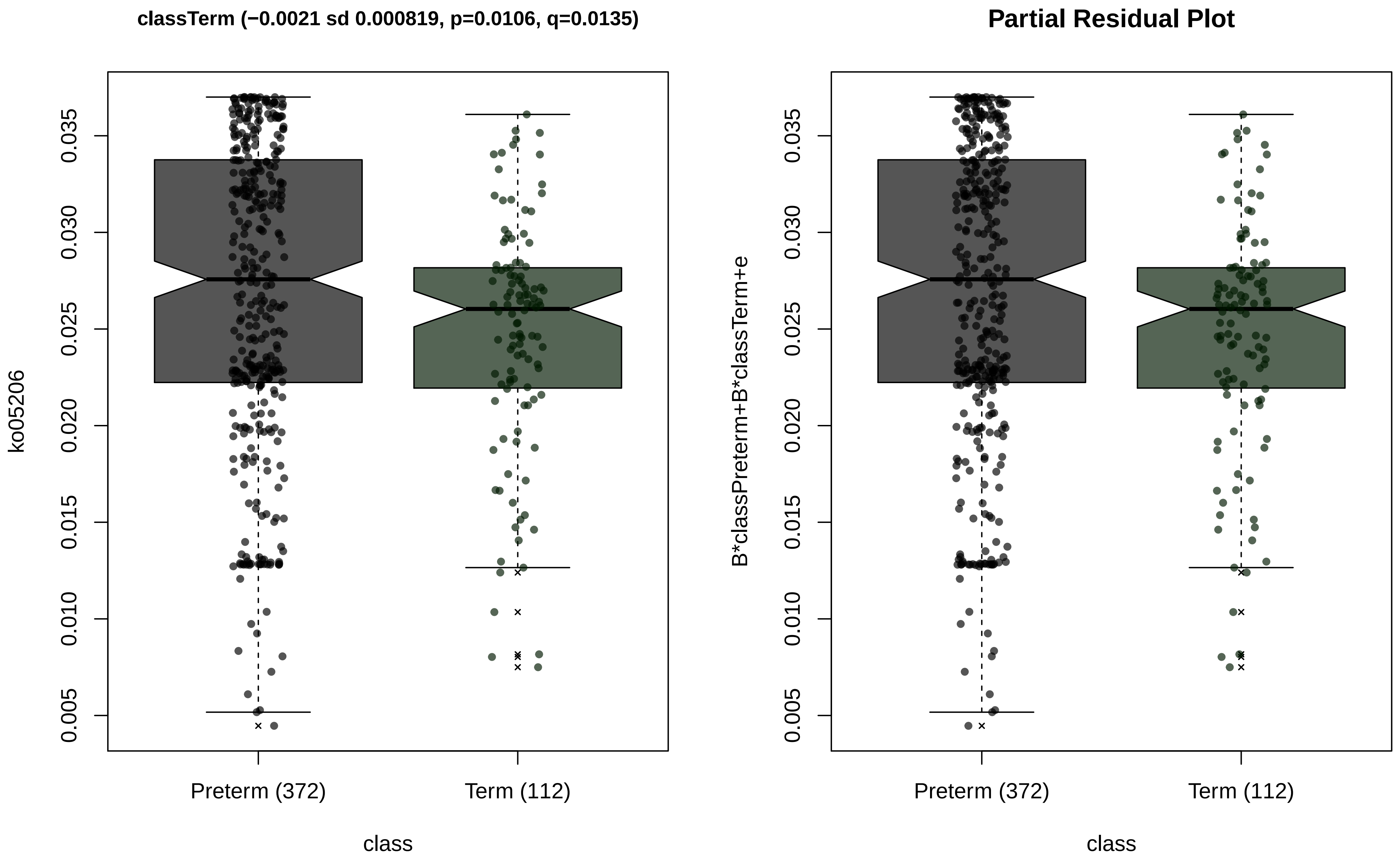

classTerm (−0.0021 sd 0.000819, p=0.0106, q=0.0135)
Partial Residual Plot
ko05206
B*classPreterm+B*classTerm+e
0.005
0.010
0.015
0.020
0.025
0.030
0.035
Preterm (372)
Term (112)
class

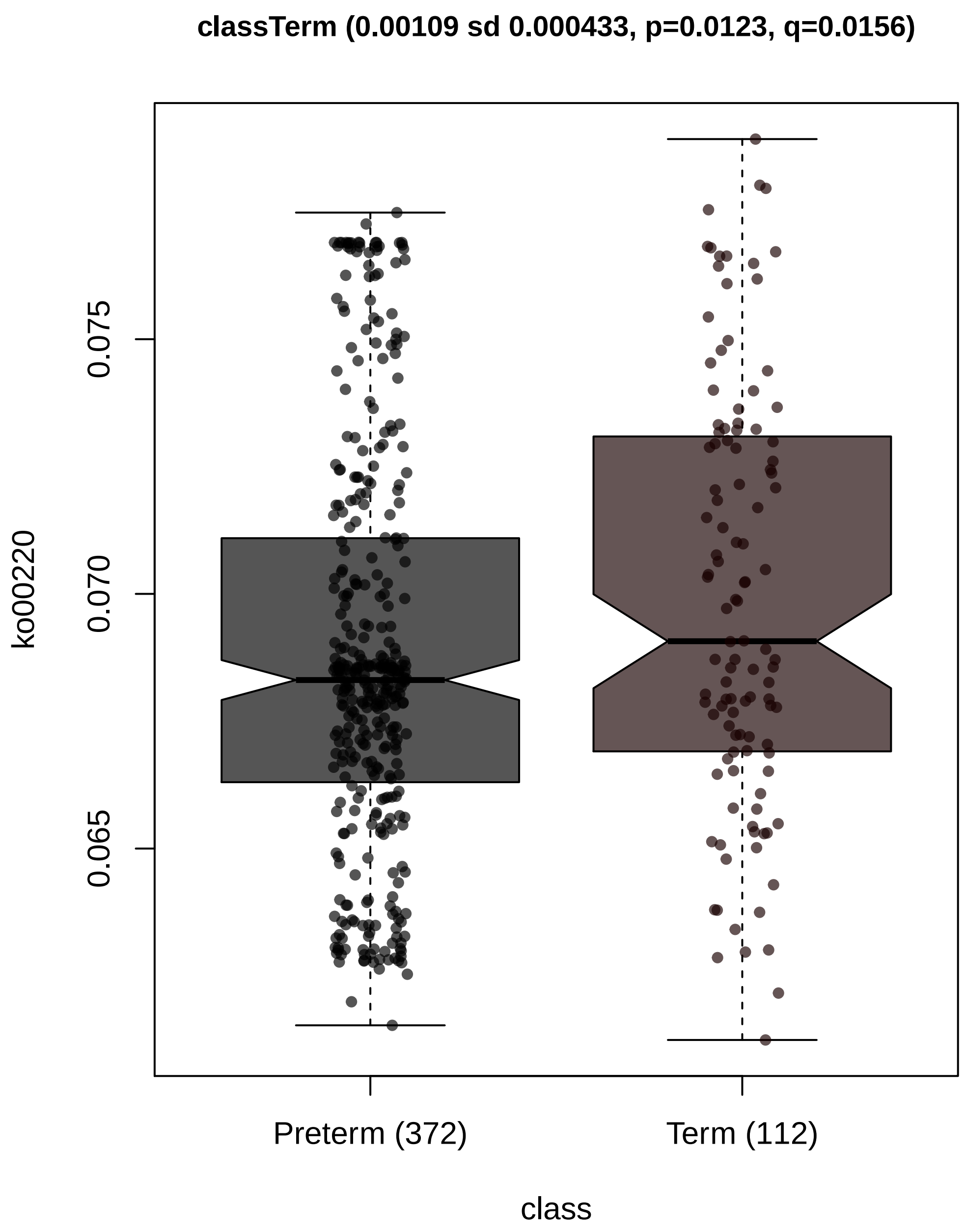

classTerm (0.00109 sd 0.000433, p=0.0123, q=0.0156)
ko00220
0.065
0.070
0.075
Preterm (372)
Term (112)
class

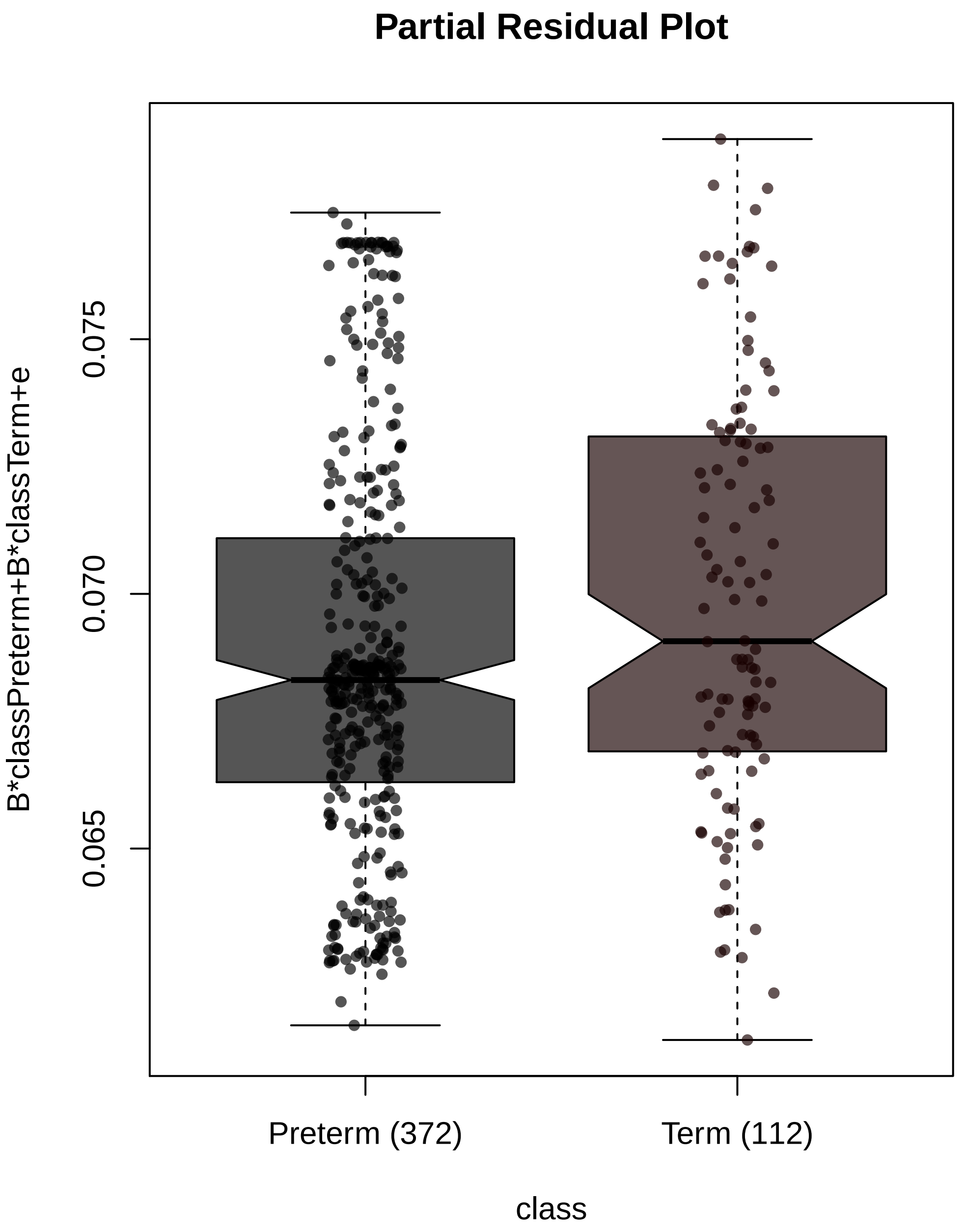

Partial Residual Plot
B*classPreterm+B*classTerm+e
0.065
0.070
0.075
Preterm (372)
Term (112)
class

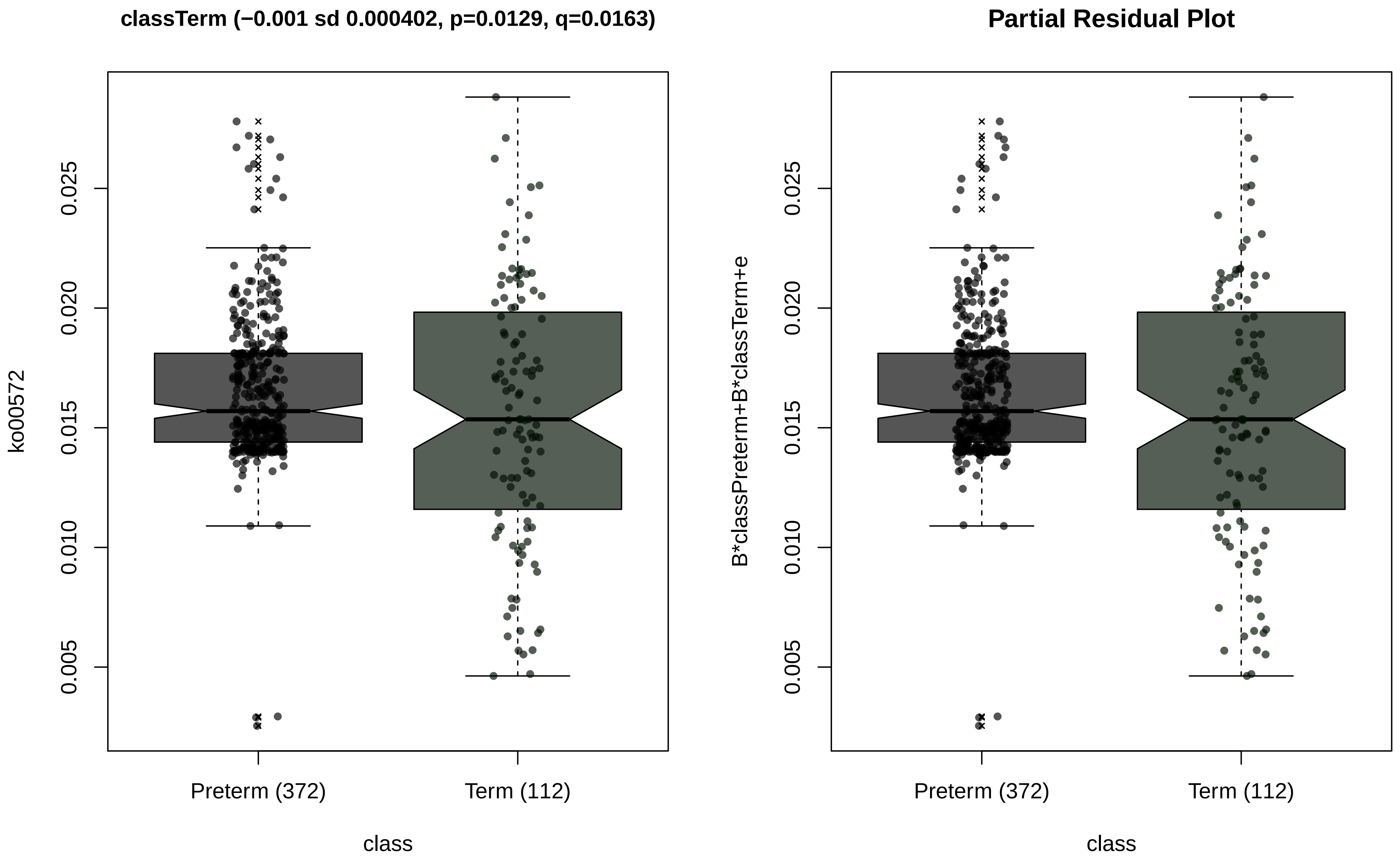

classTerm (−0.001 sd 0.000402, p=0.0129, q=0.0163)
ko00572
0.005
0.010
0.015
0.020
0.025
Preterm (372)
Term (112)
class
Partial Residual Plot
B*classPreterm+B*classTerm+e
0.005
0.010
0.015
0.020
0.025
Preterm (372)
Term (112)
class

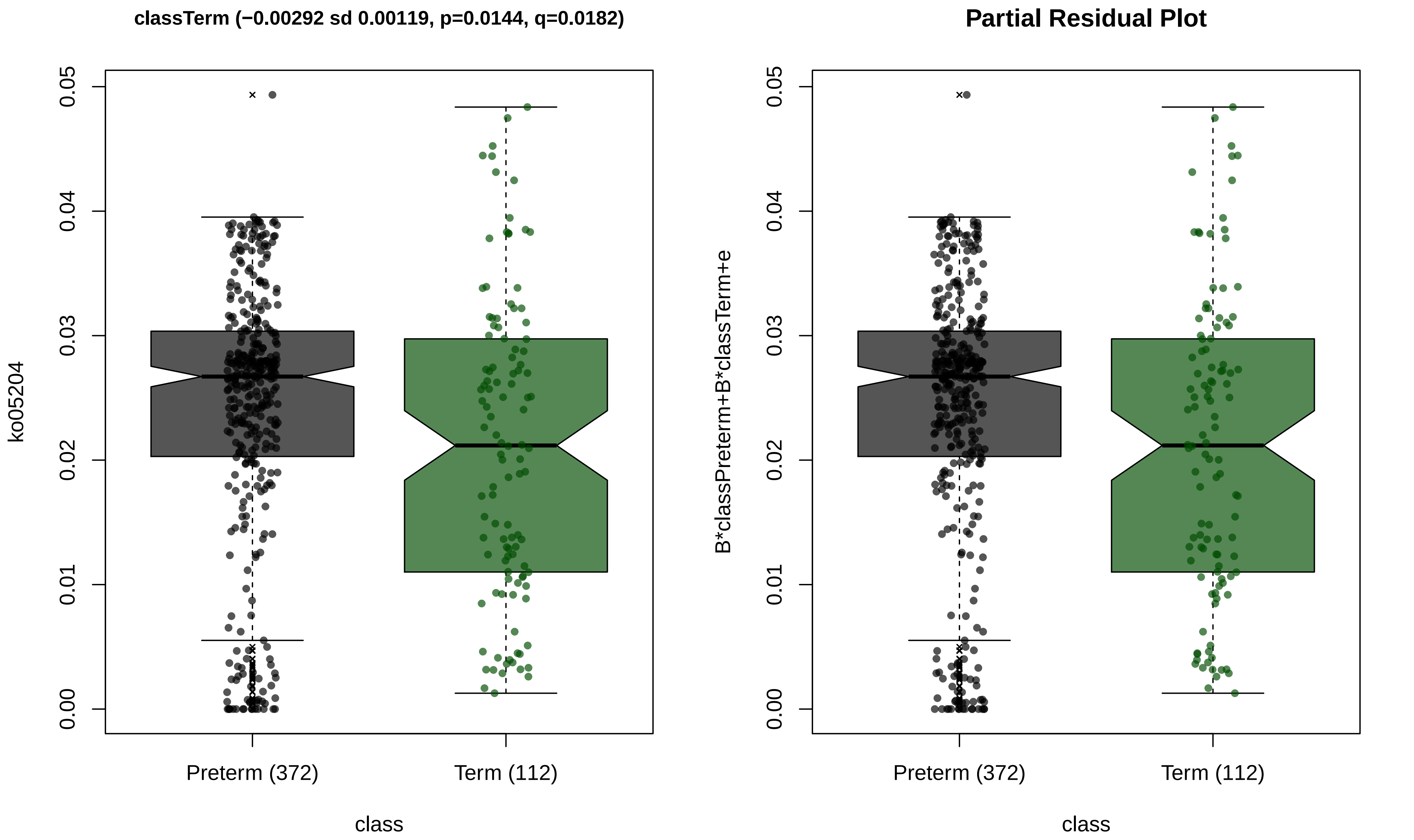

classTerm (−0.00292 sd 0.00119, p=0.0144, q=0.0182)
Partial Residual Plot
ko05204
B*classPreterm+B*classTerm+e
0.00
0.01
0.02
0.03
0.04
0.05
Preterm (372)
Term (112)
class

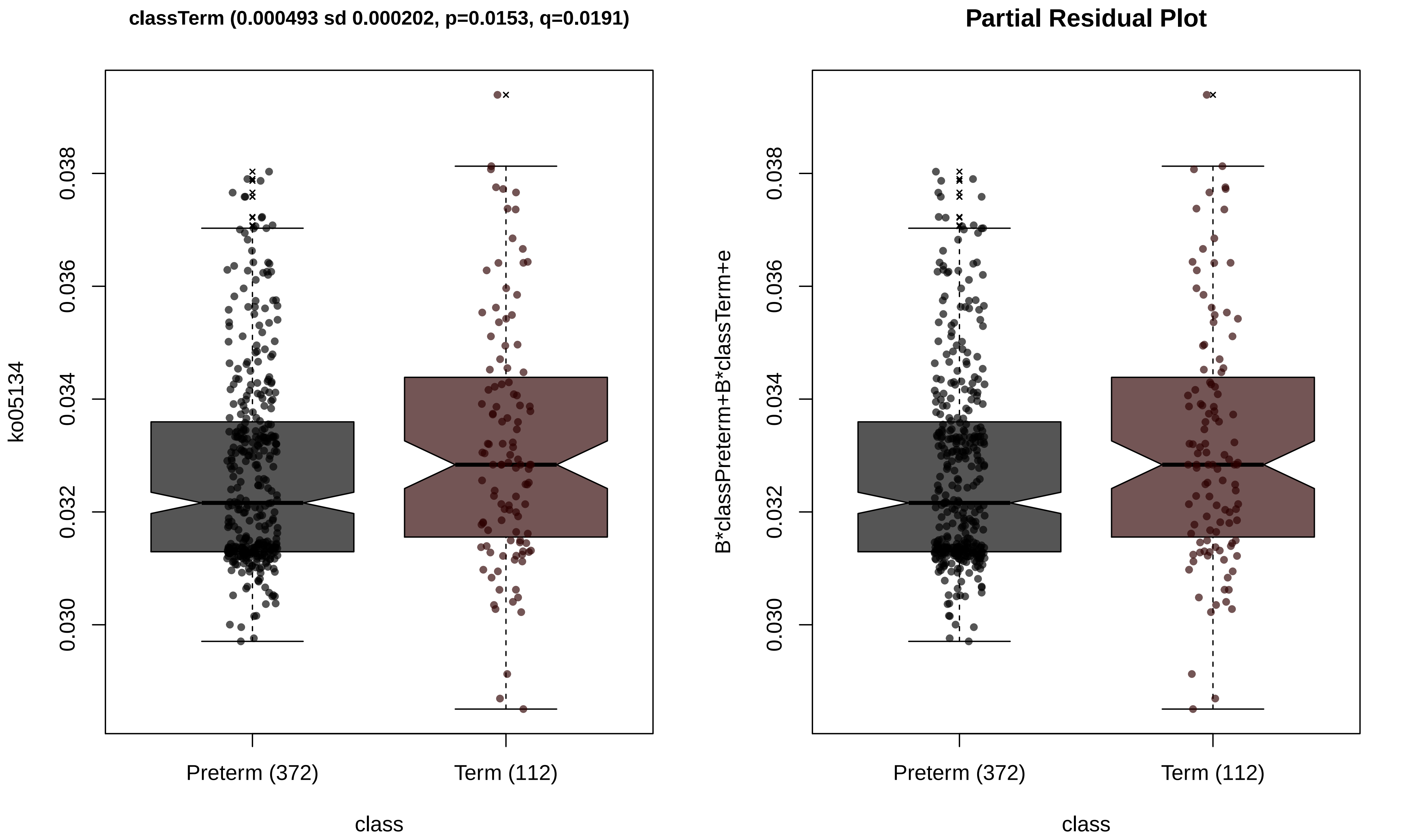

classTerm (0.000493 sd 0.000202, p=0.0153, q=0.0191)
Partial Residual Plot
ko05134
B*classPreterm+B*classTerm+e
0.030
0.032
0.034
0.036
0.038
Preterm (372)
Term (112)
class

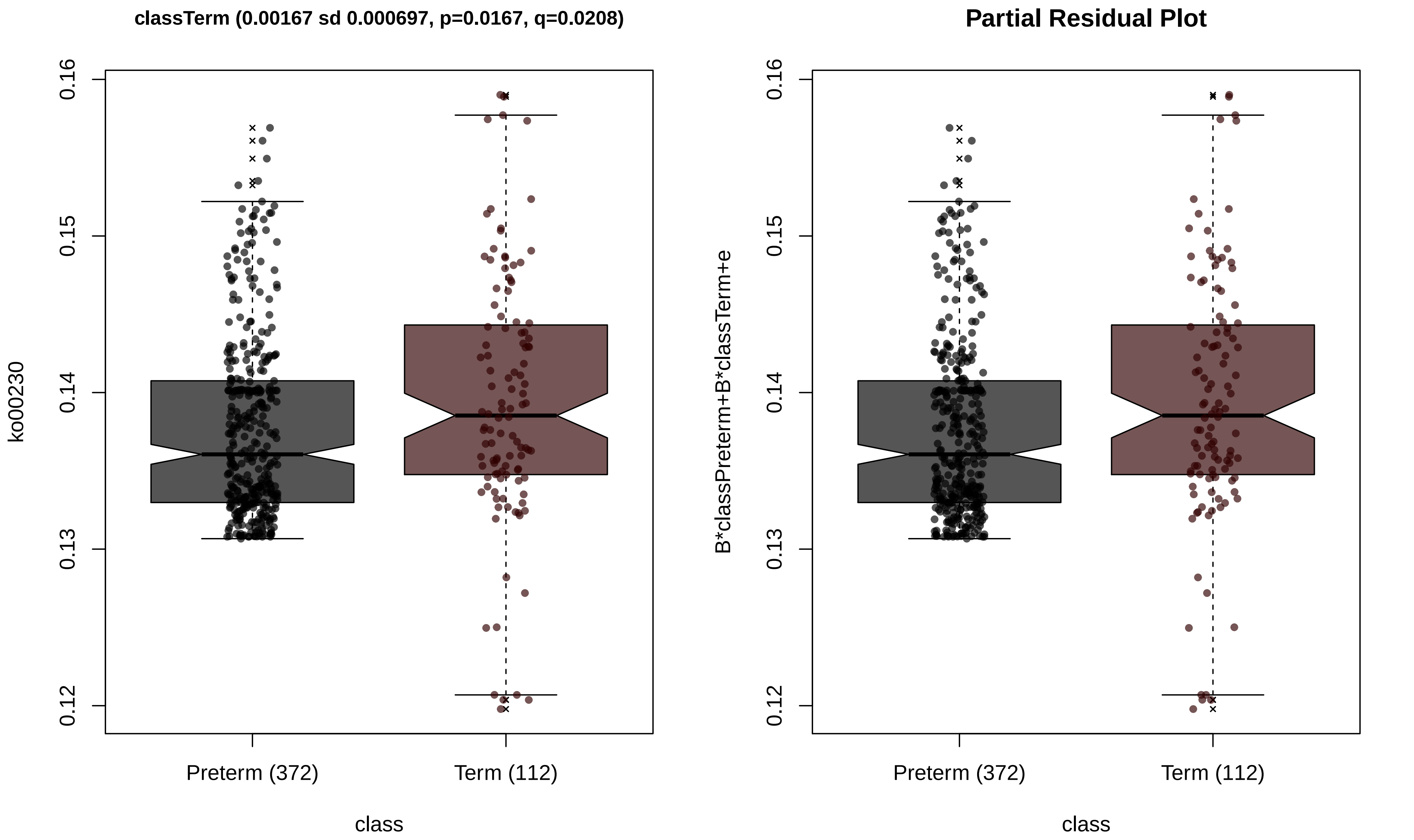

classTerm (0.00167 sd 0.000697, p=0.0167, q=0.0208)
ko00230
0.12
0.13
0.14
0.15
0.16
Preterm (372)
Term (112)
class
Partial Residual Plot
B*classPreterm+B*classTerm+e
0.12
0.13
0.14
0.15
0.16
Preterm (372)
Term (112)
class

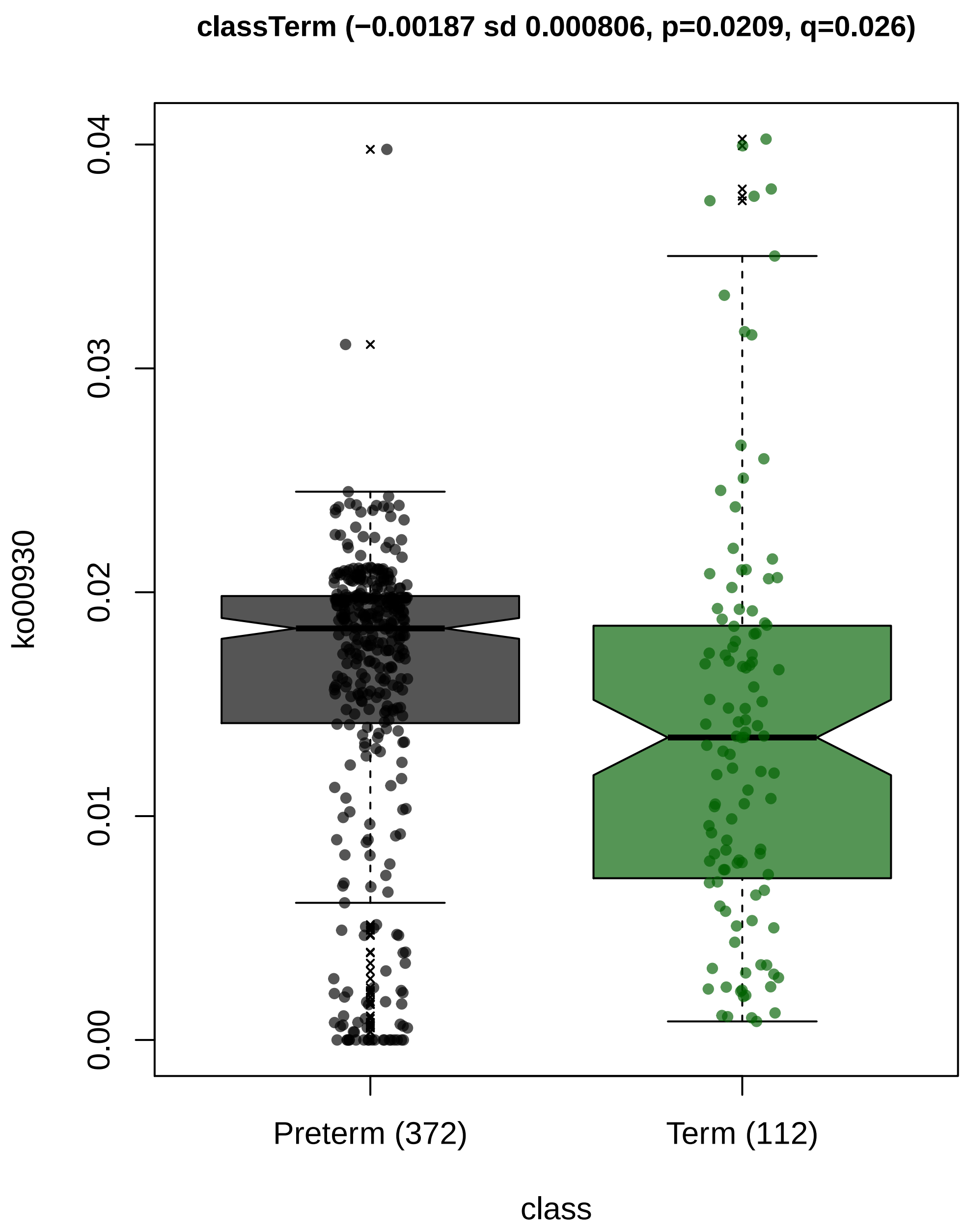

classTerm (−0.00187 sd 0.000806, p=0.0209, q=0.026)
ko00930
0.00
0.01
0.02
0.03
0.04
Preterm (372)
Term (112)
class

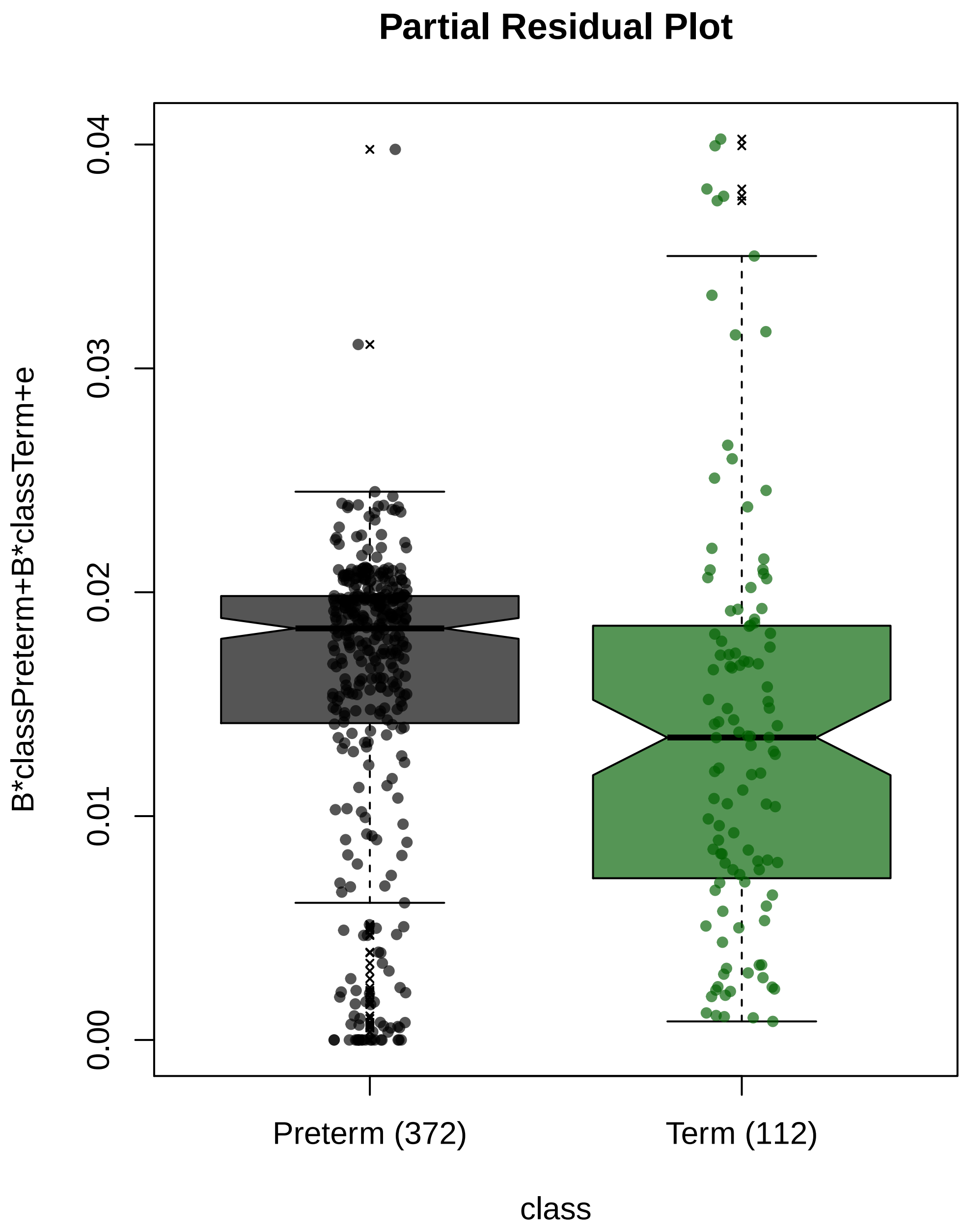

Partial Residual Plot
B*classPreterm+B*classTerm+e
0.00
0.01
0.02
0.03
0.04
Preterm (372)
Term (112)
class

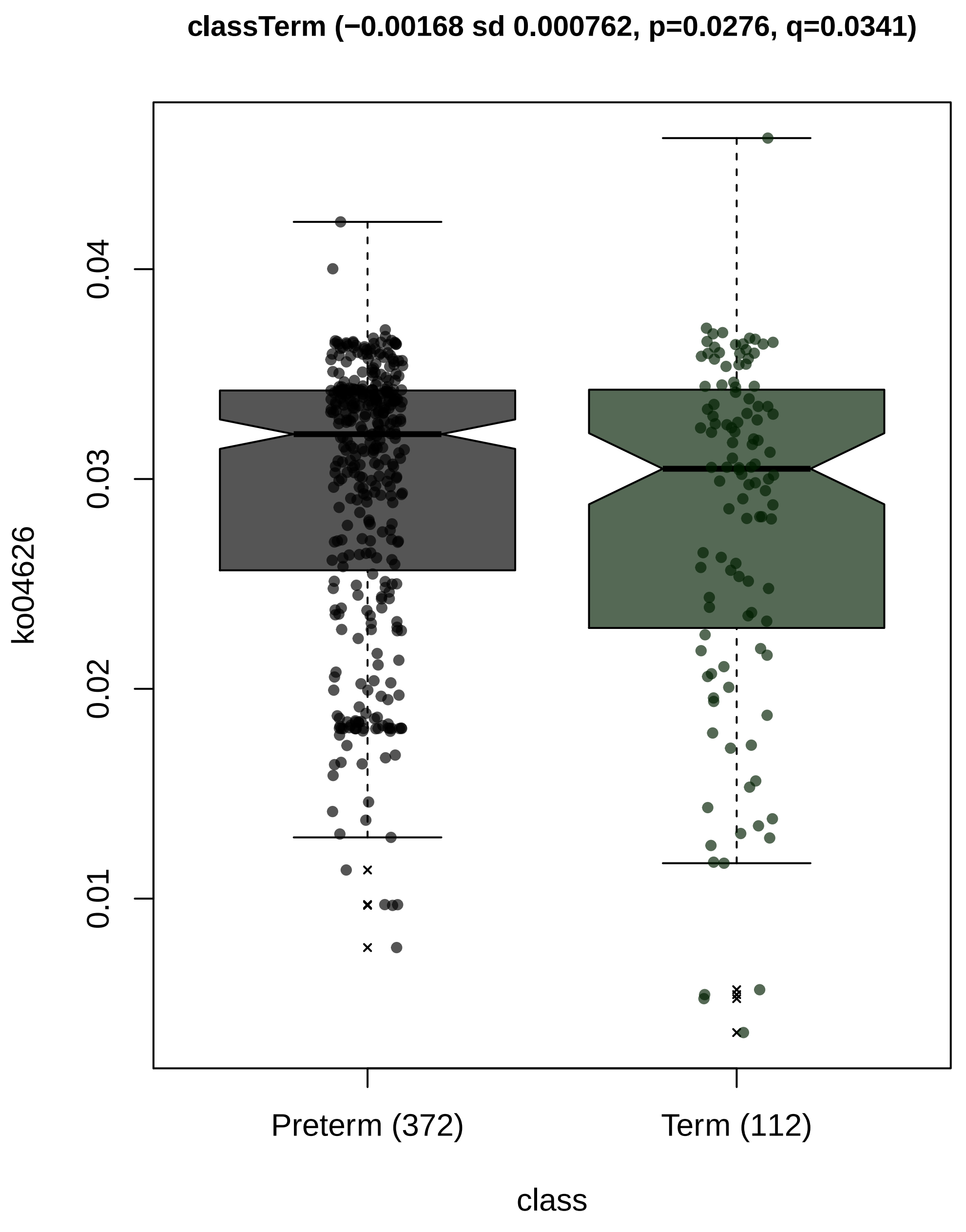

classTerm (−0.00168 sd 0.000762, p=0.0276, q=0.0341)
ko04626
0.01
0.02
0.03
0.04
Preterm (372)
Term (112)
class

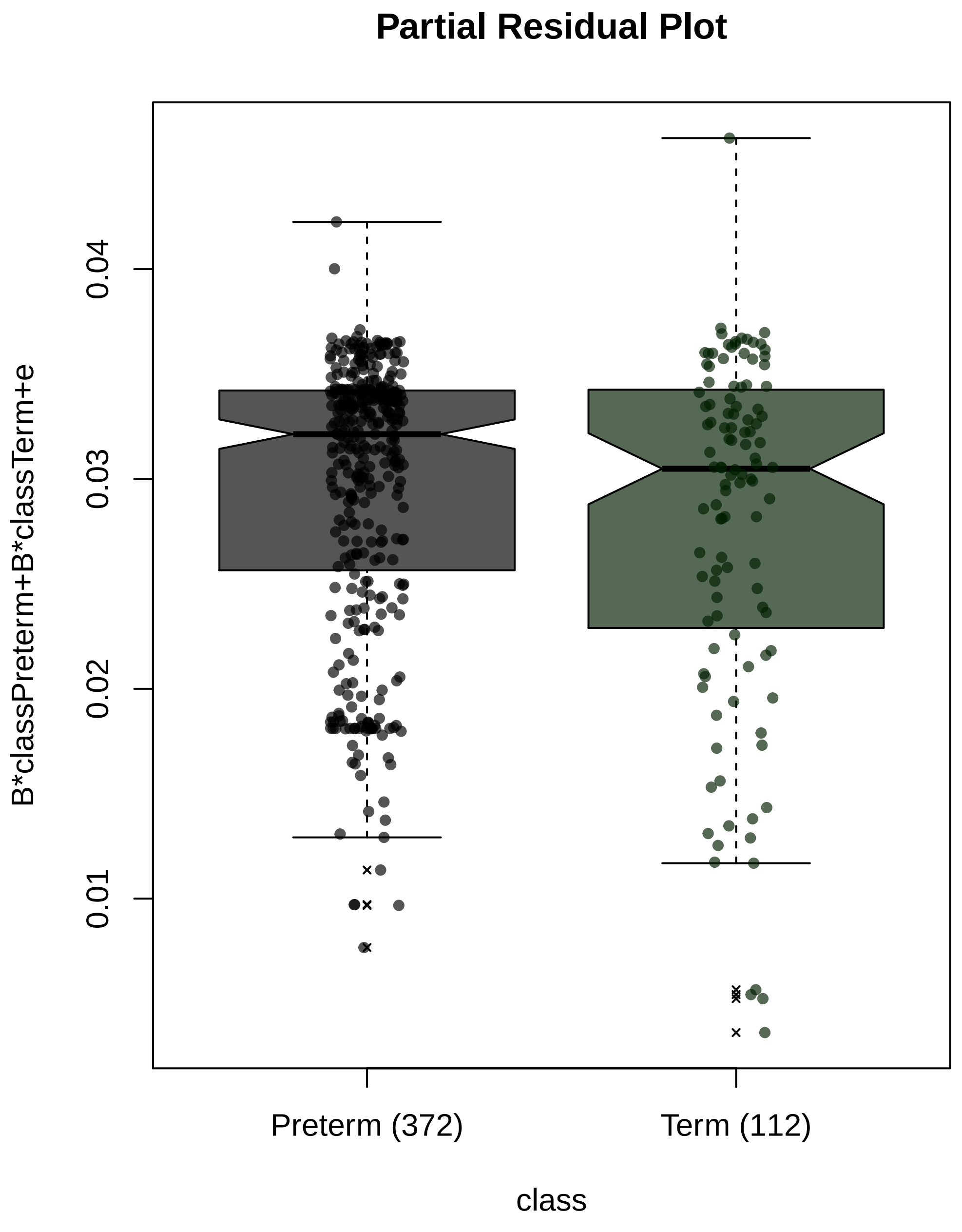

Partial Residual Plot
B*classPreterm+B*classTerm+e
0.01
0.02
0.03
0.04
Preterm (372)
Term (112)
class

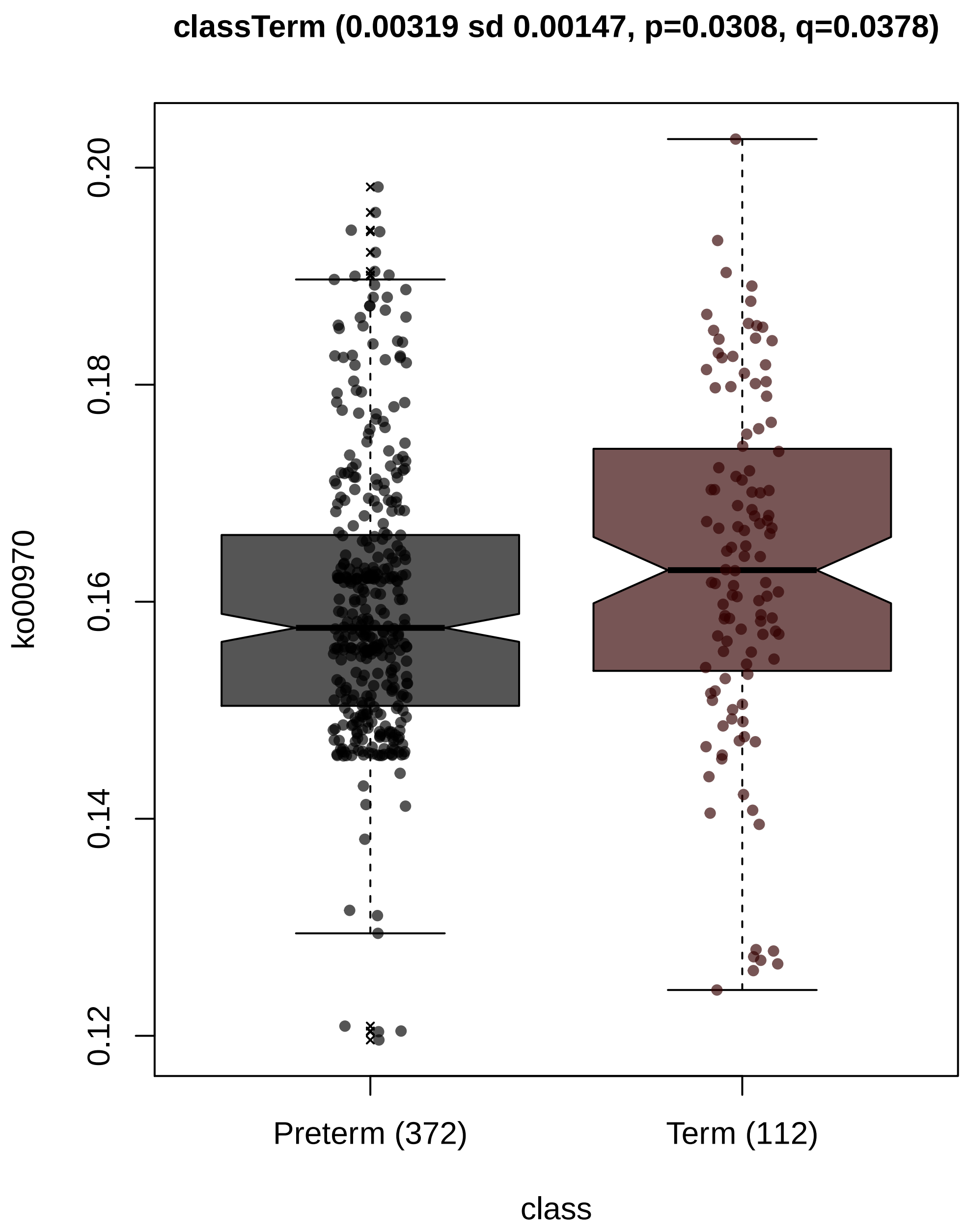
classTerm (0.00319 sd 0.00147, p=0.0308, q=0.0378)
ko00970
0.20
0.18
0.16
0.14
0.12
Preterm (372)
Term (112)
class

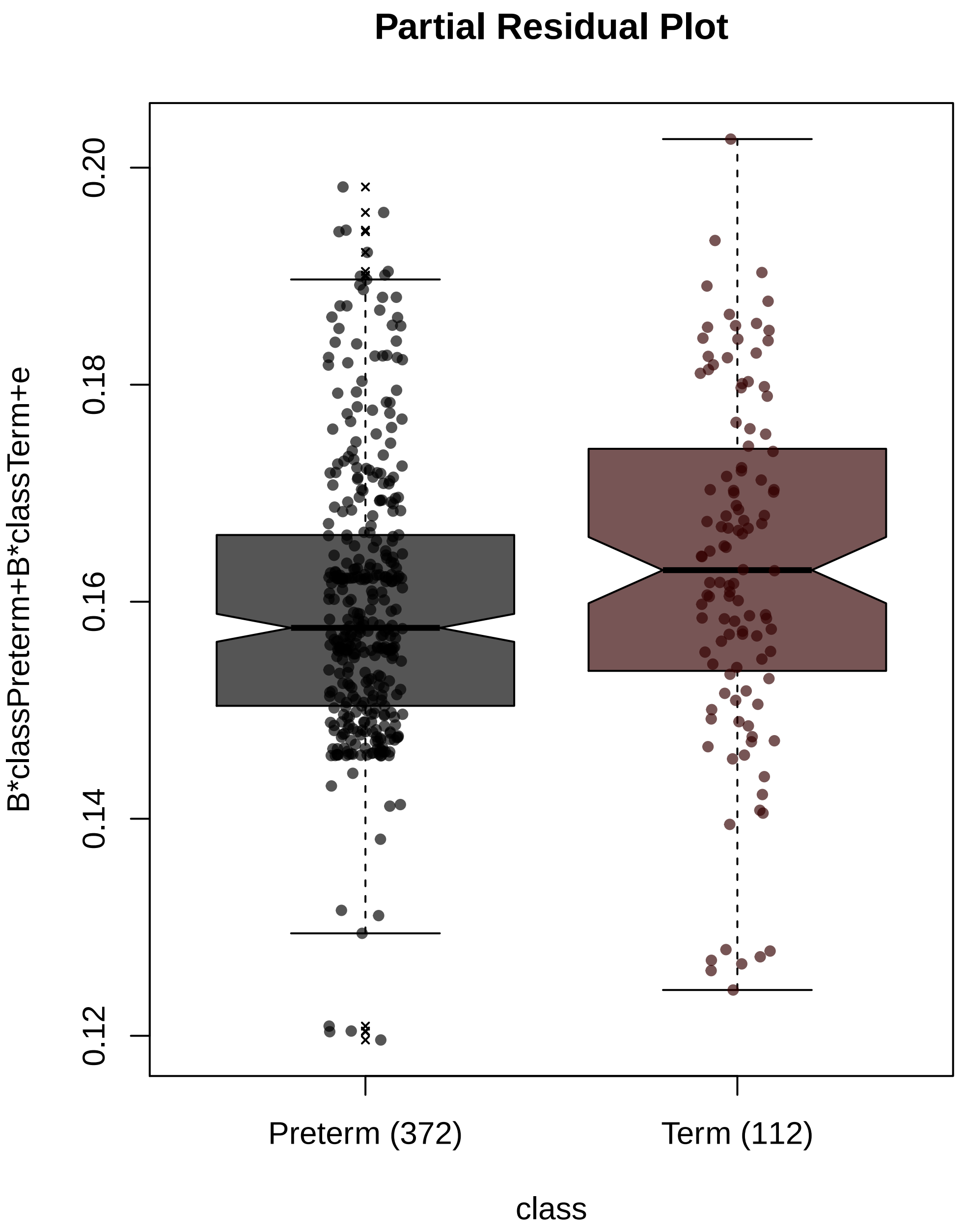
Partial Residual Plot
B*classPreterm+B*classTerm+e
0.20
0.18
0.16
0.14
0.12
Preterm (372)
Term (112)
class

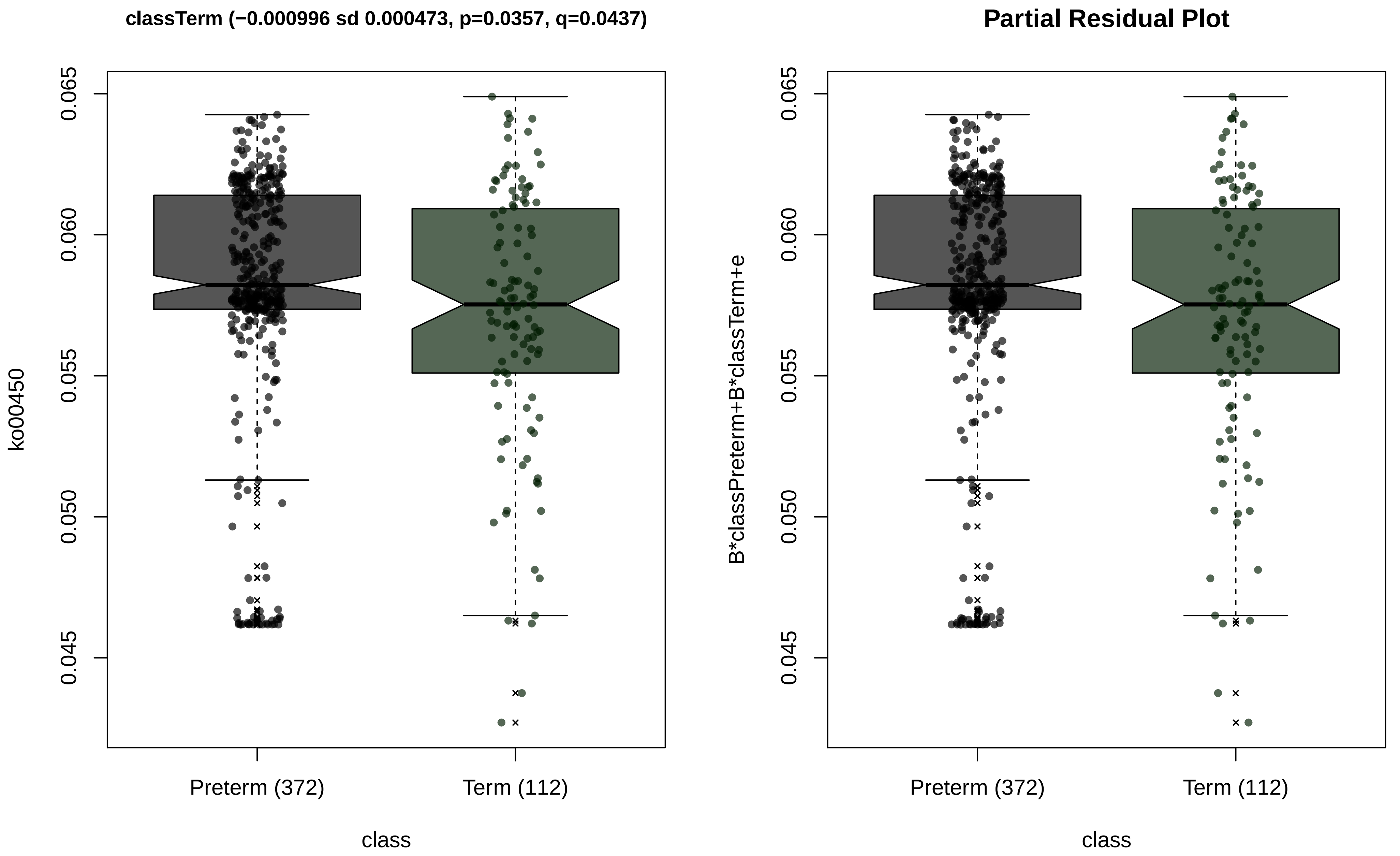
classTerm (−0.000996 sd 0.000473, p=0.0357, q=0.0437)
ko00450
0.065
0.060
0.055
0.050
0.045
Preterm (372)
Term (112)
class
Partial Residual Plot
B*classPreterm+B*classTerm+e
0.065
0.060
0.055
0.050
0.045
Preterm (372)
Term (112)
class
