# Supplementary material for: Predicted Metabolic Pathway Distributions in Stool Bacteria in Very-Low-Birth-Weight Infants: Potential Relationships with NICU Faltered Growth
Source: Nutrients. 2020 May 8;12(5):1345. doi: 10.3390/nu12051345 (PMC7284701; doi:10.3390/nu12051345)

Supplementary data

Metabolic Pathway Distributions in Very Low Birth Weight Infants: Potential Relationships with NICU Faltered Growth

S6. PCA plots between VLBW infants and term infants by week. The PCA plots are made using the prcomp function in R. Each plot is based on the pathway abundances for the preterms and terms for each week. They are 1) week1, 2) week2, 3)week3, 4) week4, 5)week 5, and 6) week6

1) Week 1

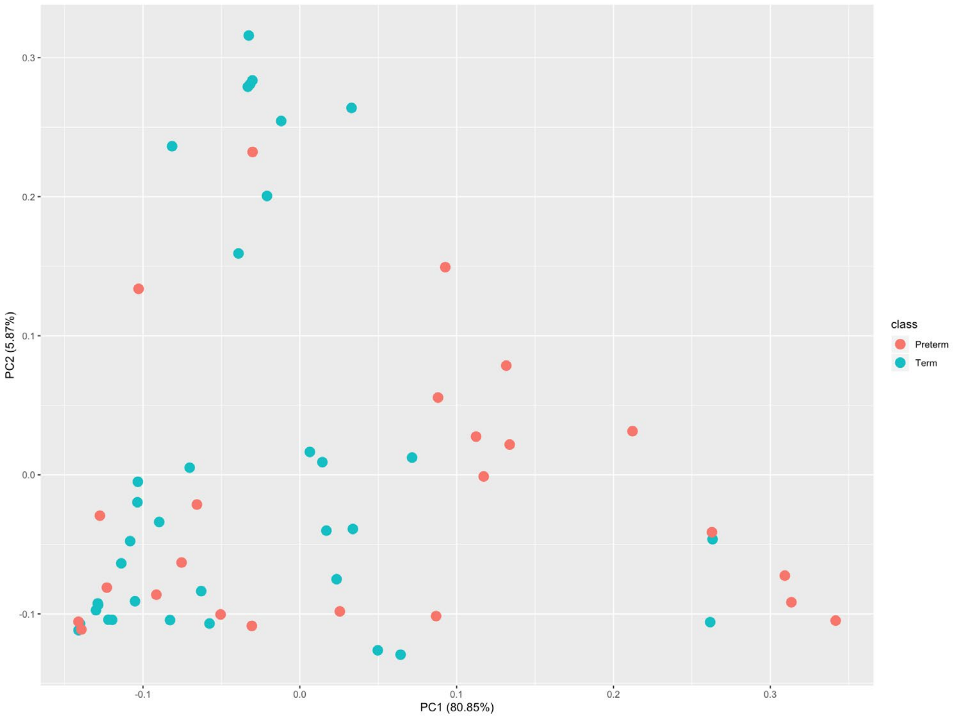

2) Week 2

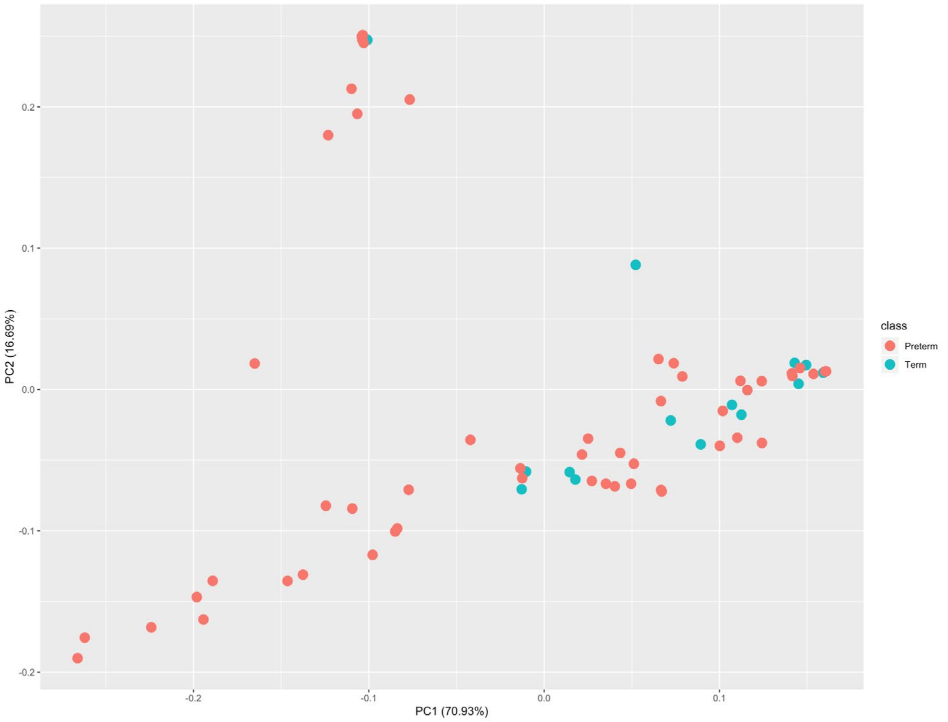

3) Week 3

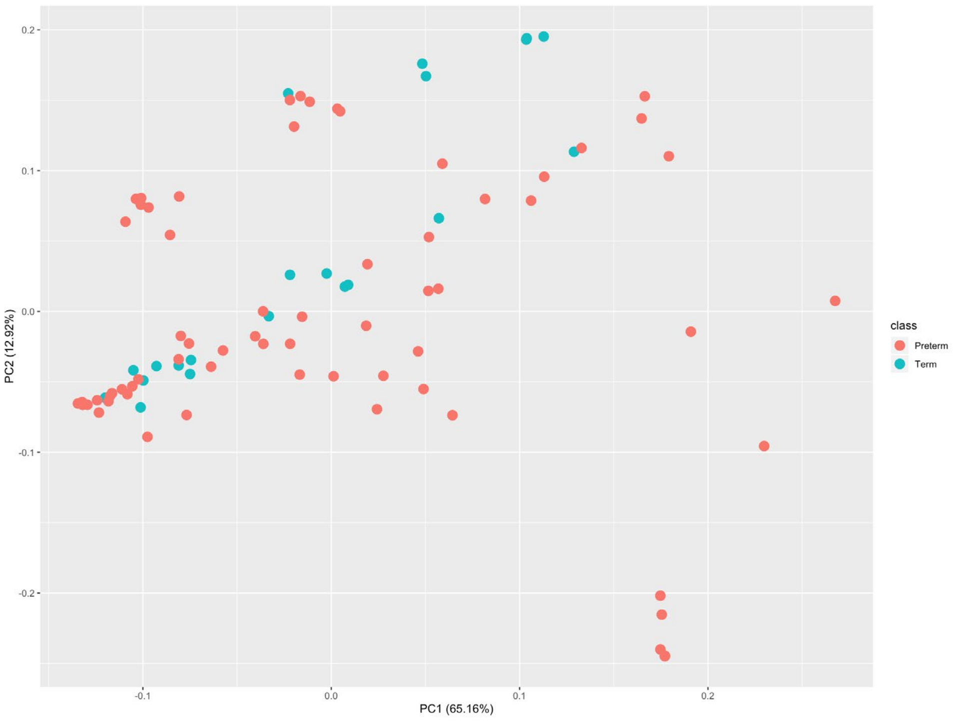

4) Week 4

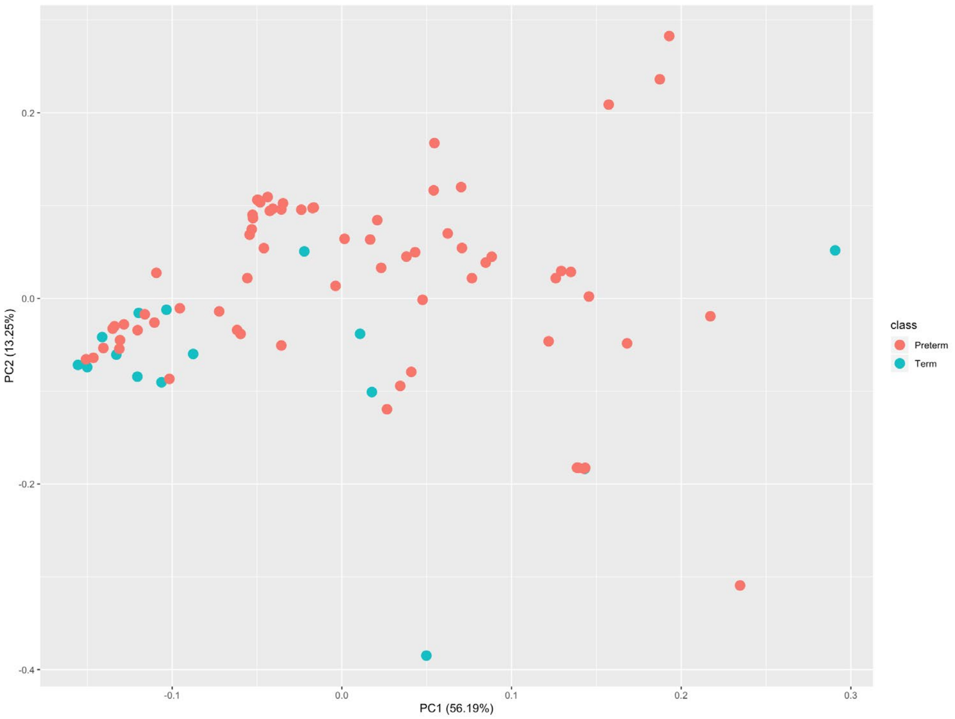

5) Week 5

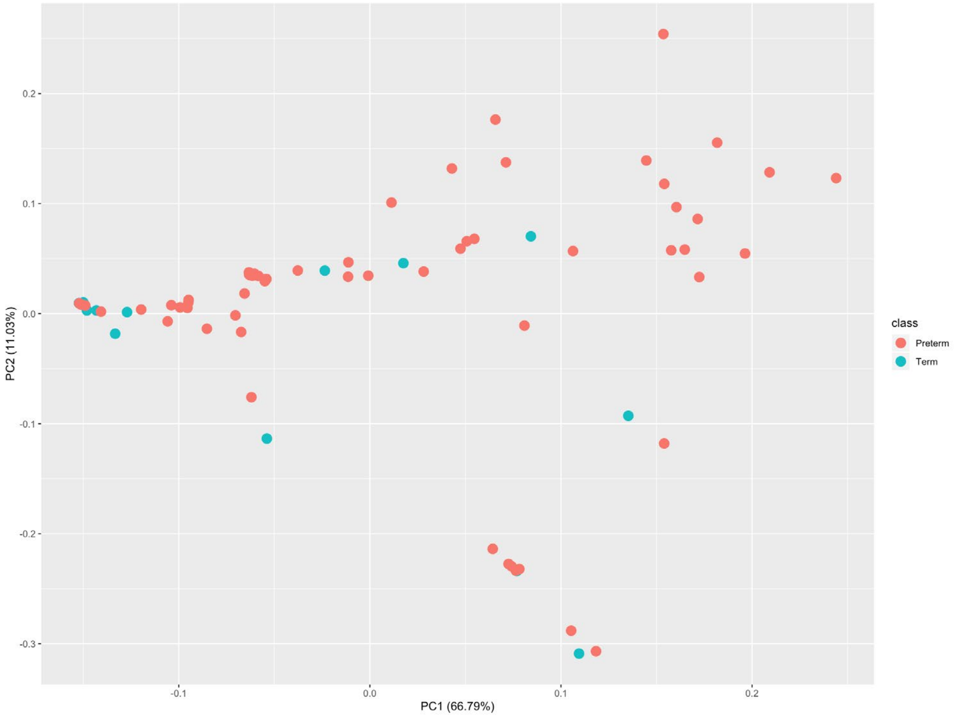

6) Week 6

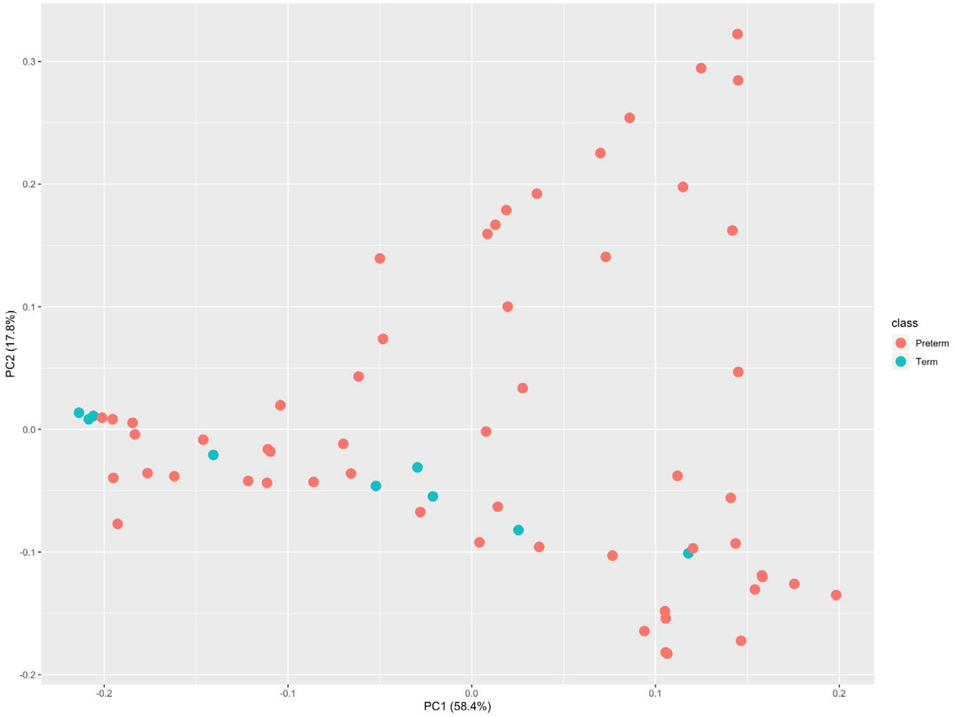

Supplement: Supplementary file 1 [file nutrients-12-01345-s001.zip › nutrients-769405-supplementary/Supplementary Meterials/Table S2_Supplementary_data.pdf]
